# The Global Flourishing Study: Study Profile and Initial Results on Flourishing

**DOI:** 10.1038/s44220-025-00423-5

**Published:** 2025-04-30

**Authors:** Tyler J. VanderWeele, Byron R. Johnson, Piotr T. Bialowolski, Rebecca Bonhag, Matt Bradshaw, Thomas Breedlove, Brendan Case, Ying Chen, Zhuo Job Chen, Victor Counted, Richard G. Cowden, Pedro Antonio de la Rosa, Chris Felton, Alex Fogleman, Cristina Gibson, Nikolitsa Grigoropoulou, Craig Gundersen, Sung Joon Jang, Kathryn A. Johnson, Blake Victor Kent, Eric S. Kim, Young-Il Kim, Hayami K. Koga, Matthew T. Lee, Noemi Le Pertel, Tim Lomas, Katelyn N. G. Long, Lucía Macchia, Christos A. Makridis, Lesley Markham, Julia S. Nakamura, Nicholas Norman-Krause, Chukwuemeka N. Okafor, Sakurako S. Okuzono, Suzanne T. Ouyang, R. Noah Padgett, Jason Paltzer, James L. Ritchie-Dunham, Zacc Ritter, Koichiro Shiba, Rajesh Srinivasan, John Ssozi, Dorota Weziak-Bialowolska, Renae Wilkinson, Robert D. Woodberry, Jennifer Wortham, George Yancey

**Affiliations:** 1 Harvard University, Cambridge, MA USA; 2 Baylor University, Waco, TX USA; 3 Kozminski University, Warsaw, Poland; 4 University of North Carolina at Charlotte, Charlotte, NC USA; 5 Regent University, Virginia Beach, VA USA; 6 University of Navarra, Pamplona, Spain; 7 Pepperdine University, Malibu, CA USA; 8 University of Bremen, Bremen, Germany; 9 Arizona State University, Tempe, AZ USA; 10 Westmont College, Santa Barbara, CA USA; 11 University of British Columbia, Vancouver, British Columbia Canada; 12 George Fox University, Newberg, OR USA; 13 Institute for Global Flourishing, New York, NY USA; 14 City University of London, London, UK; 15 Center for Open Science, Charlottesville, VA USA; 16 Belmont University, Nashville, TN USA; 17 University of Texas Health Science Center at San Antonio, San Antonio, TX USA; 18 Wisconsin Lutheran College, Wauwatosa, WI USA; 19 University of Texas at Austin, Austin, TX USA; 20 Gallup, Washington, DC USA; 21 Boston University, Boston, MA USA

**Keywords:** Social sciences, Sociology

## Abstract

The Global Flourishing Study is a longitudinal panel study of over 200,000 participants in 22 geographically and culturally diverse countries, spanning all six populated continents, with nationally representative sampling and intended annual survey data collection for 5 years to assess numerous aspects of flourishing and its possible determinants. The study is intended to expand our knowledge of the distribution and determinants of flourishing around the world. Relations between a composite flourishing index and numerous demographic characteristics are reported. Participants were also surveyed about their childhood experiences, which were analyzed to determine their associations with subsequent adult flourishing. Analyses are presented both across and within countries, and discussion is given as to how the demographic and childhood relationships vary by country and which patterns appear to be universal versus culturally specific. Brief comment is also given on the results of a whole series of papers in the Global Flourishing Study Special Collection, employing similar analyses, but with more-specific aspects of well-being. The Global Flourishing Study expands our knowledge of the distribution and determinants of well-being and provides foundational knowledge for the promotion of societal flourishing.

## Abstract (web summary)

The Global Flourishing Study provides a comprehensive view of the distribution and determinants of well-being by assessing domains such as health, happiness, meaning, character, relationships and financial security. Initial findings reveal significant variations in flourishing across countries and demographic groups, with factors such as age, marital status and religious service attendance showing strong associations with well-being.

## Main

**Fig. 1:**
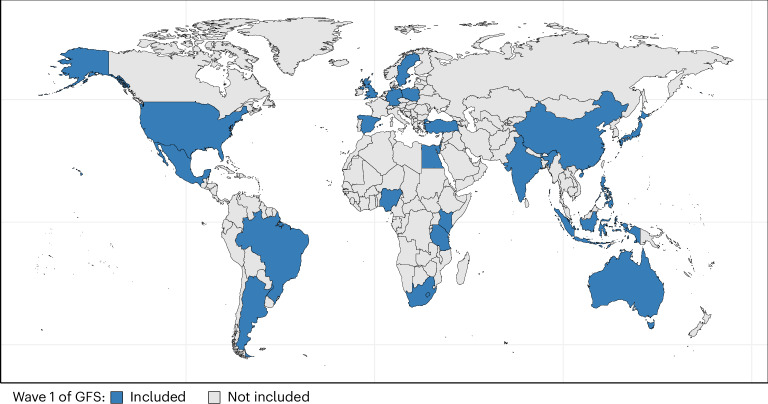
Countries included in Wave 1 of the GFS. Solid blue, included; light gray, not included.

Interest in questions of flourishing has expanded dramatically in recent years. This interest can be seen in various sectors, including psychology, economics, business, education, medicine, public health and public policy^1–10^. While this has led to an expansion in research, much remains unknown, especially as it pertains to flourishing on the global landscape. The research on well-being has been shaped largely by Western perspectives and has been carried out mostly in Western contexts. This work has thus been subject to some critique^11^, as indeed is the case with social–scientific research more generally^12^. Such limitations hinder our conceptions of, and knowledge concerning, well-being. The Global Flourishing Study (GFS) was conceived as a large, open-access, 5-year longitudinal panel research study of 22 countries (see Fig. 1), spanning all six populated continents, with nationally representative sampling in each country, to study the distribution and determinants of well-being, to advance our knowledge of flourishing in general and especially in non-Western contexts and to uncover what patterns are culturally specific and which seem more universal. Compared with other cross-national studies of well-being such as the World Values Survey or the Gallup World Poll (GWP)/World Happiness Report^13^, the GFS aims to supplement and expand upon these by both (1) providing longitudinal panel data on the same cohort of individuals over time and (2) providing a broader range of assessments on well-being or flourishing.

Flourishing is an expansive concept^1,2,5,14,15^, and the working definition underpinning the GFS has been ‘the relative attainment of a state in which all aspects of a person’s life are good, including the contexts in which that person lives.’^5,16^ Several aspects of this definition are important. First, flourishing is multidimensional—it concerns all aspects of a person’s life. One may be flourishing in certain ways but not in others. No assessment of flourishing will ever fully measure flourishing, only aspects of it. Second, flourishing may be conceived of as an ideal, but it also concerns the ‘relative attainment’ of that ideal^17^. We are never perfectly flourishing in this life, and there is always room for improvement. Third, flourishing concerns both objective and subjective aspects of life, although subjective aspects are more amenable to survey research. Fourth, the understanding of what is ‘good’ will vary across cultures and contexts, but there is arguably a great deal of common ground as well, and such common ground is a reasonable starting point for measurement^5,18^. Finally, flourishing includes the contexts in which a person lives; such contexts include one’s communities and environment. While the terms ‘flourishing’ and ‘well-being’ are often used interchangeably, flourishing arguably has a connotation of also having the environment itself being conducive to growth and being a part of one’s flourishing. The community’s well-being is a part of one’s own flourishing—a person participates in the common good of the community. While well-being might be defined as ‘the relative attainment of a state in which all aspects of a person’s life are good, as they pertain to that individual,’ flourishing also includes the well-being of the community and environment. However, since individual aspects of flourishing effectively constitute well-being, the two terms will, in many contexts, often be used interchangeably. There may also often be greater consensus across cultures around what is desirable for individual well-being than in understandings concerning what constitutes the right type of community or government, and so the composite flourishing index considered in the following focuses on those individual aspects^5^. We do, however, also offer further comment on analyses using more community-related assessments.

This Article provides a description of the Global Flourishing Study, including the nature of the sample and data collection and a description of the methodology for the development of the questionnaire, the study design and survey sampling, and the analytic approaches guiding many of the initial analyses of the data. Analyses concerning composite assessments of flourishing are presented as to how flourishing assessments vary across demographic groups and countries and how various types of childhood experiences relate to adult flourishing. Comment is offered on a range of other analyses and studies arising out of the first wave of the GFS data, and discussion given to the study’s importance and promotion of flourishing in a global context.

With the concept of flourishing being expansive, there is no unique or fully comprehensive way to partition ‘all aspects of a person’s life.’ Understandably, numerous well-being classifications have thus emerged^1,2,5,14,19^. While the GFS included questions from the flourishing conceptualization of ref. ^5^, the survey development process^20,21^ incorporated numerous cross-cultural perspectives and questions extending well beyond those proposed in ref. ^5^. In the following presentation of the initial GFS results, we roughly follow the flourishing domains of ref. ^5^—health, happiness, meaning, character, relationships and financial security. However, the GFS data and analyses do not presuppose the priority of these domains, and other researchers can certainly recategorize the items.

These aspects of well-being—health, happiness, meaning, character, relationships and financial security—may be understood as paradigmatic of broader well-being categories: physical well-being, emotional well-being, cognitive well-being, volitional well-being, social well-being and material well-being (see Table 1). A perhaps simpler division into domains derives from the World Health Organization’s definition^22^ of ‘health’ as a ‘state of complete physical, mental, and social well-being.’ The categories used here further divide mental well-being into emotional, cognitive and volitional well-being, corresponding to traditional and empirical division of the mind into emotions, intellect and will^23–28^. Social well-being might itself have further been divided into relational and communal well-being; in more expansive understandings of flourishing, communal well-being might itself be sub-divided into community, political and economic well-being. Again, there is no unique way to divide the conceptual space. Much has been written on including a spiritual dimension within the WHO definition^29^, thereby yielding, ‘a state of complete physical, mental, social, and spiritual well-being,’ which we have referred to elsewhere as the WHO+ definition^16,17^. It is, however, more difficult to attain consensus on understandings of spiritual well-being across the world religions, and we will make some remarks on this matter later in the Article. Finally, material and financial resources are sometimes taken as determinants of well-being and sometimes understood as constitutive elements of well-being^30–34^. These conceptualizations rightly vary depending on how well-being is defined. We do not take a definitive stance on this matter in the Article and present analyses alternatively including and excluding financial well-being.

**Table 1:** Well-being domains and definitions

Well-being domain	Paradigmatic examples	Definition
Physical well-being	Health	The relative attainment of a state in which all aspects of a person’s physical life are good
Emotional well-being	Happiness	The relative attainment of a state in which all aspects of a person’s emotional life are good
Cognitive well-being	Meaning	The relative attainment of a state in which all aspects of a person’s cognitive life are good
Volitional well-being	Character	The relative attainment of a state in which all aspects of a person’s volitional life are good
Social well-being	Relationships	The relative attainment of a state in which all aspects of a person’s social life are good
Material well-being	Financial security	The relative attainment of a state in which all aspects of a person’s financial and material life are good
Spiritual well-being	(Various)	The relative attainment of a state in which all aspects of a person’s spiritual life are good
Flourishing		The relative attainment of a state in which all aspects of a person’s life are good, including the contexts in which that person lives^5,16^

In what follows, we describe the GFS methodology, comment on results of the analyses for the composite flourishing index, offer brief comment on numerous papers concerned with more-specific aspects of well-being and conclude by offering discussion on the implications of the study and results for the promotion of flourishing.

## Results

### Composite flourishing

Table 2 presents nationally representative descriptive statistics of the observed sample. Slightly more than half were middle-aged (30–59 years old (53%)), women (51%), married (52%), employed (either for an employer or self-employed, 57%) and had between 9 and 15 years of education (57%). Most participants were born in the country in which they were surveyed (94%), and approximately one-third of participants attended religious services at least weekly (32%). Sample sizes in each country ranged from 1,473 (Turkey) to 38,312 (United States). Participant characteristics for each country are shown in Supplementary Tables 1a–22a.

**Table 2:** Nationally representative descriptive statistics of the observed sample

Characteristic	*N* = 202,898 (*n* (%)
**Age group (years)**	
18–24	27,007 (13)
25–29	20,700 (10)
30–39	40,256 (20)
40–49	34,464 (17)
50–59	31,793 (16)
60–69	27,763 (14)
70–79	16,776 (8.3)
80 or older	4,119 (2.0)
(Missing)	20 (<0.1)
**Gender**	
Male	98,411 (49)
Female	103,488 (51)
Other	602 (0.3)
(Missing)	397 (0.2)
**Current marital status**	
Married	107,354 (53)
Separated	5,195 (2.6)
Divorced	11,654 (5.7)
Widowed	9,823 (4.8)
Single, never married	52,115 (26)
Domestic partner	14,931 (7.4)
(Missing)	1,826 (0.9)
**Employment status**	
Employed for an employer	78,815 (39)
Self-employed	36,362 (18)
Retired	29,303 (14)
Student	10,726 (5.3)
Homemaker	21,677 (11)
Unemployed and looking for a job	16,790 (8.3)
None of these/other	8,431 (4.2)
(Missing)	793 (0.4)
**Current religious service attendance**	
More than 1 per week	26,537 (13)
1 per week	39,157 (19)
1–3 per month	19,749 (9.7)
A few times a year	41,436 (20)
Never	75,297 (37)
(Missing)	722 (0.4)
**Education (years)**	
Up to 8	45,078 (22)
9–15	115,097 (57)
16+	42,578 (21)
(Missing)	146 (<0.1)
**Immigration status**	
Born in this country	190,998 (94)
Born in another country	9,791 (4.8)
(Missing)	2,110 (1.0)
**Country**	
Argentina	6,724 (3.3)
Australia	3,844 (1.9)
Brazil	13,204 (6.5)
Egypt	4,729 (2.3)
Germany	9,506 (4.7)
Hong Kong (SAR of China)	3,012 (1.5)
India	12,765 (6.3)
Indonesia	6,992 (3.4)
Israel	3,669 (1.8)
Japan	20,543 (10)
Kenya	11,389 (5.6)
Mexico	5,776 (2.8)
Nigeria	6,827 (3.4)
Philippines	5,292 (2.6)
Poland	10,389 (5.1)
South Africa	2,651 (1.3)
Spain	6,290 (3.1)
Sweden	15,068 (7.4)
Tanzania	9,075 (4.5)
Turkey	1,473 (0.7)
United Kingdom	5,368 (2.6)
United States	38,312 (19)

SAR, Special Administrative Region.

Table 3 presents nationally representative descriptive statistics of the observed sample for retrospective assessments of childhood experiences. Most reported ‘very good’ or ‘somewhat good’ relationships with mother (89%) and father (80%) and that their parents were married (75%); a large proportion reported their family ‘lived comfortably’ (35%) or ‘got by’ (41%) financially during their childhood. About 18% of participants reported experiencing abuse and 16% feeling like an outsider growing up; most assessed their health as ‘excellent’ or ‘very good’ (64%) in childhood. A large proportion of participants attended religious services at least once per week during childhood (41%). The rate of missing data for the first wave of the GFS was low (<5% for any variable). Over all the items used as childhood predictors, the percentage of respondents with any missing data was only 12.9%.

**Table 3:** Nationally representative descriptive statistics of the observed sample for retrospective assessments of childhood experiences

Characteristic	*N* = 202,898 (*n* (%))
**Relationship with mother**	
Very good	127,836 (63)
Somewhat good	52,439 (26)
Somewhat bad	11,060 (5.5)
Very bad	4,642 (2.3)
Does not apply	5,965 (2.9)
(Missing)	956 (0.5)
**Relationship with father**	
Very good	107,742 (53)
Somewhat good	55,714 (27)
Somewhat bad	15,807 (7.8)
Very bad	8,278 (4.1)
Does not apply	13,985 (6.9)
(Missing)	1,372 (0.7)
**Parent marital status**	
Parents married	152,001 (75)
Divorced	17,726 (8.7)
Parents were never married	15,534 (7.7)
One or both parents had died	7,794 (3.8)
(Missing)	9,843 (4.9)
**Subjective financial status of family growing up**	
Lived comfortably	70,861 (35)
Got by	82,905 (41)
Found it difficult	35,852 (18)
Found it very difficult	12,606 (6.2)
(Missing)	674 (0.3)
**Abuse**^**a**^	
Yes	29,139 (14)
No	167,279 (82)
(Missing)	6,479 (3.2)
**Outsider growing up**	
Yes	28,732 (14)
No	170,577 (84)
(Missing)	3,589 (1.8)
**Self-rated health growing up**	
Excellent	67,121 (33)
Very good	63,086 (31)
Good	47,378 (23)
Fair	19,877 (9.8)
Poor	4,906 (2.4)
(Missing)	530 (0.3)
**Immigration status**	
Born in this country	190,998 (94)
Born in another country	9,791 (4.8)
(Missing)	2,110 (1.0)
**Age 12 religious service attendance**	
At least 1 per week	83,237 (41)
1–3 per month	33,308 (16)
<1 per month	36,928 (18)
Never	47,445 (23)
(Missing)	1,980 (1.0)

^a^History of abuse was not collected in Israel.

With regard to demographic relations and associations with childhood experiences, we will first comment on the results of random effects meta-analysis across the 22 countries. We then turn to variations across countries. Table 4 presents ordered means of a composite flourishing index. The index includes two indicators each across six domains: happiness, health, meaning, character, relationships and financial security (see Table 5 below and ref. ^5^). Table 4 presents weighted means so as to be nationally representative within country, and alternately includes means both without and with financial security (10 versus 12 indicators, respectively), with standard deviations, Gini coefficients and alpha reliability coefficients. Without financial security, the highest means were reported in Indonesia (8.47), Mexico (8.19) and the Philippines (8.11); the lowest were in Japan (5.93), Turkey (6.59) and the United Kingdom (6.88). When including financial security, Israel instead of Mexico has the second highest mean. Standard deviations for composite flourishing across countries range from 1.30 to 2.03. Gini coefficients for inequality tend to be highest in those countries reporting the lowest means. Cronbach’s alpha for the index is relatively high in all countries. All subsequent tables report results with financial security. Analogous analyses without financial security are given in Supplementary Tables S26–S28.

**Table 4:** Ordered means of composite flourishing index (without and with financial security), with standard deviations, Gini coefficients and alpha reliability coefficients (*N* = 202,898)

Ordering	Flourishing without financial indicators						Flourishing with financial indicators					
	Country	Mean	95% CI	s.d.	Gini	alpha	Country	Mean	95% CI	s.d.	Gini	alpha
1.	Indonesia	8.47	(8.42, 8.52)	1.35	0.09	0.88	Indonesia	8.10	(8.05, 8.15)	1.35	0.09	0.84
2.	Mexico	8.19	(8.15, 8.24)	1.36	0.09	0.88	Israel	7.87	(7.74, 7.99)	1.36	0.10	0.89
3.	Philippines	8.11	(8.06, 8.16)	1.44	0.10	0.87	Philippines	7.71	(7.66, 7.76)	1.42	0.10	0.84
4.	Israel	8.00	(7.88, 8.12)	1.34	0.09	0.88	Mexico	7.64	(7.59, 7.68)	1.38	0.10	0.83
5.	Nigeria	7.82	(7.76, 7.89)	1.46	0.10	0.82	Poland	7.55	(7.47, 7.64)	1.31	0.09	0.89
6.	Argentina	7.79	(7.74, 7.84)	1.49	0.11	0.87	Nigeria	7.37	(7.31, 7.43)	1.40	0.11	0.80
7.	Kenya	7.77	(7.71, 7.83)	1.62	0.12	0.77	Egypt	7.32	(7.25, 7.38)	1.50	0.11	0.78
8.	Poland	7.63	(7.55, 7.72)	1.30	0.09	0.88	Kenya	7.28	(7.23, 7.34)	1.61	0.12	0.77
9.	Brazil	7.63	(7.59, 7.67)	1.72	0.12	0.89	Tanzania	7.19	(7.10, 7.28)	1.83	0.14	0.81
10.	Egypt	7.63	(7.57, 7.69)	1.46	0.11	0.76	Argentina	7.14	(7.09, 7.19)	1.47	0.11	0.82
11.	Tanzania	7.48	(7.39, 7.57)	1.82	0.14	0.79	Hong Kong	7.12	(7.04, 7.20)	1.75	0.14	0.96
12.	India	7.43	(7.38, 7.48)	2.03	0.15	0.82	United States	7.11	(7.07, 7.16)	1.66	0.13	0.91
13.	South Africa	7.41	(7.32, 7.50)	1.59	0.12	0.82	Sweden	7.10	(7.07, 7.13)	1.54	0.12	0.90
14.	Spain	7.31	(7.26, 7.35)	1.42	0.11	0.87	South Africa	7.07	(6.98, 7.16)	1.55	0.12	0.81
15.	United States	7.18	(7.14, 7.23)	1.65	0.13	0.92	Brazil	7.02	(6.98, 7.06)	1.67	0.13	0.85
16.	Hong Kong	7.17	(7.09, 7.25)	1.75	0.14	0.96	Australia	7.01	(6.95, 7.08)	1.61	0.13	0.91
17.	Germany	7.10	(7.07, 7.14)	1.37	0.11	0.84	Germany	7.01	(6.97, 7.04)	1.38	0.11	0.84
18.	Sweden	7.04	(7.01, 7.07)	1.57	0.12	0.90	Spain	6.90	(6.85, 6.94)	1.40	0.11	0.84
19.	Australia	7.02	(6.95, 7.09)	1.59	0.13	0.91	India	6.87	(6.82, 6.91)	1.90	0.16	0.80
20.	United Kingdom	6.88	(6.81, 6.94)	1.70	0.14	0.91	United Kingdom	6.79	(6.72, 6.85)	1.68	0.14	0.90
21.	Turkey	6.59	(6.46, 6.71)	1.93	0.17	0.86	Turkey	6.32	(6.19, 6.44)	1.96	0.18	0.88
22.	Japan	5.93	(5.91, 5.96)	1.79	0.17	0.94	Japan	5.89	(5.87, 5.92)	1.79	0.17	0.94

CI, confidence interval; Gini, index of inequality; alpha, reliability coefficient alpha.

**Table 5:** Flourishing measure and questions

Domain	Question/statement^a^
D1. Happiness	Q1. Overall, how satisfied are you with life as a whole these days?
D1. Happiness	Q2. In general, how happy or unhappy do you usually feel?
D2. Health	Q3. In general, how would you rate your physical health?
D2. Health	Q4. How would you rate your overall mental health?
D3. Meaning	Q5. Overall, to what extent do you feel the things you do in your life are worthwhile?
D3. Meaning	Q6. I understand my purpose in life.
D4. Character	Q7. I always act to promote good in all circumstances, even in difficult and challenging situations.
D4. Character	Q8. I am always able to give up some happiness now for greater happiness later.
D5. Relationships	Q9. I am content with my friendships and relationships.
D5. Relationships	Q10. My relationships are as satisfying as I would want them to be.
D6. Financial stability	Q11. How often do you worry about being able to meet normal monthly living expenses?
D6. Financial stability	Q12. How often do you worry about safety, food or housing?

^a^Each question or statement is evaluated 0–10 (ref. ^5^). Anchors are Q1 (0, not satisfied at all,; 10, completely satisfied); Q2 (0, extremely unhappy; 10, extremely happy); Q3 and Q4 (0, poor; 10, excellent); Q5 (0, not at all worthwhile; 10, completely worthwhile); Q6, Q9 and Q10 (0, strongly disagree; 10, strongly agree); Q7 and Q8 (0, not true of me; 10, completely true of me); Q11 and Q12 (0, worry all of the time; 10, do not ever worry).

Table 6 presents random effects meta-analysis of composite flourishing means by demographic category. Aggregated across all countries, flourishing increases with age from 7.03 for those aged 18–49 years to 7.36 for those aged 80 years or older; it is relatively similar for men and women, but lower for those identifying as ‘other’ genders. Flourishing is notably higher for those married (7.34) than for those separated (6.77) or divorced (6.85) and higher for those employed (7.20) and self-employed (7.28) than for those unemployed (6.51). Flourishing tends to increase somewhat with education, but more dramatically with religious service attendance, ranging from a mean of 6.86 for those never attending to 7.67 for those attending more than weekly. It is slightly higher for those born in the country (7.16) than for those not (7.02). Results are generally similar when excluding financial security (Supplementary Table 26), but the relations with age are slightly more muted in these analyses.

**Table 6:** Random effects meta-analysis of flourishing means by demographic category (*N* = 202,898)

Variable					Prediction Interval				Global *P* value
	Category	Estimate	95% CI	S.E.	LL	UL	Heterogeneity (τ)	*I*^2^	
Age group (years)									6.05 × 10^–16^**
18–24		7.05	(6.78, 7.33)	0.14	5.68	8.07	0.65	99.2	
25–29		7.04	(6.78, 7.30)	0.13	5.58	8.14	0.60	98.8	
30–39		7.03	(6.79, 7.28)	0.13	5.51	8.18	0.59	99.3	
40–49		7.05	(6.81, 7.29)	0.12	5.46	8.15	0.57	99.1	
50–59		7.14	(6.93, 7.36)	0.11	5.61	8.03	0.51	98.7	
60–69		7.25	(7.04, 7.45)	0.11	6.14	8.02	0.48	98.5	
70–79		7.33	(7.11, 7.56)	0.12	6.38	8.19	0.51	98.1	
80 or older^a^		7.36	(7.11, 7.62)	0.13	6.16	8.10	0.52	92.8	
Gender									1.14 × 10^–15^**
Male		7.19	(6.99, 7.40)	0.11	5.76	8.08	0.49	99.5	
Female		7.12	(6.92, 7.32)	0.10	6.02	8.12	0.47	99.5	
Other^a^		6.09	(5.69, 6.50)	0.21	5.38	7.33	0.58	78.4	
Marital status									4.87 × 10^–16^**
Married		7.34	(7.15, 7.54)	0.10	6.19	8.16	0.46	99.5	
Separated		6.77	(6.54, 6.99)	0.11	5.83	7.76	0.50	93.1	
Divorced		6.85	(6.64, 7.05)	0.10	5.60	7.81	0.46	95.3	
Widowed		7.17	(6.97, 7.38)	0.10	6.27	7.98	0.45	94.4	
Domestic partner		7.01	(6.75, 7.26)	0.13	5.38	7.96	0.56	98.3	
Single, never married		6.92	(6.63, 7.21)	0.15	5.17	7.95	0.70	99.6	
Employment status									6.04 × 10^–16^**
Employed for an employer		7.20	(6.99, 7.42)	0.11	5.75	8.03	0.51	99.5	
Self-employed		7.28	(7.08, 7.48)	0.10	6.00	8.22	0.46	98.5	
Retired		7.32	(7.12, 7.52)	0.10	6.39	8.16	0.46	98.3	
Student		7.10	(6.83, 7.38)	0.14	6.09	8.23	0.65	98.4	
Homemaker		7.05	(6.85, 7.25)	0.10	6.20	8.14	0.46	97.6	
Unemployed and looking for a job		6.51	(6.15, 6.86)	0.18	4.40	7.74	0.83	98.9	
None of these/other		6.69	(6.33, 7.04)	0.18	5.12	8.00	0.82	97.6	
Education (years)									9.66 × 10^–16^**
Up to 8		7.05	(6.81, 7.30)	0.12	5.20	8.12	0.57	98.8	
9–15		7.16	(6.94, 7.38)	0.11	5.78	8.07	0.52	99.7	
16+		7.35	(7.15, 7.54)	0.10	6.31	8.11	0.45	99.1	
Religious service attendance									4.04 × 10^–16^**
>1 per week		7.67	(7.44, 7.89)	0.12	6.76	8.98	0.53	98.4	
1 per week		7.42	(7.27, 7.57)	0.08	6.58	8.08	0.35	97.6	
1–3 per month		7.21	(7.03, 7.39)	0.09	6.13	7.97	0.41	96.9	
A few times a year		7.08	(6.89, 7.28)	0.10	5.93	7.83	0.45	98.8	
Never		6.86	(6.65, 7.07)	0.11	5.72	8.00	0.50	99.3	
Immigration status									1.95 × 10^–8^**
Born in this country		7.16	(6.95, 7.36)	0.11	5.89	8.10	0.49	99.7	
Born in another country		7.02	(6.86, 7.18)	0.08	6.08	7.56	0.34	89.6	

S.E., standard error; LL, lower limit of prediction interval; UL, upper limit of prediction interval; prediction interval is the range of likely values of the estimate for a randomly selected country; τ, standard deviation of the distribution of means across countries, which is an indicator of cross-national heterogeneity; *I*^2^, an estimate of the variability in means due to heterogeneity across countries versus sampling variability, which is not uncommonly nearly 100% when there is substantial precision in estimated mean within country; Global *P* value corresponds to a test of the null hypothesis that there are no differences between the groups for that sociodemographic characteristic in all of the 22 countries. Composite flourishing outcome is the mean of all 12 individual item responses (Table 5). *P < 0.05; **P < 0.007 (Bonferroni corrected threshold).

^a^This group is very small (<0.1% of the observed sample) within several countries, leading to large uncertainty in this estimate—caution is needed in interpreting this estimate.

Table 7 presents results from random effects meta-analysis of multivariate regression of composite flourishing on all childhood predictors (Table 7) simultaneously. Good childhood relationships with mother (0.18; 95% CI: 0.11, 0.24) and father (0.11; 95% CI :0.07, 0.18) were positively associated with adult flourishing. There is some evidence that having parents who were never married (−0.11; 95% CI: −0.21, −0.01), or when one had died (−0.07; 95% CI: −0.16, 0.02), or possibly divorced (−0.07; 95% CI: −0.17, 0.02), compared with having parents who were married, was associated with lower adult flourishing. There is a notable gradient in flourishing with childhood subjective financial status, with finding it very difficult to get by associated with lower flourishing (−0.27; 95% CI: −0.35, −0.19) and living comfortably associated with higher adult flourishing (0.20; 95% CI: 0.14, 0.26) compared with those who got by. A similar gradient can be seen regarding self-rated health growing up: poor health is associated with lower (−0.34; 95% CI :−0.54, −0.14) adult flourishing, and excellent health with higher (0.46; 95% CI: 0.31, 0.62) flourishing compared with those in good health. Abuse (−0.34; 95% CI: −0.40, −0.29) and feeling like an outsider growing up (−0.26; 95% CI: −0.33, −0.18) were associated with lower adult flourishing. Attending religious services weekly was associated with higher adult flourishing (0.27; 95% CI :0.17, 0.36) compared with never attending. Flourishing tended to increase with age, but there was relatively little difference with regard to immigration status, or men versus women.

**Table 7:** Random effects meta-analysis of regression of flourishing on childhood predictors (*N* = 202,898)

					Estimated proportion of effects by threshold				
Variable	Category	Estimate	95% CI	S.E.	<−0.10	>0.10	Heterogeneity (τ)	${\boldsymbol{I}}^{\bf{2}}$	Global *P* value
Relationship with mother	(Ref: very bad/somewhat bad)								1.99 × 10^–5^**
	Very good/somewhat good	0.18	(0.11, 0.24)	0.03	0.00	0.77	0.11	53.8	
Relationship with father	(Ref: very bad/somewhat bad)								4.83 × 10^–15^**
	Very good/somewhat good	0.12	(0.07, 0.18)	0.03	0.00	0.59	0.09	55.6	
Parent marital status	(Ref: parents married)								5.44 × 10^–8^**
	Divorced	−0.07	(−0.17, 0.03)	0.05	0.45	0.14	0.21	85.1	
	Single, never married	-0.11	(−0.21, −0.01)	0.05	0.36	0.09	0.19	81.5	
	One or both parents had died	−0.07	(−0.16, 0.02)	0.05	0.36	0.09	0.17	66.3	
Subjective financial status of family growing up	(Ref: got by)								1.38 × 10^–15^**
	Lived comfortably	0.20	(0.14, 0.26)	0.03	0.00	0.82	0.14	88.8	
	Found it difficult	−0.11	(−0.16, −0.07)	0.02	0.59	0.00	0.07	56.1	
	Found it very difficult	−0.27	(−0.35, −0.19)	0.04	0.91	0.00	0.14	59.5	
Abuse	(Ref: no)								1.77 × 10^–13^**
	Yes	−0.34	(−0.40, −0.29)	0.03	1.00	0.00	0.10	66.7	
Outsider growing up	(Ref: no)								3.21 × 10^–15^**
	Yes	−0.26	(−0.33, −0.18)	0.04	0.91	0.00	0.14	77.2	
Self-rated health growing up	(Ref: good)								6.05 × 10^–16^**
	Excellent	0.46	(0.31, 0.62)	0.08	0.00	0.91	0.36	96.7	
	Very good	0.24	(0.16, 0.32)	0.04	0.00	0.86	0.18	89.9	
	Fair	−0.23	(−0.31, −0.14)	0.04	0.86	0.00	0.17	80.1	
	Poor	−0.34	(−0.54, −0.14)	0.10	0.73	0.09	0.41	85.1	
Immigration status	(Ref: born in this country)								4.84 × 10^–15^**
	Born in another country	0.03	(−0.10, 0.15)	0.06	0.32	0.45	0.25	82.0	
Age 12 religious service attendance	(Ref: never)								2.42 × 10^–15^**
	At least 1 per week	0.27	(0.17, 0.36)	0.05	0.00	0.86	0.20	84.3	
	1–3 per month	0.21	(0.11, 0.31)	0.05	0.05	0.68	0.20	85.0	
	< 1 per month	0.10	(0.05, 0.15)	0.03	0.00	0.45	0.09	59.8	
Year of birth	(Ref: 1998–2005; age 18–24)								5.38 × 10^–16^**
	1993–1998; age 25–29	−0.01	(−0.08, 0.06)	0.04	0.23	0.23	0.14	74.3	
	1983–1993; age 30–39	0.02	(−0.08, 0.12)	0.05	0.27	0.45	0.23	91.3	
	1973–1983; age 40–49	0.03	(−0.11, 0.16)	0.07	0.32	0.45	0.30	93.6	
	1963–1973; age 50–59	0.11	(−0.06, 0.29)	0.09	0.27	0.55	0.40	95.7	
	1953–1963; age 60–69	0.21	(−0.01, 0.42)	0.11	0.23	0.64	0.49	96.1	
	1943–1953; age 70–79	0.28	(−0.02, 0.58)	0.15	0.32	0.55	0.70	97.0	
	1943 or earlier; age 80+^a^	0.34	(−0.01, 0.69)	0.18	0.36	0.59	0.77	94.0	
Gender	(Ref: male)								2.94 × 10^–15^**
	Female	−0.02	(−0.07, 0.03)	0.03	0.23	0.14	0.11	86.8	
	Other^a^	−0.23	(−0.56, 0.09)	0.16	0.67	0.28	0.60	85.6	

Ref., reference; CI, confidence interval; S.E., standard error; the estimated proportion of effects is the estimated proportion of effects above (or below) a threshold based on the calibrated effect sizes^108^; *I*^2^ is an estimate of the variability in means due to heterogeneity across countries versus sampling variability; the global *P* value corresponds to the joint test of the null hypothesis that the country-specific joint parameter Wald tests (all parameters within variable groups are zero) are null for all 22 countries; additional details of heterogeneity of effects are available in the forest plots of the Supplementary Information. Composite flourishing outcome is the mean of all 12 individual item responses (Table 5). *P < 0.05; **P < 0.004 (Bonferroni corrected threshold).

^a^This group is very small (<0.1% of the observed sample) within several countries, leading to high uncertainty in this estimate—caution is needed in interpreting this estimate.

Table 8 presents E-value (the minimum strength of the association an unmeasured confounder must have with both the outcome and the predictor, above and beyond all measured covariates, for an unmeasured confounder to explain away an association) sensitivity analyses^35^ of meta-analyzed childhood predictor associations to potential unmeasured confounding. Some of these associations between the childhood predictors and adult flourishing were moderately robust to potential unmeasured confounding. For example, to explain away the association between abuse in childhood with higher adult flourishing, an unmeasured confounder that was associated with both absence of abuse and higher flourishing by risk ratios of 1.74-fold each, above and beyond the measured covariates, could suffice, but weaker joint confounder associations could not; to shift the 95% confidence interval to include the null, an unmeasured confounder that was associated with both absence of abuse and higher adult flourishing by risk ratios of 1.64-fold each, above and beyond the measured covariates, could suffice, but weaker joint confounder associations could not.

**Table 8:** Sensitivity of meta-analyzed childhood predictors to unmeasured confounding (*N* = 202,898)

Variable	Category	E-value for estimate	E-value for 95% CI
Relationship with mother	(Ref: very bad/somewhat bad)		
	Very good/somewhat good	1.46	1.33
Relationship with father	(Ref: very bad/somewhat bad)		
	Very good/somewhat good	1.36	1.25
Parent marital status	(Ref: parents married)		
	Divorced	1.25	1.00
	Single, never married	1.33	1.09
	One or both parents had died	1.25	1.00
Subjective financial status of family growing up	(Ref: got by)		
	Lived comfortably	1.49	1.38
	Found it difficult	1.34	1.25
	Found it very difficult	1.62	1.48
Abuse	(Ref: no)		
	Yes	1.74	1.64
Outsider growing up	(Ref: no)		
	Yes	1.59	1.47
Self-rated health growing up	(Ref: good)		
	Excellent	1.94	1.68
	Very good	1.57	1.43
	Fair	1.54	1.39
	Poor	1.73	1.39
Immigration status	(Ref: born in this country)		
	Born in another country	1.15	1.00
Age 12 religious service attendance	(Ref: never)		
	At least 1 per week	1.61	1.45
	1–3 per month	1.51	1.34
	<1 per month	1.31	1.19
Year of birth	(Ref: 1998–2005; age 18–24)		
	1993–1998; age 25–29	1.08	1.00
	1983–1993; age 30–39	1.11	1.00
	1973–1983; age 40–49	1.14	1.00
	1963–1973; age 50–59	1.33	1.00
	1953–1963; age 60–69	1.50	1.00
	1943–1953; age 70–79	1.63	1.00
	1943 or earlier; age 80+^a^	1.73	1.00
Gender	(Ref: male)		
	Female	1.11	1.00
	Other^a^	1.55	1.00

^a^This group is very small (<0.1% of the observed sample) within several countries leading to high uncertainty in this estimate—caution is needed in interpreting this estimate.

### Variation across countries

The overall patterns in the preceding, pooled over all countries, are illuminating but disguise important country-specific variation (Supplementary Information). We comment on this variation in the following, but caution is needed with regard to over-interpretation, especially in cases when magnitudes are not strong. Flourishing tends to increase with age in many countries, including Argentina, Australia, Brazil, Sweden and the United States, but not in all. In India, Egypt, Kenya and Japan, patterns are somewhat more U-shaped. In Spain, the pattern is somewhat U-shaped except for the youngest age group (18–24-year-olds), which reports the lowest flourishing. In Poland and Tanzania, it is mostly decreasing with age. Other countries show more complex patterns with age. Men and women are fairly similar globally, but there are greater differences in certain countries. In Brazil, men report higher flourishing than women (0.39; 95% CI: 0.32, 0.47); in Japan, women report higher flourishing than men (−0.26; 95% CI: −0.32, −0.21). The patterns of married individuals reporting higher flourishing than those divorced (or separated) are nearly universal, but differences vary from 0.92 (0.63, 1.22) in Israel to 0.10 (−0.10, 0.30) in Argentina. In most countries, those married report notably higher flourishing than those who are single, but not in all: married report lower flourishing than single in India (−0.39; 95% CI: −0.50, −0.28) and Tanzania (−0.35; 95% CI: −0.51, −0.20). Those employed effectively universally report higher flourishing than unemployed, but again differences vary from 1.50 (95% CI: 1.18, 1.83) in the United States to just 0.09 (95% CI: −0.03, 0.22) in Kenya. There is, however, considerable variation across countries in comparisons of employed versus self-employed, retired or student, with those self-employed and retired reporting higher flourishing than those employed in many developing countries and students reporting notably higher flourishing than those employed in Poland, India, Japan, Tanzania, Israel, Egypt and Kenya. Those attending religious services more than once a week (or weekly) universally report higher flourishing than those never attending, but differences vary from 2.33 (2.12, 2.54) in Hong Kong to 0.15 (−0.01, 0.30) in India. In most countries, those with more education report higher flourishing, but the reverse is the case in Hong Kong and Australia. Associations with immigration status also vary by country, with those born in the country reporting notably higher flourishing in the Philippines, Poland, India, Israel, Mexico and Hong Kong, whereas the reverse is the case in Spain and Australia.

Likewise, with the analyses concerning the childhood predictors, there are some patterns that are nearly universal and others that seem to vary by culture and context. Associations between good maternal relationship and adult flourishing were nearly universal but varied in magnitude from 0.63 (95% CI: 0.27, 0.99) in Indonesia to effectively null (−0.14; −0.43, 0.14) in Israel, and likewise for good paternal relationship. Compared with those with married parents growing up, those with divorced, separated, single or one or more deceased parents tended to have lower flourishing in adulthood, although Turkey was an anomaly with respect to divorce. Compared with a subjective financial status of getting by in childhood, living comfortably was essentially universally associated with higher adult flourishing with magnitudes ranging from 0.70 (0.53, 0.87) in Hong Kong to effectively null (−0.05; −0.17, 0.08) in Nigeria; and finding it very difficult was universally associated with lower adult flourishing with magnitudes ranging from −0.92 (−1.45, −0.40) in Turkey to null (0.00; −0.36, 0.35) in Sweden. Experiencing abuse and feeling like an outsider were likewise universally associated with lower adult flourishing. Excellent, as compared with good, childhood health was universally associated with higher adult flourishing, and while poor childhood health was nearly universally associated with lower adult flourishing, there were possibly a few exceptions: in Germany there was evidence of a positive association (0.40; 0.07, 0.74). Weekly religious service attendance growing up was associated with higher adult flourishing in almost all countries, with the largest effect sizes in Poland (1.02; 0.77,1.28) followed by Hong Kong and Turkey; however, Kenya, South Africa and Tanzania manifested slightly negative estimates (−0.22, −0.15, −0.05, respectively), although with confidence intervals including 0. Patterns across countries for immigration status, age/birth cohort and gender roughly followed that reported in the preceding for demographic statistics, although after multivariate adjustment, further gender differences emerged, with notable evidence for women having higher flourishing than men in Egypt, Japan and Hong Kong, and men having higher flourishing than women in Spain, United Kingdom, Brazil, Kenya and Argentina.

### Results on specific aspects of flourishing

The preceding analyses concerned composite flourishing. However, just as pooling across countries is of interest, but obscures country-specific relations, so also the use of a composite flourishing measure can obscure more nuanced relations with more-specific aspects of well-being. A series of papers, following the same methodology described in the preceding, has examined these various more-specific well-being domains, and many of these papers are part of the present GFS Special Collection. While these papers contain far more detailed analysis than can possibly be summarized here, some higher-level remarks may be of interest in trying to understand similarities and differences in relationships with more-specific aspects of well-being. We will provide a cursory overview of some of the results in each of the well-being domains in turn, focusing principally on any patterns that notably diverge from those reported concerning composite flourishing. Many, albeit not all, of the differences concern relations with age, gender and childhood adversity.

Patterns for self-rated physical health^36^ followed that of composite flourishing in many respects but differed in that scores decreased with age, although with some variation (for example, with U-shaped patterns in Australia, Japan and Sweden), and men report better health than women overall, with the reverse notable only in Japan. Patterns with health limitations^37^ and physical pain^38^ were conversely similar, that is, increasing in age. Physical pain was one of the very few outcomes that was adversely associated with religious service attendance^38,39^. For pain, there was evidence in some countries (for example, South Africa and Israel) that those who financially just got by reported lower levels of adult pain than those who lived comfortably in childhood^39^. Patterns for self-rated mental health tended to follow those of flourishing, but were U-shaped with age overall, although increasing in Australia, United States and Sweden and decreasing with age in Israel and Tanzania^40^. Depression and anxiety, assessed with the Patient Health Questionnaire-4 (PHQ-4)^41^, both decreased with age, were less related to religious service attendance and were somewhat higher for women than men.

With respect to emotional well-being, patterns for happiness and life satisfaction mostly follow those of flourishing, but are slightly more U-shaped (or ‘J-shaped’) with age^42^, somewhat comparable to much previous research^43^. The increasing-with-age pattern for composite flourishing is thus shaped by other aspects of well-being documented in the following. There are also notable differences in the country-specific reporting and in some of the demographic relationships between life satisfaction, life evaluation and affective happiness, and further discussion of these matters and of differences between the GFS and the GWP is given elsewhere^42^. In contrast to life satisfaction/evaluation, the outcomes of balance, inner peace and optimism are all increasing with age^44,45^. Patterns with mastery are similar but somewhat smaller in magnitude^46^. In contrast to many outcomes, suffering is not strongly patterned with age overall, or with religious service attendance, but there is notable variation in this across countries; suffering varies more with marital status, employment, education and gender, with women reporting higher suffering than men in most countries^47^ even after multivariate adjustment^48^.

Meaning and purpose manifest similar demographic relationships as with composite flourishing and are likewise increasing with age^49^. For meaning and purpose, the relationships with childhood adversity seem more complex. Death of a parent seems to matter more for meaning and purpose in Spain, Israel and Nigeria than in Australia, Philippines, Argentina or Japan (wherein estimates were positive, albeit with wide confidence intervals). In some countries (United States), difficult, or even very difficult (Argentina), childhood financial circumstances were associated with higher adult meaning, and in others (Poland), living comfortably was associated with lower adult meaning. By contrast, childhood abuse and feeling like an outsider were essentially universally associated with lower adult meaning^50^.

Concerning character (‘volitional well-being’), assessments of promoting the good are mostly increasing with age (although not in all countries; in India and Tanzania this was decreasing with age); were slightly higher with women and notably higher with religious service attendance^51,52^. Similar patterns were manifest with more objective behaviors such as, within the last month, volunteering, charitable giving and helping a stranger, although charitable giving and helping were slightly higher among men, volunteering was more uniform in age (until decreasing at 80+), and helping a stranger decreased with increasing age^53,54^. The relationships of these behaviors with childhood adversity were also more complex. Childhood abuse and feeling like an outsider predicted higher adult volunteering, giving and helping; the relationship with childhood health with both volunteering and charitable giving was U-shaped with both excellent childhood health and poor childhood health predicting higher adult volunteering than good health^55,56^. By contrast, abuse, feeling like an outsider, poor childhood health and difficult childhood financial circumstances all tended to decrease capacity for delayed gratification, showing love/care for others, gratitude and hope^57–60^, although none of these variables mattered for forgiveness^61^. Forgiveness, gratitude and showing love/care were all increasing with age; hope was relatively flat with age; and adult delayed gratification was decreasing with age. Women reported higher gratitude and love/care than men and reported similar forgiveness and hope overall, but higher after multivariate control^58,60–64^.

With regard to social well-being, patterns for close social relationships mostly followed those of composite flourishing^65,66^ except women reported very slightly higher than men, and there was little relationship with education overall. Those with lower education reported higher relationship quality in Indonesia, Kenya, Sweden and the United States, but this pattern was reversed in Brazil, Israel, Japan and Poland. In general, those who were married reported the highest relationship quality, but widowed individuals did so in 6 of the 22 countries. Having an intimate friend was roughly constant with age (but highest among 80+); by contrast, having social support was U-shaped with age, higher for women and higher for students and those retired than for those employed; other patterns were relatively similar to that of composite flourishing^67^.

Patterns showed somewhat more variation with communal dimensions of social well-being. For non-religious community engagement, weekly participation decreased with age, was slightly higher for men, higher for those single than married, higher with students than the employed, but itself increasing with religious service attendance^68^. Having a sense of belonging within the country followed many of the same patterns as composite flourishing but was slightly higher for women, slightly decreasing with education and notably higher for those born within the country^69,70^. Similar results pertain to the demographic relations with satisfaction with the city or area in which one lives (‘place satisfaction’), except for essentially no male–female difference and a weaker relation with being born within the country. Trust within the country and feeling one had a political voice were both slightly U-shaped with age and slightly higher for men; with political voice, but not trust, which was increasing with education^71,72^. Other patterns were relatively similar to that of composite flourishing.

Concerning financial well-being, many of the patterns follow those of composite flourishing, especially with respect to the childhood predictors^73^. However, across the subjective and objective financial well-being indicators, there were additional important differences, especially with respect to age, gender and religious service attendance. See ref. ^73^ for further details.

With respect to spiritual well-being, as noted previously, how this is understood will vary by tradition and culture. Moreover, many of the religious or spiritual GFS assessments concern behaviors or beliefs that may be determinants of well-being, rather than constituting spiritual well-being per se. We consequently restrict comment to a single item involving feeling loved or cared for by God or a spiritual force^74^. Even this, of course, may not be applicable in non-theistic religious traditions. We thus focus on the sub-analysis that excludes respondents who indicate that the question is not relevant. The patterns for this indicator follow fairly closely those for composite flourishing except proportions are higher for women, slightly U-shaped with education and higher for those who have immigrated.

The patterns across countries are complex, and there is no straightforward way to summarize results across all of the indicators and papers. There is considerable variability in the ordered means of countries across indicators (Supplementary Table 29). However, as will be discussed, such ordering of means needs to be interpreted cautiously since interpretation of items and response scales may vary by language and culture^75,76^. The interpretation of these thus often makes sense only on a relative basis within countries. In Table 9, we report, for each country, the four well-being indicators (out of 47 total) for which the country, relative to the other indicators, ranked highest, and the four indicators for which the country, relative to the other indicators, ranked lowest, to give some indication of the relative strengths and weaknesses of each country.

**Table 9:** Relative highest and lowest well-being indicators by country

Country	Relative strengths	Relative areas for growth
**Argentina**	Promoting good, mastery, social support, hope	Financial worry, material worry, anxiety/controlling worry, subjective financial well-being
**Australia**	Subjective financial well-being, material worry, education (social support, charitable giving, volunteering, financial worry, trust)	Freedom, relational contentment, satisfying relationships, pain
**Brazil**	Life evaluation five years from now, optimism, helping (promoting good, gratitude)	Feeling anxious (anxiety/controlling worry, pain, financial worry, material worry, city satisfaction, trust)
**Egypt**	Belonging, balance in life (mastery, forgiveness, helping)	Traumatic distress, discrimination, volunteering, pain
**Germany**	Depressed/little interest, feeling depressed (feeling anxious, loneliness, discrimination, education)	Housing, health limitations, belonging (delayed gratification, love)
**Hong Kong**	Peace, employment (balance in life, subjective financial well-being, trust)	Relational contentment, promoting good, gratitude, love
**India**	Housing, government approval, political voice, city satisfaction	Education, depressed/little interest, financial worry, material worry
**Indonesia**	Numerous^a^	Intimate friend, education, helping, employment
**Israel**	(Life evaluation today, balance in life, social support, loneliness, pain, education)	Forgiveness, volunteering, political voice, belonging
**Japan**	Anxiety/controlling worry, pain, (traumatic distress, discrimination, employment)	Numerous^b^
**Kenya**	Purpose, self-rated mental health, volunteering (optimism, relational contentment, forgiveness, self-rated physical health)	Subjective financial well-being, employment, life satisfaction, housing
**Mexico**	Mastery (happiness, life satisfaction, meaning, social support, hope, gratitude)	Material worry, subjective financial well-being, intimate friend, charitable giving
**Nigeria**	Intimate friend, forgiveness, volunteering, helping	Subjective financial well-being, housing, discrimination, education
**Philippines**	Delayed gratification, love, city satisfaction (freedom, relational contentment, satisfying relationships, intimate friend, political voice, trust)	Depressed/little interest, anxiety/controlling worry, health limitations (peace, mastery, feeling depressed, suffering, charitable giving)
**Poland**	(Traumatic distress, feeling depressed, feeling anxious, anxiety/controlling worry, suffering, health limitations)	Delayed gratification, helping, volunteering, love
**South Africa**	Pain, suffering (peace, forgiveness, housing)	Employment, city satisfaction, discrimination (meaning, charitable giving, trust)
**Spain**	Intimate friend, balance in life, mastery, discrimination	Political voice, peace, financial worry, material worry
**Sweden**	Discrimination, financial worry, material worry, subjective financial well-being	Hope (purpose, promoting good, self-rated physical health, political voice)
**Tanzania**	Self-rated mental health, government approval, discrimination, love	Life evaluation today, balance in life, feeling depressed (life satisfaction, intimate friend, education)
**Turkey**	Health limitations, government approval, education, helping	(Happiness, life satisfaction, peace, suffering, loneliness, forgiveness)
**United Kingdom**	Charitable giving, education, volunteering, employment	(Optimism, freedom, purpose, self-rated physical health, health limitations, political voice)
**United States**	Traumatic distress, subjective financial well-being (mastery, depressed/little interest, material worry, education)	Trust (freedom, satisfying relationships, government approval, belonging)

The table reports the four relative strengths and areas for growth in each country as determined by the highest and lowest ordering of the means for that country across the self-report assessments; when there was a tie such that including the tied well-being areas required listing more than four, these tied aspects are put in parentheses. For the purposes of relative strengths and weaknesses, three pairs of items were kept separate rather than combined: the PHQ-4 depression and anxiety items; the financial and material worry items; and the subjective social connectedness items.

^a^Indonesia has many relative strengths for which its self-report assessment was the highest of the 22 countries, including happiness, life satisfaction, freedom, meaning, purpose, relational contentment, satisfying relationships, promoting good, hope, gratitude, charitable giving, self-rated physical health, political voice, trust.

^b^Japan has a number of relative areas for growth for which its self-report assessment was the lowest of the 22 countries, including life evaluation five years from now, optimism, freedom, mastery, meaning, purpose, relational contentment, satisfying relationships, social support, intimate friend, self-rated mental health, promoting good, delayed gratification, hope, gratitude, love, charitable giving, helping, self-rated physical health, belonging.

## Discussion

With regard to the primary results from the flourishing index, several comments merit attention. One of the more concerning results is the relation with age. On average, when pooled across the 22 countries, flourishing is essentially flat with age through ages 18–49 and then increases with age thereafter. This is in striking contrast to earlier work—focused mostly on life satisfaction/evaluation—which had suggested a more dramatically U-shaped pattern with age^43^. Even with life satisfaction, pooled over the 22 GFS countries, this is now more J-shaped than U-shaped. As noted in the preceding, the increasing-with-age pattern in 2023, when pooled across GFS countries, now also holds on average with many specific aspects of well-being, including balance, inner peace, mastery, optimism, meaning and purpose, promoting good, charitable giving, forgiveness, gratitude, showing love/care, relationship quality, feeling belonging, place satisfaction and feeling loved by God or a spiritual force. Earlier nationally representative 2022 data in the United States identified these patterns with all of the various flourishing domains, including life satisfaction^77^. There is also other corroborating evidence concerning changes in patterns between age and life evaluation and mental health^78–80^. While there are distinctions across measures, covariate controls and countries, the cumulative evidence here and elsewhere likely reflects some real changes in well-being’s relation with age that have been taking place over time. Young people are not doing as well as they used to be. While causes are likely diverse, mental health concerns with young adults are clearly on the rise. These patterns are not universal. As noted, in some countries the patterns concerning flourishing and age are still somewhat more U-shaped (India, Egypt, Kenya, Japan) and in others (Poland, Tanzania) decreasing with age. Nevertheless, the overall global pattern is troubling. An open question concerns whether these relations constitute a newly patterned ‘age effect’ or whether this is in fact a new ‘cohort effect.’ This will be resolved only with more data over time. It is possible that because of social and economic conditions, it simply is more difficult to be young today, but that well-being for these young people will increase with age. It is, however, also possible, for example, that, within-person, over time, well-being will continue to follow a U-shaped pattern (but with the mean of that U lower) so that the younger cohorts will decline in well-being over time. Only future waves of data collection will be able to distinguish between these two possibilities, or their combination. It should also be noted that the answer to this question is not predetermined, and in fact depends a good deal on the extent to which policy is shaped to try to better support the well-being of young people.

Some of the other pooled results presented are relatively unsurprising in the context of the existing literature. The well-being literature, focused mostly on life satisfaction/evaluation, has consistently replicated the results with marriage, employment and religious service attendance^5,32,81^, for example, and these patterns were relatively consistent across countries in the GFS. While the analyses here are purely descriptive, other longitudinal, experimental and quasi-experimental studies suggest that each of these factors also has a causal role; they are likely ‘pathways’ to flourishing^5^. What was interesting in this context, however, is how substantially the magnitudes of differences in flourishing related to these demographic factors varied across countries. Future research could aim at trying to understand the explanations for these differences and whether different cross-cultural mechanisms or moderators (or possibly different patterns of confounding) may be at play. Similar considerations pertain to the differences across countries observed in the relations between flourishing and education, immigration and gender.

It is of course challenging to condense all of the varying evidence into a limited number of high-level insights. We will, however, offer three additional, admittedly selected, summary points of interest. First, flourishing is multidimensional, and different countries are flourishing in different ways. While many developed nations report comparatively higher levels of financial security and life evaluation, these same nations are not flourishing in other ways, often reporting lower meaning^49^, pro-sociality^51^ and relationship quality^65^. Japan reports strikingly low scores on many well-being indicators, and this appears to be not purely an artifact of a tendency to report in the middle of 0–10 response scales^82^ since the same patterns hold with binary indicators. Other, often middle-income, nations, such as Indonesia, Argentina, Mexico, Brazil and the Philippines report higher on these other more humanistic aspects of flourishing. The more material and the more humanistic forms of well-being sometimes diverge. While purely descriptive, the negative relationship between meaning and gross domestic product per capita is particularly striking^49^ and has been confirmed in previous work^83^. This may raise important questions about how to carry out economic development in ways that do not compromise meaning and purpose. The claim being made here is not a causal assertion about gross domestic product lowering meaning. Rather, the desired outcome of a society is presumably one with both high levels of economic development and high levels of meaning, and the question is then how to attain this. Similar considerations may pertain to relationships and character.

Second, there can sometimes be complex relationships between adversity and subsequent flourishing. Often adversity is indeed detrimental, and work must be done to address conditions of adversity and suffering. But sometimes these adverse conditions can for some give rise to development and growth and certain forms of flourishing^84,85^. The patterns are, however, complex, both across countries and across outcomes. In some countries, more adverse childhood socioeconomic circumstances were associated with lower levels of adult pain, perhaps suggesting a developed resilience. In the United States and Argentina, difficult childhood financial circumstances were associated with higher adult meaning. These patterns pertain to some countries but not others. However, there is also variation across outcomes. Aggregating across all countries, childhood abuse and feeling like an outsider somewhat surprisingly predicted higher levels of adult volunteering, giving and helping but predicted lower levels of showing love/care for others. The effects on these seemingly closely related outcomes are in opposite directions. More work needs to be done to understand the conditions or responses to adversity and suffering that lead to growth, rather than further decline. While more can and should be done to address suffering and adverse circumstances, some experience of suffering seems ubiquitous and an almost unavoidable part of the human condition^86,87^. In addition to trying to reduce suffering, we should also better seek to understand how we might approach and transform such suffering, when we cannot eliminate it, to enable, when possible, further flourishing^85^, and we should better understand the limits of such approaches.

Third, in both the demographic analyses and in the childhood predictor analyses, religious service attendance was one of the factors most consistently associated with present or subsequent well-being, across countries and across outcomes. This is consistent with much previous literature focused principally on the West^88–92^, but now expanded to a broad range of countries. Service attendance was not beneficially associated with all well-being outcomes. It was associated with slightly higher levels of suffering and of physical pain, which may arise from either differing experiences of it or sensitivity to it. However, with the vast majority of other well-being outcomes, in both the demographic analyses and the childhood predictor analyses, there were beneficial dose–response associations with service attendance, often with a fairly steep gradient. This does not of course guarantee that such associations will be found in all countries, but it is nevertheless notable that they do pertain to many of the 22 GFS countries. While the demographic analyses are purely descriptive, the childhood predictor analyses are intended to provide some evidence for causality. The near ubiquity of these associations is thought-provoking, and this factor is arguably too often neglected in our understanding of well-being and its trends over time. While the present data are insufficient to provide definitive evaluation, it is quite possible that the divergence of material and more humanistic forms of well-being noted in the preceding is in large part due to the declining religiosity of more economically advanced nations. We may need a reconsideration of spiritual pathways to well-being.

The strengths of the GFS are many and include nationally representative samples; broad cultural, geographical and non-Western coverage; large sample sizes; and indicators of numerous well-being and other outcomes along with their determinants. While there are other important multi-country international datasets that are already available (for example, GWP, World Values Survey, International Social Survey Program), the GFS has the advantage of a much wider breadth of well-being constructs and (starting with Wave 2) a longitudinal panel data structure.

The GFS and the analyses presented in this Article, however, undoubtedly also have limitations. While the GFS has a broad range of constructs and indicators and broad geographical coverage, many of the constructs are assessed with a single indicator. Although resulting issues of power are partially offset by large sample sizes, this does not mitigate concerns about validity and conceptual breadth for any given construct. The single indicators were selected on the basis of previous work on longitudinal predictive power and strong correlations with other indicators within a scale^21^, but this does not entirely resolve the concerns. The assessments are also based on self-report survey responses and may be subject to self-report biases and mode effects, which we also comment on in the following. Moreover, such insights principally concern subjective rather than objective aspects of flourishing.

It should also be noted that while the samples within each country were constructed to be approximately nationally representative, the countries themselves do not constitute a random sample of all countries. Rather, as noted, the countries were selected to (1) maximize coverage of the world’s population, (2) ensure geographic, cultural and religious diversity and (3) prioritize feasibility and existing data collection infrastructure. The countries do come from all six populated continents and constitute nearly half of the world’s population (with mainland China included in the second wave of the study, nearly two-thirds). However, no low-income countries are represented, although lower-middle-income, upper-middle-income, and high-income countries are. Results may of course differ in other countries, perhaps especially those with higher levels of conflict or adversity. The meta-analytic means should thus be interpreted in the context of the 22 countries in the study, and these may be higher than global means. Thus, for example, using 2023 GWP data, the mean life evaluation across the 22 GFS countries is 5.91 (95% CI: 5.49, 6.33) whereas the mean across all 142 GWP countries is 5.60 (95% CI: 5.41, 5.78); and further mean differences comparing GWP and GFS may arise due to seasonality and mode effects^21^. Conclusions here should thus be restricted to the 22 GFS countries. The variation of results across countries also makes clear that extrapolation to other countries may be unwarranted. Nevertheless, as noted, some of the patterns appear to be universal across the 22 countries, and for these one might speculate that they may hold more generally, although this is not guaranteed.

With regard to limitations of the analyses presented in this Article, caution is needed in interpreting cross-national differences as these may be influenced by matters of translation^75,76^, different modes of assessment, differing interpretation of items and of response scales, and seasonal effects arising from data being collected in different countries at different times of the year. Table 5 should thus be interpreted as simply providing ordered means across what are somewhat differentially interpreted items, not as ‘rankings.’ In addition, the demographic analyses are purely descriptive and should not be interpreted causally. The childhood predictors analyses constitute a synthetic longitudinal study and may provide some evidence for causality but should be interpreted cautiously. The childhood predictors assessments are retrospective and may be subject to recall bias. Nevertheless, for recall bias to completely explain away the observed associations would require that the effect of adult well-being on biasing retrospective assessments of the childhood predictors would essentially have to be at least as strong as the observed associations themselves^93^, and some of these were quite substantial. The childhood predictors analyses may also be subjected to unmeasured confounding, although these concerns were at least partially addressed through the E-value sensitivity analyses, and in some cases the associations were found to be quite robust. We did not regress current flourishing outcomes contemporaneously on all demographic factors as such analyses would have been purely cross-sectional and yet weaker in terms of capacity for providing causal evidence^94^. However, future waves of data will give further insight into potential causal relationships concerning well-being and its various potential determinants.

The GFS, and these initial results, help provide foundational knowledge for the promotion of societal flourishing. Understanding the distribution of flourishing across the globe, and by demographic group, helps us to understand who needs help, and in what ways. It allows us to identify the groups toward whom interventions or policies might be targeted to improve well-being. Our analyses likewise give insight into how childhood experiences shape well-being, which may be of interest both descriptively and in future intervention and policy development aimed at preventing adverse childhood experiences that impede adult flourishing. One cannot, however, erase the past, and the analyses here also suggest that sometimes adversity can give rise to greater resilience and can be a pathway for growth. Understanding under what circumstances this can and does take place and how it might be facilitated, and its limits, will be an important direction for future research.

The results also raise important questions for the future progress of society. Are we sufficiently investing in the future given the notable flourishing–age gradient, with the youngest groups often faring the most poorly? Can we carry out economic development in ways that do not compromise meaning and purpose and relationships and character, given that many economically developed nations are not faring as well on these measures? With economic development and secularization, have we sometimes been neglecting, or even suppressing, powerful spiritual pathways to flourishing? The very word ‘flourishing’ can arguably be used either as an abstract noun to indicate a state (as per the composite flourishing index) or to suggest a dynamic process of growth^95^. How can each nation grow and flourish? If society is to ultimately pursue flourishing, these questions of age, and of economic development, and of spiritual dynamics need to be taken into consideration.

More remains to be done both to understand and to respond to these questions and challenges. The GFS is an open-access data resource available through the Center for Open Science. In addition to our analyses, we hope many others will access the data and contribute other important insights, insights that might advance our knowledge of and capacity to promote human flourishing. What we measure shapes what we discuss, what we know, what we aim for and the policies put in place to achieve those aims. We hope that the GFS itself, and the understandings that arise from it, will shift discussion and policy toward the promotion of flourishing.

## Methods

We will first describe the methodology for the questionnaire development; then the measures, data collection, and sampling; and finally the analytic methods used both for descriptive statistics and for assessing associations of flourishing with childhood experiences assessed retrospectively.

### Questionnaire development

Development of the questionnaire for the GFS took place over eight distinct phases: (1) selection of core well-being, religion and demographic questions; (2) solicitation of further well-being, social, political, psychological, economic, community and health questions from domain experts (for example, gratitude, social connection, etc.) worldwide; (3) feedback and questionnaire refinement from scholars around the world representing numerous academic disciplines on survey questions and structure; (4) input and questionnaire refinement from experts in multicultural survey research and survey translations; (5) feedback and questionnaire refinement from an open invitation to comment on the survey, posted publicly and sent to numerous listservs, with input from over 150 scholars; (6) further questionnaire refinement via input from Gallup’s survey design specialists; (7) adaptation of items from an interviewer-administered to a self-administered survey instrument using best practices for web survey design to minimize item non response, illogical responses and incomplete responses; and (8) translation into other languages and subsequent confirmation by scholars in several participating countries that translations accurately captured the intended meaning of each question. This was then followed by piloting and cognitive interviews in all of the participating countries and subsequent introduction of retrospective childhood experience questions when it became clear that the intake and annual survey were to be separated in settings not administered by web. During the translation process, Gallup adhered to the TRAPD model (translation, review, adjudication, pretesting and documentation) for cross-cultural survey research^96^. See refs. ^20,21^ for more detailed information on the questionnaire development and the refinement of the survey, and survey items, at each stage.

### Data

Wave 1 of the GFS data included the following countries and territories: Argentina, Australia, Brazil, Egypt, Germany, Hong Kong (Special Administrative Region of China), India, Indonesia, Israel, Japan, Kenya, Mexico, Nigeria, the Philippines, Poland, South Africa, Spain, Sweden, Tanzania, Turkey, United Kingdom and the United States (Fig. 1). The countries were selected to (1) maximize coverage of the world’s population, (2) ensure geographic, cultural and religious diversity and (3) prioritize feasibility and existing data collection infrastructure. Data collection was carried out by Gallup Inc. The study protocol was approved by the institutional review boards of Gallup and of Baylor University. Gallup secures in-country institutional review boards in countries where local authorities request one. Data for Wave 1 were collected principally during 2023, with some countries beginning data collection in late 2022 and exact dates varying by country^97^.

Four additional waves of panel data on the participants will be collected annually from 2024 to 2027. Data from Hong Kong (S.A.R. of China) are available in the first wave of data collection. Data from mainland China were not included in the first data release due to fieldwork delays. The first wave of fieldwork in mainland China began in February 2024, with a second wave in November–December 2024. All Wave 1 and 2 data from mainland China are part of the April 2025 dataset release. The precise sampling design to ensure nationally representative samples varied by country, and further details are available in refs. ^97,98^. Survey items included aspects of well-being such as happiness, health, meaning, character, relationships and financial stability^5^, along with other demographic, social, economic, political, religious, personality, childhood, community, health and well-being variables. Data were also collected concerning retrospective assessments of childhood experiences. The data are freely available to all through the Center for Open Science^99^, upon submission of a pre-registration.

### Study population

The study population consisted of 202,898 adults: ages 18–24 years (*n* = 27,007 (13%)); 25–29 years (*n* = 20,700 (10%)); 30–39 years (*n* = 40,256 (20%)); 40–49 years (*n* = 34,464 (17%)); 50–59 years (*n* = 31,793 (16%)); 60–69 years (*n* = 27,763 (14%)); 70–79 years (*n* = 16,776 (8.3%)); 80 or older (*n* = 4,119 (2.0%)); missing (*n* = 20 (<0.1%)). Of these adults, 98,411 (49%) were male, 103,488 (51%) were female, and 602 (0.3%) indicated other. Data on gender were missing from 397 (0.2%). All participants provided informed consent to participate. Participants were generally compensated US$3–$6 depending on the country.

### Measures

#### Demographics variables

Continuous age was classified as 18–24, 25–29, 30–39, 40–49, 50–59, 60–69, 70–79 or 80 or older. Gender was assessed as male, female or other. Marital status was assessed as single/never married, married, separated, divorced, widowed or domestic partner. Employment was assessed as employed, self-employed, retired, student, homemaker, unemployed and searching, or other. Education was assessed as up to 8 years, 9–15 years or 16+ years. Religious service attendance was assessed as more than once per week, once per week, one to three times per month, a few times per year or never. Immigration status was dichotomously assessed with: ‘Were you born in this country, or not?’ Religious tradition/affiliation was captured with the categories of Christianity, Islam, Hinduism, Buddhism, Judaism, Sikhism, Baha’i, Jainism, Shinto, Taoism, Confucianism, Primal/Animist/Folk religion, Spiritism, African-Derived, some other religion, and no religion/atheist/agnostic; precise response categories varied by country^76^. Racial/ethnic identity was assessed in some, but not all, countries, with response categories varying by country.

#### Childhood variables

Relationship with mother during childhood was assessed with the question: ‘Please think about your relationship with your mother when you were growing up. In general, would you say that relationship was very good, somewhat good, somewhat bad, or very bad?’ Responses were dichotomized to very/somewhat good versus very/somewhat bad. An analogous variable was used for relationship with father. ‘Does not apply’ was treated as a dichotomous control variable for respondents who did not have a mother or father due to death or absence. Parental marital status during childhood was assessed with responses of married, divorced, never married, and one or both had died. Financial status was measured with: ‘Which one of these phrases comes closest to your own feelings about your family’s household income when you were growing up, such as when YOU were around 12 years old?’ Responses were lived comfortably, got by, found it difficult and found it very difficult. Abuse was assessed with yes/no responses to ‘Were you ever physically or sexually abused when you were growing up?’ Participants were separately asked: ‘When you were growing up, did you feel like an outsider in your family?’ Childhood health was assessed by: ‘In general, how was your health when you were growing up? Was it excellent, very good, good, fair or poor?’ Immigration status was assessed with: ‘Were you born in this country, or not?’ Religious service attendance during childhood was assessed with: ‘How often did YOU attend religious services or worship at a temple, mosque, shrine, church or other religious building when YOU were around 12 years old?’ with responses of at least once per week, one to three times per month, less than once per month or never. For additional details on the assessments, see the GFS codebook (https://osf.io/cg76b) or ref. ^20^.

#### Outcome variable(s)

The primary outcome measure in this Article concerns a composite index for individual aspects of flourishing^5^ using two self-report questions in each of six domains: happiness and life satisfaction, physical and mental health, meaning and purpose, character and virtue, close social relationships, and financial and material stability (Table 2). The composite flourishing index is simply a mean of the individual indicators and should be understood as nothing beyond the average of each of the more meaningful domain scores^5^. This is an index—not a scale—and aggregates across a number of disparate aspects of well-being. There is a somewhat arbitrary nature to an index insofar as one could always include more, or fewer, or different items, but ideally a suitable well-being index would have considerable conceptual breadth across numerous domains^100^. This particular assessment has received considerable empirical validation in cross-cultural research^101–104^. Other papers in the special collection focus on specific aspects of well-being or its determinants, including numerous questions that are not included in the 12-item composite. Brief comment is made on the results of these and on when the patterns differ from that of composite flourishing to provide a more comprehensive description of the results. While the flourishing index assesses various individual aspects of flourishing, comment is also made in the following, and in the other papers in the special collection on more community-oriented aspects of flourishing. Also reported in the Supplementary Information are similar analyses to those in the tables but carried out using only the first five domains (excluding financial and material stability) as financial resources are often considered an important determinant of flourishing, rather than a constitutive part of flourishing.

### Statistical analysis

#### Demographic analyses

Descriptive statistics for the full sample, weighted to be nationally representative within each country, were estimated for each of the demographic variables. Nationally representative means for the flourishing index were estimated separately for each country and ordered from highest to lowest along with 95% confidence intervals, standard deviations, Gini coefficients (for assessing inequality in the population distribution of flourishing) and alpha reliability coefficients. Variation in means for the flourishing index across demographic categories were estimated, with all analyses initially conducted by country (Supplementary Tables S1–S22). Primary results in the tables consisted of a random effects meta-analyses^105–107^ of country-specific flourishing means in each specific demographic category along with 95% confidence intervals, standard errors, lower and upper limits of a 95% prediction interval across countries, heterogeneity (τ) and *I*^2^ for evidence concerning variation of flourishing means within a particular demographic variable across countries^108^. Meta-analysis was employed over hierarchical modeling so as not to presume measurement invariance^107,109^. Forest plots of estimates are available in the Supplementary Information (parts 2–4). All meta-analyses were conducted in R^110^ using the metafor package^111^. Within each country, a global test of variation of outcome across levels of each demographic variable was conducted, and a pooled *P* value^112^ across countries reported concerning evidence for variation within any country. Bonferroni corrected *P*-value thresholds are provided on the basis of the number of demographic variables^113,114^. Religious affiliation/tradition and race/ethnicity were used, when available, as control variables within country, and associations by country are presented in Supplementary Tables S1–S29 but are not meta-analyzed since the availability of these response categories varied by country. See ref. ^107^ for more extensive description on the analytic methodology for the demographic analyses for this Article and other papers in the special collection.

#### Childhood predictor analyses

Descriptive statistics for the observed sample, weighted to be nationally representative within country, were estimated for each childhood demographic category. A weighted linear regression model with complex survey adjusted standard errors was fit within each country of composite flourishing on all of the aforementioned childhood predictor variables simultaneously. In the primary analyses, random effects meta-analyses of the regression coefficients^105,106,109^ along with confidence intervals, estimated proportions of effects across countries with effect sizes larger than 0.1 or below −0.1, heterogeneity (τ) and *I*^2^ for evidence concerning effect size variation within a given predictor category across countries are given^108^. Forest plots of estimates are available in the Supplementary Information (part 4). Religious affiliation/tradition and race/ethnicity were used within country as control variables, when available, but these coefficients themselves were not included in the meta-analyses since categories/responses varied by country. Within each country, a global test of association of each childhood predictor variable group with outcome was conducted, and a pooled *P* value^112^ across countries was reported concerning evidence for association within any country. Bonferroni corrected *P*-value thresholds are provided on the basis of the number of childhood predictor variables^113,114^. For each childhood predictor, we calculated E-values to evaluate the sensitivity of results to unmeasured confounding. An E-value is the minimum strength of the association an unmeasured confounder must have with both the outcome and the predictor, above and beyond all measured covariates, for an unmeasured confounder to explain away an association^35^. See ref. ^109^ for a more extensive description on the analytic methodology for the childhood predictor analyses for this Article and other papers in the special collection.

#### Population weighted analyses

As supplementary analyses, population weighted meta-analyses, using 2023 population sizes, were also conducted.

#### Missing data

Missing data on all variables were imputed using multivariate imputation by chained equations, and five imputed datasets were used^115–118^. To account for variation in the assessment of certain variables across countries (for example, religious affiliation/tradition and race/ethnicity), the imputation process was conducted separately in each country. This within-country imputation approach ensured that the imputation models accurately reflected country-specific contexts and assessment methods. Sampling weights were included in the imputation models to account for specific-variable missingness that may have been related to probability of inclusion in the study.

#### Accounting for complex sampling design

The GFS used different sampling schemes across countries based on availability of existing panels and recruitment needs^97^. All analyses accounted for the complex survey design components by including weights, primary sampling units and strata. Additional methodological detail, including accounting for the complex sampling design, is provided elsewhere^97,98^.

All analyses were pre-registered with the Center for Open Science (https://osf.io/registries/gfs) before data access; all code to reproduce analyses are openly available in an online repository^119^.

### Reporting summary

Further information on research design is available in the Nature Portfolio Reporting Summary linked to this article.

## Supplementary information

Supplementary Information Supplementary Figs. S1a–S143b and Tables S1a–S29.

Reporting Summary

## Data Availability

Data for Wave 1 of the Global Flourishing Study is available through the Center for Open Science upon submission of a pre-registration (10.17605/OSF.IO/3JTZ8) and will be openly available without pre-registration beginning 2026^99^. Please see https://www.cos.io/gfs-access-data for more information about data access.

## Code availability

Code for the Global Flourishing Study in multiple software packages is openly available (10.17605/osf.io/vbype).
