# Supplementary material for: The Global Flourishing Study: Study Profile and Initial Results on Flourishing
Source: Nat Ment Health. 2025 Apr 30;3(6):636–53. doi: 10.1038/s44220-025-00423-5 (PMC12165845; doi:10.1038/s44220-025-00423-5)

nature mental health

Supplementary information

https://doi.org/10.1038/s44220-025-00423-5

The Global Flourishing Study: Study Profile and Initial Results on Flourishing

In the format provided by the authors and unedited

GFS Online Supplement

Important Notes and Caveats

This online supplement to the Global Flourishing Study paper reporting on country-specific estimates of composite flourishing (both the Secure Flourishing Index and the Flourishing Index), population weighted meta-analyses, and supplemental forest plots. The analyses have several important caveats to interpretation for the demographic variation analyses and childhood predictor analyses.

For the demographic variation analyses, estimating the within country group means can be unstable when the group size is small (<1%) of the country sample size. In such cases, the uncertainty in the estimate leads to a multiple imputation adjusted degrees of freedom less than 1. This means there is not enough information to evaluate the uncertainty in the estimate. We flagged such cases with a "*". Interval estimates for the mean of continuous outcomes (ranged 0-10) were based on a Wald-type confidence interval where items were treated as continuous which could rarely lead to intervals exceeding the bounds of the observed range of values; in such cases we have truncate the limits to be within the range of observed values, and such cases are marked with a "ǂ".

For the childhood predictor analyses, analyses are based on data using a retrospective (recall) approach to obtain information about the respondents' lives at age 12. Several the childhood characteristics such as relationship quality with parents, subjective financial status growing up, etc. are necessarily highly related. This led to multicollinearity issues so some effects may be unstable. Additionally, in rare instances the confidence interval of the effect estimate can contradict the reported global p-value (e.g., for the single-category effects of relationship with mother). In such cases, the reported confidence interval is more robust with corrected degrees of freedom from the pooling across multiple imputations, whereas the global p-value is based on a Wald-type test and is less robust to uncertainty attributable to multiple imputation.

Comparing results across countries should be done with caution due to possible measurement non-invariance, seasonality effects, differences in translation, differences in the quality of weights across countries.

Table S1a. Nationally representative descriptive statistics for Argentina

**Characteristic**	**N = 6,724**[1]
**Age group**	
1998-2005; age 18-24	1,108 (16%)
1993-1998; age 25-29	719 (11%)
1983-1993; age 30-39	1,432 (21%)
1973-1983; age 40-49	1,254 (19%)
1963-1973; age 50-59	1,014 (15%)
1953-1963; age 60-69	730 (11%)
1943-1953; age 70-79	356 (5.3%)
1943 or earlier; age 80+	112 (1.7%)
(Missing)	0 (0%)
**Gender**	
Male	3,143 (47%)
Female	3,542 (53%)
Other	21 (0.3%)
(Missing)	18 (0.3%)
**Race/Ethnicity**	
Asian	43 (0.6%)
Black	95 (1.4%)
Indigenous	129 (1.9%)
Mestizo(a)	1,801 (27%)
Mullato(a)	75 (1.1%)
Other	104 (1.5%)
White	3,406 (51%)
(Missing)	1,070 (16%)
**Marital status**	
Married	1,565 (23%)
Separated	455 (6.8%)
Divorced	321 (4.8%)
Widowed	401 (6.0%)
Single, never married	2,381 (35%)
Domestic Partner	1,514 (23%)
(Missing)	88 (1.3%)
**Employment**	
Employed for an employer	2,440 (36%)
Self-employed	1,748 (26%)
Retired	773 (11%)
Student	354 (5.3%)
Homemaker	639 (9.5%)
Unemployed and looking for a job	569 (8.5%)
None of these/Other	179 (2.7%)
(Missing)	22 (0.3%)
**Religious service attendance**	
More than 1/week	532 (7.9%)
1/week	773 (12%)
1-3/month	461 (6.8%)
A few times a year	1,949 (29%)
Never	2,982 (44%)
(Missing)	27 (0.4%)
**Education**	
Up to 8 years	2,263 (34%)
9-15 years	3,823 (57%)
16+ years	635 (9.4%)
(Missing)	3 (<0.1%)
**Immigration status**	
Born in this country	6,346 (94%)
Born in another country	348 (5.2%)
(Missing)	29 (0.4%)

Characteristic	N = 6,724[1]
**Religious affiliation as an adult (now)**	
Christianity	4,992 (74%)
Islam	9 (0.1%)
Hinduism	6 (<0.1%)
Buddhism	35 (0.5%)
Judaism	40 (0.6%)
Sikhism	0 (<0.1%)
Baha'i	0 (0%)
Jainism	0 (0%)
Shinto	0 (0%)
Taoism	2 (<0.1%)
Confucianism	0 (<0.1%)
Primal, Animist, or Folk religion	19 (0.3%)
Spiritism	0 (0%)
Umbanda, Candomble, and other African-derived religions	0 (0%)
Chinese folk/traditional religion	0 (0%)
Some other religion	156 (2.3%)
No religion/Atheist/Agnostic	1,352 (20%)
(Missing)	111 (1.7%)
**Parent marital status**	
Parents married	4,110 (61%)
Divorced	637 (9.5%)
Parents were never married	1,368 (20%)
One or both parents had died	199 (3.0%)
(Missing)	410 (6.1%)
**Age 12 religious service attendance**	
At least 1/week	2,601 (39%)
1-3/month	1,204 (18%)
<1/month	1,059 (16%)
Never	1,808 (27%)
(Missing)	53 (0.8%)
**Relationship with mother**	
Very good	4,463 (66%)
Somewhat good	1,436 (21%)
Somewhat bad	299 (4.4%)
Very bad	216 (3.2%)
Does not apply	273 (4.1%)
(Missing)	36 (0.5%)
**Relationship with father**	
Very good	3,612 (54%)
Somewhat good	1,537 (23%)
Somewhat bad	440 (6.5%)
Very bad	401 (6.0%)
Does not apply	694 (10%)
(Missing)	39 (0.6%)
**Outsider growing up**	
Yes	1,165 (17%)
No	5,458 (81%)
(Missing)	101 (1.5%)
**Self-reported history of abuse**	
Yes	1,302 (19%)
No	5,271 (78%)
(Missing)	151 (2.2%)
**Self-rated health growing up**	
Excellent	2,402 (36%)
Very good	1,819 (27%)
Good	1,830 (27%)
Fair	505 (7.5%)
Poor	156 (2.3%)

**Characteristic**	**N = 6,724**[1]
(Missing)	12 (0.2%)
**Subjective financial status of family growing up**	
Lived comfortably	2,042 (30%)
Got by	2,305 (34%)
Found it difficult	1,789 (27%)
Found it very difficult	569 (8.5%)
(Missing)	19 (0.3%)
**Religious affiliation at age 12**	
Christianity	5,805 (86%)
Islam	11 (0.2%)
Hinduism	2 (<0.1%)
Buddhism	3 (<0.1%)
Judaism	51 (0.8%)
Sikhism	5 (<0.1%)
Baha'i	0 (0%)
Jainism	0 (0%)
Shinto	0 (0%)
Taoism	1 (<0.1%)
Confucianism	0 (0%)
Primal, Animist, or Folk religion	17 (0.2%)
Spiritism	0 (0%)
Umbanda, Candomble, and other African-derived religions	0 (0%)
Chinese folk/traditional religion	0 (0%)
Some other religion	10 (0.2%)
No religion/Atheist/Agnostic	697 (10%)
(Missing)	122 (1.8%)

[1] n (%)

Table S1b. Means by demographic category for Argentina (N=6724)

Variable	Category	Secure Flourishing Index				Flourishing Index			
		Mean	95% CI	SE	Global p-value	Mean	95% CI	SE	Global p-value
Age group	18-24	7.48	(7.35,7.62)	0.07	1.85e-08	6.90	(6.77,7.04)	0.07	3.96e-09
	25-29	7.70	(7.53,7.86)	0.09		6.98	(6.81,7.15)	0.09	
	30-39	7.68	(7.57,7.80)	0.06		6.97	(6.86,7.08)	0.05	
	40-49	7.97	(7.86,8.08)	0.06		7.28	(7.17,7.39)	0.05	
	50-59	8.01	(7.90,8.11)	0.05		7.33	(7.22,7.44)	0.06	
	60-69	7.83	(7.66,8.00)	0.09		7.26	(7.10,7.42)	0.08	
	70-79	7.92	(7.69,8.16)	0.12		7.41	(7.18,7.64)	0.12	
	80 or older	8.04	(7.57,8.50)	0.23		7.62	(7.14,8.10)	0.24	
Gender	Female	7.78	(7.71,7.85)	0.04	2.98e-05	7.05	(6.98,7.11)	0.03	3.22e-12
	Male	7.80	(7.73,7.88)	0.04		7.25	(7.17,7.32)	0.04	
	Other	6.71	(6.20,7.22)	0.24		6.07	(5.70,6.44)	0.17	
Marital status	Divorced	7.89	(7.71,8.07)	0.09	1.31e-10	7.30	(7.12,7.47)	0.09	2.49e-10
	Domestic partner	7.85	(7.76,7.95)	0.05		7.14	(7.04,7.24)	0.05	
	Married	8.05	(7.95,8.16)	0.05		7.40	(7.29,7.50)	0.05	
	Separated	7.76	(7.55,7.97)	0.11		7.11	(6.90,7.31)	0.11	
	Single/Never been married	7.55	(7.45,7.64)	0.05		6.92	(6.83,7.01)	0.05	
	Widowed	7.92	(7.73,8.11)	0.10		7.32	(7.13,7.51)	0.10	
Employment	Employed for an employer	7.85	(7.77,7.93)	0.04	1.47e-08	7.21	(7.13,7.28)	0.04	1.78e-10
	Homemaker	7.93	(7.75,8.11)	0.09		7.04	(6.87,7.20)	0.08	
	None of these/Other	7.32	(6.93,7.71)	0.20		6.58	(6.22,6.94)	0.18	
	Retired	7.88	(7.72,8.04)	0.08		7.29	(7.14,7.45)	0.08	
	Self-employed	7.88	(7.79,7.98)	0.05		7.28	(7.19,7.38)	0.05	
	Student	7.36	(7.16,7.55)	0.10		6.88	(6.69,7.08)	0.10	
	Unemployed and looking for a job	7.39	(7.18,7.59)	0.10		6.62	(6.42,6.83)	0.10	
Religious service attendance	A few times a year	7.87	(7.78,7.96)	0.05	< 2e-16	7.18	(7.09,7.26)	0.04	< 2e-16
	More than once a week	8.66	(8.51,8.81)	0.08		7.96	(7.79,8.13)	0.09	

Variable	Category	Secure Flourishing Index				Flourishing Index			
		Mean	95% CI	SE	Global p-value	Mean	95% CI	SE	Global p-value
	Never	7.52	(7.44,7.59)	0.04		6.89	(6.82,6.97)	0.04	
	Once a week	8.07	(7.92,8.22)	0.08		7.42	(7.27,7.58)	0.08	
	One to three times a month	7.74	(7.54,7.94)	0.10		7.11	(6.93,7.30)	0.10	
Education	Up to 8	7.95	(7.84,8.07)	0.06	5.04e-04	7.22	(7.11,7.33)	0.06	0.005
	9 to 15	7.74	(7.62,7.86)	0.06		7.25	(7.13,7.37)	0.06	
	16+	7.70	(7.64,7.76)	0.03		7.07	(7.01,7.12)	0.03	
Immigration status	Born in another country	7.87	(7.64,8.11)	0.12	0.469	7.20	(6.97,7.44)	0.12	0.553
	Born in this country	7.78	(7.73,7.84)	0.03		7.13	(7.08,7.18)	0.03	
Religious affiliation	Buddhism	7.78	(7.36,8.19)	0.20	< 2e-16	7.25	(6.79,7.72)	0.22	< 2e-16
	Christianity	7.91	(7.85,7.97)	0.03		7.22	(7.17,7.28)	0.03	
	Hinduism	5.94	*	*		5.86	*	*	
	Islam	8.32	(5.64,10.00ǂ)	1.17		8.24	(5.72,10.00ǂ)	1.10	
	Judaism	7.47	(7.04,7.90)	0.21		7.11	(6.64,7.58)	0.23	
	No religion/Atheist/ Agnostic	7.34	(7.22,7.46)	0.06		6.79	(6.67,6.91)	0.06	
	Primal, Animist, or Folk religion	8.25	(6.85,9.65)	0.56		7.89	(6.32,9.46)	0.63	
	Sikhism	7.50	*	*		7.00	*	*	
	Some other religion	7.92	(7.56,8.29)	0.19		7.23	(6.89,7.56)	0.17	
	Taoism	7.23	*	*		7.10	*	*	
	Confucianism	6.40	*	*		6.50	*	*	

Note. N=6724; p < .007 = 0.05/7 (Bonferroni corrected p-value significance threshold); Mean, estimated group mean; CI, confidence interval for the mean within group; SE, complex survey adjusted standard error or the mean; Global p-value, two-tailed Wald-type test of whether there is evidence of any differences in mean scores among groups of a demographic characteristic. *Estimate is not reported due to multiple-imputation and complex survey adjusted degrees of freedom was less than 1.00 leading to insufficient information to provide an estimate of the uncertainty in the estimate. These groups are removed when estimating the global test of mean differences.

Table S1c. Childhood predictors regression analysis results for Argentina (N=6724)

		Secure Flourishing Index					Flourishing Index				
Variable	Category	Est	95% CI	SE	Est/SD	Global p-value	Est	95% CI	SE	Est/SD	Global p-value
Relationship with mother	(Ref: Very bad/somewhat bad)					8.55e-05					2.13e-05
	Very good/somewhat good	0.41	(0.20,0.62)	0.11	0.28		0.48	(0.25,0.70)	0.12	0.33	
Relationship with father	(Ref: Very bad/somewhat bad)					0.032					0.060
	Very good/somewhat good	0.17	(0.01,0.33)	0.08	0.11		0.16	(-0.01,0.32)	0.09	0.11	
Parent marital status	(Ref: Parents married)					0.597					0.415
	Divorced	0.02	(-0.17,0.20)	0.09	0.01		0.01	(-0.17,0.19)	0.09	0.01	
	Parents were never married	0.07	(-0.07,0.22)	0.07	0.05		0.10	(-0.04,0.25)	0.07	0.07	
	One or both parents had died	0.11	(-0.19,0.42)	0.15	0.08		0.12	(-0.18,0.42)	0.15	0.08	
Subjective financial status of family growing up	(Ref: Got by)					1.14e-07					4.35e-07
	Lived comfortably	0.26	(0.14,0.37)	0.06	0.17		0.29	(0.18,0.40)	0.06	0.20	
	Found it difficult	-0.09	(-0.22,0.03)	0.06	-0.06		-0.01	(-0.14,0.11)	0.07	-0.01	
	Found it very difficult	-0.01	(-0.22,0.21)	0.11	-0.00		0.19	(-0.03,0.41)	0.11	0.13	
Abuse	(Ref: No)					3.91e-05					4.27e-06
	Yes	-0.28	(-0.42,-0.15)	0.07	-0.19		-0.33	(-0.47,-0.19)	0.07	-0.22	

		Secure Flourishing Index					Flourishing Index				
Variable	Category	Est	95% CI	SE	Est/SD	Global p-value	Est	95% CI	SE	Est/SD	Global p-value
Outsider growing up	(Ref: No)					0.001					7.32e-04
	Yes	-0.26	(-0.42,-0.09)	0.08	-0.17		-0.28	(-0.45,-0.10)	0.09	-0.19	
Self-rated health growing up	(Ref: Good)					3.00e-13					6.79e-14
	Excellent	0.40	(0.27,0.52)	0.07	0.27		0.42	(0.28,0.55)	0.07	0.28	
	Very good	0.10	(-0.03,0.23)	0.06	0.07		0.10	(-0.04,0.23)	0.07	0.07	
	Fair	-0.09	(-0.31,0.13)	0.11	-0.06		-0.09	(-0.31,0.14)	0.12	-0.06	
	Poor	-0.60	(-1.03,-0.18)	0.22	-0.41		-0.63	(-1.09,-0.18)	0.23	-0.43	
Immigration status	(Ref: Born in this country)					0.300					0.334
	Born in another country	0.12	(-0.10,0.34)	0.11	0.08		0.11	(-0.12,0.34)	0.12	0.08	
Age 12 religious service attendance	(Ref: Never)					0.059					0.010
	At least 1/week	0.17	(0.04,0.31)	0.07	0.12		0.18	(0.04,0.32)	0.07	0.12	
	1-3/month	0.06	(-0.09,0.22)	0.08	0.04		0.01	(-0.15,0.16)	0.08	0.01	
	< 1/month	0.08	(-0.06,0.23)	0.08	0.06		-0.01	(-0.16,0.15)	0.08	-0.00	
Year of birth	(Ref: 1998-2005; current age: 18-24)					9.03e-09					7.27e-08
	1993-1998; age 25-29	0.03	(-0.17,0.23)	0.10	0.02		0.14	(-0.07,0.34)	0.10	0.09	
	1983-1993; age 30-39	0.06	(-0.10,0.22)	0.08	0.04		0.17	(-0.00,0.34)	0.09	0.11	
	1973-1983; age 40-49	0.37	(0.20,0.53)	0.09	0.25		0.44	(0.27,0.62)	0.09	0.30	
	1963-1973; age 50-59	0.44	(0.27,0.62)	0.09	0.30		0.50	(0.33,0.68)	0.09	0.34	

Variable	Category	Secure Flourishing Index Est	95% CI	SE	Est/SD	Global p-value	Flourishing Index Est	95% CI	SE	Est/SD	Global p-value
	1953-1963; age 60-69	0.33	(0.12,0.53)	0.11	0.22		0.29	(0.08,0.50)	0.11	0.20	
	1943-1953; age 70-79	0.48	(0.23,0.73)	0.13	0.32		0.39	(0.13,0.64)	0.13	0.26	
	1943 or earlier; age 80+	0.56	(0.10,1.03)	0.24	0.38		0.32	(-0.11,0.76)	0.22	0.22	
Gender	(Ref: Male)					0.002					0.001
	Female	-0.10	(-0.19,0.00)	0.05	-0.06		0.09	(-0.01,0.19)	0.05	0.06	
	Other	-0.70	(-1.13,-0.27)	0.22	-0.47		-0.55	(-0.93,-0.16)	0.19	-0.37	
Religious affiliation	(Ref: No religion/Atheist/Agnostic)					0.940					0.488
	Christianity	0.01	(-0.17,0.20)	0.10	0.01		0.09	(-0.10,0.29)	0.10	0.06	
	Collapsed affiliations with prevalence<3%	0.05	(-0.42,0.51)	0.24	0.03		-0.06	(-0.48,0.37)	0.22	-0.04	
Race/ethnicity	(Ref: Plurality group)					0.267					0.078
	Non-plurality groups	0.06	(-0.05,0.16)	0.05	0.04		0.09	(-0.01,0.20)	0.05	0.06	

Note. N=6724; p < .004 (Bonferroni corrected threshold); Est., estimated effect of childhood predictor on flourishing score; CI, confidence interval; SE, standard error of the estimated effect; Est/SD, a more standardized measure of effect size--estimated effect of flourishing divided by standard deviation of flourishing--leads to the interpretation, for those with the given status (e.g., those with a good/very good relationship with mother compared to those with bad/very bad) are 0.XX standard deviations higher/lower on flourishing; the Global p-value corresponds to the two-sided joint parameter Wald-type test of whether any of the levels' parameters are non-zero, for history of abuse, outsider, relationship wtih mother/father, this is test of whether the estimated effect is non-zero, for multiple-category predictors (age, health, financial status), this is a joint test of whether any of these effects are non-zero. Note the confidence interval of the effect estimate can contradict the reported global p-value (e.g., for the single-category effects of relationship with mother). In such cases, the reported confidence interval is more robust with corrected degrees of freedom from the pooling across multiple imputations, whereas the global p-value is based on a Wald-type test and is less robust to uncertainty attributable to multiple imputation.

Table S1d. Sensitivity to unmeasured confounding of childhood predictors in Argentina (N=6724)

Variable	Category	Secure Flourishing Index		Flourishing Index	
		E-value for Estimate	E-value for 95% CI	E-value for Estimate	E-value for 95% CI
Relationship with mother	(Ref: Very bad/somewhat bad)				
	Very good/somewhat good	1.90	1.52	2.01	1.61
Relationship with father	(Ref: Very bad/somewhat bad)				
	Very good/somewhat good	1.46	1.08	1.43	1.00
Parent marital status	(Ref: Parents married)				
	Divorced	1.12	1.00	1.09	1.00
	Parents were never married	1.27	1.00	1.33	1.00
	One or both parents had died	1.35	1.00	1.37	1.00
Subjective financial status of family growing up	(Ref: Got by)				
	Lived comfortably	1.63	1.42	1.67	1.47
	Found it difficult	1.31	1.00	1.11	1.00
	Found it very difficult	1.07	1.00	1.50	1.00
Abuse	(Ref: No)				
	Yes	1.67	1.42	1.74	1.49
Outsider growing up	(Ref: No)				
	Yes	1.62	1.31	1.65	1.33
Self-rated health growing up	(Ref: Good)				
	Excellent	1.88	1.65	1.90	1.66
	Very good	1.33	1.00	1.32	1.00
	Fair	1.31	1.00	1.29	1.00
	Poor	2.27	1.49	2.31	1.48
Immigration status	(Ref: Born in this country)				
	Born in another country	1.36	1.00	1.35	1.00
Age 12 religious service attendance	(Ref: Never)				
	At least 1/week	1.47	1.20	1.47	1.19
	1-3/month	1.25	1.00	1.08	1.00
	< 1/month	1.29	1.00	1.07	1.00
Year of birth	(Ref: 1998-2005; current age: 18-24)				
	1993-1998; age 25-29	1.16	1.00	1.39	1.00
	1983-1993; age 30-39	1.24	1.00	1.45	1.00

Variable	Category	Secure Flourishing Index		Flourishing Index	
		E-value for Estimate	E-value for 95% CI	E-value for Estimate	E-value for 95% CI
	1973-1983; age 40-49	1.82	1.52	1.95	1.65
	1963-1973; age 50-59	1.96	1.64	2.06	1.74
	1953-1963; age 60-69	1.75	1.37	1.67	1.27
	1943-1953; age 70-79	2.03	1.58	1.85	1.39
	1943 or earlier; age 80+	2.19	1.33	1.74	1.00
Gender	(Ref: Male)				
	Female	1.32	1.00	1.29	1.00
	Other	2.45	1.64	2.14	1.45
Religious affiliation	(Ref: No religion/Atheist/Agnostic)				
	Christianity	1.10	1.00	1.30	1.00
	Collapsed affiliations with prevalence<3%	1.21	1.00	1.23	1.00
Race/ethnicity	(Ref: Plurality group)				
	Non-plurality groups	1.23	1.00	1.30	1.00

Table S2a. Nationally representative descriptive statistics for Australia

Characteristic	N = 3,844[1]
**Age group**	
1998-2005; age 18-24	345 (9.0%)
1993-1998; age 25-29	282 (7.3%)
1983-1993; age 30-39	641 (17%)
1973-1983; age 40-49	618 (16%)
1963-1973; age 50-59	691 (18%)
1953-1963; age 60-69	589 (15%)
1943-1953; age 70-79	498 (13%)
1943 or earlier; age 80+	178 (4.6%)
(Missing)	2 (<0.1%)
**Gender**	
Male	1,861 (48%)
Female	1,941 (50%)
Other	36 (0.9%)
(Missing)	6 (0.2%)
**Race/Ethnicity**	
Aboriginal	53 (1.4%)
Australian	1,946 (51%)
Australian British/European	1,047 (27%)
Chinese	75 (1.9%)
Indian	58 (1.5%)
Japanese	1 (<0.1%)
Malay	11 (0.3%)
New Zealander	91 (2.4%)
Other	163 (4.2%)
Other European	357 (9.3%)
Russian	7 (0.2%)
Samoan	4 (0.1%)
Sinhalese	1 (<0.1%)
Spanish	2 (<0.1%)
Sri Lankan Moor	1 (<0.1%)
Sri Lankan Tamil	7 (0.2%)
Vietnamese	7 (0.2%)
(Missing)	14 (0.4%)
**Marital status**	
Married	1,797 (47%)
Separated	158 (4.1%)
Divorced	332 (8.6%)
Widowed	215 (5.6%)
Single, never married	855 (22%)
Domestic Partner	450 (12%)
(Missing)	38 (1.0%)
**Employment**	
Employed for an employer	1,881 (49%)
Self-employed	380 (9.9%)
Retired	912 (24%)
Student	190 (5.0%)
Homemaker	137 (3.6%)
Unemployed and looking for a job	134 (3.5%)
None of these/Other	206 (5.4%)
(Missing)	4 (0.1%)
**Religious service attendance**	
More than 1/week	162 (4.2%)
1/week	299 (7.8%)
1-3/month	135 (3.5%)
A few times a year	656 (17%)
Never	2,584 (67%)
(Missing)	7 (0.2%)

**Characteristic**	**N = 3,844**[1]
**Education**	
Up to 8 years	70 (1.8%)
9-15 years	2,434 (63%)
16+ years	1,330 (35%)
(Missing)	10 (0.3%)
**Immigration status**	
Born in this country	2,953 (77%)
Born in another country	885 (23%)
(Missing)	6 (0.2%)
**Religious affiliation as an adult (now)**	
Christianity	1,592 (41%)
Islam	45 (1.2%)
Hinduism	31 (0.8%)
Buddhism	36 (0.9%)
Judaism	26 (0.7%)
Sikhism	8 (0.2%)
Baha'i	7 (0.2%)
Jainism	0 (0%)
Shinto	0 (0%)
Taoism	5 (0.1%)
Confucianism	0 (0%)
Primal, Animist, or Folk religion	23 (0.6%)
Spiritism	0 (0%)
Umbanda, Candomble, and other African-derived religions	0 (0%)
Chinese folk/traditional religion	0 (0%)
Some other religion	39 (1.0%)
No religion/Atheist/Agnostic	2,020 (53%)
(Missing)	15 (0.4%)
**Parent marital status**	
Parents married	3,048 (79%)
Divorced	462 (12%)
Parents were never married	187 (4.9%)
One or both parents had died	96 (2.5%)
(Missing)	52 (1.4%)
**Age 12 religious service attendance**	
At least 1/week	1,362 (35%)
1-3/month	486 (13%)
<1/month	600 (16%)
Never	1,307 (34%)
(Missing)	90 (2.3%)
**Relationship with mother**	
Very good	2,554 (66%)
Somewhat good	925 (24%)
Somewhat bad	218 (5.7%)
Very bad	107 (2.8%)
Does not apply	32 (0.8%)
(Missing)	7 (0.2%)
**Relationship with father**	
Very good	2,032 (53%)
Somewhat good	1,144 (30%)
Somewhat bad	315 (8.2%)
Very bad	196 (5.1%)
Does not apply	148 (3.9%)
(Missing)	9 (0.2%)
**Outsider growing up**	
Yes	756 (20%)
No	3,062 (80%)
(Missing)	26 (0.7%)
**Self-reported history of abuse**	

**Characteristic**	**N = 3,844**[1]
Yes	995 (26%)
No	2,790 (73%)
(Missing)	59 (1.5%)
**Self-rated health growing up**	
Excellent	1,736 (45%)
Very good	1,087 (28%)
Good	603 (16%)
Fair	308 (8.0%)
Poor	106 (2.8%)
(Missing)	4 (<0.1%)
**Subjective financial status of family growing up**	
Lived comfortably	1,756 (46%)
Got by	1,496 (39%)
Found it difficult	422 (11%)
Found it very difficult	154 (4.0%)
(Missing)	16 (0.4%)
**Religious affiliation at age 12**	
Christianity	2,678 (70%)
Islam	48 (1.2%)
Hinduism	39 (1.0%)
Buddhism	16 (0.4%)
Judaism	29 (0.8%)
Sikhism	6 (0.2%)
Baha'i	5 (0.1%)
Jainism	0 (0%)
Shinto	0 (0%)
Taoism	1 (<0.1%)
Confucianism	0 (0%)
Primal, Animist, or Folk religion	4 (<0.1%)
Spiritism	0 (0%)
Umbanda, Candomble, and other African-derived religions	0 (0%)
Chinese folk/traditional religion	0 (0%)
Some other religion	8 (0.2%)
No religion/Atheist/Agnostic	990 (26%)
(Missing)	21 (0.5%)

[1]n (%)

Table S2b. Means by demographic category for Australia (N=3844)

Variable	Category	Secure Flourishing Index				Flourishing Index			
		Mean	95% CI	SE	Global p-value	Mean	95% CI	SE	Global p-value
Age group	18-24	6.32	(6.06,6.57)	0.13	< 2e-16	6.28	(6.03,6.53)	0.13	< 2e-16
	25-29	6.52	(6.25,6.80)	0.14		6.46	(6.19,6.73)	0.14	
	30-39	6.69	(6.51,6.87)	0.09		6.70	(6.52,6.88)	0.09	
	40-49	6.90	(6.74,7.06)	0.08		6.81	(6.65,6.98)	0.09	
	50-59	6.88	(6.73,7.03)	0.08		6.88	(6.72,7.03)	0.08	
	60-69	7.34	(7.22,7.47)	0.07		7.36	(7.23,7.49)	0.06	
	70-79	7.81	(7.67,7.95)	0.07		7.87	(7.73,8.00)	0.07	
	80 or older	8.06	(7.88,8.23)	0.09		8.12	(7.94,8.29)	0.09	
Gender	Female	7.04	(6.95,7.14)	0.05	0.001	7.03	(6.93,7.12)	0.05	0.003
	Male	7.01	(6.92,7.11)	0.05		7.02	(6.92,7.12)	0.05	
	Other	5.93	(5.31,6.55)	0.30		5.85	(5.15,6.54)	0.34	
Marital status	Divorced	6.87	(6.67,7.08)	0.10	< 2e-16	6.85	(6.65,7.05)	0.10	< 2e-16
	Domestic partner	6.95	(6.75,7.14)	0.10		6.90	(6.70,7.10)	0.10	
	Married	7.48	(7.40,7.56)	0.04		7.49	(7.41,7.57)	0.04	
	Separated	6.25	(5.83,6.66)	0.21		6.09	(5.67,6.51)	0.21	
	Single/Never been married	6.14	(5.98,6.30)	0.08		6.14	(5.98,6.31)	0.08	
	Widowed	7.60	(7.38,7.81)	0.11		7.63	(7.41,7.85)	0.11	
Employment	Employed for an employer	6.95	(6.87,7.04)	0.04	< 2e-16	6.95	(6.86,7.03)	0.04	< 2e-16
	Homemaker	6.72	(6.29,7.15)	0.22		6.62	(6.16,7.09)	0.23	
	None of these/Other	5.89	(5.50,6.27)	0.19		5.77	(5.40,6.13)	0.19	
	Retired	7.63	(7.51,7.74)	0.06		7.68	(7.57,7.79)	0.06	
	Self-employed	7.38	(7.20,7.57)	0.09		7.37	(7.18,7.56)	0.10	
	Student	6.32	(5.98,6.65)	0.17		6.33	(5.99,6.66)	0.17	
	Unemployed and looking for a job	5.82	(5.33,6.30)	0.25		5.66	(5.17,6.15)	0.25	
Religious service attendance	A few times a year	7.12	(6.97,7.26)	0.07	< 2e-16	7.06	(6.92,7.21)	0.07	2.22e-16
	More than once a week	7.95	(7.68,8.21)	0.13		7.94	(7.66,8.21)	0.14	

		Secure Flourishing Index				Flourishing Index			
Variable	Category	Mean	95% CI	SE	Global p-value	Mean	95% CI	SE	Global p-value
	Never	6.87	(6.78,6.95)	0.04		6.88	(6.79,6.96)	0.04	
	Once a week	7.57	(7.36,7.77)	0.10		7.56	(7.35,7.76)	0.11	
	One to three times a month	7.09	(6.78,7.41)	0.16		7.09	(6.76,7.41)	0.17	
Education	Up to 8	7.64	(7.06,8.22)	0.29	1.11e-05	7.58	(6.98,8.18)	0.30	2.07e-08
	9 to 15	7.18	(7.11,7.26)	0.04		7.23	(7.16,7.31)	0.04	
	16+	6.91	(6.82,7.01)	0.05		6.88	(6.78,6.97)	0.05	
Immigration status	Born in another country	7.21	(7.08,7.33)	0.06	9.99e-04	7.24	(7.12,7.37)	0.06	5.43e-05
	Born in this country	6.96	(6.88,7.04)	0.04		6.94	(6.86,7.02)	0.04	
Religious affiliation	Baha'i	8.71	*	*	5.55e-16	8.72	*	*	< 2e-16
	Buddhism	7.23	(6.72,7.75)	0.25		7.21	(6.68,7.74)	0.26	
	Christianity	7.33	(7.24,7.43)	0.05		7.30	(7.20,7.39)	0.05	
	Hinduism	7.04	(6.07,8.01)	0.46		7.00	(6.14,7.87)	0.41	
	Islam	6.79	(5.97,7.60)	0.40		6.81	(6.03,7.60)	0.38	
	Judaism	7.12	(6.59,7.66)	0.25		7.14	(6.61,7.67)	0.24	
	No religion/Atheist/ Agnostic	6.77	(6.68,6.87)	0.05		6.80	(6.70,6.89)	0.05	
	Primal, Animist, or Folk religion	6.41	(5.39,7.43)	0.45		5.94	(4.94,6.93)	0.44	
	Sikhism	7.07	*	*		7.36	*	*	
	Some other religion	6.86	(5.80,7.93)	0.52		6.64	(5.51,7.77)	0.55	
	Taoism	7.88	*	*		7.99	*	*	

Note. N=3844; p < .007 = 0.05/7 (Bonferroni corrected p-value significance threshold); Mean, estimated group mean; CI, confidence interval for the mean within group; SE, complex survey adjusted standard error or the mean; Global p-value, two-tailed Wald-type test of whether there is evidence of any differences in mean scores among groups of a demographic characteristic. *Estimate is not reported due to multiple-imputation and complex survey adjusted degrees of freedom was less than 1.00 leading to insufficient information to provide an estimate of the uncertainty in the estimate. These groups are removed when estimating the global test of mean differences.

Table S2c. Childhood predictors regression analysis results for Australia (N=3844)

Variable	Category	Secure Flourishing Index					Flourishing Index				
		Est	95% CI	SE	Est/SD	Global p-value	Est	95% CI	SE	Est/SD	Global p-value
Relationship with mother	(Ref: Very bad/somewhat bad)					0.763					0.951
	Very good/somewhat good	-0.04	(-0.32,0.23)	0.14	-0.03		0.00	(-0.28,0.29)	0.15	0.00	
Relationship with father	(Ref: Very bad/somewhat bad)					0.363					0.149
	Very good/somewhat good	0.10	(-0.12,0.33)	0.11	0.06		0.16	(-0.06,0.39)	0.11	0.10	
Parent marital status	(Ref: Parents married)					0.060					0.008
	Divorced	-0.03	(-0.25,0.20)	0.11	-0.02		0.01	(-0.21,0.24)	0.11	0.01	
	Parents were never married	0.27	(-0.09,0.63)	0.18	0.17		0.39	(0.04,0.75)	0.18	0.25	
	One or both parents had died	0.39	(0.05,0.73)	0.17	0.24		0.48	(0.13,0.82)	0.17	0.30	
Subjective financial status of family growing up	(Ref: Got by)					0.036					0.170
	Lived comfortably	0.11	(-0.02,0.23)	0.07	0.07		0.08	(-0.05,0.21)	0.07	0.05	
	Found it difficult	-0.04	(-0.24,0.17)	0.11	-0.02		0.02	(-0.19,0.22)	0.10	0.01	
	Found it very difficult	-0.37	(-0.73,-0.01)	0.18	-0.23		-0.32	(-0.70,0.06)	0.19	-0.20	
Abuse	(Ref: No)					7.88e-09					1.33e-07
	Yes	-0.47	(-0.63,-0.31)	0.08	-0.30		-0.43	(-0.60,-0.27)	0.08	-0.27	

		Secure Flourishing Index					Flourishing Index				
Variable	Category	Est	95% CI	SE	Est/SD	Global p-value	Est	95% CI	SE	Est/SD	Global p-value
Outsider growing up	(Ref: No)					9.00e-13					3.63e-10
	Yes	-0.69	(-0.88,-0.50)	0.10	-0.43		-0.62	(-0.82,-0.43)	0.10	-0.39	
Self-rated health growing up	(Ref: Good)					< 2e-16					2.22e-16
	Excellent	0.63	(0.45,0.81)	0.09	0.40		0.61	(0.43,0.79)	0.09	0.38	
	Very good	0.24	(0.05,0.43)	0.10	0.15		0.21	(0.02,0.39)	0.10	0.13	
	Fair	-0.21	(-0.51,0.09)	0.15	-0.13		-0.17	(-0.47,0.12)	0.15	-0.11	
	Poor	-0.27	(-0.74,0.21)	0.24	-0.17		-0.22	(-0.69,0.25)	0.24	-0.14	
Immigration status	(Ref: Born in this country)					0.011					0.028
	Born in another country	0.20	(0.05,0.35)	0.08	0.12		0.17	(0.02,0.33)	0.08	0.11	
Age 12 religious service attendance	(Ref: Never)					0.139					0.061
	At least 1/week	0.13	(-0.03,0.30)	0.08	0.08		0.16	(-0.00,0.33)	0.09	0.10	
	1-3/month	-0.05	(-0.25,0.15)	0.10	-0.03		-0.05	(-0.25,0.16)	0.10	-0.03	
	< 1/month	-0.01	(-0.20,0.19)	0.10	-0.00		-0.00	(-0.19,0.19)	0.10	-0.00	
Year of birth	(Ref: 1998-2005; current age: 18-24)					< 2e-16					< 2e-16
	1993-1998; age 25-29	0.19	(-0.14,0.52)	0.17	0.12		0.21	(-0.13,0.55)	0.17	0.13	
	1983-1993; age 30-39	0.37	(0.09,0.65)	0.14	0.23		0.31	(0.02,0.60)	0.15	0.19	
	1973-1983; age 40-49	0.45	(0.18,0.72)	0.14	0.28		0.49	(0.21,0.77)	0.14	0.31	
	1963-1973; age 50-59	0.43	(0.16,0.70)	0.14	0.27		0.39	(0.11,0.67)	0.14	0.24	

Variable	Category	Secure Flourishing Index Est	95% CI	SE	Est/SD	Global p-value	Flourishing Index Est	95% CI	SE	Est/SD	Global p-value
	1953-1963; age 60-69	0.89	(0.62,1.15)	0.14	0.56		0.83	(0.55,1.10)	0.14	0.51	
	1943-1953; age 70-79	1.29	(1.01,1.57)	0.14	0.81		1.19	(0.90,1.48)	0.15	0.74	
	1943 or earlier; age 80+	1.49	(1.21,1.78)	0.14	0.94		1.40	(1.11,1.69)	0.15	0.87	
Gender	(Ref: Male)					0.276					0.213
	Female	0.09	(-0.03,0.21)	0.06	0.06		0.10	(-0.02,0.23)	0.06	0.06	
	Other	-0.12	(-0.72,0.49)	0.31	-0.07		-0.08	(-0.64,0.48)	0.29	-0.05	
Religious affiliation	(Ref: No religion/Atheist/Agnostic)					0.559					0.382
	Christianity	0.09	(-0.08,0.26)	0.09	0.06		0.12	(-0.05,0.29)	0.09	0.07	
	Collapsed affiliations with prevalence<3%	0.03	(-0.35,0.41)	0.20	0.02		0.06	(-0.33,0.46)	0.20	0.04	
Race/ethnicity	(Ref: Plurality group)					0.791					0.470
	Non-plurality groups	-0.02	(-0.15,0.11)	0.07	-0.01		-0.05	(-0.18,0.08)	0.07	-0.03	

Note. N=3844; $p < .004$ (Bonferroni corrected threshold); Est., estimated effect of childhood predictor on flourishing score; CI, confidence interval; SE, standard error of the estimated effect; Est/SD, a more standardized measure of effect size--estimated effect of flourishing divided by standard deviation of flourishing--leads to the interpretation, for those with the given status (e.g., those with a good/very good relationship with mother compared to those with bad/very bad) are 0.XX standard deviations higher/lower on flourishing; the Global p-value corresponds to the two-sided joint parameter Wald-type test of whether any of the levels' parameters are non-zero, for history of abuse, outsider, relationship wtih mother/father, this is test of whether the estimated effect is non-zero, for multiple-category predictors (age, health, financial status), this is a joint test of whether any of these effects are non-zero. Note the confidence interval of the effect estimate can contradict the reported global p-value (e.g., for the single-category effects of relationship with mother). In such cases, the reported confidence interval is more robust with corrected degrees of freedom from the pooling across multiple imputations, whereas the global p-value is based on a Wald-type test and is less robust to uncertainty attributable to multiple imputation.

Table S2d. Sensitivity to unmeasured confounding of childhood predictors in Australia (N=3844)

Variable	Category	Secure Flourishing Index		Flourishing Index	
		E-value for Estimate	E-value for 95% CI	E-value for Estimate	E-value for 95% CI
Relationship with mother	(Ref: Very bad/somewhat bad)				
	Very good/somewhat good	1.18	1.00	1.05	1.00
Relationship with father	(Ref: Very bad/somewhat bad)				
	Very good/somewhat good	1.31	1.00	1.43	1.00
Parent marital status	(Ref: Parents married)				
	Divorced	1.14	1.00	1.10	1.00
	Parents were never married	1.60	1.00	1.82	1.17
	One or both parents had died	1.80	1.20	1.95	1.37
Subjective financial status of family growing up	(Ref: Got by)				
	Lived comfortably	1.32	1.00	1.26	1.00
	Found it difficult	1.17	1.00	1.11	1.00
	Found it very difficult	1.77	1.09	1.69	1.00
Abuse	(Ref: No)				
	Yes	1.94	1.67	1.88	1.61
Outsider growing up	(Ref: No)				
	Yes	2.32	1.99	2.21	1.87
Self-rated health growing up	(Ref: Good)				
	Excellent	2.21	1.90	2.19	1.88
	Very good	1.55	1.20	1.50	1.12
	Fair	1.51	1.00	1.44	1.00
	Poor	1.60	1.00	1.53	1.00
Immigration status	(Ref: Born in this country)				
	Born in another country	1.48	1.19	1.44	1.11
Age 12 religious service attendance	(Ref: Never)				
	At least 1/week	1.37	1.00	1.43	1.00
	1-3/month	1.20	1.00	1.19	1.00
	< 1/month	1.06	1.00	1.03	1.00
Year of birth	(Ref: 1998-2005; current age: 18-24)				
	1993-1998; age 25-29	1.47	1.00	1.50	1.00
	1983-1993; age 30-39	1.77	1.29	1.68	1.14

Variable	Category	Secure Flourishing Index		Flourishing Index	
		E-value for Estimate	E-value for 95% CI	E-value for Estimate	E-value for 95% CI
	1973-1983; age 40-49	1.90	1.45	1.98	1.52
	1963-1973; age 50-59	1.87	1.42	1.81	1.34
	1953-1963; age 60-69	2.69	2.20	2.59	2.08
	1943-1953; age 70-79	3.57	2.94	3.36	2.73
	1943 or earlier; age 80+	4.09	3.39	3.88	3.18
Gender	(Ref: Male)				
	Female	1.29	1.00	1.32	1.00
	Other	1.34	1.00	1.27	1.00
Religious affiliation	(Ref: No religion/Atheist/Agnostic)				
	Christianity	1.29	1.00	1.35	1.00
	Collapsed affiliations with prevalence<3%	1.16	1.00	1.23	1.00
Race/ethnicity	(Ref: Plurality group)				
	Non-plurality groups	1.11	1.00	1.20	1.00

Table S3a. Nationally representative descriptive statistics for Brazil

Characteristic	N = 13,204[1]
**Age group**	
1998-2005; age 18-24	1,986 (15%)
1993-1998; age 25-29	1,468 (11%)
1983-1993; age 30-39	2,908 (22%)
1973-1983; age 40-49	2,638 (20%)
1963-1973; age 50-59	2,131 (16%)
1953-1963; age 60-69	1,435 (11%)
1943-1953; age 70-79	510 (3.9%)
1943 or earlier; age 80+	126 (1.0%)
(Missing)	0 (0%)
**Gender**	
Male	6,320 (48%)
Female	6,820 (52%)
Other	35 (0.3%)
(Missing)	30 (0.2%)
**Race/Ethnicity**	
Amarela	238 (1.8%)
Branca	5,169 (39%)
Indígena	131 (1.0%)
Other	61 (0.5%)
Parda	5,125 (39%)
Preta	1,615 (12%)
(Missing)	865 (6.6%)
**Marital status**	
Married	4,646 (35%)
Separated	594 (4.5%)
Divorced	865 (6.5%)
Widowed	408 (3.1%)
Single, never married	4,347 (33%)
Domestic Partner	2,081 (16%)
(Missing)	263 (2.0%)
**Employment**	
Employed for an employer	3,756 (28%)
Self-employed	2,918 (22%)
Retired	1,536 (12%)
Student	624 (4.7%)
Homemaker	1,305 (9.9%)
Unemployed and looking for a job	2,419 (18%)
None of these/Other	448 (3.4%)
(Missing)	199 (1.5%)
**Religious service attendance**	
More than 1/week	2,386 (18%)
1/week	2,272 (17%)
1-3/month	1,398 (11%)
A few times a year	3,978 (30%)
Never	3,110 (24%)
(Missing)	61 (0.5%)
**Education**	
Up to 8 years	3,139 (24%)
9-15 years	7,665 (58%)
16+ years	2,390 (18%)
(Missing)	10 (<0.1%)
**Immigration status**	
Born in this country	12,688 (96%)
Born in another country	153 (1.2%)
(Missing)	363 (2.7%)
**Religious affiliation as an adult (now)**	
Christianity	9,911 (75%)

Characteristic	N = 13,204[1]
Islam	6 (<0.1%)
Hinduism	1 (<0.1%)
Buddhism	37 (0.3%)
Judaism	31 (0.2%)
Sikhism	0 (0%)
Baha'i	2 (<0.1%)
Jainism	2 (<0.1%)
Shinto	1 (<0.1%)
Taoism	1 (<0.1%)
Confucianism	6 (<0.1%)
Primal, Animist, or Folk religion	15 (0.1%)
Spiritism	696 (5.3%)
Umbanda, Candomble, and other African-derived religions	525 (4.0%)
Chinese folk/traditional religion	0 (0%)
Some other religion	144 (1.1%)
No religion/Atheist/Agnostic	1,712 (13%)
(Missing)	113 (0.9%)
**Parent marital status**	
Parents married	8,546 (65%)
Divorced	1,384 (10%)
Parents were never married	1,985 (15%)
One or both parents had died	508 (3.8%)
(Missing)	781 (5.9%)
**Age 12 religious service attendance**	
At least 1/week	6,306 (48%)
1-3/month	2,491 (19%)
<1/month	2,629 (20%)
Never	1,707 (13%)
(Missing)	71 (0.5%)
**Relationship with mother**	
Very good	8,369 (63%)
Somewhat good	3,559 (27%)
Somewhat bad	483 (3.7%)
Very bad	214 (1.6%)
Does not apply	507 (3.8%)
(Missing)	73 (0.6%)
**Relationship with father**	
Very good	6,364 (48%)
Somewhat good	3,654 (28%)
Somewhat bad	1,035 (7.8%)
Very bad	756 (5.7%)
Does not apply	1,303 (9.9%)
(Missing)	93 (0.7%)
**Outsider growing up**	
Yes	1,659 (13%)
No	11,234 (85%)
(Missing)	311 (2.4%)
**Self-reported history of abuse**	
Yes	2,606 (20%)
No	10,147 (77%)
(Missing)	451 (3.4%)
**Self-rated health growing up**	
Excellent	5,312 (40%)
Very good	3,392 (26%)
Good	2,873 (22%)
Fair	1,368 (10%)
Poor	228 (1.7%)
(Missing)	30 (0.2%)
**Subjective financial status of family growing up**	

Characteristic	N = 13,204[1]
Lived comfortably	4,998 (38%)
Got by	4,616 (35%)
Found it difficult	2,484 (19%)
Found it very difficult	1,027 (7.8%)
(Missing)	79 (0.6%)
**Religious affiliation at age 12**	
Christianity	11,403 (86%)
Islam	15 (0.1%)
Hinduism	1 (<0.1%)
Buddhism	27 (0.2%)
Judaism	40 (0.3%)
Sikhism	0 (0%)
Baha'i	1 (<0.1%)
Jainism	4 (<0.1%)
Shinto	4 (<0.1%)
Taoism	1 (<0.1%)
Confucianism	7 (<0.1%)
Primal, Animist, or Folk religion	17 (0.1%)
Spiritism	336 (2.5%)
Umbanda, Candomble, and other African-derived religions	262 (2.0%)
Chinese folk/traditional religion	0 (0%)
Some other religion	87 (0.7%)
No religion/Atheist/Agnostic	908 (6.9%)
(Missing)	94 (0.7%)

[1] n (%)

Table S3b. Means by demographic category for Brazil (N=13204)

Variable	Category	Secure Flourishing Index				Flourishing Index			
		Mean	95% CI	SE	Global p-value	Mean	95% CI	SE	Global p-value
Age group	18-24	7.14	(7.05,7.24)	0.05	< 2e-16	6.63	(6.54,6.72)	0.05	< 2e-16
	25-29	7.33	(7.21,7.45)	0.06		6.71	(6.60,6.82)	0.06	
	30-39	7.57	(7.50,7.65)	0.04		6.93	(6.86,7.00)	0.04	
	40-49	7.67	(7.58,7.75)	0.04		7.04	(6.96,7.12)	0.04	
	50-59	7.83	(7.74,7.93)	0.05		7.20	(7.10,7.29)	0.05	
	60-69	8.05	(7.93,8.18)	0.07		7.42	(7.30,7.55)	0.06	
	70-79	8.36	(8.14,8.58)	0.11		7.78	(7.55,8.00)	0.11	
	80 or older	8.32	(7.99,8.65)	0.17		7.91	(7.50,8.31)	0.20	
Gender	Female	7.50	(7.44,7.55)	0.03	1.03e-11	6.83	(6.78,6.89)	0.03	< 2e-16
	Male	7.78	(7.72,7.84)	0.03		7.22	(7.17,7.28)	0.03	
	Other	7.21	(6.29,8.14)	0.45		6.77	(5.86,7.67)	0.44	
Marital status	Divorced	7.51	(7.36,7.67)	0.08	< 2e-16	6.94	(6.79,7.10)	0.08	< 2e-16
	Domestic partner	7.55	(7.45,7.64)	0.05		6.87	(6.78,6.96)	0.05	
	Married	8.09	(8.03,8.14)	0.03		7.44	(7.38,7.49)	0.03	
	Separated	7.21	(7.00,7.41)	0.10		6.60	(6.41,6.78)	0.10	
	Single/Never been married	7.23	(7.16,7.30)	0.04		6.68	(6.61,6.75)	0.03	
	Widowed	8.00	(7.76,8.24)	0.12		7.45	(7.20,7.69)	0.12	
Employment	Employed for an employer	7.75	(7.69,7.81)	0.03	< 2e-16	7.14	(7.08,7.20)	0.03	< 2e-16
	Homemaker	7.57	(7.43,7.70)	0.07		6.88	(6.75,7.00)	0.06	
	None of these/Other	7.39	(7.14,7.63)	0.12		6.81	(6.59,7.04)	0.11	
	Retired	8.15	(8.03,8.27)	0.06		7.58	(7.46,7.70)	0.06	
	Self-employed	7.78	(7.71,7.85)	0.04		7.16	(7.09,7.23)	0.04	
	Student	7.31	(7.15,7.48)	0.08		6.87	(6.72,7.02)	0.08	
	Unemployed and looking for a job	7.10	(7.00,7.20)	0.05		6.47	(6.37,6.57)	0.05	
Religious service attendance	A few times a year	7.59	(7.53,7.66)	0.03	< 2e-16	6.97	(6.91,7.03)	0.03	< 2e-16
	More than once a week	8.23	(8.15,8.32)	0.04		7.59	(7.51,7.67)	0.04	

Variable	Category	Secure Flourishing Index				Flourishing Index			
		Mean	95% CI	SE	Global p-value	Mean	95% CI	SE	Global p-value
	Never	7.05	(6.96,7.15)	0.05		6.49	(6.40,6.58)	0.05	
	Once a week	7.85	(7.77,7.94)	0.04		7.25	(7.16,7.33)	0.04	
	One to three times a month	7.64	(7.53,7.75)	0.06		7.02	(6.92,7.13)	0.05	
Education	Up to 8	7.78	(7.69,7.87)	0.05	6.32e-08	7.17	(7.08,7.25)	0.04	8.79e-10
	9 to 15	7.75	(7.68,7.83)	0.04		7.16	(7.09,7.24)	0.04	
	16+	7.54	(7.48,7.59)	0.03		6.92	(6.87,6.97)	0.03	
Immigration status	Born in another country	7.73	(7.36,8.11)	0.19	0.556	7.24	(6.86,7.62)	0.19	0.188
	Born in this country	7.63	(7.59,7.67)	0.02		7.02	(6.98,7.06)	0.02	
Religious affiliation	Baha'i	9.60	*	*	< 2e-16	9.55	*	*	< 2e-16
	Buddhism	7.68	(6.88,8.47)	0.38		7.29	(6.37,8.22)	0.44	
	Christianity	7.76	(7.72,7.80)	0.02		7.13	(7.09,7.17)	0.02	
	Hinduism	5.88	*	*		6.07	*	*	
	Islam	6.97	*	*		6.88	*	*	
	Judaism	6.59	(5.11,8.07)	0.68		6.30	(4.71,7.89)	0.74	
	No religion/Atheist/ Agnostic	7.00	(6.88,7.11)	0.06		6.47	(6.36,6.58)	0.06	
	Primal, Animist, or Folk religion	8.08	*	*		7.51	*	*	
	Shinto	5.70	*	*		5.52	*	*	
	Some other religion	7.64	(7.23,8.05)	0.21		7.19	(6.83,7.54)	0.18	
	Taoism	7.07	*	*		6.68	*	*	
	Confucianism	8.30	*	*		7.18	*	*	
	Jainism	5.87	*	*		5.92	*	*	
	Spiritism	7.61	(7.44,7.78)	0.09		7.04	(6.87,7.20)	0.08	
	Umbanda, Candomblé, and other African-derived religions	7.36	(7.16,7.57)	0.10		6.65	(6.46,6.85)	0.10	

Note. N=13204; p < .007 = 0.05/7 (Bonferroni corrected p-value significance threshold); Mean, estimated group mean; CI, confidence interval for the mean within group; SE, complex survey adjusted standard error or the mean; Global p-value, two-tailed Wald-type test of whether there is evidence of any differences

Variable	Category	Secure Flourishing Index				Flourishing Index			
		Mean	95% CI	SE	Global p-value	Mean	95% CI	SE	Global p-value

in mean scores among groups of a demographic characteristic. *Estimate is not reported due to multiple-imputation and complex survey adjusted degrees of freedom was less than 1.00 leading to insufficient information to provide an estimate of the uncertainty in the estimate. These groups are removed when estimating the global test of mean differences.

Table S3c. Childhood predictors regression analysis results for Brazil (N=13204)

		Secure Flourishing Index					Flourishing Index				
Variable	Category	Est	95% CI	SE	Est/SD	Global p-value	Est	95% CI	SE	Est/SD	Global p-value
Relationship with mother	(Ref: Very bad/somewhat bad)					0.042					0.011
	Very good/somewhat good	0.16	(0.00,0.32)	0.08	0.10		0.22	(0.05,0.39)	0.09	0.13	
Relationship with father	(Ref: Very bad/somewhat bad)					9.51e-14					9.88e-15
	Very good/somewhat good	0.38	(0.28,0.49)	0.05	0.22		0.41	(0.31,0.52)	0.05	0.25	
Parent marital status	(Ref: Parents married)					0.275					0.599
	Divorced	-0.07	(-0.19,0.05)	0.06	-0.04		-0.06	(-0.18,0.06)	0.06	-0.04	
	Parents were never married	-0.09	(-0.21,0.02)	0.06	-0.05		-0.05	(-0.16,0.07)	0.06	-0.03	
	One or both parents had died	-0.06	(-0.28,0.16)	0.11	-0.03		-0.08	(-0.31,0.15)	0.12	-0.05	
Subjective financial status of family growing up	(Ref: Got by)					9.76e-10					2.97e-05
	Lived comfortably	0.17	(0.08,0.25)	0.04	0.10		0.15	(0.07,0.24)	0.04	0.09	
	Found it difficult	-0.14	(-0.25,-0.04)	0.05	-0.08		-0.06	(-0.17,0.05)	0.06	-0.04	
	Found it very difficult	-0.14	(-0.29,0.01)	0.08	-0.08		-0.05	(-0.21,0.11)	0.08	-0.03	
Abuse	(Ref: No)					8.55e-15					1.32e-14
	Yes	-0.39	(-0.49,-0.29)	0.05	-0.22		-0.40	(-0.50,-0.29)	0.05	-0.24	

Variable	Category	Secure Flourishing Index					Flourishing Index				
		Est	95% CI	SE	Est/SD	Global p-value	Est	95% CI	SE	Est/SD	Global p-value
Outsider growing up	(Ref: No)					2.18e-14					9.77e-15
	Yes	-0.44	(-0.55,-0.32)	0.06	-0.25		-0.47	(-0.59,-0.35)	0.06	-0.28	
Self-rated health growing up	(Ref: Good)					< 2e-16					< 2e-16
	Excellent	0.44	(0.35,0.53)	0.05	0.26		0.51	(0.41,0.60)	0.05	0.30	
	Very good	0.13	(0.03,0.23)	0.05	0.08		0.13	(0.02,0.23)	0.05	0.08	
	Fair	-0.29	(-0.44,-0.14)	0.08	-0.17		-0.37	(-0.52,-0.21)	0.08	-0.22	
	Poor	-0.15	(-0.46,0.16)	0.16	-0.09		-0.17	(-0.51,0.17)	0.17	-0.10	
Immigration status	(Ref: Born in this country)					0.463					0.773
	Born in another country	0.10	(-0.27,0.47)	0.19	0.06		0.01	(-0.35,0.38)	0.19	0.01	
Age 12 religious service attendance	(Ref: Never)					0.004					2.75e-06
	At least 1/week	0.23	(0.10,0.36)	0.07	0.14		0.33	(0.20,0.47)	0.07	0.20	
	1-3/month	0.21	(0.06,0.35)	0.07	0.12		0.23	(0.08,0.38)	0.08	0.14	
	< 1/month	0.16	(0.02,0.30)	0.07	0.09		0.18	(0.03,0.33)	0.08	0.11	
Year of birth	(Ref: 1998-2005; current age: 18-24)					< 2e-16					< 2e-16
	1993-1998; age 25-29	0.02	(-0.12,0.16)	0.07	0.01		0.12	(-0.03,0.26)	0.07	0.07	
	1983-1993; age 30-39	0.22	(0.11,0.33)	0.06	0.13		0.34	(0.22,0.45)	0.06	0.20	
	1973-1983; age 40-49	0.30	(0.18,0.41)	0.06	0.17		0.41	(0.29,0.53)	0.06	0.25	
	1963-1973; age 50-59	0.42	(0.29,0.55)	0.07	0.24		0.54	(0.40,0.67)	0.07	0.32	

Variable	Category	Secure Flourishing Index					Flourishing Index				
		Est	95% CI	SE	Est/SD	Global p-value	Est	95% CI	SE	Est/SD	Global p-value
	1953-1963; age 60-69	0.62	(0.46,0.77)	0.08	0.36		0.74	(0.58,0.90)	0.08	0.44	
	1943-1953; age 70-79	0.99	(0.76,1.23)	0.12	0.58		1.07	(0.84,1.30)	0.12	0.64	
	1943 or earlier; age 80+	1.15	(0.70,1.60)	0.23	0.67		1.07	(0.68,1.47)	0.20	0.64	
Gender	(Ref: Male)					7.98e-05					0.454
	Female	-0.16	(-0.23,-0.09)	0.04	-0.09		-0.04	(-0.12,0.03)	0.04	-0.03	
	Other	-0.13	(-0.95,0.69)	0.42	-0.07		-0.22	(-1.13,0.69)	0.46	-0.13	
Religious affiliation	(Ref: No religion/Atheist/Agnostic)					0.703					0.904
	Christianity	-0.01	(-0.16,0.14)	0.08	-0.00		0.03	(-0.12,0.19)	0.08	0.02	
	Collapsed affiliations with prevalence<3%	0.06	(-0.15,0.26)	0.10	0.03		0.04	(-0.17,0.24)	0.11	0.02	
Race/ethnicity	(Ref: Plurality group)					1.03e-04					3.86e-07
	Non-plurality groups	0.15	(0.07,0.22)	0.04	0.08		0.19	(0.12,0.27)	0.04	0.12	

Note. N=13204; $p < .004$ (Bonferroni corrected threshold); Est., estimated effect of childhood predictor on flourishing score; CI, confidence interval; SE, standard error of the estimated effect; Est/SD, a more standardized measure of effect size--estimated effect of flourishing divided by standard deviation of flourishing--leads to the interpretation, for those with the given status (e.g., those with a good/very good relationship with mother compared to those with bad/very bad) are 0.XX standard deviations higher/lower on flourishing; the Global p-value corresponds to the two-sided joint parameter Wald-type test of whether any of the levels' parameters are non-zero, for history of abuse, outsider, relationship wtih mother/father, this is test of whether the estimated effect is non-zero, for multiple-category predictors (age, health, financial status), this is a joint test of whether any of these effects are non-zero. Note the confidence interval of the effect estimate can contradict the reported global p-value (e.g., for the single-category effects of relationship with mother). In such cases, the reported confidence interval is more robust with corrected degrees of freedom from the pooling across multiple imputations, whereas the global p-value is based on a Wald-type test and is less robust to uncertainty attributable to multiple imputation.

Table S3d. Sensitivity to unmeasured confounding of childhood predictors in Brazil (N=13204)					
		Secure Flourishing Index		Flourishing Index	
Variable	Category	E-value for Estimate	E-value for 95% CI	E-value for Estimate	E-value for 95% CI
Relationship with mother	(Ref: Very bad/somewhat bad)				
	Very good/somewhat good	1.41	1.05	1.49	1.19
Relationship with father	(Ref: Very bad/somewhat bad)				
	Very good/somewhat good	1.77	1.60	1.80	1.63
Parent marital status	(Ref: Parents married)				
	Divorced	1.24	1.00	1.21	1.00
	Parents were never married	1.29	1.00	1.19	1.00
	One or both parents had died	1.22	1.00	1.25	1.00
Subjective financial status of family growing up	(Ref: Got by)				
	Lived comfortably	1.42	1.27	1.39	1.24
	Found it difficult	1.38	1.16	1.21	1.00
	Found it very difficult	1.37	1.00	1.19	1.00
Abuse	(Ref: No)				
	Yes	1.77	1.61	1.77	1.61
Outsider growing up	(Ref: No)				
	Yes	1.85	1.67	1.88	1.69
Self-rated health growing up	(Ref: Good)				
	Excellent	1.86	1.71	1.94	1.80
	Very good	1.35	1.15	1.34	1.13
	Fair	1.63	1.38	1.72	1.48
	Poor	1.40	1.00	1.42	1.00
Immigration status	(Ref: Born in this country)				
	Born in another country	1.30	1.00	1.08	1.00
Age 12 religious service attendance	(Ref: Never)				
	At least 1/week	1.53	1.31	1.67	1.46
	1-3/month	1.48	1.22	1.51	1.26
	< 1/month	1.40	1.10	1.44	1.16
Year of birth	(Ref: 1998-2005; current age: 18-24)				
	1993-1998; age 25-29	1.12	1.00	1.33	1.00
	1983-1993; age 30-39	1.51	1.32	1.68	1.50

Variable	Category	Secure Flourishing Index		Flourishing Index	
		E-value for Estimate	E-value for 95% CI	E-value for Estimate	E-value for 95% CI
	1973-1983; age 40-49	1.63	1.44	1.79	1.60
	1963-1973; age 50-59	1.83	1.62	1.99	1.78
	1953-1963; age 60-69	2.15	1.89	2.32	2.05
	1943-1953; age 70-79	2.82	2.39	2.92	2.49
	1943 or earlier; age 80+	3.14	2.29	2.92	2.21
Gender	(Ref: Male)				
	Female	1.41	1.28	1.18	1.00
	Other	1.35	1.00	1.49	1.00
Religious affiliation	(Ref: No religion/Atheist/Agnostic)				
	Christianity	1.06	1.00	1.16	1.00
	Collapsed affiliations with prevalence<3%	1.21	1.00	1.16	1.00
Race/ethnicity	(Ref: Plurality group)				
	Non-plurality groups	1.38	1.24	1.45	1.33

Table S4a. Nationally representative descriptive statistics for Egypt

Characteristic	N = 4,729[1]
**Age group**	
1998-2005; age 18-24	960 (20%)
1993-1998; age 25-29	607 (13%)
1983-1993; age 30-39	1,204 (25%)
1973-1983; age 40-49	897 (19%)
1963-1973; age 50-59	613 (13%)
1953-1963; age 60-69	387 (8.2%)
1943-1953; age 70-79	54 (1.1%)
1943 or earlier; age 80+	7 (0.2%)
(Missing)	0 (0%)
**Gender**	
Male	2,394 (51%)
Female	2,334 (49%)
Other	0 (0%)
(Missing)	0 (<0.1%)
**Race/Ethnicity**	
Arab	4,585 (97%)
Bedouin Arab	4 (<0.1%)
Greek	1 (<0.1%)
Nubian	27 (0.6%)
Turkish	9 (0.2%)
(Missing)	102 (2.2%)
**Marital status**	
Married	3,387 (72%)
Separated	39 (0.8%)
Divorced	101 (2.1%)
Widowed	238 (5.0%)
Single, never married	947 (20%)
Domestic Partner	0 (0%)
(Missing)	17 (0.4%)
**Employment**	
Employed for an employer	1,267 (27%)
Self-employed	892 (19%)
Retired	253 (5.4%)
Student	297 (6.3%)
Homemaker	1,772 (37%)
Unemployed and looking for a job	224 (4.7%)
None of these/Other	21 (0.4%)
(Missing)	3 (<0.1%)
**Religious service attendance**	
More than 1/week	839 (18%)
1/week	960 (20%)
1-3/month	368 (7.8%)
A few times a year	458 (9.7%)
Never	2,091 (44%)
(Missing)	12 (0.3%)
**Education**	
Up to 8 years	2,486 (53%)
9-15 years	1,599 (34%)
16+ years	643 (14%)
(Missing)	1 (<0.1%)
**Immigration status**	
Born in this country	4,713 (100%)
Born in another country	16 (0.3%)
(Missing)	1 (<0.1%)
**Religious affiliation as an adult (now)**	
Christianity	120 (2.5%)
Islam	4,607 (97%)

**Characteristic**	**N = 4,729**[1]
Hinduism	0 (0%)
Buddhism	0 (0%)
Judaism	0 (0%)
Sikhism	0 (0%)
Baha'i	0 (0%)
Jainism	0 (0%)
Shinto	0 (0%)
Taoism	0 (<0.1%)
Confucianism	0 (0%)
Primal, Animist, or Folk religion	0 (0%)
Spiritism	0 (0%)
Umbanda, Candomble, and other African-derived religions	0 (0%)
Chinese folk/traditional religion	0 (0%)
Some other religion	0 (0%)
No religion/Atheist/Agnostic	0 (0%)
(Missing)	1 (<0.1%)
**Parent marital status**	
Parents married	4,049 (86%)
Divorced	131 (2.8%)
Parents were never married	9 (0.2%)
One or both parents had died	485 (10%)
(Missing)	55 (1.2%)
**Age 12 religious service attendance**	
At least 1/week	2,307 (49%)
1-3/month	570 (12%)
<1/month	629 (13%)
Never	1,165 (25%)
(Missing)	57 (1.2%)
**Relationship with mother**	
Very good	4,110 (87%)
Somewhat good	505 (11%)
Somewhat bad	21 (0.4%)
Very bad	10 (0.2%)
Does not apply	83 (1.8%)
(Missing)	0 (0%)
**Relationship with father**	
Very good	3,713 (79%)
Somewhat good	683 (14%)
Somewhat bad	56 (1.2%)
Very bad	30 (0.6%)
Does not apply	233 (4.9%)
(Missing)	14 (0.3%)
**Outsider growing up**	
Yes	260 (5.5%)
No	4,456 (94%)
(Missing)	13 (0.3%)
**Self-reported history of abuse**	
Yes	405 (8.6%)
No	4,293 (91%)
(Missing)	30 (0.6%)
**Self-rated health growing up**	
Excellent	2,687 (57%)
Very good	1,174 (25%)
Good	497 (11%)
Fair	265 (5.6%)
Poor	106 (2.2%)
(Missing)	1 (<0.1%)
**Subjective financial status of family growing up**	
Lived comfortably	1,251 (26%)

Characteristic	N = 4,729[1]
Got by	2,352 (50%)
Found it difficult	857 (18%)
Found it very difficult	268 (5.7%)
(Missing)	1 (<0.1%)
**Religious affiliation at age 12**	
Christianity	123 (2.6%)
Islam	4,602 (97%)
Hinduism	0 (0%)
Buddhism	0 (0%)
Judaism	0 (0%)
Sikhism	0 (0%)
Baha'i	0 (0%)
Jainism	1 (<0.1%)
Shinto	0 (0%)
Taoism	0 (<0.1%)
Confucianism	0 (0%)
Primal, Animist, or Folk religion	0 (0%)
Spiritism	0 (0%)
Umbanda, Candomble, and other African-derived religions	0 (0%)
Chinese folk/traditional religion	0 (0%)
Some other religion	0 (0%)
No religion/Atheist/Agnostic	0 (0%)
(Missing)	3 (<0.1%)

[1] n (%)

Table S4b. Means by demographic category for Egypt (N=4729)

Variable	Category	Secure Flourishing Index				Flourishing Index			
		Mean	95% CI	SE	Global p-value	Mean	95% CI	SE	Global p-value
Age group	18-24	7.81	(7.69,7.92)	0.06	0.026	7.51	(7.39,7.63)	0.06	0.001
	25-29	7.64	(7.51,7.76)	0.07		7.27	(7.14,7.40)	0.07	
	30-39	7.54	(7.43,7.64)	0.05		7.15	(7.04,7.26)	0.05	
	40-49	7.63	(7.53,7.73)	0.05		7.33	(7.22,7.44)	0.06	
	50-59	7.58	(7.44,7.71)	0.07		7.34	(7.20,7.47)	0.07	
	60-69	7.63	(7.43,7.82)	0.10		7.40	(7.20,7.60)	0.10	
	70-79	7.09	(6.34,7.84)	0.37		6.91	(6.14,7.68)	0.38	
	80 or older	7.85	*	*		7.69	*	*	
Gender	Female	7.74	(7.67,7.80)	0.03	5.57e-05	7.38	(7.30,7.45)	0.04	0.019
	Male	7.53	(7.44,7.61)	0.05		7.26	(7.17,7.35)	0.04	
Marital status	Divorced	7.22	(6.84,7.61)	0.19	0.170	6.90	(6.45,7.35)	0.23	0.035
	Married	7.63	(7.56,7.71)	0.04		7.30	(7.23,7.38)	0.04	
	Separated	7.33	(6.83,7.84)	0.25		6.98	(6.42,7.53)	0.27	
	Single/Never been married	7.68	(7.57,7.78)	0.05		7.43	(7.33,7.54)	0.05	
	Widowed	7.62	(7.42,7.82)	0.10		7.24	(7.03,7.45)	0.11	
Employment	Employed for an employer	7.59	(7.47,7.71)	0.06	0.005	7.32	(7.20,7.44)	0.06	0.010
	Homemaker	7.73	(7.65,7.81)	0.04		7.33	(7.25,7.42)	0.04	
	None of these/Other	8.16	(7.53,8.78)	0.29		7.95	(7.29,8.61)	0.30	
	Retired	7.34	(7.06,7.63)	0.14		7.22	(6.92,7.51)	0.15	
	Self-employed	7.57	(7.44,7.70)	0.07		7.27	(7.14,7.41)	0.07	
	Student	7.78	(7.61,7.96)	0.09		7.58	(7.40,7.75)	0.09	
	Unemployed and looking for a job	7.42	(7.15,7.69)	0.14		7.07	(6.80,7.33)	0.13	
Religious service attendance	A few times a year	7.64	(7.47,7.80)	0.08	0.448	7.34	(7.17,7.51)	0.08	0.266
	More than once a week	7.71	(7.57,7.85)	0.07		7.43	(7.29,7.56)	0.07	
	Never	7.63	(7.55,7.71)	0.04		7.28	(7.19,7.36)	0.04	
	Once a week	7.63	(7.51,7.74)	0.06		7.34	(7.21,7.48)	0.07	

Variable	Category	Secure Flourishing Index Mean	95% CI	SE	Global p-value	Flourishing Index Mean	95% CI	SE	Global p-value
	One to three times a month	7.45	(7.23,7.66)	0.11		7.17	(6.96,7.38)	0.11	
Education	Up to 8	7.48	(7.39,7.58)	0.05	6.81e-09	7.15	(7.05,7.25)	0.05	1.00e-09
	9 to 15	7.95	(7.84,8.07)	0.06		7.72	(7.59,7.84)	0.07	
	16+	7.73	(7.67,7.79)	0.03		7.42	(7.36,7.48)	0.03	
Immigration status	Born in another country	7.54	(6.75,8.33)	0.36	0.800	7.15	(6.40,7.90)	0.34	0.635
	Born in this country	7.63	(7.57,7.69)	0.03		7.32	(7.25,7.38)	0.03	
Religious affiliation	Christianity	7.32	(6.89,7.75)	0.21	< 2e-16	7.02	(6.64,7.40)	0.19	< 2e-16
	Islam	7.64	(7.58,7.70)	0.03		7.32	(7.26,7.39)	0.03	
	Taoism	3.70	*	*		4.42	*	*	

Note. N=4729; p < .007 = 0.05/7 (Bonferroni corrected p-value significance threshold); Mean, estimated group mean; CI, confidence interval for the mean within group; SE, complex survey adjusted standard error or the mean; Global p-value, two-tailed Wald-type test of whether there is evidence of any differences in mean scores among groups of a demographic characteristic. *Estimate is not reported due to multiple-imputation and complex survey adjusted degrees of freedom was less than 1.00 leading to insufficient information to provide an estimate of the uncertainty in the estimate. These groups are removed when estimating the global test of mean differences.

Table S4c. Childhood predictors regression analysis results for Egypt (N=4729)

		Secure Flourishing Index					Flourishing Index				
Variable	Category	Est	95% CI	SE	Est/SD	Global p-value	Est	95% CI	SE	Est/SD	Global p-value
Relationship with mother	(Ref: Very bad/somewhat bad)					0.763					0.428
	Very good/somewhat good	0.09	(-0.59,0.76)	0.34	0.06		0.25	(-0.40,0.90)	0.33	0.17	
Relationship with father	(Ref: Very bad/somewhat bad)					0.885					0.838
	Very good/somewhat good	0.01	(-0.30,0.33)	0.16	0.01		-0.02	(-0.30,0.26)	0.14	-0.01	
Parent marital status	(Ref: Parents married)					0.453					0.928
	Divorced	-0.21	(-0.56,0.13)	0.18	-0.15		-0.02	(-0.35,0.31)	0.17	-0.01	
	Parents were never married	-0.34	(-1.08,0.39)	0.37	-0.23		-0.17	(-0.79,0.45)	0.32	-0.12	
	One or both parents had died	-0.06	(-0.24,0.13)	0.09	-0.04		-0.01	(-0.18,0.16)	0.09	-0.01	
Subjective financial status of family growing up	(Ref: Got by)					2.61e-04					0.010
	Lived comfortably	0.19	(0.07,0.31)	0.06	0.13		0.15	(0.04,0.27)	0.06	0.10	
	Found it difficult	-0.20	(-0.38,-0.03)	0.09	-0.14		-0.13	(-0.31,0.04)	0.09	-0.09	
	Found it very difficult	-0.02	(-0.23,0.18)	0.11	-0.02		0.03	(-0.16,0.22)	0.10	0.02	
Abuse	(Ref: No)					0.002					0.002
	Yes	-0.32	(-0.51,-0.12)	0.10	-0.22		-0.31	(-0.50,-0.12)	0.10	-0.21	

Variable	Category	Secure Flourishing Index					Flourishing Index				
		Est	95% CI	SE	Est/SD	Global p-value	Est	95% CI	SE	Est/SD	Global p-value
Outsider growing up	(Ref: No)					0.882					0.556
	Yes	-0.01	(-0.21,0.18)	0.10	-0.01		-0.05	(-0.24,0.13)	0.09	-0.04	
Self-rated health growing up	(Ref: Good)					0.016					0.047
	Excellent	0.00	(-0.19,0.20)	0.10	0.00		0.05	(-0.14,0.24)	0.10	0.03	
	Very good	-0.08	(-0.26,0.11)	0.10	-0.05		-0.01	(-0.20,0.17)	0.09	-0.01	
	Fair	-0.37	(-0.64,-0.11)	0.14	-0.26		-0.29	(-0.55,-0.04)	0.13	-0.20	
	Poor	-0.27	(-0.75,0.21)	0.25	-0.18		-0.15	(-0.63,0.32)	0.24	-0.10	
Immigration status	(Ref: Born in this country)					0.617					0.689
	Born in another country	-0.17	(-0.86,0.51)	0.35	-0.12		-0.15	(-0.92,0.62)	0.39	-0.10	
Age 12 religious service attendance	(Ref: Never)					8.26e-05					5.74e-04
	At least 1/week	0.33	(0.19,0.47)	0.07	0.23		0.31	(0.17,0.45)	0.07	0.21	
	1-3/month	0.13	(-0.04,0.30)	0.09	0.09		0.17	(0.01,0.33)	0.08	0.11	
	< 1/month	0.05	(-0.12,0.23)	0.09	0.04		0.11	(-0.05,0.28)	0.08	0.07	
Year of birth	(Ref: 1998-2005; current age: 18-24)					9.19e-04					0.027
	1993-1998; age 25-29	-0.24	(-0.41,-0.07)	0.09	-0.16		-0.17	(-0.34,0.01)	0.09	-0.11	
	1983-1993; age 30-39	-0.34	(-0.48,-0.19)	0.07	-0.23		-0.25	(-0.40,-0.11)	0.07	-0.17	
	1973-1983; age 40-49	-0.15	(-0.30,0.01)	0.08	-0.10		-0.15	(-0.29,0.00)	0.07	-0.10	
	1963-1973; age 50-59	-0.14	(-0.31,0.04)	0.09	-0.09		-0.19	(-0.37,-0.02)	0.09	-0.13	

Variable	Category	Secure Flourishing Index Est	95% CI	SE	Est/SD	Global p-value	Flourishing Index Est	95% CI	SE	Est/SD	Global p-value
	1953-1963; age 60-69	-0.05	(-0.27,0.17)	0.11	-0.03		-0.13	(-0.35,0.09)	0.11	-0.09	
	1943-1953; age 70-79	-0.59	(-1.30,0.13)	0.36	-0.40		-0.72	(-1.44,0.00)	0.37	-0.48	
	1943 or earlier; age 80+	0.16	(-0.94,1.26)	0.56	0.11		0.02	(-1.09,1.14)	0.57	0.02	
Gender	(Ref: Male)					1.12e-04					1.1e-06
	Female	0.24	(0.12,0.35)	0.06	0.16		0.32	(0.20,0.44)	0.06	0.21	
Religious affiliation	(Ref: Islam)					0.035					0.060
	Collapsed affiliations with prevalence<3 %	-0.37	(-0.71,-0.03)	0.17	-0.25		-0.37	(-0.76,0.01)	0.20	-0.25	
Race/ethnicit y	(Ref: Plurality group)					0.265					0.260
	Non-plurality groups	0.30	(-0.23,0.82)	0.27	0.20		0.30	(-0.23,0.82)	0.27	0.20	

Note. N=4729; p < .004 (Bonferroni corrected threshold); Est., estimated effect of childhood predictor on flourishing score; CI, confidence interval; SE, standard error of the estimated effect; Est/SD, a more standardized measure of effect size--estimated effect of flourishing divided by standard deviation of flourishing--leads to the interpretation, for those with the given status (e.g., those with a good/very good relationship with mother compared to those with bad/very bad) are 0.XX standard deviations higher/lower on flourishing; the Global p-value corresponds to the two-sided joint parameter Wald-type test of whether any of the levels' parameters are non-zero, for history of abuse, outsider, relationship wtih mother/father, this is test of whether the estimated effect is non-zero, for multiple-category predictors (age, health, financial status), this is a joint test of whether any of these effects are non-zero. Note the confidence interval of the effect estimate can contradict the reported global p-value (e.g., for the single-category effects of relationship with mother). In such cases, the reported confidence interval is more robust with corrected degrees of freedom from the pooling across multiple imputations, whereas the global p-value is based on a Wald-type test and is less robust to uncertainty attributable to multiple imputation.

Table S4d. Sensitivity to unmeasured confounding of childhood predictors in Egypt (N=4729)

Variable	Category	Secure Flourishing Index		Flourishing Index	
		E-value for Estimate	E-value for 95% CI	E-value for Estimate	E-value for 95% CI
Relationship with mother	(Ref: Very bad/somewhat bad)				
	Very good/somewhat good	1.29	1.00	1.62	1.00
Relationship with father	(Ref: Very bad/somewhat bad)				
	Very good/somewhat good	1.10	1.00	1.12	1.00
Parent marital status	(Ref: Parents married)				
	Divorced	1.53	1.00	1.12	1.00
	Parents were never married	1.76	1.00	1.47	1.00
	One or both parents had died	1.23	1.00	1.08	1.00
Subjective financial status of family growing up	(Ref: Got by)				
	Lived comfortably	1.49	1.26	1.43	1.18
	Found it difficult	1.52	1.15	1.39	1.00
	Found it very difficult	1.13	1.00	1.15	1.00
Abuse	(Ref: No)				
	Yes	1.72	1.36	1.72	1.37
Outsider growing up	(Ref: No)				
	Yes	1.10	1.00	1.22	1.00
Self-rated health growing up	(Ref: Good)				
	Excellent	1.05	1.00	1.21	1.00
	Very good	1.27	1.00	1.11	1.00
	Fair	1.82	1.33	1.69	1.18
	Poor	1.63	1.00	1.43	1.00
Immigration status	(Ref: Born in this country)				
	Born in another country	1.46	1.00	1.43	1.00
Age 12 religious service attendance	(Ref: Never)				
	At least 1/week	1.74	1.49	1.72	1.46
	1-3/month	1.39	1.00	1.46	1.09
	< 1/month	1.22	1.00	1.35	1.00
Year of birth	(Ref: 1998-2005; current age: 18-24)				
	1993-1998; age 25-29	1.58	1.25	1.46	1.00
	1983-1993; age 30-39	1.75	1.50	1.62	1.34

Variable	Category	Secure Flourishing Index		Flourishing Index	
		E-value for Estimate	E-value for 95% CI	E-value for Estimate	E-value for 95% CI
	1973-1983; age 40-49	1.41	1.00	1.42	1.01
	1963-1973; age 50-59	1.39	1.00	1.51	1.12
	1953-1963; age 60-69	1.21	1.00	1.38	1.00
	1943-1953; age 70-79	2.20	1.00	2.50	1.02
	1943 or earlier; age 80+	1.44	1.00	1.14	1.00
Gender	(Ref: Male)				
	Female	1.58	1.37	1.74	1.52
Religious affiliation	(Ref: Islam)				
	Collapsed affiliations with prevalence<3%	1.81	1.16	1.84	1.00
Race/ethnicity	(Ref: Plurality group)				
	Non-plurality groups	1.68	1.00	1.69	1.00

Table S5a. Nationally representative descriptive statistics for Germany

Characteristic	N = 9,506[1]
**Age group**	
1998-2005; age 18-24	829 (8.7%)
1993-1998; age 25-29	774 (8.1%)
1983-1993; age 30-39	1,438 (15%)
1973-1983; age 40-49	1,494 (16%)
1963-1973; age 50-59	1,729 (18%)
1953-1963; age 60-69	1,915 (20%)
1943-1953; age 70-79	1,137 (12%)
1943 or earlier; age 80+	190 (2.0%)
(Missing)	0 (0%)
**Gender**	
Male	4,641 (49%)
Female	4,843 (51%)
Other	11 (0.1%)
(Missing)	11 (0.1%)
**Marital status**	
Married	4,784 (50%)
Separated	219 (2.3%)
Divorced	767 (8.1%)
Widowed	409 (4.3%)
Single, never married	2,627 (28%)
Domestic Partner	619 (6.5%)
(Missing)	81 (0.9%)
**Employment**	
Employed for an employer	4,950 (52%)
Self-employed	712 (7.5%)
Retired	2,480 (26%)
Student	605 (6.4%)
Homemaker	251 (2.6%)
Unemployed and looking for a job	288 (3.0%)
None of these/Other	204 (2.1%)
(Missing)	14 (0.2%)
**Religious service attendance**	
More than 1/week	285 (3.0%)
1/week	424 (4.5%)
1-3/month	550 (5.8%)
A few times a year	2,362 (25%)
Never	5,876 (62%)
(Missing)	9 (<0.1%)
**Education**	
Up to 8 years	235 (2.5%)
9-15 years	6,094 (64%)
16+ years	3,164 (33%)
(Missing)	13 (0.1%)
**Immigration status**	
Born in this country	8,722 (92%)
Born in another country	744 (7.8%)
(Missing)	40 (0.4%)
**Religious affiliation as an adult (now)**	
Christianity	5,052 (53%)
Islam	351 (3.7%)
Hinduism	12 (0.1%)
Buddhism	51 (0.5%)
Judaism	19 (0.2%)
Sikhism	5 (<0.1%)
Baha'i	3 (<0.1%)
Jainism	0 (0%)
Shinto	2 (<0.1%)

**Characteristic**	**N = 9,506**[1]
Taoism	0 (<0.1%)
Confucianism	4 (<0.1%)
Primal, Animist, or Folk religion	34 (0.4%)
Spiritism	0 (0%)
Umbanda, Candomble, and other African-derived religions	0 (0%)
Chinese folk/traditional religion	0 (0%)
Some other religion	60 (0.6%)
No religion/Atheist/Agnostic	3,815 (40%)
(Missing)	99 (1.0%)
**Parent marital status**	
Parents married	7,620 (80%)
Divorced	927 (9.8%)
Parents were never married	578 (6.1%)
One or both parents had died	245 (2.6%)
(Missing)	136 (1.4%)
**Age 12 religious service attendance**	
At least 1/week	1,943 (20%)
1-3/month	1,899 (20%)
<1/month	2,887 (30%)
Never	2,749 (29%)
(Missing)	27 (0.3%)
**Relationship with mother**	
Very good	5,497 (58%)
Somewhat good	3,031 (32%)
Somewhat bad	496 (5.2%)
Very bad	187 (2.0%)
Does not apply	241 (2.5%)
(Missing)	54 (0.6%)
**Relationship with father**	
Very good	4,652 (49%)
Somewhat good	3,012 (32%)
Somewhat bad	846 (8.9%)
Very bad	385 (4.0%)
Does not apply	538 (5.7%)
(Missing)	73 (0.8%)
**Outsider growing up**	
Yes	1,105 (12%)
No	8,262 (87%)
(Missing)	139 (1.5%)
**Self-reported history of abuse**	
Yes	1,086 (11%)
No	8,321 (88%)
(Missing)	99 (1.0%)
**Self-rated health growing up**	
Excellent	2,633 (28%)
Very good	3,518 (37%)
Good	2,582 (27%)
Fair	612 (6.4%)
Poor	134 (1.4%)
(Missing)	26 (0.3%)
**Subjective financial status of family growing up**	
Lived comfortably	3,177 (33%)
Got by	4,508 (47%)
Found it difficult	1,481 (16%)
Found it very difficult	314 (3.3%)
(Missing)	26 (0.3%)
**Religious affiliation at age 12**	
Christianity	5,751 (61%)
Islam	350 (3.7%)

Characteristic	N = 9,506[1]
Hinduism	15 (0.2%)
Buddhism	25 (0.3%)
Judaism	18 (0.2%)
Sikhism	5 (<0.1%)
Baha'i	2 (<0.1%)
Jainism	1 (<0.1%)
Shinto	0 (0%)
Taoism	0 (0%)
Confucianism	4 (<0.1%)
Primal, Animist, or Folk religion	19 (0.2%)
Spiritism	0 (0%)
Umbanda, Candomble, and other African-derived religions	0 (0%)
Chinese folk/traditional religion	0 (0%)
Some other religion	67 (0.7%)
No religion/Atheist/Agnostic	3,163 (33%)
(Missing)	85 (0.9%)

[1]n (%)

Table S5b. Means by demographic category for Germany (N=9506)

Variable	Category	Secure Flourishing Index				Flourishing Index			
		Mean	95% CI	SE	Global p-value	Mean	95% CI	SE	Global p-value
Age group	18-24	6.81	(6.67,6.94)	0.07	4.74e-11	6.70	(6.56,6.84)	0.07	4.62e-14
	25-29	7.06	(6.95,7.17)	0.06		6.97	(6.87,7.08)	0.05	
	30-39	7.10	(7.02,7.19)	0.04		7.02	(6.93,7.10)	0.04	
	40-49	7.01	(6.92,7.10)	0.04		6.90	(6.81,6.99)	0.05	
	50-59	7.07	(6.98,7.15)	0.04		6.95	(6.87,7.04)	0.04	
	60-69	7.19	(7.12,7.27)	0.04		7.07	(7.00,7.15)	0.04	
	70-79	7.33	(7.22,7.43)	0.05		7.26	(7.15,7.37)	0.06	
	80 or older	7.51	(7.31,7.71)	0.10		7.54	(7.34,7.74)	0.10	
Gender	Female	7.07	(7.02,7.12)	0.03	2.64e-07	6.96	(6.91,7.01)	0.03	3.38e-10
	Male	7.14	(7.09,7.19)	0.03		7.06	(7.01,7.11)	0.03	
	Other	5.69	(5.02,6.37)	0.27		5.45	(4.80,6.10)	0.26	
Marital status	Divorced	6.87	(6.72,7.01)	0.07	< 2e-16	6.71	(6.55,6.86)	0.08	< 2e-16
	Domestic partner	7.17	(7.06,7.29)	0.06		7.05	(6.93,7.17)	0.06	
	Married	7.33	(7.29,7.38)	0.02		7.24	(7.19,7.29)	0.02	
	Separated	6.89	(6.73,7.06)	0.08		6.84	(6.66,7.01)	0.09	
	Single/Never been married	6.75	(6.69,6.82)	0.03		6.67	(6.60,6.74)	0.03	
	Widowed	7.16	(7.00,7.32)	0.08		7.05	(6.90,7.20)	0.08	
Employment	Employed for an employer	7.16	(7.11,7.21)	0.02	< 2e-16	7.06	(7.02,7.11)	0.02	< 2e-16
	Homemaker	6.78	(6.54,7.02)	0.12		6.65	(6.41,6.90)	0.12	
	None of these/Other	6.53	(6.23,6.83)	0.15		6.44	(6.13,6.75)	0.16	
	Retired	7.14	(7.07,7.21)	0.03		7.05	(6.98,7.12)	0.04	
	Self-employed	7.35	(7.22,7.47)	0.06		7.26	(7.14,7.39)	0.06	
	Student	6.93	(6.80,7.06)	0.07		6.82	(6.69,6.95)	0.07	
	Unemployed and looking for a job	6.30	(6.08,6.53)	0.11		6.09	(5.85,6.32)	0.12	
Religious service attendance	A few times a year	7.29	(7.23,7.36)	0.03	< 2e-16	7.17	(7.11,7.24)	0.03	< 2e-16
	More than once a week	7.56	(7.38,7.75)	0.09		7.50	(7.32,7.68)	0.09	

Variable	Category	Secure Flourishing Index				Flourishing Index			
		Mean	95% CI	SE	Global p-value	Mean	95% CI	SE	Global p-value
	Never	6.95	(6.90,6.99)	0.02		6.86	(6.81,6.90)	0.02	
	Once a week	7.54	(7.37,7.70)	0.08		7.38	(7.21,7.54)	0.08	
	One to three times a month	7.41	(7.26,7.56)	0.08		7.34	(7.19,7.49)	0.08	
Education	Up to 8	6.67	(6.45,6.88)	0.11	< 2e-16	6.58	(6.35,6.80)	0.11	< 2e-16
	9 to 15	7.35	(7.29,7.41)	0.03		7.31	(7.25,7.37)	0.03	
	16+	6.99	(6.95,7.04)	0.02		6.86	(6.82,6.91)	0.02	
Immigration status	Born in another country	7.13	(7.00,7.26)	0.07	0.685	7.01	(6.88,7.14)	0.07	0.965
	Born in this country	7.10	(7.07,7.14)	0.02		7.01	(6.97,7.04)	0.02	
Religious affiliation	Baha'i	8.82	*	*	< 2e-16	8.73	*	*	< 2e-16
	Buddhism	7.39	(7.01,7.77)	0.19		7.24	(6.88,7.60)	0.18	
	Christianity	7.19	(7.15,7.24)	0.02		7.09	(7.04,7.14)	0.02	
	Hinduism	7.26	*	*		7.01	*	*	
	Islam	7.01	(6.77,7.25)	0.12		6.88	(6.64,7.12)	0.12	
	Judaism	6.98	(6.28,7.67)	0.26		7.00	(6.31,7.69)	0.26	
	No religion/Atheist/ Agnostic	7.01	(6.95,7.06)	0.03		6.92	(6.87,6.97)	0.03	
	Primal, Animist, or Folk religion	6.79	(6.22,7.37)	0.27		6.65	(5.99,7.31)	0.31	
	Shinto	3.90	*	*		4.00	*	*	
	Sikhism	7.45	*	*		7.56	*	*	
	Some other religion	6.44	(5.63,7.25)	0.40		6.24	(5.41,7.07)	0.41	
	Taoism	9.00	*	*		9.00	*	*	
	Confucianism	8.19	*	*		8.49	*	*	

Note. N=9506; $p < .007 = 0.05/7$ (Bonferroni corrected p-value significance threshold); Mean, estimated group mean; CI, confidence interval for the mean within group; SE, complex survey adjusted standard error or the mean; Global p-value, two-tailed Wald-type test of whether there is evidence of any differences in mean scores among groups of a demographic characteristic. *Estimate is not reported due to multiple-imputation and complex survey adjusted degrees of freedom was less than 1.00 leading to insufficient information to provide an estimate of the uncertainty in the estimate. These groups are removed when estimating the global test of mean differences.

Table S5c. Childhood predictors regression analysis results for Germany (N=9506)

		Secure Flourishing Index					Flourishing Index				
Variable	Category	Est	95% CI	SE	Est/SD	Global p-value	Est	95% CI	SE	Est/SD	Global p-value
Relationship with mother	(Ref: Very bad/somewhat bad)					0.023					0.016
	Very good/somewhat good	0.17	(0.02,0.32)	0.07	0.12		0.18	(0.03,0.33)	0.08	0.13	
Relationship with father	(Ref: Very bad/somewhat bad)					0.159					0.302
	Very good/somewhat good	0.08	(-0.03,0.20)	0.06	0.06		0.06	(-0.06,0.17)	0.06	0.04	
Parent marital status	(Ref: Parents married)					0.073					0.063
	Divorced	-0.11	(-0.23,0.00)	0.06	-0.08		-0.10	(-0.21,0.02)	0.06	-0.07	
	Parents were never married	-0.14	(-0.29,0.01)	0.08	-0.10		-0.14	(-0.28,0.01)	0.08	-0.10	
	One or both parents had died	-0.10	(-0.32,0.12)	0.11	-0.07		-0.16	(-0.39,0.06)	0.11	-0.12	
Subjective financial status of family growing up	(Ref: Got by)					1.34e-04					0.014
	Lived comfortably	0.12	(0.04,0.21)	0.04	0.09		0.09	(0.00,0.17)	0.04	0.06	
	Found it difficult	-0.12	(-0.24,-0.01)	0.06	-0.09		-0.09	(-0.20,0.02)	0.06	-0.07	
	Found it very difficult	-0.27	(-0.49,-0.05)	0.11	-0.20		-0.18	(-0.39,0.03)	0.11	-0.13	
Abuse	(Ref: No)					2.30e-05					2.68e-04
	Yes	-0.24	(-0.36,-0.12)	0.06	-0.18		-0.20	(-0.32,-0.08)	0.06	-0.15	

Variable	Category	Secure Flourishing Index					Flourishing Index				
		Est	95% CI	SE	Est/SD	Global p-value	Est	95% CI	SE	Est/SD	Global p-value
Outsider growing up	(Ref: No)					4.44e-16					2.00e-15
	Yes	-0.44	(-0.55,-0.33)	0.05	-0.32		-0.43	(-0.54,-0.32)	0.05	-0.31	
Self-rated health growing up	(Ref: Good)					< 2e-16					< 2e-16
	Excellent	0.62	(0.51,0.72)	0.05	0.45		0.65	(0.55,0.76)	0.05	0.48	
	Very good	0.30	(0.22,0.39)	0.04	0.22		0.32	(0.23,0.40)	0.04	0.23	
	Fair	-0.19	(-0.35,-0.02)	0.08	-0.14		-0.22	(-0.38,-0.05)	0.09	-0.16	
	Poor	0.43	(0.09,0.77)	0.17	0.31		0.46	(0.13,0.80)	0.17	0.34	
Immigration status	(Ref: Born in this country)					7.27e-05					5.91e-05
	Born in another country	0.29	(0.14,0.43)	0.07	0.21		0.28	(0.14,0.42)	0.07	0.21	
Age 12 religious service attendance	(Ref: Never)					1.94e-09					1.98e-11
	At least 1/week	0.35	(0.24,0.45)	0.05	0.25		0.38	(0.28,0.48)	0.05	0.28	
	1-3/month	0.19	(0.09,0.29)	0.05	0.14		0.18	(0.08,0.28)	0.05	0.13	
	< 1/month	0.14	(0.06,0.23)	0.05	0.11		0.15	(0.06,0.24)	0.05	0.11	
Year of birth	(Ref: 1998-2005; current age: 18-24)					< 2e-16					1.28e-14
	1993-1998; age 25-29	0.26	(0.10,0.43)	0.08	0.19		0.24	(0.08,0.41)	0.08	0.18	
	1983-1993; age 30-39	0.27	(0.11,0.42)	0.08	0.19		0.25	(0.10,0.40)	0.08	0.18	
	1973-1983; age 40-49	0.19	(0.03,0.34)	0.08	0.14		0.19	(0.03,0.34)	0.08	0.13	
	1963-1973; age 50-59	0.25	(0.10,0.41)	0.08	0.18		0.26	(0.11,0.41)	0.08	0.19	

Variable	Category	Secure Flourishing Index					Flourishing Index				
		Est	95% CI	SE	Est/SD	Global p-value	Est	95% CI	SE	Est/SD	Global p-value
	1953-1963; age 60-69	0.43	(0.28,0.58)	0.08	0.31		0.44	(0.28,0.59)	0.08	0.32	
	1943-1953; age 70-79	0.60	(0.43,0.77)	0.09	0.44		0.55	(0.38,0.72)	0.09	0.40	
	1943 or earlier; age 80+	0.88	(0.64,1.12)	0.12	0.64		0.74	(0.50,0.98)	0.12	0.54	
Gender	(Ref: Male)					0.010					0.091
	Female	-0.06	(-0.13,0.01)	0.04	-0.04		-0.02	(-0.09,0.05)	0.04	-0.02	
	Other	-0.77	(-1.33,-0.21)	0.29	-0.56		-0.63	(-1.21,-0.05)	0.29	-0.46	
Religious affiliation	(Ref: No religion/Atheist/Agnostic)					0.424					0.582
	Islam	-0.15	(-0.38,0.08)	0.12	-0.11		-0.11	(-0.34,0.12)	0.12	-0.08	
	Christianity	-0.00	(-0.08,0.07)	0.04	-0.00		-0.00	(-0.08,0.08)	0.04	-0.00	
	Collapsed affiliations with prevalence<3%	-0.23	(-0.64,0.17)	0.21	-0.17		-0.21	(-0.61,0.19)	0.21	-0.15	
Race/ethnicity	(Ref: Plurality group)										

Note. N=9506; p < .004 (Bonferroni corrected threshold); Est., estimated effect of childhood predictor on flourishing score; CI, confidence interval; SE, standard error of the estimated effect; Est/SD, a more standardized measure of effect size--estimated effect of flourishing divided by standard deviation of flourishing--leads to the interpretation, for those with the given status (e.g., those with a good/very good relationship with mother compared to those with bad/very bad) are 0.XX standard deviations higher/lower on flourishing; the Global p-value corresponds to the two-sided joint parameter Wald-type test of whether any of the levels' parameters are non-zero, for history of abuse, outsider, relationship wtih mother/father, this is test of whether the estimated effect is non-zero, for multiple-category predictors (age, health, financial status), this is a joint test of whether any of these effects are non-zero. Note the confidence interval of the effect estimate can contradict the reported global p-value (e.g., for the single-category effects of relationship with mother). In such cases, the reported confidence interval is more robust with corrected degrees of freedom from the pooling across multiple imputations, whereas the global p-value is based on a Wald-type test and is less robust to uncertainty attributable to multiple imputation.

Table S5d. Sensitivity to unmeasured confounding of childhood predictors in Germany (N=9506)

		Secure Flourishing Index		Flourishing Index	
Variable	Category	E-value for Estimate	E-value for 95% CI	E-value for Estimate	E-value for 95% CI
Relationship with mother	(Ref: Very bad/somewhat bad)				
	Very good/somewhat good	1.48	1.14	1.51	1.17
Relationship with father	(Ref: Very bad/somewhat bad)				
	Very good/somewhat good	1.30	1.00	1.24	1.00
Parent marital status	(Ref: Parents married)				
	Divorced	1.37	1.00	1.33	1.00
	Parents were never married	1.42	1.00	1.42	1.00
	One or both parents had died	1.33	1.00	1.48	1.00
Subjective financial status of family growing up	(Ref: Got by)				
	Lived comfortably	1.38	1.18	1.32	1.06
	Found it difficult	1.39	1.09	1.32	1.00
	Found it very difficult	1.67	1.22	1.50	1.00
Abuse	(Ref: No)				
	Yes	1.63	1.39	1.55	1.30
Outsider growing up	(Ref: No)				
	Yes	2.01	1.80	1.99	1.79
Self-rated health growing up	(Ref: Good)				
	Excellent	2.38	2.16	2.46	2.24
	Very good	1.75	1.58	1.78	1.61
	Fair	1.52	1.14	1.58	1.22
	Poor	1.99	1.33	2.06	1.40
Immigration status	(Ref: Born in this country)				
	Born in another country	1.71	1.43	1.71	1.44
Age 12 religious service attendance	(Ref: Never)				
	At least 1/week	1.83	1.63	1.90	1.70
	1-3/month	1.52	1.31	1.51	1.30
	< 1/month	1.43	1.23	1.44	1.24
Year of birth	(Ref: 1998-2005; current age: 18-24)				
	1993-1998; age 25-29	1.66	1.33	1.63	1.29
	1983-1993; age 30-39	1.67	1.37	1.64	1.33

Variable	Category	Secure Flourishing Index		Flourishing Index	
		E-value for Estimate	E-value for 95% CI	E-value for Estimate	E-value for 95% CI
	1973-1983; age 40-49	1.52	1.17	1.52	1.17
	1963-1973; age 50-59	1.65	1.34	1.66	1.35
	1953-1963; age 60-69	1.99	1.70	2.01	1.71
	1943-1953; age 70-79	2.33	1.99	2.24	1.90
	1943 or earlier; age 80+	2.98	2.43	2.66	2.14
Gender	(Ref: Male)				
	Female	1.25	1.00	1.14	1.00
	Other	2.72	1.56	2.41	1.24
Religious affiliation	(Ref: No religion/Atheist/Agnostic)				
	Islam	1.44	1.00	1.36	1.00
	Christianity	1.06	1.00	1.02	1.00
	Collapsed affiliations with prevalence<3%	1.61	1.00	1.57	1.00
Race/ethnicity	(Ref: Plurality group)				

Table S6a. Nationally representative descriptive statistics for Hong Kong

Characteristic	N = 3,012[1]
**Age group**	
1998-2005; age 18-24	217 (7.2%)
1993-1998; age 25-29	198 (6.6%)
1983-1993; age 30-39	507 (17%)
1973-1983; age 40-49	580 (19%)
1963-1973; age 50-59	711 (24%)
1953-1963; age 60-69	620 (21%)
1943-1953; age 70-79	164 (5.5%)
1943 or earlier; age 80+	15 (0.5%)
(Missing)	0 (0%)
**Gender**	
Male	1,390 (46%)
Female	1,620 (54%)
Other	2 (<0.1%)
(Missing)	0 (0%)
**Race/Ethnicity**	
Chinese (Cantonese)	1,930 (64%)
Chinese (Chaoshan)	201 (6.7%)
Chinese (Fujianese)	117 (3.9%)
Chinese (Hakka)	121 (4.0%)
Chinese (Other ethnicity)	264 (8.8%)
Chinese (Shanghainese)	89 (2.9%)
East Asian (Korean, Japanese)	10 (0.3%)
Other	4 (0.1%)
South Asian (Indian, Nepalese, Pakistani)	17 (0.6%)
Southeast Asian (Filipino, Indonesian, Thailand)	46 (1.5%)
Taiwanese	14 (0.4%)
White	15 (0.5%)
(Missing)	184 (6.1%)
**Marital status**	
Married	2,080 (69%)
Separated	21 (0.7%)
Divorced	105 (3.5%)
Widowed	45 (1.5%)
Single, never married	723 (24%)
Domestic Partner	37 (1.2%)
(Missing)	1 (<0.1%)
**Employment**	
Employed for an employer	2,056 (68%)
Self-employed	245 (8.1%)
Retired	423 (14%)
Student	55 (1.8%)
Homemaker	114 (3.8%)
Unemployed and looking for a job	62 (2.0%)
None of these/Other	39 (1.3%)
(Missing)	18 (0.6%)
**Religious service attendance**	
More than 1/week	237 (7.9%)
1/week	567 (19%)
1-3/month	332 (11%)
A few times a year	543 (18%)
Never	1,332 (44%)
(Missing)	1 (<0.1%)
**Education**	
Up to 8 years	433 (14%)
9-15 years	2,031 (67%)
16+ years	547 (18%)
(Missing)	0 (0%)

Characteristic	N = 3,012[1]
**Immigration status**	
Born in this country	2,637 (88%)
Born in another country	321 (11%)
(Missing)	53 (1.8%)
**Religious affiliation as an adult (now)**	
Christianity	757 (25%)
Islam	86 (2.8%)
Hinduism	20 (0.7%)
Buddhism	349 (12%)
Judaism	10 (0.3%)
Sikhism	2 (<0.1%)
Baha'i	3 (<0.1%)
Jainism	1 (<0.1%)
Shinto	19 (0.6%)
Taoism	97 (3.2%)
Confucianism	11 (0.4%)
Primal, Animist, or Folk religion	27 (0.9%)
Spiritism	0 (0%)
Umbanda, Candomble, and other African-derived religions	0 (0%)
Chinese folk/traditional religion	106 (3.5%)
Some other religion	4 (0.1%)
No religion/Atheist/Agnostic	1,518 (50%)
(Missing)	5 (0.2%)
**Parent marital status**	
Parents married	2,752 (91%)
Divorced	114 (3.8%)
Parents were never married	40 (1.3%)
One or both parents had died	50 (1.7%)
(Missing)	56 (1.8%)
**Age 12 religious service attendance**	
At least 1/week	432 (14%)
1-3/month	528 (18%)
<1/month	753 (25%)
Never	1,295 (43%)
(Missing)	4 (0.1%)
**Relationship with mother**	
Very good	1,077 (36%)
Somewhat good	1,164 (39%)
Somewhat bad	293 (9.7%)
Very bad	49 (1.6%)
Does not apply	426 (14%)
(Missing)	3 (<0.1%)
**Relationship with father**	
Very good	868 (29%)
Somewhat good	1,089 (36%)
Somewhat bad	393 (13%)
Very bad	102 (3.4%)
Does not apply	557 (19%)
(Missing)	3 (0.1%)
**Outsider growing up**	
Yes	664 (22%)
No	2,224 (74%)
(Missing)	124 (4.1%)
**Self-reported history of abuse**	
Yes	318 (11%)
No	2,688 (89%)
(Missing)	5 (0.2%)
**Self-rated health growing up**	
Excellent	545 (18%)

Characteristic	N = 3,012[1]
Very good	1,073 (36%)
Good	863 (29%)
Fair	426 (14%)
Poor	91 (3.0%)
(Missing)	13 (0.4%)
**Subjective financial status of family growing up**	
Lived comfortably	906 (30%)
Got by	1,527 (51%)
Found it difficult	473 (16%)
Found it very difficult	84 (2.8%)
(Missing)	22 (0.7%)
**Religious affiliation at age 12**	
Christianity	715 (24%)
Islam	86 (2.9%)
Hinduism	27 (0.9%)
Buddhism	323 (11%)
Judaism	16 (0.5%)
Sikhism	4 (0.1%)
Baha'i	0 (0%)
Jainism	1 (<0.1%)
Shinto	18 (0.6%)
Taoism	81 (2.7%)
Confucianism	10 (0.3%)
Primal, Animist, or Folk religion	15 (0.5%)
Spiritism	0 (0%)
Umbanda, Candomble, and other African-derived religions	0 (0%)
Chinese folk/traditional religion	108 (3.6%)
Some other religion	5 (0.2%)
No religion/Atheist/Agnostic	1,601 (53%)
(Missing)	1 (<0.1%)

[1]n (%)

Table S6b. Means by demographic category for Hong Kong (N=3012)

Variable	Category	Secure Flourishing Index				Flourishing Index			
		Mean	95% CI	SE	Global p-value	Mean	95% CI	SE	Global p-value
Age group	18-24	7.10	(6.90,7.30)	0.10	< 2e-16	7.00	(6.81,7.20)	0.10	< 2e-16
	25-29	6.72	(6.31,7.13)	0.21		6.62	(6.22,7.02)	0.20	
	30-39	6.54	(6.36,6.72)	0.09		6.46	(6.28,6.64)	0.09	
	40-49	7.04	(6.87,7.22)	0.09		6.96	(6.78,7.13)	0.09	
	50-59	7.50	(7.36,7.64)	0.07		7.48	(7.34,7.61)	0.07	
	60-69	7.55	(7.34,7.77)	0.11		7.53	(7.31,7.74)	0.11	
	70-79	7.36	(6.90,7.82)	0.23		7.43	(6.97,7.89)	0.23	
	80 or older	6.26	(6.13,6.39)	0.05		6.24	(6.07,6.41)	0.06	
Gender	Female	7.11	(6.99,7.23)	0.06	3.63e-06	7.06	(6.94,7.18)	0.06	0.010
	Male	7.24	(7.13,7.35)	0.06		7.19	(7.07,7.30)	0.06	
	Other	6.48	*	*		6.42	*	*	
Marital status	Divorced	6.96	(6.40,7.51)	0.28	< 2e-16	6.98	(6.42,7.54)	0.28	< 2e-16
	Domestic partner	6.43	(5.86,7.00)	0.28		6.32	(5.81,6.84)	0.25	
	Married	7.52	(7.42,7.61)	0.05		7.45	(7.36,7.55)	0.05	
	Separated	7.19	(6.03,8.34)	0.53		7.26	(6.18,8.34)	0.50	
	Single/Never been married	6.26	(6.10,6.42)	0.08		6.22	(6.06,6.39)	0.08	
	Widowed	6.79	(6.18,7.40)	0.30		6.83	(6.27,7.40)	0.28	
Employment	Employed for an employer	7.23	(7.15,7.32)	0.04	6.25e-08	7.17	(7.09,7.25)	0.04	1.52e-07
	Homemaker	6.71	(6.24,7.17)	0.24		6.64	(6.17,7.12)	0.24	
	None of these/Other	5.11	(3.46,6.77)	0.81		5.12	(3.57,6.67)	0.76	
	Retired	7.20	(6.92,7.47)	0.14		7.22	(6.95,7.49)	0.14	
	Self-employed	7.58	(7.23,7.93)	0.18		7.52	(7.16,7.88)	0.18	
	Student	6.51	(6.09,6.93)	0.21		6.55	(6.15,6.95)	0.20	
	Unemployed and looking for a job	5.83	(5.18,6.48)	0.33		5.70	(5.01,6.39)	0.34	
Religious service attendance	A few times a year	6.83	(6.63,7.03)	0.10	< 2e-16	6.79	(6.59,6.99)	0.10	< 2e-16
	More than once a week	9.05	(8.88,9.22)	0.09		9.00	(8.83,9.16)	0.09	

Variable	Category	Secure Flourishing Index				Flourishing Index			
		Mean	95% CI	SE	Global p-value	Mean	95% CI	SE	Global p-value
	Never	6.71	(6.59,6.84)	0.06		6.67	(6.54,6.79)	0.06	
	Once a week	7.60	(7.39,7.81)	0.11		7.54	(7.34,7.75)	0.10	
	One to three times a month	7.46	(7.27,7.64)	0.09		7.39	(7.21,7.58)	0.09	
Education	Up to 8	7.33	(7.02,7.65)	0.16	3.47e-04	7.31	(7.00,7.62)	0.16	1.75e-04
	9 to 15	6.87	(6.72,7.02)	0.08		6.81	(6.66,6.96)	0.08	
	16+	7.21	(7.12,7.31)	0.05		7.16	(7.06,7.25)	0.05	
Immigration status	Born in another country	6.70	(6.31,7.08)	0.20	0.008	6.67	(6.29,7.06)	0.19	0.012
	Born in this country	7.23	(7.15,7.30)	0.04		7.17	(7.09,7.25)	0.04	
Religious affiliation	Baha'i	8.26	*	*	< 2e-16	8.27	*	*	< 2e-16
	Buddhism	7.81	(7.56,8.06)	0.13		7.76	(7.51,8.00)	0.13	
	Christianity	7.26	(7.11,7.42)	0.08		7.20	(7.04,7.36)	0.08	
	Hinduism	7.73	(6.43,9.03)	0.41		7.66	(6.37,8.95)	0.41	
	Islam	7.64	(6.58,8.69)	0.53		7.64	(6.62,8.65)	0.51	
	Judaism	8.11	*	*		7.96	*	*	
	No religion/Atheist/ Agnostic	6.89	(6.78,7.00)	0.05		6.84	(6.73,6.95)	0.06	
	Primal, Animist, or Folk religion	6.76	(6.10,7.42)	0.29		6.60	(5.96,7.24)	0.28	
	Shinto	8.53	(8.05,9.01)	0.11		8.41	(7.94,8.87)	0.11	
	Sikhism	9.24	*	*		9.21	*	*	
	Some other religion	5.89	*	*		5.91	*	*	
	Taoism	7.12	(6.71,7.54)	0.21		7.05	(6.62,7.47)	0.21	
	Confucianism	7.73	*	*		7.69	*	*	
	Jainism	8.60	*	*		8.67	*	*	
	Chinese folk/traditional religion	7.62	(7.29,7.94)	0.16		7.60	(7.28,7.92)	0.16	

Note. N=3012; p < .007 = 0.05/7 (Bonferroni corrected p-value significance threshold); Mean, estimated group mean; CI, confidence interval for the mean within group; SE, complex survey adjusted standard error or the mean; Global p-value, two-tailed Wald-type test of whether there is evidence of any differences in mean scores among groups of a demographic characteristic. *Estimate is not reported due to multiple-imputation and complex survey adjusted degrees of

Variable	Category	Secure Flourishing Index Mean	95% CI	SE	Global p-value	Flourishing Index Mean	95% CI	SE	Global p-value

freedom was less than 1.00 leading to insufficient information to provide an estimate of the uncertainty in the estimate. These groups are removed when estimating the global test of mean differences.

Table S6c. Childhood predictors regression analysis results for Hong Kong (N=3012)

		Secure Flourishing Index					Flourishing Index				
Variable	Category	Est	95% CI	SE	Est/SD	Global p-value	Est	95% CI	SE	Est/SD	Global p-value
Relationship with mother	(Ref: Very bad/somewhat bad)					0.157					0.225
	Very good/somewhat good	0.13	(-0.07,0.33)	0.10	0.08		0.12	(-0.08,0.32)	0.10	0.07	
Relationship with father	(Ref: Very bad/somewhat bad)					0.092					0.051
	Very good/somewhat good	0.16	(-0.04,0.37)	0.10	0.09		0.19	(-0.02,0.41)	0.11	0.11	
Parent marital status	(Ref: Parents married)					0.415					0.375
	Divorced	-0.12	(-0.52,0.27)	0.20	-0.07		-0.18	(-0.57,0.22)	0.20	-0.10	
	Parents were never married	0.49	(-0.13,1.12)	0.32	0.28		0.50	(-0.15,1.15)	0.33	0.29	
	One or both parents had died	0.01	(-0.67,0.69)	0.35	0.01		-0.06	(-0.70,0.58)	0.33	-0.04	
Subjective financial status of family growing up	(Ref: Got by)					4.44e-16					1.11e-15
	Lived comfortably	0.71	(0.53,0.88)	0.09	0.40		0.70	(0.53,0.87)	0.09	0.40	
	Found it difficult	-0.25	(-0.48,-0.02)	0.12	-0.14		-0.22	(-0.45,0.01)	0.12	-0.13	
	Found it very difficult	-0.30	(-0.82,0.23)	0.27	-0.17		-0.14	(-0.68,0.40)	0.28	-0.08	
Abuse	(Ref: No)					0.488					0.701
	Yes	-0.08	(-0.30,0.14)	0.11	-0.04		-0.04	(-0.27,0.18)	0.12	-0.03	

Variable	Category	Secure Flourishing Index					Flourishing Index				
		Est	95% CI	SE	Est/SD	Global p-value	Est	95% CI	SE	Est/SD	Global p-value
Outsider growing up	(Ref: No)					0.162					0.146
	Yes	-0.14	(-0.33,0.06)	0.10	-0.08		-0.14	(-0.32,0.05)	0.10	-0.08	
Self-rated health growing up	(Ref: Good)					< 2e-16					< 2e-16
	Excellent	1.52	(1.22,1.82)	0.15	0.87		1.52	(1.23,1.81)	0.15	0.87	
	Very good	0.76	(0.59,0.92)	0.08	0.43		0.79	(0.62,0.95)	0.08	0.45	
	Fair	-0.73	(-0.94,-0.52)	0.11	-0.42		-0.75	(-0.96,-0.53)	0.11	-0.43	
	Poor	-1.49	(-2.19,-0.78)	0.36	-0.85		-1.48	(-2.23,-0.73)	0.38	-0.85	
Immigration status	(Ref: Born in this country)					0.264					0.164
	Born in another country	-0.17	(-0.47,0.13)	0.15	-0.10		-0.21	(-0.50,0.09)	0.15	-0.12	
Age 12 religious service attendance	(Ref: Never)					8.86e-06					1.80e-06
	At least 1/week	0.59	(0.31,0.86)	0.14	0.33		0.68	(0.41,0.96)	0.14	0.39	
	1-3/month	0.52	(0.31,0.74)	0.11	0.30		0.55	(0.33,0.78)	0.11	0.32	
	< 1/month	0.10	(-0.08,0.28)	0.09	0.06		0.14	(-0.04,0.32)	0.09	0.08	
Year of birth	(Ref: 1998-2005; current age: 18-24)					< 2e-16					< 2e-16
	1993-1998; age 25-29	-0.05	(-0.31,0.21)	0.13	-0.03		-0.03	(-0.30,0.23)	0.14	-0.02	
	1983-1993; age 30-39	0.03	(-0.17,0.23)	0.10	0.02		0.03	(-0.18,0.23)	0.11	0.01	
	1973-1983; age 40-49	0.31	(0.12,0.51)	0.10	0.18		0.31	(0.11,0.51)	0.10	0.18	
	1963-1973; age 50-59	0.62	(0.43,0.81)	0.10	0.35		0.55	(0.36,0.74)	0.10	0.31	

		Secure Flourishing Index					Flourishing Index				
Variable	Category	Est	95% CI	SE	Est/SD	Global p-value	Est	95% CI	SE	Est/SD	Global p-value
	1953-1963; age 60-69	0.99	(0.73,1.24)	0.13	0.56		0.91	(0.65,1.17)	0.13	0.52	
	1943-1953; age 70-79	1.29	(0.77,1.81)	0.26	0.74		1.14	(0.63,1.65)	0.26	0.65	
	1943 or earlier; age 80+	-0.26	(-0.81,0.30)	0.28	-0.15		-0.32	(-0.86,0.22)	0.28	-0.18	
Gender	(Ref: Male)					0.008					0.040
	Female	0.13	(0.01,0.25)	0.06	0.08		0.12	(0.00,0.24)	0.06	0.07	
	Other	-0.34	(-0.68,-0.00)	0.17	-0.20		-0.39	(-0.93,0.16)	0.28	-0.22	
Religious affiliation	(Ref: No religion/Atheist/Agnostic)					0.030					0.034
	Buddhism	0.05	(-0.18,0.27)	0.12	0.03		0.03	(-0.20,0.26)	0.12	0.02	
	Chinese folk/traditional religion	0.14	(-0.16,0.44)	0.15	0.08		0.10	(-0.22,0.42)	0.16	0.06	
	Christianity	-0.17	(-0.41,0.07)	0.12	-0.10		-0.21	(-0.45,0.02)	0.12	-0.12	
	Collapsed affiliations with prevalence<3%	0.25	(-0.04,0.54)	0.15	0.14		0.20	(-0.09,0.50)	0.15	0.12	
Race/ethnicity	(Ref: Plurality group)					0.324					0.149
	Non-plurality groups	0.06	(-0.10,0.23)	0.08	0.04		0.10	(-0.06,0.27)	0.08	0.06	

Note. N=3012; p < .004 (Bonferroni corrected threshold); Est., estimated effect of childhood predictor on flourishing score; CI, confidence interval; SE, standard error of the estimated effect; Est/SD, a more standardized measure of effect size--estimated effect of flourishing divided by standard deviation of flourishing--leads to the interpretation, for those with the given status (e.g., those with a good/very good relationship with mother compared to those with bad/very bad) are 0.XX standard deviations higher/lower on flourishing; the Global p-value corresponds to the two-sided joint parameter Wald-type test of whether any of the levels' parameters are non-zero, for history of abuse, outsider, relationship wtih mother/father, this is test of whether the estimated effect is non-zero, for multiple-category predictors (age, health, financial status), this is a joint test of whether any of these effects are non-zero. Note the confidence interval of the effect estimate can contradict the reported global p-value (e.g., for the single-category effects of relationship with mother). In such cases, the reported confidence

Variable	Category	Secure Flourishing Index					Flourishing Index				
		Est	95% CI	SE	Est/SD	Global p-value	Est	95% CI	SE	Est/SD	Global p-value

interval is more robust with corrected degrees of freedom from the pooling across multiple imputations, whereas the global p-value is based on a Wald-type test and is less robust to uncertainty attributable to multiple imputation.

Table S6d. Sensitivity to unmeasured confounding of childhood predictors in Hong Kong (N=3012)

		Secure Flourishing Index		Flourishing Index	
Variable	Category	E-value for Estimate	E-value for 95% CI	E-value for Estimate	E-value for 95% CI
Relationship with mother	(Ref: Very bad/somewhat bad)				
	Very good/somewhat good	1.35	1.00	1.32	1.00
Relationship with father	(Ref: Very bad/somewhat bad)				
	Very good/somewhat good	1.40	1.00	1.45	1.00
Parent marital status	(Ref: Parents married)				
	Divorced	1.33	1.00	1.42	1.00
	Parents were never married	1.91	1.00	1.92	1.00
	One or both parents had died	1.08	1.00	1.22	1.00
Subjective financial status of family growing up	(Ref: Got by)				
	Lived comfortably	2.25	1.97	2.23	1.96
	Found it difficult	1.54	1.13	1.49	1.00
	Found it very difficult	1.61	1.00	1.36	1.00
Abuse	(Ref: No)				
	Yes	1.25	1.00	1.18	1.00
Outsider growing up	(Ref: No)				
	Yes	1.35	1.00	1.35	1.00
Self-rated health growing up	(Ref: Good)				
	Excellent	3.83	3.18	3.81	3.19
	Very good	2.33	2.06	2.37	2.11
	Fair	2.28	1.95	2.31	1.97
	Poor	3.75	2.37	3.74	2.29
Immigration status	(Ref: Born in this country)				
	Born in another country	1.41	1.00	1.47	1.00
Age 12 religious service attendance	(Ref: Never)				
	At least 1/week	2.05	1.63	2.20	1.77
	1-3/month	1.95	1.62	2.00	1.65
	< 1/month	1.30	1.00	1.36	1.00
Year of birth	(Ref: 1998-2005; current age: 18-24)				
	1993-1998; age 25-29	1.19	1.00	1.15	1.00
	1983-1993; age 30-39	1.14	1.00	1.13	1.00

Variable	Category	Secure Flourishing Index		Flourishing Index	
		E-value for Estimate	E-value for 95% CI	E-value for Estimate	E-value for 95% CI
	1973-1983; age 40-49	1.63	1.32	1.63	1.31
	1963-1973; age 50-59	2.11	1.81	1.99	1.70
	1953-1963; age 60-69	2.73	2.28	2.59	2.16
	1943-1953; age 70-79	3.32	2.35	3.01	2.11
	1943 or earlier; age 80+	1.55	1.00	1.64	1.00
Gender	(Ref: Male)				
	Female	1.35	1.09	1.33	1.05
	Other	1.68	1.06	1.74	1.00
Religious affiliation	(Ref: No religion/Atheist/Agnostic)				
	Buddhism	1.18	1.00	1.13	1.00
	Chinese folk/traditional religion	1.36	1.00	1.29	1.00
	Christianity	1.41	1.00	1.48	1.00
	Collapsed affiliations with prevalence<3%	1.54	1.00	1.46	1.00
Race/ethnicity	(Ref: Plurality group)				
	Non-plurality groups	1.22	1.00	1.29	1.00

Table S7a. Nationally representative descriptive statistics for India

**Characteristic**	**N = 12,765**[1]
**Age group**	
1998-2005; age 18-24	2,543 (20%)
1993-1998; age 25-29	1,640 (13%)
1983-1993; age 30-39	3,109 (24%)
1973-1983; age 40-49	2,275 (18%)
1963-1973; age 50-59	1,574 (12%)
1953-1963; age 60-69	1,188 (9.3%)
1943-1953; age 70-79	370 (2.9%)
1943 or earlier; age 80+	67 (0.5%)
(Missing)	0 (0%)
**Gender**	
Male	6,473 (51%)
Female	6,292 (49%)
Other	0 (0%)
(Missing)	0 (0%)
**Race/Ethnicity**	
General	3,538 (28%)
Other backward caste	4,177 (33%)
Schedule caste	3,599 (28%)
Schedule tribe	1,185 (9.3%)
(Missing)	267 (2.1%)
**Marital status**	
Married	9,848 (77%)
Separated	45 (0.4%)
Divorced	25 (0.2%)
Widowed	445 (3.5%)
Single, never married	2,065 (16%)
Domestic Partner	269 (2.1%)
(Missing)	69 (0.5%)
**Employment**	
Employed for an employer	2,660 (21%)
Self-employed	3,401 (27%)
Retired	286 (2.2%)
Student	532 (4.2%)
Homemaker	4,221 (33%)
Unemployed and looking for a job	902 (7.1%)
None of these/Other	715 (5.6%)
(Missing)	48 (0.4%)
**Religious service attendance**	
More than 1/week	2,875 (23%)
1/week	3,166 (25%)
1-3/month	2,740 (21%)
A few times a year	2,090 (16%)
Never	1,823 (14%)
(Missing)	71 (0.6%)
**Education**	
Up to 8 years	11,422 (89%)
9-15 years	1,194 (9.4%)
16+ years	145 (1.1%)
(Missing)	4 (<0.1%)
**Immigration status**	
Born in this country	12,629 (99%)
Born in another country	110 (0.9%)
(Missing)	26 (0.2%)
**Religious affiliation as an adult (now)**	
Christianity	306 (2.4%)
Islam	1,555 (12%)
Hinduism	10,362 (81%)

Characteristic	N = 12,765[1]
Buddhism	230 (1.8%)
Judaism	0 (0%)
Sikhism	127 (1.0%)
Baha'i	0 (0%)
Jainism	10 (<0.1%)
Shinto	1 (<0.1%)
Taoism	0 (0%)
Confucianism	0 (0%)
Primal, Animist, or Folk religion	30 (0.2%)
Spiritism	0 (0%)
Umbanda, Candomble, and other African-derived religions	0 (0%)
Chinese folk/traditional religion	0 (0%)
Some other religion	67 (0.5%)
No religion/Atheist/Agnostic	13 (0.1%)
(Missing)	62 (0.5%)
**Parent marital status**	
Parents married	5,578 (44%)
Divorced	236 (1.8%)
Parents were never married	1,055 (8.3%)
One or both parents had died	940 (7.4%)
(Missing)	4,956 (39%)
**Age 12 religious service attendance**	
At least 1/week	5,288 (41%)
1-3/month	2,959 (23%)
<1/month	2,719 (21%)
Never	1,478 (12%)
(Missing)	321 (2.5%)
**Relationship with mother**	
Very good	11,465 (90%)
Somewhat good	788 (6.2%)
Somewhat bad	88 (0.7%)
Very bad	73 (0.6%)
Does not apply	269 (2.1%)
(Missing)	82 (0.6%)
**Relationship with father**	
Very good	10,923 (86%)
Somewhat good	995 (7.8%)
Somewhat bad	126 (1.0%)
Very bad	100 (0.8%)
Does not apply	481 (3.8%)
(Missing)	141 (1.1%)
**Outsider growing up**	
Yes	1,926 (15%)
No	10,780 (84%)
(Missing)	59 (0.5%)
**Self-reported history of abuse**	
Yes	1,468 (11%)
No	10,526 (82%)
(Missing)	771 (6.0%)
**Self-rated health growing up**	
Excellent	2,182 (17%)
Very good	3,882 (30%)
Good	4,028 (32%)
Fair	2,202 (17%)
Poor	424 (3.3%)
(Missing)	47 (0.4%)
**Subjective financial status of family growing up**	
Lived comfortably	4,946 (39%)
Got by	3,010 (24%)

Characteristic	N = 12,765[1]
Found it difficult	2,703 (21%)
Found it very difficult	2,035 (16%)
(Missing)	70 (0.5%)
**Religious affiliation at age 12**	
Christianity	254 (2.0%)
Islam	1,550 (12%)
Hinduism	10,417 (82%)
Buddhism	180 (1.4%)
Judaism	0 (0%)
Sikhism	126 (1.0%)
Baha'i	0 (0%)
Jainism	9 (<0.1%)
Shinto	4 (<0.1%)
Taoism	0 (0%)
Confucianism	0 (0%)
Primal, Animist, or Folk religion	27 (0.2%)
Spiritism	0 (0%)
Umbanda, Candomble, and other African-derived religions	0 (0%)
Chinese folk/traditional religion	0 (0%)
Some other religion	59 (0.5%)
No religion/Atheist/Agnostic	7 (<0.1%)
(Missing)	131 (1.0%)

[1]n (%)

Table S7b. Means by demographic category for India (N=12765)

Variable	Category	Secure Flourishing Index				Flourishing Index			
		Mean	95% CI	SE	Global p-value	Mean	95% CI	SE	Global p-value
Age group	18-24	7.90	(7.80,8.01)	0.05	< 2e-16	7.29	(7.20,7.38)	0.05	< 2e-16
	25-29	7.63	(7.53,7.74)	0.05		7.05	(6.95,7.15)	0.05	
	30-39	7.40	(7.32,7.49)	0.04		6.82	(6.75,6.90)	0.04	
	40-49	7.28	(7.18,7.38)	0.05		6.72	(6.62,6.81)	0.05	
	50-59	7.13	(7.01,7.25)	0.06		6.61	(6.49,6.73)	0.06	
	60-69	7.03	(6.87,7.19)	0.08		6.54	(6.39,6.69)	0.08	
	70-79	6.99	(6.70,7.27)	0.15		6.50	(6.24,6.77)	0.14	
	80 or older	7.16	(6.33,7.98)	0.41		6.92	(6.12,7.72)	0.40	
Gender	Female	7.49	(7.42,7.56)	0.04	0.004	6.88	(6.82,6.94)	0.03	0.388
	Male	7.37	(7.30,7.44)	0.03		6.85	(6.78,6.91)	0.03	
Marital status	Divorced	6.94	(5.98,7.89)	0.45	< 2e-16	6.56	(5.79,7.33)	0.36	7.77e-16
	Domestic partner	7.06	(6.69,7.44)	0.19		6.68	(6.36,7.00)	0.16	
	Married	7.41	(7.35,7.47)	0.03		6.83	(6.78,6.88)	0.03	
	Separated	6.37	(5.62,7.12)	0.37		5.89	(5.20,6.59)	0.34	
	Single/Never been married	7.78	(7.67,7.88)	0.05		7.22	(7.13,7.32)	0.05	
	Widowed	6.63	(6.35,6.90)	0.14		6.23	(5.95,6.50)	0.14	
Employment	Employed for an employer	7.32	(7.22,7.42)	0.05	3.97e-09	6.80	(6.70,6.89)	0.05	9.06e-07
	Homemaker	7.46	(7.38,7.53)	0.04		6.88	(6.81,6.95)	0.04	
	None of these/Other	7.21	(6.97,7.45)	0.12		6.66	(6.44,6.88)	0.11	
	Retired	7.35	(7.02,7.68)	0.17		6.92	(6.61,7.23)	0.16	
	Self-employed	7.51	(7.41,7.61)	0.05		6.93	(6.84,7.02)	0.05	
	Student	7.93	(7.77,8.10)	0.08		7.29	(7.13,7.45)	0.08	
	Unemployed and looking for a job	7.22	(7.03,7.41)	0.10		6.67	(6.50,6.84)	0.09	
Religious service attendance	A few times a year	7.35	(7.25,7.45)	0.05	0.001	6.79	(6.71,6.88)	0.05	0.028
	More than once a week	7.44	(7.35,7.54)	0.05		6.89	(6.80,6.98)	0.05	
	Never	7.26	(7.12,7.39)	0.07		6.75	(6.62,6.87)	0.07	

Variable	Category	Secure Flourishing Index				Flourishing Index			
		Mean	95% CI	SE	Global p-value	Mean	95% CI	SE	Global p-value
	Once a week	7.57	(7.47,7.66)	0.05		6.96	(6.87,7.05)	0.05	
	One to three times a month	7.44	(7.34,7.54)	0.05		6.86	(6.77,6.95)	0.05	
Education	Up to 8	7.38	(7.32,7.44)	0.03	2.55e-15	6.81	(6.76,6.86)	0.03	< 2e-16
	9 to 15	8.01	(7.76,8.26)	0.13		7.49	(7.26,7.72)	0.12	
	16+	7.83	(7.72,7.93)	0.05		7.28	(7.19,7.37)	0.05	
Immigration status	Born in another country	6.73	(6.35,7.12)	0.19	2.72e-04	6.32	(5.90,6.74)	0.21	0.009
	Born in this country	7.44	(7.38,7.49)	0.03		6.87	(6.82,6.92)	0.02	
Religious affiliation	Buddhism	7.51	(7.15,7.87)	0.18	< 2e-16	6.87	(6.54,7.20)	0.17	< 2e-16
	Christianity	7.76	(7.51,8.00)	0.12		7.09	(6.87,7.31)	0.11	
	Hinduism	7.44	(7.38,7.50)	0.03		6.88	(6.83,6.93)	0.03	
	Islam	7.28	(7.12,7.43)	0.08		6.74	(6.59,6.89)	0.08	
	No religion/Atheist/Agnostic	6.75	(2.15,10.00ǂ)	0.89		6.41	(1.49,10.00ǂ)	0.95	
	Primal, Animist, or Folk religion	7.21	(6.27,8.14)	0.44		6.67	(5.94,7.40)	0.35	
	Shinto	10.00	*	*		10.00	*	*	
	Sikhism	7.40	(7.06,7.74)	0.17		6.83	(6.44,7.22)	0.20	
	Some other religion	7.46	(6.72,8.19)	0.37		6.69	(6.11,7.28)	0.29	
	Jainism	6.98	*	*		6.50	*	*	

Note. N=12765; p < .007 = 0.05/7 (Bonferroni corrected p-value significance threshold); Mean, estimated group mean; CI, confidence interval for the mean within group; SE, complex survey adjusted standard error or the mean; Global p-value, two-tailed Wald-type test of whether there is evidence of any differences in mean scores among groups of a demographic characteristic. *Estimate is not reported due to multiple-imputation and complex survey adjusted degrees of freedom was less than 1.00 leading to insufficient information to provide an estimate of the uncertainty in the estimate. These groups are removed when estimating the global test of mean differences.

Table S7c. Childhood predictors regression analysis results for India (N=12765)

		Secure Flourishing Index					Flourishing Index				
Variable	Category	Est	95% CI	SE	Est/SD	Global p-value	Est	95% CI	SE	Est/SD	Global p-value
Relationship with mother	(Ref: Very bad/somewhat bad)					0.334					0.167
	Very good/somewhat good	0.15	(-0.16,0.46)	0.16	0.07		0.23	(-0.10,0.56)	0.17	0.12	
Relationship with father	(Ref: Very bad/somewhat bad)					0.731					0.670
	Very good/somewhat good	-0.04	(-0.29,0.22)	0.13	-0.02		0.05	(-0.22,0.33)	0.14	0.03	
Parent marital status	(Ref: Parents married)					0.025					0.033
	Divorced	0.21	(-0.07,0.48)	0.14	0.10		0.28	(-0.06,0.61)	0.16	0.15	
	Parents were never married	-0.10	(-0.24,0.04)	0.07	-0.05		-0.07	(-0.22,0.08)	0.07	-0.04	
	One or both parents had died	-0.10	(-0.27,0.06)	0.08	-0.05		0.09	(-0.09,0.26)	0.09	0.05	
Subjective financial status of family growing up	(Ref: Got by)					8.55e-13					2.15e-12
	Lived comfortably	0.13	(0.03,0.23)	0.05	0.07		0.09	(-0.03,0.20)	0.06	0.05	
	Found it difficult	-0.18	(-0.30,-0.06)	0.06	-0.09		-0.19	(-0.32,-0.06)	0.07	-0.10	
	Found it very difficult	-0.38	(-0.51,-0.24)	0.07	-0.18		-0.44	(-0.59,-0.30)	0.08	-0.23	
Abuse	(Ref: No)					2.99e-04					0.003
	Yes	-0.23	(-0.36,-0.10)	0.06	-0.11		-0.20	(-0.34,-0.07)	0.07	-0.11	

Variable	Category	Secure Flourishing Index					Flourishing Index				
		Est	95% CI	SE	Est/SD	Global p-value	Est	95% CI	SE	Est/SD	Global p-value
Outsider growing up	(Ref: No)					0.038					0.057
	Yes	-0.14	(-0.27,-0.01)	0.07	-0.07		-0.14	(-0.27,0.00)	0.07	-0.07	
Self-rated health growing up	(Ref: Good)					1.85e-08					3.33e-07
	Excellent	0.13	(-0.00,0.26)	0.07	0.06		0.05	(-0.09,0.20)	0.07	0.03	
	Very good	0.10	(-0.00,0.20)	0.05	0.05		0.05	(-0.06,0.17)	0.06	0.03	
	Fair	-0.19	(-0.31,-0.06)	0.06	-0.09		-0.25	(-0.38,-0.13)	0.07	-0.13	
	Poor	-0.42	(-0.65,-0.20)	0.11	-0.21		-0.41	(-0.65,-0.17)	0.12	-0.22	
Immigration status	(Ref: Born in this country)					0.052					0.011
	Born in another country	-0.42	(-0.84,0.01)	0.22	-0.20		-0.52	(-0.93,-0.12)	0.21	-0.28	
Age 12 religious service attendance	(Ref: Never)					0.261					0.101
	At least 1/week	0.12	(-0.01,0.26)	0.07	0.06		0.16	(0.02,0.30)	0.07	0.08	
	1-3/month	0.11	(-0.03,0.26)	0.07	0.06		0.14	(-0.01,0.30)	0.08	0.08	
	< 1/month	0.07	(-0.08,0.21)	0.07	0.03		0.07	(-0.09,0.23)	0.08	0.04	
Year of birth	(Ref: 1998-2005; current age: 18-24)					< 2e-16					< 2e-16
	1993-1998; age 25-29	-0.22	(-0.34,-0.09)	0.06	-0.11		-0.26	(-0.39,-0.12)	0.07	-0.14	
	1983-1993; age 30-39	-0.43	(-0.54,-0.32)	0.06	-0.21		-0.47	(-0.59,-0.35)	0.06	-0.25	
	1973-1983; age 40-49	-0.51	(-0.64,-0.38)	0.07	-0.25		-0.58	(-0.72,-0.44)	0.07	-0.30	
	1963-1973; age 50-59	-0.58	(-0.74,-0.43)	0.08	-0.29		-0.70	(-0.86,-0.53)	0.08	-0.37	

Variable	Category	Secure Flourishing Index					Flourishing Index				
		Est	95% CI	SE	Est/SD	Global p-value	Est	95% CI	SE	Est/SD	Global p-value
	1953-1963; age 60-69	-0.64	(-0.81,-0.47)	0.09	-0.31		-0.79	(-0.97,-0.60)	0.09	-0.41	
	1943-1953; age 70-79	-0.71	(-0.99,-0.44)	0.14	-0.35		-0.86	(-1.16,-0.57)	0.15	-0.45	
	1943 or earlier; age 80+	-0.40	(-1.18,0.38)	0.40	-0.20		-0.82	(-1.63,0.00)	0.42	-0.43	
Gender	(Ref: Male)					0.863					0.021
	Female	0.01	(-0.07,0.08)	0.04	0.00		0.10	(0.01,0.18)	0.04	0.05	
Religious affiliation	(Ref: Hinduism)					0.056					0.009
	Islam	-0.18	(-0.33,-0.03)	0.08	-0.09		-0.21	(-0.37,-0.05)	0.08	-0.11	
	Collapsed affiliations with prevalence<3 %	0.02	(-0.14,0.19)	0.08	0.01		0.12	(-0.05,0.30)	0.09	0.06	
Race/ethnicity	(Ref: Plurality group)					0.459					0.696
	Non-plurality groups	-0.04	(-0.13,0.06)	0.05	-0.02		-0.02	(-0.12,0.09)	0.05	-0.01	

Note. N=12765; p < .004 (Bonferroni corrected threshold); Est., estimated effect of childhood predictor on flourishing score; CI, confidence interval; SE, standard error of the estimated effect; Est/SD, a more standardized measure of effect size--estimated effect of flourishing divided by standard deviation of flourishing--leads to the interpretation, for those with the given status (e.g., those with a good/very good relationship with mother compared to those with bad/very bad) are 0.XX standard deviations higher/lower on flourishing; the Global p-value corresponds to the two-sided joint parameter Wald-type test of whether any of the levels' parameters are non-zero, for history of abuse, outsider, relationship wtih mother/father, this is test of whether the estimated effect is non-zero, for multiple-category predictors (age, health, financial status), this is a joint test of whether any of these effects are non-zero. Note the confidence interval of the effect estimate can contradict the reported global p-value (e.g., for the single-category effects of relationship with mother). In such cases, the reported confidence interval is more robust with corrected degrees of freedom from the pooling across multiple imputations, whereas the global p-value is based on a Wald-type test and is less robust to uncertainty attributable to multiple imputation.

Table S7d. Sensitivity to unmeasured confounding of childhood predictors in India (N=12765)

Variable	Category	Secure Flourishing Index		Flourishing Index	
		E-value for Estimate	E-value for 95% CI	E-value for Estimate	E-value for 95% CI
Relationship with mother	(Ref: Very bad/somewhat bad)				
	Very good/somewhat good	1.36	1.00	1.46	1.00
Relationship with father	(Ref: Very bad/somewhat bad)				
	Very good/somewhat good	1.15	1.00	1.18	1.00
Parent marital status	(Ref: Parents married)				
	Divorced	1.44	1.00	1.52	1.00
	Parents were never married	1.28	1.00	1.21	1.00
	One or both parents had died	1.28	1.00	1.24	1.00
Subjective financial status of family growing up	(Ref: Got by)				
	Lived comfortably	1.33	1.14	1.24	1.00
	Found it difficult	1.41	1.21	1.41	1.20
	Found it very difficult	1.68	1.49	1.74	1.54
Abuse	(Ref: No)				
	Yes	1.48	1.28	1.42	1.21
Outsider growing up	(Ref: No)				
	Yes	1.34	1.06	1.32	1.00
Self-rated health growing up	(Ref: Good)				
	Excellent	1.32	1.00	1.18	1.00
	Very good	1.27	1.00	1.18	1.00
	Fair	1.41	1.21	1.49	1.31
	Poor	1.75	1.44	1.69	1.38
Immigration status	(Ref: Born in this country)				
	Born in another country	1.74	1.00	1.84	1.29
Age 12 religious service attendance	(Ref: Never)				
	At least 1/week	1.32	1.00	1.35	1.09
	1-3/month	1.30	1.00	1.33	1.00
	< 1/month	1.21	1.00	1.21	1.00
Year of birth	(Ref: 1998-2005; current age: 18-24)				
	1993-1998; age 25-29	1.46	1.26	1.50	1.30
	1983-1993; age 30-39	1.75	1.60	1.77	1.61

Variable	Category	Secure Flourishing Index		Flourishing Index	
		E-value for Estimate	E-value for 95% CI	E-value for Estimate	E-value for 95% CI
	1973-1983; age 40-49	1.87	1.69	1.92	1.73
	1963-1973; age 50-59	1.97	1.76	2.08	1.86
	1953-1963; age 60-69	2.05	1.81	2.20	1.95
	1943-1953; age 70-79	2.16	1.77	2.31	1.90
	1943 or earlier; age 80+	1.72	1.00	2.24	1.00
Gender	(Ref: Male)				
	Female	1.06	1.00	1.26	1.09
Religious affiliation	(Ref: Hinduism)				
	Islam	1.40	1.13	1.43	1.18
	Collapsed affiliations with prevalence<3%	1.11	1.00	1.30	1.00
Race/ethnicity	(Ref: Plurality group)				
	Non-plurality groups	1.15	1.00	1.10	1.00

Table S8a. Nationally representative descriptive statistics for Indonesia

Characteristic	N = 6,992[1]
**Age group**	
1998-2005; age 18-24	1,216 (17%)
1993-1998; age 25-29	849 (12%)
1983-1993; age 30-39	1,591 (23%)
1973-1983; age 40-49	1,576 (23%)
1963-1973; age 50-59	1,169 (17%)
1953-1963; age 60-69	490 (7.0%)
1943-1953; age 70-79	83 (1.2%)
1943 or earlier; age 80+	17 (0.2%)
(Missing)	0 (0%)
**Gender**	
Male	3,461 (50%)
Female	3,513 (50%)
Other	7 (<0.1%)
(Missing)	11 (0.2%)
**Race/Ethnicity**	
Bali	69 (1.0%)
Banjar/Melayu Banjar	320 (4.6%)
Batak	165 (2.4%)
Betawi	251 (3.6%)
Bugis	243 (3.5%)
Jawa	2,846 (41%)
Madura	262 (3.7%)
Makasar	91 (1.3%)
Minangkabau	273 (3.9%)
Other	1,262 (18%)
Sunda/Parahyangan	1,172 (17%)
(Missing)	38 (0.5%)
**Marital status**	
Married	4,846 (69%)
Separated	81 (1.2%)
Divorced	196 (2.8%)
Widowed	425 (6.1%)
Single, never married	1,381 (20%)
Domestic Partner	18 (0.3%)
(Missing)	45 (0.6%)
**Employment**	
Employed for an employer	1,323 (19%)
Self-employed	2,187 (31%)
Retired	78 (1.1%)
Student	272 (3.9%)
Homemaker	2,138 (31%)
Unemployed and looking for a job	529 (7.6%)
None of these/Other	448 (6.4%)
(Missing)	18 (0.3%)
**Religious service attendance**	
More than 1/week	2,667 (38%)
1/week	2,529 (36%)
1-3/month	786 (11%)
A few times a year	659 (9.4%)
Never	332 (4.8%)
(Missing)	18 (0.3%)
**Education**	
Up to 8 years	3,079 (44%)
9-15 years	3,491 (50%)
16+ years	419 (6.0%)
(Missing)	2 (<0.1%)
**Immigration status**	

Characteristic	N = 6,992[1]
Born in this country	6,958 (100%)
Born in another country	34 (0.5%)
(Missing)	0 (0%)
**Religious affiliation as an adult (now)**	
Christianity	504 (7.2%)
Islam	6,406 (92%)
Hinduism	73 (1.0%)
Buddhism	3 (<0.1%)
Judaism	0 (0%)
Sikhism	0 (0%)
Baha'i	0 (0%)
Jainism	0 (0%)
Shinto	0 (0%)
Taoism	1 (<0.1%)
Confucianism	0 (0%)
Primal, Animist, or Folk religion	0 (0%)
Spiritism	0 (0%)
Umbanda, Candomble, and other African-derived religions	0 (0%)
Chinese folk/traditional religion	0 (0%)
Some other religion	1 (<0.1%)
No religion/Atheist/Agnostic	0 (0%)
(Missing)	4 (<0.1%)
**Parent marital status**	
Parents married	5,557 (79%)
Divorced	448 (6.4%)
Parents were never married	47 (0.7%)
One or both parents had died	735 (11%)
(Missing)	205 (2.9%)
**Age 12 religious service attendance**	
At least 1/week	5,363 (77%)
1-3/month	973 (14%)
<1/month	329 (4.7%)
Never	275 (3.9%)
(Missing)	51 (0.7%)
**Relationship with mother**	
Very good	6,238 (89%)
Somewhat good	583 (8.3%)
Somewhat bad	50 (0.7%)
Very bad	26 (0.4%)
Does not apply	68 (1.0%)
(Missing)	27 (0.4%)
**Relationship with father**	
Very good	6,067 (87%)
Somewhat good	628 (9.0%)
Somewhat bad	68 (1.0%)
Very bad	52 (0.7%)
Does not apply	115 (1.6%)
(Missing)	61 (0.9%)
**Outsider growing up**	
Yes	343 (4.9%)
No	6,639 (95%)
(Missing)	10 (0.1%)
**Self-reported history of abuse**	
Yes	486 (6.9%)
No	6,427 (92%)
(Missing)	79 (1.1%)
**Self-rated health growing up**	
Excellent	1,246 (18%)
Very good	1,968 (28%)

Characteristic	N = 6,992[1]
Good	2,490 (36%)
Fair	1,233 (18%)
Poor	55 (0.8%)
(Missing)	1 (<0.1%)
**Subjective financial status of family growing up**	
Lived comfortably	3,408 (49%)
Got by	2,955 (42%)
Found it difficult	439 (6.3%)
Found it very difficult	181 (2.6%)
(Missing)	9 (0.1%)
**Religious affiliation at age 12**	
Christianity	528 (7.6%)
Islam	6,373 (91%)
Hinduism	75 (1.1%)
Buddhism	5 (<0.1%)
Judaism	0 (0%)
Sikhism	0 (0%)
Baha'i	0 (0%)
Jainism	1 (<0.1%)
Shinto	0 (0%)
Taoism	0 (<0.1%)
Confucianism	1 (<0.1%)
Primal, Animist, or Folk religion	1 (<0.1%)
Spiritism	0 (0%)
Umbanda, Candomble, and other African-derived religions	0 (0%)
Chinese folk/traditional religion	0 (0%)
Some other religion	0 (0%)
No religion/Atheist/Agnostic	2 (<0.1%)
(Missing)	8 (0.1%)

[1]n (%)

Table S8b. Means by demographic category for Indonesia (N=6992)

Variable	Category	Secure Flourishing Index				Flourishing Index			
		Mean	95% CI	SE	Global p-value	Mean	95% CI	SE	Global p-value
Age group	18-24	8.31	(8.21,8.40)	0.05	1.27e-07	7.97	(7.88,8.06)	0.05	0.006
	25-29	8.56	(8.45,8.67)	0.06		8.15	(8.04,8.26)	0.05	
	30-39	8.61	(8.53,8.69)	0.04		8.19	(8.11,8.27)	0.04	
	40-49	8.56	(8.47,8.65)	0.05		8.15	(8.06,8.24)	0.05	
	50-59	8.33	(8.21,8.45)	0.06		8.04	(7.91,8.16)	0.06	
	60-69	8.28	(8.08,8.47)	0.10		8.04	(7.82,8.25)	0.11	
	70-79	8.37	(7.96,8.78)	0.21		8.27	(7.85,8.68)	0.21	
	80 or older	7.44	(5.87,9.02)	0.67		7.28	(5.78,8.79)	0.63	
Gender	Female	8.50	(8.45,8.56)	0.03	0.008	8.12	(8.06,8.18)	0.03	0.055
	Male	8.43	(8.36,8.50)	0.04		8.08	(8.01,8.16)	0.04	
	Other	6.26	(2.97,9.56)	0.85		5.92	(2.18,9.67)	0.97	
Marital status	Divorced	8.30	(8.01,8.60)	0.15	8.66e-07	7.87	(7.56,8.18)	0.16	5.08e-04
	Domestic partner	8.58	(7.61,9.55)	0.43		8.12	(7.37,8.88)	0.34	
	Married	8.55	(8.49,8.60)	0.03		8.16	(8.11,8.22)	0.03	
	Separated	8.14	(7.60,8.68)	0.27		7.87	(7.39,8.36)	0.24	
	Single/Never been married	8.27	(8.17,8.36)	0.05		7.95	(7.85,8.05)	0.05	
	Widowed	8.32	(8.14,8.51)	0.10		8.00	(7.80,8.19)	0.10	
Employment	Employed for an employer	8.39	(8.27,8.50)	0.06	5.46e-07	8.04	(7.92,8.16)	0.06	2.54e-06
	Homemaker	8.57	(8.50,8.65)	0.04		8.15	(8.07,8.23)	0.04	
	None of these/Other	8.34	(8.16,8.52)	0.09		8.01	(7.83,8.20)	0.09	
	Retired	8.35	(8.08,8.62)	0.14		8.20	(7.93,8.46)	0.13	
	Self-employed	8.56	(8.48,8.63)	0.04		8.22	(8.15,8.30)	0.04	
	Student	8.16	(7.97,8.35)	0.10		7.84	(7.65,8.03)	0.10	
	Unemployed and looking for a job	8.13	(7.94,8.32)	0.10		7.74	(7.55,7.94)	0.10	
Religious service attendance	A few times a year	8.19	(8.08,8.30)	0.06	8.04e-07	7.83	(7.72,7.94)	0.06	1.58e-07
	More than once a week	8.55	(8.47,8.63)	0.04		8.22	(8.14,8.31)	0.04	

Variable	Category	Secure Flourishing Index				Flourishing Index			
		Mean	95% CI	SE	Global p-value	Mean	95% CI	SE	Global p-value
	Never	8.36	(8.13,8.59)	0.12		8.02	(7.81,8.24)	0.11	
	Once a week	8.50	(8.43,8.58)	0.04		8.09	(8.01,8.16)	0.04	
	One to three times a month	8.35	(8.24,8.46)	0.06		7.98	(7.87,8.09)	0.06	
Education	Up to 8	8.54	(8.45,8.63)	0.04	0.010	8.13	(8.04,8.21)	0.04	0.434
	9 to 15	8.34	(8.23,8.44)	0.05		8.12	(8.02,8.22)	0.05	
	16+	8.42	(8.36,8.47)	0.03		8.07	(8.02,8.13)	0.03	
Immigration status	Born in another country	8.27	(7.43,9.10)	0.41	0.626	7.83	(7.06,8.61)	0.38	0.486
	Born in this country	8.47	(8.42,8.52)	0.03		8.10	(8.05,8.15)	0.03	
Religious affiliation	Buddhism	7.12	*	*	6.90e-10	6.54	*	*	7.28e-14
	Christianity	8.45	(8.24,8.66)	0.11		8.07	(7.88,8.25)	0.09	
	Hinduism	8.38	(8.01,8.74)	0.18		7.88	(7.63,8.14)	0.13	
	Islam	8.47	(8.42,8.52)	0.03		8.11	(8.05,8.16)	0.03	
	Some other religion	7.10	*	*		6.92	*	*	
	Taoism	6.60	*	*		6.50	*	*	

Note. N=6992; $p < .007 = 0.05/7$ (Bonferroni corrected p-value significance threshold); Mean, estimated group mean; CI, confidence interval for the mean within group; SE, complex survey adjusted standard error or the mean; Global p-value, two-tailed Wald-type test of whether there is evidence of any differences in mean scores among groups of a demographic characteristic. *Estimate is not reported due to multiple-imputation and complex survey adjusted degrees of freedom was less than 1.00 leading to insufficient information to provide an estimate of the uncertainty in the estimate. These groups are removed when estimating the global test of mean differences.

Table S8c. Childhood predictors regression analysis results for Indonesia (N=6992)

Variable	Category	Secure Flourishing Index					Flourishing Index				
		Est	95% CI	SE	Est/SD	Global p-value	Est	95% CI	SE	Est/SD	Global p-value
Relationship with mother	(Ref: Very bad/somewhat bad)					7.65e-04					0.001
	Very good/somewhat good	0.55	(0.23,0.88)	0.17	0.41		0.53	(0.20,0.86)	0.17	0.39	
Relationship with father	(Ref: Very bad/somewhat bad)					0.467					0.231
	Very good/somewhat good	0.10	(-0.17,0.38)	0.14	0.08		0.17	(-0.11,0.45)	0.14	0.13	
Parent marital status	(Ref: Parents married)					0.693					0.621
	Divorced	-0.10	(-0.31,0.11)	0.11	-0.07		-0.05	(-0.24,0.15)	0.10	-0.03	
	Parents were never married	0.17	(-0.37,0.71)	0.27	0.13		0.16	(-0.38,0.69)	0.27	0.12	
	One or both parents had died	-0.01	(-0.17,0.14)	0.08	-0.01		0.09	(-0.07,0.24)	0.08	0.06	
Subjective financial status of family growing up	(Ref: Got by)					< 2e-16					7.85e-11
	Lived comfortably	0.30	(0.21,0.39)	0.04	0.22		0.23	(0.14,0.31)	0.04	0.17	
	Found it difficult	-0.31	(-0.52,-0.10)	0.11	-0.23		-0.28	(-0.49,-0.07)	0.11	-0.21	
	Found it very difficult	-0.40	(-0.76,-0.05)	0.18	-0.30		-0.31	(-0.67,0.05)	0.18	-0.23	
Abuse	(Ref: No)					7.66e-07					5.47e-06
	Yes	-0.51	(-0.71,-0.31)	0.10	-0.38		-0.48	(-0.69,-0.27)	0.11	-0.36	

Variable	Category	Secure Flourishing Index Est	95% CI	SE	Est/SD	Global p-value	Flourishing Index Est	95% CI	SE	Est/SD	Global p-value
Outsider growing up	(Ref: No)					0.125					0.114
	Yes	-0.17	(-0.39,0.05)	0.11	-0.13		-0.19	(-0.41,0.04)	0.12	-0.14	
Self-rated health growing up	(Ref: Good)					1.76e-06					1.12e-06
	Excellent	0.24	(0.12,0.36)	0.06	0.18		0.29	(0.18,0.41)	0.06	0.22	
	Very good	0.10	(0.00,0.19)	0.05	0.07		0.14	(0.05,0.23)	0.05	0.10	
	Fair	-0.12	(-0.24,-0.01)	0.06	-0.09		-0.05	(-0.16,0.06)	0.06	-0.04	
	Poor	-0.21	(-0.71,0.30)	0.26	-0.15		-0.10	(-0.66,0.47)	0.29	-0.07	
Immigration status	(Ref: Born in this country)					0.610					0.681
	Born in another country	-0.21	(-1.03,0.61)	0.42	-0.16		-0.18	(-1.06,0.70)	0.45	-0.14	
Age 12 religious service attendance	(Ref: Never)					0.146					0.259
	At least 1/week	0.21	(0.00,0.41)	0.10	0.15		0.14	(-0.07,0.35)	0.11	0.10	
	1-3/month	0.13	(-0.11,0.37)	0.12	0.10		0.04	(-0.20,0.29)	0.13	0.03	
	< 1/month	0.16	(-0.12,0.45)	0.14	0.12		0.04	(-0.24,0.33)	0.14	0.03	
Year of birth	(Ref: 1998-2005; current age: 18-24)					6.21e-05					5.45e-08
	1993-1998; age 25-29	0.18	(0.05,0.31)	0.07	0.14		0.25	(0.12,0.39)	0.07	0.19	
	1983-1993; age 30-39	0.28	(0.17,0.40)	0.06	0.21		0.36	(0.24,0.47)	0.06	0.26	
	1973-1983; age 40-49	0.27	(0.16,0.39)	0.06	0.20		0.32	(0.20,0.45)	0.06	0.24	
	1963-1973; age 50-59	0.21	(0.06,0.36)	0.08	0.15		0.14	(-0.01,0.29)	0.08	0.10	

Variable	Category	Secure Flourishing Index Est	95% CI	SE	Est/SD	Global p-value	Flourishing Index Est	95% CI	SE	Est/SD	Global p-value
	1953-1963; age 60-69	0.25	(0.02,0.47)	0.11	0.18		0.12	(-0.10,0.33)	0.11	0.09	
	1943-1953; age 70-79	0.41	(0.01,0.82)	0.21	0.31		0.16	(-0.24,0.56)	0.20	0.12	
	1943 or earlier; age 80+	-0.25	(-1.49,1.00)	0.63	-0.18		-0.48	(-1.76,0.80)	0.65	-0.35	
Gender	(Ref: Male)					0.104					0.022
	Female	0.01	(-0.06,0.09)	0.04	0.01		0.05	(-0.03,0.13)	0.04	0.04	
	Other	-2.20	(-4.26,-0.13)	1.05	-1.63		-2.16	(-3.92,-0.39)	0.90	-1.60	
Religious affiliation	(Ref: Islam)					0.594					0.949
	Christianity	0.06	(-0.11,0.22)	0.09	0.04		0.03	(-0.15,0.21)	0.09	0.02	
	Collapsed affiliations with prevalence<3 %	-0.12	(-0.42,0.18)	0.15	-0.09		-0.03	(-0.42,0.35)	0.20	-0.03	
Race/ethnicity	(Ref: Plurality group)					0.155					0.454
	Non-plurality groups	-0.07	(-0.17,0.03)	0.05	-0.05		0.04	(-0.06,0.14)	0.05	0.03	

Note. N=6992; p < .004 (Bonferroni corrected threshold); Est., estimated effect of childhood predictor on flourishing score; CI, confidence interval; SE, standard error of the estimated effect; Est/SD, a more standardized measure of effect size--estimated effect of flourishing divided by standard deviation of flourishing--leads to the interpretation, for those with the given status (e.g., those with a good/very good relationship with mother compared to those with bad/very bad) are 0.XX standard deviations higher/lower on flourishing; the Global p-value corresponds to the two-sided joint parameter Wald-type test of whether any of the levels' parameters are non-zero, for history of abuse, outsider, relationship wtih mother/father, this is test of whether the estimated effect is non-zero, for multiple-category predictors (age, health, financial status), this is a joint test of whether any of these effects are non-zero. Note the confidence interval of the effect estimate can contradict the reported global p-value (e.g., for the single-category effects of relationship with mother). In such cases, the reported confidence interval is more robust with corrected degrees of freedom from the pooling across multiple imputations, whereas the global p-value is based on a Wald-type test and is less robust to uncertainty attributable to multiple imputation.

Table S8d. Sensitivity to unmeasured confounding of childhood predictors in Indonesia (N=6992)

Variable	Category	Secure Flourishing Index		Flourishing Index	
		E-value for Estimate	E-value for 95% CI	E-value for Estimate	E-value for 95% CI
Relationship with mother	(Ref: Very bad/somewhat bad)				
	Very good/somewhat good	2.26	1.61	2.22	1.56
Relationship with father	(Ref: Very bad/somewhat bad)				
	Very good/somewhat good	1.35	1.00	1.50	1.00
Parent marital status	(Ref: Parents married)				
	Divorced	1.34	1.00	1.21	1.00
	Parents were never married	1.50	1.00	1.46	1.00
	One or both parents had died	1.11	1.00	1.31	1.00
Subjective financial status of family growing up	(Ref: Got by)				
	Lived comfortably	1.75	1.57	1.61	1.44
	Found it difficult	1.77	1.34	1.71	1.28
	Found it very difficult	1.95	1.22	1.77	1.00
Abuse	(Ref: No)				
	Yes	2.17	1.77	2.11	1.70
Outsider growing up	(Ref: No)				
	Yes	1.50	1.00	1.52	1.00
Self-rated health growing up	(Ref: Good)				
	Excellent	1.64	1.40	1.74	1.51
	Very good	1.33	1.05	1.43	1.21
	Fair	1.40	1.10	1.23	1.00
	Poor	1.56	1.00	1.33	1.00
Immigration status	(Ref: Born in this country)				
	Born in another country	1.58	1.00	1.52	1.00
Age 12 religious service attendance	(Ref: Never)				
	At least 1/week	1.57	1.04	1.42	1.00
	1-3/month	1.41	1.00	1.21	1.00
	< 1/month	1.48	1.00	1.20	1.00
Year of birth	(Ref: 1998-2005; current age: 18-24)				
	1993-1998; age 25-29	1.52	1.23	1.65	1.39
	1983-1993; age 30-39	1.72	1.49	1.86	1.63

Variable	Category	Secure Flourishing Index		Flourishing Index	
		E-value for Estimate	E-value for 95% CI	E-value for Estimate	E-value for 95% CI
	1973-1983; age 40-49	1.70	1.46	1.80	1.56
	1963-1973; age 50-59	1.56	1.24	1.43	1.00
	1953-1963; age 60-69	1.64	1.14	1.38	1.00
	1943-1953; age 70-79	1.97	1.09	1.47	1.00
	1943 or earlier; age 80+	1.65	1.00	2.11	1.00
Gender	(Ref: Male)				
	Female	1.10	1.00	1.23	1.00
	Other	8.26	1.42	8.04	1.93
Religious affiliation	(Ref: Islam)				
	Christianity	1.24	1.00	1.15	1.00
	Collapsed affiliations with prevalence<3%	1.38	1.00	1.18	1.00
Race/ethnicity	(Ref: Plurality group)				
	Non-plurality groups	1.28	1.00	1.19	1.00

Table S9a. Nationally representative descriptive statistics for Israel

Characteristic	N = 3,669[1]
**Age group**	
1998-2005; age 18-24	553 (15%)
1993-1998; age 25-29	407 (11%)
1983-1993; age 30-39	666 (18%)
1973-1983; age 40-49	616 (17%)
1963-1973; age 50-59	542 (15%)
1953-1963; age 60-69	469 (13%)
1943-1953; age 70-79	336 (9.2%)
1943 or earlier; age 80+	79 (2.2%)
(Missing)	0 (0%)
**Gender**	
Male	1,791 (49%)
Female	1,872 (51%)
Other	0 (<0.1%)
(Missing)	6 (0.2%)
**Race/Ethnicity**	
Arab	674 (18%)
Jewish	2,926 (80%)
Other	39 (1.1%)
(Missing)	30 (0.8%)
**Marital status**	
Married	2,056 (56%)
Separated	48 (1.3%)
Divorced	258 (7.0%)
Widowed	212 (5.8%)
Single, never married	834 (23%)
Domestic Partner	193 (5.3%)
(Missing)	69 (1.9%)
**Employment**	
Employed for an employer	1,793 (49%)
Self-employed	424 (12%)
Retired	576 (16%)
Student	388 (11%)
Homemaker	211 (5.7%)
Unemployed and looking for a job	148 (4.0%)
None of these/Other	118 (3.2%)
(Missing)	10 (0.3%)
**Religious service attendance**	
More than 1/week	649 (18%)
1/week	495 (14%)
1-3/month	374 (10%)
A few times a year	1,014 (28%)
Never	1,122 (31%)
(Missing)	14 (0.4%)
**Education**	
Up to 8 years	224 (6.1%)
9-15 years	1,517 (41%)
16+ years	1,926 (52%)
(Missing)	2 (<0.1%)
**Immigration status**	
Born in this country	2,796 (76%)
Born in another country	868 (24%)
(Missing)	5 (0.1%)
**Religious affiliation as an adult (now)**	
Christianity	39 (1.1%)
Islam	656 (18%)
Hinduism	0 (0%)
Buddhism	0 (0%)

**Characteristic**	**N = 3,669**[1]
Judaism	2,897 (79%)
Sikhism	0 (0%)
Baha'i	2 (<0.1%)
Jainism	0 (0%)
Shinto	0 (0%)
Taoism	1 (<0.1%)
Confucianism	0 (0%)
Primal, Animist, or Folk religion	1 (<0.1%)
Spiritism	0 (0%)
Umbanda, Candomble, and other African-derived religions	0 (0%)
Chinese folk/traditional religion	0 (0%)
Some other religion	5 (0.1%)
No religion/Atheist/Agnostic	64 (1.7%)
(Missing)	4 (0.1%)
**Parent marital status**	
Parents married	3,172 (86%)
Divorced	284 (7.8%)
Parents were never married	36 (1.0%)
One or both parents had died	130 (3.5%)
(Missing)	47 (1.3%)
**Age 12 religious service attendance**	
At least 1/week	867 (24%)
1-3/month	435 (12%)
<1/month	810 (22%)
Never	1,539 (42%)
(Missing)	17 (0.5%)
**Relationship with mother**	
Very good	2,686 (73%)
Somewhat good	793 (22%)
Somewhat bad	110 (3.0%)
Very bad	18 (0.5%)
Does not apply	45 (1.2%)
(Missing)	17 (0.5%)
**Relationship with father**	
Very good	2,290 (62%)
Somewhat good	912 (25%)
Somewhat bad	234 (6.4%)
Very bad	37 (1.0%)
Does not apply	171 (4.7%)
(Missing)	25 (0.7%)
**Outsider growing up**	
Yes	371 (10%)
No	3,228 (88%)
(Missing)	70 (1.9%)
**Self-reported history of abuse**	
Yes	0 (0%)
No	0 (0%)
(Missing)	3,669 (100%)
**Self-rated health growing up**	
Excellent	1,785 (49%)
Very good	1,284 (35%)
Good	480 (13%)
Fair	105 (2.9%)
Poor	6 (0.2%)
(Missing)	8 (0.2%)
**Subjective financial status of family growing up**	
Lived comfortably	923 (25%)
Got by	1,822 (50%)
Found it difficult	667 (18%)

**Characteristic**	**N = 3,669**[1]
Found it very difficult	239 (6.5%)
(Missing)	17 (0.5%)
**Religious affiliation at age 12**	
Christianity	60 (1.6%)
Islam	647 (18%)
Hinduism	0 (0%)
Buddhism	0 (0%)
Judaism	2,873 (78%)
Sikhism	1 (<0.1%)
Baha'i	1 (<0.1%)
Jainism	0 (0%)
Shinto	0 (0%)
Taoism	0 (0%)
Confucianism	0 (0%)
Primal, Animist, or Folk religion	3 (<0.1%)
Spiritism	0 (0%)
Umbanda, Candomble, and other African-derived religions	0 (0%)
Chinese folk/traditional religion	0 (0%)
Some other religion	5 (0.1%)
No religion/Atheist/Agnostic	69 (1.9%)
(Missing)	10 (0.3%)

[1]n (%)

Table S9b. Means by demographic category for Israel (N=3669)

Variable	Category	Secure Flourishing Index				Flourishing Index			
		Mean	95% CI	SE	Global p-value	Mean	95% CI	SE	Global p-value
Age group	18-24	8.24	(8.08,8.39)	0.08	3.55e-06	8.08	(7.91,8.25)	0.09	7.56e-05
	25-29	8.20	(8.03,8.36)	0.08		8.02	(7.84,8.19)	0.09	
	30-39	8.07	(7.92,8.22)	0.08		7.88	(7.72,8.04)	0.08	
	40-49	8.06	(7.91,8.21)	0.08		7.90	(7.74,8.06)	0.08	
	50-59	7.96	(7.78,8.13)	0.09		7.83	(7.65,8.01)	0.09	
	60-69	8.00	(7.78,8.21)	0.11		7.92	(7.72,8.13)	0.10	
	70-79	7.42	(7.16,7.68)	0.13		7.40	(7.15,7.66)	0.13	
	80 or older	7.11	(6.58,7.64)	0.27		7.13	(6.63,7.63)	0.25	
Gender	Female	7.93	(7.80,8.07)	0.07	< 2e-16	7.79	(7.66,7.93)	0.07	< 2e-16
	Male	8.07	(7.94,8.20)	0.06		7.94	(7.81,8.08)	0.07	
	Other	7.20	*	*		7.00	*	*	
Marital status	Divorced	7.32	(7.05,7.59)	0.14	1.33e-15	7.14	(6.87,7.40)	0.14	4.44e-16
	Domestic partner	7.81	(7.59,8.03)	0.11		7.60	(7.36,7.84)	0.12	
	Married	8.19	(8.07,8.31)	0.06		8.06	(7.93,8.19)	0.06	
	Separated	7.52	(7.18,7.85)	0.16		7.29	(6.97,7.62)	0.16	
	Single/Never been married	8.02	(7.87,8.17)	0.08		7.89	(7.74,8.04)	0.08	
	Widowed	7.26	(6.92,7.59)	0.17		7.19	(6.88,7.50)	0.16	
Employment	Employed for an employer	8.13	(8.01,8.25)	0.06	1.65e-08	7.96	(7.84,8.09)	0.06	3.01e-07
	Homemaker	7.61	(7.34,7.89)	0.14		7.51	(7.24,7.78)	0.14	
	None of these/Other	8.01	(7.75,8.28)	0.13		7.95	(7.69,8.21)	0.13	
	Retired	7.61	(7.35,7.87)	0.13		7.60	(7.35,7.85)	0.13	
	Self-employed	8.02	(7.82,8.23)	0.10		7.85	(7.63,8.07)	0.11	
	Student	8.40	(8.22,8.58)	0.09		8.25	(8.04,8.45)	0.10	
	Unemployed and looking for a job	7.44	(7.12,7.77)	0.16		7.25	(6.92,7.58)	0.17	
Religious service attendance	A few times a year	7.82	(7.63,8.01)	0.10	< 2e-16	7.67	(7.48,7.85)	0.10	< 2e-16
	More than once a week	8.68	(8.54,8.82)	0.07		8.59	(8.44,8.73)	0.07	

		Secure Flourishing Index				Flourishing Index			
Variable	Category	Mean	95% CI	SE	Global p-value	Mean	95% CI	SE	Global p-value
	Never	7.70	(7.56,7.85)	0.07		7.58	(7.42,7.74)	0.08	
	Once a week	8.16	(7.97,8.35)	0.10		8.02	(7.82,8.21)	0.10	
	One to three times a month	7.98	(7.78,8.19)	0.10		7.83	(7.62,8.03)	0.10	
Education	Up to 8	7.32	(6.98,7.66)	0.17	1.47e-04	7.21	(6.88,7.54)	0.17	1.37e-04
	9 to 15	8.06	(7.93,8.19)	0.07		7.92	(7.78,8.05)	0.07	
	16+	8.02	(7.89,8.16)	0.07		7.90	(7.76,8.04)	0.07	
Immigration status	Born in another country	7.60	(7.40,7.80)	0.10	3.65e-08	7.45	(7.25,7.65)	0.10	1.33e-08
	Born in this country	8.13	(8.02,8.24)	0.06		8.00	(7.88,8.11)	0.06	
Religious affiliation	Baha'i	9.85	*	*	< 2e-16	9.42	*	*	< 2e-16
	Christianity	7.42	(6.99,7.84)	0.21		7.15	(6.74,7.55)	0.20	
	Islam	7.52	(7.33,7.71)	0.10		7.34	(7.16,7.53)	0.09	
	Judaism	8.12	(7.99,8.26)	0.07		8.00	(7.86,8.14)	0.07	
	No religion/Atheist/ Agnostic	7.65	(7.23,8.07)	0.21		7.64	(7.19,8.10)	0.23	
	Primal, Animist, or Folk religion	8.30	*	*		8.42	*	*	
	Some other religion	7.98	(6.48,9.48)	0.75		7.89	(6.60,9.19)	0.65	
	Taoism	7.30	*	*		6.08	*	*	

Note. N=3669; p < .007 = 0.05/7 (Bonferroni corrected p-value significance threshold); Mean, estimated group mean; CI, confidence interval for the mean within group; SE, complex survey adjusted standard error or the mean; Global p-value, two-tailed Wald-type test of whether there is evidence of any differences in mean scores among groups of a demographic characteristic. *Estimate is not reported due to multiple-imputation and complex survey adjusted degrees of freedom was less than 1.00 leading to insufficient information to provide an estimate of the uncertainty in the estimate. These groups are removed when estimating the global test of mean differences.

Table S9c. Childhood predictors regression analysis results for Israel (N=3669)

		Secure Flourishing Index					Flourishing Index				
Variable	Category	Est	95% CI	SE	Est/SD	Global p-value	Est	95% CI	SE	Est/SD	Global p-value
Relationship with mother	(Ref: Very bad/somewhat bad)					0.315					0.300
	Very good/somewhat good	-0.14	(-0.44,0.15)	0.15	-0.11		-0.14	(-0.43,0.14)	0.14	-0.10	
Relationship with father	(Ref: Very bad/somewhat bad)					0.732					0.797
	Very good/somewhat good	-0.04	(-0.27,0.20)	0.12	-0.03		-0.02	(-0.26,0.22)	0.12	-0.02	
Parent marital status	(Ref: Parents married)					8.19e-05					7.22e-04
	Divorced	-0.45	(-0.68,-0.22)	0.12	-0.34		-0.42	(-0.65,-0.20)	0.11	-0.31	
	Parents were never married	-0.56	(-1.00,-0.12)	0.22	-0.42		-0.45	(-0.85,-0.06)	0.20	-0.33	
	One or both parents had died	-0.43	(-0.71,-0.16)	0.14	-0.32		-0.35	(-0.64,-0.06)	0.15	-0.25	
Subjective financial status of family growing up	(Ref: Got by)					0.025					0.053
	Lived comfortably	0.13	(-0.00,0.25)	0.06	0.09		0.06	(-0.06,0.17)	0.06	0.04	
	Found it difficult	0.10	(-0.02,0.23)	0.06	0.08		0.11	(-0.02,0.24)	0.07	0.08	
	Found it very difficult	-0.18	(-0.42,0.05)	0.12	-0.14		-0.21	(-0.45,0.04)	0.12	-0.15	
Outsider growing up	(Ref: No)					0.010					0.005
	Yes	-0.27	(-0.47,-0.07)	0.10	-0.20		-0.28	(-0.47,-0.08)	0.10	-0.20	

Variable	Category	Secure Flourishing Index					Flourishing Index				
		Est	95% CI	SE	Est/SD	Global p-value	Est	95% CI	SE	Est/SD	Global p-value
Self-rated health growing up	(Ref: Good)					3.94e-04					1.64e-04
	Excellent	0.42	(0.19,0.66)	0.12	0.32		0.42	(0.18,0.66)	0.12	0.31	
	Very good	0.40	(0.19,0.61)	0.11	0.30		0.43	(0.22,0.65)	0.11	0.32	
	Fair	-0.19	(-0.49,0.10)	0.15	-0.15		-0.16	(-0.43,0.12)	0.14	-0.12	
	Poor	0.72	(-1.37,2.81)	1.07	0.54		0.93	(-0.88,2.73)	0.92	0.68	
Immigration status	(Ref: Born in this country)					9.23e-05					0.001
	Born in another country	-0.37	(-0.55,-0.20)	0.09	-0.28		-0.30	(-0.47,-0.12)	0.09	-0.22	
Age 12 religious service attendance	(Ref: Never)					2.39e-04					6.76e-05
	At least 1/week	0.29	(0.10,0.48)	0.10	0.22		0.30	(0.12,0.47)	0.09	0.22	
	1-3/month	0.39	(0.19,0.58)	0.10	0.29		0.43	(0.23,0.63)	0.10	0.32	
	< 1/month	0.33	(0.17,0.49)	0.08	0.24		0.34	(0.18,0.49)	0.08	0.25	
Year of birth	(Ref: 1998-2005; current age: 18-24)					0.008					1.62e-04
	1993-1998; age 25-29	-0.08	(-0.24,0.09)	0.08	-0.06		-0.05	(-0.21,0.11)	0.08	-0.04	
	1983-1993; age 30-39	-0.12	(-0.30,0.05)	0.09	-0.09		-0.10	(-0.26,0.06)	0.08	-0.07	
	1973-1983; age 40-49	-0.09	(-0.30,0.12)	0.11	-0.07		-0.10	(-0.30,0.10)	0.10	-0.07	
	1963-1973; age 50-59	-0.13	(-0.32,0.07)	0.10	-0.10		-0.18	(-0.36,0.00)	0.09	-0.13	
	1953-1963; age 60-69	-0.03	(-0.24,0.18)	0.11	-0.02		-0.15	(-0.35,0.06)	0.11	-0.11	
	1943-1953; age 70-79	-0.48	(-0.73,-0.23)	0.13	-0.36		-0.68	(-0.93,-0.42)	0.13	-0.49	

Variable	Category	Secure Flourishing Index					Flourishing Index				
		Est	95% CI	SE	Est/SD	Global p-value	Est	95% CI	SE	Est/SD	Global p-value
	1943 or earlier; age 80+	-0.59	(-1.05,-0.14)	0.23	-0.44		-0.83	(-1.33,-0.34)	0.25	-0.61	
Gender	(Ref: Male)					7.68e-04					3.51e-04
	Female	-0.09	(-0.20,0.03)	0.06	-0.06		-0.07	(-0.18,0.04)	0.06	-0.05	
	Other	-0.68	(-1.02,-0.33)	0.18	-0.50		-0.71	(-1.05,-0.37)	0.17	-0.52	
Religious affiliation	(Ref: Judaism)					0.056					0.128
	Islam	-0.60	(-1.17,-0.03)	0.29	-0.45		-0.48	(-1.04,0.08)	0.28	-0.35	
	Collapsed affiliations with prevalence<3 %	-0.19	(-0.55,0.17)	0.18	-0.14		-0.12	(-0.48,0.24)	0.18	-0.09	
Race/ethnicit y	(Ref: Plurality group)					0.235					0.125
	Non-plurality groups	-0.22	(-0.74,0.29)	0.26	-0.17		-0.30	(-0.81,0.21)	0.25	-0.22	
Abuse	(Ref: No)										

Note. N=3669; p < .004 (Bonferroni corrected threshold); Est., estimated effect of childhood predictor on flourishing score; CI, confidence interval; SE, standard error of the estimated effect; Est/SD, a more standardized measure of effect size--estimated effect of flourishing divided by standard deviation of flourishing--leads to the interpretation, for those with the given status (e.g., those with a good/very good relationship with mother compared to those with bad/very bad) are 0.XX standard deviations higher/lower on flourishing; the Global p-value corresponds to the two-sided joint parameter Wald-type test of whether any of the levels' parameters are non-zero, for history of abuse, outsider, relationship wtih mother/father, this is test of whether the estimated effect is non-zero, for multiple-category predictors (age, health, financial status), this is a joint test of whether any of these effects are non-zero. Note the confidence interval of the effect estimate can contradict the reported global p-value (e.g., for the single-category effects of relationship with mother). In such cases, the reported confidence interval is more robust with corrected degrees of freedom from the pooling across multiple imputations, whereas the global p-value is based on a Wald-type test and is less robust to uncertainty attributable to multiple imputation.

Table S9d. Sensitivity to unmeasured confounding of childhood predictors in Israel (N=3669)

		Secure Flourishing Index		Flourishing Index	
Variable	Category	E-value for Estimate	E-value for 95% CI	E-value for Estimate	E-value for 95% CI
Relationship with mother	(Ref: Very bad/somewhat bad)				
	Very good/somewhat good	1.43	1.00	1.44	1.00
Relationship with father	(Ref: Very bad/somewhat bad)				
	Very good/somewhat good	1.18	1.00	1.14	1.00
Parent marital status	(Ref: Parents married)				
	Divorced	2.05	1.60	2.00	1.55
	Parents were never married	2.26	1.38	2.06	1.24
	One or both parents had died	2.00	1.47	1.85	1.24
Subjective financial status of family growing up	(Ref: Got by)				
	Lived comfortably	1.40	1.00	1.24	1.00
	Found it difficult	1.34	1.00	1.36	1.00
	Found it very difficult	1.51	1.00	1.57	1.00
Outsider growing up	(Ref: No)				
	Yes	1.68	1.26	1.71	1.31
Self-rated health growing up	(Ref: Good)				
	Excellent	1.99	1.53	2.00	1.52
	Very good	1.95	1.54	2.02	1.59
	Fair	1.54	1.00	1.47	1.00
	Poor	2.62	1.00	3.16	1.00
Immigration status	(Ref: Born in this country)				
	Born in another country	1.88	1.54	1.75	1.40
Age 12 religious service attendance	(Ref: Never)				
	At least 1/week	1.72	1.35	1.75	1.40
	1-3/month	1.92	1.54	2.02	1.62
	< 1/month	1.80	1.49	1.82	1.52
Year of birth	(Ref: 1998-2005; current age: 18-24)				
	1993-1998; age 25-29	1.29	1.00	1.23	1.00
	1983-1993; age 30-39	1.39	1.00	1.34	1.00
	1973-1983; age 40-49	1.32	1.00	1.35	1.00
	1963-1973; age 50-59	1.40	1.00	1.52	1.00

Variable	Category	Secure Flourishing Index		Flourishing Index	
		E-value for Estimate	E-value for 95% CI	E-value for Estimate	E-value for 95% CI
	1953-1963; age 60-69	1.17	1.00	1.45	1.00
	1943-1953; age 70-79	2.09	1.60	2.54	1.99
	1943 or earlier; age 80+	2.34	1.43	2.92	1.84
Gender	(Ref: Male)				
	Female	1.31	1.00	1.28	1.00
	Other	2.51	1.81	2.63	1.90
Religious affiliation	(Ref: Judaism)				
	Islam	2.34	1.19	2.12	1.00
	Collapsed affiliations with prevalence<3%	1.53	1.00	1.39	1.00
Race/ethnicity	(Ref: Plurality group)				
	Non-plurality groups	1.60	1.00	1.75	1.00
Abuse	(Ref: No)				

Table S10a. Nationally representative descriptive statistics for Japan

Characteristic	N = 20,543[1]
**Age group**	
1998-2005; age 18-24	1,589 (7.7%)
1993-1998; age 25-29	806 (3.9%)
1983-1993; age 30-39	2,851 (14%)
1973-1983; age 40-49	3,363 (16%)
1963-1973; age 50-59	3,770 (18%)
1953-1963; age 60-69	4,118 (20%)
1943-1953; age 70-79	3,554 (17%)
1943 or earlier; age 80+	493 (2.4%)
(Missing)	0 (0%)
**Gender**	
Male	9,847 (48%)
Female	10,602 (52%)
Other	28 (0.1%)
(Missing)	66 (0.3%)
**Marital status**	
Married	11,837 (58%)
Separated	190 (0.9%)
Divorced	2,126 (10%)
Widowed	1,179 (5.7%)
Single, never married	5,004 (24%)
Domestic Partner	144 (0.7%)
(Missing)	64 (0.3%)
**Employment**	
Employed for an employer	10,853 (53%)
Self-employed	1,748 (8.5%)
Retired	2,535 (12%)
Student	491 (2.4%)
Homemaker	1,276 (6.2%)
Unemployed and looking for a job	622 (3.0%)
None of these/Other	2,983 (15%)
(Missing)	36 (0.2%)
**Religious service attendance**	
More than 1/week	316 (1.5%)
1/week	348 (1.7%)
1-3/month	862 (4.2%)
A few times a year	3,112 (15%)
Never	15,788 (77%)
(Missing)	117 (0.6%)
**Education**	
Up to 8 years	567 (2.8%)
9-15 years	14,893 (72%)
16+ years	5,083 (25%)
(Missing)	0 (0%)
**Immigration status**	
Born in this country	19,548 (95%)
Born in another country	158 (0.8%)
(Missing)	837 (4.1%)
**Religious affiliation as an adult (now)**	
Christianity	381 (1.9%)
Islam	10 (<0.1%)
Hinduism	5 (<0.1%)
Buddhism	6,709 (33%)
Judaism	10 (<0.1%)
Sikhism	6 (<0.1%)
Baha'i	2 (<0.1%)
Jainism	11 (<0.1%)
Shinto	469 (2.3%)

Characteristic	N = 20,543[1]
Taoism	7 (<0.1%)
Confucianism	17 (<0.1%)
Primal, Animist, or Folk religion	19 (<0.1%)
Spiritism	0 (0%)
Umbanda, Candomble, and other African-derived religions	0 (0%)
Chinese folk/traditional religion	0 (0%)
Some other religion	46 (0.2%)
No religion/Atheist/Agnostic	12,497 (61%)
(Missing)	355 (1.7%)
**Parent marital status**	
Parents married	17,713 (86%)
Divorced	1,127 (5.5%)
Parents were never married	591 (2.9%)
One or both parents had died	754 (3.7%)
(Missing)	359 (1.7%)
**Age 12 religious service attendance**	
At least 1/week	398 (1.9%)
1-3/month	883 (4.3%)
<1/month	5,023 (24%)
Never	14,117 (69%)
(Missing)	123 (0.6%)
**Relationship with mother**	
Very good	5,630 (27%)
Somewhat good	9,461 (46%)
Somewhat bad	2,750 (13%)
Very bad	799 (3.9%)
Does not apply	1,838 (8.9%)
(Missing)	66 (0.3%)
**Relationship with father**	
Very good	4,156 (20%)
Somewhat good	9,081 (44%)
Somewhat bad	3,446 (17%)
Very bad	1,223 (6.0%)
Does not apply	2,580 (13%)
(Missing)	57 (0.3%)
**Outsider growing up**	
Yes	1,963 (9.6%)
No	17,136 (83%)
(Missing)	1,444 (7.0%)
**Self-reported history of abuse**	
Yes	1,482 (7.2%)
No	18,964 (92%)
(Missing)	96 (0.5%)
**Self-rated health growing up**	
Excellent	2,711 (13%)
Very good	7,106 (35%)
Good	6,689 (33%)
Fair	3,199 (16%)
Poor	758 (3.7%)
(Missing)	80 (0.4%)
**Subjective financial status of family growing up**	
Lived comfortably	8,320 (41%)
Got by	8,799 (43%)
Found it difficult	2,398 (12%)
Found it very difficult	973 (4.7%)
(Missing)	52 (0.3%)
**Religious affiliation at age 12**	
Christianity	343 (1.7%)
Islam	7 (<0.1%)

Characteristic	N = 20,543[1]
Hinduism	4 (<0.1%)
Buddhism	6,536 (32%)
Judaism	0 (0%)
Sikhism	0 (0%)
Baha'i	7 (<0.1%)
Jainism	1 (<0.1%)
Shinto	382 (1.9%)
Taoism	14 (<0.1%)
Confucianism	25 (0.1%)
Primal, Animist, or Folk religion	13 (<0.1%)
Spiritism	0 (0%)
Umbanda, Candomble, and other African-derived religions	0 (0%)
Chinese folk/traditional religion	0 (0%)
Some other religion	46 (0.2%)
No religion/Atheist/Agnostic	12,950 (63%)
(Missing)	215 (1.0%)

[1] n (%)

Table S10b. Means by demographic category for Japan (N=20543)

Variable	Category	Secure Flourishing Index				Flourishing Index			
		Mean	95% CI	SE	Global p-value	Mean	95% CI	SE	Global p-value
Age group	18-24	5.75	(5.63,5.86)	0.06	< 2e-16	5.68	(5.57,5.79)	0.06	< 2e-16
	25-29	5.66	(5.52,5.81)	0.07		5.57	(5.43,5.71)	0.07	
	30-39	5.59	(5.51,5.67)	0.04		5.51	(5.43,5.59)	0.04	
	40-49	5.53	(5.46,5.61)	0.04		5.45	(5.38,5.53)	0.04	
	50-59	5.66	(5.59,5.72)	0.03		5.61	(5.55,5.68)	0.03	
	60-69	6.13	(6.08,6.19)	0.03		6.14	(6.08,6.20)	0.03	
	70-79	6.66	(6.61,6.71)	0.03		6.67	(6.62,6.73)	0.03	
	80 or older	6.86	(6.69,7.02)	0.08		6.88	(6.71,7.05)	0.09	
Gender	Female	6.07	(6.03,6.11)	0.02	< 2e-16	6.02	(5.98,6.06)	0.02	< 2e-16
	Male	5.79	(5.75,5.83)	0.02		5.76	(5.72,5.80)	0.02	
	Other	5.41	(4.78,6.04)	0.30		5.30	(4.69,5.91)	0.30	
Marital status	Divorced	5.67	(5.57,5.77)	0.05	< 2e-16	5.59	(5.49,5.69)	0.05	< 2e-16
	Domestic partner	5.52	(5.14,5.91)	0.20		5.29	(4.90,5.67)	0.20	
	Married	6.23	(6.19,6.26)	0.02		6.19	(6.16,6.23)	0.02	
	Separated	5.82	(5.48,6.16)	0.17		5.78	(5.44,6.11)	0.17	
	Single/Never been married	5.20	(5.15,5.26)	0.03		5.17	(5.12,5.22)	0.03	
	Widowed	6.61	(6.48,6.73)	0.06		6.62	(6.49,6.75)	0.06	
Employment	Employed for an employer	5.81	(5.77,5.85)	0.02	< 2e-16	5.75	(5.71,5.79)	0.02	< 2e-16
	Homemaker	6.25	(6.15,6.34)	0.05		6.24	(6.15,6.34)	0.05	
	None of these/Other	6.10	(6.04,6.17)	0.03		6.09	(6.03,6.16)	0.03	
	Retired	6.36	(6.29,6.42)	0.03		6.39	(6.32,6.45)	0.03	
	Self-employed	6.07	(5.97,6.16)	0.05		5.99	(5.90,6.09)	0.05	
	Student	6.09	(5.93,6.25)	0.08		6.09	(5.93,6.24)	0.08	
	Unemployed and looking for a job	4.47	(4.31,4.62)	0.08		4.39	(4.24,4.54)	0.08	
Religious service attendance	A few times a year	6.16	(6.10,6.23)	0.03	< 2e-16	6.11	(6.04,6.17)	0.03	< 2e-16
	More than once a week	7.02	(6.79,7.25)	0.12		6.91	(6.68,7.15)	0.12	

Variable	Category	Secure Flourishing Index				Flourishing Index			
		Mean	95% CI	SE	Global p-value	Mean	95% CI	SE	Global p-value
	Never	5.83	(5.80,5.86)	0.02		5.80	(5.77,5.83)	0.02	
	Once a week	6.97	(6.77,7.17)	0.10		6.92	(6.71,7.12)	0.10	
	One to three times a month	6.16	(6.04,6.28)	0.06		6.11	(5.99,6.24)	0.06	
Education	Up to 8	5.26	(5.07,5.46)	0.10	< 2e-16	5.17	(4.98,5.37)	0.10	< 2e-16
	9 to 15	6.31	(6.25,6.36)	0.03		6.31	(6.26,6.37)	0.03	
	16+	5.83	(5.80,5.86)	0.02		5.78	(5.75,5.81)	0.02	
Immigration status	Born in another country	6.06	(5.80,6.31)	0.13	0.345	6.01	(5.75,6.27)	0.13	0.378
	Born in this country	5.93	(5.90,5.96)	0.01		5.89	(5.87,5.92)	0.01	
Religious affiliation	Baha'i	5.50	*	*	< 2e-16	4.71	*	*	< 2e-16
	Buddhism	6.24	(6.19,6.28)	0.02		6.21	(6.16,6.26)	0.02	
	Christianity	6.76	(6.55,6.97)	0.11		6.67	(6.47,6.88)	0.10	
	Hinduism	6.75	*	*		6.74	*	*	
	Islam	6.27	*	*		6.12	*	*	
	Judaism	5.88	*	*		5.82	*	*	
	No religion/Atheist/ Agnostic	5.74	(5.70,5.78)	0.02		5.70	(5.66,5.73)	0.02	
	Primal, Animist, or Folk religion	5.74	(4.40,7.07)	0.41		5.74	(4.14,7.33)	0.49	
	Shinto	6.14	(5.95,6.33)	0.10		6.09	(5.90,6.28)	0.10	
	Sikhism	6.35	*	*		6.28	*	*	
	Some other religion	5.59	(4.80,6.39)	0.39		5.50	(4.72,6.27)	0.38	
	Taoism	6.94	*	*		6.89	*	*	
	Confucianism	6.54	(4.16,8.92)	0.41		6.60	(4.19,9.01)	0.41	
	Jainism	6.60	(5.43,7.77)	0.58		6.39	(5.17,7.61)	0.60	

Note. N=20543; $p < .007 = 0.05/7$ (Bonferroni corrected p-value significance threshold); Mean, estimated group mean; CI, confidence interval for the mean within group; SE, complex survey adjusted standard error or the mean; Global p-value, two-tailed Wald-type test of whether there is evidence of any differences in mean scores among groups of a demographic characteristic. *Estimate is not reported due to multiple-imputation and complex survey adjusted degrees of freedom was less than 1.00 leading to insufficient information to provide an estimate of the uncertainty in the estimate. These groups are removed when estimating the global test of mean differences.

Table S10c. Childhood predictors regression analysis results for Japan (N=20543)

		Secure Flourishing Index					Flourishing Index				
Variable	Category	Est	95% CI	SE	Est/SD	Global p-value	Est	95% CI	SE	Est/SD	Global p-value
Relationship with mother	(Ref: Very bad/somewhat bad)					0.008					0.006
	Very good/somewhat good	0.10	(0.02,0.18)	0.04	0.06		0.11	(0.03,0.18)	0.04	0.06	
Relationship with father	(Ref: Very bad/somewhat bad)					< 2e-16					< 2e-16
	Very good/somewhat good	0.32	(0.25,0.38)	0.03	0.18		0.34	(0.27,0.40)	0.03	0.19	
Parent marital status	(Ref: Parents married)					0.114					0.023
	Divorced	0.12	(-0.01,0.26)	0.07	0.07		0.18	(0.04,0.31)	0.07	0.10	
	Parents were never married	0.06	(-0.09,0.21)	0.08	0.03		0.03	(-0.12,0.18)	0.08	0.02	
	One or both parents had died	0.11	(-0.03,0.25)	0.07	0.06		0.12	(-0.02,0.26)	0.07	0.07	
Subjective financial status of family growing up	(Ref: Got by)					< 2e-16					< 2e-16
	Lived comfortably	0.38	(0.32,0.43)	0.03	0.21		0.34	(0.28,0.40)	0.03	0.19	
	Found it difficult	-0.19	(-0.27,-0.10)	0.04	-0.10		-0.16	(-0.24,-0.07)	0.04	-0.09	
	Found it very difficult	-0.57	(-0.73,-0.42)	0.08	-0.32		-0.50	(-0.65,-0.34)	0.08	-0.28	
Abuse	(Ref: No)					6.18e-05					0.001
	Yes	-0.24	(-0.35,-0.12)	0.06	-0.13		-0.19	(-0.31,-0.07)	0.06	-0.11	

Variable	Category	Secure Flourishing Index					Flourishing Index				
		Est	95% CI	SE	Est/SD	Global p-value	Est	95% CI	SE	Est/SD	Global p-value
Outsider growing up	(Ref: No)					4.03e-04					7.32e-04
	Yes	-0.18	(-0.30,-0.06)	0.06	-0.10		-0.17	(-0.30,-0.05)	0.06	-0.10	
Self-rated health growing up	(Ref: Good)					< 2e-16					< 2e-16
	Excellent	1.13	(1.04,1.22)	0.05	0.63		1.20	(1.11,1.29)	0.05	0.67	
	Very good	0.52	(0.46,0.57)	0.03	0.29		0.54	(0.48,0.60)	0.03	0.30	
	Fair	-0.51	(-0.59,-0.44)	0.04	-0.29		-0.53	(-0.61,-0.45)	0.04	-0.30	
	Poor	-0.82	(-1.00,-0.64)	0.09	-0.46		-0.83	(-1.01,-0.65)	0.09	-0.46	
Immigration status	(Ref: Born in this country)					0.001					0.001
	Born in another country	0.37	(0.15,0.60)	0.12	0.21		0.37	(0.14,0.59)	0.11	0.20	
Age 12 religious service attendance	(Ref: Never)					< 2e-16					< 2e-16
	At least 1/week	0.46	(0.26,0.66)	0.10	0.26		0.49	(0.29,0.68)	0.10	0.27	
	1-3/month	0.64	(0.52,0.77)	0.06	0.36		0.64	(0.52,0.76)	0.06	0.36	
	< 1/month	0.17	(0.12,0.23)	0.03	0.10		0.17	(0.11,0.23)	0.03	0.10	
Year of birth	(Ref: 1998-2005; current age: 18-24)					< 2e-16					< 2e-16
	1993-1998; age 25-29	-0.06	(-0.21,0.10)	0.08	-0.03		-0.03	(-0.19,0.12)	0.08	-0.02	
	1983-1993; age 30-39	-0.07	(-0.19,0.05)	0.06	-0.04		-0.06	(-0.18,0.06)	0.06	-0.03	
	1973-1983; age 40-49	-0.16	(-0.28,-0.04)	0.06	-0.09		-0.15	(-0.27,-0.03)	0.06	-0.08	
	1963-1973; age 50-59	0.03	(-0.08,0.14)	0.06	0.02		0.00	(-0.11,0.11)	0.06	0.00	

Variable	Category	Secure Flourishing Index					Flourishing Index				
		Est	95% CI	SE	Est/SD	Global p-value	Est	95% CI	SE	Est/SD	Global p-value
	1953-1963; age 60-69	0.47	(0.36,0.58)	0.06	0.26		0.39	(0.28,0.50)	0.06	0.22	
	1943-1953; age 70-79	0.93	(0.82,1.04)	0.06	0.52		0.84	(0.73,0.95)	0.06	0.47	
	1943 or earlier; age 80+	1.18	(0.99,1.36)	0.09	0.66		1.07	(0.88,1.25)	0.09	0.60	
Gender	(Ref: Male)					4.44e-16					< 2e-16
	Female	0.21	(0.16,0.26)	0.03	0.12		0.24	(0.19,0.29)	0.03	0.13	
	Other	0.39	(-0.19,0.97)	0.30	0.22		0.44	(-0.18,1.07)	0.32	0.25	
Religious affiliation	(Ref: No religion/Atheist/Agnostic)					8.92e-07					1.95e-07
	Buddhism	0.15	(0.09,0.20)	0.03	0.08		0.15	(0.10,0.21)	0.03	0.09	
	Collapsed affiliations with prevalence<3%	0.10	(-0.03,0.23)	0.07	0.06		0.13	(-0.00,0.26)	0.07	0.07	
Race/ethnicity	(Ref: Plurality group)										

Note. N=20543; p < .004 (Bonferroni corrected threshold); Est., estimated effect of childhood predictor on flourishing score; CI, confidence interval; SE, standard error of the estimated effect; Est/SD, a more standardized measure of effect size--estimated effect of flourishing divided by standard deviation of flourishing--leads to the interpretation, for those with the given status (e.g., those with a good/very good relationship with mother compared to those with bad/very bad) are 0.XX standard deviations higher/lower on flourishing; the Global p-value corresponds to the two-sided joint parameter Wald-type test of whether any of the levels' parameters are non-zero, for history of abuse, outsider, relationship wtih mother/father, this is test of whether the estimated effect is non-zero, for multiple-category predictors (age, health, financial status), this is a joint test of whether any of these effects are non-zero. Note the confidence interval of the effect estimate can contradict the reported global p-value (e.g., for the single-category effects of relationship with mother). In such cases, the reported confidence interval is more robust with corrected degrees of freedom from the pooling across multiple imputations, whereas the global p-value is based on a Wald-type test and is less robust to uncertainty attributable to multiple imputation.

Table S10d. Sensitivity to unmeasured confounding of childhood predictors in Japan (N=20543)

Variable	Category	Secure Flourishing Index		Flourishing Index	
		E-value for Estimate	E-value for 95% CI	E-value for Estimate	E-value for 95% CI
Relationship with mother	(Ref: Very bad/somewhat bad)				
	Very good/somewhat good	1.29	1.12	1.30	1.14
Relationship with father	(Ref: Very bad/somewhat bad)				
	Very good/somewhat good	1.63	1.53	1.66	1.56
Parent marital status	(Ref: Parents married)				
	Divorced	1.32	1.00	1.41	1.17
	Parents were never married	1.20	1.00	1.15	1.00
	One or both parents had died	1.31	1.00	1.32	1.00
Subjective financial status of family growing up	(Ref: Got by)				
	Lived comfortably	1.72	1.63	1.66	1.58
	Found it difficult	1.43	1.30	1.38	1.24
	Found it very difficult	2.01	1.78	1.90	1.67
Abuse	(Ref: No)				
	Yes	1.51	1.32	1.44	1.24
Outsider growing up	(Ref: No)				
	Yes	1.42	1.22	1.41	1.19
Self-rated health growing up	(Ref: Good)				
	Excellent	2.95	2.78	3.08	2.92
	Very good	1.92	1.84	1.96	1.87
	Fair	1.92	1.81	1.95	1.83
	Poor	2.40	2.12	2.42	2.13
Immigration status	(Ref: Born in this country)				
	Born in another country	1.71	1.37	1.70	1.36
Age 12 religious service attendance	(Ref: Never)				
	At least 1/week	1.84	1.55	1.88	1.59
	1-3/month	2.12	1.94	2.11	1.93
	< 1/month	1.41	1.31	1.41	1.31
Year of birth	(Ref: 1998-2005; current age: 18-24)				
	1993-1998; age 25-29	1.20	1.00	1.15	1.00
	1983-1993; age 30-39	1.23	1.00	1.21	1.00

Variable	Category	Secure Flourishing Index		Flourishing Index	
		E-value for Estimate	E-value for 95% CI	E-value for Estimate	E-value for 95% CI
	1973-1983; age 40-49	1.39	1.17	1.37	1.14
	1963-1973; age 50-59	1.14	1.00	1.01	1.00
	1953-1963; age 60-69	1.85	1.69	1.74	1.57
	1943-1953; age 70-79	2.59	2.41	2.44	2.26
	1943 or earlier; age 80+	3.04	2.70	2.84	2.51
Gender	(Ref: Male)				
	Female	1.47	1.39	1.51	1.43
	Other	1.73	1.00	1.82	1.00
Religious affiliation	(Ref: No religion/Atheist/Agnostic)				
	Buddhism	1.37	1.27	1.38	1.28
	Collapsed affiliations with prevalence<3%	1.29	1.00	1.34	1.00
Race/ethnicity	(Ref: Plurality group)				

Table S11a. Nationally representative descriptive statistics for Kenya

Characteristic	N = 11,389[1]
**Age group**	
1998-2005; age 18-24	2,868 (25%)
1993-1998; age 25-29	2,035 (18%)
1983-1993; age 30-39	2,564 (23%)
1973-1983; age 40-49	1,708 (15%)
1963-1973; age 50-59	1,072 (9.4%)
1953-1963; age 60-69	710 (6.2%)
1943-1953; age 70-79	360 (3.2%)
1943 or earlier; age 80+	67 (0.6%)
(Missing)	5 (<0.1%)
**Gender**	
Male	5,567 (49%)
Female	5,813 (51%)
Other	2 (<0.1%)
(Missing)	7 (<0.1%)
**Race/Ethnicity**	
Embu	197 (1.7%)
Kalenjin	1,377 (12%)
Kamba	1,299 (11%)
Kenyan Somali/Somali	396 (3.5%)
Kikuyu	2,119 (19%)
Kisii	789 (6.9%)
Luhya	1,943 (17%)
Luo	1,120 (9.8%)
Maasai	237 (2.1%)
Meru	630 (5.5%)
Miji Kenda tribes	708 (6.2%)
Other	548 (4.8%)
(Missing)	27 (0.2%)
**Marital status**	
Married	6,626 (58%)
Separated	467 (4.1%)
Divorced	111 (1.0%)
Widowed	464 (4.1%)
Single, never married	3,531 (31%)
Domestic Partner	146 (1.3%)
(Missing)	43 (0.4%)
**Employment**	
Employed for an employer	1,467 (13%)
Self-employed	3,630 (32%)
Retired	319 (2.8%)
Student	1,136 (10.0%)
Homemaker	1,537 (13%)
Unemployed and looking for a job	3,153 (28%)
None of these/Other	138 (1.2%)
(Missing)	9 (<0.1%)
**Religious service attendance**	
More than 1/week	2,774 (24%)
1/week	6,063 (53%)
1-3/month	1,219 (11%)
A few times a year	855 (7.5%)
Never	465 (4.1%)
(Missing)	13 (0.1%)
**Education**	
Up to 8 years	4,485 (39%)
9-15 years	6,115 (54%)
16+ years	783 (6.9%)
(Missing)	6 (<0.1%)

Characteristic	N = 11,389[1]
**Immigration status**	
Born in this country	11,270 (99%)
Born in another country	117 (1.0%)
(Missing)	2 (<0.1%)
**Religious affiliation as an adult (now)**	
Christianity	10,334 (91%)
Islam	918 (8.1%)
Hinduism	0 (0%)
Buddhism	1 (<0.1%)
Judaism	3 (<0.1%)
Sikhism	0 (0%)
Baha'i	1 (<0.1%)
Jainism	1 (<0.1%)
Shinto	0 (0%)
Taoism	0 (0%)
Confucianism	3 (<0.1%)
Primal, Animist, or Folk religion	7 (<0.1%)
Spiritism	0 (0%)
Umbanda, Candomble, and other African-derived religions	0 (0%)
Chinese folk/traditional religion	0 (0%)
Some other religion	5 (<0.1%)
No religion/Atheist/Agnostic	108 (0.9%)
(Missing)	9 (<0.1%)
**Parent marital status**	
Parents married	9,238 (81%)
Divorced	697 (6.1%)
Parents were never married	681 (6.0%)
One or both parents had died	471 (4.1%)
(Missing)	301 (2.6%)
**Age 12 religious service attendance**	
At least 1/week	9,189 (81%)
1-3/month	1,687 (15%)
<1/month	236 (2.1%)
Never	198 (1.7%)
(Missing)	79 (0.7%)
**Relationship with mother**	
Very good	9,418 (83%)
Somewhat good	1,435 (13%)
Somewhat bad	130 (1.1%)
Very bad	100 (0.9%)
Does not apply	240 (2.1%)
(Missing)	66 (0.6%)
**Relationship with father**	
Very good	7,958 (70%)
Somewhat good	1,896 (17%)
Somewhat bad	216 (1.9%)
Very bad	220 (1.9%)
Does not apply	967 (8.5%)
(Missing)	132 (1.2%)
**Outsider growing up**	
Yes	1,223 (11%)
No	10,114 (89%)
(Missing)	52 (0.5%)
**Self-reported history of abuse**	
Yes	1,300 (11%)
No	10,039 (88%)
(Missing)	49 (0.4%)
**Self-rated health growing up**	
Excellent	4,449 (39%)

Characteristic	N = 11,389[1]
Very good	2,598 (23%)
Good	2,582 (23%)
Fair	1,384 (12%)
Poor	349 (3.1%)
(Missing)	26 (0.2%)
**Subjective financial status of family growing up**	
Lived comfortably	3,026 (27%)
Got by	3,279 (29%)
Found it difficult	4,071 (36%)
Found it very difficult	994 (8.7%)
(Missing)	19 (0.2%)
**Religious affiliation at age 12**	
Christianity	10,369 (91%)
Islam	916 (8.0%)
Hinduism	0 (0%)
Buddhism	5 (<0.1%)
Judaism	6 (<0.1%)
Sikhism	0 (<0.1%)
Baha'i	3 (<0.1%)
Jainism	1 (<0.1%)
Shinto	0 (0%)
Taoism	0 (0%)
Confucianism	0 (0%)
Primal, Animist, or Folk religion	13 (0.1%)
Spiritism	0 (0%)
Umbanda, Candomble, and other African-derived religions	0 (0%)
Chinese folk/traditional religion	0 (0%)
Some other religion	0 (<0.1%)
No religion/Atheist/Agnostic	67 (0.6%)
(Missing)	9 (<0.1%)

[1]n (%)

Table S11b. Means by demographic category for Kenya (N=11389)

		Secure Flourishing Index				Flourishing Index			
Variable	Category	Mean	95% CI	SE	Global p-value	Mean	95% CI	SE	Global p-value
Age group	18-24	7.95	(7.88,8.02)	0.04	2.26e-07	7.47	(7.40,7.54)	0.04	2.48e-09
	25-29	7.89	(7.80,7.98)	0.04		7.39	(7.30,7.48)	0.05	
	30-39	7.74	(7.66,7.82)	0.04		7.23	(7.16,7.31)	0.04	
	40-49	7.59	(7.47,7.71)	0.06		7.07	(6.96,7.19)	0.06	
	50-59	7.60	(7.45,7.75)	0.08		7.10	(6.95,7.24)	0.07	
	60-69	7.63	(7.43,7.83)	0.10		7.21	(7.00,7.42)	0.11	
	70-79	7.58	(7.24,7.91)	0.17		7.25	(6.92,7.59)	0.17	
	80 or older	7.70	(6.88,8.52)	0.41		7.22	(6.41,8.02)	0.40	
Gender	Female	7.74	(7.67,7.81)	0.04	0.074	7.23	(7.16,7.30)	0.04	0.010
	Male	7.80	(7.73,7.88)	0.04		7.34	(7.28,7.41)	0.04	
	Other	9.26	*	*		7.91	*	*	
Marital status	Divorced	7.05	(6.68,7.42)	0.18	2.00e-08	6.69	(6.35,7.02)	0.17	1.03e-08
	Domestic partner	7.53	(7.22,7.84)	0.16		7.12	(6.80,7.43)	0.16	
	Married	7.81	(7.74,7.88)	0.04		7.32	(7.24,7.39)	0.04	
	Separated	7.38	(7.18,7.58)	0.10		6.83	(6.63,7.03)	0.10	
	Single/Never been married	7.85	(7.78,7.92)	0.04		7.37	(7.30,7.44)	0.04	
	Widowed	7.25	(6.99,7.51)	0.13		6.82	(6.56,7.09)	0.13	
Employment	Employed for an employer	7.78	(7.68,7.88)	0.05	3.14e-05	7.33	(7.23,7.42)	0.05	6.35e-06
	Homemaker	7.77	(7.63,7.90)	0.07		7.27	(7.14,7.41)	0.07	
	None of these/Other	7.41	(7.06,7.77)	0.18		6.94	(6.60,7.28)	0.17	
	Retired	7.41	(7.10,7.73)	0.16		7.08	(6.77,7.38)	0.16	
	Self-employed	7.74	(7.65,7.82)	0.04		7.28	(7.20,7.36)	0.04	
	Student	7.99	(7.89,8.08)	0.05		7.52	(7.42,7.61)	0.05	
	Unemployed and looking for a job	7.79	(7.70,7.87)	0.04		7.23	(7.15,7.32)	0.04	
Religious service attendance	A few times a year	7.35	(7.21,7.50)	0.07	4.44e-16	6.93	(6.78,7.07)	0.07	2.50e-10
	More than once a week	7.83	(7.73,7.93)	0.05		7.32	(7.22,7.42)	0.05	

Variable	Category	Secure Flourishing Index				Flourishing Index			
		Mean	95% CI	SE	Global p-value	Mean	95% CI	SE	Global p-value
	Never	7.30	(7.07,7.53)	0.12		6.95	(6.72,7.17)	0.11	
	Once a week	7.89	(7.82,7.95)	0.03		7.37	(7.31,7.44)	0.03	
	One to three times a month	7.54	(7.42,7.67)	0.06		7.14	(7.02,7.26)	0.06	
Education	Up to 8	7.74	(7.64,7.83)	0.05	0.021	7.25	(7.16,7.34)	0.05	0.022
	9 to 15	7.63	(7.49,7.77)	0.07		7.15	(7.01,7.29)	0.07	
	16+	7.82	(7.76,7.88)	0.03		7.33	(7.27,7.39)	0.03	
Immigration status	Born in another country	7.49	(7.12,7.86)	0.19	0.130	7.03	(6.63,7.42)	0.20	0.193
	Born in this country	7.77	(7.71,7.84)	0.03		7.29	(7.23,7.35)	0.03	
Religious affiliation	Baha'i	9.70	*	*	< 2e-16	9.42	*	*	< 2e-16
	Buddhism	7.20	*	*		7.08	*	*	
	Christianity	7.78	(7.72,7.84)	0.03		7.30	(7.24,7.35)	0.03	
	Islam	7.66	(7.38,7.93)	0.14		7.16	(6.90,7.42)	0.13	
	Judaism	8.90	*	*		7.56	*	*	
	No religion/Atheist/ Agnostic	7.58	(7.22,7.95)	0.19		7.23	(6.84,7.61)	0.19	
	Primal, Animist, or Folk religion	6.69	(5.21,8.17)	0.74		6.16	(4.51,7.81)	0.82	
	Some other religion	8.17	*	*		7.90	*	*	
	Confucianism	6.50	*	*		6.58	*	*	
	Jainism	7.00	*	*		6.83	*	*	

Note. N=11389; p < .007 = 0.05/7 (Bonferroni corrected p-value significance threshold); Mean, estimated group mean; CI, confidence interval for the mean within group; SE, complex survey adjusted standard error or the mean; Global p-value, two-tailed Wald-type test of whether there is evidence of any differences in mean scores among groups of a demographic characteristic. *Estimate is not reported due to multiple-imputation and complex survey adjusted degrees of freedom was less than 1.00 leading to insufficient information to provide an estimate of the uncertainty in the estimate. These groups are removed when estimating the global test of mean differences.

Table S11c. Childhood predictors regression analysis results for Kenya (N=11389)

		Secure Flourishing Index					Flourishing Index				
Variable	Category	Est	95% CI	SE	Est/SD	Global p-value	Est	95% CI	SE	Est/SD	Global p-value
Relationship with mother	(Ref: Very bad/somewhat bad)					0.125					0.255
	Very good/somewhat good	0.18	(-0.05,0.42)	0.12	0.11		0.14	(-0.10,0.39)	0.13	0.09	
Relationship with father	(Ref: Very bad/somewhat bad)					0.248					0.193
	Very good/somewhat good	0.10	(-0.07,0.28)	0.09	0.06		0.12	(-0.06,0.30)	0.09	0.07	
Parent marital status	(Ref: Parents married)					3.09e-07					5.35e-07
	Divorced	-0.23	(-0.40,-0.05)	0.09	-0.14		-0.27	(-0.45,-0.09)	0.09	-0.17	
	Parents were never married	-0.43	(-0.59,-0.26)	0.08	-0.26		-0.45	(-0.63,-0.27)	0.09	-0.28	
	One or both parents had died	-0.18	(-0.39,0.03)	0.11	-0.11		-0.16	(-0.37,0.04)	0.10	-0.10	
Subjective financial status of family growing up	(Ref: Got by)					7.92e-06					4.90e-05
	Lived comfortably	0.19	(0.09,0.30)	0.05	0.12		0.21	(0.11,0.31)	0.05	0.13	
	Found it difficult	-0.05	(-0.15,0.04)	0.05	-0.03		0.01	(-0.09,0.10)	0.05	0.00	
	Found it very difficult	-0.11	(-0.28,0.05)	0.08	-0.07		-0.06	(-0.22,0.11)	0.08	-0.04	
Abuse	(Ref: No)					5.01e-13					7.51e-14
	Yes	-0.47	(-0.59,-0.35)	0.06	-0.29		-0.48	(-0.60,-0.37)	0.06	-0.30	

Variable	Category	Secure Flourishing Index					Flourishing Index				
		Est	95% CI	SE	Est/SD	Global p-value	Est	95% CI	SE	Est/SD	Global p-value
Outsider growing up	(Ref: No)					0.098					0.039
	Yes	-0.11	(-0.24,0.02)	0.07	-0.07		-0.14	(-0.28,-0.01)	0.07	-0.09	
Self-rated health growing up	(Ref: Good)					1.49e-06					4.43e-04
	Excellent	0.16	(0.06,0.26)	0.05	0.10		0.12	(0.02,0.23)	0.05	0.08	
	Very good	0.14	(0.04,0.24)	0.05	0.09		0.11	(0.00,0.21)	0.05	0.07	
	Fair	-0.10	(-0.22,0.03)	0.06	-0.06		-0.07	(-0.20,0.05)	0.06	-0.04	
	Poor	-0.28	(-0.55,-0.01)	0.14	-0.18		-0.25	(-0.52,0.02)	0.14	-0.16	
Immigration status	(Ref: Born in this country)					0.285					0.167
	Born in another country	-0.20	(-0.57,0.17)	0.19	-0.13		-0.24	(-0.58,0.10)	0.17	-0.15	
Age 12 religious service attendance	(Ref: Never)					0.139					0.227
	At least 1/week	-0.18	(-0.54,0.19)	0.19	-0.11		-0.12	(-0.50,0.25)	0.19	-0.08	
	1-3/month	-0.17	(-0.55,0.21)	0.19	-0.10		-0.12	(-0.51,0.27)	0.20	-0.07	
	< 1/month	-0.41	(-0.81,0.00)	0.21	-0.25		-0.35	(-0.77,0.07)	0.21	-0.22	
Year of birth	(Ref: 1998-2005; current age: 18-24)					1.41e-07					1.80e-06
	1993-1998; age 25-29	-0.06	(-0.16,0.04)	0.05	-0.04		-0.05	(-0.14,0.05)	0.05	-0.03	
	1983-1993; age 30-39	-0.20	(-0.29,-0.11)	0.05	-0.12		-0.18	(-0.27,-0.09)	0.05	-0.11	
	1973-1983; age 40-49	-0.36	(-0.48,-0.24)	0.06	-0.22		-0.33	(-0.45,-0.21)	0.06	-0.21	
	1963-1973; age 50-59	-0.35	(-0.50,-0.19)	0.08	-0.21		-0.33	(-0.50,-0.17)	0.08	-0.21	

Variable	Category	Secure Flourishing Index Est	95% CI	SE	Est/SD	Global p-value	Flourishing Index Est	95% CI	SE	Est/SD	Global p-value
	1953-1963; age 60-69	-0.26	(-0.47,-0.05)	0.11	-0.16		-0.33	(-0.53,-0.13)	0.10	-0.20	
	1943-1953; age 70-79	-0.24	(-0.58,0.09)	0.17	-0.15		-0.41	(-0.74,-0.08)	0.17	-0.26	
	1943 or earlier; age 80+	-0.31	(-1.03,0.42)	0.37	-0.19		-0.28	(-1.03,0.47)	0.38	-0.18	
Gender	(Ref: Male)					0.007					0.043
	Female	-0.11	(-0.19,-0.03)	0.04	-0.07		-0.06	(-0.13,0.02)	0.04	-0.04	
	Other	0.74	(-0.06,1.54)	0.41	0.46		1.58	(0.12,3.04)	0.75	0.98	
Religious affiliation	(Ref: Christianity)					0.602					0.694
	Islam	-0.11	(-0.34,0.11)	0.11	-0.07		-0.10	(-0.34,0.13)	0.12	-0.06	
	Collapsed affiliations with prevalence<3 %	-0.05	(-0.62,0.52)	0.29	-0.03		-0.04	(-0.60,0.51)	0.28	-0.03	
Race/ethnicity	(Ref: Plurality group)					0.826					0.369
	Non-plurality groups	0.02	(-0.12,0.15)	0.07	0.01		0.07	(-0.08,0.21)	0.07	0.04	

Note. N=11389; p < .004 (Bonferroni corrected threshold); Est., estimated effect of childhood predictor on flourishing score; CI, confidence interval; SE, standard error of the estimated effect; Est/SD, a more standardized measure of effect size--estimated effect of flourishing divided by standard deviation of flourishing--leads to the interpretation, for those with the given status (e.g., those with a good/very good relationship with mother compared to those with bad/very bad) are 0.XX standard deviations higher/lower on flourishing; the Global p-value corresponds to the two-sided joint parameter Wald-type test of whether any of the levels' parameters are non-zero, for history of abuse, outsider, relationship wtih mother/father, this is test of whether the estimated effect is non-zero, for multiple-category predictors (age, health, financial status), this is a joint test of whether any of these effects are non-zero. Note the confidence interval of the effect estimate can contradict the reported global p-value (e.g., for the single-category effects of relationship with mother). In such cases, the reported confidence interval is more robust with corrected degrees of freedom from the pooling across multiple imputations, whereas the global p-value is based on a Wald-type test and is less robust to uncertainty attributable to multiple imputation.

Table S11d. Sensitivity to unmeasured confounding of childhood predictors in Kenya (N=11389)

		Secure Flourishing Index		Flourishing Index	
Variable	Category	E-value for Estimate	E-value for 95% CI	E-value for Estimate	E-value for 95% CI
Relationship with mother	(Ref: Very bad/somewhat bad)				
	Very good/somewhat good	1.46	1.00	1.39	1.00
Relationship with father	(Ref: Very bad/somewhat bad)				
	Very good/somewhat good	1.31	1.00	1.34	1.00
Parent marital status	(Ref: Parents married)				
	Divorced	1.53	1.21	1.60	1.28
	Parents were never married	1.86	1.59	1.90	1.61
	One or both parents had died	1.45	1.00	1.42	1.00
Subjective financial status of family growing up	(Ref: Got by)				
	Lived comfortably	1.47	1.28	1.50	1.33
	Found it difficult	1.21	1.00	1.07	1.00
	Found it very difficult	1.33	1.00	1.21	1.00
Abuse	(Ref: No)				
	Yes	1.94	1.74	1.96	1.76
Outsider growing up	(Ref: No)				
	Yes	1.33	1.00	1.38	1.07
Self-rated health growing up	(Ref: Good)				
	Excellent	1.42	1.23	1.35	1.11
	Very good	1.38	1.18	1.32	1.04
	Fair	1.31	1.00	1.25	1.00
	Poor	1.62	1.09	1.57	1.00
Immigration status	(Ref: Born in this country)				
	Born in another country	1.49	1.00	1.55	1.00
Age 12 religious service attendance	(Ref: Never)				
	At least 1/week	1.45	1.00	1.35	1.00
	1-3/month	1.43	1.00	1.34	1.00
	< 1/month	1.83	1.00	1.73	1.00
Year of birth	(Ref: 1998-2005; current age: 18-24)				
	1993-1998; age 25-29	1.22	1.00	1.19	1.00
	1983-1993; age 30-39	1.48	1.32	1.44	1.28

Variable	Category	Secure Flourishing Index		Flourishing Index	
		E-value for Estimate	E-value for 95% CI	E-value for Estimate	E-value for 95% CI
	1973-1983; age 40-49	1.75	1.55	1.70	1.50
	1963-1973; age 50-59	1.73	1.47	1.70	1.43
	1953-1963; age 60-69	1.59	1.21	1.70	1.37
	1943-1953; age 70-79	1.56	1.00	1.83	1.27
	1943 or earlier; age 80+	1.66	1.00	1.62	1.00
Gender	(Ref: Male)				
	Female	1.32	1.15	1.22	1.00
	Other	2.41	1.00	4.30	1.35
Religious affiliation	(Ref: Christianity)				
	Islam	1.33	1.00	1.31	1.00
	Collapsed affiliations with prevalence<3%	1.20	1.00	1.19	1.00
Race/ethnicity	(Ref: Plurality group)				
	Non-plurality groups	1.10	1.00	1.24	1.00

Table S12a. Nationally representative descriptive statistics for Mexico

Characteristic	N = 5,776[1]
**Age group**	
1998-2005; age 18-24	986 (17%)
1993-1998; age 25-29	623 (11%)
1983-1993; age 30-39	1,312 (23%)
1973-1983; age 40-49	1,027 (18%)
1963-1973; age 50-59	873 (15%)
1953-1963; age 60-69	611 (11%)
1943-1953; age 70-79	277 (4.8%)
1943 or earlier; age 80+	68 (1.2%)
(Missing)	0 (0%)
**Gender**	
Male	2,755 (48%)
Female	2,997 (52%)
Other	3 (<0.1%)
(Missing)	21 (0.4%)
**Race/Ethnicity**	
Black	108 (1.9%)
Indigenous	594 (10%)
Mestizo	2,762 (48%)
Mulatto	63 (1.1%)
Other	339 (5.9%)
White	1,116 (19%)
(Missing)	794 (14%)
**Marital status**	
Married	2,089 (36%)
Separated	403 (7.0%)
Divorced	230 (4.0%)
Widowed	347 (6.0%)
Single, never married	1,432 (25%)
Domestic Partner	1,109 (19%)
(Missing)	166 (2.9%)
**Employment**	
Employed for an employer	1,921 (33%)
Self-employed	1,091 (19%)
Retired	386 (6.7%)
Student	247 (4.3%)
Homemaker	1,257 (22%)
Unemployed and looking for a job	564 (9.8%)
None of these/Other	169 (2.9%)
(Missing)	141 (2.4%)
**Religious service attendance**	
More than 1/week	609 (11%)
1/week	1,261 (22%)
1-3/month	676 (12%)
A few times a year	2,054 (36%)
Never	1,134 (20%)
(Missing)	43 (0.7%)
**Education**	
Up to 8 years	1,291 (22%)
9-15 years	3,180 (55%)
16+ years	1,304 (23%)
(Missing)	1 (<0.1%)
**Immigration status**	
Born in this country	5,517 (96%)
Born in another country	108 (1.9%)
(Missing)	151 (2.6%)
**Religious affiliation as an adult (now)**	
Christianity	4,844 (84%)

Characteristic	N = 5,776[1]
Islam	2 (<0.1%)
Hinduism	3 (<0.1%)
Buddhism	6 (0.1%)
Judaism	7 (0.1%)
Sikhism	0 (0%)
Baha'i	1 (<0.1%)
Jainism	1 (<0.1%)
Shinto	2 (<0.1%)
Taoism	4 (<0.1%)
Confucianism	1 (<0.1%)
Primal, Animist, or Folk religion	20 (0.3%)
Spiritism	0 (0%)
Umbanda, Candomble, and other African-derived religions	0 (0%)
Chinese folk/traditional religion	0 (0%)
Some other religion	41 (0.7%)
No religion/Atheist/Agnostic	770 (13%)
(Missing)	75 (1.3%)
**Parent marital status**	
Parents married	3,999 (69%)
Divorced	341 (5.9%)
Parents were never married	827 (14%)
One or both parents had died	176 (3.0%)
(Missing)	432 (7.5%)
**Age 12 religious service attendance**	
At least 1/week	2,514 (44%)
1-3/month	1,162 (20%)
<1/month	1,087 (19%)
Never	944 (16%)
(Missing)	69 (1.2%)
**Relationship with mother**	
Very good	3,912 (68%)
Somewhat good	1,340 (23%)
Somewhat bad	177 (3.1%)
Very bad	90 (1.6%)
Does not apply	177 (3.1%)
(Missing)	80 (1.4%)
**Relationship with father**	
Very good	3,089 (53%)
Somewhat good	1,556 (27%)
Somewhat bad	335 (5.8%)
Very bad	267 (4.6%)
Does not apply	470 (8.1%)
(Missing)	60 (1.0%)
**Outsider growing up**	
Yes	772 (13%)
No	4,897 (85%)
(Missing)	107 (1.9%)
**Self-reported history of abuse**	
Yes	905 (16%)
No	4,604 (80%)
(Missing)	267 (4.6%)
**Self-rated health growing up**	
Excellent	1,860 (32%)
Very good	1,350 (23%)
Good	1,677 (29%)
Fair	743 (13%)
Poor	133 (2.3%)
(Missing)	14 (0.2%)
**Subjective financial status of family growing up**	

Characteristic	N = 5,776[1]
Lived comfortably	1,775 (31%)
Got by	1,872 (32%)
Found it difficult	1,712 (30%)
Found it very difficult	369 (6.4%)
(Missing)	48 (0.8%)
**Religious affiliation at age 12**	
Christianity	5,337 (92%)
Islam	6 (<0.1%)
Hinduism	1 (<0.1%)
Buddhism	1 (<0.1%)
Judaism	8 (0.1%)
Sikhism	4 (<0.1%)
Baha'i	1 (<0.1%)
Jainism	0 (0%)
Shinto	2 (<0.1%)
Taoism	5 (<0.1%)
Confucianism	0 (0%)
Primal, Animist, or Folk religion	2 (<0.1%)
Spiritism	0 (0%)
Umbanda, Candomble, and other African-derived religions	0 (0%)
Chinese folk/traditional religion	0 (0%)
Some other religion	7 (0.1%)
No religion/Atheist/Agnostic	328 (5.7%)
(Missing)	74 (1.3%)

[1]n (%)

Table S12b. Means by demographic category for Mexico (N=5776)

Variable	Category	Secure Flourishing Index				Flourishing Index			
		Mean	95% CI	SE	Global p-value	Mean	95% CI	SE	Global p-value
Age group	18-24	8.01	(7.91,8.11)	0.05	8.86e-06	7.48	(7.38,7.58)	0.05	2.30e-07
	25-29	8.00	(7.86,8.14)	0.07		7.42	(7.28,7.56)	0.07	
	30-39	8.20	(8.11,8.29)	0.05		7.58	(7.49,7.68)	0.05	
	40-49	8.25	(8.14,8.36)	0.06		7.68	(7.56,7.79)	0.06	
	50-59	8.35	(8.25,8.46)	0.05		7.76	(7.64,7.87)	0.06	
	60-69	8.28	(8.14,8.42)	0.07		7.81	(7.66,7.96)	0.08	
	70-79	8.37	(8.18,8.55)	0.09		7.96	(7.77,8.15)	0.10	
	80 or older	7.98	(7.37,8.58)	0.30		7.80	(7.26,8.33)	0.27	
Gender	Female	8.15	(8.09,8.21)	0.03	0.095	7.54	(7.48,7.60)	0.03	3.01e-04
	Male	8.24	(8.17,8.30)	0.03		7.74	(7.67,7.81)	0.04	
	Other	7.47	*	*		7.57	*	*	
Marital status	Divorced	8.19	(7.97,8.41)	0.11	2.55e-15	7.67	(7.45,7.90)	0.12	4.68e-11
	Domestic partner	8.21	(8.10,8.31)	0.05		7.56	(7.46,7.66)	0.05	
	Married	8.39	(8.33,8.46)	0.03		7.83	(7.77,7.90)	0.04	
	Separated	7.93	(7.76,8.11)	0.09		7.37	(7.18,7.55)	0.09	
	Single/Never been married	7.93	(7.84,8.02)	0.05		7.46	(7.37,7.55)	0.05	
	Widowed	8.32	(8.14,8.51)	0.09		7.73	(7.54,7.93)	0.10	
Employment	Employed for an employer	8.19	(8.11,8.27)	0.04	8.83e-10	7.67	(7.58,7.75)	0.04	2.41e-14
	Homemaker	8.27	(8.18,8.36)	0.05		7.59	(7.50,7.69)	0.05	
	None of these/Other	8.00	(7.72,8.28)	0.14		7.45	(7.18,7.72)	0.14	
	Retired	8.39	(8.24,8.55)	0.08		8.06	(7.91,8.21)	0.08	
	Self-employed	8.31	(8.22,8.40)	0.05		7.75	(7.66,7.85)	0.05	
	Student	7.80	(7.60,8.00)	0.10		7.45	(7.24,7.65)	0.11	
	Unemployed and looking for a job	7.89	(7.75,8.03)	0.07		7.24	(7.10,7.38)	0.07	
Religious service attendance	A few times a year	8.20	(8.13,8.26)	0.03	< 2e-16	7.61	(7.54,7.69)	0.04	< 2e-16
	More than once a week	8.62	(8.51,8.74)	0.06		8.09	(7.96,8.22)	0.07	

Variable	Category	Secure Flourishing Index				Flourishing Index			
		Mean	95% CI	SE	Global p-value	Mean	95% CI	SE	Global p-value
	Never	7.83	(7.72,7.94)	0.06		7.31	(7.20,7.42)	0.06	
	Once a week	8.29	(8.20,8.38)	0.05		7.71	(7.62,7.81)	0.05	
	One to three times a month	8.21	(8.09,8.33)	0.06		7.69	(7.57,7.81)	0.06	
Education	Up to 8	8.19	(8.09,8.28)	0.05	0.983	7.61	(7.51,7.71)	0.05	0.092
	9 to 15	8.20	(8.11,8.29)	0.05		7.73	(7.63,7.82)	0.05	
	16+	8.19	(8.13,8.25)	0.03		7.61	(7.55,7.67)	0.03	
Immigration status	Born in another country	7.58	(7.20,7.96)	0.19	0.001	7.10	(6.73,7.48)	0.19	0.005
	Born in this country	8.20	(8.16,8.25)	0.02		7.65	(7.60,7.69)	0.02	
Religious affiliation	Baha'i	7.50	*	*	< 2e-16	6.83	*	*	< 2e-16
	Buddhism	5.37	*	*		5.57	*	*	
	Christianity	8.26	(8.21,8.31)	0.02		7.69	(7.64,7.74)	0.02	
	Hinduism	8.22	*	*		7.71	*	*	
	Islam	8.49	*	*		7.56	*	*	
	Judaism	8.48	*	*		8.27	*	*	
	No religion/Atheist/Agnostic	7.77	(7.64,7.91)	0.07		7.32	(7.19,7.45)	0.07	
	Primal, Animist, or Folk religion	8.18	(7.00,9.36)	0.47		7.50	(5.99,9.00)	0.60	
	Shinto	7.40	*	*		6.39	*	*	
	Some other religion	8.25	(7.86,8.63)	0.19		7.71	(7.30,8.12)	0.20	
	Taoism	9.10	*	*		8.25	*	*	
	Confucianism	8.00	*	*		6.75	*	*	
	Jainism	6.30	*	*		5.83	*	*	

Note. N=5776; $p < .007 = 0.05/7$ (Bonferroni corrected p-value significance threshold); Mean, estimated group mean; CI, confidence interval for the mean within group; SE, complex survey adjusted standard error or the mean; Global p-value, two-tailed Wald-type test of whether there is evidence of any differences in mean scores among groups of a demographic characteristic. *Estimate is not reported due to multiple-imputation and complex survey adjusted degrees of freedom was less than 1.00 leading to insufficient information to provide an estimate of the uncertainty in the estimate. These groups are removed when estimating the global test of mean differences.

Table S12c. Childhood predictors regression analysis results for Mexico (N=5776)

		Secure Flourishing Index					Flourishing Index				
Variable	Category	Est	95% CI	SE	Est/SD	Global p-value	Est	95% CI	SE	Est/SD	Global p-value
Relationship with mother	(Ref: Very bad/somewhat bad)					3.99e-04					7.55e-05
	Very good/somewhat good	0.35	(0.15,0.55)	0.10	0.26		0.40	(0.20,0.60)	0.10	0.29	
Relationship with father	(Ref: Very bad/somewhat bad)					0.811					0.679
	Very good/somewhat good	0.01	(-0.13,0.16)	0.08	0.01		0.03	(-0.12,0.18)	0.08	0.02	
Parent marital status	(Ref: Parents married)					0.283					0.475
	Divorced	0.10	(-0.08,0.28)	0.09	0.07		0.09	(-0.09,0.27)	0.09	0.07	
	Parents were never married	-0.05	(-0.18,0.09)	0.07	-0.03		-0.03	(-0.16,0.11)	0.07	-0.02	
	One or both parents had died	0.12	(-0.07,0.31)	0.10	0.09		0.09	(-0.10,0.29)	0.10	0.07	
Subjective financial status of family growing up	(Ref: Got by)					2.19e-13					2.20e-06
	Lived comfortably	0.31	(0.20,0.41)	0.05	0.23		0.26	(0.15,0.36)	0.05	0.19	
	Found it difficult	-0.10	(-0.21,0.01)	0.06	-0.07		0.04	(-0.07,0.15)	0.06	0.03	
	Found it very difficult	-0.18	(-0.39,0.03)	0.11	-0.13		-0.03	(-0.24,0.19)	0.11	-0.02	
Abuse	(Ref: No)					5.87e-09					1.92e-08
	Yes	-0.36	(-0.48,-0.24)	0.06	-0.26		-0.35	(-0.48,-0.23)	0.06	-0.26	

		Secure Flourishing Index					Flourishing Index				
Variable	Category	Est	95% CI	SE	Est/SD	Global p-value	Est	95% CI	SE	Est/SD	Global p-value
Outsider growing up	(Ref: No)					3.24e-05					9.35e-06
	Yes	-0.29	(-0.43,-0.15)	0.07	-0.21		-0.32	(-0.46,-0.17)	0.07	-0.23	
Self-rated health growing up	(Ref: Good)					8.27e-13					1.98e-13
	Excellent	0.35	(0.25,0.46)	0.05	0.26		0.36	(0.26,0.47)	0.05	0.26	
	Very good	0.24	(0.12,0.35)	0.06	0.17		0.19	(0.08,0.30)	0.06	0.14	
	Fair	-0.09	(-0.25,0.06)	0.08	-0.07		-0.12	(-0.27,0.03)	0.08	-0.09	
	Poor	-0.17	(-0.54,0.20)	0.19	-0.13		-0.17	(-0.55,0.20)	0.19	-0.13	
Immigration status	(Ref: Born in this country)					0.025					0.008
	Born in another country	-0.40	(-0.76,-0.05)	0.18	-0.30		-0.48	(-0.84,-0.13)	0.18	-0.35	
Age 12 religious service attendance	(Ref: Never)					0.106					0.015
	At least 1/week	0.15	(0.01,0.28)	0.07	0.11		0.15	(0.03,0.28)	0.06	0.11	
	1-3/month	0.05	(-0.09,0.18)	0.07	0.04		0.03	(-0.11,0.16)	0.07	0.02	
	< 1/month	0.05	(-0.10,0.20)	0.08	0.04		-0.01	(-0.15,0.14)	0.07	-0.01	
Year of birth	(Ref: 1998-2005; current age: 18-24)					1.10e-09					1.55e-05
	1993-1998; age 25-29	-0.01	(-0.17,0.16)	0.08	-0.01		0.02	(-0.14,0.18)	0.08	0.01	
	1983-1993; age 30-39	0.13	(-0.00,0.26)	0.07	0.09		0.19	(0.06,0.32)	0.07	0.14	
	1973-1983; age 40-49	0.23	(0.08,0.38)	0.08	0.17		0.22	(0.07,0.37)	0.08	0.16	
	1963-1973; age 50-59	0.34	(0.20,0.49)	0.07	0.25		0.35	(0.21,0.50)	0.07	0.26	

Variable	Category	Secure Flourishing Index Est	95% CI	SE	Est/SD	Global p-value	Flourishing Index Est	95% CI	SE	Est/SD	Global p-value
	1953-1963; age 60-69	0.39	(0.22,0.57)	0.09	0.29		0.26	(0.09,0.43)	0.09	0.19	
	1943-1953; age 70-79	0.56	(0.35,0.77)	0.11	0.41		0.36	(0.16,0.56)	0.10	0.26	
	1943 or earlier; age 80+	0.30	(-0.18,0.79)	0.25	0.22		-0.10	(-0.64,0.43)	0.27	-0.07	
Gender	(Ref: Male)					0.250					0.743
	Female	-0.07	(-0.16,0.02)	0.05	-0.05		0.03	(-0.06,0.12)	0.05	0.02	
	Other	0.37	(-0.76,1.51)	0.58	0.28		-0.27	(-1.43,0.89)	0.59	-0.20	
Religious affiliation	(Ref: No religion/Atheist/Agnostic)					0.239					0.035
	Christianity	0.17	(-0.03,0.36)	0.10	0.12		0.26	(0.06,0.46)	0.10	0.19	
	Collapsed affiliations with prevalence<3%	0.17	(-0.53,0.88)	0.36	0.13		0.26	(-0.54,1.06)	0.41	0.19	
Race/ethnicity	(Ref: Plurality group)					0.439					0.315
	Non-plurality groups	0.03	(-0.06,0.12)	0.05	0.02		0.04	(-0.05,0.13)	0.05	0.03	

Note. N=5776; p < .004 (Bonferroni corrected threshold); Est., estimated effect of childhood predictor on flourishing score; CI, confidence interval; SE, standard error of the estimated effect; Est/SD, a more standardized measure of effect size--estimated effect of flourishing divided by standard deviation of flourishing--leads to the interpretation, for those with the given status (e.g., those with a good/very good relationship with mother compared to those with bad/very bad) are 0.XX standard deviations higher/lower on flourishing; the Global p-value corresponds to the two-sided joint parameter Wald-type test of whether any of the levels' parameters are non-zero, for history of abuse, outsider, relationship wtih mother/father, this is test of whether the estimated effect is non-zero, for multiple-category predictors (age, health, financial status), this is a joint test of whether any of these effects are non-zero. Note the confidence interval of the effect estimate can contradict the reported global p-value (e.g., for the single-category effects of relationship with mother). In such cases, the reported confidence interval is more robust with corrected degrees of freedom from the pooling across multiple imputations, whereas the global p-value is based on a Wald-type test and is less robust to uncertainty attributable to multiple imputation.

Table S12d. Sensitivity to unmeasured confounding of childhood predictors in Mexico (N=5776)

Variable	Category	Secure Flourishing Index		Flourishing Index	
		E-value for Estimate	E-value for 95% CI	E-value for Estimate	E-value for 95% CI
Relationship with mother	(Ref: Very bad/somewhat bad)				
	Very good/somewhat good	1.83	1.45	1.94	1.55
Relationship with father	(Ref: Very bad/somewhat bad)				
	Very good/somewhat good	1.11	1.00	1.16	1.00
Parent marital status	(Ref: Parents married)				
	Divorced	1.33	1.00	1.32	1.00
	Parents were never married	1.21	1.00	1.16	1.00
	One or both parents had died	1.38	1.00	1.33	1.00
Subjective financial status of family growing up	(Ref: Got by)				
	Lived comfortably	1.75	1.55	1.67	1.46
	Found it difficult	1.33	1.00	1.19	1.00
	Found it very difficult	1.50	1.00	1.15	1.00
Abuse	(Ref: No)				
	Yes	1.84	1.61	1.85	1.61
Outsider growing up	(Ref: No)				
	Yes	1.72	1.44	1.78	1.49
Self-rated health growing up	(Ref: Good)				
	Excellent	1.84	1.64	1.87	1.66
	Very good	1.61	1.39	1.52	1.29
	Fair	1.33	1.00	1.39	1.00
	Poor	1.48	1.00	1.50	1.00
Immigration status	(Ref: Born in this country)				
	Born in another country	1.94	1.22	2.11	1.40
Age 12 religious service attendance	(Ref: Never)				
	At least 1/week	1.44	1.10	1.45	1.15
	1-3/month	1.21	1.00	1.15	1.00
	< 1/month	1.23	1.00	1.08	1.00
Year of birth	(Ref: 1998-2005; current age: 18-24)				
	1993-1998; age 25-29	1.08	1.00	1.13	1.00
	1983-1993; age 30-39	1.40	1.00	1.53	1.24

Variable	Category	Secure Flourishing Index		Flourishing Index	
		E-value for Estimate	E-value for 95% CI	E-value for Estimate	E-value for 95% CI
	1973-1983; age 40-49	1.60	1.29	1.59	1.27
	1963-1973; age 50-59	1.82	1.54	1.85	1.56
	1953-1963; age 60-69	1.92	1.57	1.66	1.31
	1943-1953; age 70-79	2.26	1.84	1.87	1.47
	1943 or earlier; age 80+	1.74	1.00	1.35	1.00
Gender	(Ref: Male)				
	Female	1.27	1.00	1.15	1.00
	Other	1.88	1.00	1.69	1.00
Religious affiliation	(Ref: No religion/Atheist/Agnostic)				
	Christianity	1.48	1.00	1.66	1.25
	Collapsed affiliations with prevalence<3%	1.49	1.00	1.67	1.00
Race/ethnicity	(Ref: Plurality group)				
	Non-plurality groups	1.16	1.00	1.19	1.00

Table S13a. Nationally representative descriptive statistics for Nigeria

Characteristic	N = 6,827[1]
**Age group**	
1998-2005; age 18-24	1,533 (22%)
1993-1998; age 25-29	1,193 (17%)
1983-1993; age 30-39	1,943 (28%)
1973-1983; age 40-49	1,059 (16%)
1963-1973; age 50-59	619 (9.1%)
1953-1963; age 60-69	296 (4.3%)
1943-1953; age 70-79	133 (2.0%)
1943 or earlier; age 80+	50 (0.7%)
(Missing)	0 (0%)
**Gender**	
Male	3,371 (49%)
Female	3,456 (51%)
Other	0 (<0.1%)
(Missing)	0 (0%)
**Race/Ethnicity**	
Edo	116 (1.7%)
Efik	48 (0.7%)
Fulani	266 (3.9%)
Hausa	2,342 (34%)
Ibibio	180 (2.6%)
Idoma	61 (0.9%)
Igala	77 (1.1%)
Igbo (Ibo)	1,111 (16%)
Ijaw	110 (1.6%)
Kanuri	31 (0.5%)
Other	1,014 (15%)
Tiv	198 (2.9%)
Urhobo	38 (0.6%)
Yoruba	1,230 (18%)
(Missing)	4 (<0.1%)
**Marital status**	
Married	4,065 (60%)
Separated	117 (1.7%)
Divorced	71 (1.0%)
Widowed	231 (3.4%)
Single, never married	2,289 (34%)
Domestic Partner	12 (0.2%)
(Missing)	42 (0.6%)
**Employment**	
Employed for an employer	699 (10%)
Self-employed	3,898 (57%)
Retired	178 (2.6%)
Student	650 (9.5%)
Homemaker	499 (7.3%)
Unemployed and looking for a job	684 (10%)
None of these/Other	211 (3.1%)
(Missing)	8 (0.1%)
**Religious service attendance**	
More than 1/week	4,049 (59%)
1/week	1,895 (28%)
1-3/month	531 (7.8%)
A few times a year	254 (3.7%)
Never	77 (1.1%)
(Missing)	20 (0.3%)
**Education**	
Up to 8 years	2,575 (38%)
9-15 years	4,120 (60%)

Characteristic	N = 6,827[1]
16+ years	130 (1.9%)
(Missing)	2 (<0.1%)
**Immigration status**	
Born in this country	6,779 (99%)
Born in another country	47 (0.7%)
(Missing)	1 (<0.1%)
**Religious affiliation as an adult (now)**	
Christianity	3,476 (51%)
Islam	3,302 (48%)
Hinduism	0 (0%)
Buddhism	0 (0%)
Judaism	0 (0%)
Sikhism	0 (0%)
Baha'i	0 (0%)
Jainism	0 (0%)
Shinto	1 (<0.1%)
Taoism	0 (0%)
Confucianism	0 (<0.1%)
Primal, Animist, or Folk religion	24 (0.3%)
Spiritism	0 (0%)
Umbanda, Candomble, and other African-derived religions	0 (0%)
Chinese folk/traditional religion	0 (0%)
Some other religion	1 (<0.1%)
No religion/Atheist/Agnostic	15 (0.2%)
(Missing)	9 (0.1%)
**Parent marital status**	
Parents married	5,568 (82%)
Divorced	307 (4.5%)
Parents were never married	335 (4.9%)
One or both parents had died	462 (6.8%)
(Missing)	154 (2.3%)
**Age 12 religious service attendance**	
At least 1/week	5,907 (87%)
1-3/month	600 (8.8%)
<1/month	136 (2.0%)
Never	138 (2.0%)
(Missing)	45 (0.7%)
**Relationship with mother**	
Very good	5,986 (88%)
Somewhat good	648 (9.5%)
Somewhat bad	62 (0.9%)
Very bad	18 (0.3%)
Does not apply	104 (1.5%)
(Missing)	9 (0.1%)
**Relationship with father**	
Very good	5,578 (82%)
Somewhat good	924 (14%)
Somewhat bad	76 (1.1%)
Very bad	43 (0.6%)
Does not apply	177 (2.6%)
(Missing)	29 (0.4%)
**Outsider growing up**	
Yes	669 (9.8%)
No	6,059 (89%)
(Missing)	99 (1.5%)
**Self-reported history of abuse**	
Yes	880 (13%)
No	5,851 (86%)
(Missing)	96 (1.4%)

Characteristic	N = 6,827[1]
**Self-rated health growing up**	
Excellent	2,644 (39%)
Very good	2,613 (38%)
Good	1,152 (17%)
Fair	306 (4.5%)
Poor	98 (1.4%)
(Missing)	14 (0.2%)
**Subjective financial status of family growing up**	
Lived comfortably	2,192 (32%)
Got by	2,381 (35%)
Found it difficult	1,661 (24%)
Found it very difficult	563 (8.3%)
(Missing)	29 (0.4%)
**Religious affiliation at age 12**	
Christianity	3,463 (51%)
Islam	3,314 (49%)
Hinduism	0 (0%)
Buddhism	0 (<0.1%)
Judaism	0 (0%)
Sikhism	0 (0%)
Baha'i	0 (0%)
Jainism	0 (0%)
Shinto	0 (0%)
Taoism	0 (0%)
Confucianism	0 (<0.1%)
Primal, Animist, or Folk religion	17 (0.3%)
Spiritism	0 (0%)
Umbanda, Candomble, and other African-derived religions	0 (0%)
Chinese folk/traditional religion	0 (0%)
Some other religion	0 (0%)
No religion/Atheist/Agnostic	19 (0.3%)
(Missing)	14 (0.2%)

[1]n (%)

Table S13b. Means by demographic category for Nigeria (N=6827)

		Secure Flourishing Index				Flourishing Index			
Variable	Category	Mean	95% CI	SE	Global p-value	Mean	95% CI	SE	Global p-value
Age group	18-24	7.85	(7.76,7.93)	0.04	0.797	7.43	(7.35,7.51)	0.04	0.761
	25-29	7.82	(7.73,7.92)	0.05		7.37	(7.28,7.47)	0.05	
	30-39	7.81	(7.73,7.89)	0.04		7.36	(7.28,7.43)	0.04	
	40-49	7.78	(7.62,7.94)	0.08		7.30	(7.15,7.46)	0.08	
	50-59	7.98	(7.73,8.23)	0.13		7.46	(7.22,7.71)	0.12	
	60-69	7.86	(7.53,8.19)	0.17		7.40	(7.07,7.72)	0.16	
	70-79	7.50	(6.76,8.24)	0.37		7.12	(6.35,7.89)	0.39	
	80 or older	7.39	(6.46,8.32)	0.46		7.07	(6.16,7.97)	0.45	
Gender	Female	7.86	(7.79,7.93)	0.04	< 2e-16	7.41	(7.34,7.48)	0.04	< 2e-16
	Male	7.78	(7.70,7.87)	0.04		7.34	(7.26,7.42)	0.04	
	Other	8.30	*	*		8.17	*	*	
Marital status	Divorced	7.30	(6.85,7.76)	0.23	0.005	6.90	(6.41,7.38)	0.24	0.001
	Domestic partner	7.28	(3.92,10.65)	1.21		6.93	(4.20,9.66)	0.98	
	Married	7.81	(7.72,7.89)	0.04		7.35	(7.27,7.43)	0.04	
	Separated	7.40	(7.04,7.75)	0.18		6.91	(6.58,7.24)	0.17	
	Single/Never been married	7.88	(7.81,7.95)	0.04		7.44	(7.38,7.51)	0.03	
	Widowed	7.96	(7.55,8.38)	0.21		7.44	(7.04,7.83)	0.20	
Employment	Employed for an employer	7.82	(7.70,7.95)	0.06	0.297	7.40	(7.28,7.52)	0.06	0.340
	Homemaker	7.71	(7.53,7.89)	0.09		7.28	(7.11,7.46)	0.09	
	None of these/Other	7.63	(7.30,7.97)	0.17		7.24	(6.90,7.57)	0.17	
	Retired	7.73	(7.25,8.20)	0.24		7.27	(6.80,7.73)	0.24	
	Self-employed	7.88	(7.80,7.96)	0.04		7.41	(7.34,7.49)	0.04	
	Student	7.83	(7.72,7.95)	0.06		7.41	(7.31,7.52)	0.05	
	Unemployed and looking for a job	7.68	(7.48,7.87)	0.10		7.21	(7.02,7.40)	0.10	
Religious service attendance	A few times a year	7.78	(7.50,8.06)	0.14	7.60e-08	7.29	(7.04,7.55)	0.13	6.61e-06
	More than once a week	7.96	(7.88,8.04)	0.04		7.48	(7.40,7.55)	0.04	

Variable	Category	Secure Flourishing Index				Flourishing Index			
		Mean	95% CI	SE	Global p-value	Mean	95% CI	SE	Global p-value
	Never	7.23	(6.72,7.74)	0.26		6.75	(6.25,7.25)	0.25	
	Once a week	7.71	(7.61,7.81)	0.05		7.31	(7.21,7.40)	0.05	
	One to three times a month	7.32	(7.11,7.52)	0.10		6.96	(6.77,7.16)	0.10	
Education	Up to 8	7.79	(7.65,7.92)	0.07	0.483	7.34	(7.22,7.46)	0.06	0.452
	9 to 15	7.76	(7.55,7.96)	0.10		7.29	(7.08,7.51)	0.11	
	16+	7.85	(7.80,7.90)	0.03		7.40	(7.34,7.45)	0.03	
Immigration status	Born in another country	7.62	(7.26,7.97)	0.18	0.229	7.25	(6.87,7.63)	0.19	0.498
	Born in this country	7.83	(7.76,7.89)	0.03		7.37	(7.31,7.43)	0.03	
Religious affiliation	Christianity	7.80	(7.72,7.88)	0.04	3.55e-05	7.33	(7.24,7.41)	0.04	< 2e-16
	Islam	7.85	(7.76,7.94)	0.05		7.43	(7.34,7.51)	0.04	
	No religion/Atheist/ Agnostic	7.61	(6.56,8.66)	0.44		6.84	(5.94,7.74)	0.37	
	Primal, Animist, or Folk religion	7.78	(6.90,8.66)	0.41		7.13	(6.33,7.94)	0.38	
	Shinto	7.50	*	*		7.33	*	*	
	Some other religion	7.60	*	*		7.25	*	*	
	Confucianism	8.00	*	*		7.92	*	*	

Note. N=6827; p < .007 = 0.05/7 (Bonferroni corrected p-value significance threshold); Mean, estimated group mean; CI, confidence interval for the mean within group; SE, complex survey adjusted standard error or the mean; Global p-value, two-tailed Wald-type test of whether there is evidence of any differences in mean scores among groups of a demographic characteristic. *Estimate is not reported due to multiple-imputation and complex survey adjusted degrees of freedom was less than 1.00 leading to insufficient information to provide an estimate of the uncertainty in the estimate. These groups are removed when estimating the global test of mean differences.

Table S13c. Childhood predictors regression analysis results for Nigeria (N=6827)

		Secure Flourishing Index					Flourishing Index				
Variable	Category	Est	95% CI	SE	Est/SD	Global p-value	Est	95% CI	SE	Est/SD	Global p-value
Relationship with mother	(Ref: Very bad/somewhat bad)					0.917					0.728
	Very good/somewhat good	0.01	(-0.34,0.36)	0.18	0.01		-0.06	(-0.41,0.29)	0.18	-0.04	
Relationship with father	(Ref: Very bad/somewhat bad)					0.488					0.805
	Very good/somewhat good	-0.11	(-0.43,0.21)	0.16	-0.08		-0.04	(-0.32,0.25)	0.15	-0.03	
Parent marital status	(Ref: Parents married)					2.63e-09					8.49e-08
	Divorced	-0.18	(-0.45,0.08)	0.13	-0.12		-0.26	(-0.55,0.02)	0.15	-0.19	
	Parents were never married	-0.42	(-0.60,-0.24)	0.09	-0.29		-0.28	(-0.45,-0.11)	0.09	-0.20	
	One or both parents had died	-0.56	(-0.81,-0.31)	0.13	-0.38		-0.60	(-0.86,-0.35)	0.13	-0.43	
Subjective financial status of family growing up	(Ref: Got by)					0.294					0.576
	Lived comfortably	-0.04	(-0.17,0.08)	0.06	-0.03		-0.04	(-0.17,0.10)	0.07	-0.03	
	Found it difficult	-0.05	(-0.18,0.08)	0.07	-0.04		-0.05	(-0.19,0.09)	0.07	-0.04	
	Found it very difficult	-0.23	(-0.46,0.01)	0.12	-0.16		-0.17	(-0.40,0.07)	0.12	-0.12	
Abuse	(Ref: No)					0.003					0.003
	Yes	-0.20	(-0.34,-0.07)	0.07	-0.14		-0.21	(-0.36,-0.07)	0.07	-0.15	

Variable	Category	Secure Flourishing Index					Flourishing Index				
		Est	95% CI	SE	Est/SD	Global p-value	Est	95% CI	SE	Est/SD	Global p-value
Outsider growing up	(Ref: No)					0.363					0.435
	Yes	-0.08	(-0.24,0.09)	0.08	-0.05		-0.07	(-0.24,0.11)	0.09	-0.05	
Self-rated health growing up	(Ref: Good)					0.397					0.371
	Excellent	0.07	(-0.08,0.23)	0.08	0.05		0.08	(-0.08,0.24)	0.08	0.06	
	Very good	-0.03	(-0.18,0.11)	0.07	-0.02		-0.03	(-0.18,0.12)	0.08	-0.02	
	Fair	-0.09	(-0.32,0.14)	0.12	-0.06		-0.11	(-0.35,0.12)	0.12	-0.08	
	Poor	0.02	(-0.44,0.48)	0.23	0.02		-0.02	(-0.42,0.39)	0.21	-0.01	
Immigration status	(Ref: Born in this country)					0.777					0.476
	Born in another country	-0.05	(-0.41,0.30)	0.18	-0.03		-0.11	(-0.42,0.20)	0.16	-0.08	
Age 12 religious service attendance	(Ref: Never)					0.072					0.028
	At least 1/week	0.32	(0.01,0.63)	0.16	0.22		0.32	(-0.01,0.66)	0.17	0.23	
	1-3/month	0.21	(-0.14,0.57)	0.18	0.15		0.21	(-0.16,0.59)	0.19	0.15	
	< 1/month	0.06	(-0.34,0.47)	0.21	0.04		-0.04	(-0.45,0.38)	0.21	-0.03	
Year of birth	(Ref: 1998-2005; current age: 18-24)					0.800					0.637
	1993-1998; age 25-29	-0.03	(-0.15,0.08)	0.06	-0.02		-0.00	(-0.12,0.12)	0.06	-0.00	
	1983-1993; age 30-39	-0.06	(-0.17,0.04)	0.05	-0.04		-0.03	(-0.14,0.08)	0.06	-0.02	
	1973-1983; age 40-49	-0.07	(-0.24,0.10)	0.09	-0.05		-0.02	(-0.19,0.16)	0.09	-0.01	
	1963-1973; age 50-59	0.10	(-0.15,0.36)	0.13	0.07		0.20	(-0.06,0.46)	0.13	0.14	

Variable	Category	Secure Flourishing Index					Flourishing Index				
		Est	95% CI	SE	Est/SD	Global p-value	Est	95% CI	SE	Est/SD	Global p-value
	1953-1963; age 60-69	0.02	(-0.30,0.34)	0.16	0.02		0.06	(-0.28,0.39)	0.17	0.04	
	1943-1953; age 70-79	-0.22	(-0.98,0.53)	0.38	-0.15		-0.25	(-0.97,0.47)	0.37	-0.18	
	1943 or earlier; age 80+	-0.33	(-1.12,0.47)	0.41	-0.22		-0.46	(-1.30,0.38)	0.43	-0.33	
Gender	(Ref: Male)					< 2e-16					3.68e-08
	Female	0.05	(-0.04,0.14)	0.04	0.04		0.05	(-0.03,0.14)	0.04	0.04	
	Other	0.96	(0.77,1.15)	0.10	0.66		0.64	(0.43,0.84)	0.10	0.46	
Religious affiliation	(Ref: Christianity)					0.632					0.944
	Islam	0.01	(-0.14,0.16)	0.08	0.01		-0.02	(-0.17,0.14)	0.08	-0.01	
	Collapsed affiliations with prevalence<3%	-0.33	(-1.05,0.38)	0.36	-0.23		0.06	(-0.64,0.77)	0.36	0.05	
Race/ethnicity	(Ref: Plurality group)					0.189					0.358
	Non-plurality groups	-0.10	(-0.24,0.05)	0.07	-0.07		-0.08	(-0.24,0.09)	0.08	-0.05	

Note. N=6827; p < .004 (Bonferroni corrected threshold); Est., estimated effect of childhood predictor on flourishing score; CI, confidence interval; SE, standard error of the estimated effect; Est/SD, a more standardized measure of effect size--estimated effect of flourishing divided by standard deviation of flourishing--leads to the interpretation, for those with the given status (e.g., those with a good/very good relationship with mother compared to those with bad/very bad) are 0.XX standard deviations higher/lower on flourishing; the Global p-value corresponds to the two-sided joint parameter Wald-type test of whether any of the levels' parameters are non-zero, for history of abuse, outsider, relationship wtih mother/father, this is test of whether the estimated effect is non-zero, for multiple-category predictors (age, health, financial status), this is a joint test of whether any of these effects are non-zero. Note the confidence interval of the effect estimate can contradict the reported global p-value (e.g., for the single-category effects of relationship with mother). In such cases, the reported confidence interval is more robust with corrected degrees of freedom from the pooling across multiple imputations, whereas the global p-value is based on a Wald-type test and is less robust to uncertainty attributable to multiple imputation.

Table S13d. Sensitivity to unmeasured confounding of childhood predictors in Nigeria (N=6827)

Variable	Category	Secure Flourishing Index		Flourishing Index	
		E-value for Estimate	E-value for 95% CI	E-value for Estimate	E-value for 95% CI
Relationship with mother	(Ref: Very bad/somewhat bad)				
	Very good/somewhat good	1.09	1.00	1.23	1.00
Relationship with father	(Ref: Very bad/somewhat bad)				
	Very good/somewhat good	1.36	1.00	1.17	1.00
Parent marital status	(Ref: Parents married)				
	Divorced	1.50	1.00	1.64	1.00
	Parents were never married	1.95	1.61	1.67	1.35
	One or both parents had died	2.23	1.75	2.28	1.79
Subjective financial status of family growing up	(Ref: Got by)				
	Lived comfortably	1.20	1.00	1.18	1.00
	Found it difficult	1.22	1.00	1.22	1.00
	Found it very difficult	1.59	1.00	1.46	1.00
Abuse	(Ref: No)				
	Yes	1.54	1.27	1.55	1.26
Outsider growing up	(Ref: No)				
	Yes	1.28	1.00	1.25	1.00
Self-rated health growing up	(Ref: Good)				
	Excellent	1.28	1.00	1.29	1.00
	Very good	1.17	1.00	1.17	1.00
	Fair	1.32	1.00	1.35	1.00
	Poor	1.14	1.00	1.12	1.00
Immigration status	(Ref: Born in this country)				
	Born in another country	1.22	1.00	1.35	1.00
Age 12 religious service attendance	(Ref: Never)				
	At least 1/week	1.77	1.10	1.74	1.00
	1-3/month	1.56	1.00	1.55	1.00
	< 1/month	1.25	1.00	1.18	1.00
Year of birth	(Ref: 1998-2005; current age: 18-24)				
	1993-1998; age 25-29	1.18	1.00	1.01	1.00
	1983-1993; age 30-39	1.25	1.00	1.16	1.00

Variable	Category	Secure Flourishing Index		Flourishing Index	
		E-value for Estimate	E-value for 95% CI	E-value for Estimate	E-value for 95% CI
	1973-1983; age 40-49	1.27	1.00	1.12	1.00
	1963-1973; age 50-59	1.35	1.00	1.52	1.00
	1953-1963; age 60-69	1.14	1.00	1.23	1.00
	1943-1953; age 70-79	1.58	1.00	1.62	1.00
	1943 or earlier; age 80+	1.78	1.00	1.99	1.00
Gender	(Ref: Male)				
	Female	1.22	1.00	1.22	1.00
	Other	3.15	2.69	2.34	1.94
Religious affiliation	(Ref: Christianity)				
	Islam	1.09	1.00	1.11	1.00
	Collapsed affiliations with prevalence<3%	1.79	1.00	1.25	1.00
Race/ethnicity	(Ref: Plurality group)				
	Non-plurality groups	1.33	1.00	1.28	1.00

Table S14a. Nationally representative descriptive statistics for Philippines

Characteristic	N = 5,292[1]
**Age group**	
1998-2005; age 18-24	1,073 (20%)
1993-1998; age 25-29	695 (13%)
1983-1993; age 30-39	1,160 (22%)
1973-1983; age 40-49	972 (18%)
1963-1973; age 50-59	732 (14%)
1953-1963; age 60-69	495 (9.4%)
1943-1953; age 70-79	143 (2.7%)
1943 or earlier; age 80+	23 (0.4%)
(Missing)	0 (0%)
**Gender**	
Male	2,625 (50%)
Female	2,643 (50%)
Other	13 (0.2%)
(Missing)	11 (0.2%)
**Race/Ethnicity**	
Aeta	1 (<0.1%)
Badjao	2 (<0.1%)
Bicolano/Bikolano	300 (5.7%)
Cebuano	656 (12%)
Chinese-Filipino	3 (<0.1%)
Igorot	42 (0.8%)
Ilocano/Ilokano	429 (8.1%)
Ilonggo/Hiligaynon	428 (8.1%)
Kapampangan	107 (2.0%)
Maguindanaoan	84 (1.6%)
Mangyan	2 (<0.1%)
Maranao	39 (0.7%)
Masbateno	54 (1.0%)
Other	244 (4.6%)
Pangasinense	107 (2.0%)
Tagalog	1,691 (32%)
Tausug	94 (1.8%)
Visayan/Bisaya	739 (14%)
Waray	216 (4.1%)
Zamboangueno	51 (1.0%)
(Missing)	3 (<0.1%)
**Marital status**	
Married	2,385 (45%)
Separated	249 (4.7%)
Divorced	9 (0.2%)
Widowed	274 (5.2%)
Single, never married	1,206 (23%)
Domestic Partner	1,152 (22%)
(Missing)	16 (0.3%)
**Employment**	
Employed for an employer	1,350 (26%)
Self-employed	1,379 (26%)
Retired	158 (3.0%)
Student	585 (11%)
Homemaker	1,049 (20%)
Unemployed and looking for a job	658 (12%)
None of these/Other	113 (2.1%)
(Missing)	0 (0%)
**Religious service attendance**	
More than 1/week	844 (16%)
1/week	1,929 (36%)
1-3/month	1,374 (26%)

Characteristic	N = 5,292[1]
A few times a year	929 (18%)
Never	210 (4.0%)
(Missing)	6 (0.1%)
**Education**	
Up to 8 years	1,188 (22%)
9-15 years	3,722 (70%)
16+ years	381 (7.2%)
(Missing)	1 (<0.1%)
**Immigration status**	
Born in this country	5,284 (100%)
Born in another country	8 (0.1%)
(Missing)	0 (0%)
**Religious affiliation as an adult (now)**	
Christianity	4,914 (93%)
Islam	297 (5.6%)
Hinduism	0 (0%)
Buddhism	4 (<0.1%)
Judaism	4 (<0.1%)
Sikhism	0 (0%)
Baha'i	1 (<0.1%)
Jainism	0 (0%)
Shinto	0 (0%)
Taoism	0 (0%)
Confucianism	0 (0%)
Primal, Animist, or Folk religion	5 (<0.1%)
Spiritism	0 (0%)
Umbanda, Candomble, and other African-derived religions	0 (0%)
Chinese folk/traditional religion	0 (0%)
Some other religion	35 (0.7%)
No religion/Atheist/Agnostic	23 (0.4%)
(Missing)	9 (0.2%)
**Parent marital status**	
Parents married	4,575 (86%)
Divorced	64 (1.2%)
Parents were never married	517 (9.8%)
One or both parents had died	51 (1.0%)
(Missing)	85 (1.6%)
**Age 12 religious service attendance**	
At least 1/week	2,453 (46%)
1-3/month	1,699 (32%)
<1/month	892 (17%)
Never	201 (3.8%)
(Missing)	47 (0.9%)
**Relationship with mother**	
Very good	3,333 (63%)
Somewhat good	1,703 (32%)
Somewhat bad	124 (2.3%)
Very bad	39 (0.7%)
Does not apply	59 (1.1%)
(Missing)	35 (0.7%)
**Relationship with father**	
Very good	3,443 (65%)
Somewhat good	1,429 (27%)
Somewhat bad	159 (3.0%)
Very bad	58 (1.1%)
Does not apply	108 (2.0%)
(Missing)	95 (1.8%)
**Outsider growing up**	
Yes	395 (7.5%)

Characteristic	N = 5,292[1]
No	4,884 (92%)
(Missing)	13 (0.2%)
**Self-reported history of abuse**	
Yes	420 (7.9%)
No	4,837 (91%)
(Missing)	35 (0.7%)
**Self-rated health growing up**	
Excellent	1,041 (20%)
Very good	559 (11%)
Good	2,174 (41%)
Fair	1,246 (24%)
Poor	272 (5.1%)
(Missing)	0 (<0.1%)
**Subjective financial status of family growing up**	
Lived comfortably	937 (18%)
Got by	3,006 (57%)
Found it difficult	1,055 (20%)
Found it very difficult	291 (5.5%)
(Missing)	3 (<0.1%)
**Religious affiliation at age 12**	
Christianity	4,968 (94%)
Islam	276 (5.2%)
Hinduism	0 (0%)
Buddhism	1 (<0.1%)
Judaism	0 (0%)
Sikhism	4 (<0.1%)
Baha'i	1 (<0.1%)
Jainism	0 (0%)
Shinto	0 (0%)
Taoism	0 (0%)
Confucianism	0 (0%)
Primal, Animist, or Folk religion	14 (0.3%)
Spiritism	0 (0%)
Umbanda, Candomble, and other African-derived religions	0 (0%)
Chinese folk/traditional religion	0 (0%)
Some other religion	9 (0.2%)
No religion/Atheist/Agnostic	9 (0.2%)
(Missing)	11 (0.2%)

[1]n (%)

Table S14b. Means by demographic category for Philippines (N=5292)

Variable	Category	Secure Flourishing Index				Flourishing Index			
		Mean	95% CI	SE	Global p-value	Mean	95% CI	SE	Global p-value
Age group	18-24	8.14	(8.04,8.25)	0.05	0.003	7.69	(7.59,7.80)	0.05	0.269
	25-29	8.22	(8.06,8.38)	0.08		7.76	(7.61,7.91)	0.08	
	30-39	8.25	(8.16,8.33)	0.04		7.81	(7.72,7.90)	0.05	
	40-49	8.04	(7.94,8.14)	0.05		7.66	(7.57,7.75)	0.05	
	50-59	7.96	(7.81,8.12)	0.08		7.60	(7.45,7.76)	0.08	
	60-69	8.00	(7.82,8.19)	0.09		7.69	(7.50,7.88)	0.10	
	70-79	7.91	(7.60,8.22)	0.16		7.66	(7.32,8.00)	0.17	
	80 or older	7.56	(6.78,8.33)	0.36		7.56	(6.71,8.41)	0.39	
Gender	Female	8.14	(8.08,8.19)	0.03	0.073	7.69	(7.64,7.75)	0.03	0.003
	Male	8.09	(8.01,8.17)	0.04		7.73	(7.65,7.81)	0.04	
	Other	7.30	(6.41,8.20)	0.38		6.70	(6.01,7.39)	0.29	
Marital status	Divorced	7.37	(4.87,9.88)	0.52	5.73e-06	6.87	(4.83,8.91)	0.42	6.77e-04
	Domestic partner	8.25	(8.17,8.34)	0.04		7.78	(7.69,7.86)	0.04	
	Married	8.15	(8.07,8.22)	0.04		7.75	(7.67,7.82)	0.04	
	Separated	7.71	(7.50,7.92)	0.11		7.36	(7.16,7.57)	0.11	
	Single/Never been married	7.99	(7.88,8.10)	0.06		7.61	(7.51,7.72)	0.05	
	Widowed	8.10	(7.91,8.29)	0.10		7.83	(7.64,8.03)	0.10	
Employment	Employed for an employer	8.21	(8.12,8.31)	0.05	0.073	7.84	(7.74,7.93)	0.05	1.98e-05
	Homemaker	8.08	(7.98,8.18)	0.05		7.61	(7.51,7.71)	0.05	
	None of these/Other	7.80	(7.48,8.13)	0.16		7.35	(7.06,7.64)	0.15	
	Retired	8.19	(7.92,8.46)	0.14		8.00	(7.72,8.27)	0.14	
	Self-employed	8.13	(8.03,8.22)	0.05		7.78	(7.69,7.88)	0.05	
	Student	8.07	(7.96,8.19)	0.06		7.61	(7.50,7.73)	0.06	
	Unemployed and looking for a job	7.98	(7.82,8.15)	0.08		7.52	(7.36,7.68)	0.08	
Religious service attendance	A few times a year	7.90	(7.79,8.01)	0.06	4.93e-09	7.48	(7.38,7.59)	0.05	1.02e-08
	More than once a week	8.25	(8.12,8.38)	0.07		7.85	(7.72,7.98)	0.07	

Variable	Category	Secure Flourishing Index				Flourishing Index			
		Mean	95% CI	SE	Global p-value	Mean	95% CI	SE	Global p-value
	Never	7.38	(7.05,7.71)	0.17		7.06	(6.73,7.38)	0.16	
	Once a week	8.23	(8.16,8.31)	0.04		7.82	(7.74,7.89)	0.04	
	One to three times a month	8.11	(8.02,8.19)	0.04		7.71	(7.63,7.80)	0.04	
Education	Up to 8	8.02	(7.90,8.13)	0.06	0.144	7.59	(7.48,7.71)	0.06	0.018
	9 to 15	8.20	(8.03,8.37)	0.09		7.89	(7.72,8.06)	0.09	
	16+	8.13	(8.08,8.19)	0.03		7.73	(7.67,7.78)	0.03	
Immigration status	Born in another country	7.11	(5.47,8.74)	0.57	0.080	6.67	(5.15,8.19)	0.53	0.052
	Born in this country	8.11	(8.06,8.16)	0.03		7.71	(7.66,7.76)	0.03	
Religious affiliation	Baha'i	8.00	*	*	< 2e-16	7.92	*	*	< 2e-16
	Buddhism	8.82	*	*		8.67	*	*	
	Christianity	8.11	(8.06,8.17)	0.03		7.71	(7.66,7.76)	0.03	
	Islam	8.05	(7.81,8.28)	0.12		7.59	(7.35,7.83)	0.12	
	Judaism	8.81	*	*		7.76	*	*	
	No religion/Atheist/ Agnostic	8.33	(7.51,9.15)	0.38		8.04	(7.14,8.95)	0.41	
	Primal, Animist, or Folk religion	5.57	(4.26,6.88)	0.65		5.46	(4.28,6.64)	0.59	
	Some other religion	8.14	(7.68,8.60)	0.22		7.80	(7.37,8.23)	0.21	

Note. N=5292; $p < .007 = 0.05/7$ (Bonferroni corrected p-value significance threshold); Mean, estimated group mean; CI, confidence interval for the mean within group; SE, complex survey adjusted standard error or the mean; Global p-value, two-tailed Wald-type test of whether there is evidence of any differences in mean scores among groups of a demographic characteristic. *Estimate is not reported due to multiple-imputation and complex survey adjusted degrees of freedom was less than 1.00 leading to insufficient information to provide an estimate of the uncertainty in the estimate. These groups are removed when estimating the global test of mean differences.

Table S14c. Childhood predictors regression analysis results for Philippines (N=5292)

		Secure Flourishing Index					Flourishing Index				
Variable	Category	Est	95% CI	SE	Est/SD	Global p-value	Est	95% CI	SE	Est/SD	Global p-value
Relationship with mother	(Ref: Very bad/somewhat bad)					0.196					0.160
	Very good/somewhat good	0.17	(-0.09,0.43)	0.13	0.12		0.20	(-0.08,0.49)	0.15	0.14	
Relationship with father	(Ref: Very bad/somewhat bad)					0.663					0.780
	Very good/somewhat good	-0.05	(-0.27,0.18)	0.12	-0.03		-0.03	(-0.26,0.20)	0.12	-0.02	
Parent marital status	(Ref: Parents married)					0.034					0.160
	Divorced	-0.30	(-0.58,-0.02)	0.14	-0.21		-0.14	(-0.42,0.15)	0.14	-0.10	
	Parents were never married	-0.03	(-0.18,0.13)	0.08	-0.02		0.03	(-0.12,0.19)	0.08	0.02	
	One or both parents had died	0.40	(-0.04,0.84)	0.22	0.28		0.43	(-0.00,0.86)	0.22	0.30	
Subjective financial status of family growing up	(Ref: Got by)					7.03e-11					1.52e-06
	Lived comfortably	0.29	(0.16,0.42)	0.07	0.20		0.27	(0.13,0.40)	0.07	0.19	
	Found it difficult	-0.21	(-0.34,-0.09)	0.06	-0.15		-0.13	(-0.26,-0.00)	0.07	-0.09	
	Found it very difficult	-0.28	(-0.51,-0.06)	0.11	-0.20		-0.12	(-0.35,0.11)	0.12	-0.09	
Abuse	(Ref: No)					0.005					0.004
	Yes	-0.30	(-0.52,-0.09)	0.11	-0.21		-0.31	(-0.52,-0.09)	0.11	-0.22	

Variable	Category	Secure Flourishing Index					Flourishing Index				
		Est	95% CI	SE	Est/SD	Global p-value	Est	95% CI	SE	Est/SD	Global p-value
Outsider growing up	(Ref: No)					0.044					0.046
	Yes	-0.22	(-0.43,-0.01)	0.11	-0.15		-0.24	(-0.48,-0.00)	0.12	-0.17	
Self-rated health growing up	(Ref: Good)					5.00e-10					7.08e-10
	Excellent	0.10	(-0.02,0.23)	0.06	0.07		0.15	(0.02,0.28)	0.06	0.11	
	Very good	-0.00	(-0.16,0.15)	0.08	-0.00		0.02	(-0.12,0.17)	0.07	0.02	
	Fair	-0.31	(-0.44,-0.19)	0.07	-0.22		-0.29	(-0.42,-0.16)	0.07	-0.20	
	Poor	-0.58	(-0.83,-0.32)	0.13	-0.40		-0.57	(-0.84,-0.30)	0.14	-0.40	
Immigration status	(Ref: Born in this country)					0.046					0.075
	Born in another country	-1.04	(-2.06,-0.02)	0.52	-0.72		-1.06	(-2.22,0.10)	0.59	-0.74	
Age 12 religious service attendance	(Ref: Never)					0.601					0.583
	At least 1/week	0.21	(-0.16,0.57)	0.19	0.14		0.26	(-0.13,0.65)	0.20	0.18	
	1-3/month	0.17	(-0.20,0.54)	0.19	0.12		0.23	(-0.17,0.62)	0.20	0.16	
	< 1/month	0.22	(-0.16,0.59)	0.19	0.15		0.25	(-0.15,0.65)	0.20	0.17	
Year of birth	(Ref: 1998-2005; current age: 18-24)					0.259					0.024
	1993-1998; age 25-29	0.07	(-0.09,0.23)	0.08	0.05		0.08	(-0.09,0.25)	0.09	0.06	
	1983-1993; age 30-39	0.17	(0.05,0.29)	0.06	0.12		0.15	(0.03,0.28)	0.06	0.11	
	1973-1983; age 40-49	0.04	(-0.09,0.16)	0.07	0.02		-0.04	(-0.18,0.10)	0.07	-0.03	
	1963-1973; age 50-59	0.02	(-0.16,0.20)	0.09	0.01		-0.08	(-0.26,0.10)	0.09	-0.05	

Table S14d. Sensitivity to unmeasured confounding of childhood predictors in Philippines (N=5292)

		Secure Flourishing Index		Flourishing Index	
Variable	Category	E-value for Estimate	E-value for 95% CI	E-value for Estimate	E-value for 95% CI
Relationship with mother	(Ref: Very bad/somewhat bad)				
	Very good/somewhat good	1.47	1.00	1.53	1.00
Relationship with father	(Ref: Very bad/somewhat bad)				
	Very good/somewhat good	1.21	1.00	1.16	1.00
Parent marital status	(Ref: Parents married)				
	Divorced	1.73	1.14	1.40	1.00
	Parents were never married	1.15	1.00	1.17	1.00
	One or both parents had died	1.92	1.00	1.94	1.00
Subjective financial status of family growing up	(Ref: Got by)				
	Lived comfortably	1.70	1.45	1.65	1.40
	Found it difficult	1.56	1.31	1.39	1.05
	Found it very difficult	1.69	1.24	1.38	1.00
Abuse	(Ref: No)				
	Yes	1.73	1.31	1.72	1.32
Outsider growing up	(Ref: No)				
	Yes	1.56	1.06	1.61	1.06
Self-rated health growing up	(Ref: Good)				
	Excellent	1.34	1.00	1.43	1.14
	Very good	1.05	1.00	1.14	1.00
	Fair	1.75	1.50	1.69	1.45
	Poor	2.25	1.75	2.23	1.72
Immigration status	(Ref: Born in this country)				
	Born in another country	3.31	1.13	3.31	1.00
Age 12 religious service attendance	(Ref: Never)				
	At least 1/week	1.55	1.00	1.63	1.00
	1-3/month	1.48	1.00	1.58	1.00
	< 1/month	1.56	1.00	1.61	1.00
Year of birth	(Ref: 1998-2005; current age: 18-24)				
	1993-1998; age 25-29	1.27	1.00	1.28	1.00
	1983-1993; age 30-39	1.47	1.20	1.44	1.17

Variable	Category	Secure Flourishing Index		Flourishing Index	
		E-value for Estimate	E-value for 95% CI	E-value for Estimate	E-value for 95% CI
	1973-1983; age 40-49	1.18	1.00	1.18	1.00
	1963-1973; age 50-59	1.12	1.00	1.28	1.00
	1953-1963; age 60-69	1.39	1.00	1.12	1.00
	1943-1953; age 70-79	1.30	1.00	1.35	1.00
	1943 or earlier; age 80+	1.14	1.00	2.08	1.00
Gender	(Ref: Male)				
	Female	1.20	1.00	1.18	1.00
	Other	3.27	2.00	2.74	1.32
Religious affiliation	(Ref: Christianity)				
	Islam	1.24	1.00	1.12	1.00
	Collapsed affiliations with prevalence<3%	1.56	1.00	1.25	1.00
Race/ethnicity	(Ref: Plurality group)				
	Non-plurality groups	1.44	1.21	1.37	1.10

Table S15a. Nationally representative descriptive statistics for Poland

**Characteristic**	**N = 10,389**[1]
**Age group**	
1998-2005; age 18-24	955 (9.2%)
1993-1998; age 25-29	761 (7.3%)
1983-1993; age 30-39	2,159 (21%)
1973-1983; age 40-49	1,956 (19%)
1963-1973; age 50-59	1,670 (16%)
1953-1963; age 60-69	1,909 (18%)
1943-1953; age 70-79	833 (8.0%)
1943 or earlier; age 80+	145 (1.4%)
(Missing)	1 (<0.1%)
**Gender**	
Male	4,974 (48%)
Female	5,387 (52%)
Other	3 (<0.1%)
(Missing)	26 (0.2%)
**Race/Ethnicity**	
Belarussian	2 (<0.1%)
German	4 (<0.1%)
Kashubians	3 (<0.1%)
Other	4 (<0.1%)
Polish	10,309 (99%)
Silesia	14 (0.1%)
Ukrainian	38 (0.4%)
(Missing)	14 (0.1%)
**Marital status**	
Married	6,065 (58%)
Separated	111 (1.1%)
Divorced	529 (5.1%)
Widowed	990 (9.5%)
Single, never married	1,811 (17%)
Domestic Partner	504 (4.8%)
(Missing)	379 (3.6%)
**Employment**	
Employed for an employer	5,837 (56%)
Self-employed	686 (6.6%)
Retired	2,434 (23%)
Student	515 (5.0%)
Homemaker	338 (3.3%)
Unemployed and looking for a job	284 (2.7%)
None of these/Other	169 (1.6%)
(Missing)	126 (1.2%)
**Religious service attendance**	
More than 1/week	305 (2.9%)
1/week	3,263 (31%)
1-3/month	2,081 (20%)
A few times a year	3,064 (29%)
Never	1,597 (15%)
(Missing)	78 (0.8%)
**Education**	
Up to 8 years	1,238 (12%)
9-15 years	6,130 (59%)
16+ years	3,020 (29%)
(Missing)	1 (<0.1%)
**Immigration status**	
Born in this country	10,258 (99%)
Born in another country	108 (1.0%)
(Missing)	23 (0.2%)
**Religious affiliation as an adult (now)**	

**Characteristic**	**N = 10,389**[1]
Christianity	9,378 (90%)
Islam	2 (<0.1%)
Hinduism	0 (0%)
Buddhism	2 (<0.1%)
Judaism	0 (0%)
Sikhism	1 (<0.1%)
Baha'i	0 (0%)
Jainism	3 (<0.1%)
Shinto	1 (<0.1%)
Taoism	0 (0%)
Confucianism	0 (0%)
Primal, Animist, or Folk religion	11 (0.1%)
Spiritism	0 (0%)
Umbanda, Candomble, and other African-derived religions	0 (0%)
Chinese folk/traditional religion	0 (0%)
Some other religion	0 (0%)
No religion/Atheist/Agnostic	942 (9.1%)
(Missing)	50 (0.5%)
**Parent marital status**	
Parents married	8,972 (86%)
Divorced	587 (5.7%)
Parents were never married	193 (1.9%)
One or both parents had died	313 (3.0%)
(Missing)	324 (3.1%)
**Age 12 religious service attendance**	
At least 1/week	4,751 (46%)
1-3/month	2,689 (26%)
<1/month	2,161 (21%)
Never	354 (3.4%)
(Missing)	434 (4.2%)
**Relationship with mother**	
Very good	4,879 (47%)
Somewhat good	4,973 (48%)
Somewhat bad	285 (2.7%)
Very bad	58 (0.6%)
Does not apply	80 (0.8%)
(Missing)	112 (1.1%)
**Relationship with father**	
Very good	4,231 (41%)
Somewhat good	4,984 (48%)
Somewhat bad	516 (5.0%)
Very bad	78 (0.7%)
Does not apply	407 (3.9%)
(Missing)	173 (1.7%)
**Outsider growing up**	
Yes	490 (4.7%)
No	9,615 (93%)
(Missing)	284 (2.7%)
**Self-reported history of abuse**	
Yes	325 (3.1%)
No	10,009 (96%)
(Missing)	55 (0.5%)
**Self-rated health growing up**	
Excellent	2,676 (26%)
Very good	5,371 (52%)
Good	1,779 (17%)
Fair	406 (3.9%)
Poor	123 (1.2%)
(Missing)	34 (0.3%)

Characteristic	N = 10,389[1]
**Subjective financial status of family growing up**	
Lived comfortably	1,384 (13%)
Got by	6,257 (60%)
Found it difficult	2,133 (21%)
Found it very difficult	509 (4.9%)
(Missing)	106 (1.0%)
**Religious affiliation at age 12**	
Christianity	9,861 (95%)
Islam	3 (<0.1%)
Hinduism	0 (0%)
Buddhism	2 (<0.1%)
Judaism	0 (0%)
Sikhism	1 (<0.1%)
Baha'i	0 (0%)
Jainism	0 (0%)
Shinto	0 (0%)
Taoism	0 (0%)
Confucianism	0 (0%)
Primal, Animist, or Folk religion	5 (<0.1%)
Spiritism	0 (0%)
Umbanda, Candomble, and other African-derived religions	0 (0%)
Chinese folk/traditional religion	0 (0%)
Some other religion	0 (0%)
No religion/Atheist/Agnostic	482 (4.6%)
(Missing)	35 (0.3%)

[1]n (%)

Table S15b. Means by demographic category for Poland (N=10389)

Variable	Category	Secure Flourishing Index				Flourishing Index			
		Mean	95% CI	SE	Global p-value	Mean	95% CI	SE	Global p-value
Age group	18-24	7.87	(7.72,8.02)	0.08	5.08e-04	7.87	(7.71,8.02)	0.08	4.50e-05
	25-29	7.75	(7.63,7.86)	0.06		7.65	(7.54,7.77)	0.06	
	30-39	7.70	(7.59,7.82)	0.06		7.60	(7.48,7.71)	0.06	
	40-49	7.58	(7.44,7.72)	0.07		7.50	(7.37,7.64)	0.07	
	50-59	7.52	(7.40,7.65)	0.06		7.44	(7.31,7.56)	0.06	
	60-69	7.62	(7.49,7.74)	0.06		7.52	(7.40,7.65)	0.06	
	70-79	7.53	(7.31,7.74)	0.11		7.48	(7.27,7.70)	0.11	
	80 or older	7.23	(6.77,7.68)	0.23		7.08	(6.58,7.58)	0.25	
Gender	Female	7.65	(7.56,7.74)	0.05	0.084	7.54	(7.45,7.63)	0.05	0.086
	Male	7.62	(7.51,7.72)	0.05		7.56	(7.46,7.67)	0.05	
	Other	5.88	*	*		5.76	*	*	
Marital status	Divorced	7.15	(6.92,7.37)	0.11	2.91e-08	6.97	(6.73,7.20)	0.12	4.26e-12
	Domestic partner	7.59	(7.43,7.75)	0.08		7.48	(7.32,7.64)	0.08	
	Married	7.74	(7.65,7.84)	0.05		7.68	(7.59,7.78)	0.05	
	Separated	7.32	(7.04,7.60)	0.14		7.12	(6.86,7.37)	0.13	
	Single/Never been married	7.57	(7.44,7.71)	0.07		7.52	(7.37,7.66)	0.07	
	Widowed	7.38	(7.22,7.54)	0.08		7.22	(7.05,7.39)	0.09	
Employment	Employed for an employer	7.68	(7.58,7.78)	0.05	3.23e-07	7.59	(7.49,7.68)	0.05	3.39e-10
	Homemaker	7.59	(7.30,7.88)	0.15		7.45	(7.16,7.75)	0.15	
	None of these/Other	7.08	(6.68,7.47)	0.20		6.92	(6.54,7.31)	0.20	
	Retired	7.54	(7.41,7.66)	0.06		7.46	(7.33,7.59)	0.07	
	Self-employed	7.72	(7.59,7.85)	0.06		7.68	(7.55,7.81)	0.07	
	Student	8.07	(7.88,8.27)	0.10		8.12	(7.92,8.32)	0.10	
	Unemployed and looking for a job	6.91	(6.43,7.39)	0.24		6.82	(6.38,7.27)	0.23	
Religious service attendance	A few times a year	7.63	(7.52,7.74)	0.06	3.69e-05	7.52	(7.41,7.62)	0.05	1.02e-06
	More than once a week	7.71	(7.34,8.08)	0.19		7.50	(7.10,7.91)	0.21	

Variable	Category	Secure Flourishing Index				Flourishing Index			
		Mean	95% CI	SE	Global p-value	Mean	95% CI	SE	Global p-value
	Never	7.36	(7.20,7.51)	0.08		7.26	(7.11,7.41)	0.08	
	Once a week	7.78	(7.67,7.88)	0.05		7.75	(7.64,7.86)	0.05	
	One to three times a month	7.61	(7.46,7.76)	0.07		7.53	(7.39,7.67)	0.07	
Education	Up to 8	7.11	(6.80,7.42)	0.16	6.33e-06	7.01	(6.71,7.31)	0.15	2.01e-06
	9 to 15	7.80	(7.72,7.88)	0.04		7.72	(7.64,7.80)	0.04	
	16+	7.66	(7.57,7.74)	0.04		7.58	(7.49,7.66)	0.04	
Immigration status	Born in another country	7.01	(6.58,7.43)	0.21	0.003	6.84	(6.47,7.21)	0.19	1.43e-04
	Born in this country	7.64	(7.55,7.73)	0.04		7.56	(7.47,7.65)	0.04	
Religious affiliation	Buddhism	5.73	*	*	< 2e-16	5.58	*	*	< 2e-16
	Christianity	7.64	(7.55,7.73)	0.05		7.55	(7.46,7.65)	0.05	
	Islam	8.66	*	*		8.39	*	*	
	No religion/Atheist/ Agnostic	7.59	(7.46,7.72)	0.06		7.55	(7.41,7.69)	0.07	
	Primal, Animist, or Folk religion	6.67	(4.98,8.37)	0.27		6.55	(4.68,8.43)	0.30	
	Shinto	5.70	*	*		5.25	*	*	
	Sikhism	5.40	*	*		5.50	*	*	
	Jainism	8.30	*	*		8.17	*	*	

Note. N=10389; p < .007 = 0.05/7 (Bonferroni corrected p-value significance threshold); Mean, estimated group mean; CI, confidence interval for the mean within group; SE, complex survey adjusted standard error or the mean; Global p-value, two-tailed Wald-type test of whether there is evidence of any differences in mean scores among groups of a demographic characteristic. *Estimate is not reported due to multiple-imputation and complex survey adjusted degrees of freedom was less than 1.00 leading to insufficient information to provide an estimate of the uncertainty in the estimate. These groups are removed when estimating the global test of mean differences.

Table S15c. Childhood predictors regression analysis results for Poland (N=10389)

		Secure Flourishing Index					Flourishing Index				
Variable	Category	Est	95% CI	SE	Est/SD	Global p-value	Est	95% CI	SE	Est/SD	Global p-value
Relationship with mother	(Ref: Very bad/somewhat bad)					0.200					0.172
	Very good/somewhat good	0.20	(-0.12,0.53)	0.16	0.16		0.21	(-0.10,0.52)	0.16	0.16	
Relationship with father	(Ref: Very bad/somewhat bad)					0.050					0.038
	Very good/somewhat good	0.27	(-0.01,0.55)	0.14	0.21		0.30	(0.01,0.59)	0.15	0.23	
Parent marital status	(Ref: Parents married)					4.67e-08					1.00e-07
	Divorced	-0.40	(-0.58,-0.23)	0.09	-0.31		-0.39	(-0.56,-0.21)	0.09	-0.30	
	Parents were never married	-0.60	(-0.93,-0.27)	0.17	-0.46		-0.58	(-0.89,-0.27)	0.16	-0.44	
	One or both parents had died	-0.20	(-0.51,0.12)	0.16	-0.15		-0.18	(-0.47,0.12)	0.15	-0.13	
Subjective financial status of family growing up	(Ref: Got by)					0.105					0.398
	Lived comfortably	0.02	(-0.09,0.14)	0.06	0.02		-0.02	(-0.13,0.09)	0.06	-0.01	
	Found it difficult	-0.07	(-0.17,0.04)	0.05	-0.05		-0.06	(-0.17,0.04)	0.05	-0.05	
	Found it very difficult	-0.33	(-0.63,-0.02)	0.16	-0.25		-0.22	(-0.54,0.10)	0.16	-0.17	
Abuse	(Ref: No)					3.68e-05					6.47e-05
	Yes	-0.57	(-0.84,-0.30)	0.14	-0.44		-0.52	(-0.77,-0.26)	0.13	-0.40	

Variable	Category	Secure Flourishing Index Est	95% CI	SE	Est/SD	Global p-value	Flourishing Index Est	95% CI	SE	Est/SD	Global p-value
Outsider growing up	(Ref: No)					0.063					0.080
	Yes	-0.21	(-0.47,0.05)	0.13	-0.16		-0.19	(-0.45,0.06)	0.13	-0.15	
Self-rated health growing up	(Ref: Good)					7.30e-11					4.07e-11
	Excellent	0.48	(0.32,0.64)	0.08	0.37		0.47	(0.31,0.63)	0.08	0.36	
	Very good	0.21	(0.08,0.34)	0.07	0.16		0.20	(0.06,0.33)	0.07	0.15	
	Fair	-0.31	(-0.60,-0.03)	0.15	-0.24		-0.34	(-0.63,-0.05)	0.15	-0.26	
	Poor	0.15	(-0.44,0.75)	0.30	0.12		0.14	(-0.42,0.71)	0.29	0.11	
Immigration status	(Ref: Born in this country)					0.180					0.234
	Born in another country	-0.33	(-0.81,0.15)	0.25	-0.25		-0.34	(-0.89,0.22)	0.28	-0.26	
Age 12 religious service attendance	(Ref: Never)					< 2e-16					4.44e-16
	At least 1/week	0.98	(0.73,1.22)	0.13	0.75		0.94	(0.71,1.18)	0.12	0.72	
	1-3/month	0.70	(0.45,0.95)	0.13	0.54		0.70	(0.46,0.94)	0.12	0.53	
	< 1/month	0.48	(0.22,0.75)	0.14	0.37		0.48	(0.23,0.73)	0.13	0.37	
Year of birth	(Ref: 1998-2005; current age: 18-24)					4.99e-06					5.14e-05
	1993-1998; age 25-29	-0.22	(-0.37,-0.07)	0.08	-0.17		-0.14	(-0.29,0.01)	0.08	-0.10	
	1983-1993; age 30-39	-0.27	(-0.40,-0.13)	0.07	-0.21		-0.17	(-0.31,-0.04)	0.07	-0.13	
	1973-1983; age 40-49	-0.36	(-0.53,-0.20)	0.08	-0.28		-0.30	(-0.47,-0.14)	0.08	-0.23	
	1963-1973; age 50-59	-0.44	(-0.59,-0.28)	0.08	-0.34		-0.37	(-0.53,-0.21)	0.08	-0.28	

Variable	Category	Secure Flourishing Index Est	95% CI	SE	Est/SD	Global p-value	Flourishing Index Est	95% CI	SE	Est/SD	Global p-value
	1953-1963; age 60-69	-0.42	(-0.60,-0.25)	0.09	-0.32		-0.35	(-0.53,-0.18)	0.09	-0.27	
	1943-1953; age 70-79	-0.42	(-0.67,-0.18)	0.13	-0.33		-0.40	(-0.65,-0.16)	0.12	-0.31	
	1943 or earlier; age 80+	-0.76	(-1.24,-0.29)	0.24	-0.59		-0.65	(-1.08,-0.22)	0.22	-0.50	
Gender	(Ref: Male)					0.037					0.096
	Female	-0.05	(-0.13,0.03)	0.04	-0.04		0.01	(-0.07,0.09)	0.04	0.01	
	Other	-1.78	(-3.25,-0.31)	0.75	-1.37		-1.71	(-3.29,-0.12)	0.81	-1.31	
Religious affiliation	(Ref: No religion/Atheist/Agnostic)					0.003					0.004
	Christianity	-0.27	(-0.46,-0.08)	0.10	-0.21		-0.25	(-0.43,-0.06)	0.09	-0.19	
	Collapsed affiliations with prevalence<3%	-1.06	(-1.90,-0.22)	0.43	-0.82		-1.08	(-1.91,-0.26)	0.42	-0.83	
Race/ethnicity	(Ref: Plurality group)					0.964					0.506
	Non-plurality groups	0.01	(-0.43,0.45)	0.23	0.01		0.16	(-0.31,0.62)	0.24	0.12	

Note. N=10389; p < .004 (Bonferroni corrected threshold); Est., estimated effect of childhood predictor on flourishing score; CI, confidence interval; SE, standard error of the estimated effect; Est/SD, a more standardized measure of effect size--estimated effect of flourishing divided by standard deviation of flourishing--leads to the interpretation, for those with the given status (e.g., those with a good/very good relationship with mother compared to those with bad/very bad) are 0.XX standard deviations higher/lower on flourishing; the Global p-value corresponds to the two-sided joint parameter Wald-type test of whether any of the levels' parameters are non-zero, for history of abuse, outsider, relationship wtih mother/father, this is test of whether the estimated effect is non-zero, for multiple-category predictors (age, health, financial status), this is a joint test of whether any of these effects are non-zero. Note the confidence interval of the effect estimate can contradict the reported global p-value (e.g., for the single-category effects of relationship with mother). In such cases, the reported confidence interval is more robust with corrected degrees of freedom from the pooling across multiple imputations, whereas the global p-value is based on a Wald-type test and is less robust to uncertainty attributable to multiple imputation.

Table S15d. Sensitivity to unmeasured confounding of childhood predictors in Poland (N=10389)

		Secure Flourishing Index		Flourishing Index	
Variable	Category	E-value for Estimate	E-value for 95% CI	E-value for Estimate	E-value for 95% CI
Relationship with mother	(Ref: Very bad/somewhat bad)				
	Very good/somewhat good	1.57	1.00	1.59	1.00
Relationship with father	(Ref: Very bad/somewhat bad)				
	Very good/somewhat good	1.71	1.00	1.77	1.08
Parent marital status	(Ref: Parents married)				
	Divorced	1.98	1.63	1.95	1.60
	Parents were never married	2.41	1.72	2.37	1.70
	One or both parents had died	1.56	1.00	1.52	1.00
Subjective financial status of family growing up	(Ref: Got by)				
	Lived comfortably	1.14	1.00	1.13	1.00
	Found it difficult	1.27	1.00	1.27	1.00
	Found it very difficult	1.82	1.14	1.60	1.00
Abuse	(Ref: No)				
	Yes	2.33	1.76	2.23	1.70
Outsider growing up	(Ref: No)				
	Yes	1.58	1.00	1.56	1.00
Self-rated health growing up	(Ref: Good)				
	Excellent	2.14	1.81	2.13	1.80
	Very good	1.59	1.30	1.56	1.27
	Fair	1.79	1.16	1.85	1.23
	Poor	1.47	1.00	1.45	1.00
Immigration status	(Ref: Born in this country)				
	Born in another country	1.83	1.00	1.85	1.00
Age 12 religious service attendance	(Ref: Never)				
	At least 1/week	3.36	2.71	3.29	2.67
	1-3/month	2.64	2.08	2.64	2.10
	< 1/month	2.15	1.60	2.15	1.63
Year of birth	(Ref: 1998-2005; current age: 18-24)				
	1993-1998; age 25-29	1.60	1.27	1.43	1.00
	1983-1993; age 30-39	1.70	1.42	1.51	1.20

Variable	Category	Secure Flourishing Index		Flourishing Index	
		E-value for Estimate	E-value for 95% CI	E-value for Estimate	E-value for 95% CI
	1973-1983; age 40-49	1.90	1.57	1.78	1.43
	1963-1973; age 50-59	2.05	1.72	1.91	1.59
	1953-1963; age 60-69	2.02	1.66	1.87	1.52
	1943-1953; age 70-79	2.02	1.52	1.99	1.48
	1943 or earlier; age 80+	2.80	1.74	2.53	1.60
Gender	(Ref: Male)				
	Female	1.22	1.00	1.09	1.00
	Other	6.38	1.79	6.07	1.41
Religious affiliation	(Ref: No religion/Atheist/Agnostic)				
	Christianity	1.70	1.30	1.66	1.26
	Collapsed affiliations with prevalence<3%	3.61	1.62	3.70	1.69
Race/ethnicity	(Ref: Plurality group)				
	Non-plurality groups	1.08	1.00	1.48	1.00

Table S16a. Nationally representative descriptive statistics for South Africa

Characteristic	N = 2,651[1]
**Age group**	
1998-2005; age 18-24	461 (17%)
1993-1998; age 25-29	364 (14%)
1983-1993; age 30-39	655 (25%)
1973-1983; age 40-49	522 (20%)
1963-1973; age 50-59	309 (12%)
1953-1963; age 60-69	195 (7.4%)
1943-1953; age 70-79	120 (4.5%)
1943 or earlier; age 80+	17 (0.6%)
(Missing)	9 (0.3%)
**Gender**	
Male	1,288 (49%)
Female	1,356 (51%)
Other	2 (<0.1%)
(Missing)	4 (0.2%)
**Race/Ethnicity**	
Asian/Indian	6 (0.2%)
Black	2,381 (90%)
Colored	252 (9.5%)
Other	1 (<0.1%)
White	8 (0.3%)
(Missing)	3 (0.1%)
**Marital status**	
Married	539 (20%)
Separated	76 (2.9%)
Divorced	51 (1.9%)
Widowed	133 (5.0%)
Single, never married	1,561 (59%)
Domestic Partner	264 (10.0%)
(Missing)	28 (1.0%)
**Employment**	
Employed for an employer	569 (21%)
Self-employed	412 (16%)
Retired	243 (9.2%)
Student	204 (7.7%)
Homemaker	137 (5.2%)
Unemployed and looking for a job	1,008 (38%)
None of these/Other	74 (2.8%)
(Missing)	3 (0.1%)
**Religious service attendance**	
More than 1/week	414 (16%)
1/week	891 (34%)
1-3/month	574 (22%)
A few times a year	431 (16%)
Never	334 (13%)
(Missing)	7 (0.3%)
**Education**	
Up to 8 years	668 (25%)
9-15 years	1,796 (68%)
16+ years	183 (6.9%)
(Missing)	4 (0.2%)
**Immigration status**	
Born in this country	2,511 (95%)
Born in another country	139 (5.2%)
(Missing)	1 (<0.1%)
**Religious affiliation as an adult (now)**	
Christianity	2,163 (82%)
Islam	62 (2.3%)

**Characteristic**	**N = 2,651**[1]
Hinduism	1 (<0.1%)
Buddhism	12 (0.5%)
Judaism	0 (0%)
Sikhism	0 (0%)
Baha'i	0 (0%)
Jainism	2 (<0.1%)
Shinto	2 (<0.1%)
Taoism	1 (<0.1%)
Confucianism	0 (0%)
Primal, Animist, or Folk religion	127 (4.8%)
Spiritism	0 (0%)
Umbanda, Candomble, and other African-derived religions	0 (0%)
Chinese folk/traditional religion	0 (0%)
Some other religion	5 (0.2%)
No religion/Atheist/Agnostic	253 (9.6%)
(Missing)	23 (0.9%)
**Parent marital status**	
Parents married	1,321 (50%)
Divorced	131 (5.0%)
Parents were never married	904 (34%)
One or both parents had died	140 (5.3%)
(Missing)	155 (5.8%)
**Age 12 religious service attendance**	
At least 1/week	1,681 (63%)
1-3/month	552 (21%)
<1/month	175 (6.6%)
Never	217 (8.2%)
(Missing)	26 (1.0%)
**Relationship with mother**	
Very good	2,186 (82%)
Somewhat good	263 (9.9%)
Somewhat bad	51 (1.9%)
Very bad	39 (1.5%)
Does not apply	90 (3.4%)
(Missing)	21 (0.8%)
**Relationship with father**	
Very good	1,656 (62%)
Somewhat good	333 (13%)
Somewhat bad	86 (3.3%)
Very bad	159 (6.0%)
Does not apply	331 (12%)
(Missing)	85 (3.2%)
**Outsider growing up**	
Yes	434 (16%)
No	2,211 (83%)
(Missing)	6 (0.2%)
**Self-reported history of abuse**	
Yes	450 (17%)
No	2,149 (81%)
(Missing)	52 (2.0%)
**Self-rated health growing up**	
Excellent	1,225 (46%)
Very good	590 (22%)
Good	370 (14%)
Fair	266 (10%)
Poor	183 (6.9%)
(Missing)	17 (0.6%)
**Subjective financial status of family growing up**	
Lived comfortably	1,050 (40%)

**Characteristic**	**N = 2,651**[1]
Got by	875 (33%)
Found it difficult	432 (16%)
Found it very difficult	289 (11%)
(Missing)	5 (0.2%)
**Religious affiliation at age 12**	
Christianity	2,323 (88%)
Islam	52 (2.0%)
Hinduism	2 (<0.1%)
Buddhism	11 (0.4%)
Judaism	0 (0%)
Sikhism	0 (0%)
Baha'i	0 (0%)
Jainism	0 (0%)
Shinto	2 (<0.1%)
Taoism	1 (<0.1%)
Confucianism	0 (0%)
Primal, Animist, or Folk religion	117 (4.4%)
Spiritism	0 (0%)
Umbanda, Candomble, and other African-derived religions	0 (0%)
Chinese folk/traditional religion	0 (0%)
Some other religion	7 (0.3%)
No religion/Atheist/Agnostic	107 (4.1%)
(Missing)	27 (1.0%)

[1]n (%)

Table S16b. Means by demographic category for South Africa (N=2651)

Variable	Category	Secure Flourishing Index				Flourishing Index			
		Mean	95% CI	SE	Global p-value	Mean	95% CI	SE	Global p-value
Age group	18-24	7.53	(7.35,7.71)	0.09	0.028	7.23	(7.07,7.40)	0.08	0.010
	25-29	7.34	(7.17,7.50)	0.08		6.99	(6.82,7.15)	0.08	
	30-39	7.58	(7.43,7.73)	0.08		7.22	(7.07,7.37)	0.08	
	40-49	7.21	(7.04,7.39)	0.09		6.88	(6.71,7.06)	0.09	
	50-59	7.42	(7.16,7.68)	0.13		7.02	(6.76,7.29)	0.13	
	60-69	7.15	(6.65,7.65)	0.25		6.76	(6.27,7.26)	0.25	
	70-79	7.42	(6.91,7.92)	0.26		7.21	(6.68,7.73)	0.27	
	80 or older	7.96	(6.52,9.40)	0.60		7.98	(6.72,9.24)	0.52	
Gender	Female	7.37	(7.24,7.49)	0.06	0.440	7.02	(6.90,7.13)	0.06	0.303
	Male	7.46	(7.34,7.57)	0.06		7.13	(7.02,7.24)	0.06	
	Other	7.15	*	*		7.07	*	*	
Marital status	Divorced	6.82	(6.36,7.28)	0.23	0.016	6.52	(6.02,7.02)	0.25	0.065
	Domestic partner	7.46	(7.19,7.72)	0.13		7.09	(6.83,7.34)	0.13	
	Married	7.50	(7.29,7.72)	0.11		7.14	(6.93,7.35)	0.11	
	Separated	7.05	(6.65,7.44)	0.20		6.79	(6.42,7.16)	0.18	
	Single/Never been married	7.40	(7.30,7.50)	0.05		7.07	(6.97,7.16)	0.05	
	Widowed	7.50	(6.90,8.11)	0.31		7.18	(6.54,7.82)	0.32	
Employment	Employed for an employer	7.54	(7.38,7.70)	0.08	0.255	7.24	(7.08,7.39)	0.08	0.016
	Homemaker	7.22	(6.86,7.58)	0.18		6.92	(6.54,7.30)	0.19	
	None of these/Other	7.70	(7.11,8.28)	0.29		7.59	(7.00,8.17)	0.29	
	Retired	7.34	(6.90,7.78)	0.22		6.95	(6.50,7.40)	0.23	
	Self-employed	7.46	(7.24,7.68)	0.11		7.10	(6.90,7.31)	0.10	
	Student	7.51	(7.24,7.78)	0.14		7.22	(6.99,7.45)	0.12	
	Unemployed and looking for a job	7.32	(7.20,7.44)	0.06		6.95	(6.83,7.06)	0.06	
Religious service attendance	A few times a year	7.19	(6.99,7.39)	0.10	0.005	6.86	(6.66,7.06)	0.10	0.009
	More than once a week	7.67	(7.43,7.91)	0.12		7.30	(7.06,7.53)	0.12	

Variable	Category	Secure Flourishing Index				Flourishing Index			
		Mean	95% CI	SE	Global p-value	Mean	95% CI	SE	Global p-value
	Never	7.15	(6.91,7.40)	0.12		6.81	(6.56,7.06)	0.13	
	Once a week	7.50	(7.36,7.64)	0.07		7.15	(7.02,7.28)	0.07	
	One to three times a month	7.39	(7.23,7.54)	0.08		7.10	(6.95,7.26)	0.08	
Education	Up to 8	7.38	(7.15,7.60)	0.11	0.344	7.05	(6.83,7.27)	0.11	0.369
	9 to 15	7.60	(7.35,7.85)	0.13		7.24	(7.01,7.48)	0.12	
	16+	7.40	(7.31,7.49)	0.05		7.06	(6.97,7.15)	0.04	
Immigration status	Born in another country	7.32	(6.93,7.71)	0.20	0.635	6.98	(6.57,7.39)	0.21	0.641
	Born in this country	7.41	(7.32,7.51)	0.05		7.08	(6.99,7.17)	0.05	
Religious affiliation	Buddhism	6.91	*	*	< 2e-16	6.39	*	*	< 2e-16
	Christianity	7.42	(7.32,7.52)	0.05		7.08	(6.98,7.17)	0.05	
	Hinduism	8.50	*	*		8.00	*	*	
	Islam	7.72	(7.18,8.26)	0.27		7.29	(6.76,7.83)	0.27	
	No religion/Atheist/ Agnostic	7.36	(7.12,7.59)	0.12		7.09	(6.86,7.32)	0.12	
	Primal, Animist, or Folk religion	7.16	(6.74,7.59)	0.22		6.83	(6.37,7.29)	0.23	
	Shinto	9.47	*	*		8.14	*	*	
	Some other religion	7.25	*	*		6.50	*	*	
	Taoism	9.20	*	*		8.67	*	*	
	Jainism	8.50	*	*		8.58	*	*	

Note. N=2651; $p < .007 = 0.05/7$ (Bonferroni corrected p-value significance threshold); Mean, estimated group mean; CI, confidence interval for the mean within group; SE, complex survey adjusted standard error or the mean; Global p-value, two-tailed Wald-type test of whether there is evidence of any differences in mean scores among groups of a demographic characteristic. *Estimate is not reported due to multiple-imputation and complex survey adjusted degrees of freedom was less than 1.00 leading to insufficient information to provide an estimate of the uncertainty in the estimate. These groups are removed when estimating the global test of mean differences.

Table S16c. Childhood predictors regression analysis results for South Africa (N=2651)

		Secure Flourishing Index					Flourishing Index				
Variable	Category	Est	95% CI	SE	Est/SD	Global p-value	Est	95% CI	SE	Est/SD	Global p-value
Relationship with mother	(Ref: Very bad/somewhat bad)					0.454					0.414
	Very good/somewhat good	0.16	(-0.26,0.57)	0.21	0.10		0.17	(-0.24,0.57)	0.21	0.11	
Relationship with father	(Ref: Very bad/somewhat bad)					0.440					0.765
	Very good/somewhat good	0.08	(-0.15,0.32)	0.12	0.05		-0.01	(-0.24,0.23)	0.12	-0.01	
Parent marital status	(Ref: Parents married)					0.803					0.687
	Divorced	0.14	(-0.21,0.49)	0.18	0.09		0.14	(-0.18,0.47)	0.17	0.09	
	Parents were never married	-0.03	(-0.20,0.14)	0.09	-0.02		-0.05	(-0.22,0.12)	0.09	-0.03	
	One or both parents had died	-0.06	(-0.54,0.42)	0.24	-0.04		0.01	(-0.48,0.49)	0.25	0.00	
Subjective financial status of family growing up	(Ref: Got by)					0.092					0.257
	Lived comfortably	0.01	(-0.17,0.20)	0.09	0.01		0.01	(-0.18,0.20)	0.10	0.00	
	Found it difficult	-0.21	(-0.43,-0.00)	0.11	-0.14		-0.18	(-0.41,0.04)	0.12	-0.12	
	Found it very difficult	-0.32	(-0.67,0.03)	0.18	-0.20		-0.26	(-0.63,0.10)	0.18	-0.17	
Abuse	(Ref: No)					2.09e-06					1.2e-07
	Yes	-0.50	(-0.71,-0.29)	0.11	-0.31		-0.57	(-0.78,-0.35)	0.11	-0.37	

Variable	Category	Secure Flourishing Index					Flourishing Index				
		Est	95% CI	SE	Est/SD	Global p-value	Est	95% CI	SE	Est/SD	Global p-value
Outsider growing up	(Ref: No)					0.479					0.937
	Yes	-0.08	(-0.29,0.14)	0.11	-0.05		-0.01	(-0.21,0.20)	0.11	-0.00	
Self-rated health growing up	(Ref: Good)					0.087					0.044
	Excellent	0.27	(0.05,0.49)	0.11	0.17		0.30	(0.08,0.51)	0.11	0.19	
	Very good	0.18	(-0.08,0.43)	0.13	0.11		0.18	(-0.07,0.43)	0.13	0.12	
	Fair	0.02	(-0.32,0.36)	0.17	0.01		0.03	(-0.31,0.37)	0.17	0.02	
	Poor	0.05	(-0.26,0.36)	0.16	0.03		0.02	(-0.33,0.37)	0.18	0.01	
Immigration status	(Ref: Born in this country)					0.609					0.563
	Born in another country	-0.10	(-0.50,0.29)	0.20	-0.07		-0.11	(-0.48,0.26)	0.19	-0.07	
Age 12 religious service attendance	(Ref: Never)					0.360					0.575
	At least 1/week	-0.12	(-0.52,0.27)	0.20	-0.08		-0.11	(-0.55,0.33)	0.22	-0.07	
	1-3/month	0.03	(-0.37,0.44)	0.21	0.02		0.01	(-0.43,0.46)	0.23	0.01	
	< 1/month	-0.15	(-0.60,0.30)	0.23	-0.10		-0.15	(-0.64,0.35)	0.25	-0.09	
Year of birth	(Ref: 1998-2005; current age: 18-24)					0.031					0.048
	1993-1998; age 25-29	-0.25	(-0.48,-0.01)	0.12	-0.16		-0.19	(-0.43,0.05)	0.12	-0.12	
	1983-1993; age 30-39	0.02	(-0.20,0.23)	0.11	0.01		0.07	(-0.15,0.30)	0.11	0.05	
	1973-1983; age 40-49	-0.28	(-0.52,-0.04)	0.12	-0.18		-0.25	(-0.50,-0.00)	0.13	-0.16	
	1963-1973; age 50-59	-0.11	(-0.42,0.19)	0.15	-0.07		-0.03	(-0.33,0.27)	0.15	-0.02	

Variable	Category	Secure Flourishing Index					Flourishing Index				
		Est	95% CI	SE	Est/SD	Global p-value	Est	95% CI	SE	Est/SD	Global p-value
	1953-1963; age 60-69	-0.41	(-0.91,0.10)	0.26	-0.26		-0.33	(-0.84,0.17)	0.26	-0.21	
	1943-1953; age 70-79	0.04	(-0.47,0.54)	0.26	0.02		-0.08	(-0.57,0.42)	0.25	-0.05	
	1943 or earlier; age 80+	0.66	(-0.40,1.72)	0.54	0.41		0.33	(-0.88,1.54)	0.62	0.21	
Gender	(Ref: Male)					0.154					0.285
	Female	-0.14	(-0.27,0.00)	0.07	-0.09		-0.10	(-0.24,0.04)	0.07	-0.07	
	Other	-0.07	(-0.70,0.56)	0.32	-0.04		-0.31	(-1.01,0.40)	0.36	-0.20	
Religious affiliation	(Ref: No religion/Atheist/Agnostic)					0.299					0.724
	Primal, Animist, or Folk religion	-0.41	(-0.96,0.14)	0.28	-0.26		-0.19	(-0.78,0.39)	0.30	-0.13	
	Christianity	-0.10	(-0.58,0.37)	0.24	-0.06		0.02	(-0.48,0.52)	0.25	0.01	
	Collapsed affiliations with prevalence<3%	-0.19	(-0.89,0.51)	0.36	-0.12		0.07	(-0.65,0.78)	0.37	0.04	
Race/ethnicity	(Ref: Plurality group)					0.643					0.884
	Non-plurality groups	-0.08	(-0.41,0.25)	0.17	-0.05		-0.02	(-0.36,0.31)	0.17	-0.02	

Note. N=2651; p < .004 (Bonferroni corrected threshold); Est., estimated effect of childhood predictor on flourishing score; CI, confidence interval; SE, standard error of the estimated effect; Est/SD, a more standardized measure of effect size--estimated effect of flourishing divided by standard deviation of flourishing--leads to the interpretation, for those with the given status (e.g., those with a good/very good relationship with mother compared to those with bad/very bad) are 0.XX standard deviations higher/lower on flourishing; the Global p-value corresponds to the two-sided joint parameter Wald-type test of whether any of the levels' parameters are non-zero, for history of abuse, outsider, relationship wtih mother/father, this is test of whether the estimated effect is non-zero, for multiple-category predictors (age, health, financial status), this is a joint test of whether any of these effects are non-zero. Note the confidence interval of the effect estimate can contradict the reported global p-value (e.g., for the single-category effects of relationship with mother). In such cases, the reported confidence

Variable	Category	Secure Flourishing Index					Flourishing Index				
		Est	95% CI	SE	Est/SD	Global p-value	Est	95% CI	SE	Est/SD	Global p-value

interval is more robust with corrected degrees of freedom from the pooling across multiple imputations, whereas the global p-value is based on a Wald-type test and is less robust to uncertainty attributable to multiple imputation.

Table S16d. Sensitivity to unmeasured confounding of childhood predictors in South Africa (N=2651)

		Secure Flourishing Index		Flourishing Index	
Variable	Category	E-value for Estimate	E-value for 95% CI	E-value for Estimate	E-value for 95% CI
Relationship with mother	(Ref: Very bad/somewhat bad)				
	Very good/somewhat good	1.42	1.00	1.44	1.00
Relationship with father	(Ref: Very bad/somewhat bad)				
	Very good/somewhat good	1.28	1.00	1.07	1.00
Parent marital status	(Ref: Parents married)				
	Divorced	1.38	1.00	1.39	1.00
	Parents were never married	1.15	1.00	1.21	1.00
	One or both parents had died	1.23	1.00	1.06	1.00
Subjective financial status of family growing up	(Ref: Got by)				
	Lived comfortably	1.10	1.00	1.06	1.00
	Found it difficult	1.52	1.05	1.46	1.00
	Found it very difficult	1.70	1.00	1.60	1.00
Abuse	(Ref: No)				
	Yes	2.01	1.65	2.11	1.75
Outsider growing up	(Ref: No)				
	Yes	1.27	1.00	1.07	1.00
Self-rated health growing up	(Ref: Good)				
	Excellent	1.62	1.21	1.66	1.28
	Very good	1.46	1.00	1.45	1.00
	Fair	1.12	1.00	1.16	1.00
	Poor	1.20	1.00	1.11	1.00
Immigration status	(Ref: Born in this country)				
	Born in another country	1.32	1.00	1.33	1.00
Age 12 religious service attendance	(Ref: Never)				
	At least 1/week	1.36	1.00	1.33	1.00
	1-3/month	1.16	1.00	1.09	1.00
	< 1/month	1.41	1.00	1.40	1.00
Year of birth	(Ref: 1998-2005; current age: 18-24)				
	1993-1998; age 25-29	1.58	1.10	1.47	1.00
	1983-1993; age 30-39	1.11	1.00	1.26	1.00

Variable	Category	Secure Flourishing Index		Flourishing Index	
		E-value for Estimate	E-value for 95% CI	E-value for Estimate	E-value for 95% CI
	1973-1983; age 40-49	1.64	1.19	1.58	1.03
	1963-1973; age 50-59	1.34	1.00	1.15	1.00
	1953-1963; age 60-69	1.85	1.00	1.72	1.00
	1943-1953; age 70-79	1.17	1.00	1.26	1.00
	1943 or earlier; age 80+	2.30	1.00	1.71	1.00
Gender	(Ref: Male)				
	Female	1.38	1.00	1.32	1.00
	Other	1.25	1.00	1.67	1.00
Religious affiliation	(Ref: No religion/Atheist/Agnostic)				
	Primal, Animist, or Folk religion	1.86	1.00	1.48	1.00
	Christianity	1.32	1.00	1.11	1.00
	Collapsed affiliations with prevalence<3%	1.48	1.00	1.24	1.00
Race/ethnicity	(Ref: Plurality group)				
	Non-plurality groups	1.27	1.00	1.13	1.00

Table S17a. Nationally representative descriptive statistics for Spain

Characteristic	N = 6,290[1]
**Age group**	
1998-2005; age 18-24	594 (9.4%)
1993-1998; age 25-29	450 (7.2%)
1983-1993; age 30-39	1,111 (18%)
1973-1983; age 40-49	1,396 (22%)
1963-1973; age 50-59	1,252 (20%)
1953-1963; age 60-69	977 (16%)
1943-1953; age 70-79	467 (7.4%)
1943 or earlier; age 80+	43 (0.7%)
(Missing)	0 (0%)
**Gender**	
Male	3,142 (50%)
Female	3,119 (50%)
Other	6 (0.1%)
(Missing)	22 (0.4%)
**Marital status**	
Married	2,947 (47%)
Separated	237 (3.8%)
Divorced	518 (8.2%)
Widowed	189 (3.0%)
Single, never married	1,742 (28%)
Domestic Partner	589 (9.4%)
(Missing)	67 (1.1%)
**Employment**	
Employed for an employer	2,862 (45%)
Self-employed	576 (9.2%)
Retired	1,278 (20%)
Student	448 (7.1%)
Homemaker	345 (5.5%)
Unemployed and looking for a job	646 (10%)
None of these/Other	123 (2.0%)
(Missing)	11 (0.2%)
**Religious service attendance**	
More than 1/week	317 (5.0%)
1/week	662 (11%)
1-3/month	437 (6.9%)
A few times a year	1,972 (31%)
Never	2,875 (46%)
(Missing)	27 (0.4%)
**Education**	
Up to 8 years	802 (13%)
9-15 years	4,145 (66%)
16+ years	1,341 (21%)
(Missing)	2 (<0.1%)
**Immigration status**	
Born in this country	5,479 (87%)
Born in another country	788 (13%)
(Missing)	23 (0.4%)
**Religious affiliation as an adult (now)**	
Christianity	4,074 (65%)
Islam	135 (2.1%)
Hinduism	7 (0.1%)
Buddhism	36 (0.6%)
Judaism	4 (<0.1%)
Sikhism	3 (<0.1%)
Baha'i	2 (<0.1%)
Jainism	1 (<0.1%)
Shinto	0 (0%)

Characteristic	N = 6,290[1]
Taoism	5 (<0.1%)
Confucianism	3 (<0.1%)
Primal, Animist, or Folk religion	7 (0.1%)
Spiritism	0 (0%)
Umbanda, Candomble, and other African-derived religions	0 (0%)
Chinese folk/traditional religion	0 (0%)
Some other religion	27 (0.4%)
No religion/Atheist/Agnostic	1,932 (31%)
(Missing)	55 (0.9%)
**Parent marital status**	
Parents married	5,285 (84%)
Divorced	378 (6.0%)
Parents were never married	312 (5.0%)
One or both parents had died	126 (2.0%)
(Missing)	188 (3.0%)
**Age 12 religious service attendance**	
At least 1/week	2,391 (38%)
1-3/month	1,132 (18%)
<1/month	1,287 (20%)
Never	1,445 (23%)
(Missing)	36 (0.6%)
**Relationship with mother**	
Very good	4,557 (72%)
Somewhat good	1,258 (20%)
Somewhat bad	248 (3.9%)
Very bad	92 (1.5%)
Does not apply	107 (1.7%)
(Missing)	28 (0.4%)
**Relationship with father**	
Very good	4,131 (66%)
Somewhat good	1,397 (22%)
Somewhat bad	309 (4.9%)
Very bad	178 (2.8%)
Does not apply	243 (3.9%)
(Missing)	33 (0.5%)
**Outsider growing up**	
Yes	579 (9.2%)
No	5,637 (90%)
(Missing)	75 (1.2%)
**Self-reported history of abuse**	
Yes	659 (10%)
No	5,510 (88%)
(Missing)	122 (1.9%)
**Self-rated health growing up**	
Excellent	2,450 (39%)
Very good	2,286 (36%)
Good	1,235 (20%)
Fair	164 (2.6%)
Poor	135 (2.1%)
(Missing)	20 (0.3%)
**Subjective financial status of family growing up**	
Lived comfortably	2,041 (32%)
Got by	2,956 (47%)
Found it difficult	1,154 (18%)
Found it very difficult	110 (1.7%)
(Missing)	29 (0.5%)
**Religious affiliation at age 12**	
Christianity	5,119 (81%)
Islam	132 (2.1%)

Characteristic	N = 6,290[1]
Hinduism	5 (<0.1%)
Buddhism	8 (0.1%)
Judaism	5 (<0.1%)
Sikhism	2 (<0.1%)
Baha'i	0 (0%)
Jainism	0 (0%)
Shinto	0 (0%)
Taoism	0 (0%)
Confucianism	1 (<0.1%)
Primal, Animist, or Folk religion	4 (<0.1%)
Spiritism	0 (0%)
Umbanda, Candomble, and other African-derived religions	0 (0%)
Chinese folk/traditional religion	0 (0%)
Some other religion	13 (0.2%)
No religion/Atheist/Agnostic	972 (15%)
(Missing)	29 (0.5%)

[1] n (%)

Table S17b. Means by demographic category for Spain (N=6290)

Variable	Category	Secure Flourishing Index				Flourishing Index			
		Mean	95% CI	SE	Global p-value	Mean	95% CI	SE	Global p-value
Age group	18-24	7.13	(6.98,7.28)	0.07	0.068	6.75	(6.61,6.88)	0.07	0.002
	25-29	7.33	(7.19,7.47)	0.07		6.90	(6.76,7.04)	0.07	
	30-39	7.26	(7.17,7.35)	0.05		6.83	(6.75,6.92)	0.04	
	40-49	7.32	(7.24,7.40)	0.04		6.87	(6.79,6.95)	0.04	
	50-59	7.30	(7.20,7.41)	0.05		6.87	(6.77,6.97)	0.05	
	60-69	7.40	(7.27,7.53)	0.07		7.04	(6.91,7.16)	0.06	
	70-79	7.34	(7.06,7.61)	0.14		7.00	(6.71,7.29)	0.15	
	80 or older	7.81	(7.30,8.33)	0.25		7.69	(7.18,8.19)	0.25	
Gender	Female	7.23	(7.17,7.30)	0.03	0.003	6.77	(6.70,6.83)	0.03	4.48e-08
	Male	7.38	(7.31,7.45)	0.03		7.02	(6.96,7.09)	0.03	
	Other	7.70	(6.25,9.15)	0.29		7.47	(6.02,8.93)	0.29	
Marital status	Divorced	7.25	(7.08,7.41)	0.09	2.56e-06	6.84	(6.67,7.01)	0.09	2.63e-06
	Domestic partner	7.36	(7.22,7.49)	0.07		6.90	(6.77,7.04)	0.07	
	Married	7.43	(7.36,7.50)	0.04		7.02	(6.95,7.09)	0.04	
	Separated	7.20	(6.96,7.44)	0.12		6.82	(6.58,7.05)	0.12	
	Single/Never been married	7.14	(7.06,7.21)	0.04		6.72	(6.65,6.80)	0.04	
	Widowed	7.09	(6.75,7.42)	0.17		6.74	(6.39,7.08)	0.17	
Employment	Employed for an employer	7.39	(7.33,7.45)	0.03	8.79e-06	6.98	(6.92,7.03)	0.03	1.22e-12
	Homemaker	7.32	(7.08,7.57)	0.12		6.80	(6.59,7.01)	0.11	
	None of these/Other	7.07	(6.78,7.36)	0.15		6.62	(6.34,6.89)	0.14	
	Retired	7.33	(7.20,7.47)	0.07		7.01	(6.87,7.15)	0.07	
	Self-employed	7.35	(7.23,7.47)	0.06		6.96	(6.84,7.08)	0.06	
	Student	7.10	(6.94,7.27)	0.08		6.75	(6.59,6.90)	0.08	
	Unemployed and looking for a job	7.02	(6.89,7.16)	0.07		6.44	(6.32,6.57)	0.07	
Religious service attendance	A few times a year	7.31	(7.23,7.39)	0.04	< 2e-16	6.87	(6.79,6.95)	0.04	< 2e-16
	More than once a week	7.86	(7.64,8.07)	0.11		7.49	(7.28,7.70)	0.11	

Variable	Category	Secure Flourishing Index				Flourishing Index			
		Mean	95% CI	SE	Global p-value	Mean	95% CI	SE	Global p-value
	Never	7.12	(7.06,7.19)	0.03		6.73	(6.66,6.80)	0.03	
	Once a week	7.69	(7.55,7.84)	0.07		7.30	(7.16,7.44)	0.07	
	One to three times a month	7.49	(7.32,7.66)	0.09		7.05	(6.89,7.21)	0.08	
Education	Up to 8	7.29	(7.10,7.47)	0.09	8.06e-06	6.81	(6.63,6.99)	0.09	1.04e-08
	9 to 15	7.50	(7.41,7.58)	0.04		7.14	(7.05,7.22)	0.04	
	16+	7.25	(7.19,7.30)	0.03		6.83	(6.78,6.89)	0.03	
Immigration status	Born in another country	7.76	(7.65,7.88)	0.06	1.55e-15	7.23	(7.12,7.35)	0.06	9.52e-10
	Born in this country	7.24	(7.19,7.29)	0.03		6.85	(6.80,6.90)	0.03	
Religious affiliation	Baha'i	7.66	*	*	< 2e-16	7.58	*	*	< 2e-16
	Buddhism	6.71	(6.01,7.41)	0.33		6.39	(5.79,7.00)	0.29	
	Christianity	7.42	(7.36,7.48)	0.03		6.98	(6.93,7.04)	0.03	
	Hinduism	6.40	*	*		6.22	*	*	
	Islam	7.21	(6.88,7.54)	0.17		6.80	(6.51,7.09)	0.15	
	Judaism	7.75	*	*		7.44	*	*	
	No religion/Atheist/ Agnostic	7.10	(7.02,7.18)	0.04		6.73	(6.65,6.81)	0.04	
	Primal, Animist, or Folk religion	6.94	*	*		6.69	*	*	
	Sikhism	7.64	*	*		7.82	*	*	
	Some other religion	7.64	(7.03,8.25)	0.28		7.18	(6.43,7.93)	0.34	
	Taoism	5.92	*	*		5.39	*	*	
	Confucianism	3.00	*	*		2.93	*	*	
	Jainism	5.00	*	*		5.42	*	*	

Note. N=6290; $p < .007 = 0.05/7$ (Bonferroni corrected p-value significance threshold); Mean, estimated group mean; CI, confidence interval for the mean within group; SE, complex survey adjusted standard error or the mean; Global p-value, two-tailed Wald-type test of whether there is evidence of any differences in mean scores among groups of a demographic characteristic. *Estimate is not reported due to multiple-imputation and complex survey adjusted degrees of freedom was less than 1.00 leading to insufficient information to provide an estimate of the uncertainty in the estimate. These groups are removed when estimating the global test of mean differences.

Table S17c. Childhood predictors regression analysis results for Spain (N=6290)

Variable	Category	Secure Flourishing Index					Flourishing Index				
		Est	95% CI	SE	Est/SD	Global p-value	Est	95% CI	SE	Est/SD	Global p-value
Relationship with mother	(Ref: Very bad/somewhat bad)					9.19e-07					4.13e-07
	Very good/somewhat good	0.51	(0.31,0.72)	0.10	0.36		0.55	(0.34,0.76)	0.11	0.39	
Relationship with father	(Ref: Very bad/somewhat bad)					0.145					0.145
	Very good/somewhat good	0.12	(-0.04,0.28)	0.08	0.08		0.12	(-0.04,0.28)	0.08	0.09	
Parent marital status	(Ref: Parents married)					0.516					0.443
	Divorced	0.03	(-0.17,0.22)	0.10	0.02		0.01	(-0.19,0.20)	0.10	0.00	
	Parents were never married	0.03	(-0.14,0.21)	0.09	0.02		0.02	(-0.16,0.21)	0.10	0.02	
	One or both parents had died	-0.17	(-0.43,0.09)	0.13	-0.12		-0.22	(-0.50,0.06)	0.14	-0.16	
Subjective financial status of family growing up	(Ref: Got by)					3.81e-04					0.044
	Lived comfortably	0.13	(0.03,0.23)	0.05	0.09		0.09	(-0.01,0.20)	0.05	0.07	
	Found it difficult	-0.16	(-0.30,-0.03)	0.07	-0.11		-0.08	(-0.22,0.05)	0.07	-0.06	
	Found it very difficult	-0.34	(-0.73,0.05)	0.20	-0.24		-0.30	(-0.73,0.14)	0.22	-0.21	
Abuse	(Ref: No)					2.46e-06					1.11e-06
	Yes	-0.33	(-0.47,-0.19)	0.07	-0.23		-0.36	(-0.51,-0.22)	0.08	-0.26	

Variable	Category	Secure Flourishing Index Est	95% CI	SE	Est/SD	Global p-value	Flourishing Index Est	95% CI	SE	Est/SD	Global p-value
Outsider growing up	(Ref: No)					3.98e-05					8.10e-06
	Yes	-0.32	(-0.48,-0.17)	0.08	-0.23		-0.35	(-0.50,-0.20)	0.08	-0.25	
Self-rated health growing up	(Ref: Good)					1.13e-13					< 2e-16
	Excellent	0.54	(0.41,0.68)	0.07	0.38		0.62	(0.49,0.76)	0.07	0.45	
	Very good	0.35	(0.22,0.47)	0.06	0.24		0.36	(0.23,0.48)	0.06	0.26	
	Fair	0.04	(-0.27,0.35)	0.16	0.03		0.01	(-0.30,0.32)	0.16	0.01	
	Poor	0.20	(-0.22,0.62)	0.21	0.14		0.22	(-0.22,0.67)	0.23	0.16	
Immigration status	(Ref: Born in this country)					< 2e-16					< 2e-16
	Born in another country	0.54	(0.42,0.67)	0.06	0.38		0.66	(0.54,0.79)	0.06	0.48	
Age 12 religious service attendance	(Ref: Never)					2.08e-07					8.18e-08
	At least 1/week	0.22	(0.10,0.34)	0.06	0.15		0.24	(0.12,0.36)	0.06	0.17	
	1-3/month	0.25	(0.13,0.37)	0.06	0.17		0.22	(0.09,0.35)	0.06	0.16	
	< 1/month	-0.04	(-0.17,0.09)	0.07	-0.03		-0.07	(-0.21,0.06)	0.07	-0.05	
Year of birth	(Ref: 1998-2005; current age: 18-24)					0.005					0.150
	1993-1998; age 25-29	0.09	(-0.09,0.28)	0.09	0.07		0.13	(-0.06,0.32)	0.10	0.09	
	1983-1993; age 30-39	0.04	(-0.11,0.19)	0.08	0.03		0.07	(-0.09,0.23)	0.08	0.05	
	1973-1983; age 40-49	0.08	(-0.07,0.22)	0.07	0.05		0.13	(-0.03,0.28)	0.08	0.09	
	1963-1973; age 50-59	0.09	(-0.07,0.25)	0.08	0.06		0.12	(-0.04,0.29)	0.08	0.09	

Variable	Category	Secure Flourishing Index Est	95% CI	SE	Est/SD	Global p-value	Flourishing Index Est	95% CI	SE	Est/SD	Global p-value
	1953-1963; age 60-69	0.26	(0.09,0.44)	0.09	0.18		0.22	(0.04,0.41)	0.09	0.16	
	1943-1953; age 70-79	0.25	(-0.04,0.53)	0.15	0.17		0.18	(-0.11,0.46)	0.15	0.13	
	1943 or earlier; age 80+	0.83	(0.32,1.34)	0.26	0.58		0.58	(0.09,1.06)	0.25	0.41	
Gender	(Ref: Male)					2.04e-07					0.042
	Female	-0.22	(-0.31,-0.13)	0.05	-0.15		-0.10	(-0.19,-0.01)	0.05	-0.07	
	Other	0.38	(0.02,0.74)	0.18	0.27		0.18	(-0.26,0.62)	0.22	0.13	
Religious affiliation	(Ref: No religion/Atheist/Agnostic)					0.921					0.151
	Christianity	0.02	(-0.09,0.14)	0.06	0.02		0.12	(-0.00,0.24)	0.06	0.08	
	Collapsed affiliations with prevalence<3%	0.02	(-0.24,0.29)	0.13	0.02		0.11	(-0.20,0.41)	0.15	0.08	
Race/ethnicity	(Ref: Plurality group)										

Note. N=6290; p < .004 (Bonferroni corrected threshold); Est., estimated effect of childhood predictor on flourishing score; CI, confidence interval; SE, standard error of the estimated effect; Est/SD, a more standardized measure of effect size--estimated effect of flourishing divided by standard deviation of flourishing--leads to the interpretation, for those with the given status (e.g., those with a good/very good relationship with mother compared to those with bad/very bad) are 0.XX standard deviations higher/lower on flourishing; the Global p-value corresponds to the two-sided joint parameter Wald-type test of whether any of the levels' parameters are non-zero, for history of abuse, outsider, relationship wtih mother/father, this is test of whether the estimated effect is non-zero, for multiple-category predictors (age, health, financial status), this is a joint test of whether any of these effects are non-zero. Note the confidence interval of the effect estimate can contradict the reported global p-value (e.g., for the single-category effects of relationship with mother). In such cases, the reported confidence interval is more robust with corrected degrees of freedom from the pooling across multiple imputations, whereas the global p-value is based on a Wald-type test and is less robust to uncertainty attributable to multiple imputation.

Table S17d. Sensitivity to unmeasured confounding of childhood predictors in Spain (N=6290)

Variable	Category	Secure Flourishing Index		Flourishing Index	
		E-value for Estimate	E-value for 95% CI	E-value for Estimate	E-value for 95% CI
Relationship with mother	(Ref: Very bad/somewhat bad)				
	Very good/somewhat good	2.14	1.75	2.19	1.79
Relationship with father	(Ref: Very bad/somewhat bad)				
	Very good/somewhat good	1.38	1.00	1.37	1.00
Parent marital status	(Ref: Parents married)				
	Divorced	1.16	1.00	1.07	1.00
	Parents were never married	1.18	1.00	1.14	1.00
	One or both parents had died	1.48	1.00	1.57	1.00
Subjective financial status of family growing up	(Ref: Got by)				
	Lived comfortably	1.39	1.15	1.32	1.00
	Found it difficult	1.46	1.16	1.30	1.00
	Found it very difficult	1.81	1.00	1.71	1.00
Abuse	(Ref: No)				
	Yes	1.79	1.52	1.84	1.56
Outsider growing up	(Ref: No)				
	Yes	1.77	1.48	1.81	1.52
Self-rated health growing up	(Ref: Good)				
	Excellent	2.20	1.94	2.34	2.07
	Very good	1.82	1.58	1.82	1.59
	Fair	1.19	1.00	1.09	1.00
	Poor	1.54	1.00	1.57	1.00
Immigration status	(Ref: Born in this country)				
	Born in another country	2.21	1.96	2.43	2.17
Age 12 religious service attendance	(Ref: Never)				
	At least 1/week	1.57	1.33	1.60	1.37
	1-3/month	1.63	1.39	1.57	1.32
	< 1/month	1.19	1.00	1.27	1.00
Year of birth	(Ref: 1998-2005; current age: 18-24)				
	1993-1998; age 25-29	1.32	1.00	1.39	1.00
	1983-1993; age 30-39	1.18	1.00	1.26	1.00

Variable	Category	Secure Flourishing Index		Flourishing Index	
		E-value for Estimate	E-value for 95% CI	E-value for Estimate	E-value for 95% CI
	1973-1983; age 40-49	1.29	1.00	1.39	1.00
	1963-1973; age 50-59	1.31	1.00	1.38	1.00
	1953-1963; age 60-69	1.65	1.30	1.57	1.19
	1943-1953; age 70-79	1.63	1.00	1.49	1.00
	1943 or earlier; age 80+	2.82	1.77	2.25	1.32
Gender	(Ref: Male)				
	Female	1.57	1.40	1.34	1.11
	Other	1.89	1.14	1.50	1.00
Religious affiliation	(Ref: No religion/Atheist/Agnostic)				
	Christianity	1.14	1.00	1.37	1.00
	Collapsed affiliations with prevalence<3%	1.14	1.00	1.34	1.00
Race/ethnicity	(Ref: Plurality group)				

Table S18a. Nationally representative descriptive statistics for Sweden

Characteristic	N = 15,068[1]
**Age group**	
1998-2005; age 18-24	1,515 (10%)
1993-1998; age 25-29	1,399 (9.3%)
1983-1993; age 30-39	2,398 (16%)
1973-1983; age 40-49	2,221 (15%)
1963-1973; age 50-59	2,493 (17%)
1953-1963; age 60-69	2,168 (14%)
1943-1953; age 70-79	2,253 (15%)
1943 or earlier; age 80+	621 (4.1%)
(Missing)	0 (0%)
**Gender**	
Male	7,536 (50%)
Female	7,493 (50%)
Other	27 (0.2%)
(Missing)	12 (<0.1%)
**Marital status**	
Married	6,408 (43%)
Separated	426 (2.8%)
Divorced	801 (5.3%)
Widowed	433 (2.9%)
Single, never married	3,854 (26%)
Domestic Partner	3,073 (20%)
(Missing)	72 (0.5%)
**Employment**	
Employed for an employer	7,907 (52%)
Self-employed	1,243 (8.3%)
Retired	3,832 (25%)
Student	1,332 (8.8%)
Homemaker	75 (0.5%)
Unemployed and looking for a job	324 (2.2%)
None of these/Other	337 (2.2%)
(Missing)	18 (0.1%)
**Religious service attendance**	
More than 1/week	236 (1.6%)
1/week	434 (2.9%)
1-3/month	486 (3.2%)
A few times a year	3,950 (26%)
Never	9,918 (66%)
(Missing)	45 (0.3%)
**Education**	
Up to 8 years	252 (1.7%)
9-15 years	10,790 (72%)
16+ years	4,026 (27%)
(Missing)	0 (0%)
**Immigration status**	
Born in this country	13,922 (92%)
Born in another country	1,052 (7.0%)
(Missing)	94 (0.6%)
**Religious affiliation as an adult (now)**	
Christianity	8,346 (55%)
Islam	470 (3.1%)
Hinduism	22 (0.1%)
Buddhism	110 (0.7%)
Judaism	54 (0.4%)
Sikhism	4 (<0.1%)
Baha'i	6 (<0.1%)
Jainism	0 (0%)
Shinto	0 (<0.1%)

Characteristic	N = 15,068[1]
Taoism	4 (<0.1%)
Confucianism	0 (0%)
Primal, Animist, or Folk religion	83 (0.5%)
Spiritism	0 (0%)
Umbanda, Candomble, and other African-derived religions	0 (0%)
Chinese folk/traditional religion	0 (0%)
Some other religion	198 (1.3%)
No religion/Atheist/Agnostic	5,697 (38%)
(Missing)	74 (0.5%)
**Parent marital status**	
Parents married	10,887 (72%)
Divorced	1,927 (13%)
Parents were never married	1,747 (12%)
One or both parents had died	362 (2.4%)
(Missing)	145 (1.0%)
**Age 12 religious service attendance**	
At least 1/week	955 (6.3%)
1-3/month	1,362 (9.0%)
<1/month	6,224 (41%)
Never	6,472 (43%)
(Missing)	54 (0.4%)
**Relationship with mother**	
Very good	8,743 (58%)
Somewhat good	4,513 (30%)
Somewhat bad	1,194 (7.9%)
Very bad	371 (2.5%)
Does not apply	216 (1.4%)
(Missing)	30 (0.2%)
**Relationship with father**	
Very good	7,134 (47%)
Somewhat good	4,885 (32%)
Somewhat bad	1,588 (11%)
Very bad	725 (4.8%)
Does not apply	720 (4.8%)
(Missing)	16 (0.1%)
**Outsider growing up**	
Yes	1,867 (12%)
No	13,034 (86%)
(Missing)	168 (1.1%)
**Self-reported history of abuse**	
Yes	2,288 (15%)
No	12,735 (85%)
(Missing)	45 (0.3%)
**Self-rated health growing up**	
Excellent	5,733 (38%)
Very good	5,124 (34%)
Good	2,669 (18%)
Fair	1,108 (7.4%)
Poor	397 (2.6%)
(Missing)	38 (0.2%)
**Subjective financial status of family growing up**	
Lived comfortably	5,951 (39%)
Got by	7,717 (51%)
Found it difficult	1,238 (8.2%)
Found it very difficult	140 (0.9%)
(Missing)	22 (0.1%)
**Religious affiliation at age 12**	
Christianity	10,617 (70%)
Islam	462 (3.1%)

**Characteristic**	**N = 15,068**[1]
Hinduism	16 (0.1%)
Buddhism	41 (0.3%)
Judaism	51 (0.3%)
Sikhism	9 (<0.1%)
Baha'i	3 (<0.1%)
Jainism	0 (0%)
Shinto	1 (<0.1%)
Taoism	0 (0%)
Confucianism	4 (<0.1%)
Primal, Animist, or Folk religion	31 (0.2%)
Spiritism	0 (0%)
Umbanda, Candomble, and other African-derived religions	0 (0%)
Chinese folk/traditional religion	0 (0%)
Some other religion	69 (0.5%)
No religion/Atheist/Agnostic	3,738 (25%)
(Missing)	26 (0.2%)

[1]n (%)

Table S18b. Means by demographic category for Sweden (N=15068)

Variable	Category	Secure Flourishing Index				Flourishing Index			
		Mean	95% CI	SE	Global p-value	Mean	95% CI	SE	Global p-value
Age group	18-24	6.52	(6.42,6.61)	0.05	< 2e-16	6.59	(6.50,6.68)	0.05	< 2e-16
	25-29	6.66	(6.56,6.76)	0.05		6.64	(6.54,6.73)	0.05	
	30-39	6.72	(6.65,6.80)	0.04		6.72	(6.65,6.79)	0.04	
	40-49	6.82	(6.73,6.90)	0.04		6.82	(6.74,6.90)	0.04	
	50-59	7.10	(7.02,7.17)	0.04		7.16	(7.09,7.23)	0.04	
	60-69	7.38	(7.31,7.45)	0.04		7.46	(7.40,7.53)	0.03	
	70-79	7.65	(7.58,7.71)	0.03		7.78	(7.72,7.84)	0.03	
	80 or older	7.64	(7.51,7.77)	0.07		7.81	(7.69,7.93)	0.06	
Gender	Female	7.01	(6.97,7.05)	0.02	2.09e-05	7.05	(7.01,7.09)	0.02	6.06e-07
	Male	7.08	(7.04,7.12)	0.02		7.15	(7.11,7.19)	0.02	
	Other	5.57	(4.80,6.33)	0.37		5.74	(5.06,6.43)	0.33	
Marital status	Divorced	6.95	(6.83,7.07)	0.06	< 2e-16	6.94	(6.83,7.06)	0.06	< 2e-16
	Domestic partner	7.00	(6.94,7.06)	0.03		7.01	(6.96,7.07)	0.03	
	Married	7.50	(7.46,7.54)	0.02		7.56	(7.52,7.60)	0.02	
	Separated	6.66	(6.48,6.84)	0.09		6.66	(6.48,6.84)	0.09	
	Single/Never been married	6.34	(6.28,6.41)	0.03		6.42	(6.36,6.49)	0.03	
	Widowed	7.32	(7.16,7.49)	0.08		7.47	(7.32,7.62)	0.08	
Employment	Employed for an employer	6.96	(6.92,7.00)	0.02	< 2e-16	7.00	(6.96,7.03)	0.02	< 2e-16
	Homemaker	7.05	(6.66,7.44)	0.20		6.98	(6.60,7.37)	0.19	
	None of these/Other	5.84	(5.60,6.08)	0.12		5.79	(5.56,6.03)	0.12	
	Retired	7.48	(7.42,7.53)	0.03		7.60	(7.55,7.65)	0.03	
	Self-employed	7.46	(7.35,7.57)	0.06		7.50	(7.39,7.61)	0.06	
	Student	6.55	(6.45,6.64)	0.05		6.59	(6.50,6.69)	0.05	
	Unemployed and looking for a job	5.56	(5.33,5.79)	0.12		5.51	(5.28,5.74)	0.12	
Religious service attendance	A few times a year	7.29	(7.24,7.35)	0.03	< 2e-16	7.34	(7.28,7.39)	0.03	< 2e-16
	More than once a week	7.90	(7.64,8.16)	0.13		7.79	(7.54,8.05)	0.13	

Variable	Category	Secure Flourishing Index				Flourishing Index			
		Mean	95% CI	SE	Global p-value	Mean	95% CI	SE	Global p-value
	Never	6.90	(6.87,6.94)	0.02		6.97	(6.93,7.00)	0.02	
	Once a week	7.31	(7.12,7.50)	0.10		7.28	(7.10,7.47)	0.10	
	One to three times a month	7.18	(7.03,7.34)	0.08		7.21	(7.06,7.37)	0.08	
Education	Up to 8	7.19	(6.94,7.44)	0.13	2.56e-10	7.26	(7.02,7.50)	0.12	2.38e-14
	9 to 15	7.20	(7.15,7.26)	0.03		7.28	(7.22,7.33)	0.03	
	16+	6.98	(6.94,7.02)	0.02		7.02	(6.99,7.06)	0.02	
Immigration status	Born in another country	7.13	(7.01,7.25)	0.06	0.140	7.09	(6.98,7.21)	0.06	0.927
	Born in this country	7.04	(7.01,7.07)	0.02		7.10	(7.07,7.13)	0.02	
Religious affiliation	Baha'i	7.23	*	*	< 2e-16	6.84	*	*	< 2e-16
	Buddhism	6.71	(6.34,7.09)	0.19		6.73	(6.35,7.12)	0.19	
	Christianity	7.28	(7.24,7.32)	0.02		7.33	(7.30,7.37)	0.02	
	Hinduism	6.27	(4.55,7.99)	0.74		6.25	(4.63,7.86)	0.69	
	Islam	7.09	(6.89,7.30)	0.10		6.98	(6.78,7.18)	0.10	
	Judaism	6.98	(6.28,7.68)	0.35		7.00	(6.35,7.66)	0.32	
	No religion/Atheist/ Agnostic	6.75	(6.70,6.79)	0.02		6.82	(6.77,6.86)	0.02	
	Primal, Animist, or Folk religion	6.16	(5.69,6.64)	0.24		6.26	(5.81,6.70)	0.22	
	Shinto	5.80	*	*		6.00	*	*	
	Sikhism	7.76	*	*		7.82	*	*	
	Some other religion	6.16	(5.75,6.56)	0.21		6.10	(5.71,6.49)	0.20	
	Taoism	6.11	*	*		6.49	*	*	

Note. N=15068; p < .007 = 0.05/7 (Bonferroni corrected p-value significance threshold); Mean, estimated group mean; CI, confidence interval for the mean within group; SE, complex survey adjusted standard error or the mean; Global p-value, two-tailed Wald-type test of whether there is evidence of any differences in mean scores among groups of a demographic characteristic. *Estimate is not reported due to multiple-imputation and complex survey adjusted degrees of freedom was less than 1.00 leading to insufficient information to provide an estimate of the uncertainty in the estimate. These groups are removed when estimating the global test of mean differences.

Table S18c. Childhood predictors regression analysis results for Sweden (N=15068)

Variable	Category	Secure Flourishing Index					Flourishing Index				
		Est	95% CI	SE	Est/SD	Global p-value	Est	95% CI	SE	Est/SD	Global p-value
Relationship with mother	(Ref: Very bad/somewhat bad)					0.418					0.474
	Very good/somewhat good	0.04	(-0.06,0.14)	0.05	0.03		0.04	(-0.07,0.14)	0.05	0.03	
Relationship with father	(Ref: Very bad/somewhat bad)					0.005					0.001
	Very good/somewhat good	0.12	(0.04,0.21)	0.04	0.08		0.15	(0.06,0.24)	0.05	0.10	
Parent marital status	(Ref: Parents married)					0.027					0.008
	Divorced	0.10	(0.01,0.18)	0.04	0.06		0.13	(0.04,0.22)	0.05	0.08	
	Parents were never married	-0.06	(-0.16,0.03)	0.05	-0.04		-0.06	(-0.15,0.04)	0.05	-0.04	
	One or both parents had died	0.10	(-0.09,0.28)	0.10	0.06		0.09	(-0.11,0.29)	0.10	0.06	
Subjective financial status of family growing up	(Ref: Got by)					< 2e-16					4.77e-13
	Lived comfortably	0.27	(0.22,0.33)	0.03	0.17		0.22	(0.17,0.28)	0.03	0.15	
	Found it difficult	-0.09	(-0.19,0.02)	0.06	-0.05		-0.03	(-0.15,0.08)	0.06	-0.02	
	Found it very difficult	-0.02	(-0.37,0.34)	0.18	-0.01		0.03	(-0.34,0.39)	0.19	0.02	
Abuse	(Ref: No)					1.48e-05					0.002
	Yes	-0.19	(-0.27,-0.10)	0.04	-0.12		-0.14	(-0.23,-0.05)	0.05	-0.09	

Variable	Category	Secure Flourishing Index					Flourishing Index				
		Est	95% CI	SE	Est/SD	Global p-value	Est	95% CI	SE	Est/SD	Global p-value
Outsider growing up	(Ref: No)					1.06e-13					1.32e-13
	Yes	-0.39	(-0.49,-0.28)	0.05	-0.25		-0.40	(-0.51,-0.29)	0.05	-0.26	
Self-rated health growing up	(Ref: Good)					< 2e-16					< 2e-16
	Excellent	0.98	(0.90,1.06)	0.04	0.62		1.01	(0.93,1.10)	0.04	0.66	
	Very good	0.48	(0.40,0.56)	0.04	0.30		0.50	(0.41,0.58)	0.04	0.32	
	Fair	-0.18	(-0.31,-0.05)	0.07	-0.12		-0.16	(-0.29,-0.03)	0.07	-0.10	
	Poor	-0.52	(-0.75,-0.29)	0.12	-0.33		-0.54	(-0.78,-0.30)	0.12	-0.35	
Immigration status	(Ref: Born in this country)					0.029					0.003
	Born in another country	0.12	(0.01,0.23)	0.06	0.08		0.17	(0.06,0.29)	0.06	0.11	
Age 12 religious service attendance	(Ref: Never)					9.05e-05					2.84e-06
	At least 1/week	0.28	(0.16,0.41)	0.06	0.18		0.34	(0.21,0.47)	0.07	0.22	
	1-3/month	0.08	(-0.01,0.18)	0.05	0.05		0.10	(0.01,0.20)	0.05	0.07	
	< 1/month	0.06	(0.00,0.12)	0.03	0.04		0.07	(0.01,0.13)	0.03	0.04	
Year of birth	(Ref: 1998-2005; current age: 18-24)					< 2e-16					< 2e-16
	1993-1998; age 25-29	0.12	(0.01,0.24)	0.06	0.08		0.21	(0.09,0.34)	0.06	0.14	
	1983-1993; age 30-39	0.22	(0.12,0.33)	0.05	0.14		0.29	(0.18,0.40)	0.06	0.19	
	1973-1983; age 40-49	0.25	(0.14,0.37)	0.06	0.16		0.32	(0.20,0.43)	0.06	0.21	
	1963-1973; age 50-59	0.51	(0.40,0.62)	0.06	0.32		0.51	(0.40,0.63)	0.06	0.33	

Variable	Category	Secure Flourishing Index Est	95% CI	SE	Est/SD	Global p-value	Flourishing Index Est	95% CI	SE	Est/SD	Global p-value
	1953-1963; age 60-69	0.75	(0.64,0.86)	0.06	0.48		0.73	(0.62,0.84)	0.06	0.47	
	1943-1953; age 70-79	1.01	(0.90,1.12)	0.06	0.64		0.94	(0.83,1.06)	0.06	0.61	
	1943 or earlier; age 80+	1.06	(0.91,1.21)	0.07	0.67		0.95	(0.79,1.11)	0.08	0.62	
Gender	(Ref: Male)					0.072					0.004
	Female	0.05	(-0.01,0.10)	0.03	0.03		0.08	(0.02,0.13)	0.03	0.05	
	Other	-0.42	(-1.00,0.16)	0.30	-0.27		-0.54	(-1.15,0.06)	0.31	-0.35	
Religious affiliation	(Ref: No religion/Atheist/Agnostic)					5.08e-06					8.03e-07
	Islam	0.32	(0.13,0.52)	0.10	0.20		0.41	(0.20,0.61)	0.10	0.26	
	Christianity	0.16	(0.09,0.23)	0.03	0.10		0.17	(0.10,0.24)	0.04	0.11	
	Collapsed affiliations with prevalence<3%	0.16	(-0.11,0.42)	0.13	0.10		0.25	(-0.03,0.53)	0.14	0.16	
Race/ethnicity	(Ref: Plurality group)										

Note. N=15068; p < .004 (Bonferroni corrected threshold); Est., estimated effect of childhood predictor on flourishing score; CI, confidence interval; SE, standard error of the estimated effect; Est/SD, a more standardized measure of effect size--estimated effect of flourishing divided by standard deviation of flourishing--leads to the interpretation, for those with the given status (e.g., those with a good/very good relationship with mother compared to those with bad/very bad) are 0.XX standard deviations higher/lower on flourishing; the Global p-value corresponds to the two-sided joint parameter Wald-type test of whether any of the levels' parameters are non-zero, for history of abuse, outsider, relationship wtih mother/father, this is test of whether the estimated effect is non-zero, for multiple-category predictors (age, health, financial status), this is a joint test of whether any of these effects are non-zero. Note the confidence interval of the effect estimate can contradict the reported global p-value (e.g., for the single-category effects of relationship with mother). In such cases, the reported confidence interval is more robust with corrected degrees of freedom from the pooling across multiple imputations, whereas the global p-value is based on a Wald-type test and is less robust to uncertainty attributable to multiple imputation.

Table S18d. Sensitivity to unmeasured confounding of childhood predictors in Sweden (N=15068)

		Secure Flourishing Index		Flourishing Index	
Variable	Category	E-value for Estimate	E-value for 95% CI	E-value for Estimate	E-value for 95% CI
Relationship with mother	(Ref: Very bad/somewhat bad)				
	Very good/somewhat good	1.19	1.00	1.17	1.00
Relationship with father	(Ref: Very bad/somewhat bad)				
	Very good/somewhat good	1.36	1.17	1.40	1.23
Parent marital status	(Ref: Parents married)				
	Divorced	1.31	1.08	1.36	1.17
	Parents were never married	1.24	1.00	1.22	1.00
	One or both parents had died	1.31	1.00	1.29	1.00
Subjective financial status of family growing up	(Ref: Got by)				
	Lived comfortably	1.63	1.53	1.54	1.43
	Found it difficult	1.29	1.00	1.16	1.00
	Found it very difficult	1.11	1.00	1.15	1.00
Abuse	(Ref: No)				
	Yes	1.48	1.32	1.38	1.20
Outsider growing up	(Ref: No)				
	Yes	1.82	1.65	1.83	1.65
Self-rated health growing up	(Ref: Good)				
	Excellent	2.96	2.79	2.99	2.81
	Very good	1.99	1.85	2.00	1.86
	Fair	1.47	1.21	1.42	1.14
	Poor	2.06	1.66	2.08	1.67
Immigration status	(Ref: Born in this country)				
	Born in another country	1.36	1.09	1.44	1.22
Age 12 religious service attendance	(Ref: Never)				
	At least 1/week	1.64	1.43	1.73	1.51
	1-3/month	1.28	1.00	1.32	1.07
	< 1/month	1.23	1.05	1.25	1.07
Year of birth	(Ref: 1998-2005; current age: 18-24)				
	1993-1998; age 25-29	1.36	1.08	1.52	1.30
	1983-1993; age 30-39	1.54	1.35	1.65	1.47

Variable	Category	Secure Flourishing Index		Flourishing Index	
		E-value for Estimate	E-value for 95% CI	E-value for Estimate	E-value for 95% CI
	1973-1983; age 40-49	1.60	1.40	1.69	1.50
	1963-1973; age 50-59	2.04	1.85	2.03	1.83
	1953-1963; age 60-69	2.49	2.28	2.42	2.21
	1943-1953; age 70-79	3.03	2.79	2.84	2.61
	1943 or earlier; age 80+	3.15	2.83	2.87	2.55
Gender	(Ref: Male)				
	Female	1.19	1.00	1.26	1.13
	Other	1.89	1.00	2.08	1.00
Religious affiliation	(Ref: No religion/Atheist/Agnostic)				
	Islam	1.71	1.37	1.85	1.50
	Christianity	1.43	1.30	1.44	1.31
	Collapsed affiliations with prevalence<3%	1.42	1.00	1.58	1.00
Race/ethnicity	(Ref: Plurality group)				

Table S19a. Nationally representative descriptive statistics for Tanzania

Characteristic	N = 9,075[1]
**Age group**	
1998-2005; age 18-24	2,284 (25%)
1993-1998; age 25-29	1,349 (15%)
1983-1993; age 30-39	2,060 (23%)
1973-1983; age 40-49	1,503 (17%)
1963-1973; age 50-59	912 (10%)
1953-1963; age 60-69	575 (6.3%)
1943-1953; age 70-79	297 (3.3%)
1943 or earlier; age 80+	93 (1.0%)
(Missing)	2 (<0.1%)
**Gender**	
Male	4,299 (47%)
Female	4,776 (53%)
Other	0 (0%)
(Missing)	0 (0%)
**Race/Ethnicity**	
African	9,060 (100%)
Arab	11 (0.1%)
Indian	3 (<0.1%)
(Missing)	2 (<0.1%)
**Marital status**	
Married	5,577 (61%)
Separated	404 (4.5%)
Divorced	103 (1.1%)
Widowed	450 (5.0%)
Single, never married	2,260 (25%)
Domestic Partner	275 (3.0%)
(Missing)	7 (<0.1%)
**Employment**	
Employed for an employer	513 (5.6%)
Self-employed	4,625 (51%)
Retired	139 (1.5%)
Student	319 (3.5%)
Homemaker	1,796 (20%)
Unemployed and looking for a job	1,491 (16%)
None of these/Other	186 (2.1%)
(Missing)	6 (<0.1%)
**Religious service attendance**	
More than 1/week	2,622 (29%)
1/week	4,268 (47%)
1-3/month	1,082 (12%)
A few times a year	814 (9.0%)
Never	288 (3.2%)
(Missing)	1 (<0.1%)
**Education**	
Up to 8 years	6,699 (74%)
9-15 years	2,252 (25%)
16+ years	122 (1.3%)
(Missing)	2 (<0.1%)
**Immigration status**	
Born in this country	9,048 (100%)
Born in another country	25 (0.3%)
(Missing)	1 (<0.1%)
**Religious affiliation as an adult (now)**	
Christianity	5,647 (62%)
Islam	3,189 (35%)
Hinduism	0 (0%)
Buddhism	0 (0%)

Characteristic	N = 9,075[1]
Judaism	0 (0%)
Sikhism	0 (0%)
Baha'i	0 (0%)
Jainism	0 (0%)
Shinto	0 (0%)
Taoism	1 (<0.1%)
Confucianism	0 (0%)
Primal, Animist, or Folk religion	12 (0.1%)
Spiritism	0 (0%)
Umbanda, Candomble, and other African-derived religions	0 (0%)
Chinese folk/traditional religion	0 (0%)
Some other religion	0 (0%)
No religion/Atheist/Agnostic	216 (2.4%)
(Missing)	10 (0.1%)
**Parent marital status**	
Parents married	6,929 (76%)
Divorced	678 (7.5%)
Parents were never married	751 (8.3%)
One or both parents had died	313 (3.4%)
(Missing)	404 (4.4%)
**Age 12 religious service attendance**	
At least 1/week	5,580 (61%)
1-3/month	2,383 (26%)
<1/month	333 (3.7%)
Never	595 (6.6%)
(Missing)	184 (2.0%)
**Relationship with mother**	
Very good	7,739 (85%)
Somewhat good	796 (8.8%)
Somewhat bad	84 (0.9%)
Very bad	84 (0.9%)
Does not apply	303 (3.3%)
(Missing)	70 (0.8%)
**Relationship with father**	
Very good	6,831 (75%)
Somewhat good	1,101 (12%)
Somewhat bad	203 (2.2%)
Very bad	247 (2.7%)
Does not apply	550 (6.1%)
(Missing)	142 (1.6%)
**Outsider growing up**	
Yes	734 (8.1%)
No	8,320 (92%)
(Missing)	22 (0.2%)
**Self-reported history of abuse**	
Yes	716 (7.9%)
No	8,328 (92%)
(Missing)	32 (0.3%)
**Self-rated health growing up**	
Excellent	2,406 (27%)
Very good	2,036 (22%)
Good	2,946 (32%)
Fair	1,177 (13%)
Poor	456 (5.0%)
(Missing)	54 (0.6%)
**Subjective financial status of family growing up**	
Lived comfortably	2,611 (29%)
Got by	2,909 (32%)
Found it difficult	2,679 (30%)

**Characteristic**	**N = 9,075**[1]
Found it very difficult	814 (9.0%)
(Missing)	61 (0.7%)
**Religious affiliation at age 12**	
Christianity	5,651 (62%)
Islam	3,060 (34%)
Hinduism	0 (0%)
Buddhism	0 (0%)
Judaism	0 (0%)
Sikhism	0 (0%)
Baha'i	1 (<0.1%)
Jainism	0 (0%)
Shinto	0 (0%)
Taoism	0 (0%)
Confucianism	0 (0%)
Primal, Animist, or Folk religion	11 (0.1%)
Spiritism	0 (0%)
Umbanda, Candomble, and other African-derived religions	0 (0%)
Chinese folk/traditional religion	0 (0%)
Some other religion	0 (0%)
No religion/Atheist/Agnostic	345 (3.8%)
(Missing)	7 (<0.1%)

[1]n (%)

Table S19b. Means by demographic category for Tanzania (N=9075)

Variable	Category	Secure Flourishing Index				Flourishing Index			
		Mean	95% CI	SE	Global p-value	Mean	95% CI	SE	Global p-value
Age group	18-24	7.91	(7.81,8.02)	0.05	< 2e-16	7.66	(7.56,7.77)	0.05	< 2e-16
	25-29	7.70	(7.57,7.83)	0.07		7.41	(7.27,7.55)	0.07	
	30-39	7.46	(7.34,7.58)	0.06		7.16	(7.04,7.28)	0.06	
	40-49	7.29	(7.15,7.43)	0.07		6.97	(6.83,7.11)	0.07	
	50-59	7.06	(6.90,7.23)	0.09		6.78	(6.61,6.94)	0.08	
	60-69	7.04	(6.78,7.29)	0.13		6.80	(6.54,7.06)	0.13	
	70-79	6.66	(6.23,7.10)	0.22		6.29	(5.86,6.73)	0.22	
	80 or older	6.32	(5.78,6.85)	0.27		6.00	(5.45,6.55)	0.28	
Gender	Female	7.49	(7.39,7.60)	0.05	0.538	7.20	(7.10,7.31)	0.05	0.628
	Male	7.46	(7.36,7.56)	0.05		7.18	(7.08,7.28)	0.05	
Marital status	Divorced	7.00	(6.64,7.36)	0.18	2.00e-15	6.60	(6.22,6.99)	0.19	< 2e-16
	Domestic partner	7.34	(7.05,7.64)	0.15		7.07	(6.77,7.37)	0.15	
	Married	7.47	(7.36,7.57)	0.05		7.18	(7.07,7.28)	0.05	
	Separated	6.91	(6.69,7.12)	0.11		6.57	(6.34,6.79)	0.11	
	Single/Never been married	7.79	(7.68,7.90)	0.06		7.53	(7.42,7.64)	0.06	
	Widowed	6.77	(6.46,7.09)	0.16		6.43	(6.12,6.75)	0.16	
Employment	Employed for an employer	7.92	(7.74,8.09)	0.09	6.66e-16	7.69	(7.52,7.86)	0.09	< 2e-16
	Homemaker	7.40	(7.25,7.55)	0.08		7.10	(6.95,7.25)	0.08	
	None of these/Other	6.79	(6.26,7.33)	0.27		6.37	(5.85,6.90)	0.27	
	Retired	6.97	(6.58,7.37)	0.20		6.77	(6.39,7.16)	0.19	
	Self-employed	7.44	(7.33,7.54)	0.06		7.15	(7.04,7.26)	0.06	
	Student	8.23	(8.06,8.41)	0.09		7.99	(7.81,8.16)	0.09	
	Unemployed and looking for a job	7.52	(7.38,7.66)	0.07		7.23	(7.10,7.36)	0.07	
Religious service attendance	A few times a year	7.17	(6.99,7.35)	0.09	7.77e-05	6.90	(6.72,7.08)	0.09	7.68e-04
	More than once a week	7.54	(7.43,7.66)	0.06		7.24	(7.13,7.36)	0.06	
	Never	7.16	(6.88,7.44)	0.14		7.02	(6.75,7.29)	0.14	

Variable	Category	Secure Flourishing Index Mean	95% CI	SE	Global p-value	Flourishing Index Mean	95% CI	SE	Global p-value
	Once a week	7.56	(7.44,7.67)	0.06		7.26	(7.15,7.38)	0.06	
	One to three times a month	7.32	(7.16,7.48)	0.08		7.04	(6.88,7.21)	0.08	
Education	Up to 8	7.32	(7.21,7.42)	0.05	< 2e-16	7.02	(6.92,7.13)	0.05	< 2e-16
	9 to 15	8.22	(8.00,8.44)	0.11		7.94	(7.69,8.18)	0.12	
	16+	7.92	(7.83,8.01)	0.05		7.65	(7.56,7.74)	0.05	
Immigration status	Born in another country	7.67	(6.65,8.68)	0.49	0.695	7.41	(6.61,8.21)	0.39	0.566
	Born in this country	7.48	(7.39,7.56)	0.04		7.19	(7.10,7.28)	0.04	
Religious affiliation	Christianity	7.56	(7.45,7.66)	0.05	< 2e-16	7.29	(7.18,7.39)	0.05	< 2e-16
	Islam	7.37	(7.24,7.50)	0.07		7.05	(6.92,7.18)	0.07	
	No religion/Atheist/ Agnostic	6.98	(6.63,7.33)	0.18		6.71	(6.39,7.04)	0.16	
	Primal, Animist, or Folk religion	8.08	(7.02,9.14)	0.41		7.56	(6.11,9.01)	0.57	
	Taoism	6.60	*	*		6.33	*	*	

Note. N=9075; $p < .007 = 0.05/7$ (Bonferroni corrected p-value significance threshold); Mean, estimated group mean; CI, confidence interval for the mean within group; SE, complex survey adjusted standard error or the mean; Global p-value, two-tailed Wald-type test of whether there is evidence of any differences in mean scores among groups of a demographic characteristic. *Estimate is not reported due to multiple-imputation and complex survey adjusted degrees of freedom was less than 1.00 leading to insufficient information to provide an estimate of the uncertainty in the estimate. These groups are removed when estimating the global test of mean differences.

Table S19c. Childhood predictors regression analysis results for Tanzania (N=9075)

Variable	Category	Secure Flourishing Index					Flourishing Index				
		Est	95% CI	SE	Est/SD	Global p-value	Est	95% CI	SE	Est/SD	Global p-value
Relationship with mother	(Ref: Very bad/somewhat bad)					0.207					0.319
	Very good/somewhat good	0.17	(-0.09,0.43)	0.13	0.09		0.14	(-0.13,0.41)	0.14	0.08	
Relationship with father	(Ref: Very bad/somewhat bad)					0.720					0.542
	Very good/somewhat good	0.03	(-0.16,0.22)	0.10	0.02		0.06	(-0.14,0.25)	0.10	0.03	
Parent marital status	(Ref: Parents married)					0.010					0.014
	Divorced	-0.24	(-0.42,-0.06)	0.09	-0.13		-0.24	(-0.43,-0.06)	0.09	-0.13	
	Parents were never married	0.01	(-0.16,0.19)	0.09	0.01		0.05	(-0.13,0.23)	0.09	0.03	
	One or both parents had died	-0.30	(-0.59,-0.01)	0.15	-0.17		-0.25	(-0.53,0.04)	0.15	-0.13	
Subjective financial status of family growing up	(Ref: Got by)					7.06e-07					8.64e-05
	Lived comfortably	0.08	(-0.03,0.19)	0.06	0.05		0.04	(-0.08,0.15)	0.06	0.02	
	Found it difficult	-0.19	(-0.30,-0.08)	0.06	-0.11		-0.16	(-0.28,-0.05)	0.06	-0.09	
	Found it very difficult	-0.47	(-0.68,-0.27)	0.10	-0.26		-0.40	(-0.60,-0.19)	0.10	-0.22	
Abuse	(Ref: No)					1.65e-08					2.19e-08
	Yes	-0.52	(-0.69,-0.35)	0.09	-0.29		-0.49	(-0.66,-0.33)	0.08	-0.27	

Variable	Category	Secure Flourishing Index Est	95% CI	SE	Est/SD	Global p-value	Flourishing Index Est	95% CI	SE	Est/SD	Global p-value
Outsider growing up	(Ref: No)					0.207					0.139
	Yes	-0.12	(-0.32,0.07)	0.10	-0.07		-0.14	(-0.33,0.05)	0.09	-0.08	
Self-rated health growing up	(Ref: Good)					1.20e-04					9.15e-05
	Excellent	0.20	(0.07,0.33)	0.07	0.11		0.20	(0.07,0.33)	0.07	0.11	
	Very good	0.25	(0.10,0.41)	0.08	0.14		0.26	(0.11,0.41)	0.08	0.14	
	Fair	0.11	(-0.05,0.27)	0.08	0.06		0.11	(-0.06,0.27)	0.08	0.06	
	Poor	-0.30	(-0.52,-0.08)	0.11	-0.16		-0.30	(-0.52,-0.09)	0.11	-0.17	
Immigration status	(Ref: Born in this country)					0.329					0.536
	Born in another country	0.25	(-0.25,0.75)	0.26	0.14		0.22	(-0.48,0.93)	0.36	0.12	
Age 12 religious service attendance	(Ref: Never)					0.139					0.134
	At least 1/week	-0.03	(-0.30,0.23)	0.14	-0.02		0.01	(-0.27,0.28)	0.14	0.00	
	1-3/month	-0.20	(-0.50,0.11)	0.15	-0.11		-0.15	(-0.47,0.16)	0.16	-0.08	
	< 1/month	-0.10	(-0.47,0.28)	0.19	-0.05		-0.10	(-0.47,0.28)	0.19	-0.05	
Year of birth	(Ref: 1998-2005; current age: 18-24)					< 2e-16					< 2e-16
	1993-1998; age 25-29	-0.23	(-0.37,-0.10)	0.07	-0.13		-0.19	(-0.32,-0.07)	0.07	-0.11	
	1983-1993; age 30-39	-0.45	(-0.58,-0.31)	0.07	-0.24		-0.40	(-0.53,-0.27)	0.07	-0.22	
	1973-1983; age 40-49	-0.61	(-0.76,-0.47)	0.07	-0.34		-0.56	(-0.70,-0.41)	0.08	-0.30	
	1963-1973; age 50-59	-0.83	(-1.01,-0.66)	0.09	-0.46		-0.81	(-0.98,-0.63)	0.09	-0.44	

Variable	Category	Secure Flourishing Index Est	95% CI	SE	Est/SD	Global p-value	Flourishing Index Est	95% CI	SE	Est/SD	Global p-value
	1953-1963; age 60-69	-0.88	(-1.14,-0.63)	0.13	-0.49		-0.89	(-1.14,-0.64)	0.13	-0.49	
	1943-1953; age 70-79	-1.38	(-1.81,-0.96)	0.22	-0.76		-1.27	(-1.70,-0.85)	0.22	-0.70	
	1943 or earlier; age 80+	-1.74	(-2.29,-1.19)	0.28	-0.96		-1.67	(-2.21,-1.13)	0.28	-0.91	
Gender	(Ref: Male)					0.934					0.895
	Female	-0.00	(-0.10,0.09)	0.05	-0.00		0.01	(-0.09,0.10)	0.05	0.00	
Religious affiliation	(Ref: No religion/Atheist/Agnostic)					0.025					0.013
	Islam	0.24	(-0.17,0.66)	0.21	0.13		0.26	(-0.19,0.70)	0.23	0.14	
	Christianity	0.42	(0.03,0.82)	0.20	0.23		0.39	(-0.04,0.82)	0.22	0.21	
	Collapsed affiliations with prevalence<3%	0.77	(-0.27,1.82)	0.53	0.42		1.04	(0.29,1.79)	0.38	0.57	
Race/ethnicity	(Ref: Plurality group)					0.087					0.013
	Non-plurality groups	-0.44	(-0.95,0.06)	0.26	-0.24		-0.51	(-0.91,-0.11)	0.20	-0.28	

Note. N=9075; p < .004 (Bonferroni corrected threshold); Est., estimated effect of childhood predictor on flourishing score; CI, confidence interval; SE, standard error of the estimated effect; Est/SD, a more standardized measure of effect size--estimated effect of flourishing divided by standard deviation of flourishing--leads to the interpretation, for those with the given status (e.g., those with a good/very good relationship with mother compared to those with bad/very bad) are 0.XX standard deviations higher/lower on flourishing; the Global p-value corresponds to the two-sided joint parameter Wald-type test of whether any of the levels' parameters are non-zero, for history of abuse, outsider, relationship wtih mother/father, this is test of whether the estimated effect is non-zero, for multiple-category predictors (age, health, financial status), this is a joint test of whether any of these effects are non-zero. Note the confidence interval of the effect estimate can contradict the reported global p-value (e.g., for the single-category effects of relationship with mother). In such cases, the reported confidence interval is more robust with corrected degrees of freedom from the pooling across multiple imputations, whereas the global p-value is based on a Wald-type test and is less robust to uncertainty attributable to multiple imputation.

Table S19d. Sensitivity to unmeasured confounding of childhood predictors in Tanzania (N=9075)

		Secure Flourishing Index		Flourishing Index	
Variable	Category	E-value for Estimate	E-value for 95% CI	E-value for Estimate	E-value for 95% CI
Relationship with mother	(Ref: Very bad/somewhat bad)				
	Very good/somewhat good	1.39	1.00	1.35	1.00
Relationship with father	(Ref: Very bad/somewhat bad)				
	Very good/somewhat good	1.14	1.00	1.20	1.00
Parent marital status	(Ref: Parents married)				
	Divorced	1.50	1.20	1.51	1.20
	Parents were never married	1.09	1.00	1.19	1.00
	One or both parents had died	1.60	1.09	1.52	1.00
Subjective financial status of family growing up	(Ref: Got by)				
	Lived comfortably	1.25	1.00	1.15	1.00
	Found it difficult	1.43	1.24	1.39	1.19
	Found it very difficult	1.85	1.55	1.74	1.44
Abuse	(Ref: No)				
	Yes	1.91	1.66	1.88	1.64
Outsider growing up	(Ref: No)				
	Yes	1.32	1.00	1.35	1.00
Self-rated health growing up	(Ref: Good)				
	Excellent	1.45	1.23	1.45	1.23
	Very good	1.53	1.29	1.54	1.29
	Fair	1.31	1.00	1.30	1.00
	Poor	1.59	1.25	1.60	1.26
Immigration status	(Ref: Born in this country)				
	Born in another country	1.52	1.00	1.48	1.00
Age 12 religious service attendance	(Ref: Never)				
	At least 1/week	1.15	1.00	1.07	1.00
	1-3/month	1.44	1.00	1.37	1.00
	< 1/month	1.27	1.00	1.28	1.00
Year of birth	(Ref: 1998-2005; current age: 18-24)				
	1993-1998; age 25-29	1.50	1.28	1.44	1.22
	1983-1993; age 30-39	1.81	1.61	1.75	1.56

Variable	Category	Secure Flourishing Index		Flourishing Index	
		E-value for Estimate	E-value for 95% CI	E-value for Estimate	E-value for 95% CI
	1973-1983; age 40-49	2.05	1.84	1.97	1.76
	1963-1973; age 50-59	2.40	2.13	2.36	2.09
	1953-1963; age 60-69	2.48	2.08	2.50	2.10
	1943-1953; age 70-79	3.40	2.61	3.19	2.43
	1943 or earlier; age 80+	4.19	3.01	4.04	2.91
Gender	(Ref: Male)				
	Female	1.05	1.00	1.06	1.00
Religious affiliation	(Ref: No religion/Atheist/Agnostic)				
	Islam	1.51	1.00	1.53	1.00
	Christianity	1.77	1.13	1.73	1.00
	Collapsed affiliations with prevalence<3%	2.30	1.00	2.75	1.58
Race/ethnicity	(Ref: Plurality group)				
	Non-plurality groups	1.80	1.00	1.90	1.30

Table S20a. Nationally representative descriptive statistics for Turkey

**Characteristic**	**N = 1,473**[1]
**Age group**	
1998-2005; age 18-24	222 (15%)
1993-1998; age 25-29	152 (10%)
1983-1993; age 30-39	315 (21%)
1973-1983; age 40-49	312 (21%)
1963-1973; age 50-59	225 (15%)
1953-1963; age 60-69	164 (11%)
1943-1953; age 70-79	65 (4.4%)
1943 or earlier; age 80+	18 (1.2%)
(Missing)	0 (0%)
**Gender**	
Male	754 (51%)
Female	719 (49%)
Other	0 (0%)
(Missing)	0 (0%)
**Race/Ethnicity**	
Albanian	8 (0.5%)
Arab	51 (3.5%)
Armenian	1 (<0.1%)
Azeri	9 (0.6%)
Bosnian	5 (0.3%)
Circassian	19 (1.3%)
Georgian	4 (0.3%)
Greek	1 (<0.1%)
Kurdish/Zaza	252 (17%)
Laz	25 (1.7%)
Other	58 (3.9%)
Turkish	1,030 (70%)
Uyghur	1 (<0.1%)
(Missing)	9 (0.6%)
**Marital status**	
Married	936 (64%)
Separated	13 (0.9%)
Divorced	64 (4.3%)
Widowed	64 (4.3%)
Single, never married	379 (26%)
Domestic Partner	0 (0%)
(Missing)	17 (1.1%)
**Employment**	
Employed for an employer	413 (28%)
Self-employed	255 (17%)
Retired	205 (14%)
Student	107 (7.3%)
Homemaker	347 (24%)
Unemployed and looking for a job	87 (5.9%)
None of these/Other	59 (4.0%)
(Missing)	0 (0%)
**Religious service attendance**	
More than 1/week	493 (33%)
1/week	271 (18%)
1-3/month	174 (12%)
A few times a year	255 (17%)
Never	274 (19%)
(Missing)	6 (0.4%)
**Education**	
Up to 8 years	436 (30%)
9-15 years	711 (48%)
16+ years	326 (22%)

Characteristic	N = 1,473[1]
(Missing)	0 (0%)
**Immigration status**	
Born in this country	1,415 (96%)
Born in another country	58 (4.0%)
(Missing)	0 (0%)
**Religious affiliation as an adult (now)**	
Christianity	2 (0.1%)
Islam	1,381 (94%)
Hinduism	0 (0%)
Buddhism	1 (<0.1%)
Judaism	1 (<0.1%)
Sikhism	1 (<0.1%)
Baha'i	0 (0%)
Jainism	0 (0%)
Shinto	0 (0%)
Taoism	0 (0%)
Confucianism	0 (0%)
Primal, Animist, or Folk religion	1 (<0.1%)
Spiritism	0 (0%)
Umbanda, Candomble, and other African-derived religions	0 (0%)
Chinese folk/traditional religion	0 (0%)
Some other religion	1 (<0.1%)
No religion/Atheist/Agnostic	66 (4.5%)
(Missing)	19 (1.3%)
**Parent marital status**	
Parents married	1,325 (90%)
Divorced	57 (3.9%)
Parents were never married	7 (0.5%)
One or both parents had died	61 (4.1%)
(Missing)	23 (1.5%)
**Age 12 religious service attendance**	
At least 1/week	609 (41%)
1-3/month	238 (16%)
<1/month	225 (15%)
Never	383 (26%)
(Missing)	18 (1.2%)
**Relationship with mother**	
Very good	970 (66%)
Somewhat good	401 (27%)
Somewhat bad	48 (3.2%)
Very bad	26 (1.8%)
Does not apply	21 (1.4%)
(Missing)	7 (0.5%)
**Relationship with father**	
Very good	795 (54%)
Somewhat good	425 (29%)
Somewhat bad	73 (5.0%)
Very bad	95 (6.5%)
Does not apply	60 (4.1%)
(Missing)	25 (1.7%)
**Outsider growing up**	
Yes	157 (11%)
No	1,306 (89%)
(Missing)	9 (0.6%)
**Self-reported history of abuse**	
Yes	158 (11%)
No	1,290 (88%)
(Missing)	25 (1.7%)
**Self-rated health growing up**	

**Characteristic**	**N = 1,473**[1]
Excellent	377 (26%)
Very good	410 (28%)
Good	419 (28%)
Fair	220 (15%)
Poor	47 (3.2%)
(Missing)	0 (<0.1%)
**Subjective financial status of family growing up**	
Lived comfortably	498 (34%)
Got by	647 (44%)
Found it difficult	218 (15%)
Found it very difficult	108 (7.3%)
(Missing)	2 (0.1%)
**Religious affiliation at age 12**	
Christianity	1 (<0.1%)
Islam	1,439 (98%)
Hinduism	0 (0%)
Buddhism	0 (0%)
Judaism	1 (<0.1%)
Sikhism	0 (0%)
Baha'i	0 (0%)
Jainism	0 (0%)
Shinto	0 (0%)
Taoism	0 (0%)
Confucianism	0 (0%)
Primal, Animist, or Folk religion	0 (0%)
Spiritism	0 (0%)
Umbanda, Candomble, and other African-derived religions	0 (0%)
Chinese folk/traditional religion	0 (0%)
Some other religion	0 (0%)
No religion/Atheist/Agnostic	13 (0.9%)
(Missing)	19 (1.3%)

[1]n (%)

Table S20b. Means by demographic category for Turkey (N=1473)

		Secure Flourishing Index				Flourishing Index			
Variable	Category	Mean	95% CI	SE	Global p-value	Mean	95% CI	SE	Global p-value
Age group	18-24	6.48	(6.23,6.74)	0.13	0.339	6.19	(5.94,6.44)	0.13	0.089
	25-29	6.57	(6.17,6.96)	0.20		6.27	(5.88,6.67)	0.20	
	30-39	6.47	(6.20,6.74)	0.14		6.17	(5.90,6.44)	0.14	
	40-49	6.57	(6.31,6.82)	0.13		6.26	(5.99,6.52)	0.13	
	50-59	6.96	(6.64,7.29)	0.17		6.75	(6.42,7.08)	0.17	
	60-69	6.55	(6.09,7.00)	0.23		6.34	(5.87,6.81)	0.24	
	70-79	6.34	(5.56,7.13)	0.39		6.12	(5.28,6.96)	0.42	
	80 or older	6.96	(5.95,7.97)	0.44		6.97	(6.05,7.89)	0.40	
Gender	Female	6.58	(6.39,6.77)	0.10	0.897	6.27	(6.08,6.47)	0.10	0.493
	Male	6.59	(6.44,6.75)	0.08		6.36	(6.20,6.52)	0.08	
Marital status	Divorced	6.19	(5.44,6.94)	0.37	0.361	6.01	(5.27,6.76)	0.37	0.562
	Married	6.66	(6.50,6.82)	0.08		6.37	(6.20,6.53)	0.08	
	Separated	6.95	(6.00,7.90)	0.39		6.71	(5.77,7.65)	0.38	
	Single/Never been married	6.46	(6.27,6.66)	0.10		6.23	(6.03,6.42)	0.10	
	Widowed	6.62	(5.83,7.41)	0.39		6.34	(5.55,7.14)	0.40	
Employment	Employed for an employer	6.82	(6.61,7.02)	0.10	7.69e-04	6.51	(6.31,6.72)	0.10	0.001
	Homemaker	6.47	(6.16,6.78)	0.16		6.15	(5.84,6.47)	0.16	
	None of these/Other	5.94	(5.31,6.57)	0.31		5.72	(5.08,6.35)	0.32	
	Retired	6.73	(6.35,7.10)	0.19		6.49	(6.11,6.88)	0.20	
	Self-employed	6.75	(6.46,7.05)	0.15		6.55	(6.25,6.86)	0.15	
	Student	6.27	(5.98,6.56)	0.15		6.07	(5.78,6.36)	0.15	
	Unemployed and looking for a job	5.97	(5.50,6.43)	0.23		5.65	(5.19,6.11)	0.23	
Religious service attendance	A few times a year	6.24	(6.00,6.48)	0.12	1.19e-07	5.89	(5.64,6.13)	0.12	8.89e-09
	More than once a week	6.95	(6.73,7.18)	0.12		6.73	(6.50,6.97)	0.12	
	Never	5.94	(5.63,6.24)	0.16		5.67	(5.37,5.97)	0.15	
	Once a week	6.79	(6.51,7.07)	0.14		6.51	(6.23,6.79)	0.14	

Variable	Category	Secure Flourishing Index Mean	95% CI	SE	Global p-value	Flourishing Index Mean	95% CI	SE	Global p-value
	One to three times a month	6.78	(6.42,7.13)	0.18		6.48	(6.11,6.85)	0.19	
Education	Up to 8	6.43	(6.16,6.71)	0.14	0.109	6.15	(5.87,6.43)	0.14	0.054
	9 to 15	6.76	(6.61,6.91)	0.08		6.52	(6.37,6.67)	0.08	
	16+	6.60	(6.42,6.78)	0.09		6.33	(6.14,6.51)	0.09	
Immigration status	Born in another country	6.84	(6.24,7.44)	0.30	0.388	6.44	(5.79,7.09)	0.32	0.699
	Born in this country	6.58	(6.45,6.70)	0.06		6.31	(6.18,6.44)	0.07	
Religious affiliation	Buddhism	7.90	*	*	< 2e-16	8.25	*	*	< 2e-16
	Christianity	5.53	*	*		4.88	*	*	
	Islam	6.61	(6.48,6.73)	0.07		6.34	(6.20,6.47)	0.07	
	Judaism	9.00	*	*		9.17	*	*	
	No religion/Atheist/ Agnostic	6.17	(5.79,6.55)	0.19		5.90	(5.55,6.24)	0.17	
	Primal, Animist, or Folk religion	6.61	*	*		6.45	*	*	
	Sikhism	6.90	*	*		6.42	*	*	
	Some other religion	7.46	*	*		7.20	*	*	

Note. N=1473; $p < .007 = 0.05/7$ (Bonferroni corrected p-value significance threshold); Mean, estimated group mean; CI, confidence interval for the mean within group; SE, complex survey adjusted standard error or the mean; Global p-value, two-tailed Wald-type test of whether there is evidence of any differences in mean scores among groups of a demographic characteristic. *Estimate is not reported due to multiple-imputation and complex survey adjusted degrees of freedom was less than 1.00 leading to insufficient information to provide an estimate of the uncertainty in the estimate. These groups are removed when estimating the global test of mean differences.

Table S20c. Childhood predictors regression analysis results for Turkey (N=1473)

Variable	Category	Secure Flourishing Index					Flourishing Index				
		Est	95% CI	SE	Est/SD	Global p-value	Est	95% CI	SE	Est/SD	Global p-value
Relationship with mother	(Ref: Very bad/somewhat bad)					0.682					0.764
	Very good/somewhat good	-0.11	(-0.69,0.46)	0.29	-0.06		-0.08	(-0.64,0.48)	0.29	-0.04	
Relationship with father	(Ref: Very bad/somewhat bad)					0.328					0.297
	Very good/somewhat good	-0.18	(-0.55,0.20)	0.19	-0.09		-0.19	(-0.57,0.19)	0.19	-0.10	
Parent marital status	(Ref: Parents married)					0.003					7.63e-04
	Divorced	1.06	(0.49,1.63)	0.29	0.55		1.15	(0.60,1.71)	0.28	0.59	
	Parents were never married	-0.04	(-1.17,1.10)	0.58	-0.02		-0.01	(-1.07,1.04)	0.54	-0.01	
	One or both parents had died	0.13	(-0.53,0.80)	0.34	0.07		0.11	(-0.52,0.73)	0.32	0.06	
Subjective financial status of family growing up	(Ref: Got by)					2.21e-06					1.69e-04
	Lived comfortably	0.45	(0.16,0.74)	0.15	0.23		0.36	(0.08,0.64)	0.14	0.18	
	Found it difficult	-0.26	(-0.59,0.08)	0.17	-0.13		-0.26	(-0.59,0.07)	0.17	-0.13	
	Found it very difficult	-0.96	(-1.51,-0.42)	0.28	-0.50		-0.82	(-1.39,-0.25)	0.29	-0.42	
Abuse	(Ref: No)					0.007					0.004
	Yes	-0.50	(-0.87,-0.13)	0.19	-0.26		-0.53	(-0.90,-0.17)	0.19	-0.27	

Variable	Category	Secure Flourishing Index					Flourishing Index				
		Est	95% CI	SE	Est/SD	Global p-value	Est	95% CI	SE	Est/SD	Global p-value
Outsider growing up	(Ref: No)					0.455					0.281
	Yes	-0.14	(-0.53,0.24)	0.20	-0.07		-0.21	(-0.60,0.18)	0.20	-0.11	
Self-rated health growing up	(Ref: Good)					6.25e-07					1.17e-05
	Excellent	0.22	(-0.12,0.56)	0.17	0.11		0.23	(-0.10,0.56)	0.17	0.12	
	Very good	0.23	(-0.11,0.56)	0.17	0.12		0.21	(-0.12,0.54)	0.17	0.11	
	Fair	-0.34	(-0.72,0.05)	0.20	-0.18		-0.38	(-0.76,0.00)	0.20	-0.19	
	Poor	-1.74	(-2.46,-1.01)	0.37	-0.90		-1.67	(-2.50,-0.84)	0.42	-0.85	
Immigration status	(Ref: Born in this country)					0.531					0.285
	Born in another country	0.19	(-0.40,0.78)	0.30	0.10		0.30	(-0.25,0.86)	0.28	0.15	
Age 12 religious service attendance	(Ref: Never)					3.95e-04					6.17e-04
	At least 1/week	0.56	(0.24,0.88)	0.16	0.29		0.50	(0.18,0.82)	0.16	0.25	
	1-3/month	0.62	(0.25,0.98)	0.19	0.32		0.58	(0.22,0.94)	0.18	0.29	
	< 1/month	0.13	(-0.25,0.52)	0.20	0.07		0.04	(-0.34,0.42)	0.19	0.02	
Year of birth	(Ref: 1998-2005; current age: 18-24)					0.007					0.043
	1993-1998; age 25-29	0.17	(-0.26,0.59)	0.21	0.09		0.15	(-0.28,0.58)	0.22	0.08	
	1983-1993; age 30-39	0.15	(-0.19,0.49)	0.17	0.08		0.13	(-0.21,0.48)	0.18	0.07	
	1973-1983; age 40-49	0.28	(-0.06,0.62)	0.17	0.15		0.28	(-0.06,0.62)	0.17	0.14	
	1963-1973; age 50-59	0.77	(0.39,1.15)	0.19	0.40		0.68	(0.30,1.06)	0.19	0.35	

Variable	Category	Secure Flourishing Index Est	95% CI	SE	Est/SD	Global p-value	Flourishing Index Est	95% CI	SE	Est/SD	Global p-value
	1953-1963; age 60-69	0.31	(-0.21,0.84)	0.27	0.16		0.22	(-0.29,0.73)	0.26	0.11	
	1943-1953; age 70-79	0.05	(-0.72,0.81)	0.39	0.02		-0.04	(-0.78,0.70)	0.38	-0.02	
	1943 or earlier; age 80+	0.78	(-0.04,1.59)	0.42	0.40		0.45	(-0.35,1.26)	0.41	0.23	
Gender	(Ref: Male)					0.959					0.532
	Female	0.00	(-0.25,0.25)	0.13	0.00		0.08	(-0.17,0.33)	0.13	0.04	
Religious affiliation	(Ref: Islam)					0.420					0.517
	Collapsed affiliations with prevalence<3 %	-0.28	(-0.98,0.41)	0.35	-0.15		-0.20	(-0.82,0.41)	0.31	-0.10	
Race/ethnicity	(Ref: Plurality group)										

Note. N=1473; p < .004 (Bonferroni corrected threshold); Est., estimated effect of childhood predictor on flourishing score; CI, confidence interval; SE, standard error of the estimated effect; Est/SD, a more standardized measure of effect size--estimated effect of flourishing divided by standard deviation of flourishing--leads to the interpretation, for those with the given status (e.g., those with a good/very good relationship with mother compared to those with bad/very bad) are 0.XX standard deviations higher/lower on flourishing; the Global p-value corresponds to the two-sided joint parameter Wald-type test of whether any of the levels' parameters are non-zero, for history of abuse, outsider, relationship wtih mother/father, this is test of whether the estimated effect is non-zero, for multiple-category predictors (age, health, financial status), this is a joint test of whether any of these effects are non-zero. Note the confidence interval of the effect estimate can contradict the reported global p-value (e.g., for the single-category effects of relationship with mother). In such cases, the reported confidence interval is more robust with corrected degrees of freedom from the pooling across multiple imputations, whereas the global p-value is based on a Wald-type test and is less robust to uncertainty attributable to multiple imputation.

Table S20d. Sensitivity to unmeasured confounding of childhood predictors in Turkey (N=1473)

Variable	Category	Secure Flourishing Index		Flourishing Index	
		E-value for Estimate	E-value for 95% CI	E-value for Estimate	E-value for 95% CI
Relationship with mother	(Ref: Very bad/somewhat bad)				
	Very good/somewhat good	1.29	1.00	1.23	1.00
Relationship with father	(Ref: Very bad/somewhat bad)				
	Very good/somewhat good	1.39	1.00	1.42	1.00
Parent marital status	(Ref: Parents married)				
	Divorced	2.66	1.82	2.83	1.98
	Parents were never married	1.15	1.00	1.08	1.00
	One or both parents had died	1.32	1.00	1.29	1.00
Subjective financial status of family growing up	(Ref: Got by)				
	Lived comfortably	1.77	1.37	1.65	1.23
	Found it difficult	1.50	1.00	1.51	1.00
	Found it very difficult	2.50	1.73	2.30	1.50
Abuse	(Ref: No)				
	Yes	1.84	1.32	1.89	1.38
Outsider growing up	(Ref: No)				
	Yes	1.34	1.00	1.44	1.00
Self-rated health growing up	(Ref: Good)				
	Excellent	1.45	1.00	1.47	1.00
	Very good	1.46	1.00	1.44	1.00
	Fair	1.62	1.00	1.68	1.00
	Poor	3.91	2.58	3.82	2.34
Immigration status	(Ref: Born in this country)				
	Born in another country	1.41	1.00	1.57	1.00
Age 12 religious service attendance	(Ref: Never)				
	At least 1/week	1.92	1.48	1.84	1.40
	1-3/month	2.00	1.50	1.95	1.45
	< 1/month	1.32	1.00	1.15	1.00
Year of birth	(Ref: 1998-2005; current age: 18-24)				
	1993-1998; age 25-29	1.37	1.00	1.36	1.00
	1983-1993; age 30-39	1.35	1.00	1.33	1.00

Variable	Category	Secure Flourishing Index		Flourishing Index	
		E-value for Estimate	E-value for 95% CI	E-value for Estimate	E-value for 95% CI
	1973-1983; age 40-49	1.54	1.00	1.54	1.00
	1963-1973; age 50-59	2.22	1.69	2.09	1.56
	1953-1963; age 60-69	1.58	1.00	1.46	1.00
	1943-1953; age 70-79	1.17	1.00	1.17	1.00
	1943 or earlier; age 80+	2.22	1.00	1.78	1.00
Gender	(Ref: Male)				
	Female	1.03	1.00	1.24	1.00
Religious affiliation	(Ref: Islam)				
	Collapsed affiliations with prevalence<3%	1.54	1.00	1.43	1.00
Race/ethnicity	(Ref: Plurality group)				

Table S21a. Nationally representative descriptive statistics for United Kingdom

Characteristic	N = 5,368[1]
**Age group**	
1998-2005; age 18-24	490 (9.1%)
1993-1998; age 25-29	391 (7.3%)
1983-1993; age 30-39	946 (18%)
1973-1983; age 40-49	827 (15%)
1963-1973; age 50-59	949 (18%)
1953-1963; age 60-69	889 (17%)
1943-1953; age 70-79	711 (13%)
1943 or earlier; age 80+	163 (3.0%)
(Missing)	1 (<0.1%)
**Gender**	
Male	2,557 (48%)
Female	2,789 (52%)
Other	14 (0.3%)
(Missing)	9 (0.2%)
**Race/Ethnicity**	
Asian	426 (7.9%)
Black	152 (2.8%)
Other	96 (1.8%)
White	4,647 (87%)
(Missing)	47 (0.9%)
**Marital status**	
Married	2,510 (47%)
Separated	114 (2.1%)
Divorced	435 (8.1%)
Widowed	294 (5.5%)
Single, never married	1,456 (27%)
Domestic Partner	512 (9.5%)
(Missing)	48 (0.9%)
**Employment**	
Employed for an employer	2,798 (52%)
Self-employed	469 (8.7%)
Retired	1,262 (24%)
Student	229 (4.3%)
Homemaker	184 (3.4%)
Unemployed and looking for a job	215 (4.0%)
None of these/Other	201 (3.7%)
(Missing)	11 (0.2%)
**Religious service attendance**	
More than 1/week	291 (5.4%)
1/week	499 (9.3%)
1-3/month	293 (5.5%)
A few times a year	1,165 (22%)
Never	3,110 (58%)
(Missing)	10 (0.2%)
**Education**	
Up to 8 years	1,314 (24%)
9-15 years	2,072 (39%)
16+ years	1,974 (37%)
(Missing)	8 (0.2%)
**Immigration status**	
Born in this country	4,659 (87%)
Born in another country	682 (13%)
(Missing)	27 (0.5%)
**Religious affiliation as an adult (now)**	
Christianity	2,750 (51%)
Islam	218 (4.1%)
Hinduism	61 (1.1%)

Characteristic	N = 5,368[1]
Buddhism	30 (0.6%)
Judaism	44 (0.8%)
Sikhism	29 (0.5%)
Baha'i	6 (0.1%)
Jainism	4 (<0.1%)
Shinto	0 (0%)
Taoism	4 (<0.1%)
Confucianism	2 (<0.1%)
Primal, Animist, or Folk religion	36 (0.7%)
Spiritism	0 (0%)
Umbanda, Candomble, and other African-derived religions	0 (0%)
Chinese folk/traditional religion	0 (0%)
Some other religion	61 (1.1%)
No religion/Atheist/Agnostic	2,099 (39%)
(Missing)	25 (0.5%)
**Parent marital status**	
Parents married	4,343 (81%)
Divorced	481 (9.0%)
Parents were never married	315 (5.9%)
One or both parents had died	154 (2.9%)
(Missing)	75 (1.4%)
**Age 12 religious service attendance**	
At least 1/week	1,732 (32%)
1-3/month	733 (14%)
<1/month	903 (17%)
Never	1,972 (37%)
(Missing)	28 (0.5%)
**Relationship with mother**	
Very good	3,435 (64%)
Somewhat good	1,338 (25%)
Somewhat bad	325 (6.1%)
Very bad	150 (2.8%)
Does not apply	92 (1.7%)
(Missing)	27 (0.5%)
**Relationship with father**	
Very good	2,907 (54%)
Somewhat good	1,383 (26%)
Somewhat bad	407 (7.6%)
Very bad	321 (6.0%)
Does not apply	321 (6.0%)
(Missing)	29 (0.5%)
**Outsider growing up**	
Yes	1,017 (19%)
No	4,308 (80%)
(Missing)	43 (0.8%)
**Self-reported history of abuse**	
Yes	864 (16%)
No	4,455 (83%)
(Missing)	49 (0.9%)
**Self-rated health growing up**	
Excellent	2,154 (40%)
Very good	1,736 (32%)
Good	995 (19%)
Fair	332 (6.2%)
Poor	130 (2.4%)
(Missing)	20 (0.4%)
**Subjective financial status of family growing up**	
Lived comfortably	2,552 (48%)
Got by	1,933 (36%)

**Characteristic**	**N = 5,368**[1]
Found it difficult	632 (12%)
Found it very difficult	230 (4.3%)
(Missing)	22 (0.4%)
**Religious affiliation at age 12**	
Christianity	3,461 (64%)
Islam	230 (4.3%)
Hinduism	88 (1.6%)
Buddhism	15 (0.3%)
Judaism	59 (1.1%)
Sikhism	30 (0.6%)
Baha'i	5 (<0.1%)
Jainism	0 (<0.1%)
Shinto	0 (0%)
Taoism	2 (<0.1%)
Confucianism	3 (<0.1%)
Primal, Animist, or Folk religion	22 (0.4%)
Spiritism	0 (0%)
Umbanda, Candomble, and other African-derived religions	0 (0%)
Chinese folk/traditional religion	0 (0%)
Some other religion	24 (0.5%)
No religion/Atheist/Agnostic	1,409 (26%)
(Missing)	21 (0.4%)

[1]n (%)

Table S21b. Means by demographic category for United Kingdom (N=5368)

Variable	Category	Secure Flourishing Index				Flourishing Index			
		Mean	95% CI	SE	Global p-value	Mean	95% CI	SE	Global p-value
Age group	18-24	6.43	(6.18,6.69)	0.13	2.53e-06	6.36	(6.12,6.59)	0.12	2.90e-12
	25-29	7.01	(6.83,7.19)	0.09		6.89	(6.71,7.07)	0.09	
	30-39	6.79	(6.64,6.95)	0.08		6.69	(6.54,6.84)	0.07	
	40-49	6.76	(6.60,6.91)	0.08		6.56	(6.40,6.71)	0.08	
	50-59	6.83	(6.68,6.98)	0.07		6.71	(6.57,6.86)	0.08	
	60-69	6.95	(6.78,7.12)	0.09		6.87	(6.70,7.04)	0.09	
	70-79	7.21	(7.03,7.39)	0.09		7.25	(7.08,7.42)	0.09	
	80 or older	7.46	(7.02,7.89)	0.22		7.56	(7.15,7.97)	0.21	
Gender	Female	6.80	(6.71,6.89)	0.05	0.002	6.68	(6.59,6.76)	0.05	1.32e-04
	Male	6.98	(6.88,7.07)	0.05		6.92	(6.83,7.01)	0.05	
	Other	5.20	(3.42,6.97)	0.78		5.30	(3.53,7.06)	0.77	
Marital status	Divorced	6.47	(6.20,6.73)	0.14	< 2e-16	6.35	(6.09,6.61)	0.13	< 2e-16
	Domestic partner	6.89	(6.71,7.07)	0.09		6.71	(6.53,6.90)	0.09	
	Married	7.29	(7.21,7.38)	0.04		7.22	(7.13,7.30)	0.04	
	Separated	6.15	(5.81,6.49)	0.17		5.91	(5.56,6.27)	0.18	
	Single/Never been married	6.32	(6.18,6.45)	0.07		6.23	(6.10,6.35)	0.07	
	Widowed	6.97	(6.67,7.27)	0.15		7.02	(6.73,7.30)	0.15	
Employment	Employed for an employer	6.93	(6.85,7.02)	0.04	< 2e-16	6.81	(6.73,6.89)	0.04	< 2e-16
	Homemaker	6.90	(6.55,7.25)	0.18		6.62	(6.26,6.98)	0.18	
	None of these/Other	5.24	(4.81,5.67)	0.22		5.06	(4.63,5.49)	0.22	
	Retired	7.19	(7.05,7.32)	0.07		7.22	(7.09,7.35)	0.07	
	Self-employed	6.95	(6.73,7.16)	0.11		6.82	(6.61,7.04)	0.11	
	Student	6.55	(6.25,6.85)	0.15		6.51	(6.25,6.77)	0.13	
	Unemployed and looking for a job	6.06	(5.64,6.48)	0.21		5.87	(5.48,6.26)	0.20	
Religious service attendance	A few times a year	7.05	(6.93,7.16)	0.06	< 2e-16	6.93	(6.81,7.04)	0.06	< 2e-16
	More than once a week	7.85	(7.66,8.05)	0.10		7.76	(7.56,7.95)	0.10	

Variable	Category	Secure Flourishing Index				Flourishing Index			
		Mean	95% CI	SE	Global p-value	Mean	95% CI	SE	Global p-value
	Never	6.60	(6.51,6.69)	0.05		6.52	(6.43,6.61)	0.05	
	Once a week	7.38	(7.21,7.55)	0.09		7.31	(7.14,7.47)	0.08	
	One to three times a month	7.39	(7.11,7.68)	0.14		7.28	(6.99,7.56)	0.14	
Education	Up to 8	6.82	(6.64,7.01)	0.10	1.83e-07	6.78	(6.59,6.97)	0.10	2.34e-09
	9 to 15	7.07	(6.99,7.15)	0.04		6.98	(6.90,7.06)	0.04	
	16+	6.73	(6.65,6.82)	0.04		6.61	(6.52,6.69)	0.04	
Immigration status	Born in another country	7.01	(6.83,7.19)	0.09	0.120	6.90	(6.73,7.06)	0.08	0.174
	Born in this country	6.86	(6.79,6.93)	0.04		6.77	(6.70,6.84)	0.04	
Religious affiliation	Baha'i	6.60	*	*	< 2e-16	5.95	*	*	< 2e-16
	Buddhism	6.91	(6.27,7.55)	0.30		6.86	(6.23,7.50)	0.30	
	Christianity	7.18	(7.10,7.27)	0.05		7.08	(6.99,7.17)	0.05	
	Hinduism	6.95	(6.37,7.53)	0.29		6.90	(6.34,7.47)	0.28	
	Islam	7.23	(6.93,7.53)	0.15		6.98	(6.69,7.27)	0.15	
	Judaism	6.90	(6.10,7.70)	0.39		6.94	(6.08,7.79)	0.42	
	No religion/Atheist/ Agnostic	6.45	(6.35,6.55)	0.05		6.39	(6.29,6.49)	0.05	
	Primal, Animist, or Folk religion	6.52	(5.86,7.18)	0.32		6.47	(5.78,7.16)	0.33	
	Sikhism	7.29	(6.77,7.81)	0.24		7.13	(6.62,7.64)	0.24	
	Some other religion	6.57	(6.16,6.98)	0.20		6.34	(5.92,6.76)	0.21	
	Taoism	6.40	*	*		6.35	*	*	
	Confucianism	9.00	*	*		8.83	*	*	
	Jainism	5.00	*	*		5.00	*	*	

Note. N=5368; $p < .007 = 0.05/7$ (Bonferroni corrected p-value significance threshold); Mean, estimated group mean; CI, confidence interval for the mean within group; SE, complex survey adjusted standard error or the mean; Global p-value, two-tailed Wald-type test of whether there is evidence of any differences in mean scores among groups of a demographic characteristic. *Estimate is not reported due to multiple-imputation and complex survey adjusted degrees of freedom was less than 1.00 leading to insufficient information to provide an estimate of the uncertainty in the estimate. These groups are removed when estimating the global test of mean differences.

Table S21c. Childhood predictors regression analysis results for United Kingdom (N=5368)

		Secure Flourishing Index					Flourishing Index				
Variable	Category	Est	95% CI	SE	Est/SD	Global p-value	Est	95% CI	SE	Est/SD	Global p-value
Relationship with mother	(Ref: Very bad/somewhat bad)					0.029					0.019
	Very good/somewhat good	0.23	(0.02,0.44)	0.11	0.14		0.26	(0.04,0.47)	0.11	0.15	
Relationship with father	(Ref: Very bad/somewhat bad)					0.088					0.078
	Very good/somewhat good	0.15	(-0.02,0.33)	0.09	0.09		0.16	(-0.02,0.34)	0.09	0.10	
Parent marital status	(Ref: Parents married)					0.004					0.034
	Divorced	-0.23	(-0.46,-0.00)	0.12	-0.14		-0.18	(-0.41,0.06)	0.12	-0.10	
	Parents were never married	-0.46	(-0.76,-0.16)	0.15	-0.27		-0.35	(-0.67,-0.04)	0.16	-0.21	
	One or both parents had died	-0.27	(-0.59,0.06)	0.17	-0.16		-0.32	(-0.65,0.01)	0.17	-0.19	
Subjective financial status of family growing up	(Ref: Got by)					8.19e-04					0.022
	Lived comfortably	0.13	(0.00,0.26)	0.07	0.08		0.08	(-0.05,0.21)	0.07	0.05	
	Found it difficult	-0.04	(-0.23,0.15)	0.10	-0.02		-0.02	(-0.22,0.17)	0.10	-0.01	
	Found it very difficult	-0.63	(-1.02,-0.23)	0.20	-0.37		-0.56	(-0.97,-0.14)	0.21	-0.33	
Abuse	(Ref: No)					7.40e-09					7.93e-08
	Yes	-0.54	(-0.72,-0.36)	0.09	-0.32		-0.51	(-0.70,-0.33)	0.10	-0.30	

Variable	Category	Secure Flourishing Index					Flourishing Index				
		Est	95% CI	SE	Est/SD	Global p-value	Est	95% CI	SE	Est/SD	Global p-value
Outsider growing up	(Ref: No)					0.065					0.134
	Yes	-0.15	(-0.32,0.01)	0.08	-0.09		-0.13	(-0.30,0.04)	0.09	-0.08	
Self-rated health growing up	(Ref: Good)					< 2e-16					< 2e-16
	Excellent	0.48	(0.32,0.64)	0.08	0.28		0.47	(0.31,0.63)	0.08	0.28	
	Very good	0.34	(0.19,0.49)	0.08	0.20		0.35	(0.19,0.50)	0.08	0.21	
	Fair	-0.61	(-0.89,-0.34)	0.14	-0.36		-0.70	(-0.99,-0.42)	0.15	-0.42	
	Poor	-0.35	(-0.90,0.20)	0.28	-0.21		-0.39	(-0.94,0.16)	0.28	-0.23	
Immigration status	(Ref: Born in this country)					0.085					0.107
	Born in another country	0.15	(-0.02,0.33)	0.09	0.09		0.16	(-0.03,0.34)	0.10	0.09	
Age 12 religious service attendance	(Ref: Never)					0.005					0.005
	At least 1/week	0.26	(0.10,0.43)	0.09	0.15		0.28	(0.11,0.45)	0.09	0.17	
	1-3/month	0.27	(0.09,0.46)	0.10	0.16		0.28	(0.09,0.47)	0.10	0.17	
	< 1/month	0.10	(-0.07,0.28)	0.09	0.06		0.14	(-0.03,0.32)	0.09	0.09	
Year of birth	(Ref: 1998-2005; current age: 18-24)					7.17e-07					0.005
	1993-1998; age 25-29	0.38	(0.11,0.66)	0.14	0.23		0.43	(0.14,0.71)	0.15	0.25	
	1983-1993; age 30-39	0.22	(-0.02,0.46)	0.12	0.13		0.26	(-0.00,0.52)	0.13	0.15	
	1973-1983; age 40-49	0.08	(-0.17,0.33)	0.13	0.05		0.21	(-0.06,0.47)	0.13	0.12	
	1963-1973; age 50-59	0.24	(-0.01,0.49)	0.13	0.14		0.29	(0.02,0.55)	0.14	0.17	

Variable	Category	Secure Flourishing Index Est	95% CI	SE	Est/SD	Global p-value	Flourishing Index Est	95% CI	SE	Est/SD	Global p-value
	1953-1963; age 60-69	0.33	(0.07,0.60)	0.14	0.20		0.35	(0.07,0.62)	0.14	0.21	
	1943-1953; age 70-79	0.61	(0.34,0.88)	0.14	0.36		0.51	(0.22,0.79)	0.15	0.30	
	1943 or earlier; age 80+	0.93	(0.50,1.36)	0.22	0.55		0.75	(0.28,1.21)	0.24	0.45	
Gender	(Ref: Male)					0.001					0.026
	Female	-0.21	(-0.33,-0.10)	0.06	-0.13		-0.15	(-0.27,-0.03)	0.06	-0.09	
	Other	-0.73	(-2.11,0.64)	0.70	-0.43		-0.91	(-2.30,0.48)	0.71	-0.54	
Religious affiliation	(Ref: No religion/Atheist/Agnostic)					1.94e-04					2.86e-05
	Islam	0.34	(0.02,0.67)	0.17	0.20		0.47	(0.12,0.82)	0.18	0.28	
	Christianity	0.34	(0.19,0.50)	0.08	0.20		0.37	(0.21,0.53)	0.08	0.22	
	Collapsed affiliations with prevalence<3%	0.13	(-0.18,0.43)	0.16	0.08		0.13	(-0.17,0.43)	0.15	0.08	
Race/ethnicity	(Ref: Plurality group)					0.784					0.853
	Non-plurality groups	0.02	(-0.18,0.23)	0.10	0.01		0.02	(-0.20,0.23)	0.11	0.01	

Note. N=5368; p < .004 (Bonferroni corrected threshold); Est., estimated effect of childhood predictor on flourishing score; CI, confidence interval; SE, standard error of the estimated effect; Est/SD, a more standardized measure of effect size--estimated effect of flourishing divided by standard deviation of flourishing--leads to the interpretation, for those with the given status (e.g., those with a good/very good relationship with mother compared to those with bad/very bad) are 0.XX standard deviations higher/lower on flourishing; the Global p-value corresponds to the two-sided joint parameter Wald-type test of whether any of the levels' parameters are non-zero, for history of abuse, outsider, relationship wtih mother/father, this is test of whether the estimated effect is non-zero, for multiple-category predictors (age, health, financial status), this is a joint test of whether any of these effects are non-zero. Note the confidence interval of the effect estimate can contradict the reported global p-value (e.g., for the single-category effects of relationship with mother). In such cases, the reported confidence interval is more robust with corrected degrees of freedom from the pooling across multiple imputations, whereas the global p-value is based on a Wald-type test and is less robust to uncertainty attributable to multiple imputation.

Table S21d. Sensitivity to unmeasured confounding of childhood predictors in United Kingdom (N=5368)

Variable	Category	Secure Flourishing Index		Flourishing Index	
		E-value for Estimate	E-value for 95% CI	E-value for Estimate	E-value for 95% CI
Relationship with mother	(Ref: Very bad/somewhat bad)				
	Very good/somewhat good	1.52	1.12	1.56	1.17
Relationship with father	(Ref: Very bad/somewhat bad)				
	Very good/somewhat good	1.39	1.00	1.40	1.00
Parent marital status	(Ref: Parents married)				
	Divorced	1.52	1.05	1.43	1.00
	Parents were never married	1.89	1.41	1.71	1.16
	One or both parents had died	1.58	1.00	1.65	1.00
Subjective financial status of family growing up	(Ref: Got by)				
	Lived comfortably	1.36	1.05	1.26	1.00
	Found it difficult	1.17	1.00	1.12	1.00
	Found it very difficult	2.16	1.52	2.03	1.37
Abuse	(Ref: No)				
	Yes	2.01	1.72	1.96	1.67
Outsider growing up	(Ref: No)				
	Yes	1.40	1.00	1.35	1.00
Self-rated health growing up	(Ref: Good)				
	Excellent	1.91	1.66	1.89	1.64
	Very good	1.69	1.45	1.70	1.46
	Fair	2.14	1.69	2.28	1.81
	Poor	1.72	1.00	1.77	1.00
Immigration status	(Ref: Born in this country)				
	Born in another country	1.40	1.00	1.39	1.00
Age 12 religious service attendance	(Ref: Never)				
	At least 1/week	1.57	1.29	1.60	1.32
	1-3/month	1.59	1.28	1.60	1.27
	< 1/month	1.31	1.00	1.37	1.00
Year of birth	(Ref: 1998-2005; current age: 18-24)				
	1993-1998; age 25-29	1.76	1.32	1.83	1.37
	1983-1993; age 30-39	1.51	1.00	1.56	1.00

Variable	Category	Secure Flourishing Index		Flourishing Index	
		E-value for Estimate	E-value for 95% CI	E-value for Estimate	E-value for 95% CI
	1973-1983; age 40-49	1.26	1.00	1.48	1.00
	1963-1973; age 50-59	1.53	1.00	1.61	1.12
	1953-1963; age 60-69	1.68	1.23	1.70	1.24
	1943-1953; age 70-79	2.13	1.70	1.95	1.50
	1943 or earlier; age 80+	2.70	1.95	2.35	1.60
Gender	(Ref: Male)				
	Female	1.49	1.29	1.38	1.14
	Other	2.34	1.00	2.63	1.00
Religious affiliation	(Ref: No religion/Atheist/Agnostic)				
	Islam	1.70	1.11	1.89	1.34
	Christianity	1.70	1.45	1.74	1.49
	Collapsed affiliations with prevalence<3%	1.35	1.00	1.35	1.00
Race/ethnicity	(Ref: Plurality group)				
	Non-plurality groups	1.13	1.00	1.10	1.00

Table S22a. Nationally representative descriptive statistics for United States

Characteristic	N = 38,312[1]
**Age group**	
1998-2005; age 18-24	2,682 (7.0%)
1993-1998; age 25-29	3,540 (9.2%)
1983-1993; age 30-39	7,284 (19%)
1973-1983; age 40-49	5,649 (15%)
1963-1973; age 50-59	6,745 (18%)
1953-1963; age 60-69	6,832 (18%)
1943-1953; age 70-79	4,054 (11%)
1943 or earlier; age 80+	1,525 (4.0%)
(Missing)	0 (0%)
**Gender**	
Male	18,222 (48%)
Female	19,562 (51%)
Other	392 (1.0%)
(Missing)	136 (0.4%)
**Race/Ethnicity**	
Asian	2,466 (6.4%)
Black	4,501 (12%)
Hispanic	6,724 (18%)
Other	997 (2.6%)
White	23,605 (62%)
(Missing)	20 (<0.1%)
**Marital status**	
Married	20,360 (53%)
Separated	727 (1.9%)
Divorced	3,636 (9.5%)
Widowed	1,978 (5.2%)
Single, never married	9,431 (25%)
Domestic Partner	1,971 (5.1%)
(Missing)	207 (0.5%)
**Employment**	
Employed for an employer	19,502 (51%)
Self-employed	3,445 (9.0%)
Retired	9,016 (24%)
Student	1,145 (3.0%)
Homemaker	2,049 (5.3%)
Unemployed and looking for a job	1,777 (4.6%)
None of these/Other	1,292 (3.4%)
(Missing)	87 (0.2%)
**Religious service attendance**	
More than 1/week	2,633 (6.9%)
1/week	5,887 (15%)
1-3/month	2,819 (7.4%)
A few times a year	8,870 (23%)
Never	17,975 (47%)
(Missing)	128 (0.3%)
**Education**	
Up to 8 years	210 (0.5%)
9-15 years	25,322 (66%)
16+ years	12,705 (33%)
(Missing)	75 (0.2%)
**Immigration status**	
Born in this country	34,865 (91%)
Born in another country	3,020 (7.9%)
(Missing)	427 (1.1%)
**Religious affiliation as an adult (now)**	
Christianity	22,954 (60%)
Islam	205 (0.5%)

**Characteristic**	**N = 38,312**[1]
Hinduism	167 (0.4%)
Buddhism	336 (0.9%)
Judaism	638 (1.7%)
Sikhism	24 (<0.1%)
Baha'i	13 (<0.1%)
Jainism	18 (<0.1%)
Shinto	12 (<0.1%)
Taoism	93 (0.2%)
Confucianism	8 (<0.1%)
Primal, Animist, or Folk religion	240 (0.6%)
Spiritism	0 (0%)
Umbanda, Candomble, and other African-derived religions	0 (0%)
Chinese folk/traditional religion	0 (0%)
Some other religion	1,267 (3.3%)
No religion/Atheist/Agnostic	11,870 (31%)
(Missing)	467 (1.2%)
**Parent marital status**	
Parents married	27,415 (72%)
Divorced	6,325 (17%)
Parents were never married	3,048 (8.0%)
One or both parents had died	1,024 (2.7%)
(Missing)	500 (1.3%)
**Age 12 religious service attendance**	
At least 1/week	18,609 (49%)
1-3/month	6,644 (17%)
<1/month	5,829 (15%)
Never	7,085 (18%)
(Missing)	145 (0.4%)
**Relationship with mother**	
Very good	20,590 (54%)
Somewhat good	11,525 (30%)
Somewhat bad	3,523 (9.2%)
Very bad	1,874 (4.9%)
Does not apply	694 (1.8%)
(Missing)	106 (0.3%)
**Relationship with father**	
Very good	15,313 (40%)
Somewhat good	12,665 (33%)
Somewhat bad	4,879 (13%)
Very bad	2,604 (6.8%)
Does not apply	2,811 (7.3%)
(Missing)	38 (0.1%)
**Outsider growing up**	
Yes	10,185 (27%)
No	27,714 (72%)
(Missing)	413 (1.1%)
**Self-reported history of abuse**	
Yes	10,026 (26%)
No	28,045 (73%)
(Missing)	242 (0.6%)
**Self-rated health growing up**	
Excellent	16,866 (44%)
Very good	12,108 (32%)
Good	6,444 (17%)
Fair	2,303 (6.0%)
Poor	520 (1.4%)
(Missing)	71 (0.2%)
**Subjective financial status of family growing up**	
Lived comfortably	15,116 (39%)

**Characteristic**	**N = 38,312**[1]
Got by	15,682 (41%)
Found it difficult	5,152 (13%)
Found it very difficult	2,342 (6.1%)
(Missing)	19 (<0.1%)
**Religious affiliation at age 12**	
Christianity	30,444 (79%)
Islam	220 (0.6%)
Hinduism	203 (0.5%)
Buddhism	172 (0.4%)
Judaism	787 (2.1%)
Sikhism	47 (0.1%)
Baha'i	4 (<0.1%)
Jainism	18 (<0.1%)
Shinto	6 (<0.1%)
Taoism	17 (<0.1%)
Confucianism	8 (<0.1%)
Primal, Animist, or Folk religion	67 (0.2%)
Spiritism	0 (0%)
Umbanda, Candomble, and other African-derived religions	0 (0%)
Chinese folk/traditional religion	0 (0%)
Some other religion	359 (0.9%)
No religion/Atheist/Agnostic	5,845 (15%)
(Missing)	115 (0.3%)

[1] n (%)

Table S22b. Means by demographic category for United States (N=38312)

Variable	Category	Secure Flourishing Index				Flourishing Index			
		Mean	95% CI	SE	Global p-value	Mean	95% CI	SE	Global p-value
Age group	18-24	6.37	(6.08,6.66)	0.15	< 2e-16	6.27	(5.99,6.55)	0.14	< 2e-16
	25-29	6.34	(6.12,6.56)	0.11		6.25	(6.05,6.46)	0.10	
	30-39	6.80	(6.70,6.91)	0.05		6.69	(6.59,6.80)	0.06	
	40-49	6.99	(6.90,7.08)	0.05		6.88	(6.79,6.97)	0.05	
	50-59	7.37	(7.31,7.44)	0.03		7.27	(7.20,7.34)	0.03	
	60-69	7.68	(7.64,7.73)	0.02		7.65	(7.60,7.70)	0.02	
	70-79	7.94	(7.89,7.98)	0.02		7.95	(7.90,8.00)	0.02	
	80 or older	7.98	(7.75,8.21)	0.12		8.06	(7.86,8.27)	0.11	
Gender	Female	7.18	(7.12,7.25)	0.03	4.31e-04	7.09	(7.02,7.15)	0.03	3.68e-06
	Male	7.20	(7.14,7.27)	0.03		7.16	(7.09,7.22)	0.03	
	Other	6.19	(5.69,6.69)	0.26		6.03	(5.58,6.48)	0.23	
Marital status	Divorced	7.18	(7.09,7.27)	0.04	< 2e-16	7.06	(6.97,7.15)	0.04	< 2e-16
	Domestic partner	6.61	(6.39,6.82)	0.11		6.49	(6.28,6.69)	0.10	
	Married	7.60	(7.57,7.64)	0.02		7.55	(7.52,7.59)	0.02	
	Separated	6.36	(5.91,6.81)	0.23		6.06	(5.64,6.49)	0.22	
	Single/Never been married	6.38	(6.26,6.50)	0.06		6.29	(6.17,6.41)	0.06	
	Widowed	7.55	(7.38,7.72)	0.09		7.55	(7.39,7.71)	0.08	
Employment	Employed for an employer	7.11	(7.05,7.16)	0.03	< 2e-16	7.03	(6.98,7.09)	0.03	< 2e-16
	Homemaker	7.11	(6.91,7.30)	0.10		7.01	(6.81,7.20)	0.10	
	None of these/Other	6.20	(5.83,6.57)	0.19		5.99	(5.62,6.36)	0.19	
	Retired	7.79	(7.73,7.84)	0.03		7.80	(7.75,7.86)	0.03	
	Self-employed	7.43	(7.28,7.59)	0.08		7.29	(7.14,7.44)	0.08	
	Student	6.39	(6.01,6.76)	0.19		6.34	(5.97,6.70)	0.19	
	Unemployed and looking for a job	5.75	(5.40,6.10)	0.18		5.53	(5.21,5.85)	0.16	
Religious service attendance	A few times a year	7.34	(7.26,7.42)	0.04	< 2e-16	7.24	(7.16,7.32)	0.04	< 2e-16
	More than once a week	8.15	(8.04,8.25)	0.05		8.06	(7.95,8.17)	0.06	

Variable	Category	Secure Flourishing Index				Flourishing Index			
		Mean	95% CI	SE	Global p-value	Mean	95% CI	SE	Global p-value
	Never	6.69	(6.62,6.76)	0.04		6.65	(6.57,6.72)	0.04	
	Once a week	7.88	(7.81,7.94)	0.03		7.78	(7.71,7.85)	0.03	
	One to three times a month	7.46	(7.29,7.62)	0.09		7.39	(7.22,7.56)	0.09	
Education	Up to 8	7.37	(6.80,7.95)	0.29	< 2e-16	6.96	(6.38,7.55)	0.30	< 2e-16
	9 to 15	7.42	(7.39,7.45)	0.02		7.45	(7.42,7.48)	0.02	
	16+	7.06	(6.99,7.12)	0.03		6.94	(6.88,7.01)	0.03	
Immigration status	Born in another country	7.42	(7.24,7.60)	0.09	0.007	7.29	(7.11,7.47)	0.09	0.043
	Born in this country	7.16	(7.11,7.21)	0.02		7.09	(7.05,7.14)	0.02	
Religious affiliation	Baha'i	7.52	(2.99,10.0ǂ)	0.62	< 2e-16	7.67	(3.49,10.0ǂ)	0.57	< 2e-16
	Buddhism	7.12	(6.76,7.48)	0.18		7.04	(6.68,7.41)	0.19	
	Christianity	7.52	(7.46,7.57)	0.03		7.42	(7.37,7.47)	0.03	
	Hinduism	7.51	(7.21,7.82)	0.15		7.55	(7.24,7.86)	0.16	
	Islam	6.72	(6.16,7.29)	0.29		6.73	(6.22,7.23)	0.26	
	Judaism	7.39	(7.17,7.60)	0.10		7.41	(7.12,7.70)	0.13	
	No religion/Atheist/ Agnostic	6.62	(6.54,6.71)	0.04		6.62	(6.53,6.70)	0.04	
	Primal, Animist, or Folk religion	6.21	(5.69,6.74)	0.27		5.89	(5.38,6.39)	0.26	
	Shinto	8.55	(7.20,9.91)	0.62		8.39	(6.85,9.93)	0.70	
	Sikhism	7.29	(6.26,8.32)	0.44		7.25	(6.20,8.30)	0.45	
	Some other religion	6.61	(6.30,6.93)	0.16		6.38	(6.07,6.70)	0.16	
	Taoism	5.92	(5.38,6.47)	0.27		5.52	(4.73,6.32)	0.40	
	Confucianism	7.81	*	*		7.80	*	*	
	Jainism	6.33	(4.34,8.31)	0.52		6.14	(3.76,8.52)	0.62	

Note. N=38312; p < .007 = 0.05/7 (Bonferroni corrected p-value significance threshold); Mean, estimated group mean; CI, confidence interval for the mean within group; SE, complex survey adjusted standard error or the mean; Global p-value, two-tailed Wald-type test of whether there is evidence of any differences in mean scores among groups of a demographic characteristic. *Estimate is not reported due to multiple-imputation and complex survey adjusted degrees of freedom was less than 1.00 leading to insufficient information to provide an estimate of the uncertainty in the estimate. These groups are removed when estimating the global test of mean differences.

Table S22c. Childhood predictors regression analysis results for United States (N=38312)

		Secure Flourishing Index					Flourishing Index				
Variable	Category	Est	95% CI	SE	Est/SD	Global p-value	Est	95% CI	SE	Est/SD	Global p-value
Relationship with mother	(Ref: Very bad/somewhat bad)					0.023					0.003
	Very good/somewhat good	0.15	(0.02,0.28)	0.07	0.09		0.21	(0.07,0.35)	0.07	0.12	
Relationship with father	(Ref: Very bad/somewhat bad)					0.010					1.47e-04
	Very good/somewhat good	0.14	(0.03,0.25)	0.06	0.09		0.21	(0.10,0.32)	0.06	0.13	
Parent marital status	(Ref: Parents married)					0.533					0.772
	Divorced	-0.07	(-0.19,0.05)	0.06	-0.04		-0.02	(-0.14,0.10)	0.06	-0.01	
	Parents were never married	-0.06	(-0.29,0.17)	0.12	-0.04		0.03	(-0.22,0.29)	0.13	0.02	
	One or both parents had died	-0.15	(-0.45,0.16)	0.15	-0.09		-0.13	(-0.44,0.17)	0.16	-0.08	
Subjective financial status of family growing up	(Ref: Got by)					2.19e-06					1.77e-04
	Lived comfortably	0.19	(0.11,0.27)	0.04	0.12		0.16	(0.08,0.24)	0.04	0.10	
	Found it difficult	0.12	(-0.00,0.24)	0.06	0.07		0.17	(0.05,0.30)	0.06	0.10	
	Found it very difficult	-0.13	(-0.35,0.10)	0.12	-0.08		-0.04	(-0.26,0.18)	0.11	-0.02	
Abuse	(Ref: No)					2.97e-08					5.22e-06
	Yes	-0.29	(-0.39,-0.19)	0.05	-0.17		-0.24	(-0.34,-0.14)	0.05	-0.15	

Variable	Category	Secure Flourishing Index					Flourishing Index				
		Est	95% CI	SE	Est/SD	Global p-value	Est	95% CI	SE	Est/SD	Global p-value
Outsider growing up	(Ref: No)					< 2e-16					8.88e-16
	Yes	-0.54	(-0.66,-0.43)	0.06	-0.33		-0.47	(-0.58,-0.35)	0.06	-0.28	
Self-rated health growing up	(Ref: Good)					< 2e-16					< 2e-16
	Excellent	0.82	(0.69,0.94)	0.06	0.50		0.83	(0.70,0.96)	0.07	0.50	
	Very good	0.37	(0.25,0.50)	0.06	0.23		0.40	(0.26,0.53)	0.07	0.24	
	Fair	-0.20	(-0.43,0.04)	0.12	-0.12		-0.20	(-0.44,0.05)	0.12	-0.12	
	Poor	-0.57	(-1.17,0.03)	0.31	-0.34		-0.36	(-1.06,0.33)	0.36	-0.22	
Immigration status	(Ref: Born in this country)					0.015					0.005
	Born in another country	0.21	(0.04,0.38)	0.09	0.13		0.25	(0.07,0.42)	0.09	0.15	
Age 12 religious service attendance	(Ref: Never)					5.86e-04					0.001
	At least 1/week	0.24	(0.10,0.37)	0.07	0.14		0.25	(0.11,0.38)	0.07	0.15	
	1-3/month	0.18	(0.03,0.32)	0.07	0.11		0.17	(0.03,0.32)	0.07	0.11	
	< 1/month	0.05	(-0.09,0.20)	0.07	0.03		0.09	(-0.06,0.24)	0.08	0.06	
Year of birth	(Ref: 1998-2005; current age: 18-24)					< 2e-16					< 2e-16
	1993-1998; age 25-29	0.02	(-0.27,0.32)	0.15	0.02		0.02	(-0.30,0.33)	0.16	0.01	
	1983-1993; age 30-39	0.36	(0.10,0.61)	0.13	0.22		0.37	(0.10,0.64)	0.14	0.22	
	1973-1983; age 40-49	0.45	(0.20,0.71)	0.13	0.28		0.46	(0.19,0.73)	0.14	0.28	
	1963-1973; age 50-59	0.69	(0.44,0.93)	0.13	0.42		0.70	(0.44,0.96)	0.13	0.42	

Variable	Category	Secure Flourishing Index					Flourishing Index				
		Est	95% CI	SE	Est/SD	Global p-value	Est	95% CI	SE	Est/SD	Global p-value
	1953-1963; age 60-69	0.97	(0.73,1.21)	0.12	0.59		0.93	(0.67,1.18)	0.13	0.56	
	1943-1953; age 70-79	1.22	(0.98,1.47)	0.12	0.74		1.14	(0.89,1.40)	0.13	0.69	
	1943 or earlier; age 80+	1.30	(0.99,1.61)	0.16	0.79		1.15	(0.83,1.48)	0.17	0.69	
Gender	(Ref: Male)					0.077					0.016
	Female	0.06	(-0.02,0.13)	0.04	0.03		0.10	(0.02,0.17)	0.04	0.06	
	Other	-0.31	(-0.71,0.09)	0.20	-0.19		-0.23	(-0.69,0.23)	0.23	-0.14	
Religious affiliation	(Ref: No religion/Atheist/Agnostic)					0.297					0.030
	Christianity	0.12	(-0.03,0.27)	0.08	0.07		0.20	(0.04,0.35)	0.08	0.12	
	Collapsed affiliations with prevalence<3%	0.11	(-0.07,0.28)	0.09	0.06		0.10	(-0.09,0.29)	0.10	0.06	
Race/ethnicity	(Ref: Plurality group)					0.616					0.031
	Non-plurality groups	0.02	(-0.06,0.11)	0.04	0.01		0.09	(0.01,0.18)	0.04	0.06	

Note. N=38312; p < .004 (Bonferroni corrected threshold); Est., estimated effect of childhood predictor on flourishing score; CI, confidence interval; SE, standard error of the estimated effect; Est/SD, a more standardized measure of effect size--estimated effect of flourishing divided by standard deviation of flourishing--leads to the interpretation, for those with the given status (e.g., those with a good/very good relationship with mother compared to those with bad/very bad) are 0.XX standard deviations higher/lower on flourishing; the Global p-value corresponds to the two-sided joint parameter Wald-type test of whether any of the levels' parameters are non-zero, for history of abuse, outsider, relationship wtih mother/father, this is test of whether the estimated effect is non-zero, for multiple-category predictors (age, health, financial status), this is a joint test of whether any of these effects are non-zero. Note the confidence interval of the effect estimate can contradict the reported global p-value (e.g., for the single-category effects of relationship with mother). In such cases, the reported confidence interval is more robust with corrected degrees of freedom from the pooling across multiple imputations, whereas the global p-value is based on a Wald-type test and is less robust to uncertainty attributable to multiple imputation.

Table S22d. Sensitivity to unmeasured confounding of childhood predictors in United States (N=38312)

Variable	Category	Secure Flourishing Index		Flourishing Index	
		E-value for Estimate	E-value for 95% CI	E-value for Estimate	E-value for 95% CI
Relationship with mother	(Ref: Very bad/somewhat bad)				
	Very good/somewhat good	1.39	1.11	1.49	1.24
Relationship with father	(Ref: Very bad/somewhat bad)				
	Very good/somewhat good	1.38	1.15	1.50	1.30
Parent marital status	(Ref: Parents married)				
	Divorced	1.24	1.00	1.12	1.00
	Parents were never married	1.22	1.00	1.15	1.00
	One or both parents had died	1.38	1.00	1.37	1.00
Subjective financial status of family growing up	(Ref: Got by)				
	Lived comfortably	1.46	1.33	1.41	1.27
	Found it difficult	1.33	1.00	1.43	1.19
	Found it very difficult	1.35	1.00	1.17	1.00
Abuse	(Ref: No)				
	Yes	1.62	1.45	1.54	1.37
Outsider growing up	(Ref: No)				
	Yes	2.03	1.85	1.91	1.72
Self-rated health growing up	(Ref: Good)				
	Excellent	2.50	2.28	2.54	2.31
	Very good	1.76	1.55	1.80	1.58
	Fair	1.47	1.00	1.47	1.00
	Poor	2.07	1.00	1.74	1.00
Immigration status	(Ref: Born in this country)				
	Born in another country	1.49	1.17	1.56	1.25
Age 12 religious service attendance	(Ref: Never)				
	At least 1/week	1.54	1.30	1.56	1.32
	1-3/month	1.43	1.15	1.43	1.15
	< 1/month	1.21	1.00	1.29	1.00
Year of birth	(Ref: 1998-2005; current age: 18-24)				
	1993-1998; age 25-29	1.13	1.00	1.10	1.00
	1983-1993; age 30-39	1.73	1.30	1.75	1.30

Variable	Category	Secure Flourishing Index E-value for Estimate	Secure Flourishing Index E-value for 95% CI	Flourishing Index E-value for Estimate	Flourishing Index E-value for 95% CI
	1973-1983; age 40-49	1.88	1.47	1.90	1.46
	1963-1973; age 50-59	2.27	1.86	2.30	1.86
	1953-1963; age 60-69	2.79	2.34	2.72	2.26
	1943-1953; age 70-79	3.32	2.81	3.16	2.65
	1943 or earlier; age 80+	3.49	2.84	3.18	2.53
Gender	(Ref: Male)				
	Female	1.21	1.00	1.30	1.13
	Other	1.66	1.00	1.53	1.00
Religious affiliation	(Ref: No religion/Atheist/Agnostic)				
	Christianity	1.34	1.00	1.47	1.18
	Collapsed affiliations with prevalence<3%	1.31	1.00	1.30	1.00
Race/ethnicity	(Ref: Plurality group)				
	Non-plurality groups	1.12	1.00	1.29	1.08

Table S23. Population weighted meta-analysis of demographic means.

Variable	Category	Secure Flourishing Index Est	Secure Flourishing Index 95% CI	Secure Flourishing Index SE	Flourishing Index Est	Flourishing Index 95% CI	Flourishing Index SE
Age group							
	18-24	7.49	(7.44,7.55)	0.027	7.08	(7.03,7.12)	0.025
	25-29	7.43	(7.38,7.48)	0.027	7.00	(6.95,7.05)	0.025
	30-39	7.40	(7.36,7.44)	0.019	6.97	(6.93,7.00)	0.018
	40-49	7.36	(7.32,7.41)	0.022	6.94	(6.90,6.99)	0.022
	50-59	7.37	(7.31,7.42)	0.027	6.98	(6.93,7.03)	0.026
	60-69	7.38	(7.31,7.45)	0.036	7.04	(6.97,7.10)	0.034
	70-79	7.42	(7.29,7.55)	0.065	7.11	(6.99,7.23)	0.062
	80 or older	7.44	(7.09,7.79)	0.180	7.27	(6.93,7.62)	0.174
Gender							
	Male	7.45	(7.42,7.48)	0.015	7.08	(7.05,7.11)	0.014
	Female	7.49	(7.46,7.52)	0.015	7.05	(7.02,7.08)	0.014
	Other	6.34	(5.95,6.73)	0.199	6.07	(5.65,6.49)	0.214
Marital status							
	Married	7.59	(7.57,7.62)	0.013	7.18	(7.16,7.20)	0.012
	Separated	6.81	(6.51,7.10)	0.150	6.42	(6.14,6.69)	0.141
	Divorced	7.12	(6.77,7.48)	0.180	6.79	(6.50,7.07)	0.146

Variable	Category	Secure Flourishing Index			Flourishing Index		
		Est	95% CI	SE	Est	95% CI	SE
	Widowed	7.23	(7.11,7.34)	0.059	6.91	(6.79,7.02)	0.059
	Domestic partner	7.23	(7.02,7.44)	0.105	6.86	(6.69,7.03)	0.088
	Single, never married	7.40	(7.35,7.44)	0.023	7.01	(6.97,7.05)	0.021
Employment status							
	Employed for an employer	7.43	(7.39,7.48)	0.022	7.05	(7.01,7.09)	0.021
	Self-employed	7.57	(7.53,7.62)	0.024	7.16	(7.12,7.20)	0.021
	Retired	7.55	(7.41,7.68)	0.069	7.26	(7.13,7.38)	0.065
	Student	7.51	(7.43,7.59)	0.041	7.12	(7.04,7.19)	0.040
	Homemaker	7.47	(7.42,7.51)	0.022	7.03	(6.99,7.07)	0.021
	Unemployed and looking for a job	6.98	(6.89,7.07)	0.046	6.54	(6.46,6.62)	0.041
	None of these/other	7.13	(7.02,7.24)	0.057	6.73	(6.62,6.83)	0.052
Education							
	Up to 8 years	7.42	(7.36,7.49)	0.034	6.97	(6.91,7.04)	0.035
	9-15 years	7.60	(7.55,7.64)	0.022	7.19	(7.16,7.23)	0.020
	16+ years	7.78	(7.67,7.88)	0.051	7.43	(7.33,7.52)	0.047
Religious service attendance							
	>1/week	7.78	(7.74,7.83)	0.022	7.38	(7.34,7.42)	0.021
	1/week	7.71	(7.67,7.75)	0.021	7.28	(7.24,7.32)	0.020
	1-3/month	7.50	(7.45,7.54)	0.025	7.10	(7.05,7.14)	0.023
	A few times a year	7.42	(7.37,7.46)	0.023	7.01	(6.97,7.05)	0.021
	Never	7.18	(7.11,7.24)	0.033	6.81	(6.75,6.87)	0.031
Immigration status							
	Born in another country	7.16	(6.98,7.33)	0.089	6.81	(6.62,6.99)	0.094
	Born in this country	7.46	(7.44,7.49)	0.012	7.06	(7.04,7.08)	0.011

Table S24. Population weighted meta-analysis of childhood predictors.

		Secure Flourishing Index			Flourishing Index		
Variable	Category	Est	95% CI	SE	Est	95% CI	SE
Relationship with mother	(Ref: Very bad/somewhat bad)						
	Very good/somewhat good	0.18	(0.06,0.31)	0.066	0.23	(0.09,0.37)	0.070
Relationship with father	(Ref: Very bad/somewhat bad)						
	Very good/somewhat good	0.06	(-0.05,0.16)	0.055	0.11	(-0.00,0.22)	0.058
Parent marital status	(Ref: Parents married)						
	Divorced	0.06	(-0.05,0.17)	0.057	0.11	(-0.02,0.24)	0.067
	Single, never married	-0.09	(-0.17,-0.01)	0.042	-0.05	(-0.13,0.04)	0.043
	One or both parents had died	-0.08	(-0.16,0.00)	0.041	0.00	(-0.08,0.08)	0.042
Subjective financial status of family growing up	(Ref: Got by)						
	Lived comfortably	0.18	(0.13,0.22)	0.022	0.14	(0.09,0.19)	0.024
	Found it difficult	-0.14	(-0.20,-0.09)	0.028	-0.12	(-0.18,-0.06)	0.030
	Found it very difficult	-0.33	(-0.40,-0.26)	0.037	-0.30	(-0.37,-0.22)	0.038
Abuse	(Ref: No)						
	Yes	-0.31	(-0.37,-0.25)	0.029	-0.29	(-0.35,-0.23)	0.030
Outsider growing up	(Ref: No)						
	Yes	-0.23	(-0.29,-0.17)	0.030	-0.22	(-0.28,-0.16)	0.032
Self-rated health growing up	(Ref: Good)						
	Excellent	0.33	(0.27,0.39)	0.029	0.32	(0.26,0.38)	0.032
	Very good	0.17	(0.12,0.22)	0.024	0.16	(0.11,0.21)	0.025
	Fair	-0.21	(-0.27,-0.15)	0.031	-0.24	(-0.30,-0.18)	0.032
	Poor	-0.37	(-0.50,-0.25)	0.064	-0.34	(-0.47,-0.20)	0.069
Immigration status	(Ref: Born in this country)						

Variable	Category	Secure Flourishing Index			Flourishing Index		
		Est	95% CI	SE	Est	95% CI	SE
	Born in another country	-0.17	(-0.36,0.02)	0.096	-0.21	(-0.40,-0.03)	0.094
Age 12 religious service attendance	(Ref: Never)						
	At least 1/week	0.21	(0.15,0.27)	0.032	0.23	(0.16,0.29)	0.034
	1-3/month	0.17	(0.11,0.24)	0.035	0.18	(0.11,0.25)	0.037
	< 1/month	0.08	(0.02,0.15)	0.035	0.08	(0.00,0.15)	0.038
Year of birth	(Ref: 1998-2005; age 18-24)						
	1993-1998; age 25-29	-0.06	(-0.13,0.00)	0.032	-0.06	(-0.12,0.01)	0.034
	1983-1993; age 30-39	-0.09	(-0.14,-0.03)	0.028	-0.08	(-0.14,-0.02)	0.031
	1973-1983; age 40-49	-0.11	(-0.17,-0.05)	0.032	-0.12	(-0.19,-0.05)	0.034
	1963-1973; age 50-59	-0.07	(-0.14,-0.00)	0.036	-0.11	(-0.19,-0.04)	0.038
	1953-1963; age 60-69	-0.03	(-0.11,0.05)	0.041	-0.11	(-0.20,-0.03)	0.043
	1943-1953; age 70-79	0.02	(-0.11,0.15)	0.065	-0.10	(-0.23,0.03)	0.068
	1943 or earlier; age 80+	0.16	(-0.17,0.49)	0.170	-0.11	(-0.46,0.23)	0.178
Gender	(Ref: Male)						
	Female	-0.00	(-0.04,0.03)	0.018	0.07	(0.03,0.11)	0.019
	Other	-0.43	(-0.79,-0.08)	0.179	-0.47	(-0.79,-0.15)	0.165
Father.na	(Ref:)						
Mother.na	(Ref:)						
Race_plurality	(Ref:)						
	Father missing flag	0.04	(-0.04,0.13)	0.043	0.05	(-0.04,0.13)	0.046
	Mother missing flag	0.05	(-0.06,0.15)	0.053	0.06	(-0.05,0.17)	0.055
	Non-plurality groups	-0.02	(-0.07,0.03)	0.026	0.02	(-0.03,0.07)	0.027

Table S25. Sensitivity to unmeasured confounding of population weighted meta-analysis of childhood predictors.

Variable	Category	Secure Flourishing Index		Flourishing Index	
		E-value for Estimate	E-value for 95% CI	E-value for Estimate	E-value for 95% CI
Relationship with mother	(Ref: Very bad/somewhat bad)				
	Very good/somewhat good	1.47	1.22	1.55	1.30
Relationship with father	(Ref: Very bad/somewhat bad)				
	Very good/somewhat good	1.22	1.00	1.33	1.00
Parent marital status	(Ref: Parents married)				
	Divorced	1.22	1.00	1.33	1.00
	Single, never married	1.29	1.07	1.20	1.00
	One or both parents had died	1.27	1.00	1.03	1.00
Subjective financial status of family growing up	(Ref: Got by)				
	Lived comfortably	1.45	1.38	1.38	1.29
	Found it difficult	1.39	1.29	1.34	1.22
	Found it very difficult	1.71	1.59	1.66	1.53
Abuse	(Ref: No)				
	Yes	1.68	1.59	1.65	1.54
Outsider growing up	(Ref: No)				
	Yes	1.54	1.44	1.53	1.42
Self-rated health growing up	(Ref: Good)				
	Excellent	1.72	1.62	1.70	1.59
	Very good	1.44	1.35	1.42	1.33
	Fair	1.51	1.40	1.56	1.45
	Poor	1.79	1.58	1.72	1.49
Immigration status	(Ref: Born in this country)				
	Born in another country	1.44	1.00	1.51	1.14
Age 12 religious service attendance	(Ref: Never)				
	At least 1/week	1.51	1.40	1.54	1.42
	1-3/month	1.45	1.32	1.46	1.33
	< 1/month	1.28	1.10	1.26	1.02
Year of birth	(Ref: 1998-2005; age 18-24)				
	1993-1998; age 25-29	1.23	1.00	1.22	1.00
	1983-1993; age 30-39	1.29	1.16	1.27	1.12
	1973-1983; age 40-49	1.33	1.20	1.35	1.21

Variable	Category	Secure Flourishing Index		Flourishing Index	
		E-value for Estimate	E-value for 95% CI	E-value for Estimate	E-value for 95% CI
	1963-1973; age 50-59	1.26	1.04	1.34	1.18
	1953-1963; age 60-69	1.15	1.00	1.33	1.14
	1943-1953; age 70-79	1.12	1.00	1.31	1.00
	1943 or earlier; age 80+	1.42	1.00	1.34	1.00
Gender	(Ref: Male)				
	Female	1.03	1.00	1.25	1.16
	Other	1.89	1.28	1.95	1.40

Table S26. Random effects meta-analysis of composite flourishing without financial indicators means by demographic category

Variable	Category	Est	95% CI	SE	Prediction Interval LL	Prediction Interval UL	Heterogeneity (τ)	I^2	Global p-value
Age group									6.05e-16***
	18-24	7.05	(6.78,7.33)	0.14	5.68	8.07	0.65	99.2	
	25-29	7.04	(6.78,7.30)	0.13	5.58	8.14	0.60	98.8	
	30-39	7.03	(6.79,7.28)	0.13	5.51	8.18	0.59	99.3	
	40-49	7.05	(6.81,7.29)	0.12	5.46	8.15	0.57	99.1	
	50-59	7.14	(6.93,7.36)	0.11	5.61	8.03	0.51	98.7	
	60-69	7.25	(7.04,7.45)	0.11	6.14	8.02	0.48	98.5	
	70-79	7.33	(7.11,7.56)	0.12	6.38	8.19	0.51	98.1	
	80 or older	7.36	(7.11,7.62)	0.13	6.16	8.10	0.52	92.8	
Gender									1.14e-15***
	Male	7.19	(6.99,7.40)	0.11	5.76	8.08	0.49	99.5	
	Female	7.12	(6.92,7.32)	0.10	6.02	8.12	0.47	99.5	
	Other	6.09	(5.69,6.50)	0.21	5.38	7.33	0.58	78.4	
Marital status									4.87e-16***
	Married	7.34	(7.15,7.54)	0.10	6.19	8.16	0.46	99.5	
	Separated	6.77	(6.54,6.99)	0.11	5.83	7.76	0.50	93.1	
	Divorced	6.85	(6.64,7.05)	0.10	5.60	7.81	0.46	95.3	
	Widowed	7.17	(6.97,7.38)	0.10	6.27	7.98	0.45	94.4	
	Domestic partner	7.01	(6.75,7.26)	0.13	5.38	7.96	0.56	98.3	
	Single, never married	6.92	(6.63,7.21)	0.15	5.17	7.95	0.70	99.6	
Employment status									6.04e-16***
	Employed for an employer	7.20	(6.99,7.42)	0.11	5.75	8.03	0.51	99.5	
	Self-employed	7.28	(7.08,7.48)	0.10	6.00	8.22	0.46	98.5	
	Retired	7.32	(7.12,7.52)	0.10	6.39	8.16	0.46	98.3	
	Student	7.10	(6.83,7.38)	0.14	6.09	8.23	0.65	98.4	
	Homemaker	7.05	(6.85,7.25)	0.10	6.20	8.14	0.46	97.6	
	Unemployed and looking for a job	6.51	(6.15,6.86)	0.18	4.40	7.74	0.83	98.9	
	None of these/other	6.69	(6.33,7.04)	0.18	5.12	8.00	0.82	97.6	
Education									9.66e-16***
	Up to 8 years	7.05	(6.81,7.30)	0.12	5.20	8.12	0.57	98.8	
	9-15 years	7.16	(6.94,7.38)	0.11	5.78	8.07	0.52	99.7	
	16+ years	7.35	(7.15,7.54)	0.10	6.31	8.11	0.45	99.1	
Religious service attendance									4.04e-16***

Variable	Category	Est	95% CI	SE	Prediction Interval LL	Prediction Interval UL	Heterogeneity (τ)	I^2	Global p-value
	>1/week	7.67	(7.44,7.89)	0.12	6.76	8.98	0.53	98.4	
	1/week	7.42	(7.27,7.57)	0.08	6.58	8.08	0.35	97.6	
	1-3/month	7.21	(7.03,7.39)	0.09	6.13	7.97	0.41	96.9	
	A few times a year	7.08	(6.89,7.28)	0.10	5.93	7.83	0.45	98.8	
	Never	6.86	(6.65,7.07)	0.11	5.72	8.00	0.50	99.3	
Immigration status									1.95e-08***
	Born in another country	7.02	(6.86,7.18)	0.08	6.08	7.56	0.34	89.6	
	Born in this country	7.16	(6.95,7.36)	0.11	5.89	8.10	0.49	99.7	

Note. N = 202,898. *p < .05; **p < .007 (Bonferroni corrected threshold); ǂGroup is very small (<0.1% of the observed sample) within several countries leading to large uncertainty in this estimate—be cautious about interpreting this estimate; LL=lower limits of prediction interval; UL=upper limit of prediction interval; prediction interval is the range of likely values of the estimate for a randomly selected country; τ is the standard deviation of the distribution of means across countries, which is an indicator of cross-national heterogeneity; I2 is an estimate of the variability in means due to heterogeneity across countries vs. sampling variability which is not uncommonly nearly 100% when there is nice precision in estimated mean within country; and the Global p-value corresponds to a test of the null hypothesis that there are no differences between the groups for that sociodemographic characteristic in all of the 22 countries. Composite flourishing outcome is mean of individual item responses (see Table 2).

Table S27. Random effects meta-analysis of regression of composite flourishing without financial indicators on childhood predictors.

Variable	Category	Est	95% CI	SE	Estimated Proportion of Est < -0.10	Estimated Proportion of Est > 0.10	Heterogeneity (τ)	I^2	Global p-value
Relationship with mother	(Ref: Very bad/somewhat bad)								8.86e-06***
	Very good/somewhat good	0.20	(0.12,0.27)	0.04	0.00	0.77	0.12	58.8	
Relationship with father	(Ref: Very bad/somewhat bad)								4.73e-15***
	Very good/somewhat good	0.14	(0.08,0.19)	0.03	0.00	0.55	0.10	59.1	
Parent marital status	(Ref: Parents married)								9.30e-07***
	Divorced	-0.04	(-0.15,0.07)	0.06	0.36	0.23	0.23	86.6	
	Single, never married	-0.08	(-0.17,0.02)	0.05	0.32	0.09	0.18	78.8	
	One or both parents had died	-0.05	(-0.15,0.05)	0.05	0.41	0.14	0.19	70.2	
Subjective financial status of family growing up	(Ref: Got by)								4.04e-15***
	Lived comfortably	0.17	(0.10,0.23)	0.03	0.00	0.55	0.14	88.7	
	Found it difficult	-0.07	(-0.11,-0.02)	0.02	0.41	0.05	0.08	58.3	
	Found it very difficult	-0.19	(-0.28,-0.11)	0.05	0.68	0.05	0.16	64.4	
Abuse	(Ref: No)								2.36e-13***
	Yes	-0.33	(-0.39,-0.27)	0.03	1.00	0.00	0.12	71.1	
Outsider growing up	(Ref: No)								1.27e-14***
	Yes	-0.26	(-0.32,-0.19)	0.03	0.86	0.00	0.13	73.0	
Self-rated health growing up	(Ref: Good)								5.38e-16***
	Excellent	0.48	(0.32,0.64)	0.08	0.00	0.86	0.37	96.8	
	Very good	0.25	(0.16,0.33)	0.04	0.00	0.82	0.19	90.2	

Variable	Category	Est	95% CI	SE	Estimated Proportion of Est < -0.10	Estimated Proportion of Est > 0.10	Heterogeneity (τ)	I^2	Global p-value
	Fair	-0.23	(-0.32,-0.14)	0.05	0.77	0.00	0.19	82.4	
	Poor	-0.31	(-0.50,-0.11)	0.10	0.73	0.14	0.40	83.9	
Immigration status	(Ref: Born in this country)								4.84e-15***
	Born in another country	0.02	(-0.12,0.16)	0.07	0.32	0.45	0.27	84.5	
Age 12 religious service attendance	(Ref: Never)								3.24e-15***
	At least 1/week	0.29	(0.20,0.38)	0.05	0.00	0.86	0.19	82.6	
	1-3/month	0.22	(0.12,0.31)	0.05	0.00	0.68	0.20	84.3	
	< 1/month	0.10	(0.04,0.15)	0.03	0.00	0.41	0.10	64.7	
Year of birth	(Ref: 1998-2005; age 18-24)								6.04e-16***
	1993-1998; age 25-29	0.03	(-0.04,0.10)	0.04	0.23	0.36	0.14	74.3	
	1983-1993; age 30-39	0.05	(-0.05,0.15)	0.05	0.23	0.50	0.23	90.9	
	1973-1983; age 40-49	0.05	(-0.08,0.19)	0.07	0.32	0.55	0.31	93.7	
	1963-1973; age 50-59	0.11	(-0.07,0.29)	0.09	0.27	0.59	0.41	95.6	
	1953-1963; age 60-69	0.16	(-0.05,0.37)	0.11	0.32	0.59	0.49	96.0	
	1943-1953; age 70-79	0.18	(-0.12,0.48)	0.15	0.36	0.55	0.69	96.8	
	1943 or earlier; age 80+	0.17	(-0.17,0.51)	0.17	0.41	0.50	0.76	93.5	
Gender	(Ref: Male)								4.40e-15***
	Female	0.04	(-0.01,0.08)	0.02	0.05	0.14	0.10	83.0	
	Other	-0.28	(-0.59,0.02)	0.15	0.78	0.22	0.53	79.5	

Variable	Category	Est	95% CI	SE	Estimated Proportion of Est < -0.10	Estimated Proportion of Est > 0.10	Heterogeneity (τ)	I^2	Global p-value

Note. N = 202,898. *p < .05; **p < .004 (Bonferroni corrected threshold); ǂGroup is very small (<0.1% of the observed sample) within several countries leading to high uncertainty in this estimate—be cautious about interpreting this estimate; CI = confidence interval; the estimated proportion of effects is the estimated proportion of effects above (or below) a threshold based on the calibrated effect sizes (Mathur & VanderWeele, 2020); I2 is an estimate of the variability in means due to heterogeneity across countries vs. sampling variability; the Global p-value corresponds to the joint test of the null hypothesis that the country-specific joint parameter Wald tests (all parameters within variable groups are zero) are all null all 22 countries; and additional details of heterogeneity of effects are available in the forest plots of our online supplemental material. Composite flourishing outcome is mean of individual item responses (see Table 2).

Table S28. Sensitivity of meta-analyzed childhood predictors to unmeasured confounding of composite flourishing without financial indicators.

Variable	Category	Evalue for Estimate	Evalue for 95% CI
Relationship with mother	(Ref: Very bad/somewhat bad)		
	Very good/somewhat good	1.48	1.35
Relationship with father	(Ref: Very bad/somewhat bad)		
	Very good/somewhat good	1.38	1.27
Parent marital status	(Ref: Parents married)		
	Divorced	1.18	1.00
	Single, never married	1.26	1.00
	One or both parents had died	1.20	1.00
Subjective financial status of family growing up	(Ref: Got by)		
	Lived comfortably	1.44	1.32
	Found it difficult	1.24	1.13
	Found it very difficult	1.48	1.32
Abuse	(Ref: No)		
	Yes	1.72	1.62
Outsider growing up	(Ref: No)		
	Yes	1.59	1.47
Self-rated health growing up	(Ref: Good)		
	Excellent	1.97	1.70
	Very good	1.57	1.43
	Fair	1.55	1.39
	Poor	1.67	1.33
Immigration status	(Ref: Born in this country)		
	Born in another country	1.13	1.00
Age 12 religious service attendance	(Ref: Never)		
	At least 1/week	1.64	1.49
	1-3/month	1.52	1.35
	< 1/month	1.30	1.17
Year of birth	(Ref: 1998-2005; age 18-24)		
	1993-1998; age 25-29	1.14	1.00
	1983-1993; age 30-39	1.21	1.00
	1973-1983; age 40-49	1.21	1.00
	1963-1973; age 50-59	1.33	1.00
	1953-1963; age 60-69	1.42	1.00
	1943-1953; age 70-79	1.46	1.00

Variable	Category	Evalue for Estimate	Evalue for 95% CI
	1943 or earlier; age 80+	1.44	1.00
Gender	(Ref: Male)		
	Female	1.18	1.00
	Other	1.63	1.00

Note. N = 202,898; the E-value is the minimum strength of the association an unmeasured confounder must have with both the outcome and the predictor, above and beyond all measured covariates, for an unmeasured confounder to explain away an association (VanderWeele & Ding, 2017, p. 269-270); and ǂGroup is very small (<0.1% of the observed sample) within several countries leading to high uncertainty in this estimate—be cautious about interpreting this estimate.

Part 2. Forest plots of means by demographic groups.

a. Secure Flourishing Index; b. Flourishing Index (does not contain financial items)

Figure S1. Heterogeneity in means scores across countries within group: Age group-18-24. (a) Flourishing with financial indicators (12 items) [left panel]; (b) Flourishing without financial indicators (10 items) [right panel]. N=202,898, subgroup means and standard errors are computed accounting for the complex sampling design using all data simultaneously. Analyses conducted: Random-effects meta-analysis of country-specific means. Squares represent the the point estimate (mean) for each country. The lines represented the +/-1.96*SE, standard error, around the mean; the overall pooled mean is represented by the diamond. The reported p-value for Q-statistics is necessarily 1-sided because of the use of the chi-squared distribution to test whether heterogeneity is greater than zero (i.e., a two-sided test is not applicable). No adjustments for multiple testing were made.

Figure S1a Forest plot for `Age group`-`18-24`

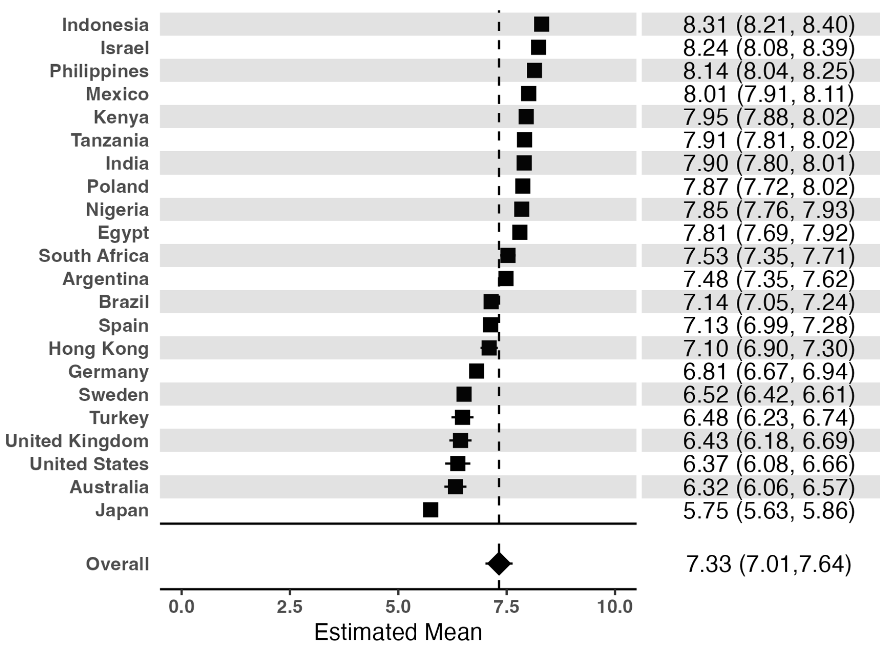

τ=0.746; Q(df=21)=2788.83, p=<2e-16;Q-profile 95% CI [0.553, 1.014]; I^2=99.33;

Figure S1b. Forest plot for `Age group`-`18-24`

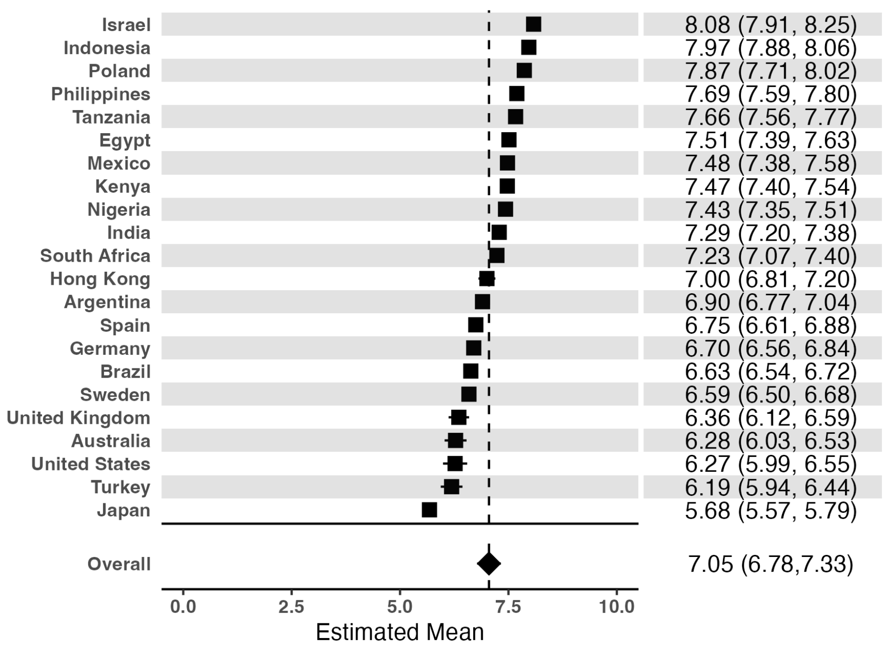

τ=0.653; Q(df=21)=2125.23, p=<2e-16;Q-profile 95% CI [0.483, 0.889]; I^2=99.17;

Figure S2. Heterogeneity in means scores across countries within group: Age group-25-29. (a) Flourishing with financial indicators (12 items) [left panel]; (b) Flourishing without financial indicators (10 items) [right panel]. N=202,898, subgroup means and standard errors are computed accounting for the complex sampling design using all data simultaneously. Analyses conducted: Random-effects meta-analysis of country-specific means. Squares represent the the point estimate (mean) for each country. The lines represented the +/-1.96*SE, standard error, around the mean; the overall pooled mean is represented by the diamond. The reported p-value for Q-statistics is necessarily 1-sided because of the use of the chi-squared distribution to test whether heterogeneity is greater than zero (i.e., a two-sided test is not applicable). No adjustments for multiple testing were made.

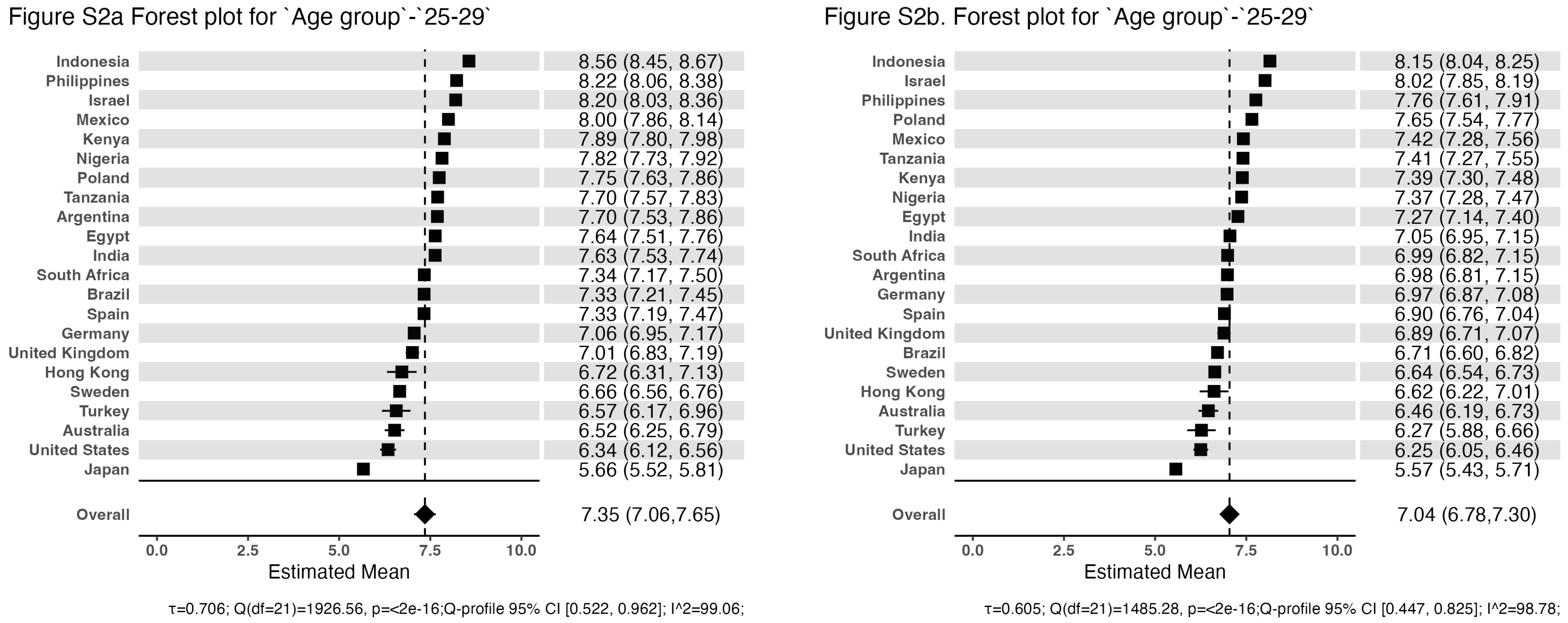

Figure S3. Heterogeneity in means scores across countries within group: Age group-30-39. (a) Flourishing with financial indicators (12 items) [left panel]; (b) Flourishing without financial indicators (10 items) [right panel]. N=202,898, subgroup means and standard errors are computed accounting for the complex sampling design using all data simultaneously. Analyses conducted: Random-effects meta-analysis of country-specific means. Squares represent the the point estimate (mean) for each country. The lines represented the +/-1.96*SE, standard error, around the mean; the overall pooled mean is represented by the diamond. The reported p-value for Q-statistics is necessarily 1-sided because of the use of the chi-squared distribution to test whether heterogeneity is greater than zero (i.e., a two-sided test is not applicable). No adjustments for multiple testing were made.

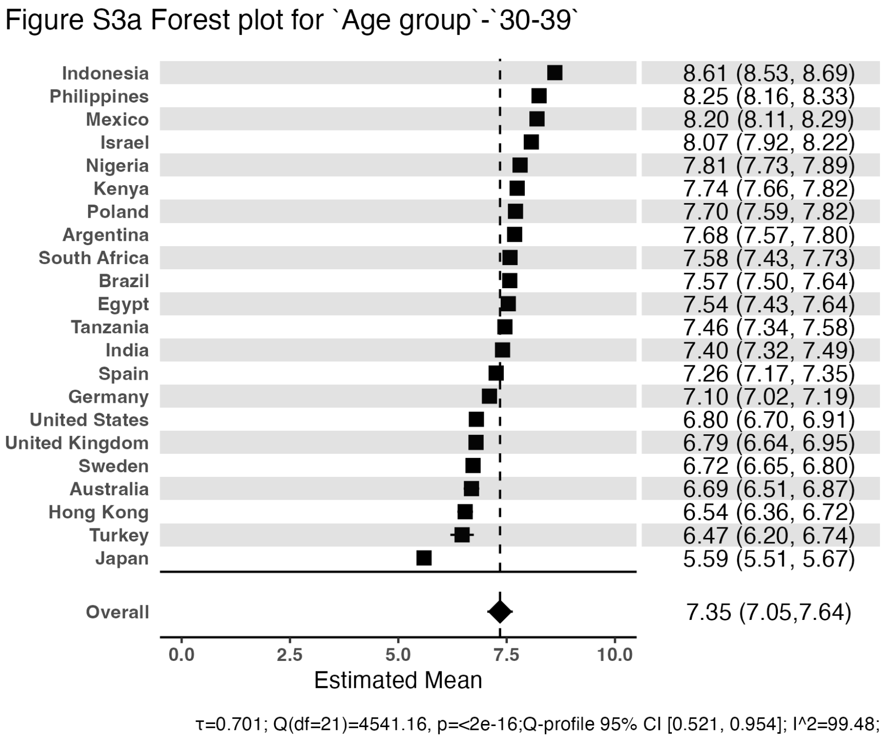

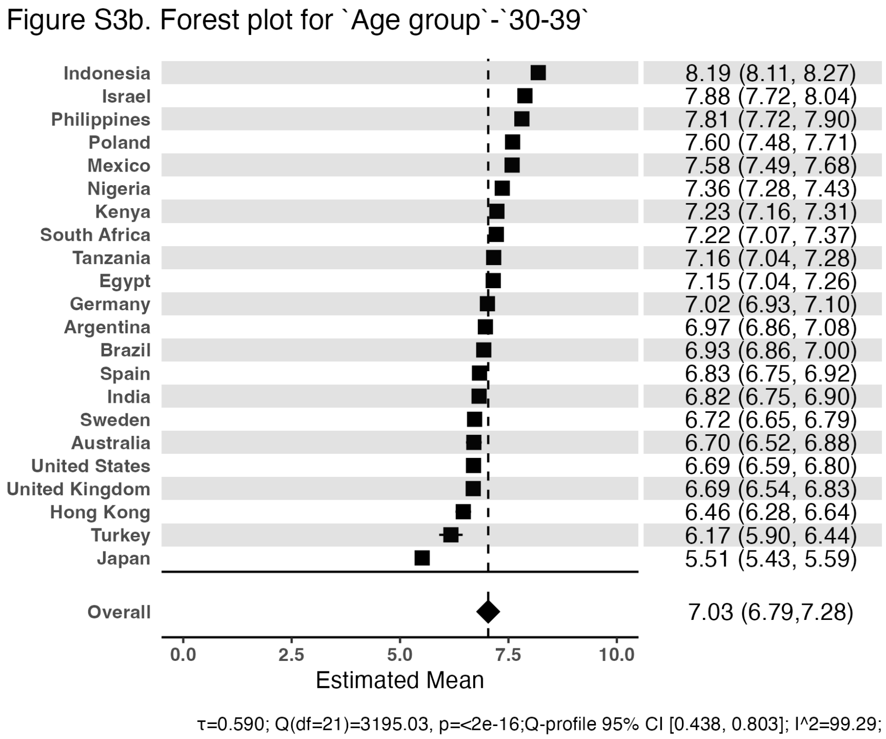

Figure S4. Heterogeneity in means scores across countries within group: Age group-40-49. (a) Flourishing with financial indicators (12 items) [left panel]; (b) Flourishing without financial indicators (10 items) [right panel]. N=202,898, subgroup means and standard errors are computed accounting for the complex sampling design using all data simultaneously. Analyses conducted: Random-effects meta-analysis of country-specific means. Squares represent the the point estimate (mean) for each country. The lines represented the +/-1.96*SE, standard error, around the mean; the overall pooled mean is represented by the diamond. The reported p-value for Q-statistics is necessarily 1-sided because of the use of the chi-squared distribution to test whether heterogeneity is greater than zero (i.e., a two-sided test is not applicable). No adjustments for multiple testing were made.

Figure S4a Forest plot for `Age group`-`40-49`

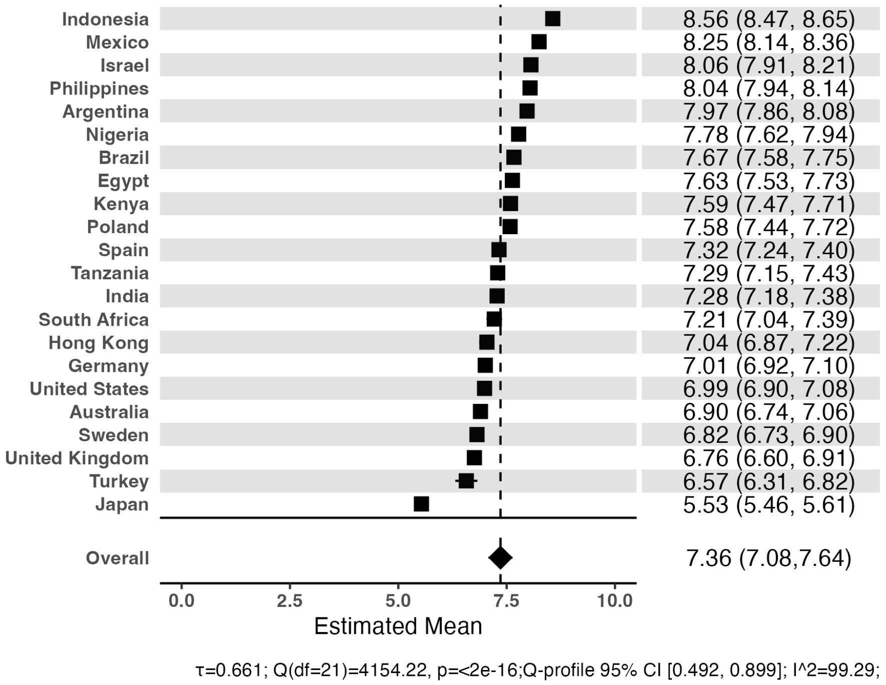

Figure S4b. Forest plot for `Age group`-`40-49`

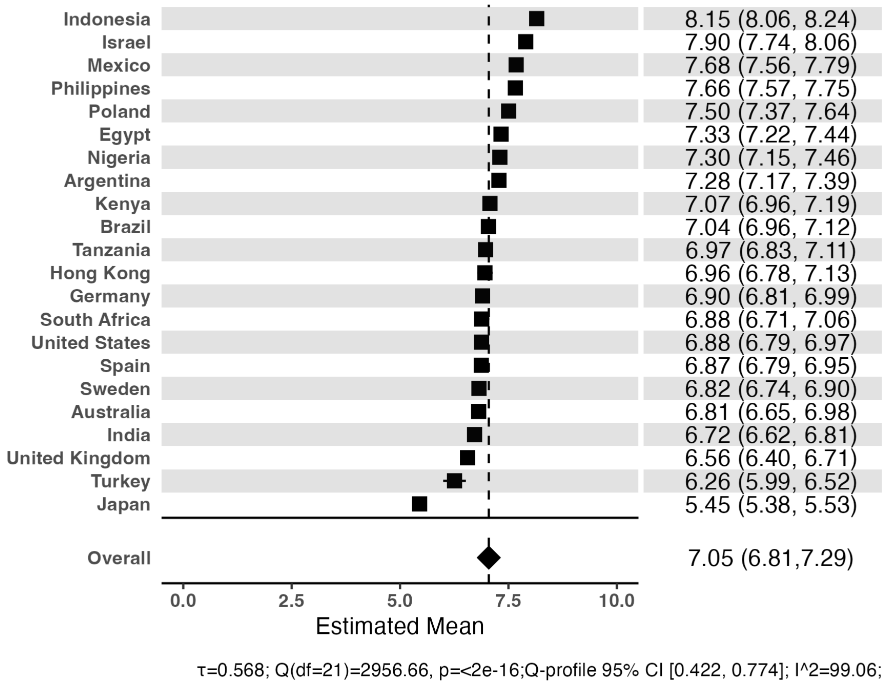

Figure S5. Heterogeneity in means scores across countries within group: Age group-50-59. (a) Flourishing with financial indicators (12 items) [left panel]; (b) Flourishing without financial indicators (10 items) [right panel]. N=202,898, subgroup means and standard errors are computed accounting for the complex sampling design using all data simultaneously. Analyses conducted: Random-effects meta-analysis of country-specific means. Squares represent the the point estimate (mean) for each country. The lines represented the +/-1.96*SE, standard error, around the mean; the overall pooled mean is represented by the diamond. The reported p-value for Q-statistics is necessarily 1-sided because of the use of the chi-squared distribution to test whether heterogeneity is greater than zero (i.e., a two-sided test is not applicable). No adjustments for multiple testing were made.

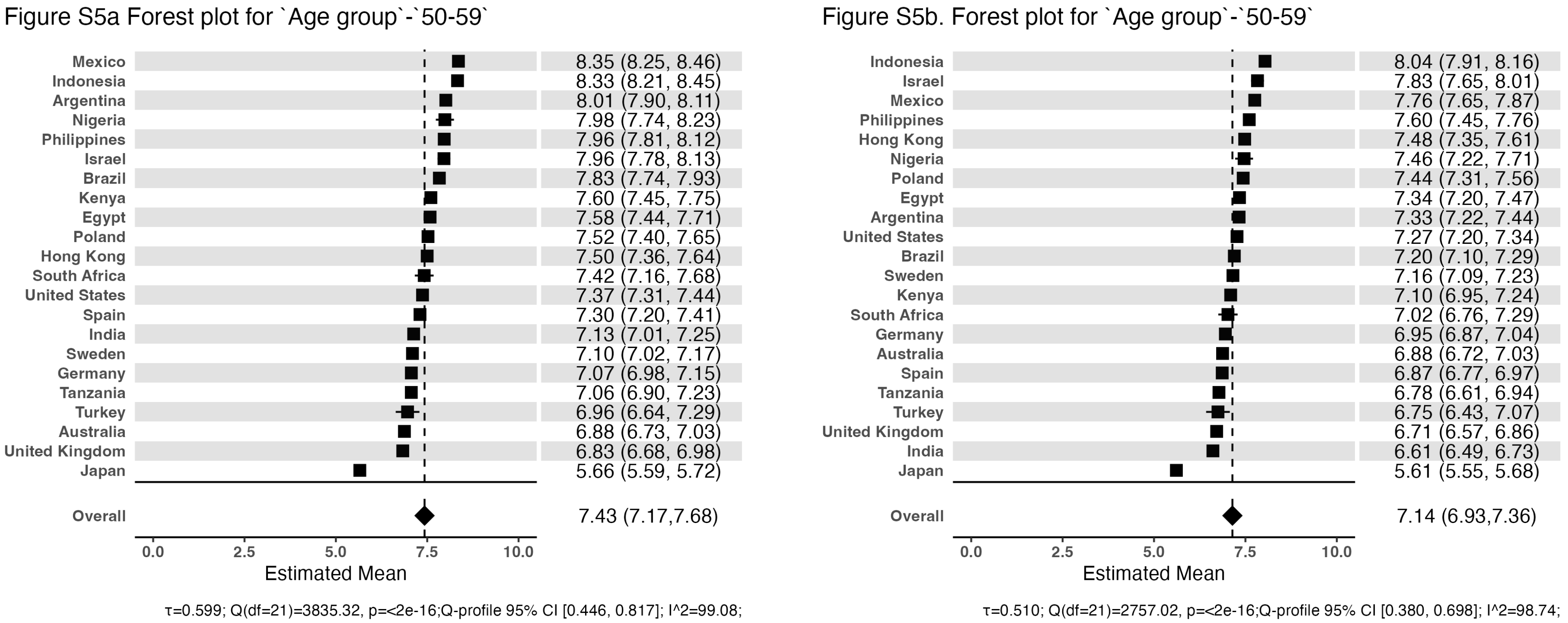

Figure S6. Heterogeneity in means scores across countries within group: Age group-60-69. (a) Flourishing with financial indicators (12 items) [left panel]; (b) Flourishing without financial indicators (10 items) [right panel]. N=202,898, subgroup means and standard errors are computed accounting for the complex sampling design using all data simultaneously. Analyses conducted: Random-effects meta-analysis of country-specific means. Squares represent the the point estimate (mean) for each country. The lines represented the +/-1.96*SE, standard error, around the mean; the overall pooled mean is represented by the diamond. The reported p-value for Q-statistics is necessarily 1-sided because of the use of the chi-squared distribution to test whether heterogeneity is greater than zero (i.e., a two-sided test is not applicable). No adjustments for multiple testing were made.

Figure S6a Forest plot for `Age group`-`60-69`

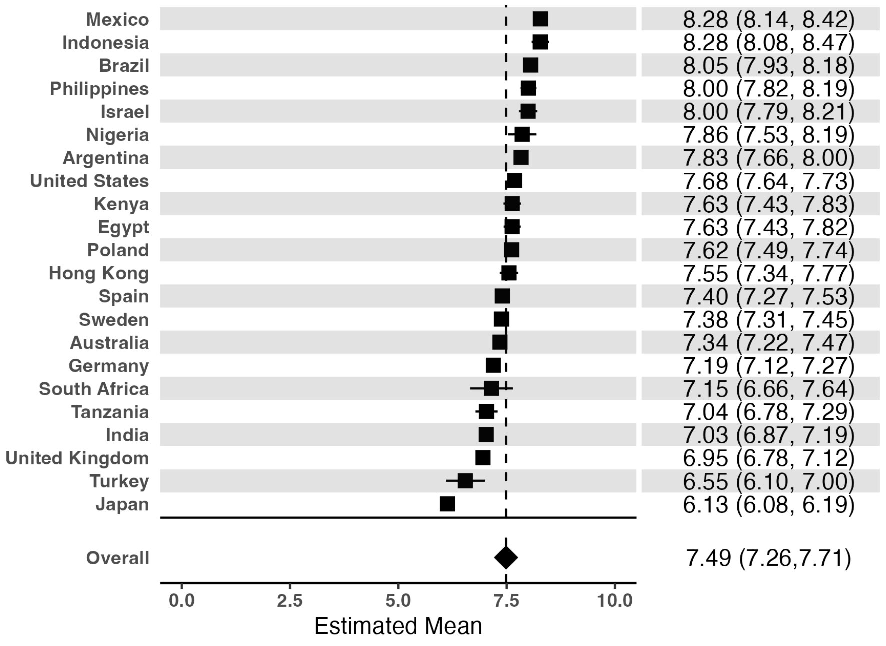

τ=0.527; Q(df=21)=2512.64, p=<2e-16;Q-profile 95% CI [0.387, 0.722]; I^2=98.72;

Figure S6b. Forest plot for `Age group`-`60-69`

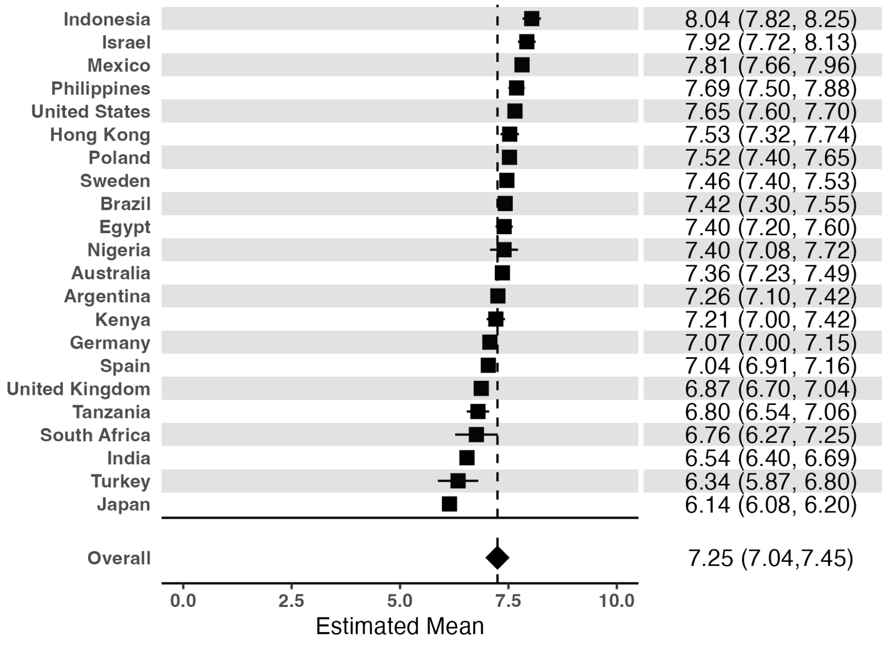

τ=0.485; Q(df=21)=2099.57, p=<2e-16;Q-profile 95% CI [0.352, 0.664]; I^2=98.49;

Figure S7. Heterogeneity in means scores across countries within group: Age group-70-79. (a) Flourishing with financial indicators (12 items) [left panel]; (b) Flourishing without financial indicators (10 items) [right panel]. N=202,898, subgroup means and standard errors are computed accounting for the complex sampling design using all data simultaneously. Analyses conducted: Random-effects meta-analysis of country-specific means. Squares represent the the point estimate (mean) for each country. The lines represented the +/-1.96*SE, standard error, around the mean; the overall pooled mean is represented by the diamond. The reported p-value for Q-statistics is necessarily 1-sided because of the use of the chi-squared distribution to test whether heterogeneity is greater than zero (i.e., a two-sided test is not applicable). No adjustments for multiple testing were made.

Figure S7a Forest plot for `Age group`-`70-79`

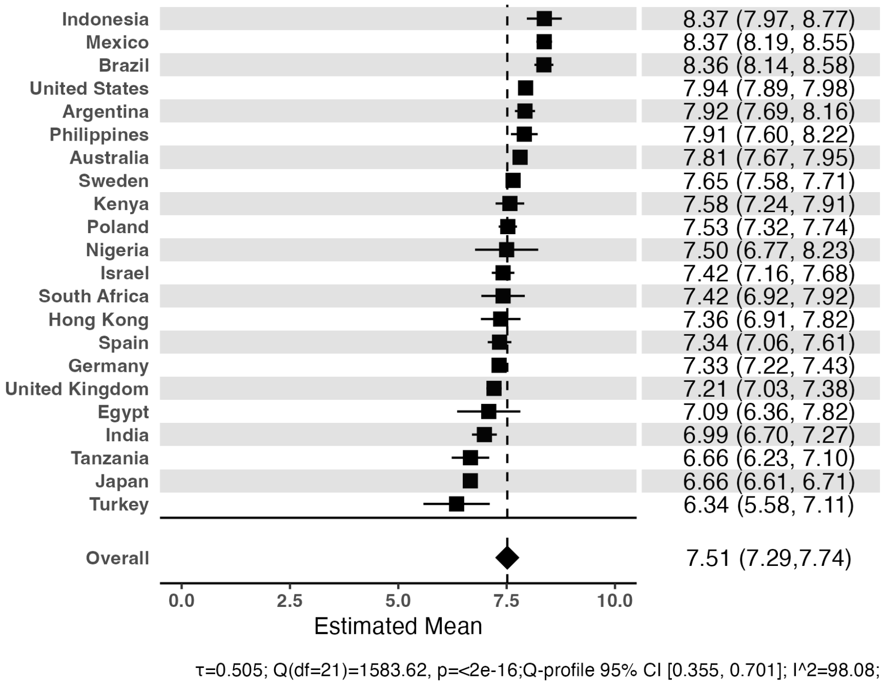

Figure S7b. Forest plot for `Age group`-`70-79`

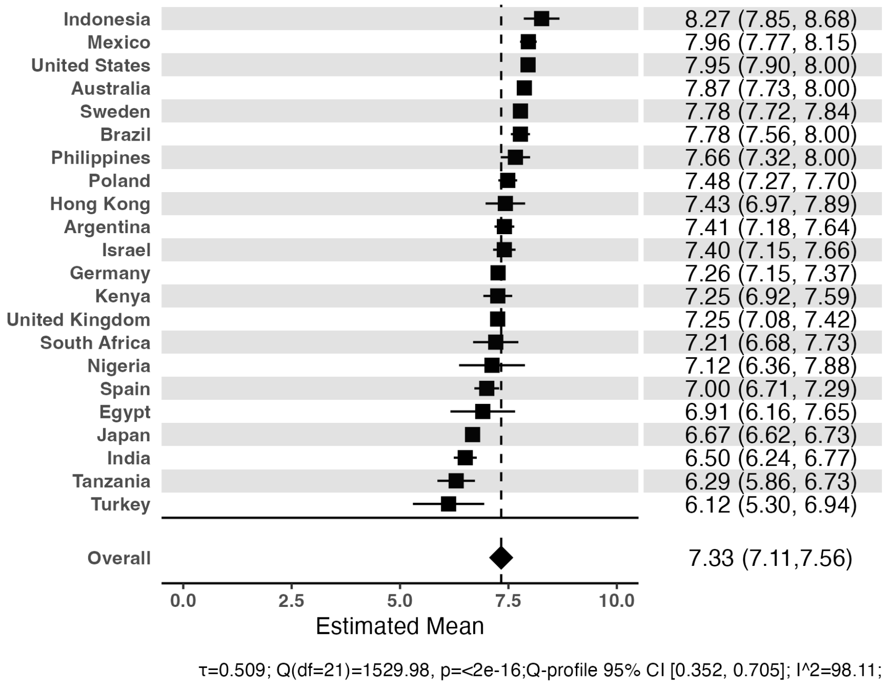

Figure S8. Heterogeneity in means scores across countries within group: Age group-80 or older. (a) Flourishing with financial indicators (12 items) [left panel]; (b) Flourishing without financial indicators (10 items) [right panel]. N=202,898, subgroup means and standard errors are computed accounting for the complex sampling design using all data simultaneously. Analyses conducted: Random-effects meta-analysis of country-specific means. Squares represent the the point estimate (mean) for each country. The lines represented the +/-1.96*SE, standard error, around the mean; the overall pooled mean is represented by the diamond. The reported p-value for Q-statistics is necessarily 1-sided because of the use of the chi-squared distribution to test whether heterogeneity is greater than zero (i.e., a two-sided test is not applicable). No adjustments for multiple testing were made.

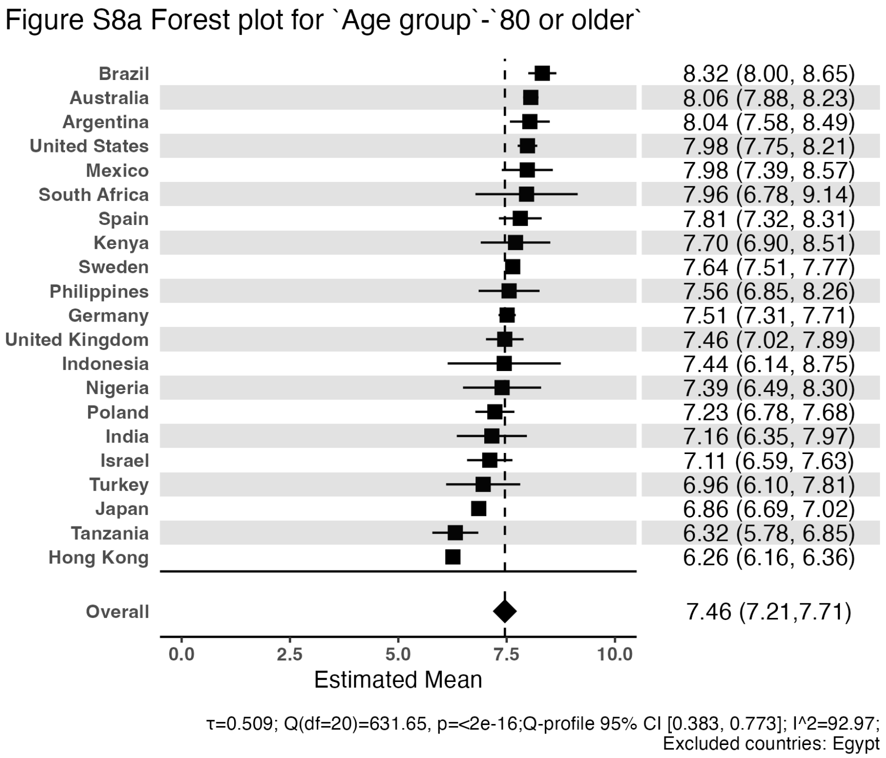

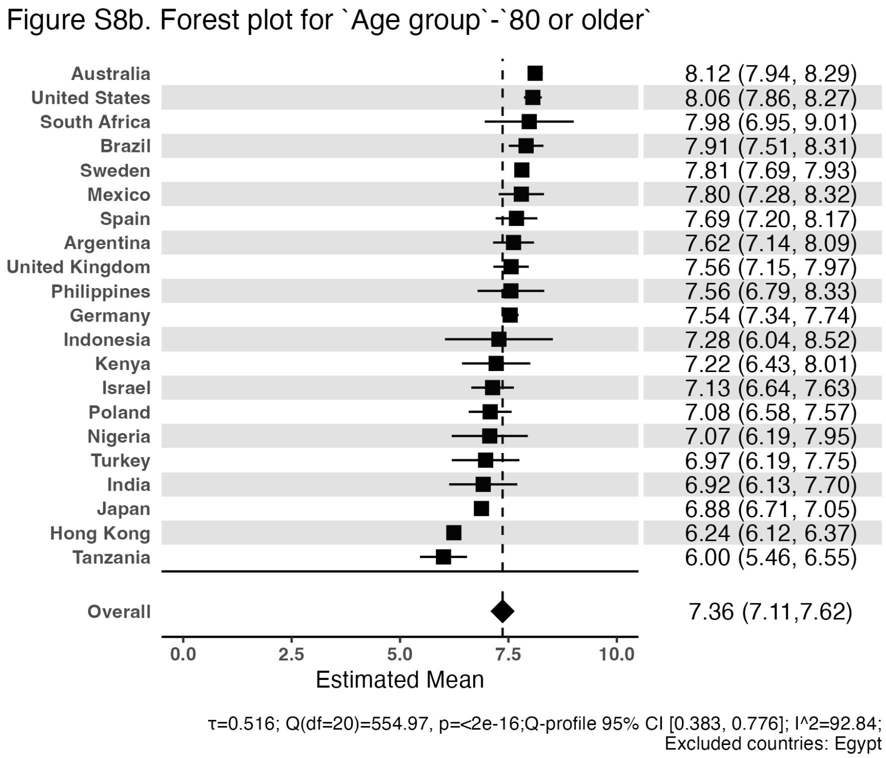

Figure S9. Heterogeneity in means scores across countries within group: Gender-Female. (a) Flourishing with financial indicators (12 items) [left panel]; (b) Flourishing without financial indicators (10 items) [right panel]. N=202,898, subgroup means and standard errors are computed accounting for the complex sampling design using all data simultaneously. Analyses conducted: Random-effects meta-analysis of country-specific means. Squares represent the the point estimate (mean) for each country. The lines represented the +/-1.96*SE, standard error, around the mean; the overall pooled mean is represented by the diamond. The reported p-value for Q-statistics is necessarily 1-sided because of the use of the chi-squared distribution to test whether heterogeneity is greater than zero (i.e., a two-sided test is not applicable). No adjustments for multiple testing were made.

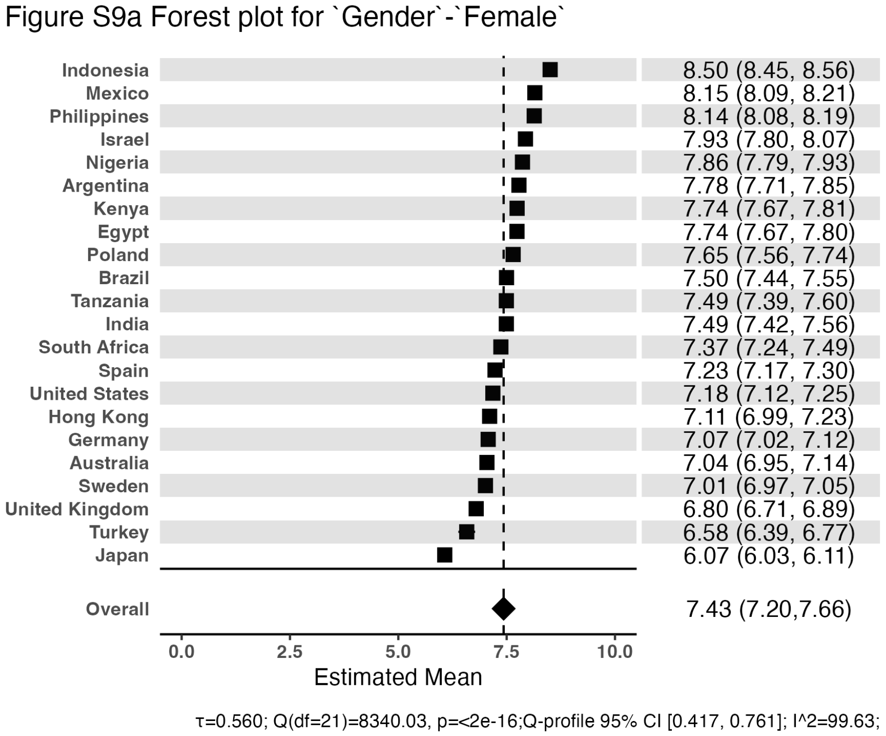

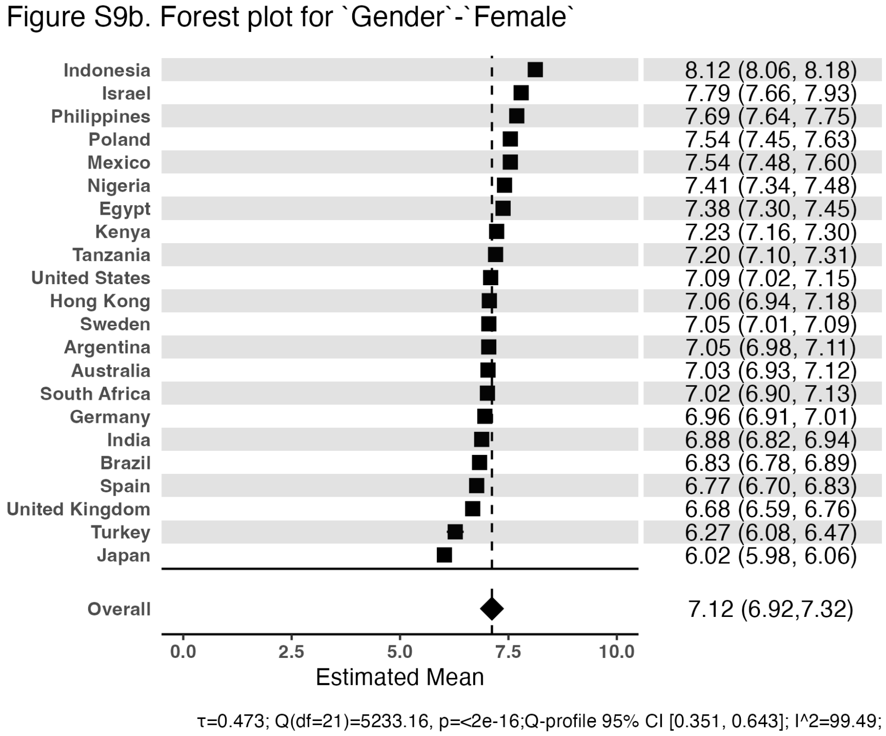

Figure S10. Heterogeneity in means scores across countries within group: Gender-Male. (a) Flourishing with financial indicators (12 items) [left panel]; (b) Flourishing without financial indicators (10 items) [right panel]. N=202,898, subgroup means and standard errors are computed accounting for the complex sampling design using all data simultaneously. Analyses conducted: Random-effects meta-analysis of country-specific means. Squares represent the the point estimate (mean) for each country. The lines represented the +/-1.96*SE, standard error, around the mean; the overall pooled mean is represented by the diamond. The reported p-value for Q-statistics is necessarily 1-sided because of the use of the chi-squared distribution to test whether heterogeneity is greater than zero (i.e., a two-sided test is not applicable). No adjustments for multiple testing were made.

Figure S10a Forest plot for `Gender`-`Male`

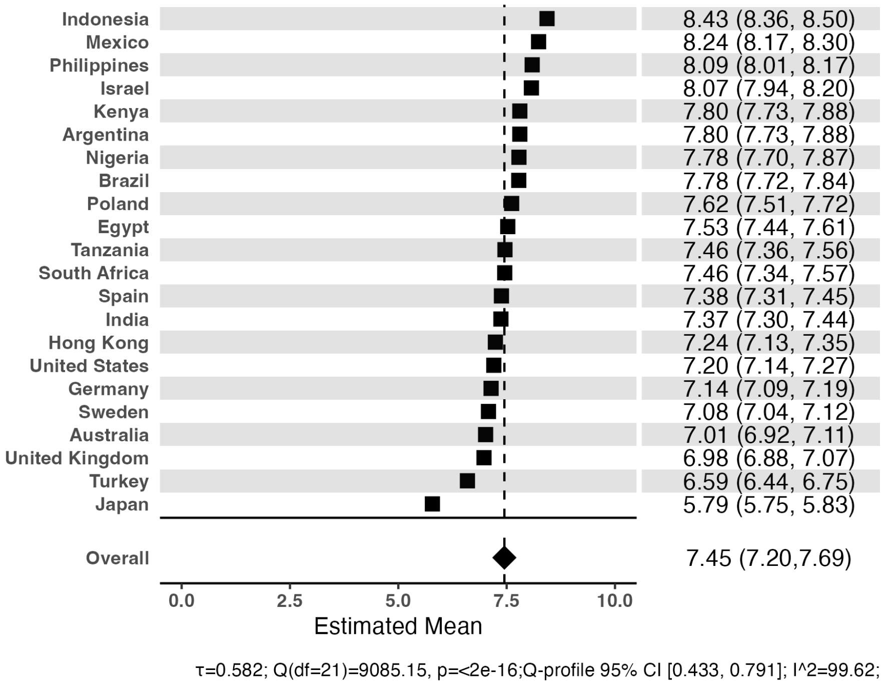

Figure S10b. Forest plot for `Gender`-`Male`

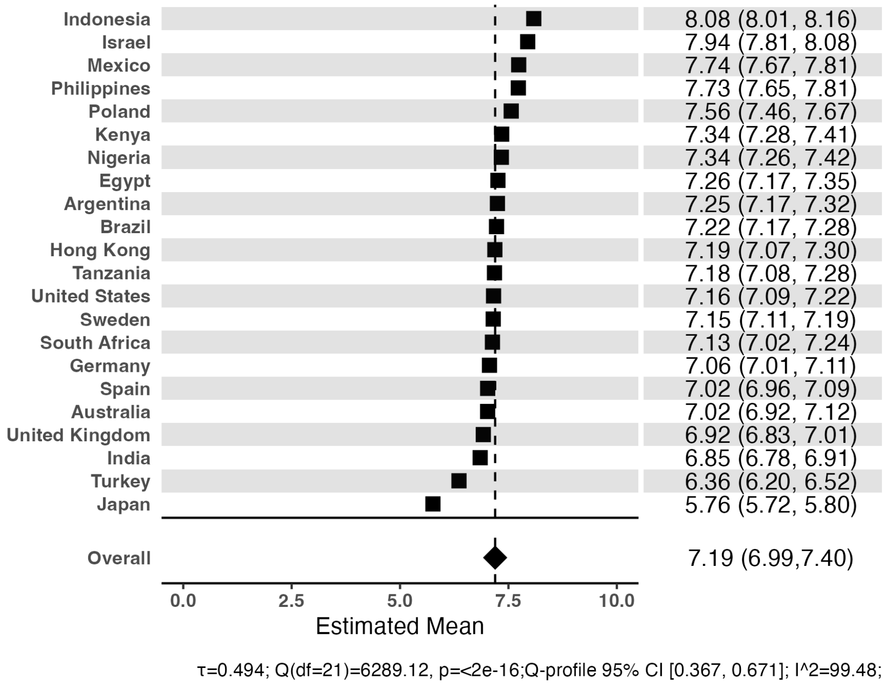

Figure S11. Heterogeneity in means scores across countries within group: Gender-Other. (a) Flourishing with financial indicators (12 items) [left panel]; (b) Flourishing without financial indicators (10 items) [right panel]. N=202,898, subgroup means and standard errors are computed accounting for the complex sampling design using all data simultaneously. Analyses conducted: Random-effects meta-analysis of country-specific means. Squares represent the the point estimate (mean) for each country. The lines represented the +/-1.96*SE, standard error, around the mean; the overall pooled mean is represented by the diamond. The reported p-value for Q-statistics is necessarily 1-sided because of the use of the chi-squared distribution to test whether heterogeneity is greater than zero (i.e., a two-sided test is not applicable). No adjustments for multiple testing were made.

Figure S11a Forest plot for `Gender`-`Other`

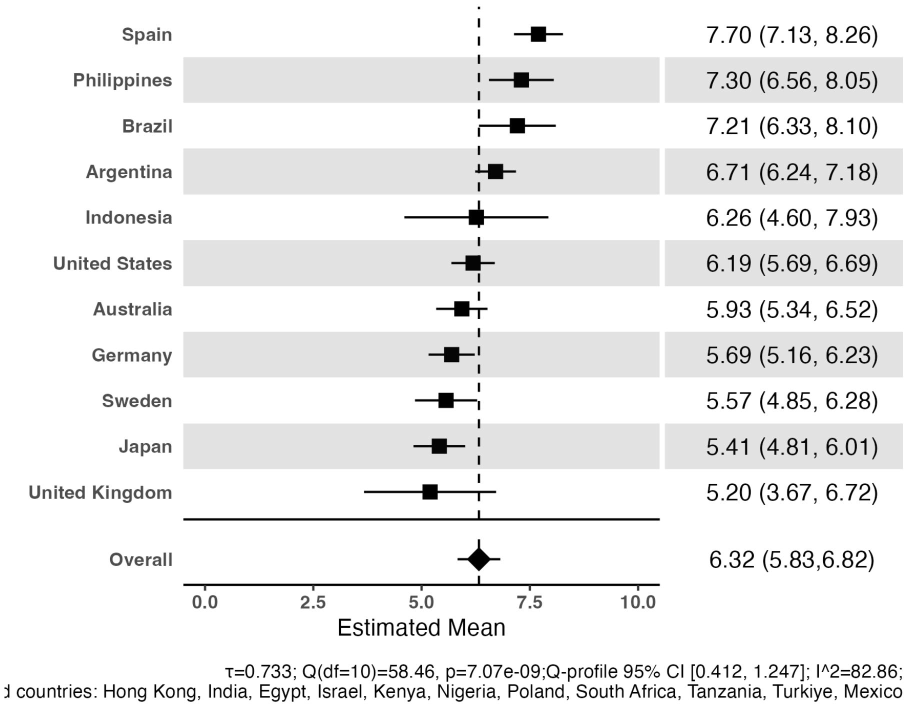

Figure S11b. Forest plot for `Gender`-`Other`

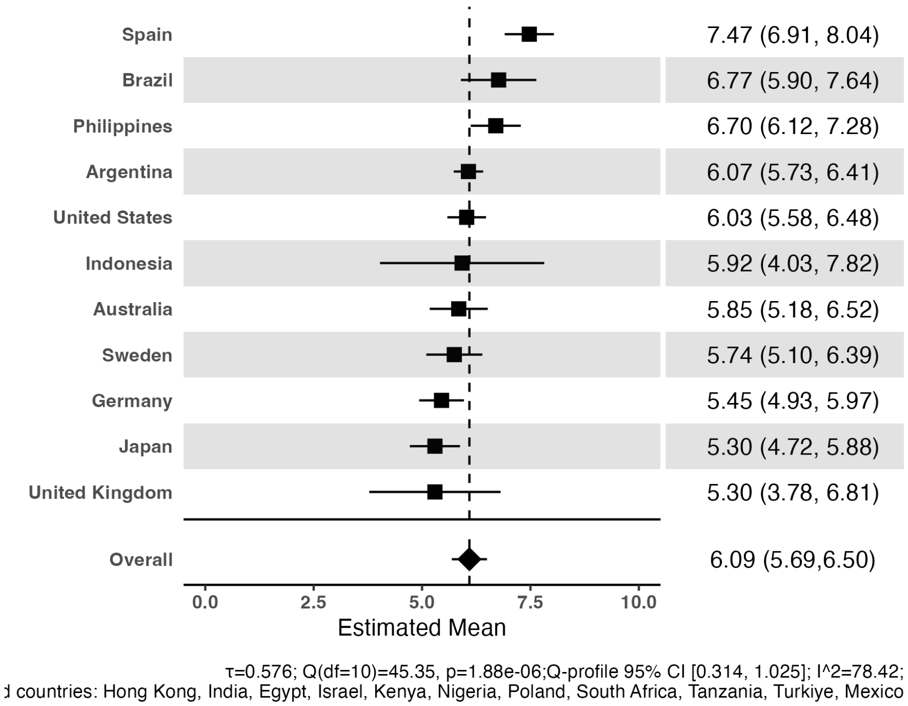

Figure S12. Heterogeneity in means scores across countries within group: Marital status-Divorced. (a) Flourishing with financial indicators (12 items) [left panel]; (b) Flourishing without financial indicators (10 items) [right panel]. N=202,898, subgroup means and standard errors are computed accounting for the complex sampling design using all data simultaneously. Analyses conducted: Random-effects meta-analysis of country-specific means. Squares represent the the point estimate (mean) for each country. The lines represented the +/-1.96*SE, standard error, around the mean; the overall pooled mean is represented by the diamond. The reported p-value for Q-statistics is necessarily 1-sided because of the use of the chi-squared distribution to test whether heterogeneity is greater than zero (i.e., a two-sided test is not applicable). No adjustments for multiple testing were made.

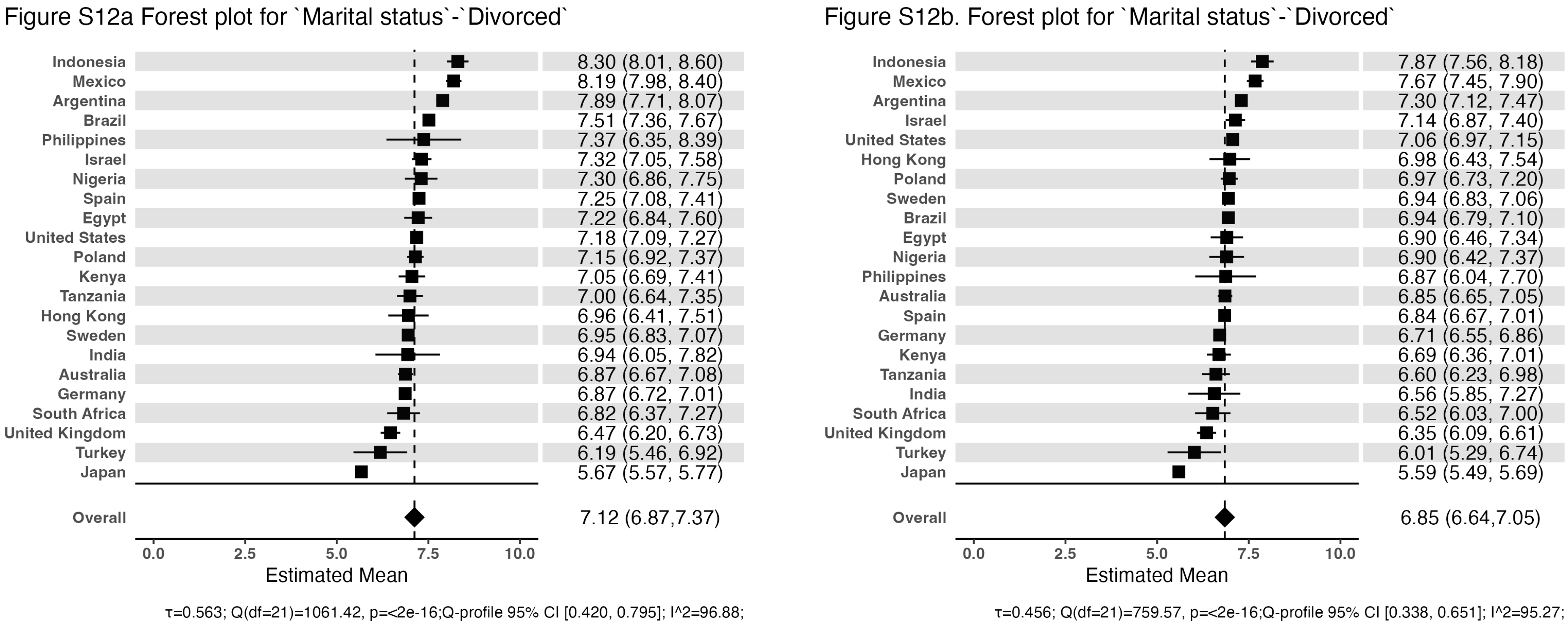

Figure S13. Heterogeneity in means scores across countries within group: Marital status-Domestic partner. (a) Flourishing with financial indicators (12 items) [left panel]; (b) Flourishing without financial indicators (10 items) [right panel]. N=202,898, subgroup means and standard errors are computed accounting for the complex sampling design using all data simultaneously. Analyses conducted: Random-effects meta-analysis of country-specific means. Squares represent the the point estimate (mean) for each country. The lines represented the +/-1.96*SE, standard error, around the mean; the overall pooled mean is represented by the diamond. The reported p-value for Q-statistics is necessarily 1-sided because of the use of the chi-squared distribution to test whether heterogeneity is greater than zero (i.e., a two-sided test is not applicable). No adjustments for multiple testing were made.

Figure S13a Forest plot for `Marital status`-`Domestic partner`

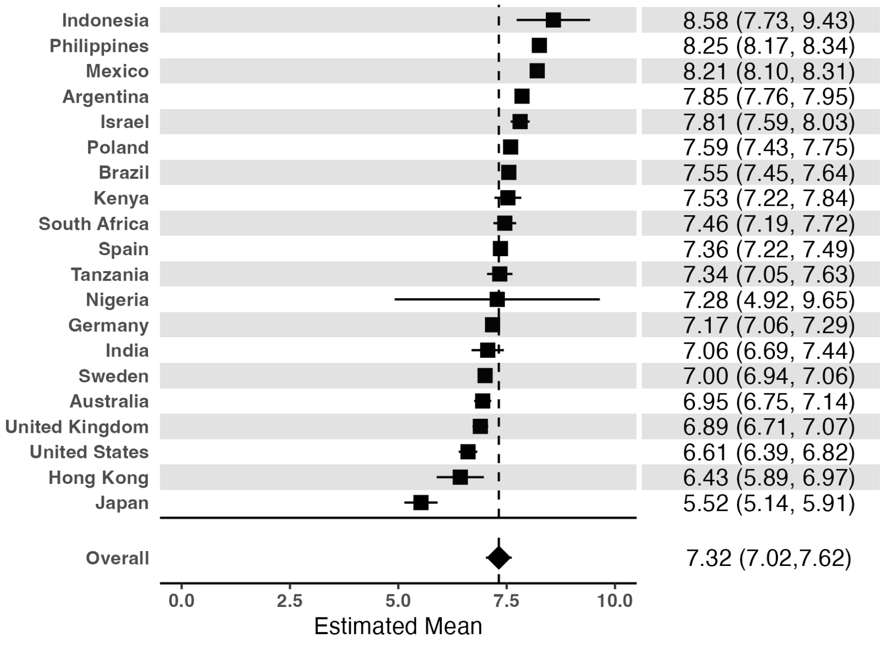

τ=0.652; Q(df=19)=1107.34, p=<2e-16;Q-profile 95% CI [0.458, 0.921]; I^2=98.75;
Excluded countries: Egypt, Turkiye

Figure S13b. Forest plot for `Marital status`-`Domestic partner`

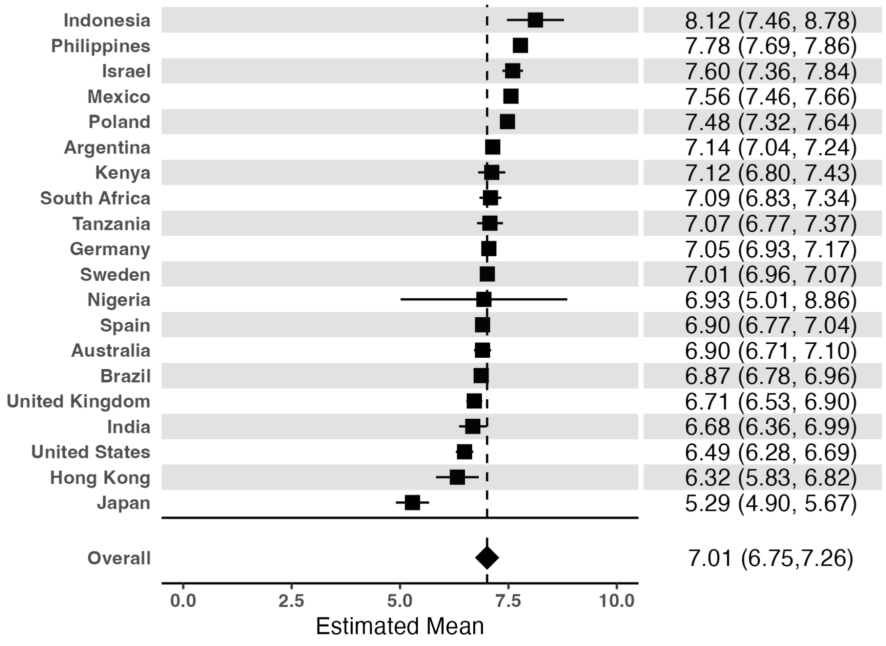

τ=0.557; Q(df=19)=553.66, p=<2e-16;Q-profile 95% CI [0.382, 0.785]; I^2=98.34;
Excluded countries: Egypt, Turkiye

Figure S14. Heterogeneity in means scores across countries within group: Marital status-Married. (a) Flourishing with financial indicators (12 items) [left panel]; (b) Flourishing without financial indicators (10 items) [right panel]. N=202,898, subgroup means and standard errors are computed accounting for the complex sampling design using all data simultaneously. Analyses conducted: Random-effects meta-analysis of country-specific means. Squares represent the the point estimate (mean) for each country. The lines represented the +/-1.96*SE, standard error, around the mean; the overall pooled mean is represented by the diamond. The reported p-value for Q-statistics is necessarily 1-sided because of the use of the chi-squared distribution to test whether heterogeneity is greater than zero (i.e., a two-sided test is not applicable). No adjustments for multiple testing were made.

Figure S14a Forest plot for `Marital status`-`Married`

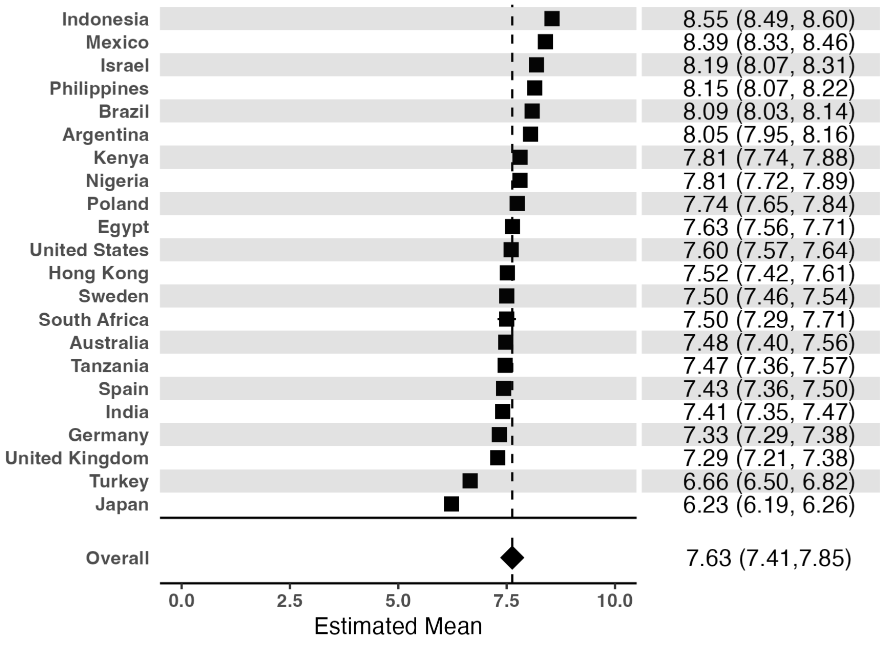

τ=0.522; Q(df=21)=8466.50, p=<2e-16;Q-profile 95% CI [0.389, 0.710]; I^2=99.60;

Figure S14b. Forest plot for `Marital status`-`Married`

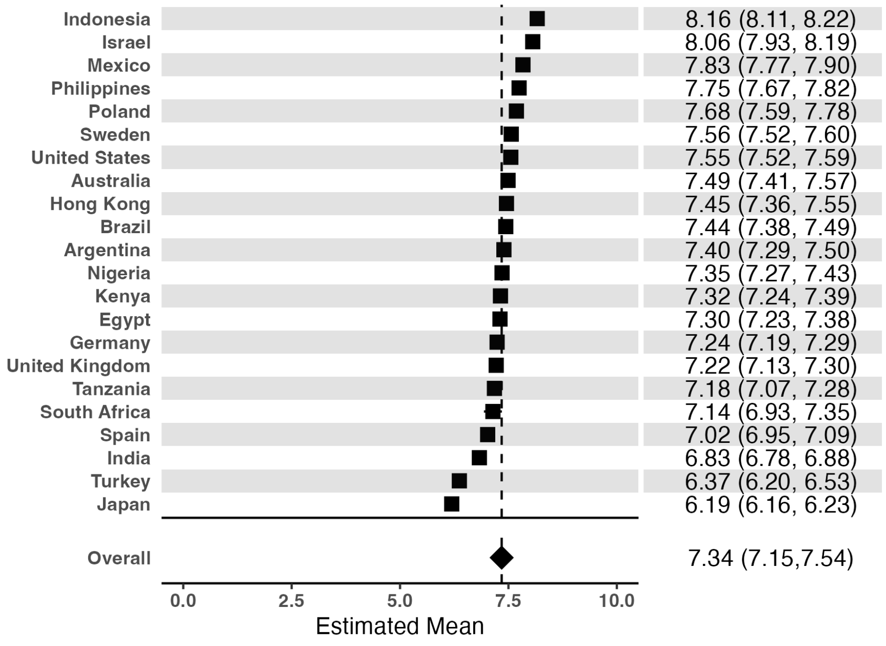

τ=0.465; Q(df=21)=6198.50, p=<2e-16;Q-profile 95% CI [0.345, 0.632]; I^2=99.51;

Figure S15. Heterogeneity in means scores across countries within group: Marital status-Separated. (a) Flourishing with financial indicators (12 items) [left panel]; (b) Flourishing without financial indicators (10 items) [right panel]. N=202,898, subgroup means and standard errors are computed accounting for the complex sampling design using all data simultaneously. Analyses conducted: Random-effects meta-analysis of country-specific means. Squares represent the the point estimate (mean) for each country. The lines represented the +/-1.96*SE, standard error, around the mean; the overall pooled mean is represented by the diamond. The reported p-value for Q-statistics is necessarily 1-sided because of the use of the chi-squared distribution to test whether heterogeneity is greater than zero (i.e., a two-sided test is not applicable). No adjustments for multiple testing were made.

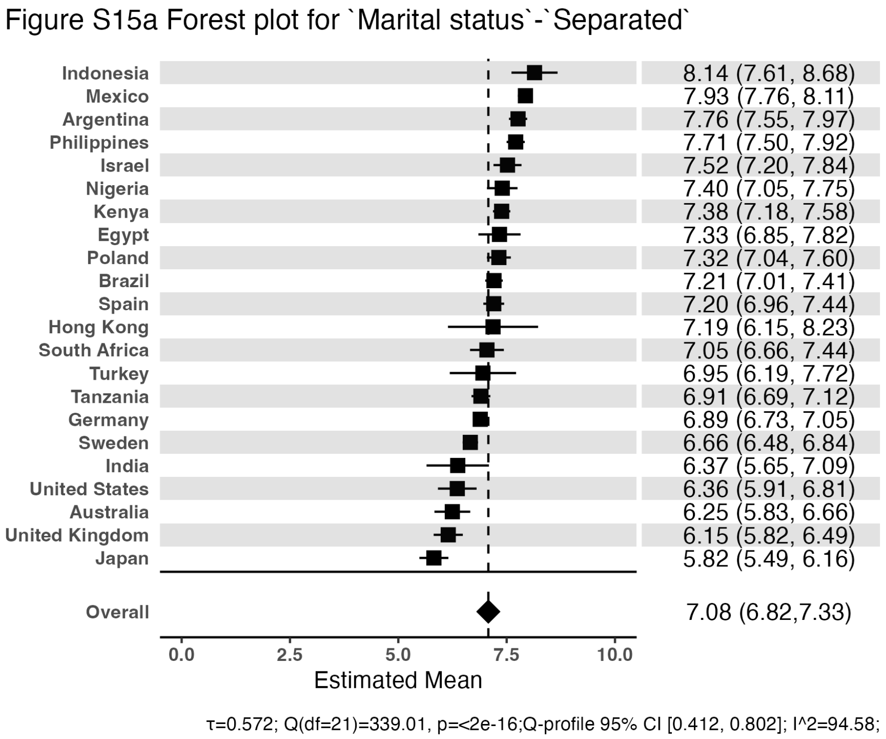

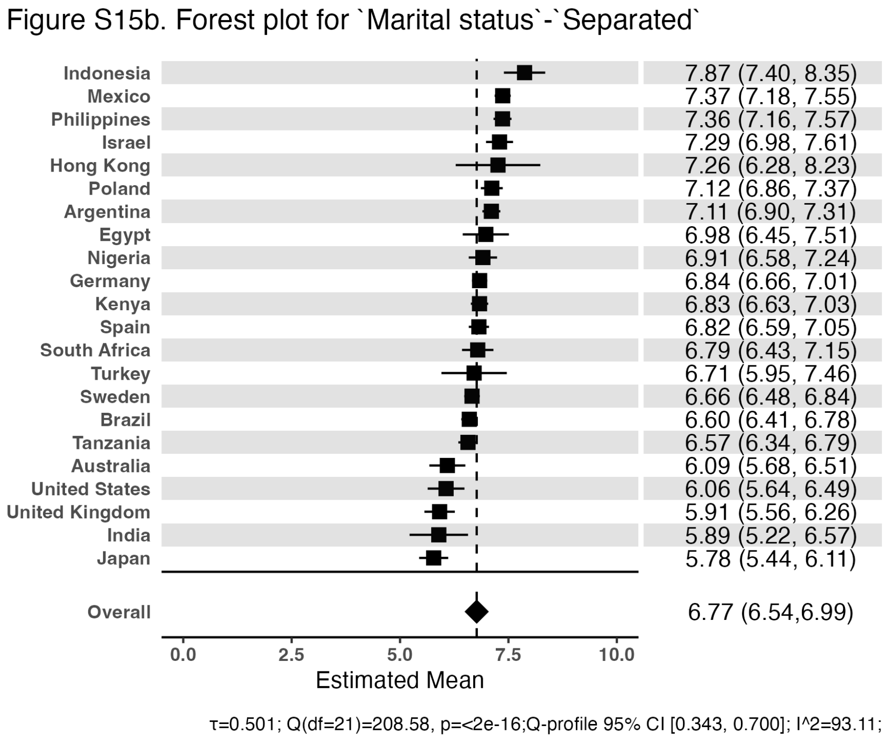

Figure S16. Heterogeneity in means scores across countries within group: Marital status-Single, never married. (a) Flourishing with financial indicators (12 items) [left panel]; (b) Flourishing without financial indicators (10 items) [right panel]. N=202,898, subgroup means and standard errors are computed accounting for the complex sampling design using all data simultaneously. Analyses conducted: Random-effects meta-analysis of country-specific means. Squares represent the the point estimate (mean) for each country. The lines represented the +/-1.96*SE, standard error, around the mean; the overall pooled mean is represented by the diamond. The reported p-value for Q-statistics is necessarily 1-sided because of the use of the chi-squared distribution to test whether heterogeneity is greater than zero (i.e., a two-sided test is not applicable). No adjustments for multiple testing were made.

Figure S16a Forest plot for `Marital status`-`Single, never married`

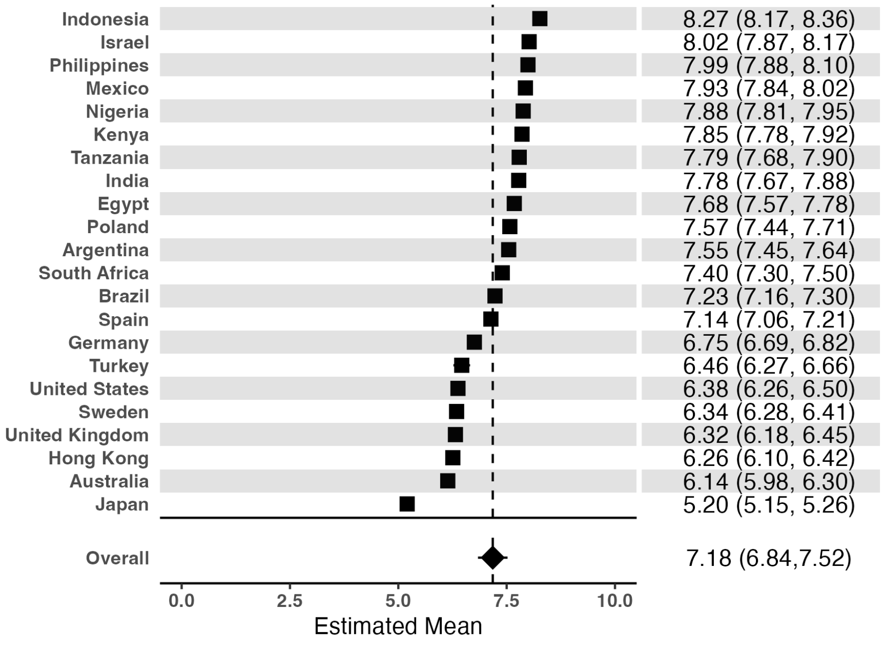

τ=0.814; Q(df=21)=8580.56, p=<2e-16;Q-profile 95% CI [0.606, 1.106]; I^2=99.67;

Figure S16b. Forest plot for `Marital status`-`Single, never married`

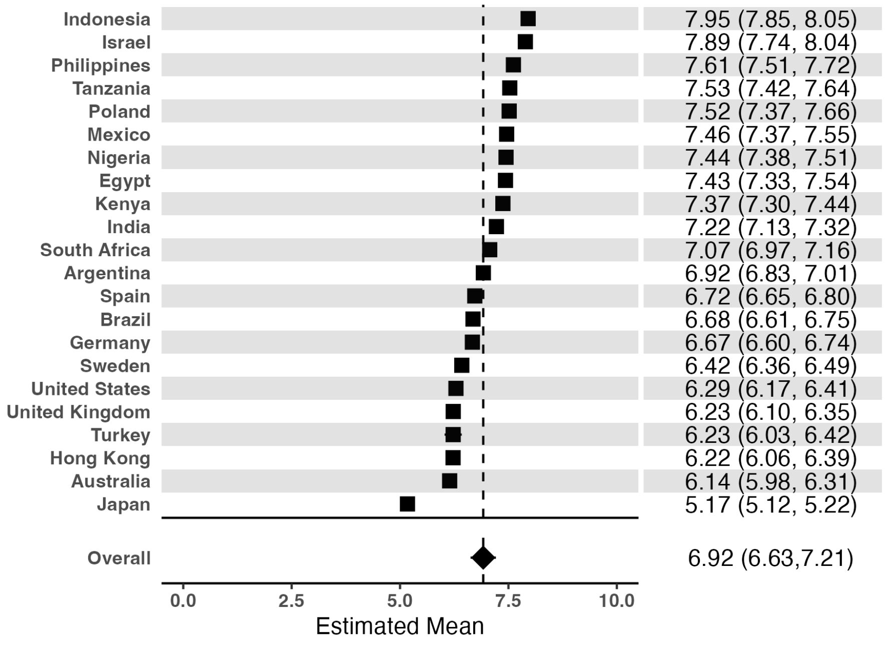

τ=0.697; Q(df=21)=6346.66, p=<2e-16;Q-profile 95% CI [0.519, 0.948]; I^2=99.57;

Figure S17. Heterogeneity in means scores across countries within group: Marital status-Widowed. (a) Flourishing with financial indicators (12 items) [left panel]; (b) Flourishing without financial indicators (10 items) [right panel]. N=202,898, subgroup means and standard errors are computed accounting for the complex sampling design using all data simultaneously. Analyses conducted: Random-effects meta-analysis of country-specific means. Squares represent the the point estimate (mean) for each country. The lines represented the +/-1.96*SE, standard error, around the mean; the overall pooled mean is represented by the diamond. The reported p-value for Q-statistics is necessarily 1-sided because of the use of the chi-squared distribution to test whether heterogeneity is greater than zero (i.e., a two-sided test is not applicable). No adjustments for multiple testing were made.

Figure S17a Forest plot for `Marital status`-`Widowed`

Figure S17b. Forest plot for `Marital status`-`Widowed`

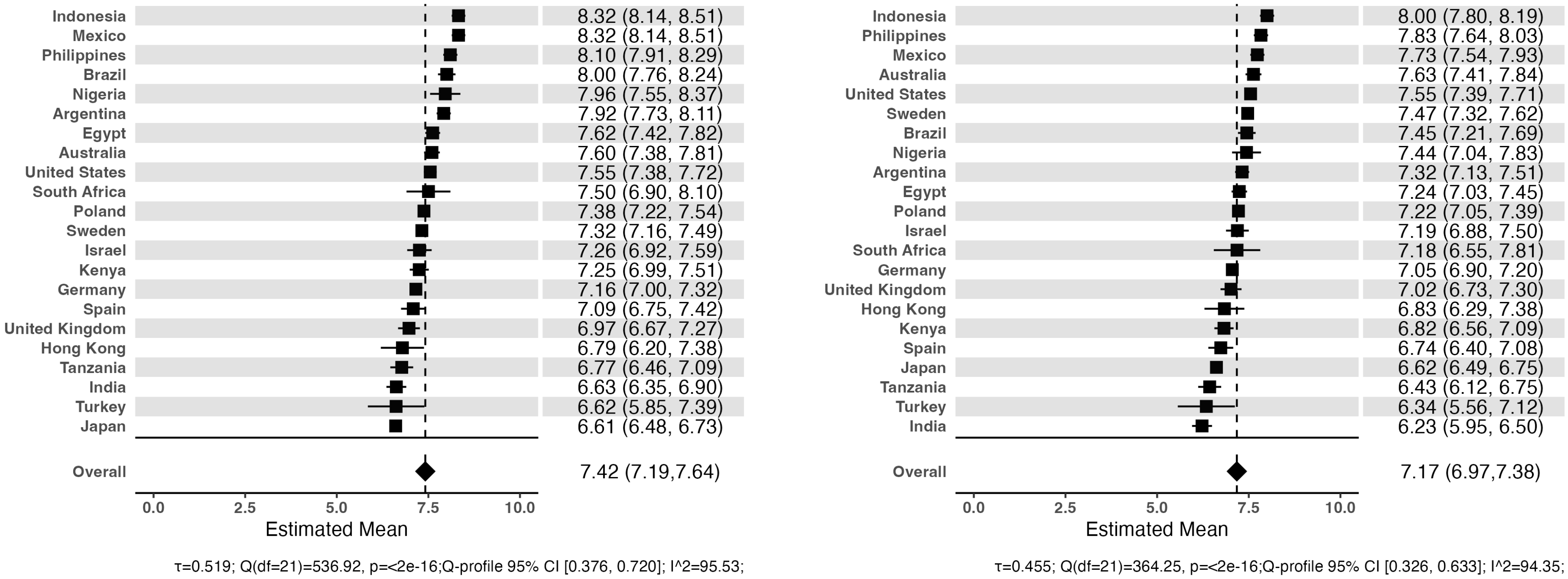

Figure S18. Heterogeneity in means scores across countries within group: Employment status-Employed for an employer. (a) Flourishing with financial indicators (12 items) [left panel]; (b) Flourishing without financial indicators (10 items) [right panel]. N=202,898, subgroup means and standard errors are computed accounting for the complex sampling design using all data simultaneously. Analyses conducted: Random-effects meta-analysis of country-specific means. Squares represent the the point estimate (mean) for each country. The lines represented the +/-1.96*SE, standard error, around the mean; the overall pooled mean is represented by the diamond. The reported p-value for Q-statistics is necessarily 1-sided because of the use of the chi-squared distribution to test whether heterogeneity is greater than zero (i.e., a two-sided test is not applicable). No adjustments for multiple testing were made.

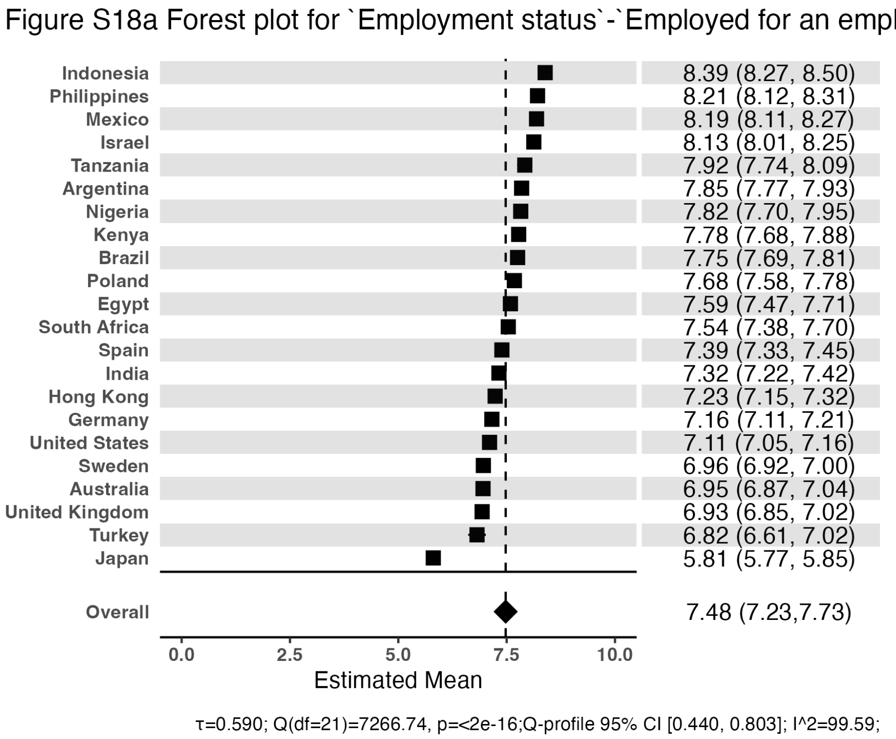

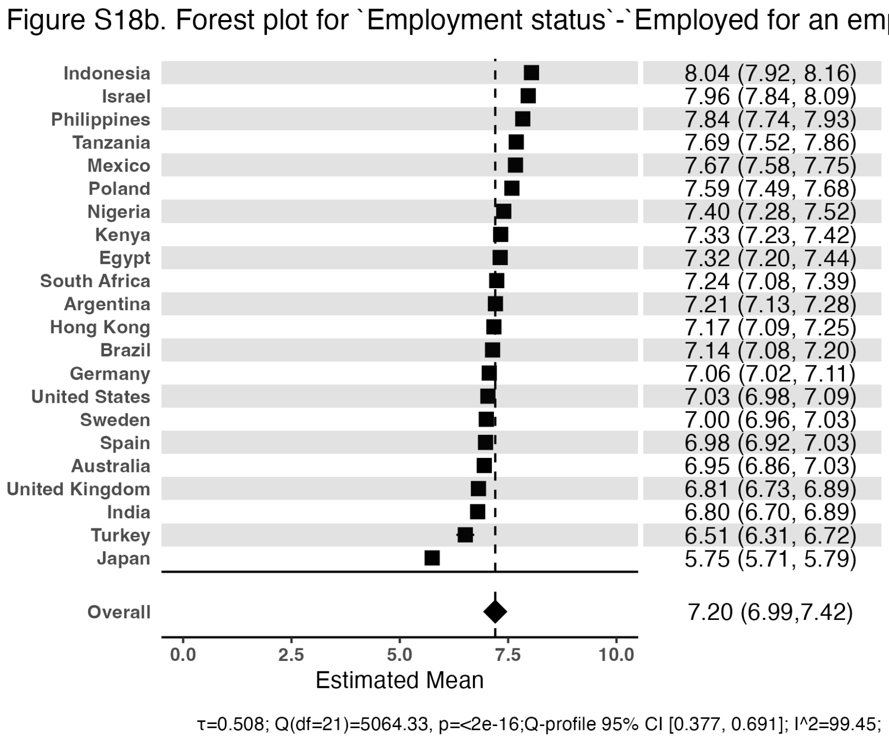

Figure S19. Heterogeneity in means scores across countries within group: Employment status-Homemaker. (a) Flourishing with financial indicators (12 items) [left panel]; (b) Flourishing without financial indicators (10 items) [right panel]. N=202,898, subgroup means and standard errors are computed accounting for the complex sampling design using all data simultaneously. Analyses conducted: Random-effects meta-analysis of country-specific means. Squares represent the the point estimate (mean) for each country. The lines represented the +/-1.96*SE, standard error, around the mean; the overall pooled mean is represented by the diamond. The reported p-value for Q-statistics is necessarily 1-sided because of the use of the chi-squared distribution to test whether heterogeneity is greater than zero (i.e., a two-sided test is not applicable). No adjustments for multiple testing were made.

Figure S19a Forest plot for `Employment status`-`Homemaker`

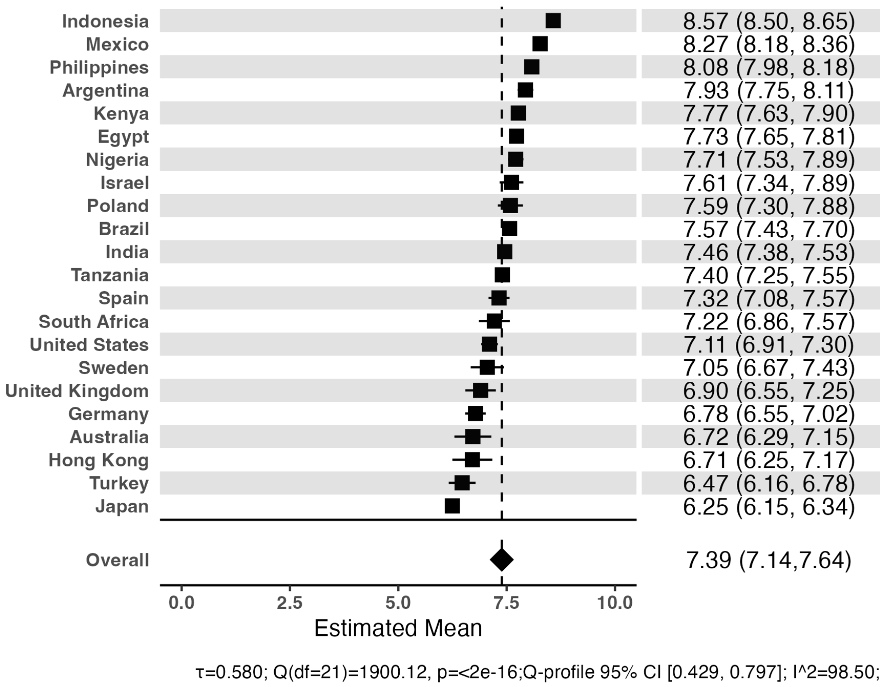

Figure S19b. Forest plot for `Employment status`-`Homemaker`

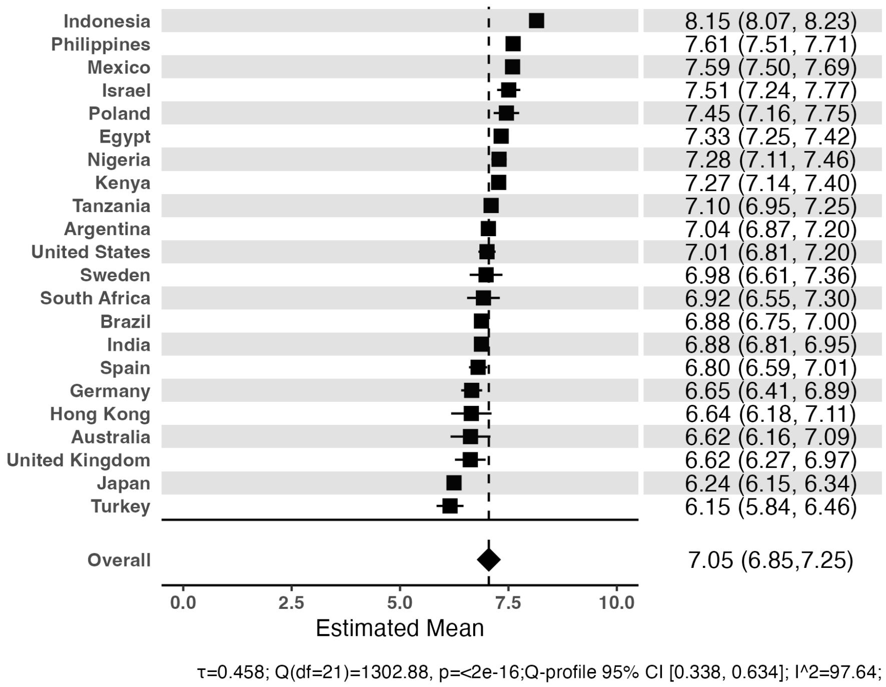

Figure S20. Heterogeneity in means scores across countries within group: Employment status-None of these/other. (a) Flourishing with financial indicators (12 items) [left panel]; (b) Flourishing without financial indicators (10 items) [right panel]. N=202,898, subgroup means and standard errors are computed accounting for the complex sampling design using all data simultaneously. Analyses conducted: Random-effects meta-analysis of country-specific means. Squares represent the the point estimate (mean) for each country. The lines represented the +/-1.96*SE, standard error, around the mean; the overall pooled mean is represented by the diamond. The reported p-value for Q-statistics is necessarily 1-sided because of the use of the chi-squared distribution to test whether heterogeneity is greater than zero (i.e., a two-sided test is not applicable). No adjustments for multiple testing were made.

Figure S20a Forest plot for `Employment status`-`None of these/other`

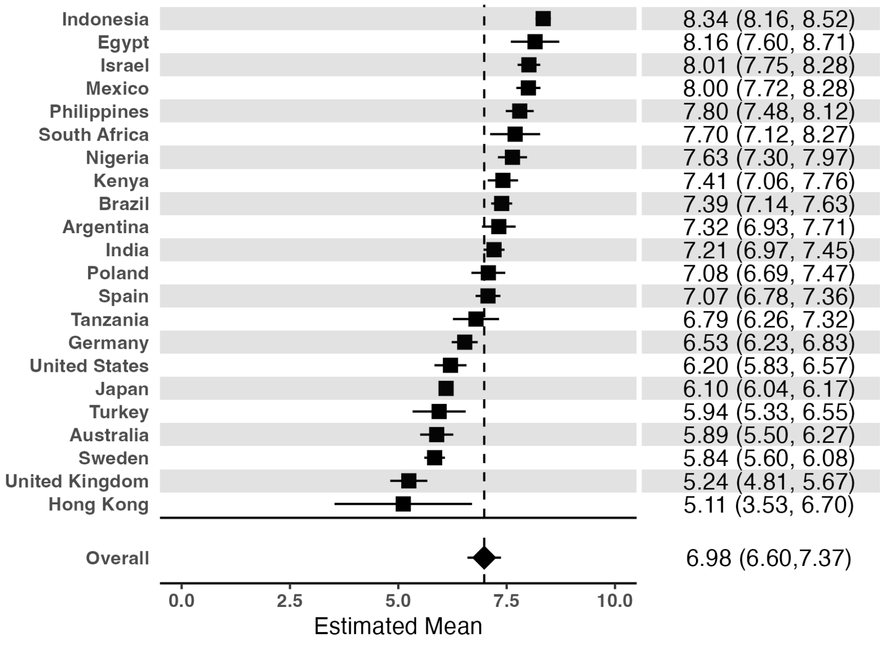

τ=0.898; Q(df=21)=1163.91, p=<2e-16;Q-profile 95% CI [0.638, 1.225]; I^2=97.87;

Figure S20b. Forest plot for `Employment status`-`None of these/other`

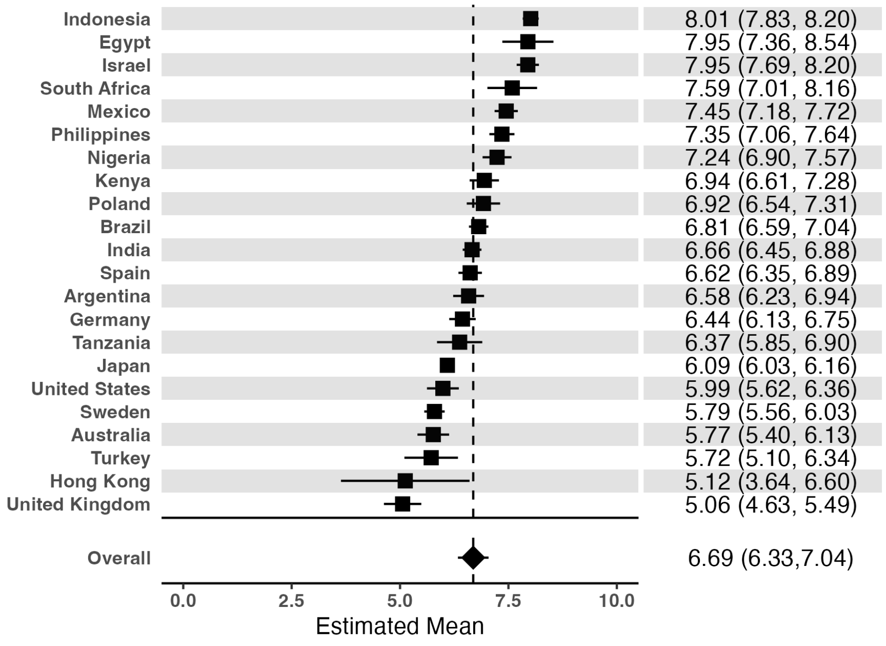

τ=0.816; Q(df=21)=805.39, p=<2e-16;Q-profile 95% CI [0.578, 1.116]; I^2=97.56;

Figure S21. Heterogeneity in means scores across countries within group: Employment status-Retired. (a) Flourishing with financial indicators (12 items) [left panel]; (b) Flourishing without financial indicators (10 items) [right panel]. N=202,898, subgroup means and standard errors are computed accounting for the complex sampling design using all data simultaneously. Analyses conducted: Random-effects meta-analysis of country-specific means. Squares represent the the point estimate (mean) for each country. The lines represented the +/-1.96*SE, standard error, around the mean; the overall pooled mean is represented by the diamond. The reported p-value for Q-statistics is necessarily 1-sided because of the use of the chi-squared distribution to test whether heterogeneity is greater than zero (i.e., a two-sided test is not applicable). No adjustments for multiple testing were made.

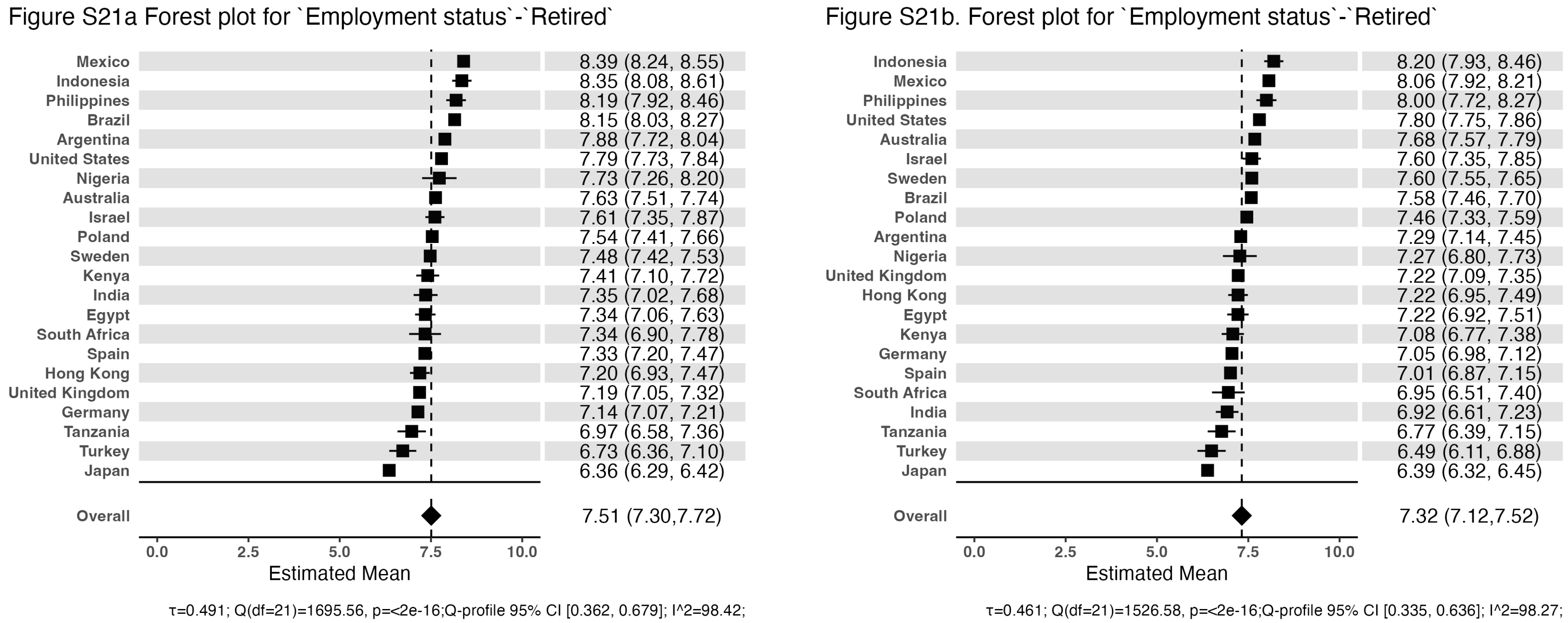

Figure S22. Heterogeneity in means scores across countries within group: Employment status-Self-employed. (a) Flourishing with financial indicators (12 items) [left panel]; (b) Flourishing without financial indicators (10 items) [right panel]. N=202,898, subgroup means and standard errors are computed accounting for the complex sampling design using all data simultaneously. Analyses conducted: Random-effects meta-analysis of country-specific means. Squares represent the the point estimate (mean) for each country. The lines represented the +/-1.96*SE, standard error, around the mean; the overall pooled mean is represented by the diamond. The reported p-value for Q-statistics is necessarily 1-sided because of the use of the chi-squared distribution to test whether heterogeneity is greater than zero (i.e., a two-sided test is not applicable). No adjustments for multiple testing were made.

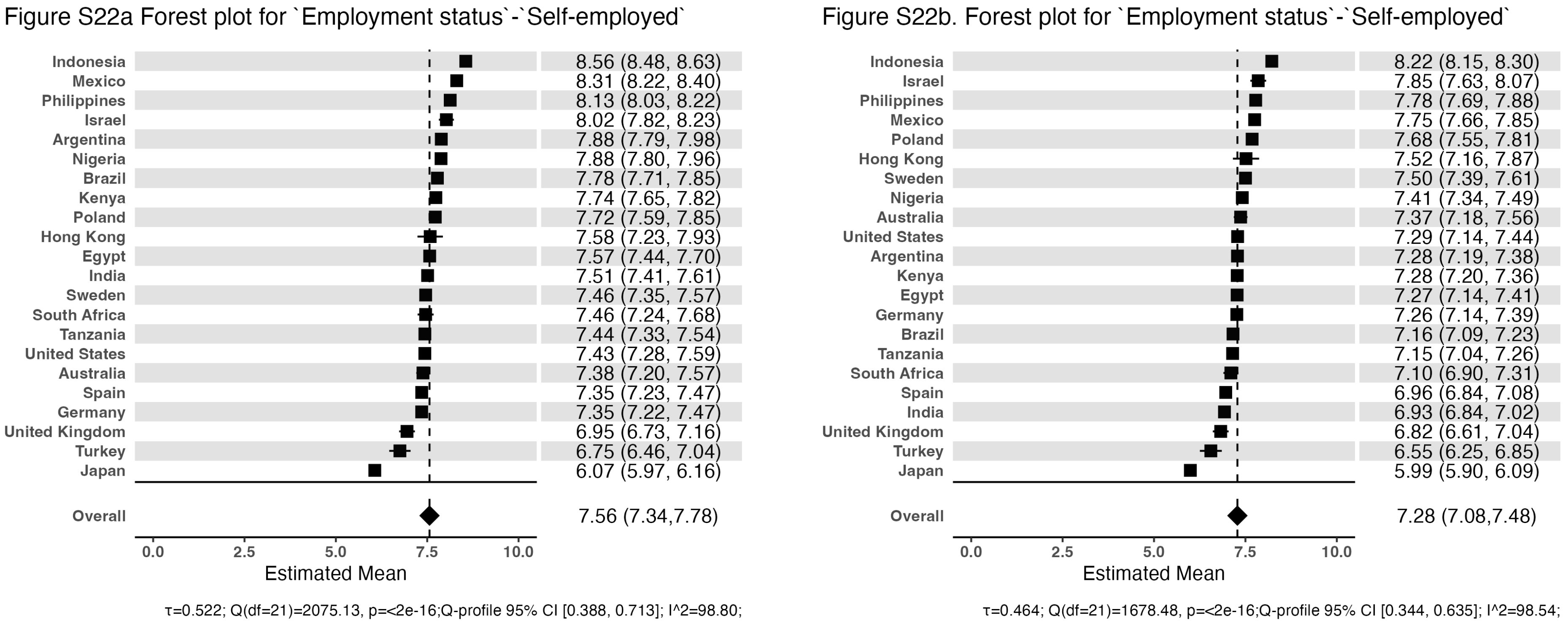

Figure S23. Heterogeneity in means scores across countries within group: Employment status-Student. (a) Flourishing with financial indicators (12 items) [left panel]; (b) Flourishing without financial indicators (10 items) [right panel]. N=202,898, subgroup means and standard errors are computed accounting for the complex sampling design using all data simultaneously. Analyses conducted: Random-effects meta-analysis of country-specific means. Squares represent the the point estimate (mean) for each country. The lines represented the +/-1.96*SE, standard error, around the mean; the overall pooled mean is represented by the diamond. The reported p-value for Q-statistics is necessarily 1-sided because of the use of the chi-squared distribution to test whether heterogeneity is greater than zero (i.e., a two-sided test is not applicable). No adjustments for multiple testing were made.

Figure S23a Forest plot for `Employment status`-`Student`

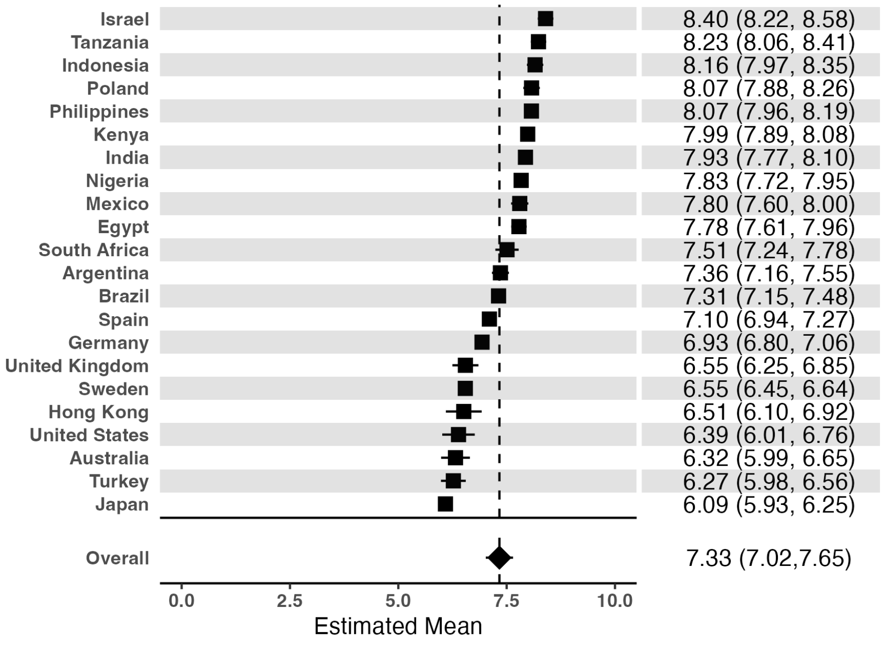

τ=0.744; Q(df=21)=1486.59, p=<2e-16;Q-profile 95% CI [0.548, 1.014]; I^2=98.73;

Figure S23b. Forest plot for `Employment status`-`Student`

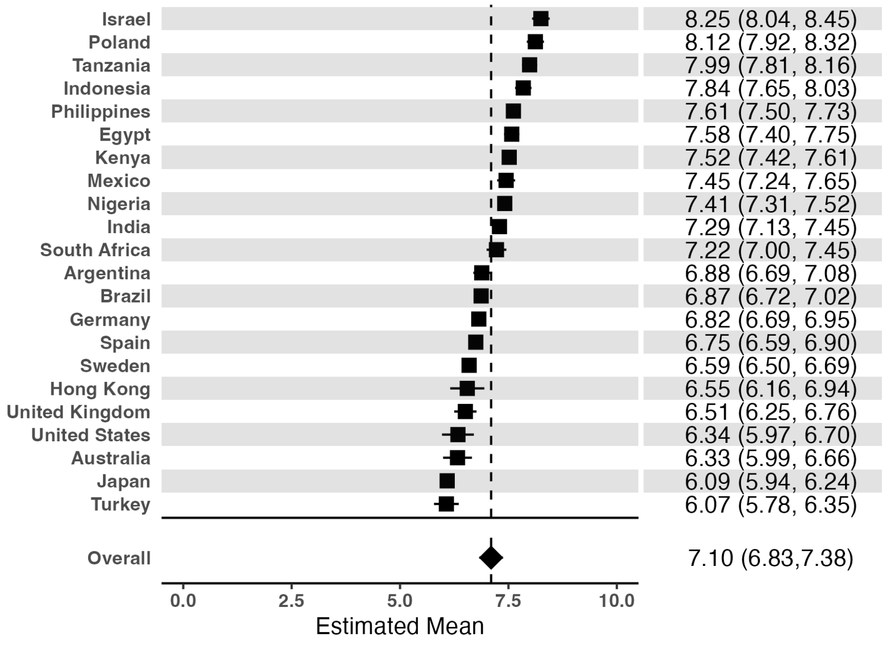

τ=0.649; Q(df=21)=1047.67, p=<2e-16;Q-profile 95% CI [0.476, 0.885]; I^2=98.44;

Figure S24. Heterogeneity in means scores across countries within group: Employment status-Unemployed and looking for a job. (a) Flourishing with financial indicators (12 items) [left panel]; (b) Flourishing without financial indicators (10 items) [right panel]. N=202,898, subgroup means and standard errors are computed accounting for the complex sampling design using all data simultaneously. Analyses conducted: Random-effects meta-analysis of country-specific means. Squares represent the the point estimate (mean) for each country. The lines represented the +/- 1.96*SE, standard error, around the mean; the overall pooled mean is represented by the diamond. The reported p-value for Q-statistics is necessarily 1-sided because of the use of the chi-squared distribution to test whether heterogeneity is greater than zero (i.e., a two-sided test is not applicable). No adjustments for multiple testing were made.

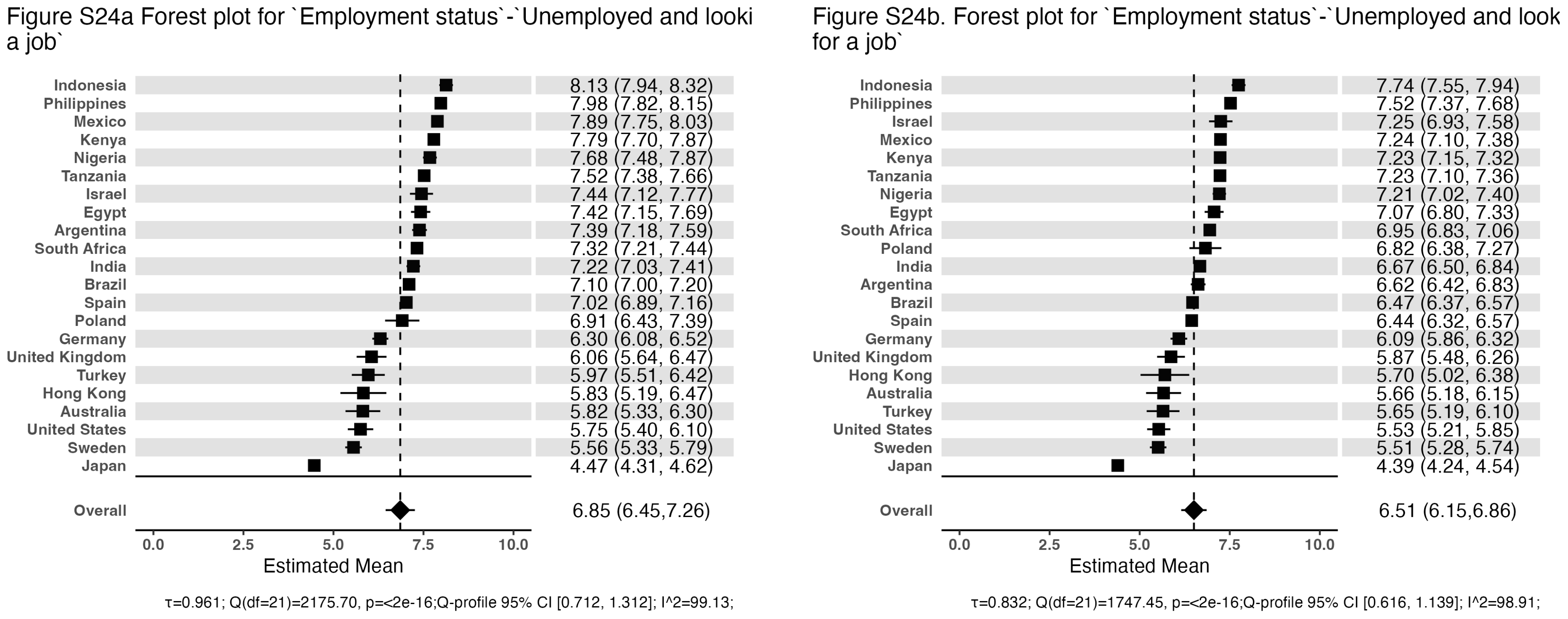

Figure S25. Heterogeneity in means scores across countries within group: Religious service attendance-A few times a year. (a) Flourishing with financial indicators (12 items) [left panel]; (b) Flourishing without financial indicators (10 items) [right panel]. N=202,898, subgroup means and standard errors are computed accounting for the complex sampling design using all data simultaneously. Analyses conducted: Random-effects meta-analysis of country-specific means. Squares represent the the point estimate (mean) for each country. The lines represented the +/-1.96*SE, standard error, around the mean; the overall pooled mean is represented by the diamond. The reported p-value for Q-statistics is necessarily 1-sided because of the use of the chi-squared distribution to test whether heterogeneity is greater than zero (i.e., a two-sided test is not applicable). No adjustments for multiple testing were made.

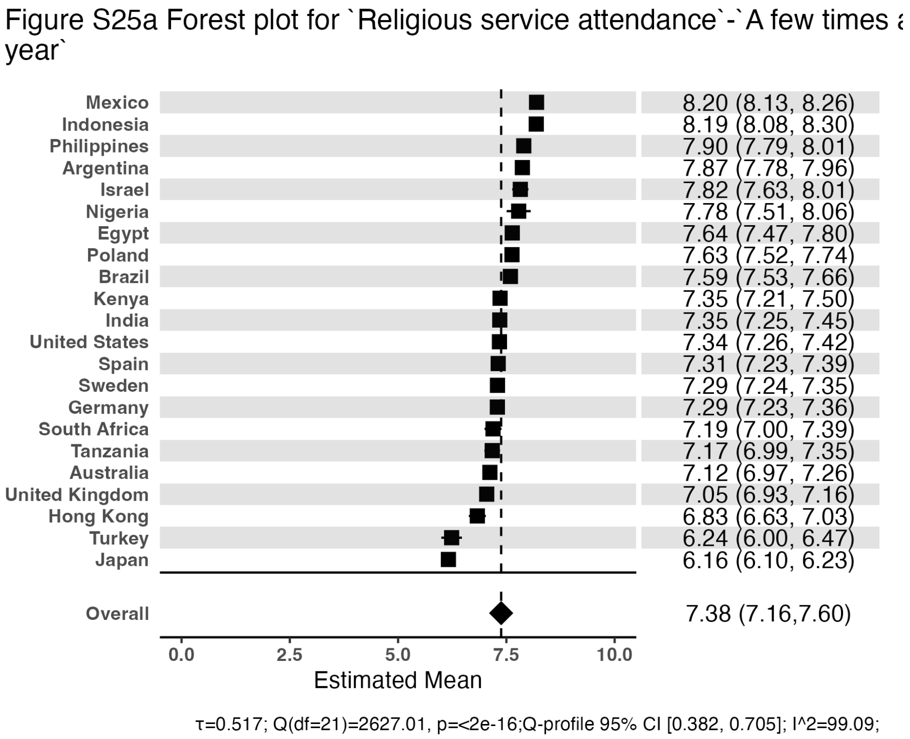

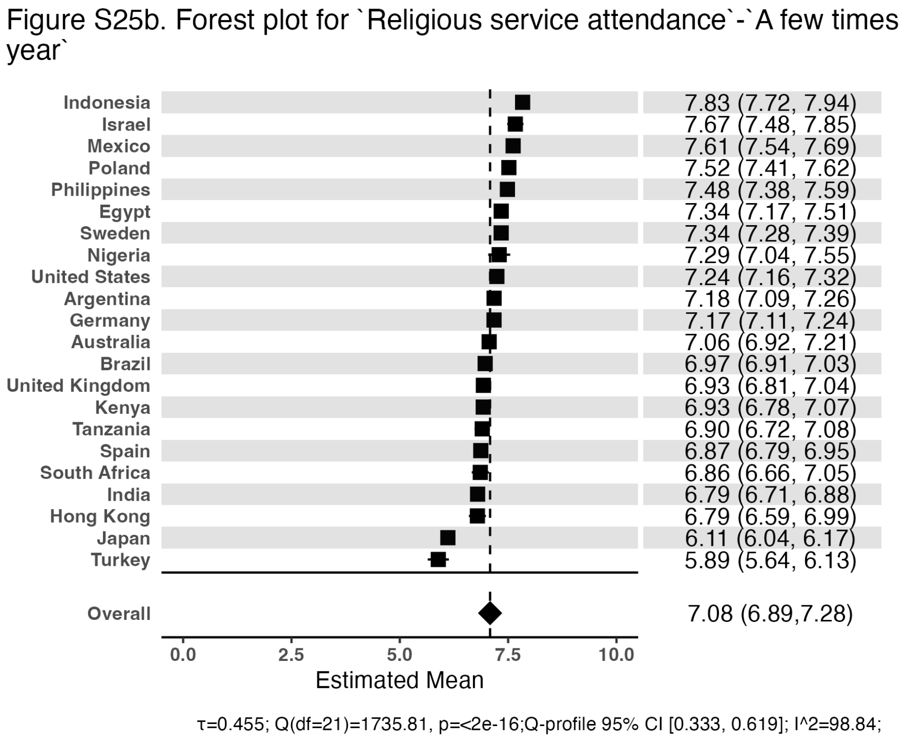

Figure S26. Heterogeneity in means scores across countries within group: Religious service attendance->1/week. (a) Flourishing with financial indicators (12 items) [left panel]; (b) Flourishing without financial indicators (10 items) [right panel]. N=202,898, subgroup means and standard errors are computed accounting for the complex sampling design using all data simultaneously. Analyses conducted: Random-effects meta-analysis of country-specific means. Squares represent the the point estimate (mean) for each country. The lines represented the +/-1.96*SE, standard error, around the mean; the overall pooled mean is represented by the diamond. The reported p-value for Q-statistics is necessarily 1-sided because of the use of the chi-squared distribution to test whether heterogeneity is greater than zero (i.e., a two-sided test is not applicable). No adjustments for multiple testing were made.

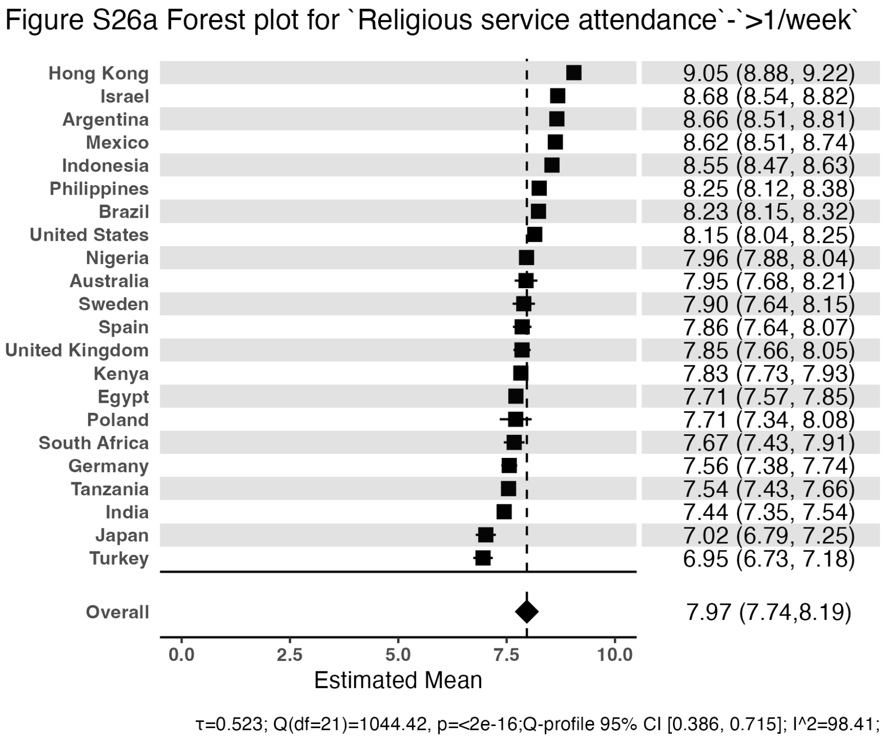

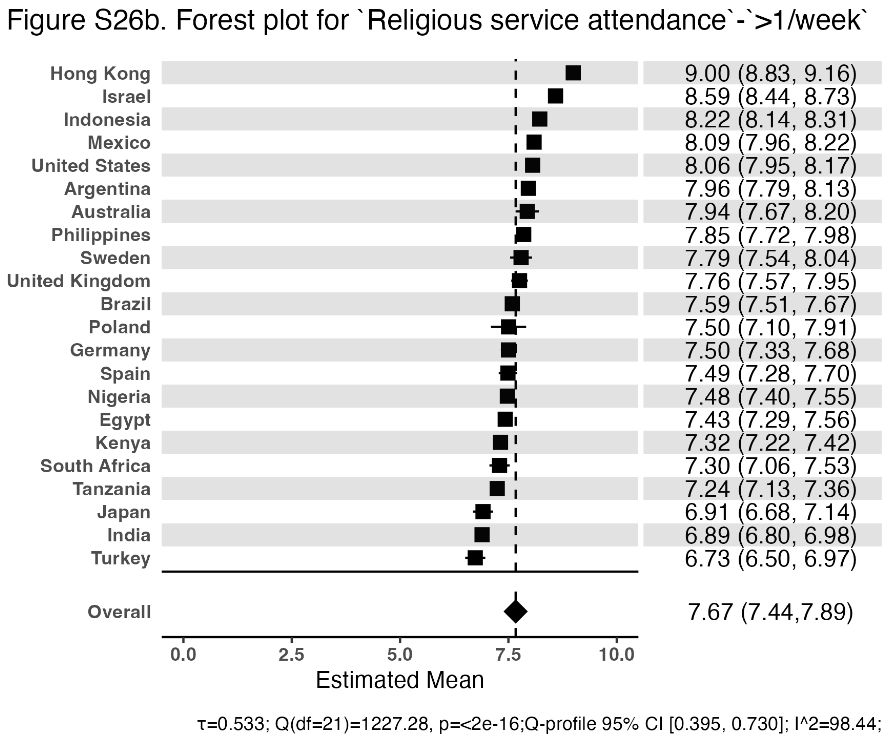

Figure S27. Heterogeneity in means scores across countries within group: Religious service attendance-Never. (a) Flourishing with financial indicators (12 items) [left panel]; (b) Flourishing without financial indicators (10 items) [right panel]. N=202,898, subgroup means and standard errors are computed accounting for the complex sampling design using all data simultaneously. Analyses conducted: Random-effects meta-analysis of country-specific means. Squares represent the the point estimate (mean) for each country. The lines represented the +/-1.96*SE, standard error, around the mean; the overall pooled mean is represented by the diamond. The reported p-value for Q-statistics is necessarily 1-sided because of the use of the chi-squared distribution to test whether heterogeneity is greater than zero (i.e., a two-sided test is not applicable). No adjustments for multiple testing were made.

Figure S27a Forest plot for `Religious service attendance`-`Never`

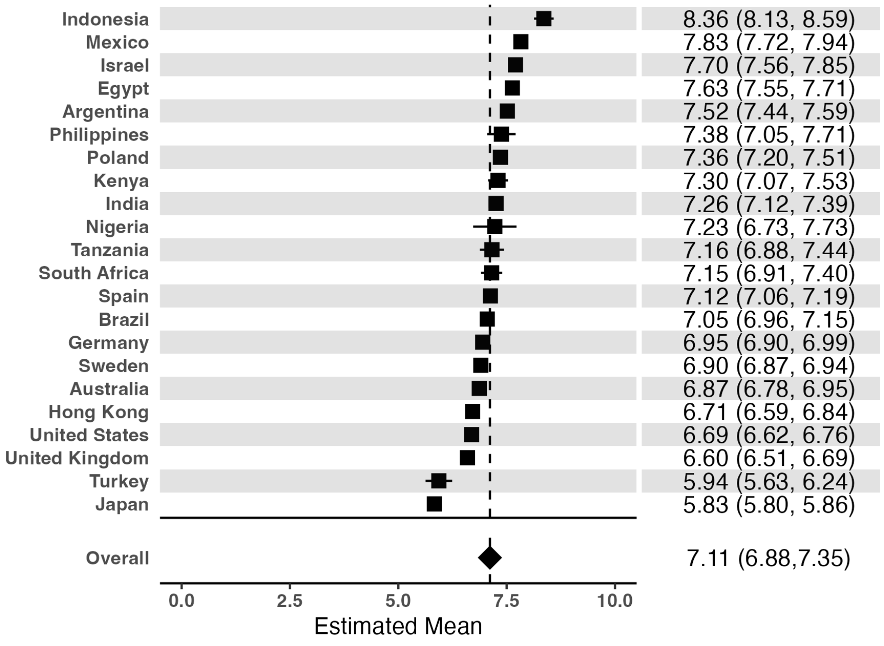

τ=0.561; Q(df=21)=5164.88, p=<2e-16;Q-profile 95% CI [0.414, 0.767]; I^2=99.46;

Figure S27b. Forest plot for `Religious service attendance`-`Never`

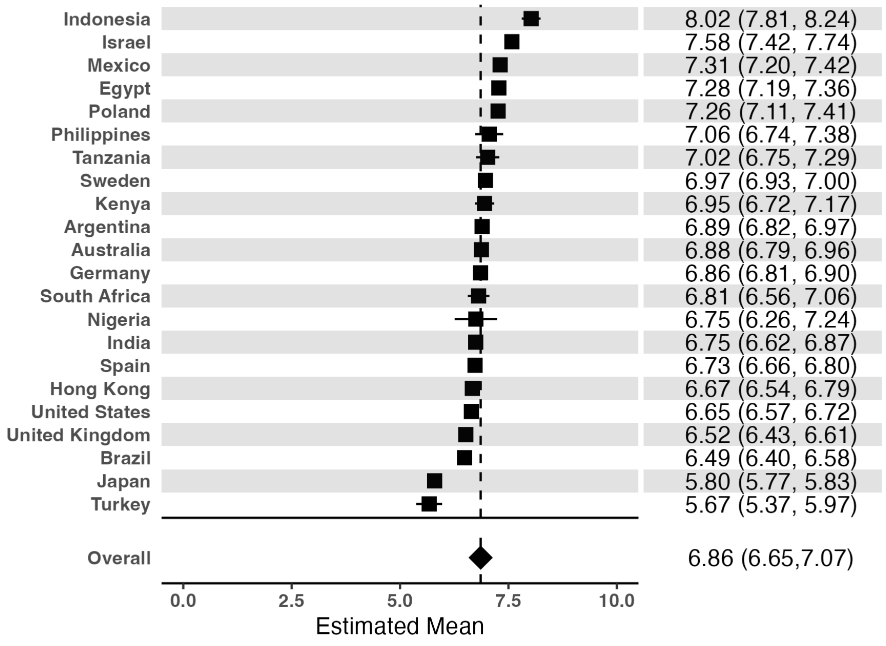

τ=0.498; Q(df=21)=3886.60, p=<2e-16;Q-profile 95% CI [0.364, 0.680]; I^2=99.32;

Figure S28. Heterogeneity in means scores across countries within group: Religious service attendance-1/week. (a) Flourishing with financial indicators (12 items) [left panel]; (b) Flourishing without financial indicators (10 items) [right panel]. N=202,898, subgroup means and standard errors are computed accounting for the complex sampling design using all data simultaneously. Analyses conducted: Random-effects meta-analysis of country-specific means. Squares represent the the point estimate (mean) for each country. The lines represented the +/-1.96*SE, standard error, around the mean; the overall pooled mean is represented by the diamond. The reported p-value for Q-statistics is necessarily 1-sided because of the use of the chi-squared distribution to test whether heterogeneity is greater than zero (i.e., a two-sided test is not applicable). No adjustments for multiple testing were made.

Figure S28a Forest plot for `Religious service attendance`-`1/week`

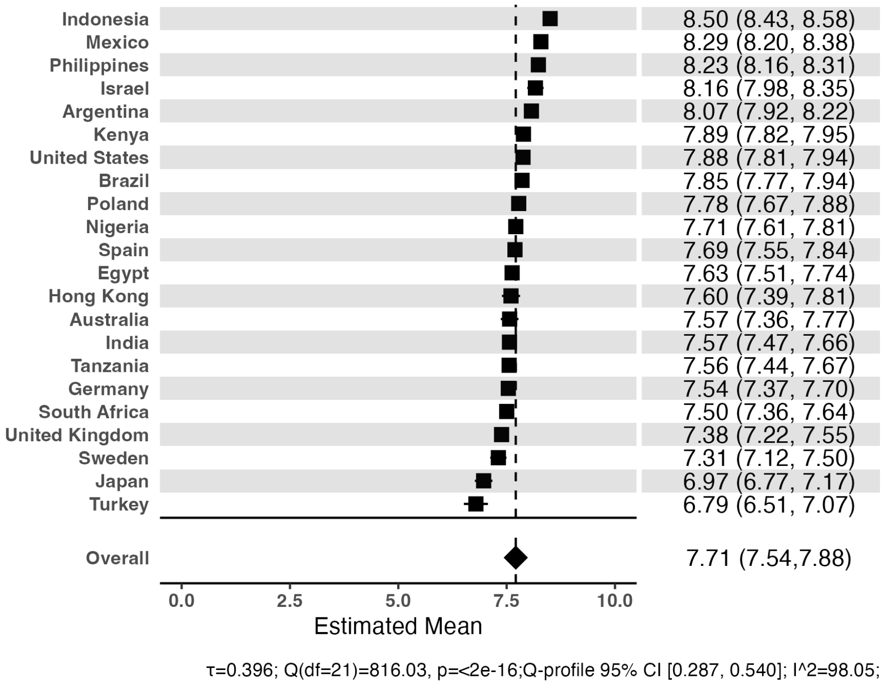

Figure S28b. Forest plot for `Religious service attendance`-`1/week`

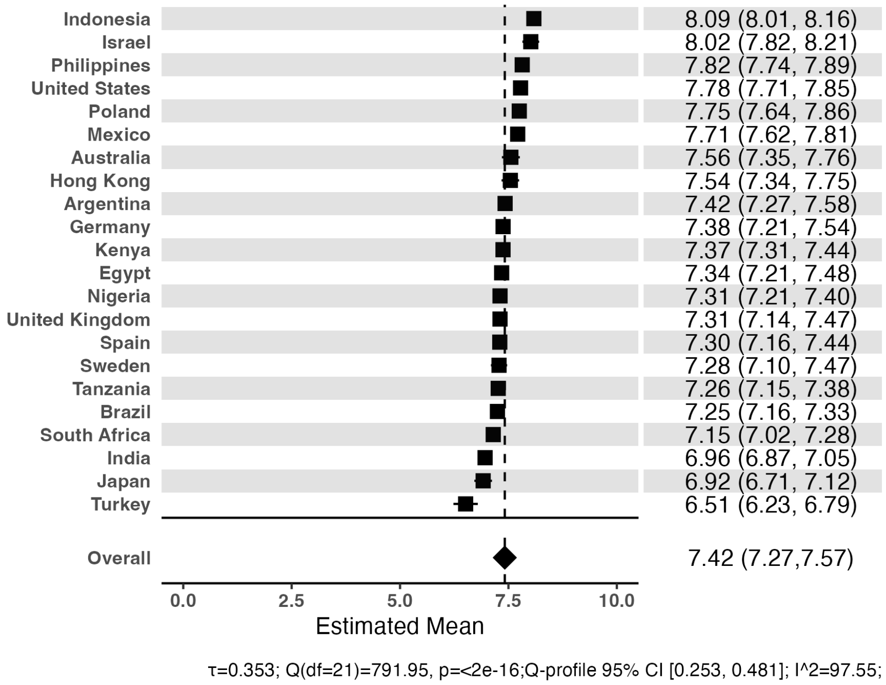

Figure S29. Heterogeneity in means scores across countries within group: Religious service attendance-1-3/month. (a) Flourishing with financial indicators (12 items) [left panel]; (b) Flourishing without financial indicators (10 items) [right panel]. N=202,898, subgroup means and standard errors are computed accounting for the complex sampling design using all data simultaneously. Analyses conducted: Random-effects meta-analysis of country-specific means. Squares represent the the point estimate (mean) for each country. The lines represented the +/-1.96*SE, standard error, around the mean; the overall pooled mean is represented by the diamond. The reported p-value for Q-statistics is necessarily 1-sided because of the use of the chi-squared distribution to test whether heterogeneity is greater than zero (i.e., a two-sided test is not applicable). No adjustments for multiple testing were made.

Figure S29a Forest plot for `Religious service attendance`-`1-3/month`

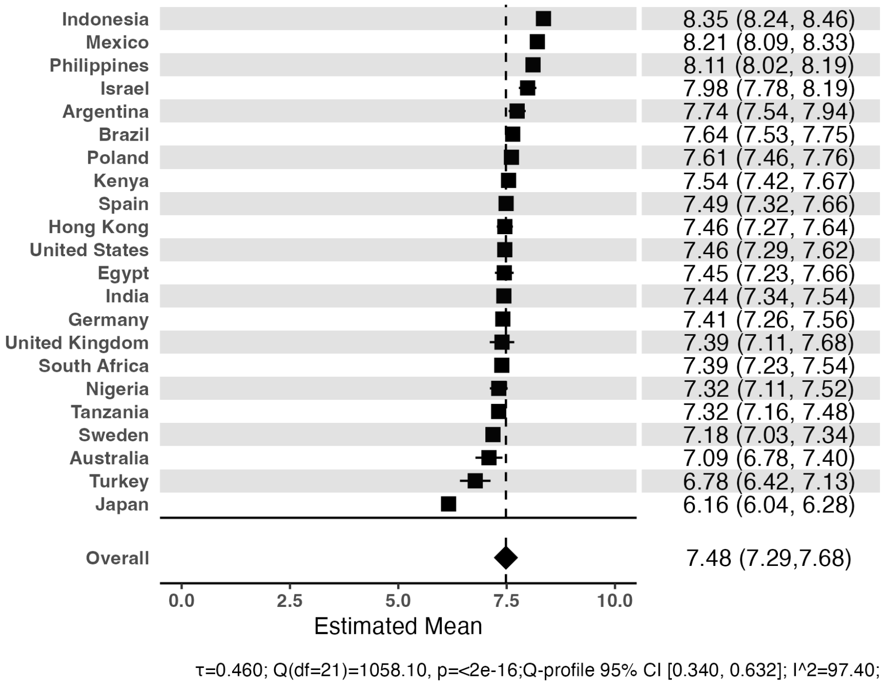

Figure S29b. Forest plot for `Religious service attendance`-`1-3/month`

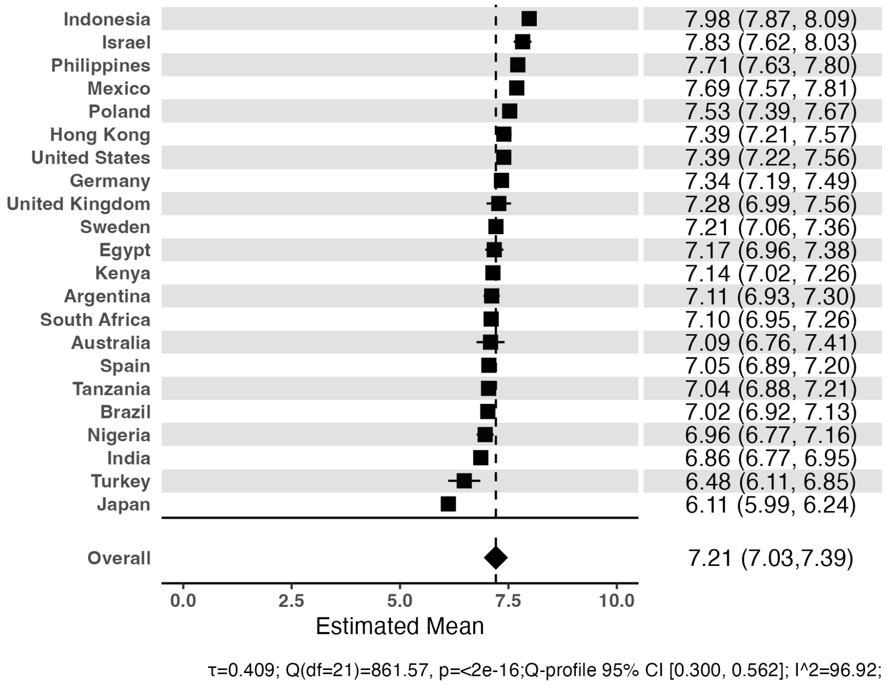

Figure S30. Heterogeneity in means scores across countries within group: Education-Up to 8 years. (a) Flourishing with financial indicators (12 items) [left panel]; (b) Flourishing without financial indicators (10 items) [right panel]. N=202,898, subgroup means and standard errors are computed accounting for the complex sampling design using all data simultaneously. Analyses conducted: Random-effects meta-analysis of country-specific means. Squares represent the the point estimate (mean) for each country. The lines represented the +/-1.96*SE, standard error, around the mean; the overall pooled mean is represented by the diamond. The reported p-value for Q-statistics is necessarily 1-sided because of the use of the chi-squared distribution to test whether heterogeneity is greater than zero (i.e., a two-sided test is not applicable). No adjustments for multiple testing were made.

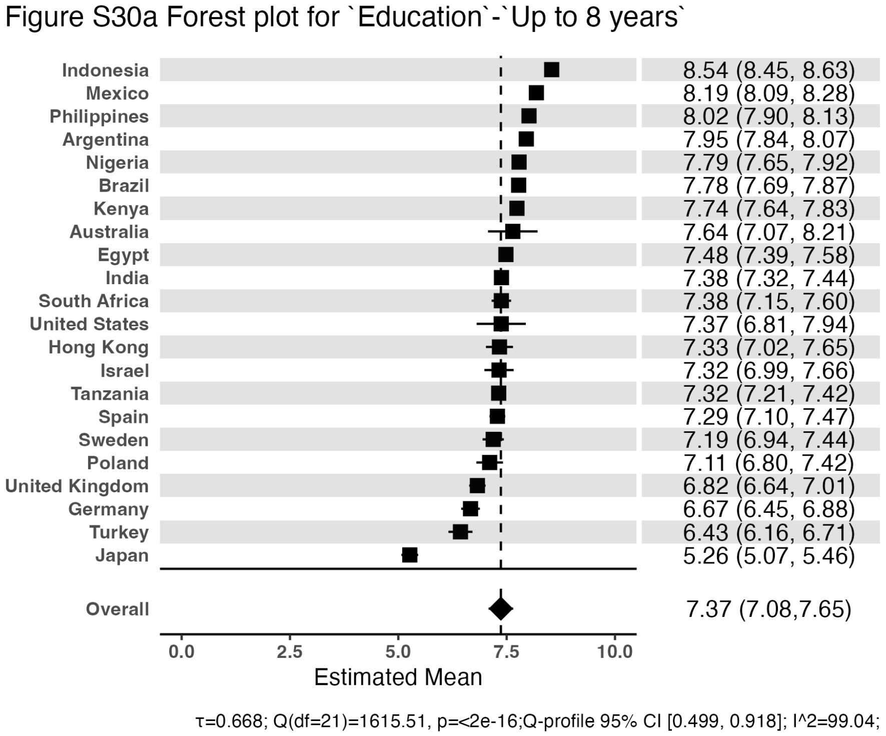

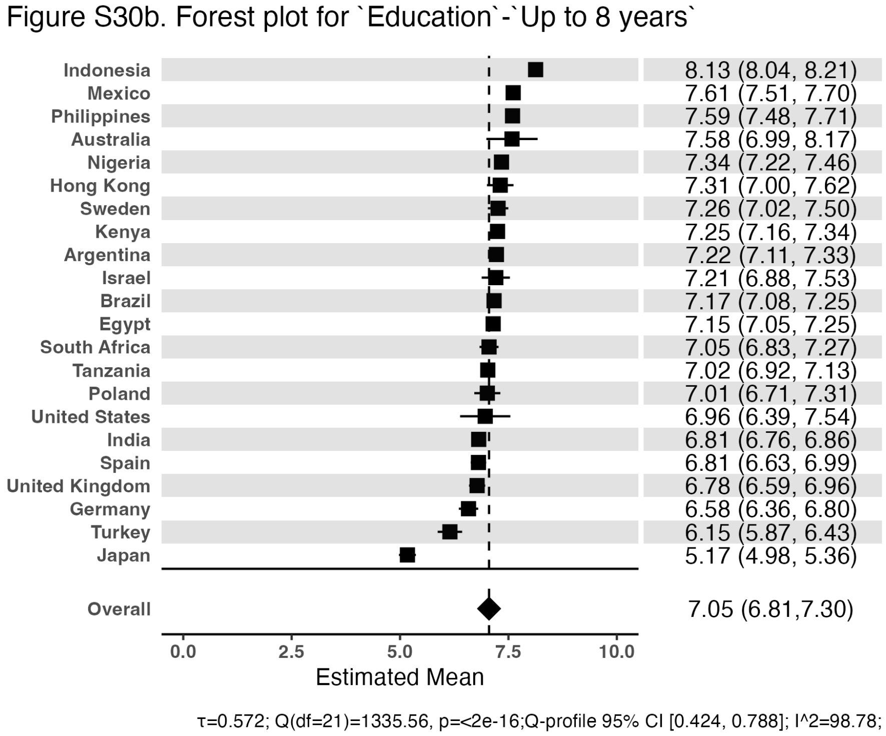

Figure S31. Heterogeneity in means scores across countries within group: Education-16+ years. (a) Flourishing with financial indicators (12 items) [left panel]; (b) Flourishing without financial indicators (10 items) [right panel]. N=202,898, subgroup means and standard errors are computed accounting for the complex sampling design using all data simultaneously. Analyses conducted: Random-effects meta-analysis of country-specific means. Squares represent the the point estimate (mean) for each country. The lines represented the +/-1.96*SE, standard error, around the mean; the overall pooled mean is represented by the diamond. The reported p-value for Q-statistics is necessarily 1-sided because of the use of the chi-squared distribution to test whether heterogeneity is greater than zero (i.e., a two-sided test is not applicable). No adjustments for multiple testing were made.

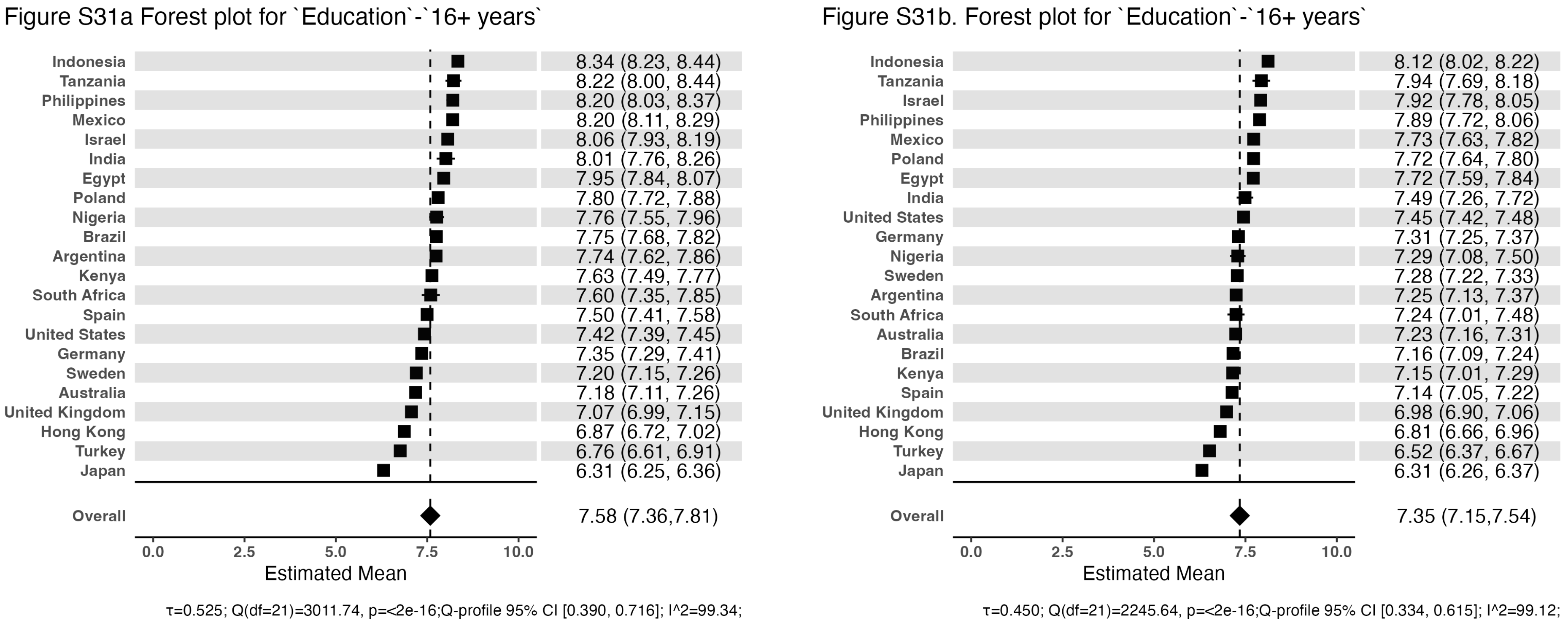

Figure S32. Heterogeneity in means scores across countries within group: Education-9-15 years. (a) Flourishing with financial indicators (12 items) [left panel]; (b) Flourishing without financial indicators (10 items) [right panel]. N=202,898, subgroup means and standard errors are computed accounting for the complex sampling design using all data simultaneously. Analyses conducted: Random-effects meta-analysis of country-specific means. Squares represent the the point estimate (mean) for each country. The lines represented the +/-1.96*SE, standard error, around the mean; the overall pooled mean is represented by the diamond. The reported p-value for Q-statistics is necessarily 1-sided because of the use of the chi-squared distribution to test whether heterogeneity is greater than zero (i.e., a two-sided test is not applicable). No adjustments for multiple testing were made.

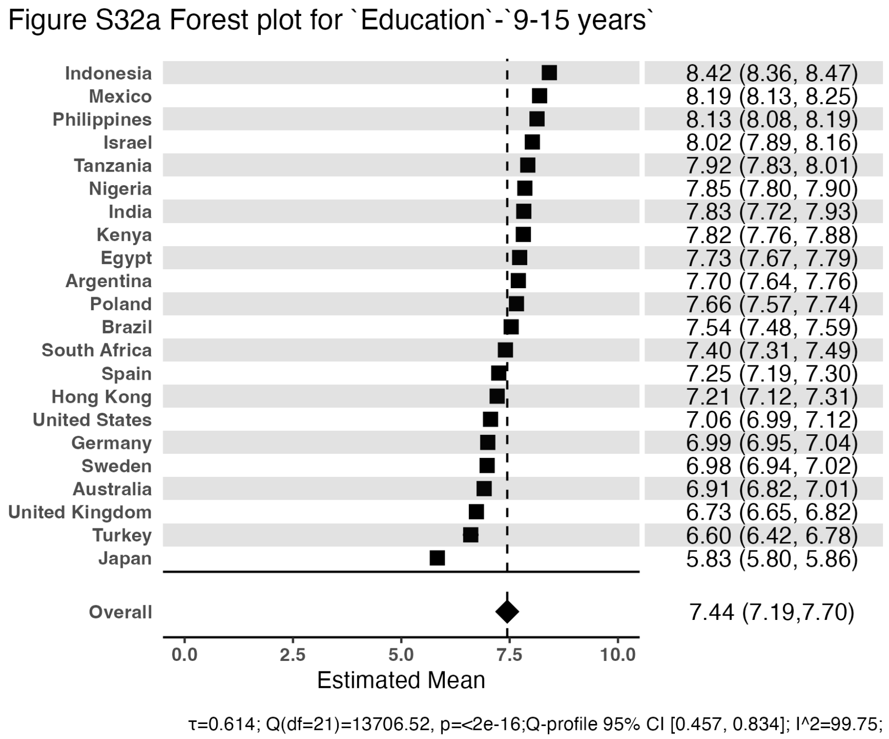

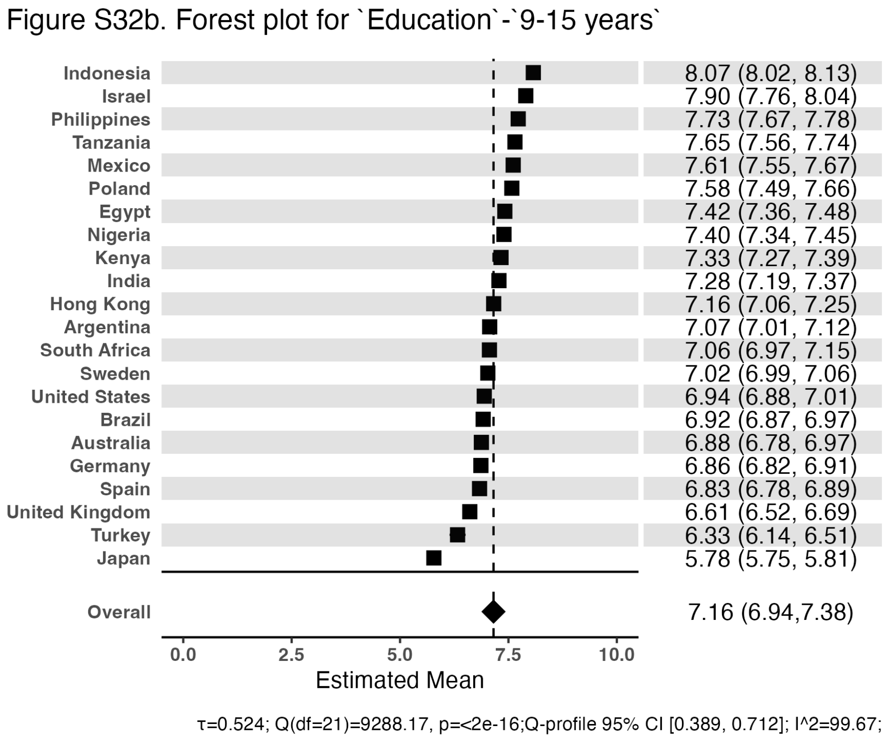

Figure S33. Heterogeneity in means scores across countries within group: Immigration status-Born in another country. (a) Flourishing with financial indicators (12 items) [left panel]; (b) Flourishing without financial indicators (10 items) [right panel]. N=202,898, subgroup means and standard errors are computed accounting for the complex sampling design using all data simultaneously. Analyses conducted: Random-effects meta-analysis of country-specific means. Squares represent the the point estimate (mean) for each country. The lines represented the +/-1.96*SE, standard error, around the mean; the overall pooled mean is represented by the diamond. The reported p-value for Q-statistics is necessarily 1-sided because of the use of the chi-squared distribution to test whether heterogeneity is greater than zero (i.e., a two-sided test is not applicable). No adjustments for multiple testing were made.

Figure S33a Forest plot for `Immigration status`-`Born in another country

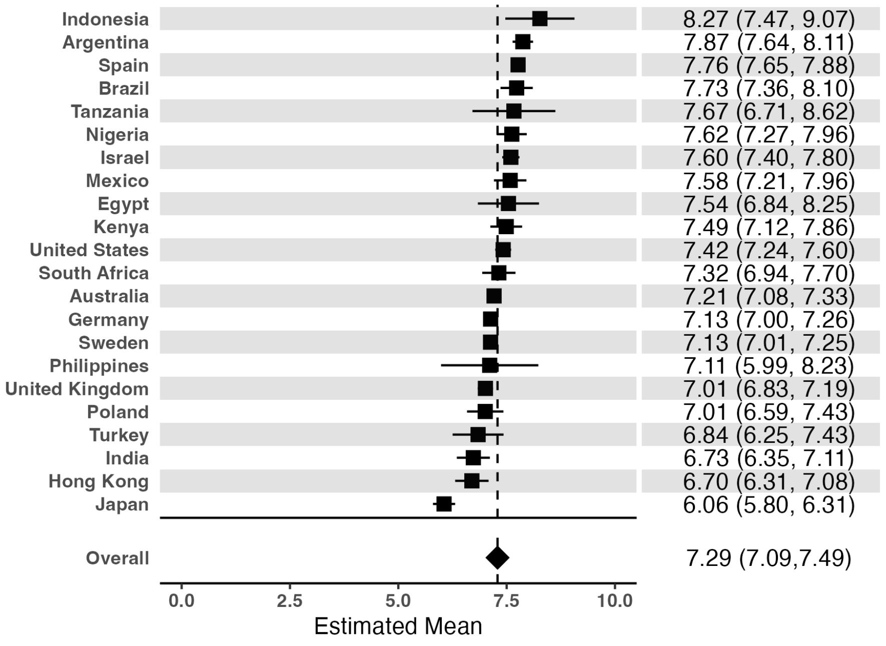

τ=0.431; Q(df=21)=250.41, p=<2e-16;Q-profile 95% CI [0.303, 0.620]; I^2=93.21;

Figure S33b. Forest plot for `Immigration status`-`Born in another countr

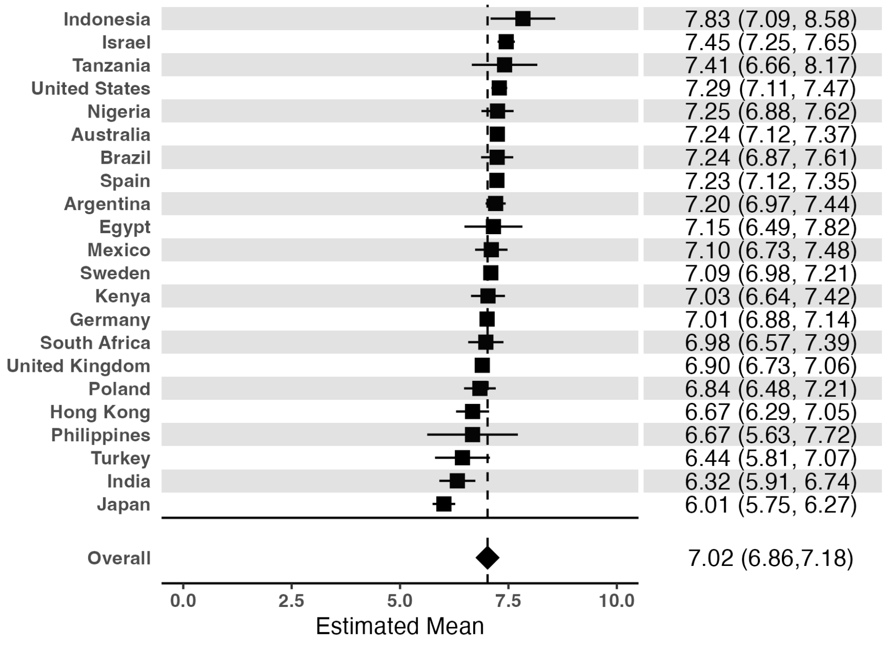

τ=0.337; Q(df=21)=132.78, p=<2e-16;Q-profile 95% CI [0.221, 0.489]; I^2=89.64;

Figure S34. Heterogeneity in means scores across countries within group: Immigration status-Born in this country. (a) Flourishing with financial indicators (12 items) [left panel]; (b) Flourishing without financial indicators (10 items) [right panel]. N=202,898, subgroup means and standard errors are computed accounting for the complex sampling design using all data simultaneously. Analyses conducted: Random-effects meta-analysis of country-specific means. Squares represent the the point estimate (mean) for each country. The lines represented the +/-1.96*SE, standard error, around the mean; the overall pooled mean is represented by the diamond. The reported p-value for Q-statistics is necessarily 1-sided because of the use of the chi-squared distribution to test whether heterogeneity is greater than zero (i.e., a two-sided test is not applicable). No adjustments for multiple testing were made.

Figure S34a Forest plot for `Immigration status`-`Born in this country`

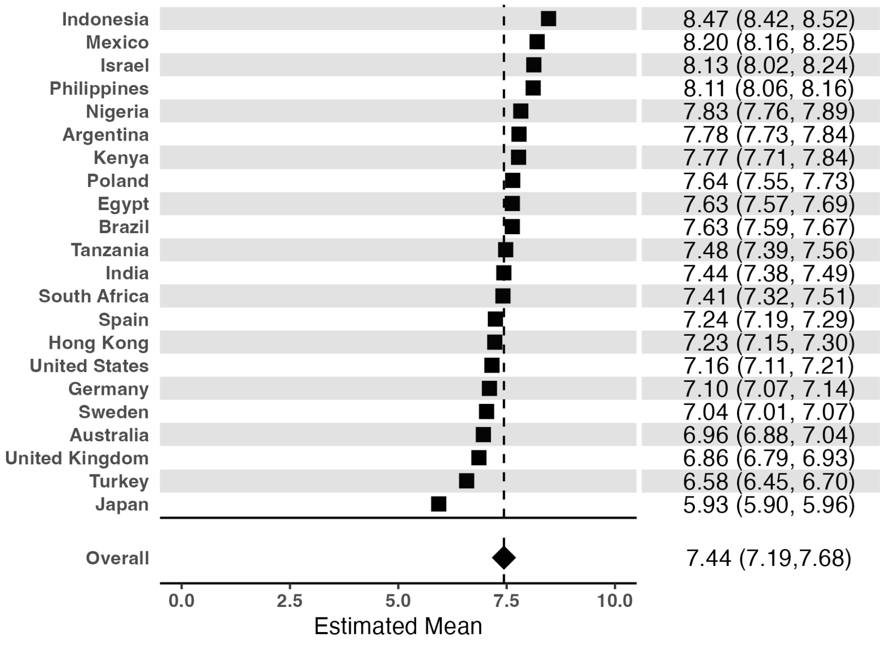

τ=0.580; Q(df=21)=15968.54, p=<2e-16;Q-profile 95% CI [0.432, 0.787]; I^2=99.79;

Figure S34b. Forest plot for `Immigration status`-`Born in this country`

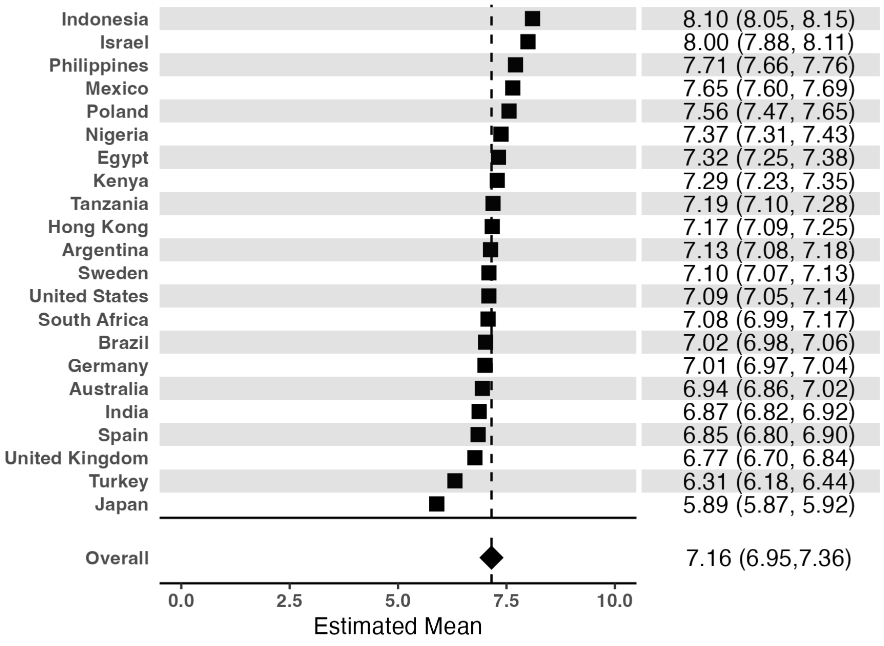

τ=0.493; Q(df=21)=10303.35, p=<2e-16;Q-profile 95% CI [0.367, 0.670]; I^2=99.72;

Part 3. Forest plots of pairwise differences among means within demographic groups.
a. Secure Flourishing Index; b. Flourishing Index (does not contain financial items)

Figure S35. Heterogeneity in pairwise compairisons acrossx countries Age group-(Ref: 18-24) 25-29. (a) Flourishing with financial indicators (12 items) [left panel]; (b) Flourishing without financial indicators (10 items) [right panel]. N=202,898, subgroup means and standard errors are computed accounting for the complex sampling design using all data simultaneously. Analyses conducted: Random-effects meta-analysis of country-specific means. Squares represent the the point estimate (mean) for each country. The lines represented the +/-1.96*SE, standard error, around the mean; the overall pooled mean is represented by the diamond. The reported p-value for Q-statistics is necessarily 1-sided because of the use of the chi-squared distribution to test whether heterogeneity is greater than zero (i.e., a two-sided test is not applicable). No adjustments for multiple testing were made.

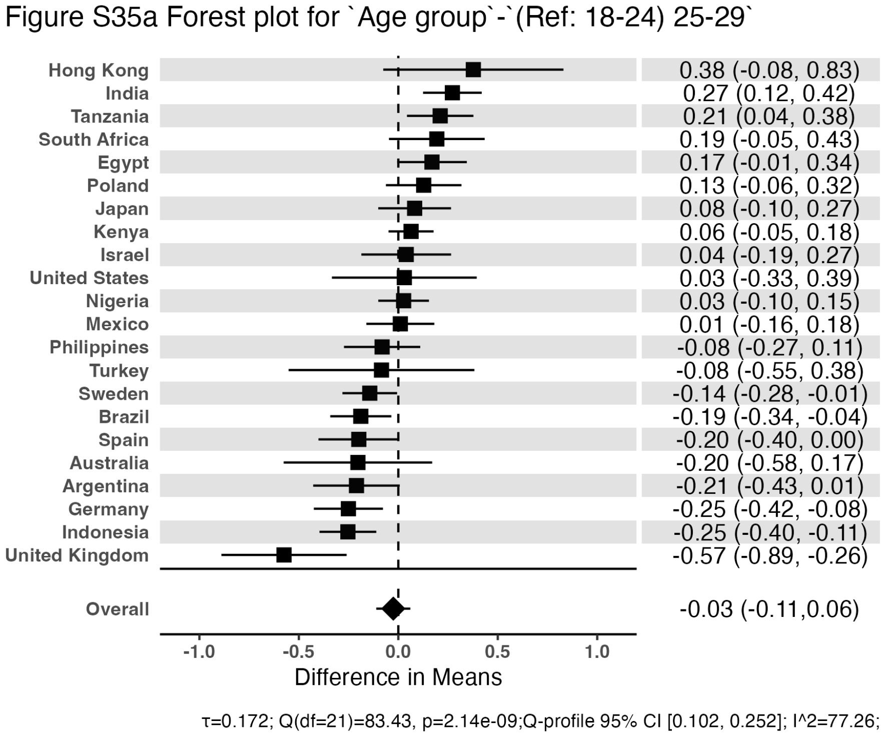

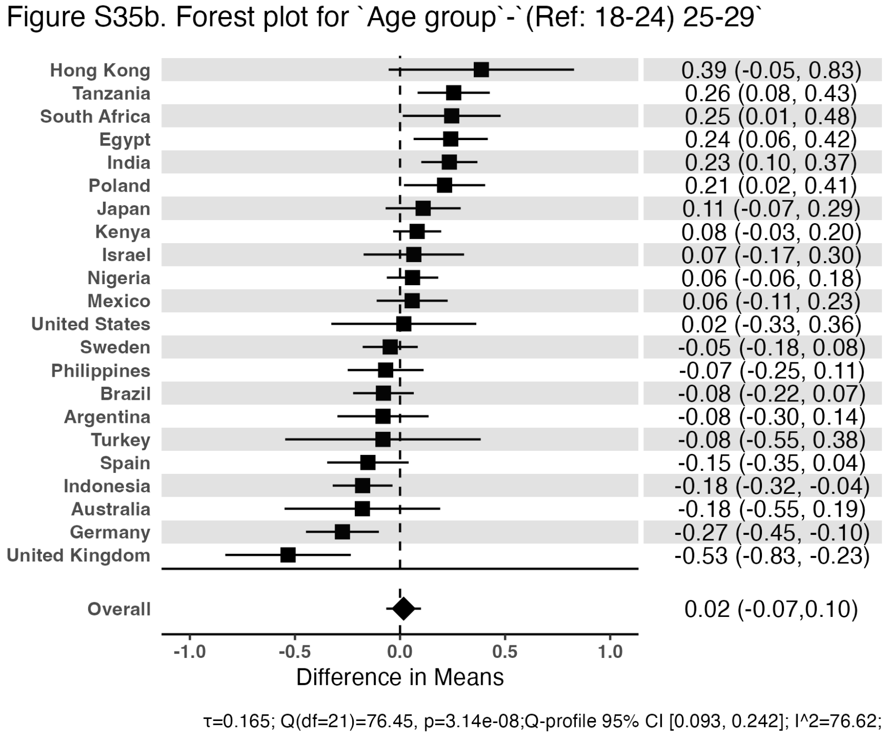

Figure S36. Heterogeneity in pairwise compairisons acrossx countries Age group-(Ref: 18-24) 30-39. (a) Flourishing with financial indicators (12 items) [left panel]; (b) Flourishing without financial indicators (10 items) [right panel]. N=202,898, subgroup means and standard errors are computed accounting for the complex sampling design using all data simultaneously. Analyses conducted: Random-effects meta-analysis of country-specific means. Squares represent the the point estimate (mean) for each country. The lines represented the +/-1.96*SE, standard error, around the mean; the overall pooled mean is represented by the diamond. The reported p-value for Q-statistics is necessarily 1-sided because of the use of the chi-squared distribution to test whether heterogeneity is greater than zero (i.e., a two-sided test is not applicable). No adjustments for multiple testing were made.

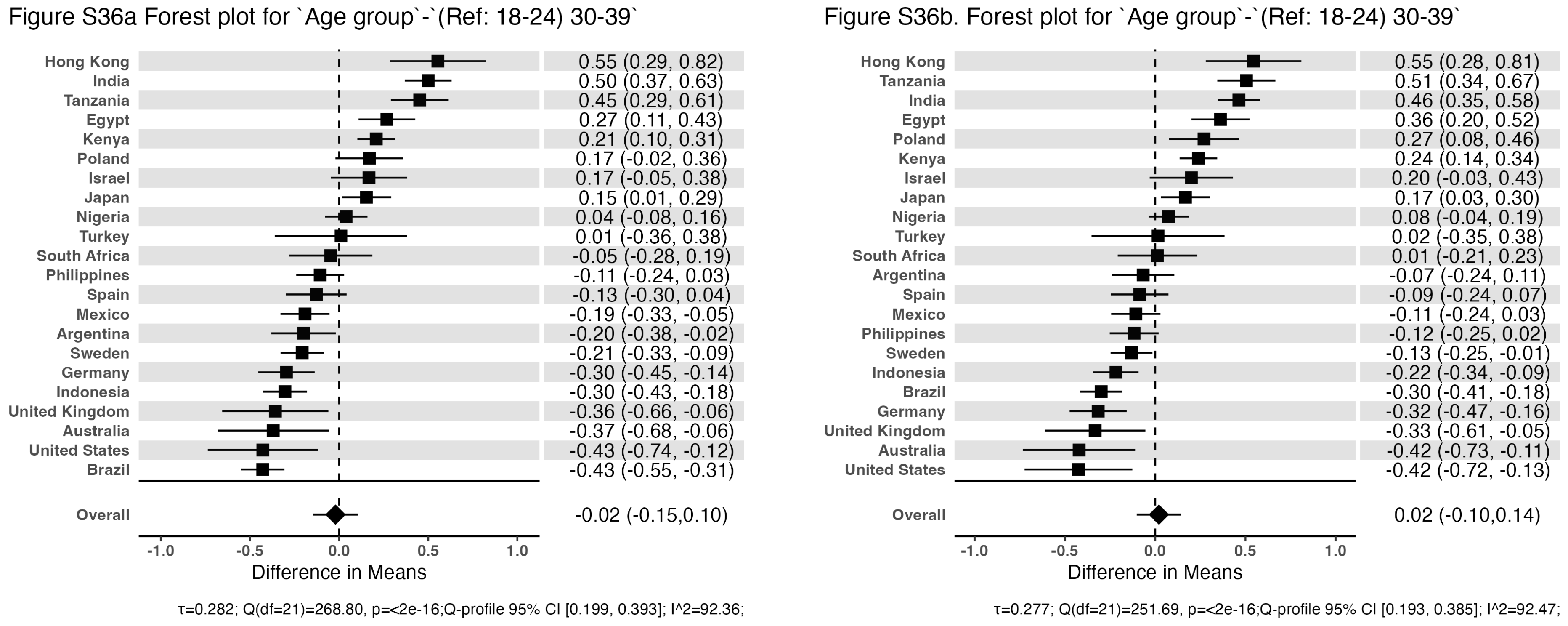

Figure S37. Heterogeneity in pairwise compairisons acrossx countries Age group-(Ref: 18-24) 40-49. (a) Flourishing with financial indicators (12 items) [left panel]; (b) Flourishing without financial indicators (10 items) [right panel]. N=202,898, subgroup means and standard errors are computed accounting for the complex sampling design using all data simultaneously. Analyses conducted: Random-effects meta-analysis of country-specific means. Squares represent the the point estimate (mean) for each country. The lines represented the +/-1.96*SE, standard error, around the mean; the overall pooled mean is represented by the diamond. The reported p-value for Q-statistics is necessarily 1-sided because of the use of the chi-squared distribution to test whether heterogeneity is greater than zero (i.e., a two-sided test is not applicable). No adjustments for multiple testing were made.

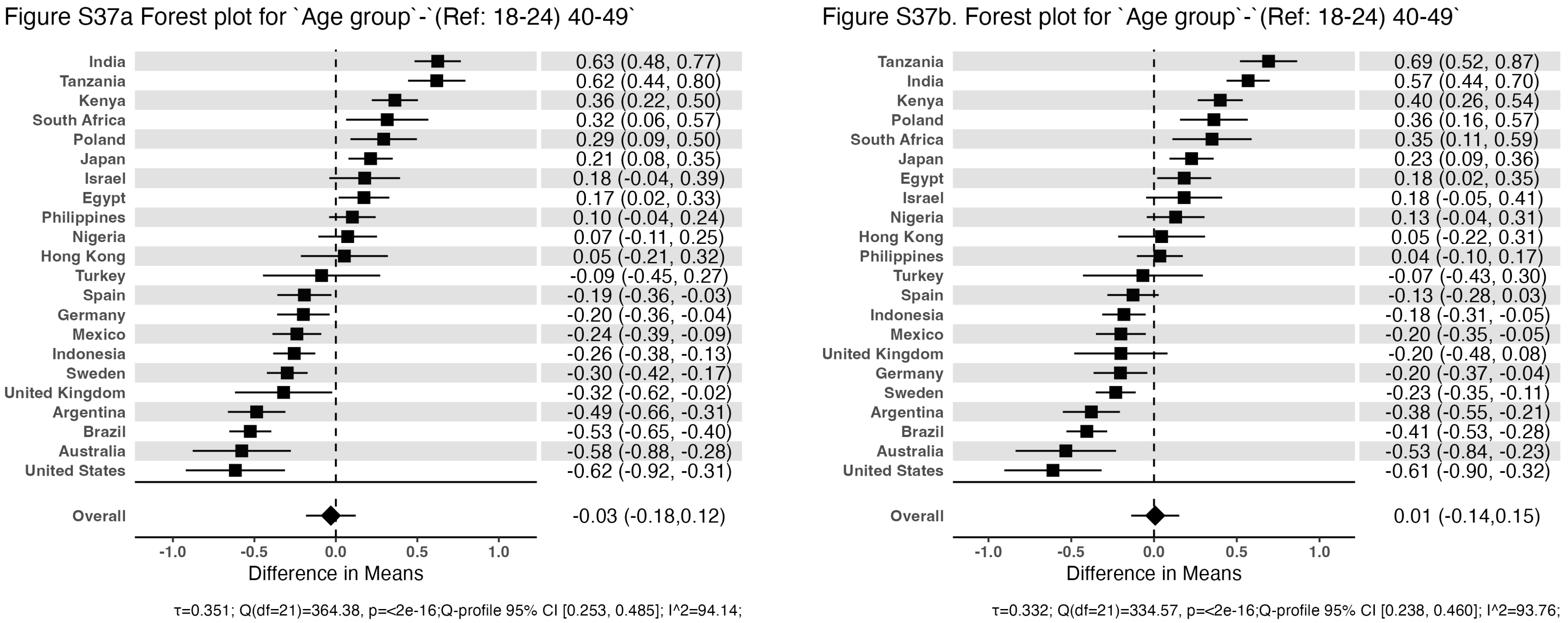

Figure S38. Heterogeneity in pairwise compairisons acrossx countries Age group-(Ref: 18-24) 50-59. (a) Flourishing with financial indicators (12 items) [left panel]; (b) Flourishing without financial indicators (10 items) [right panel]. N=202,898, subgroup means and standard errors are computed accounting for the complex sampling design using all data simultaneously. Analyses conducted: Random-effects meta-analysis of country-specific means. Squares represent the the point estimate (mean) for each country. The lines represented the +/-1.96*SE, standard error, around the mean; the overall pooled mean is represented by the diamond. The reported p-value for Q-statistics is necessarily 1-sided because of the use of the chi-squared distribution to test whether heterogeneity is greater than zero (i.e., a two-sided test is not applicable). No adjustments for multiple testing were made.

Figure S38a Forest plot for `Age group`-`(Ref: 18-24) 50-59`

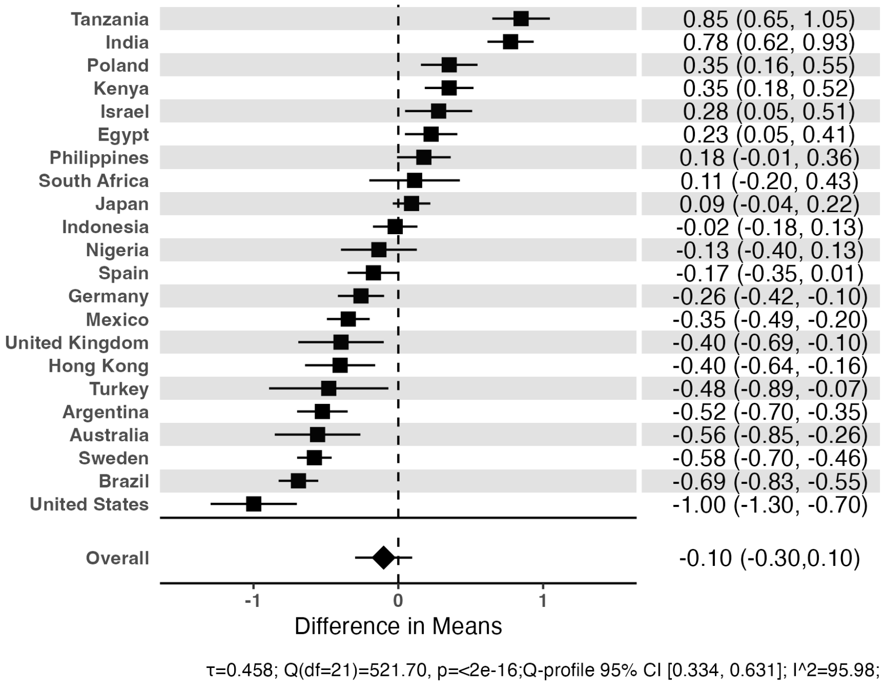

Figure S38b. Forest plot for `Age group`-`(Ref: 18-24) 50-59`

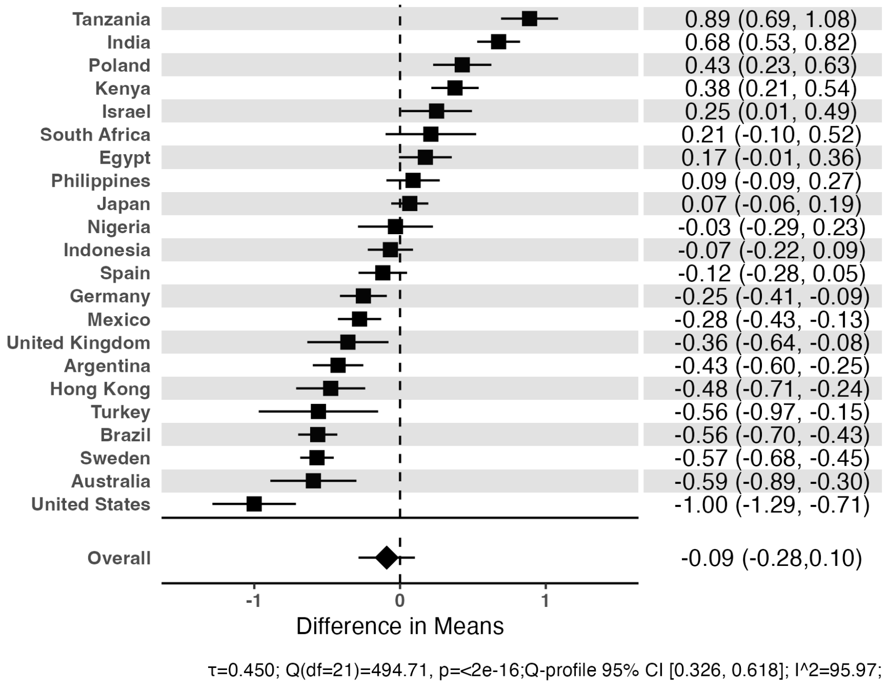

Figure S39. Heterogeneity in pairwise compairisons acrossx countries Age group-(Ref: 18-24) 60-69. (a) Flourishing with financial indicators (12 items) [left panel]; (b) Flourishing without financial indicators (10 items) [right panel]. N=202,898, subgroup means and standard errors are computed accounting for the complex sampling design using all data simultaneously. Analyses conducted: Random-effects meta-analysis of country-specific means. Squares represent the the point estimate (mean) for each country. The lines represented the +/-1.96*SE, standard error, around the mean; the overall pooled mean is represented by the diamond. The reported p-value for Q-statistics is necessarily 1-sided because of the use of the chi-squared distribution to test whether heterogeneity is greater than zero (i.e., a two-sided test is not applicable). No adjustments for multiple testing were made.

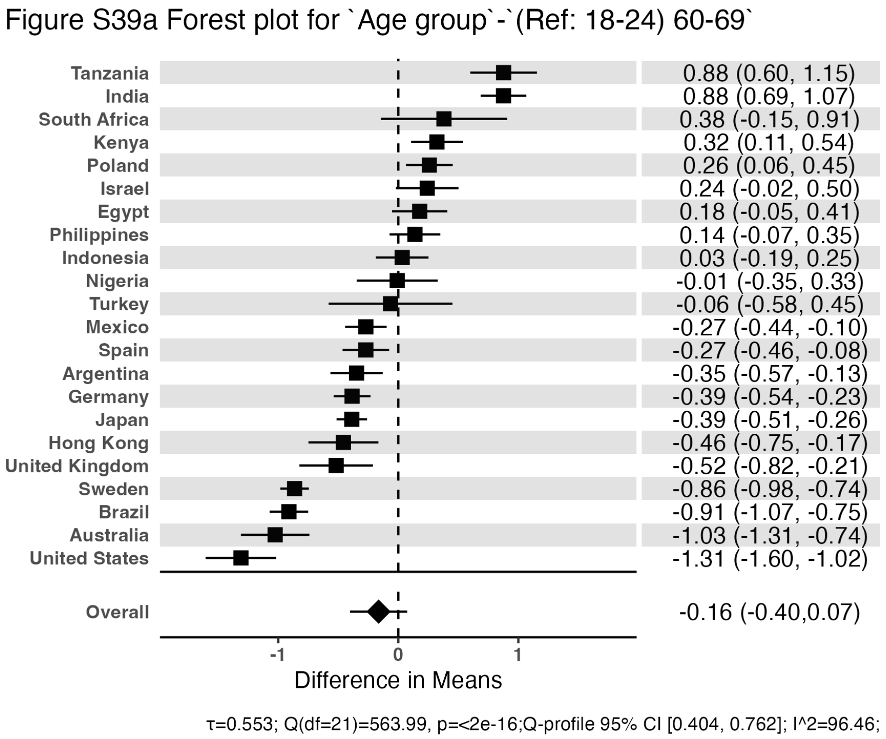

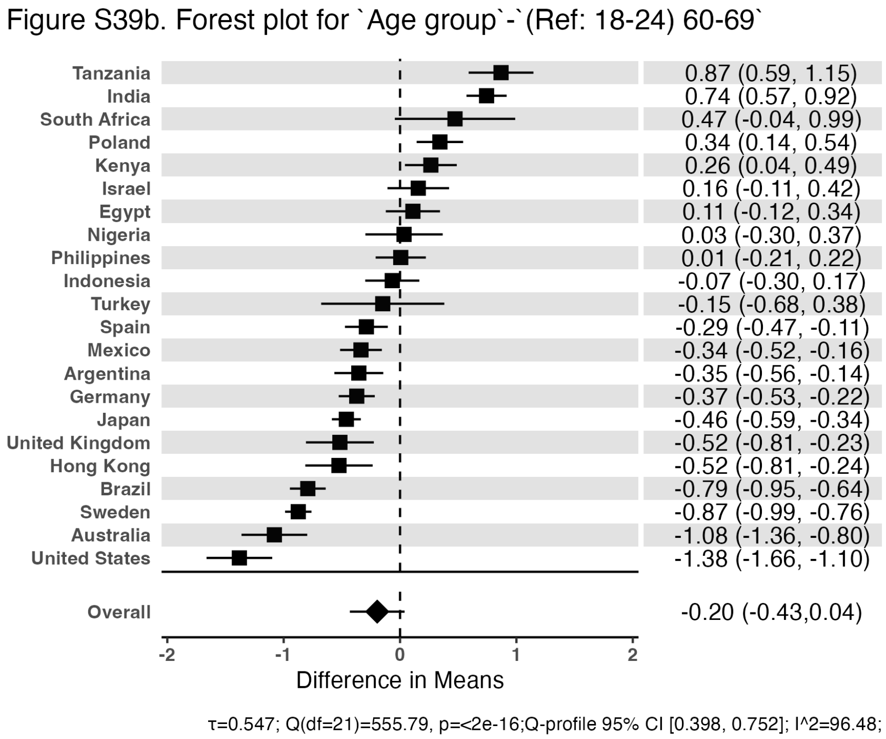

Figure S40. Heterogeneity in pairwise compairisons acrossx countries Age group-(Ref: 18-24) 70-79. (a) Flourishing with financial indicators (12 items) [left panel]; (b) Flourishing without financial indicators (10 items) [right panel]. N=202,898, subgroup means and standard errors are computed accounting for the complex sampling design using all data simultaneously. Analyses conducted: Random-effects meta-analysis of country-specific means. Squares represent the the point estimate (mean) for each country. The lines represented the +/-1.96*SE, standard error, around the mean; the overall pooled mean is represented by the diamond. The reported p-value for Q-statistics is necessarily 1-sided because of the use of the chi-squared distribution to test whether heterogeneity is greater than zero (i.e., a two-sided test is not applicable). No adjustments for multiple testing were made.

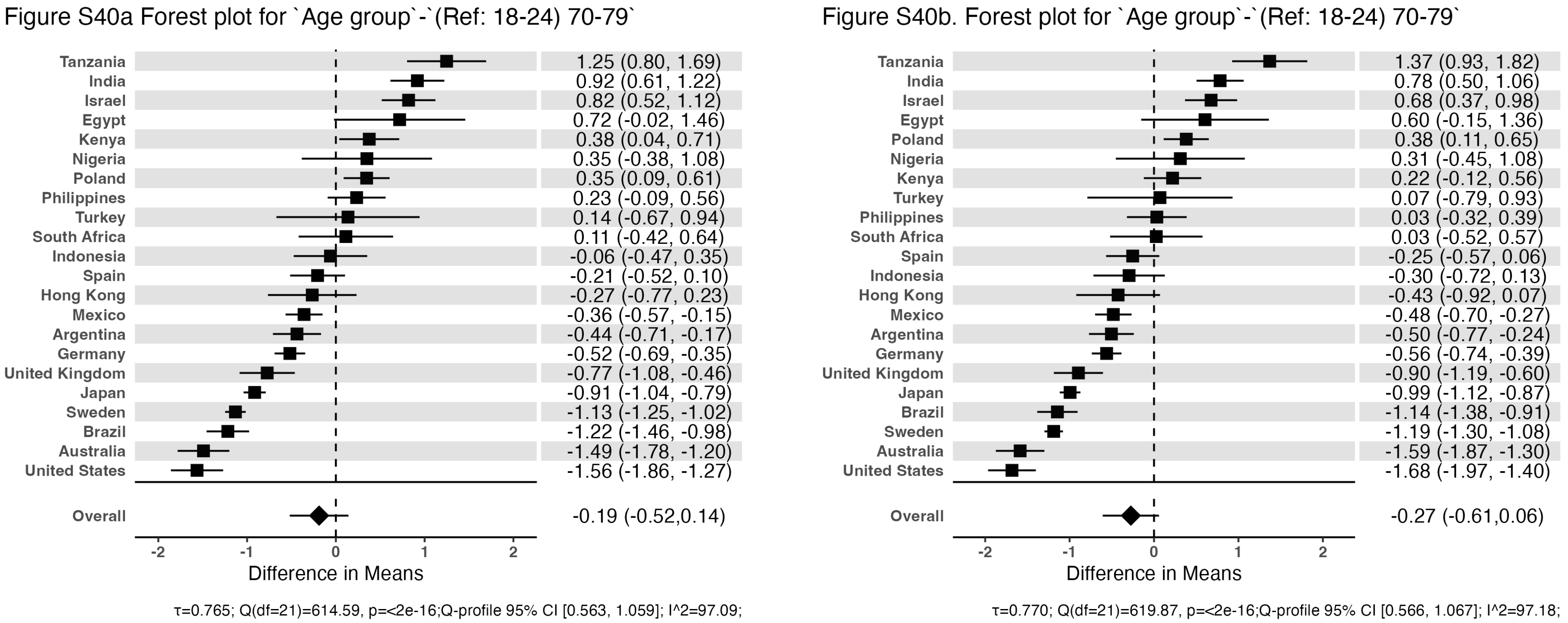

Figure S41. Heterogeneity in pairwise compairisons acrossx countries Age group-(Ref: 18-24) 80 or older. (a) Flourishing with financial indicators (12 items) [left panel]; (b) Flourishing without financial indicators (10 items) [right panel]. N=202,898, subgroup means and standard errors are computed accounting for the complex sampling design using all data simultaneously. Analyses conducted: Random-effects meta-analysis of country-specific means. Squares represent the the point estimate (mean) for each country. The lines represented the +/-1.96*SE, standard error, around the mean; the overall pooled mean is represented by the diamond. The reported p-value for Q-statistics is necessarily 1-sided because of the use of the chi-squared distribution to test whether heterogeneity is greater than zero (i.e., a two-sided test is not applicable). No adjustments for multiple testing were made.

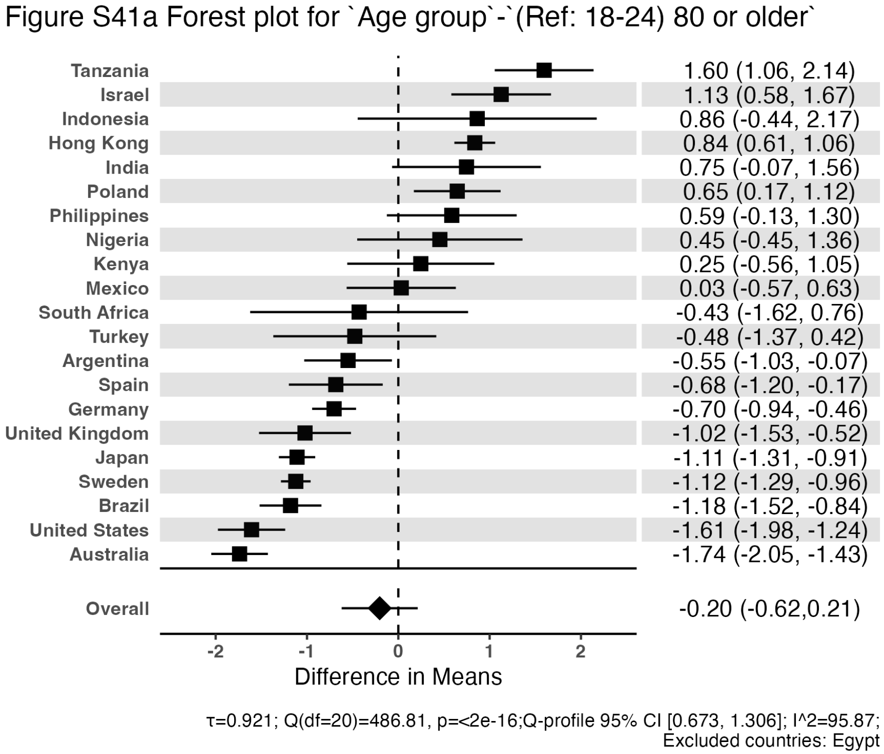

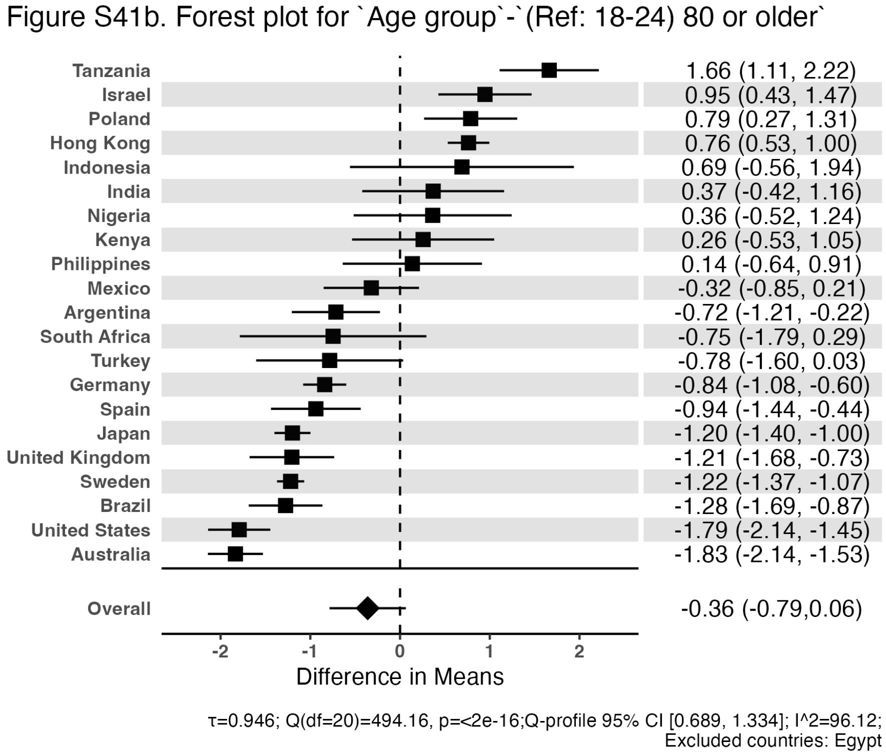

Figure S42. Heterogeneity in pairwise compairisons acrossx countries Age group-(Ref: 25-29) 30-39. (a) Flourishing with financial indicators (12 items) [left panel]; (b) Flourishing without financial indicators (10 items) [right panel]. N=202,898, subgroup means and standard errors are computed accounting for the complex sampling design using all data simultaneously. Analyses conducted: Random-effects meta-analysis of country-specific means. Squares represent the the point estimate (mean) for each country. The lines represented the +/-1.96*SE, standard error, around the mean; the overall pooled mean is represented by the diamond. The reported p-value for Q-statistics is necessarily 1-sided because of the use of the chi-squared distribution to test whether heterogeneity is greater than zero (i.e., a two-sided test is not applicable). No adjustments for multiple testing were made.

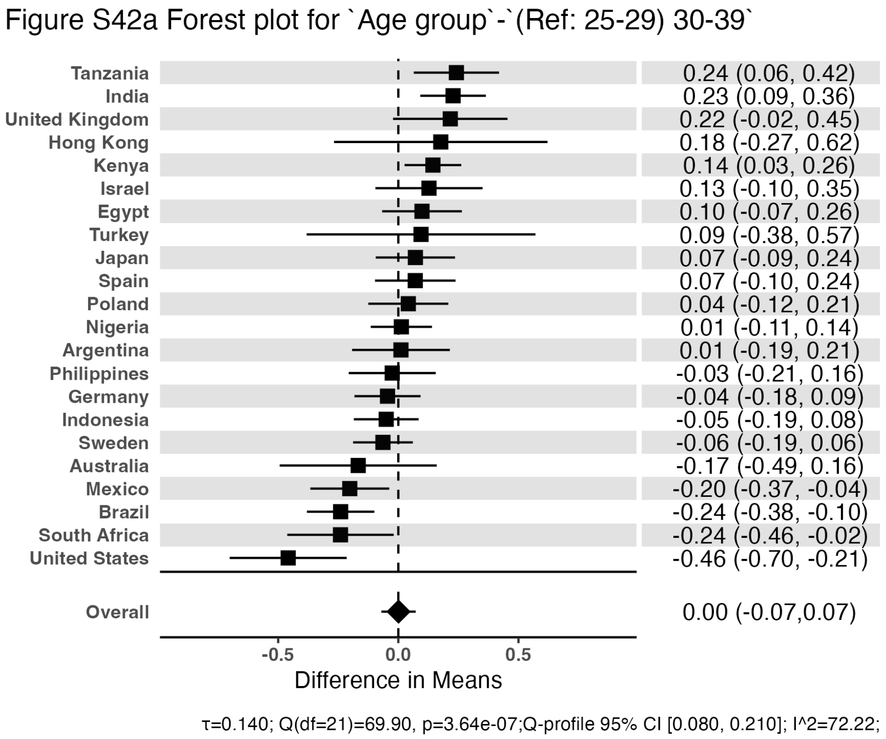

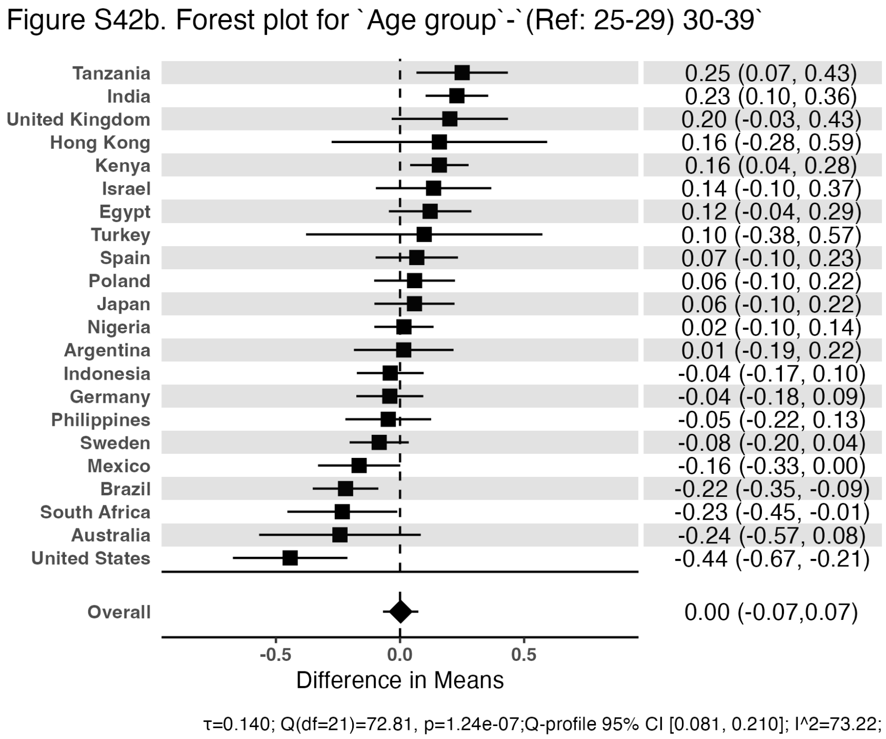

Figure S43. Heterogeneity in pairwise compairisons acrossx countries Age group-(Ref: 25-29) 40-49. (a) Flourishing with financial indicators (12 items) [left panel]; (b) Flourishing without financial indicators (10 items) [right panel]. N=202,898, subgroup means and standard errors are computed accounting for the complex sampling design using all data simultaneously. Analyses conducted: Random-effects meta-analysis of country-specific means. Squares represent the the point estimate (mean) for each country. The lines represented the +/-1.96*SE, standard error, around the mean; the overall pooled mean is represented by the diamond. The reported p-value for Q-statistics is necessarily 1-sided because of the use of the chi-squared distribution to test whether heterogeneity is greater than zero (i.e., a two-sided test is not applicable). No adjustments for multiple testing were made.

Figure S43a Forest plot for `Age group`-`(Ref: 25-29) 40-49`

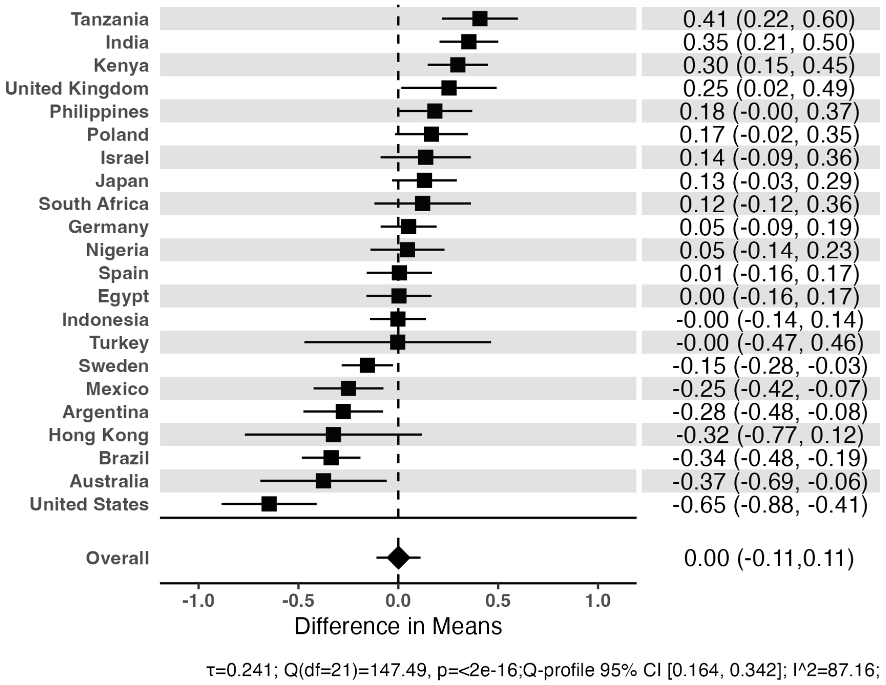

Figure S43b. Forest plot for `Age group`-`(Ref: 25-29) 40-49`

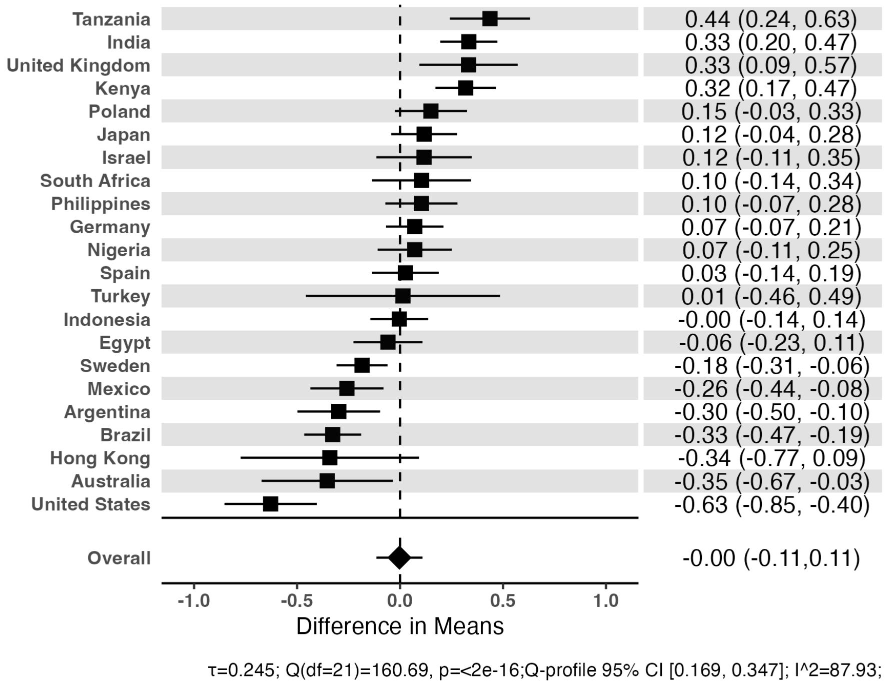

Figure S44. Heterogeneity in pairwise compairisons acrossx countries Age group-(Ref: 25-29) 50-59. (a) Flourishing with financial indicators (12 items) [left panel]; (b) Flourishing without financial indicators (10 items) [right panel]. N=202,898, subgroup means and standard errors are computed accounting for the complex sampling design using all data simultaneously. Analyses conducted: Random-effects meta-analysis of country-specific means. Squares represent the the point estimate (mean) for each country. The lines represented the +/-1.96*SE, standard error, around the mean; the overall pooled mean is represented by the diamond. The reported p-value for Q-statistics is necessarily 1-sided because of the use of the chi-squared distribution to test whether heterogeneity is greater than zero (i.e., a two-sided test is not applicable). No adjustments for multiple testing were made.

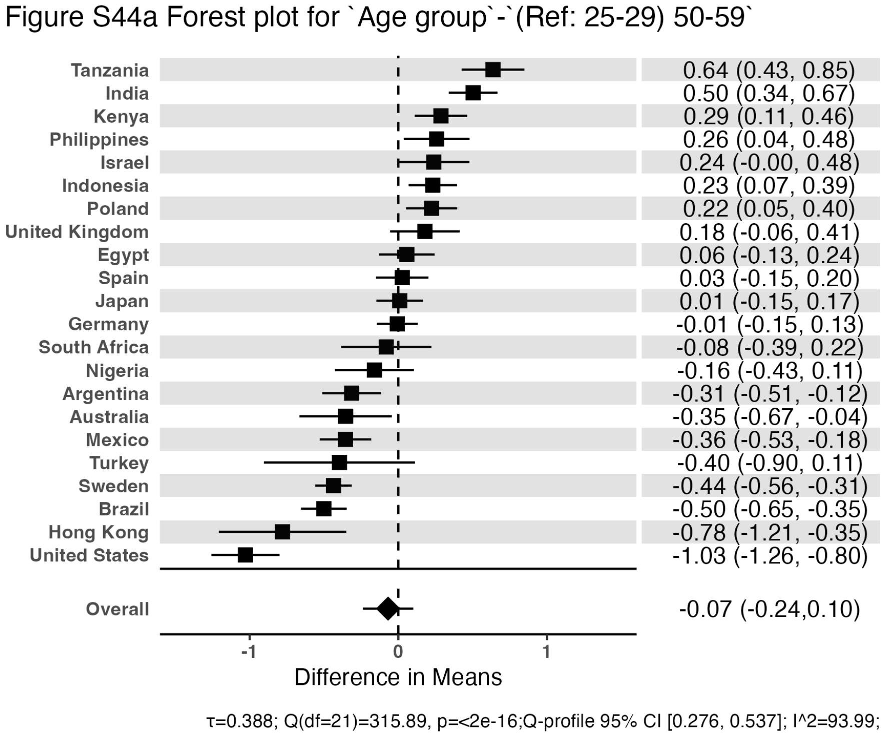

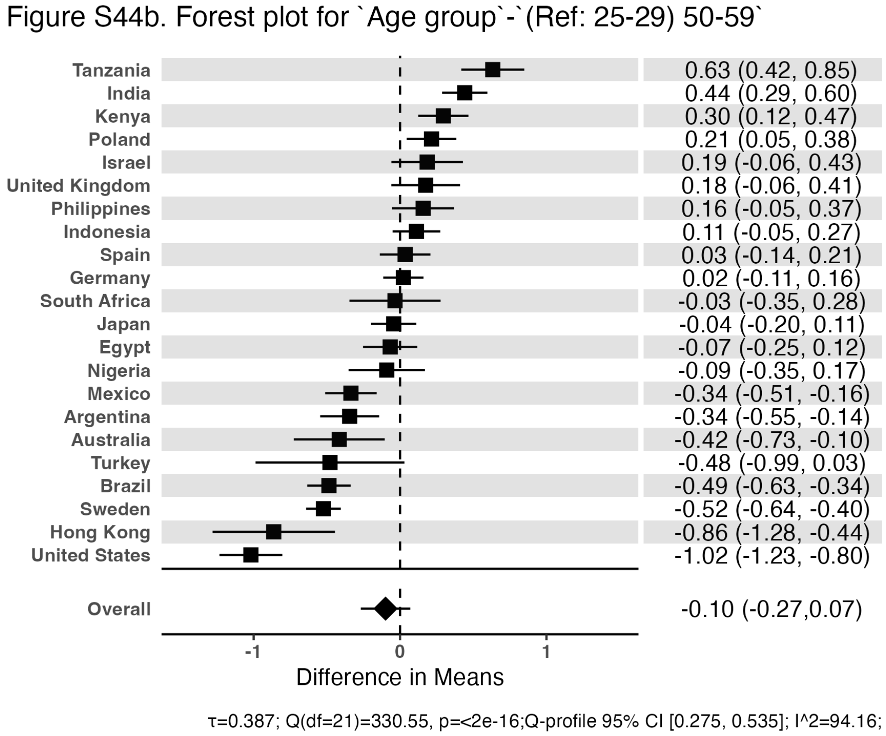

Figure S45. Heterogeneity in pairwise compairisons acrossx countries Age group-(Ref: 25-29) 60-69. (a) Flourishing with financial indicators (12 items) [left panel]; (b) Flourishing without financial indicators (10 items) [right panel]. N=202,898, subgroup means and standard errors are computed accounting for the complex sampling design using all data simultaneously. Analyses conducted: Random-effects meta-analysis of country-specific means. Squares represent the the point estimate (mean) for each country. The lines represented the +/-1.96*SE, standard error, around the mean; the overall pooled mean is represented by the diamond. The reported p-value for Q-statistics is necessarily 1-sided because of the use of the chi-squared distribution to test whether heterogeneity is greater than zero (i.e., a two-sided test is not applicable). No adjustments for multiple testing were made.

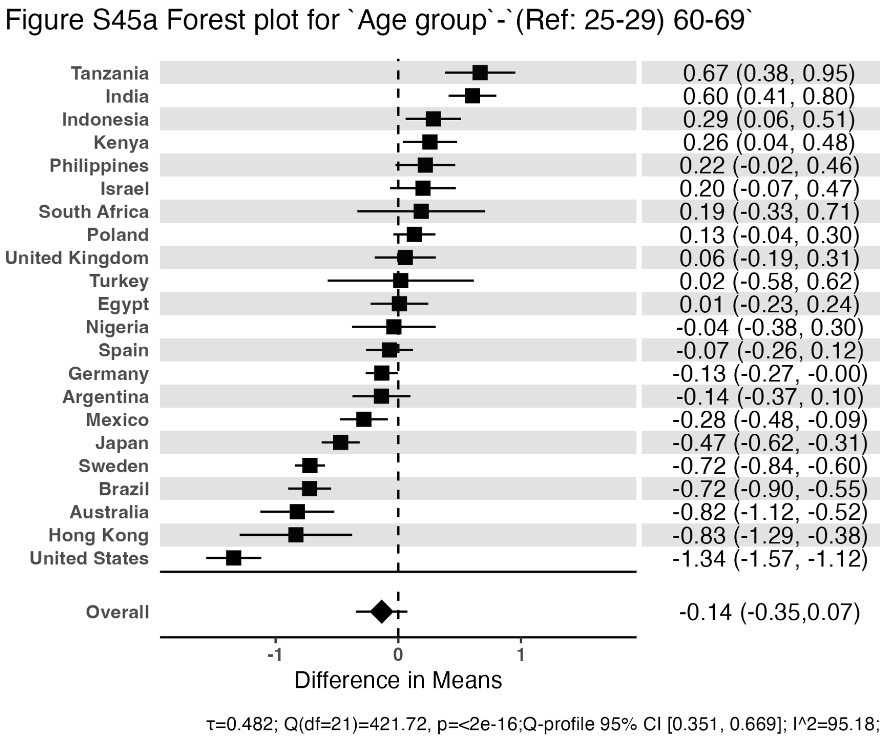

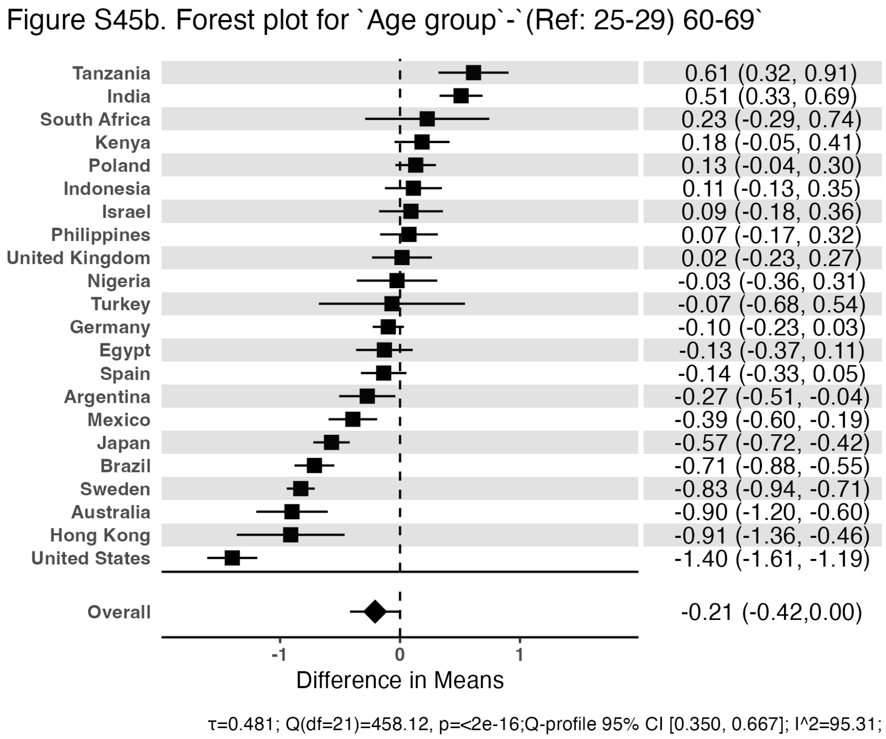

Figure S46. Heterogeneity in pairwise compairisons acrossx countries Age group-(Ref: 25-29) 70-79. (a) Flourishing with financial indicators (12 items) [left panel]; (b) Flourishing without financial indicators (10 items) [right panel]. N=202,898, subgroup means and standard errors are computed accounting for the complex sampling design using all data simultaneously. Analyses conducted: Random-effects meta-analysis of country-specific means. Squares represent the the point estimate (mean) for each country. The lines represented the +/-1.96*SE, standard error, around the mean; the overall pooled mean is represented by the diamond. The reported p-value for Q-statistics is necessarily 1-sided because of the use of the chi-squared distribution to test whether heterogeneity is greater than zero (i.e., a two-sided test is not applicable). No adjustments for multiple testing were made.

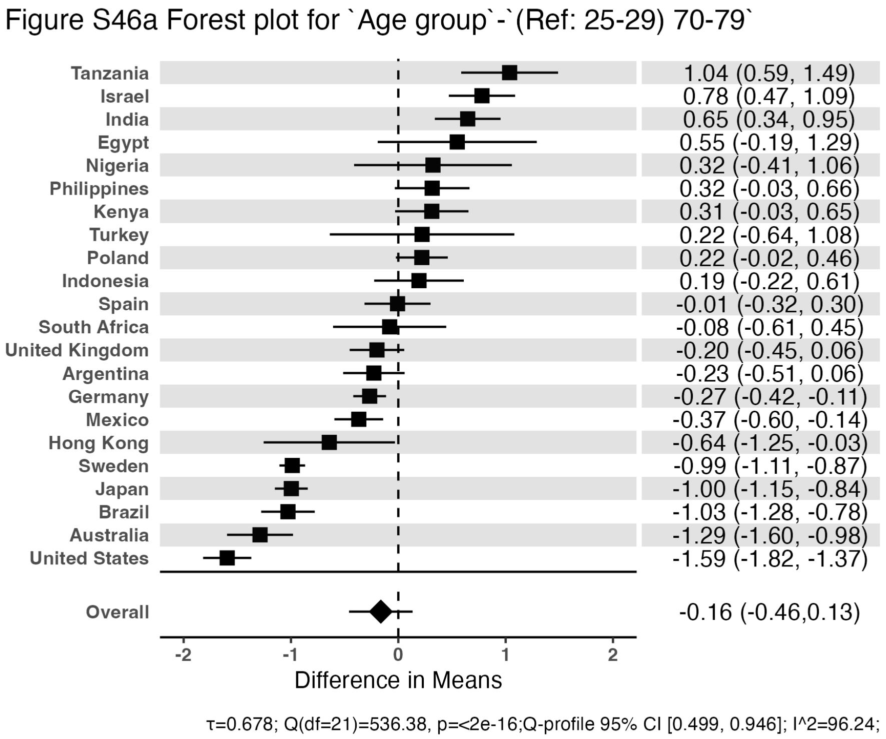

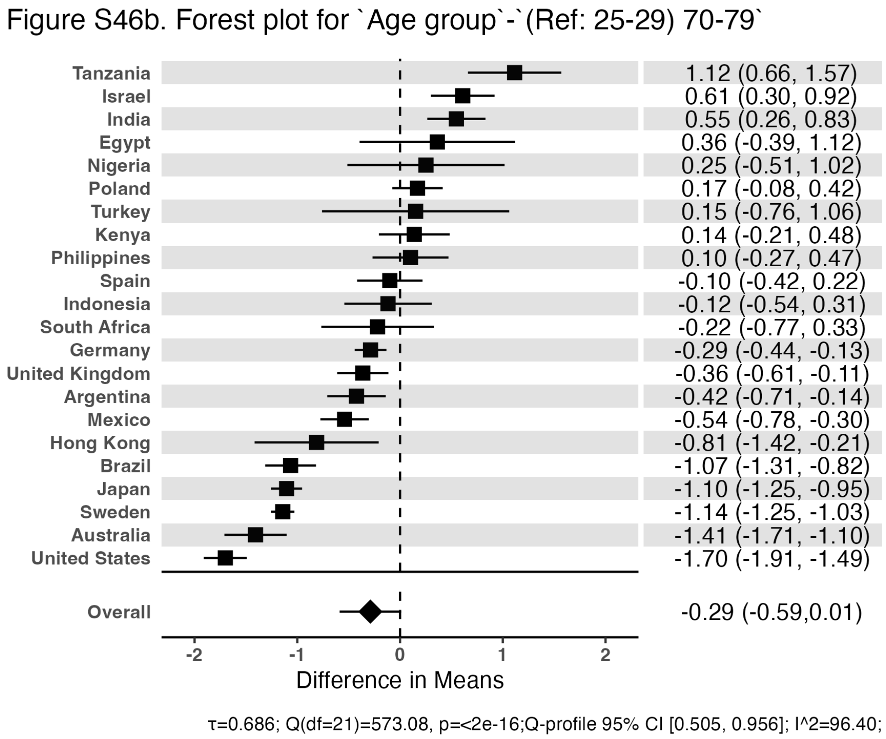

Figure S47. Heterogeneity in pairwise compairisons acrossx countries Age group-(Ref: 25-29) 80 or older. (a) Flourishing with financial indicators (12 items) [left panel]; (b) Flourishing without financial indicators (10 items) [right panel]. N=202,898, subgroup means and standard errors are computed accounting for the complex sampling design using all data simultaneously. Analyses conducted: Random-effects meta-analysis of country-specific means. Squares represent the the point estimate (mean) for each country. The lines represented the +/-1.96*SE, standard error, around the mean; the overall pooled mean is represented by the diamond. The reported p-value for Q-statistics is necessarily 1-sided because of the use of the chi-squared distribution to test whether heterogeneity is greater than zero (i.e., a two-sided test is not applicable). No adjustments for multiple testing were made.

Figure S47a Forest plot for `Age group`-`(Ref: 25-29) 80 or older`

Figure S47b. Forest plot for `Age group`-`(Ref: 25-29) 80 or older`

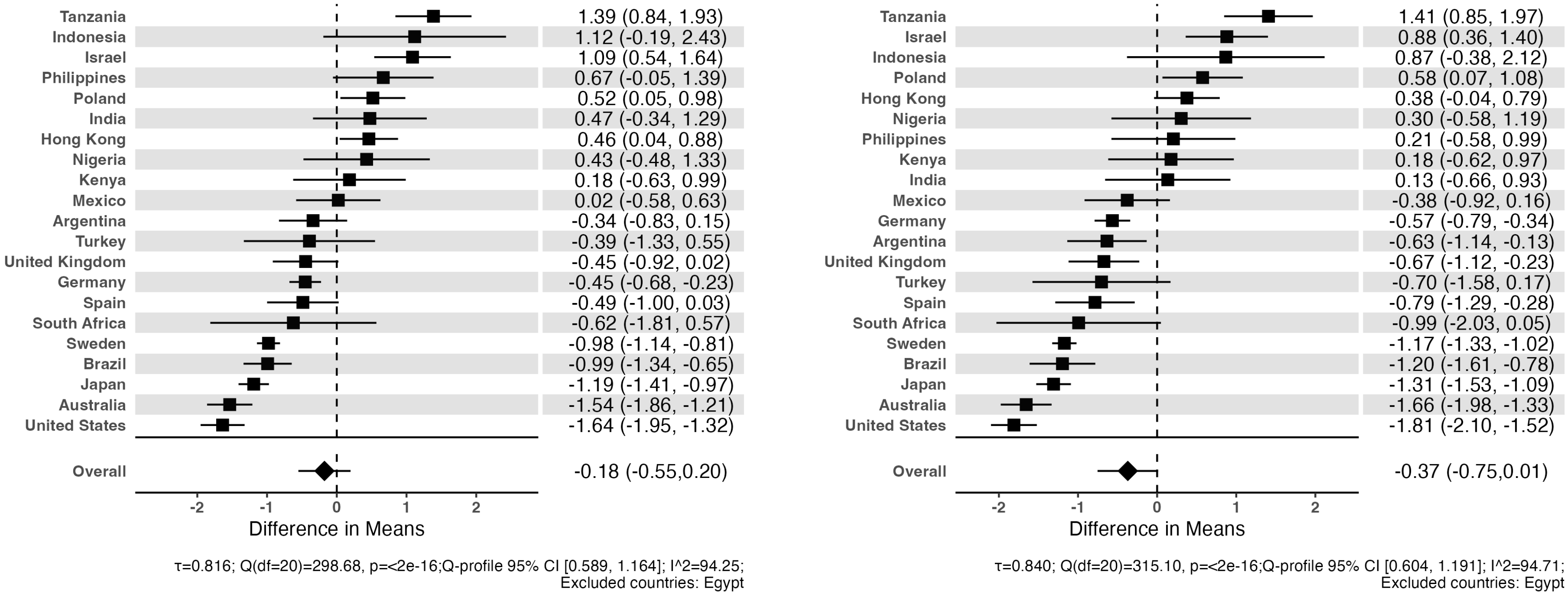

Figure S48. Heterogeneity in pairwise compairisons acrossx countries Age group-(Ref: 30-39) 40-49. (a) Flourishing with financial indicators (12 items) [left panel]; (b) Flourishing without financial indicators (10 items) [right panel]. N=202,898, subgroup means and standard errors are computed accounting for the complex sampling design using all data simultaneously. Analyses conducted: Random-effects meta-analysis of country-specific means. Squares represent the the point estimate (mean) for each country. The lines represented the +/-1.96*SE, standard error, around the mean; the overall pooled mean is represented by the diamond. The reported p-value for Q-statistics is necessarily 1-sided because of the use of the chi-squared distribution to test whether heterogeneity is greater than zero (i.e., a two-sided test is not applicable). No adjustments for multiple testing were made.

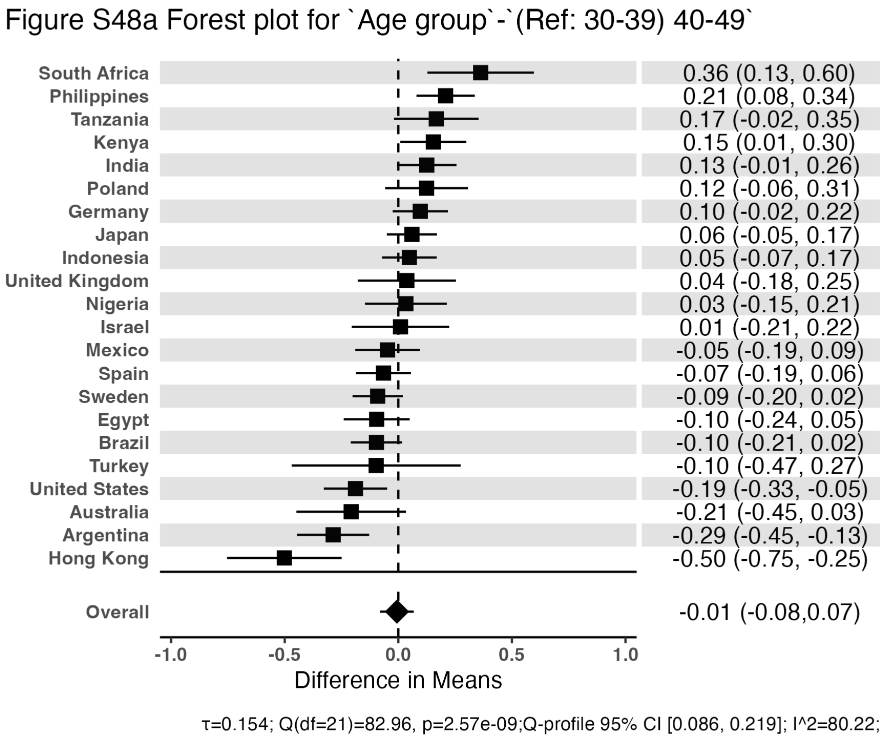

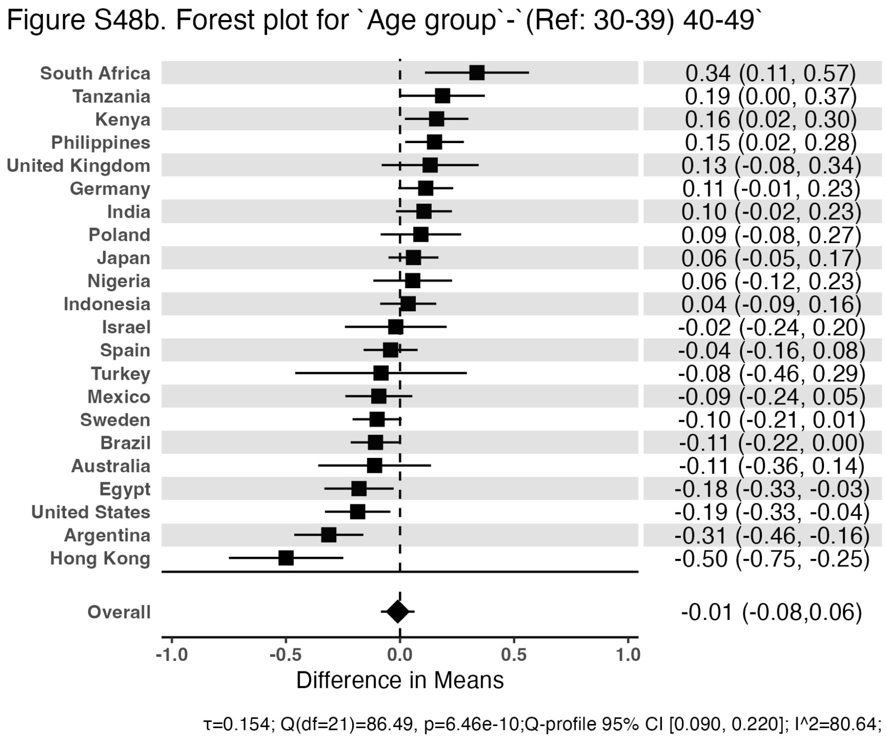

Figure S49. Heterogeneity in pairwise compairisons acrossx countries Age group-(Ref: 30-39) 50-59. (a) Flourishing with financial indicators (12 items) [left panel]; (b) Flourishing without financial indicators (10 items) [right panel]. N=202,898, subgroup means and standard errors are computed accounting for the complex sampling design using all data simultaneously. Analyses conducted: Random-effects meta-analysis of country-specific means. Squares represent the the point estimate (mean) for each country. The lines represented the +/-1.96*SE, standard error, around the mean; the overall pooled mean is represented by the diamond. The reported p-value for Q-statistics is necessarily 1-sided because of the use of the chi-squared distribution to test whether heterogeneity is greater than zero (i.e., a two-sided test is not applicable). No adjustments for multiple testing were made.

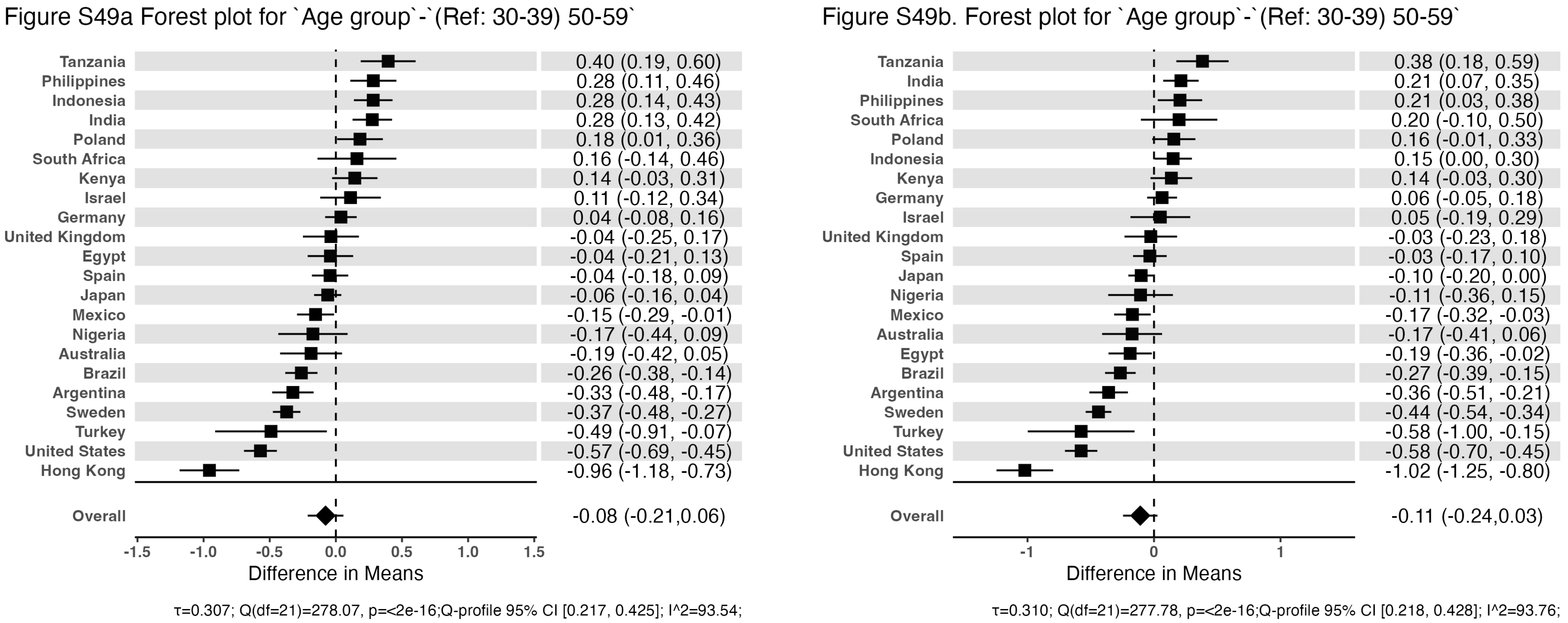

Figure S50. Heterogeneity in pairwise compairisons acrossx countries Age group-(Ref: 30-39) 60-69. (a) Flourishing with financial indicators (12 items) [left panel]; (b) Flourishing without financial indicators (10 items) [right panel]. N=202,898, subgroup means and standard errors are computed accounting for the complex sampling design using all data simultaneously. Analyses conducted: Random-effects meta-analysis of country-specific means. Squares represent the the point estimate (mean) for each country. The lines represented the +/-1.96*SE, standard error, around the mean; the overall pooled mean is represented by the diamond. The reported p-value for Q-statistics is necessarily 1-sided because of the use of the chi-squared distribution to test whether heterogeneity is greater than zero (i.e., a two-sided test is not applicable). No adjustments for multiple testing were made.

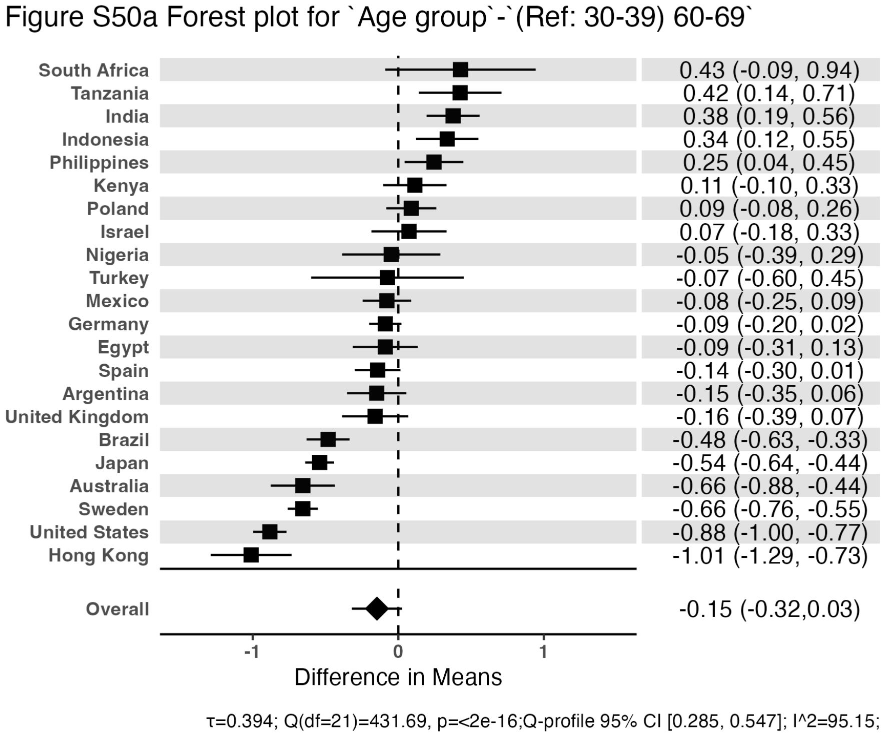

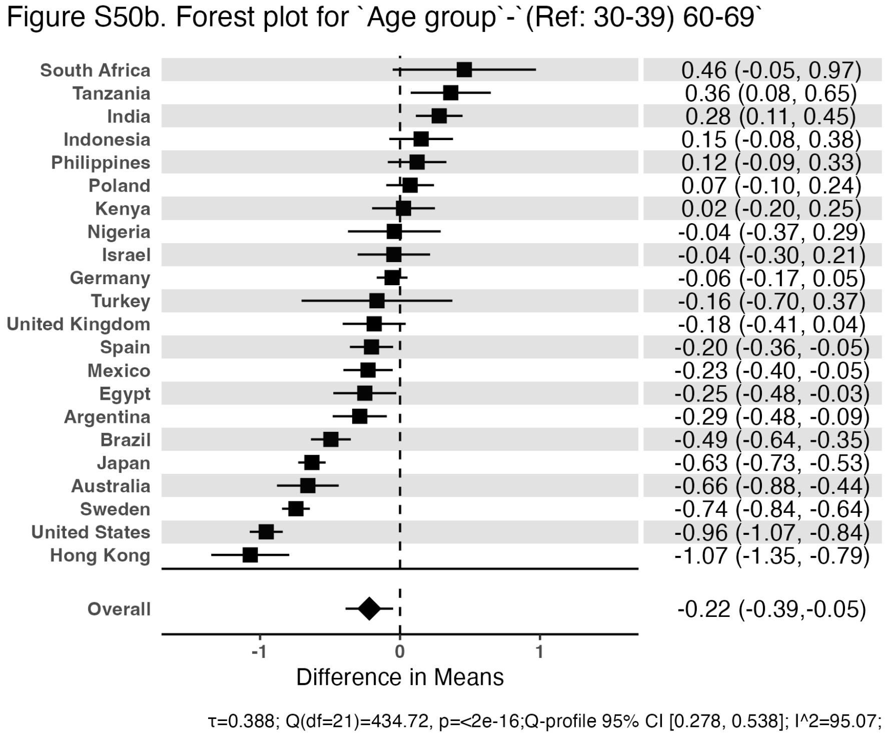

Figure S51. Heterogeneity in pairwise compairisons acrossx countries Age group-(Ref: 30-39) 70-79. (a) Flourishing with financial indicators (12 items) [left panel]; (b) Flourishing without financial indicators (10 items) [right panel]. N=202,898, subgroup means and standard errors are computed accounting for the complex sampling design using all data simultaneously. Analyses conducted: Random-effects meta-analysis of country-specific means. Squares represent the the point estimate (mean) for each country. The lines represented the +/-1.96*SE, standard error, around the mean; the overall pooled mean is represented by the diamond. The reported p-value for Q-statistics is necessarily 1-sided because of the use of the chi-squared distribution to test whether heterogeneity is greater than zero (i.e., a two-sided test is not applicable). No adjustments for multiple testing were made.

Figure S51a Forest plot for `Age group`-`(Ref: 30-39) 70-79`

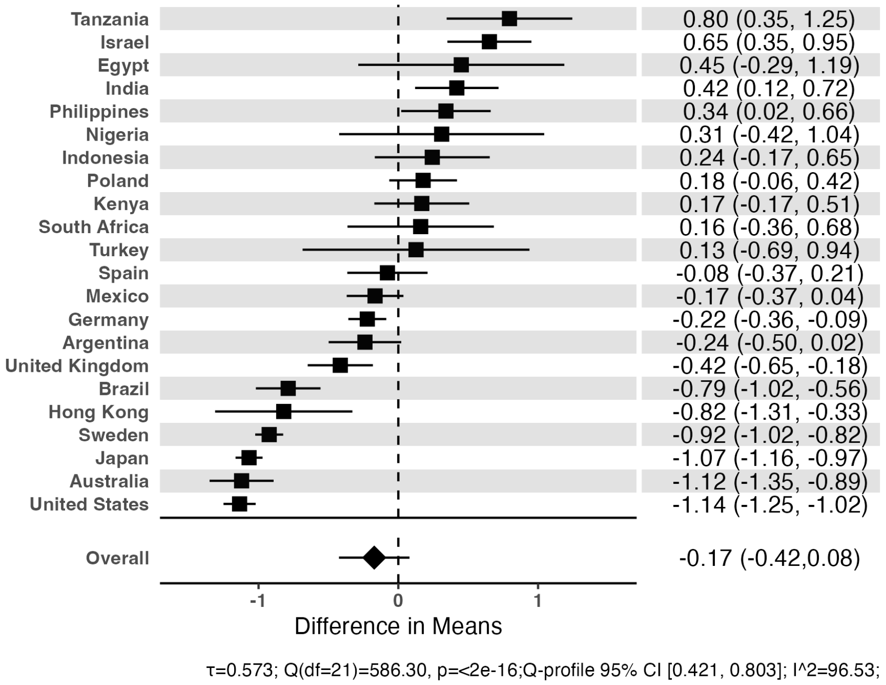

Figure S51b. Forest plot for `Age group`-`(Ref: 30-39) 70-79`

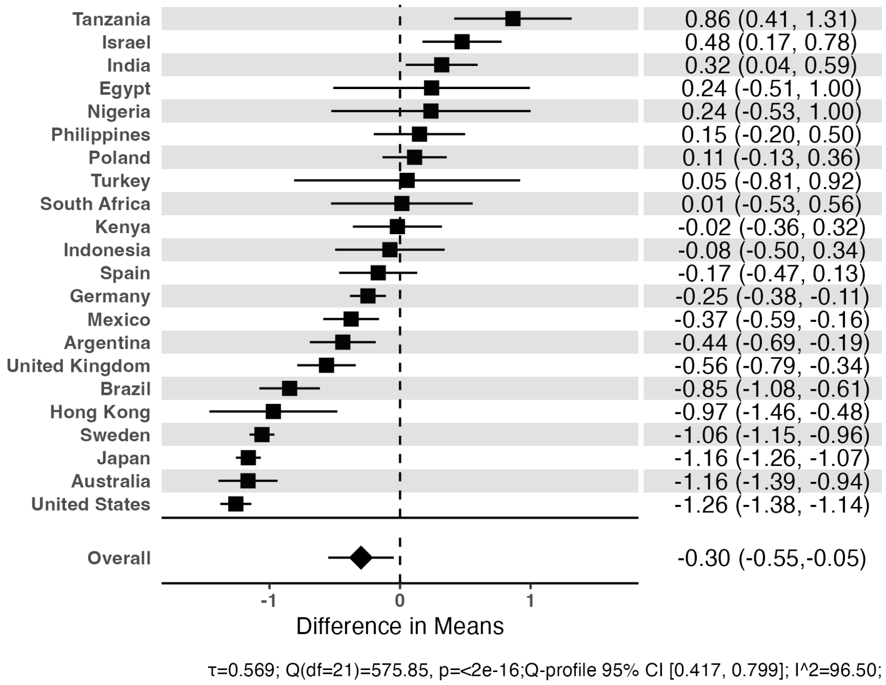

Figure S52. Heterogeneity in pairwise compairisons acrossx countries Age group-(Ref: 30-39) 80 or older. (a) Flourishing with financial indicators (12 items) [left panel]; (b) Flourishing without financial indicators (10 items) [right panel]. N=202,898, subgroup means and standard errors are computed accounting for the complex sampling design using all data simultaneously. Analyses conducted: Random-effects meta-analysis of country-specific means. Squares represent the the point estimate (mean) for each country. The lines represented the +/-1.96*SE, standard error, around the mean; the overall pooled mean is represented by the diamond. The reported p-value for Q-statistics is necessarily 1-sided because of the use of the chi-squared distribution to test whether heterogeneity is greater than zero (i.e., a two-sided test is not applicable). No adjustments for multiple testing were made.

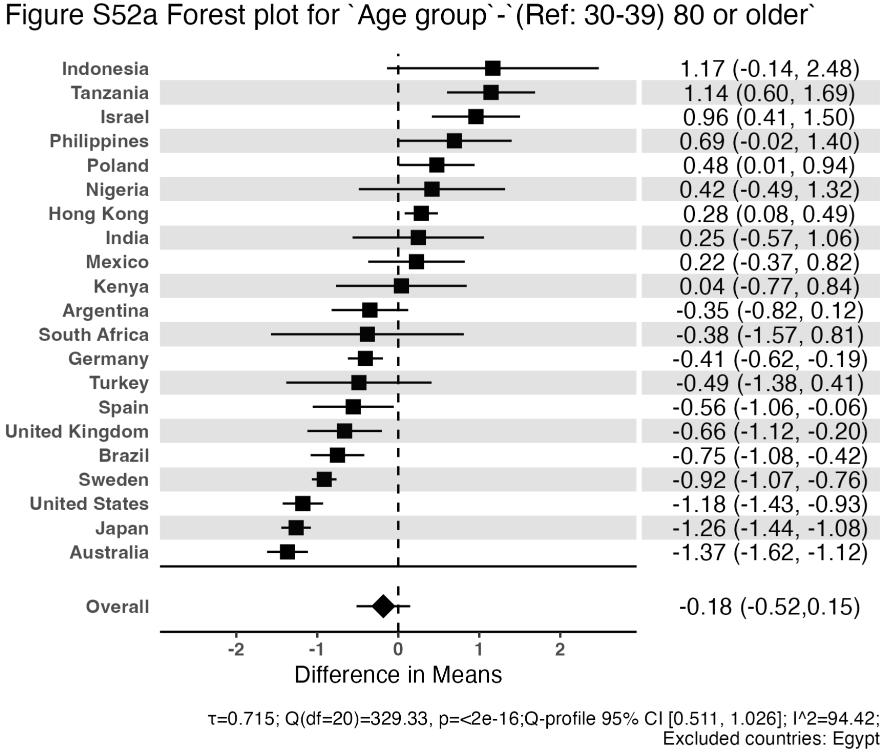

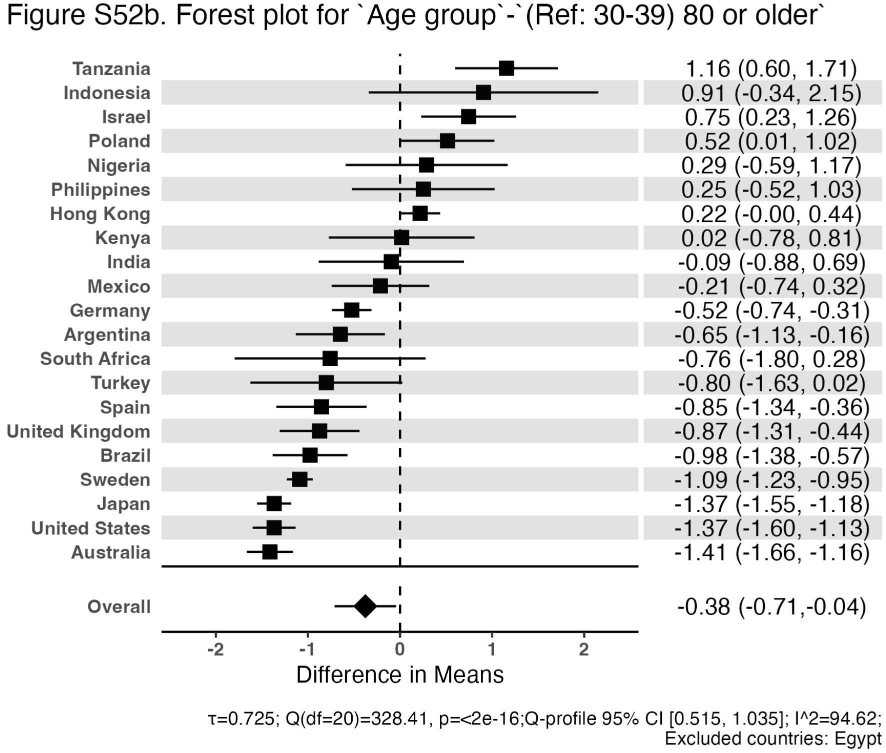

Figure S53. Heterogeneity in pairwise compairisons acrossx countries Age group-(Ref: 40-49) 50-59. (a) Flourishing with financial indicators (12 items) [left panel]; (b) Flourishing without financial indicators (10 items) [right panel]. N=202,898, subgroup means and standard errors are computed accounting for the complex sampling design using all data simultaneously. Analyses conducted: Random-effects meta-analysis of country-specific means. Squares represent the the point estimate (mean) for each country. The lines represented the +/-1.96*SE, standard error, around the mean; the overall pooled mean is represented by the diamond. The reported p-value for Q-statistics is necessarily 1-sided because of the use of the chi-squared distribution to test whether heterogeneity is greater than zero (i.e., a two-sided test is not applicable). No adjustments for multiple testing were made.

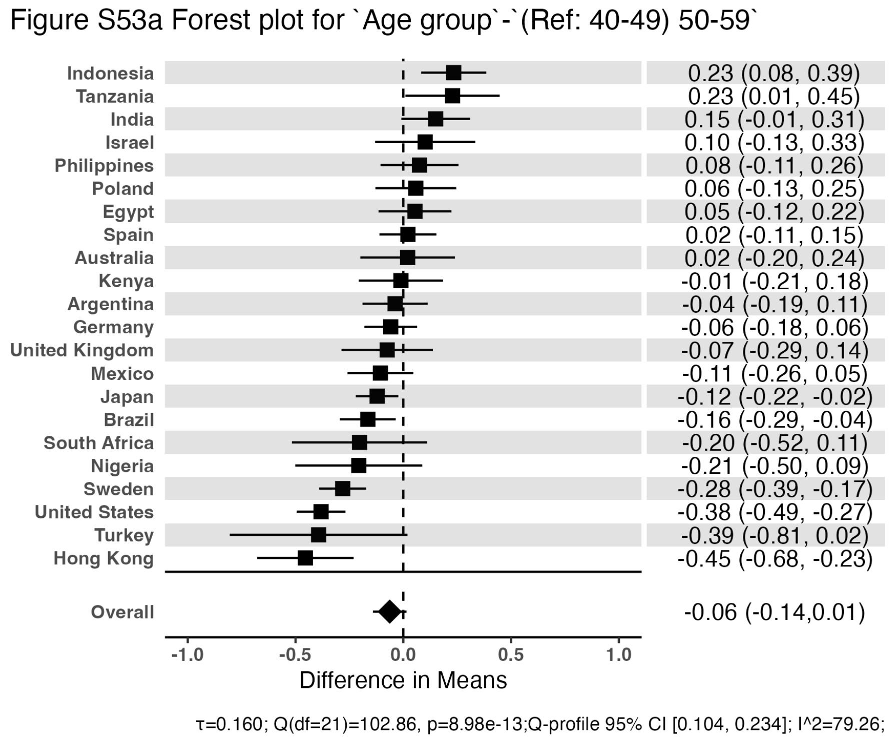

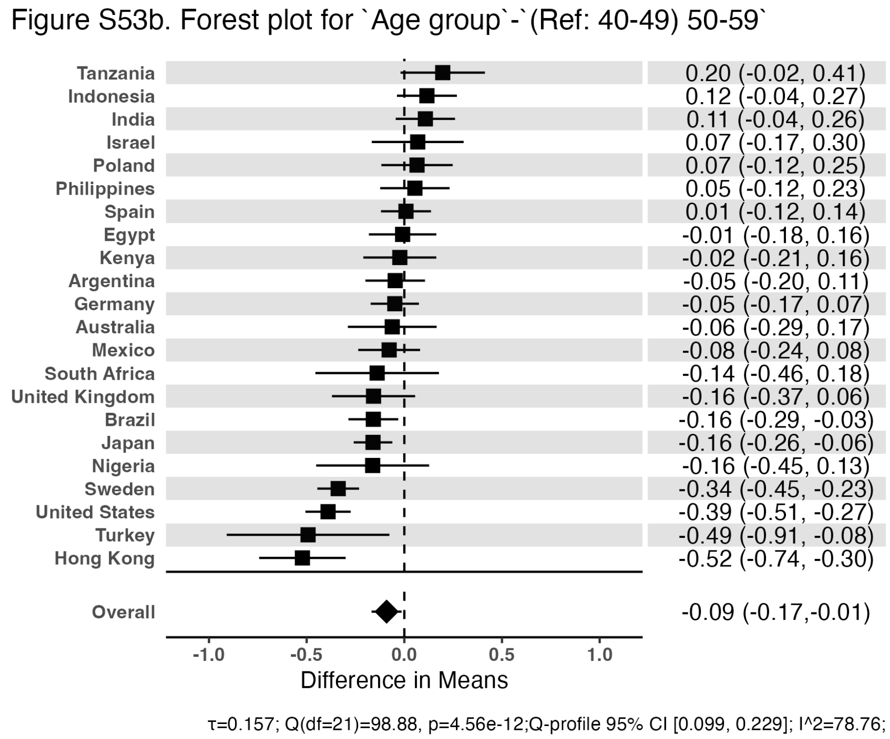

Figure S54. Heterogeneity in pairwise compairisons acrossx countries Age group-(Ref: 40-49) 60-69. (a) Flourishing with financial indicators (12 items) [left panel]; (b) Flourishing without financial indicators (10 items) [right panel]. N=202,898, subgroup means and standard errors are computed accounting for the complex sampling design using all data simultaneously. Analyses conducted: Random-effects meta-analysis of country-specific means. Squares represent the the point estimate (mean) for each country. The lines represented the +/-1.96*SE, standard error, around the mean; the overall pooled mean is represented by the diamond. The reported p-value for Q-statistics is necessarily 1-sided because of the use of the chi-squared distribution to test whether heterogeneity is greater than zero (i.e., a two-sided test is not applicable). No adjustments for multiple testing were made.

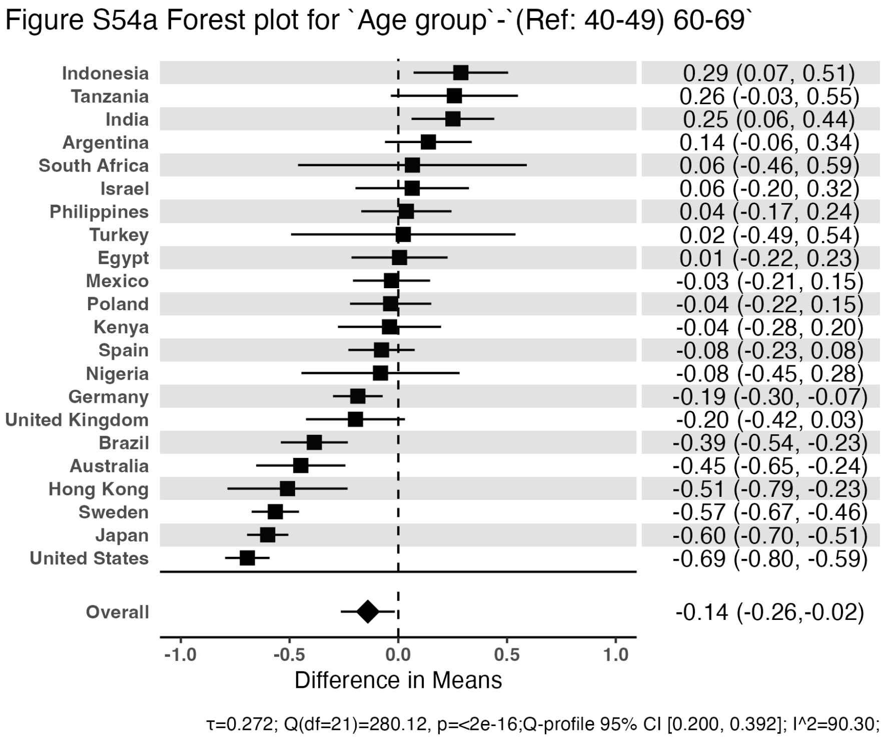

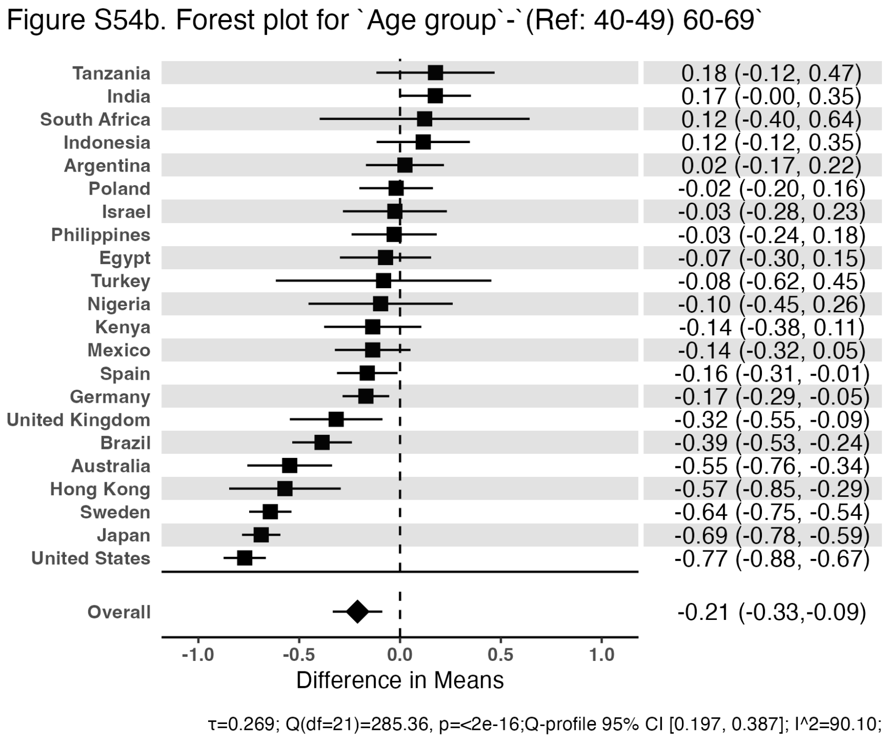

Figure S55. Heterogeneity in pairwise compairisons acrossx countries Age group-(Ref: 40-49) 70-79. (a) Flourishing with financial indicators (12 items) [left panel]; (b) Flourishing without financial indicators (10 items) [right panel]. N=202,898, subgroup means and standard errors are computed accounting for the complex sampling design using all data simultaneously. Analyses conducted: Random-effects meta-analysis of country-specific means. Squares represent the the point estimate (mean) for each country. The lines represented the +/-1.96*SE, standard error, around the mean; the overall pooled mean is represented by the diamond. The reported p-value for Q-statistics is necessarily 1-sided because of the use of the chi-squared distribution to test whether heterogeneity is greater than zero (i.e., a two-sided test is not applicable). No adjustments for multiple testing were made.

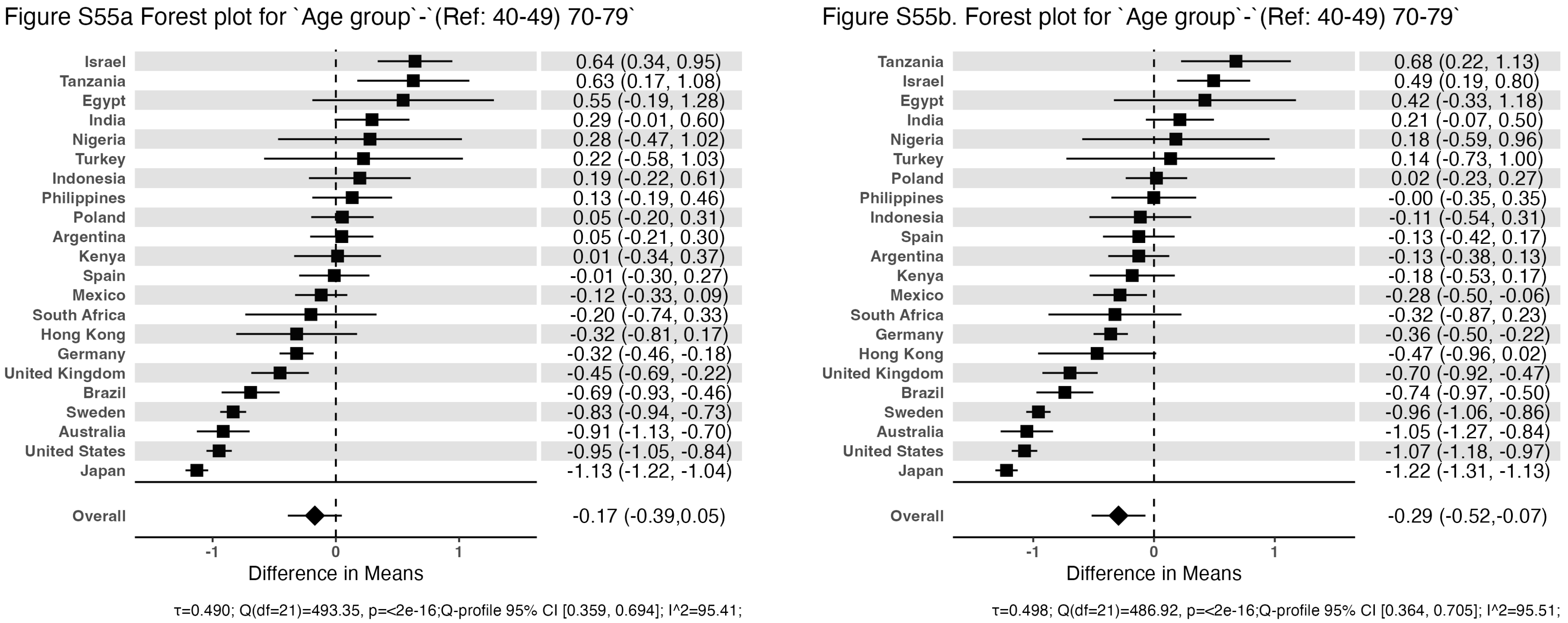

Figure S56. Heterogeneity in pairwise compairisons acrossx countries Age group-(Ref: 40-49) 80 or older. (a) Flourishing with financial indicators (12 items) [left panel]; (b) Flourishing without financial indicators (10 items) [right panel]. N=202,898, subgroup means and standard errors are computed accounting for the complex sampling design using all data simultaneously. Analyses conducted: Random-effects meta-analysis of country-specific means. Squares represent the the point estimate (mean) for each country. The lines represented the +/-1.96*SE, standard error, around the mean; the overall pooled mean is represented by the diamond. The reported p-value for Q-statistics is necessarily 1-sided because of the use of the chi-squared distribution to test whether heterogeneity is greater than zero (i.e., a two-sided test is not applicable). No adjustments for multiple testing were made.

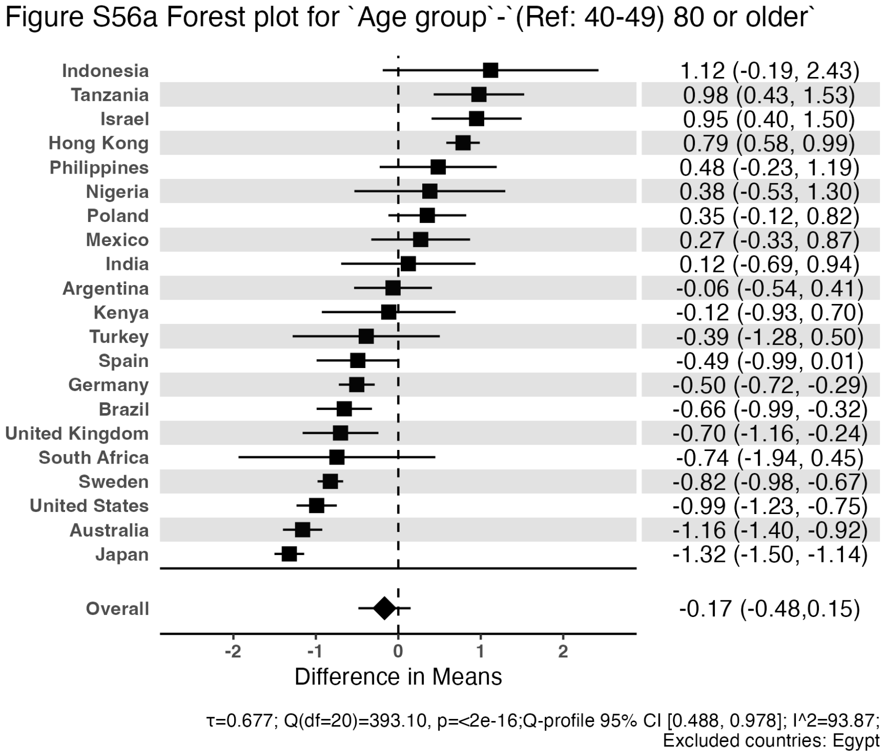

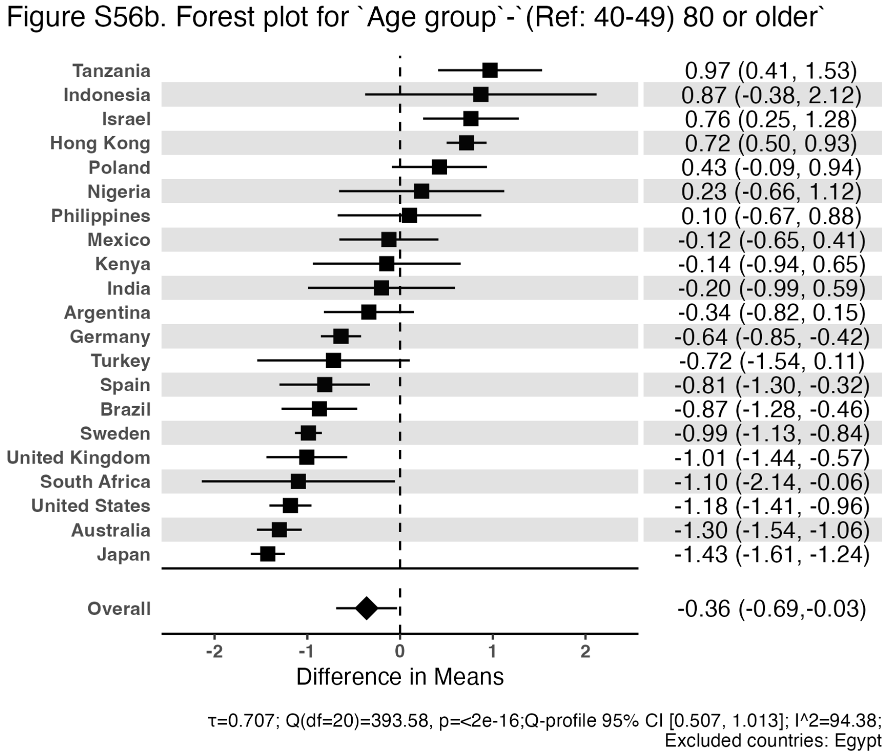

Figure S57. Heterogeneity in pairwise compairisons acrossx countries Age group-(Ref: 50-59) 60-69. (a) Flourishing with financial indicators (12 items) [left panel]; (b) Flourishing without financial indicators (10 items) [right panel]. N=202,898, subgroup means and standard errors are computed accounting for the complex sampling design using all data simultaneously. Analyses conducted: Random-effects meta-analysis of country-specific means. Squares represent the the point estimate (mean) for each country. The lines represented the +/-1.96*SE, standard error, around the mean; the overall pooled mean is represented by the diamond. The reported p-value for Q-statistics is necessarily 1-sided because of the use of the chi-squared distribution to test whether heterogeneity is greater than zero (i.e., a two-sided test is not applicable). No adjustments for multiple testing were made.

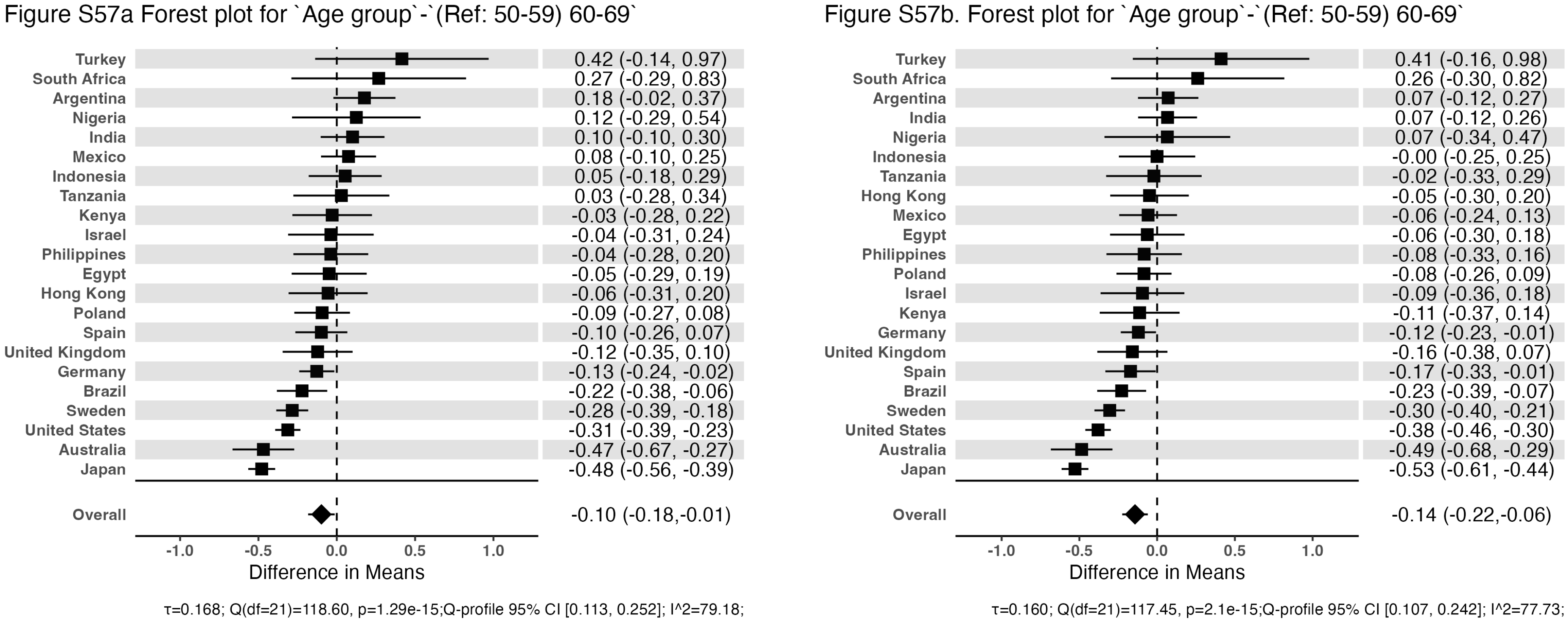

Figure S58. Heterogeneity in pairwise compairisons acrossx countries Age group-(Ref: 50-59) 70-79. (a) Flourishing with financial indicators (12 items) [left panel]; (b) Flourishing without financial indicators (10 items) [right panel]. N=202,898, subgroup means and standard errors are computed accounting for the complex sampling design using all data simultaneously. Analyses conducted: Random-effects meta-analysis of country-specific means. Squares represent the the point estimate (mean) for each country. The lines represented the +/-1.96*SE, standard error, around the mean; the overall pooled mean is represented by the diamond. The reported p-value for Q-statistics is necessarily 1-sided because of the use of the chi-squared distribution to test whether heterogeneity is greater than zero (i.e., a two-sided test is not applicable). No adjustments for multiple testing were made.

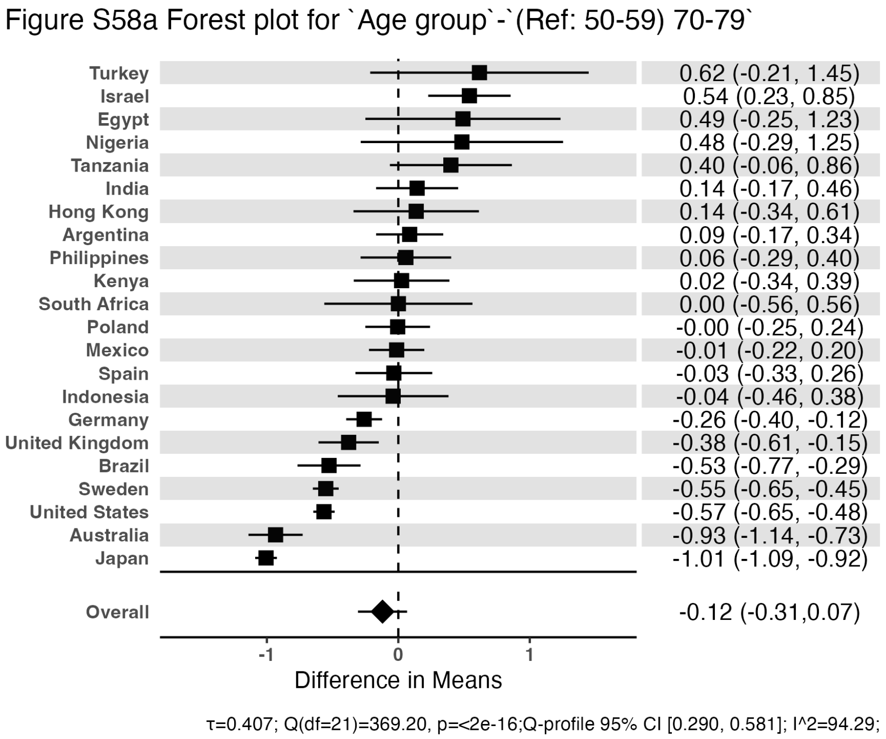

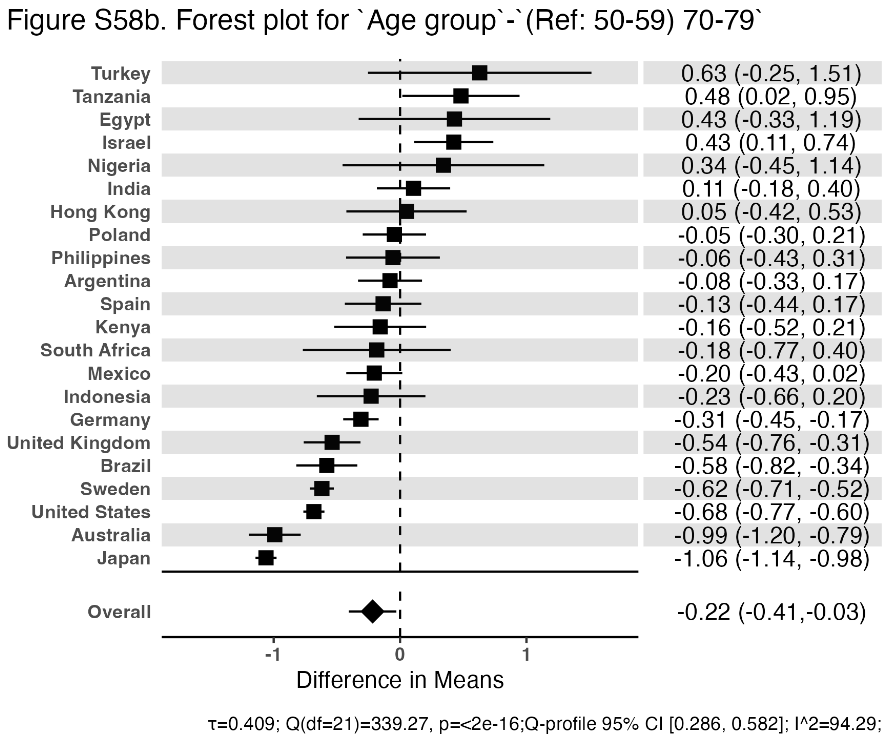

Figure S59. Heterogeneity in pairwise compairisons acrossx countries Age group-(Ref: 50-59) 80 or older. (a) Flourishing with financial indicators (12 items) [left panel]; (b) Flourishing without financial indicators (10 items) [right panel]. N=202,898, subgroup means and standard errors are computed accounting for the complex sampling design using all data simultaneously. Analyses conducted: Random-effects meta-analysis of country-specific means. Squares represent the the point estimate (mean) for each country. The lines represented the +/-1.96*SE, standard error, around the mean; the overall pooled mean is represented by the diamond. The reported p-value for Q-statistics is necessarily 1-sided because of the use of the chi-squared distribution to test whether heterogeneity is greater than zero (i.e., a two-sided test is not applicable). No adjustments for multiple testing were made.

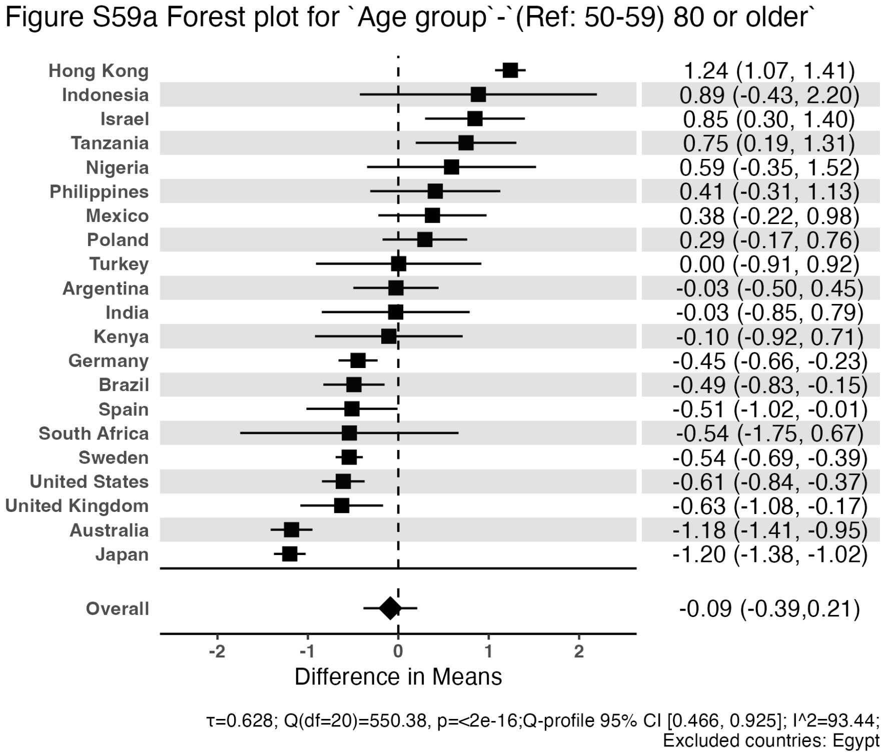

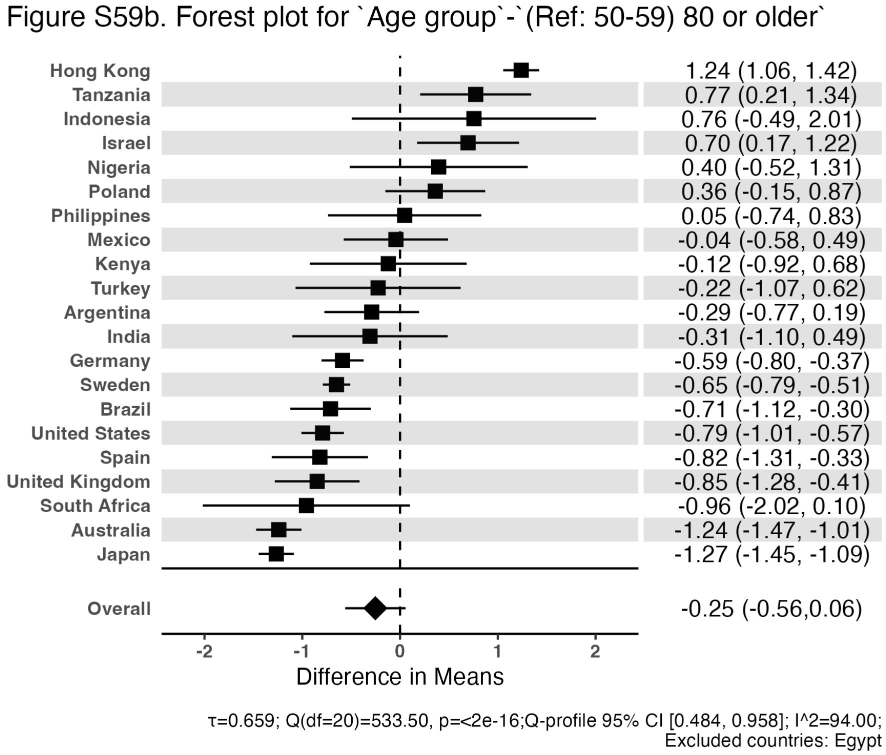

Figure S60. Heterogeneity in pairwise compairisons acrossx countries Age group-(Ref: 60-69) 70-79. (a) Flourishing with financial indicators (12 items) [left panel]; (b) Flourishing without financial indicators (10 items) [right panel]. N=202,898, subgroup means and standard errors are computed accounting for the complex sampling design using all data simultaneously. Analyses conducted: Random-effects meta-analysis of country-specific means. Squares represent the the point estimate (mean) for each country. The lines represented the +/-1.96*SE, standard error, around the mean; the overall pooled mean is represented by the diamond. The reported p-value for Q-statistics is necessarily 1-sided because of the use of the chi-squared distribution to test whether heterogeneity is greater than zero (i.e., a two-sided test is not applicable). No adjustments for multiple testing were made.

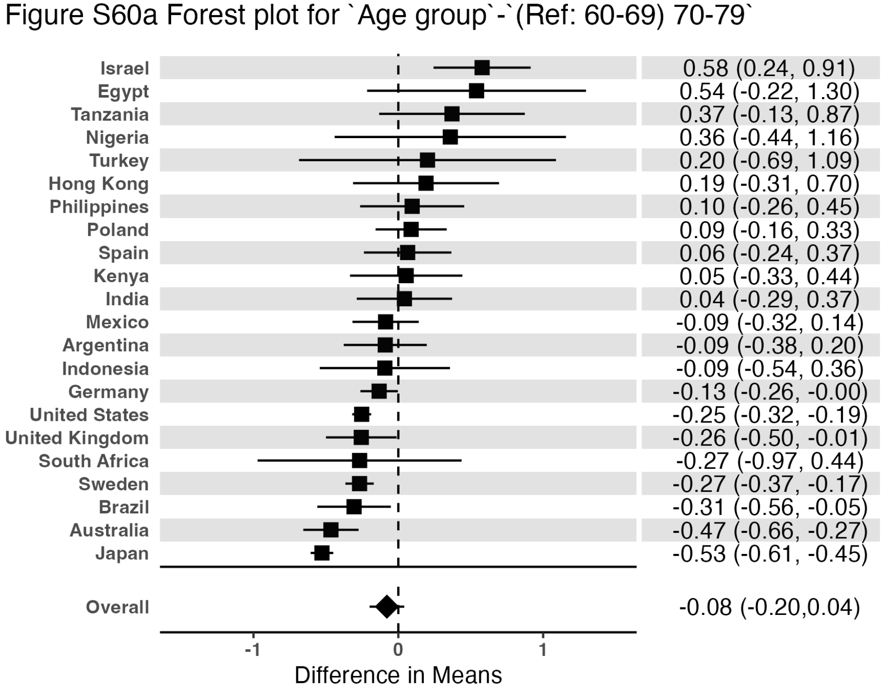

τ=0.228; Q(df=21)=119.77, p=7.87e-16;Q-profile 95% CI [0.148, 0.355]; I^2=85.07;

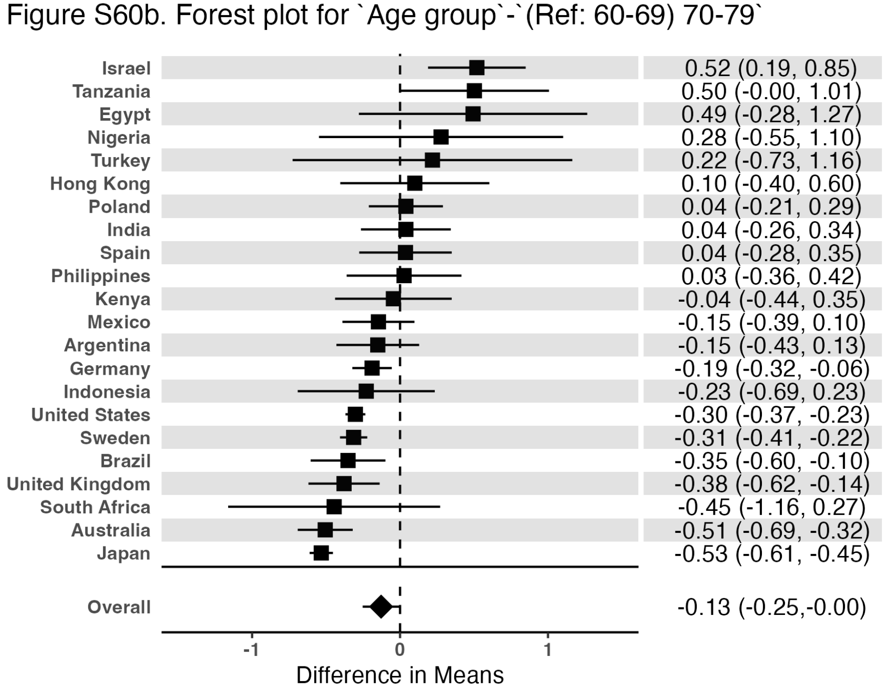

τ=0.240; Q(df=21)=107.71, p=1.22e-13;Q-profile 95% CI [0.148, 0.366]; I^2=86.28;

Figure S61. Heterogeneity in pairwise compairisons acrossx countries Age group-(Ref: 60-69) 80 or older. (a) Flourishing with financial indicators (12 items) [left panel]; (b) Flourishing without financial indicators (10 items) [right panel]. N=202,898, subgroup means and standard errors are computed accounting for the complex sampling design using all data simultaneously. Analyses conducted: Random-effects meta-analysis of country-specific means. Squares represent the the point estimate (mean) for each country. The lines represented the +/-1.96*SE, standard error, around the mean; the overall pooled mean is represented by the diamond. The reported p-value for Q-statistics is necessarily 1-sided because of the use of the chi-squared distribution to test whether heterogeneity is greater than zero (i.e., a two-sided test is not applicable). No adjustments for multiple testing were made.

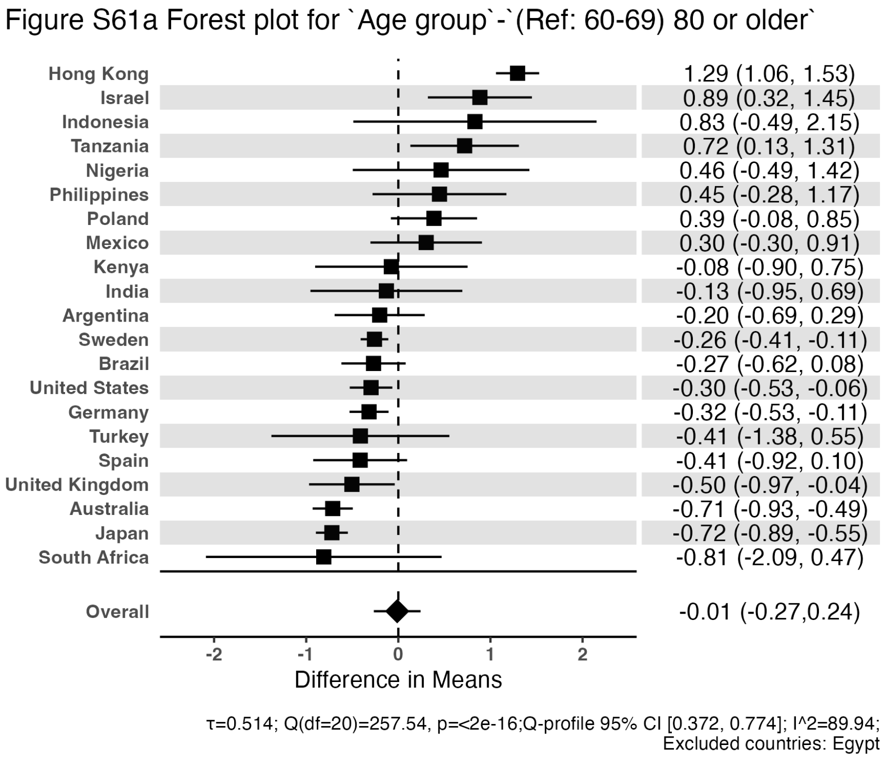

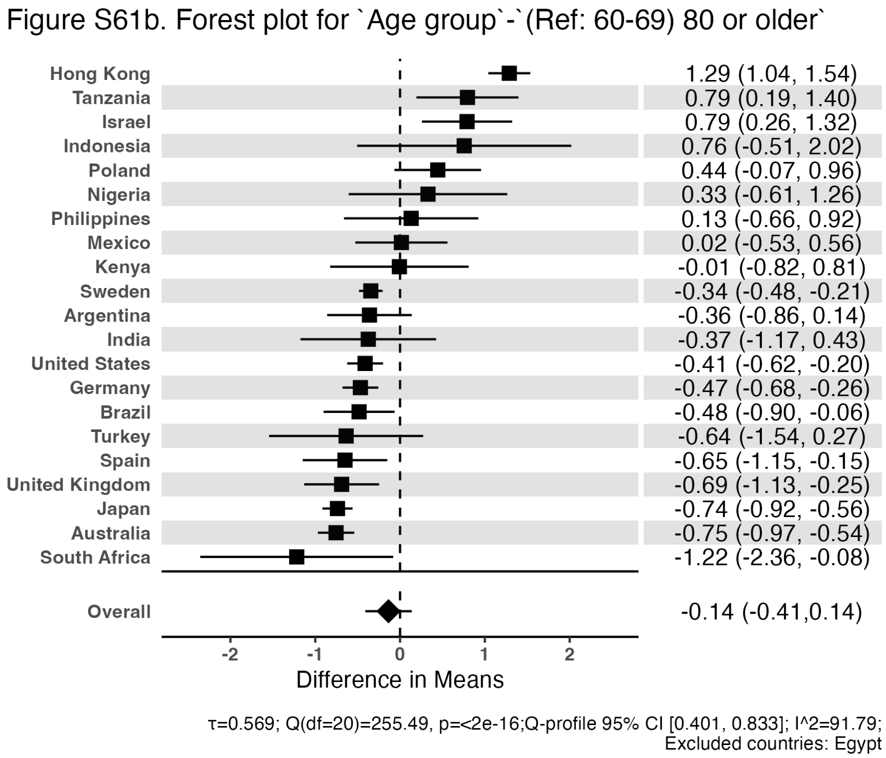

Figure S62. Heterogeneity in pairwise compairisons acrossx countries Age group-(Ref: 70-79) 80 or older. (a) Flourishing with financial indicators (12 items) [left panel]; (b) Flourishing without financial indicators (10 items) [right panel]. N=202,898, subgroup means and standard errors are computed accounting for the complex sampling design using all data simultaneously. Analyses conducted: Random-effects meta-analysis of country-specific means. Squares represent the the point estimate (mean) for each country. The lines represented the +/-1.96*SE, standard error, around the mean; the overall pooled mean is represented by the diamond. The reported p-value for Q-statistics is necessarily 1-sided because of the use of the chi-squared distribution to test whether heterogeneity is greater than zero (i.e., a two-sided test is not applicable). No adjustments for multiple testing were made.

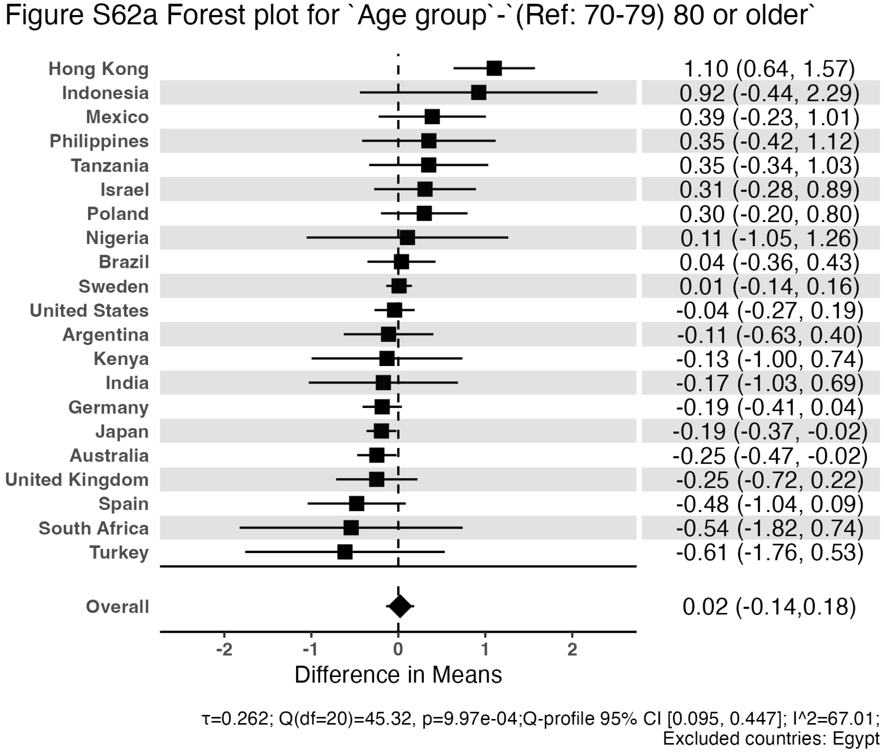

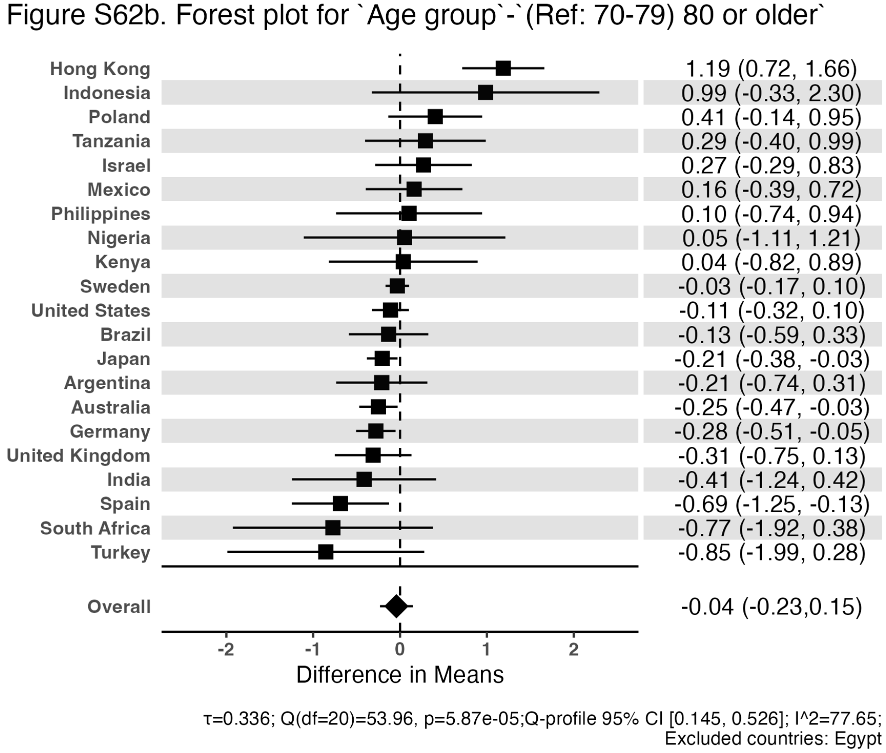

Figure S63. Heterogeneity in pairwise compairisons acrossx countries Gender-(Ref: Female) Male. (a) Flourishing with financial indicators (12 items) [left panel]; (b) Flourishing without financial indicators (10 items) [right panel]. N=202,898, subgroup means and standard errors are computed accounting for the complex sampling design using all data simultaneously. Analyses conducted: Random-effects meta-analysis of country-specific means. Squares represent the the point estimate (mean) for each country. The lines represented the +/-1.96*SE, standard error, around the mean; the overall pooled mean is represented by the diamond. The reported p-value for Q-statistics is necessarily 1-sided because of the use of the chi-squared distribution to test whether heterogeneity is greater than zero (i.e., a two-sided test is not applicable). No adjustments for multiple testing were made.

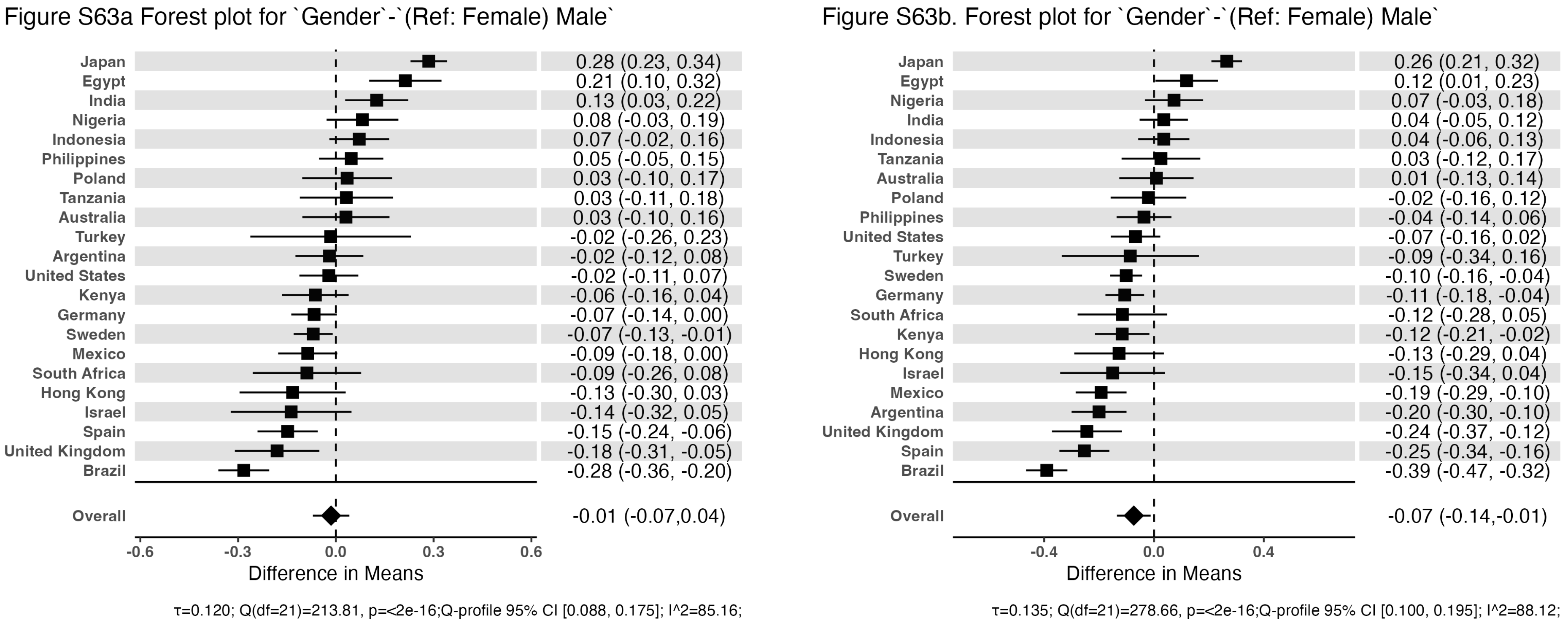

Figure S64. Heterogeneity in pairwise compairisons acrossx countries Gender-(Ref: Female) Other. (a) Flourishing with financial indicators (12 items) [left panel]; (b) Flourishing without financial indicators (10 items) [right panel]. N=202,898, subgroup means and standard errors are computed accounting for the complex sampling design using all data simultaneously. Analyses conducted: Random-effects meta-analysis of country-specific means. Squares represent the the point estimate (mean) for each country. The lines represented the +/-1.96*SE, standard error, around the mean; the overall pooled mean is represented by the diamond. The reported p-value for Q-statistics is necessarily 1-sided because of the use of the chi-squared distribution to test whether heterogeneity is greater than zero (i.e., a two-sided test is not applicable). No adjustments for multiple testing were made.

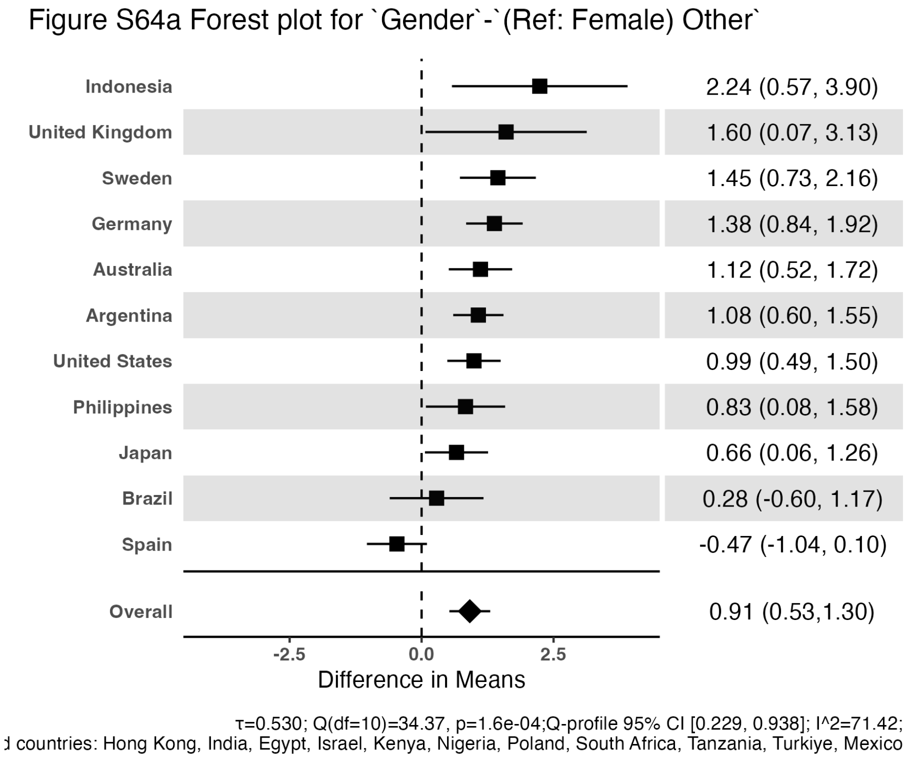

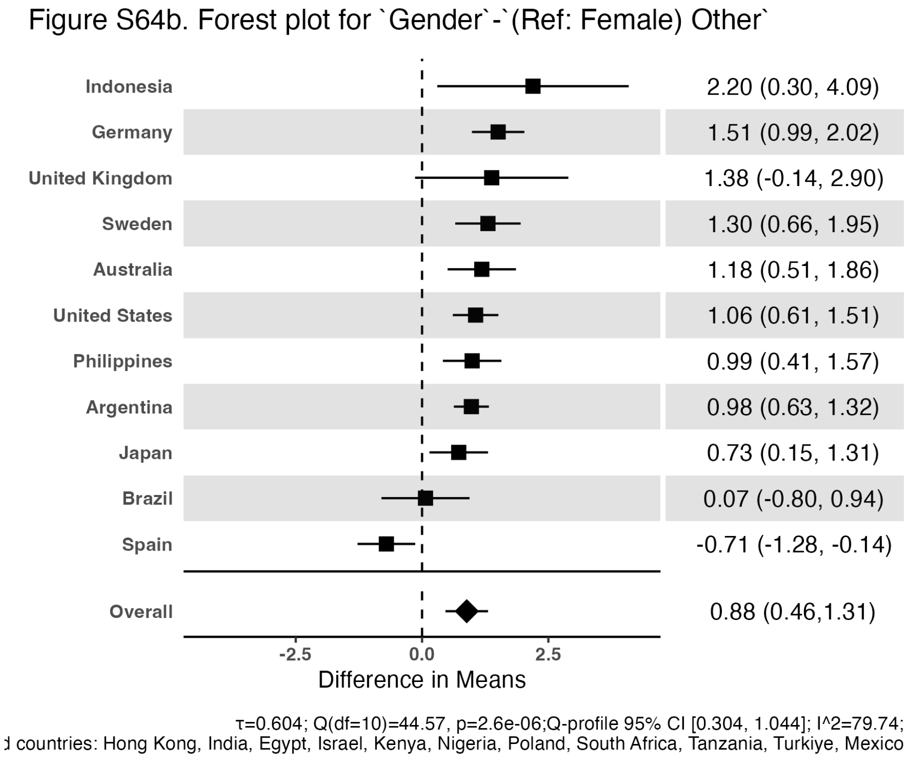

Figure S65. Heterogeneity in pairwise compairisons acrossx countries Gender-(Ref: Male) Other. (a) Flourishing with financial indicators (12 items) [left panel]; (b) Flourishing without financial indicators (10 items) [right panel]. N=202,898, subgroup means and standard errors are computed accounting for the complex sampling design using all data simultaneously. Analyses conducted: Random-effects meta-analysis of country-specific means. Squares represent the the point estimate (mean) for each country. The lines represented the +/-1.96*SE, standard error, around the mean; the overall pooled mean is represented by the diamond. The reported p-value for Q-statistics is necessarily 1-sided because of the use of the chi-squared distribution to test whether heterogeneity is greater than zero (i.e., a two-sided test is not applicable). No adjustments for multiple testing were made.

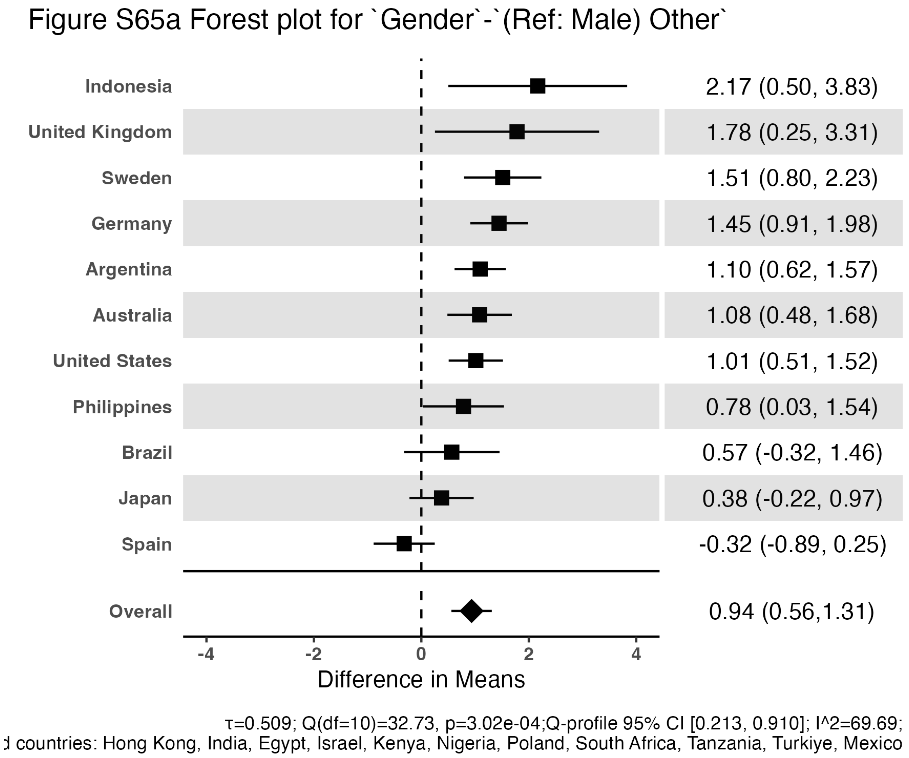

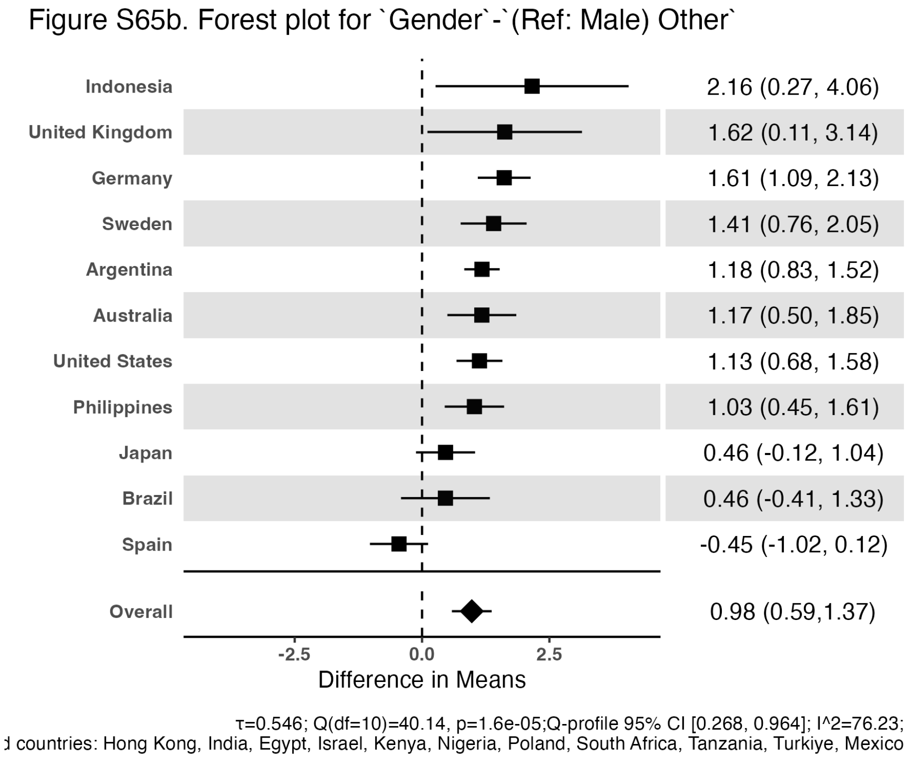

Figure S66. Heterogeneity in pairwise compairisons acrossx countries Marital status-(Ref: Divorced) Domestic partner. (a) Flourishing with financial indicators (12 items) [left panel]; (b) Flourishing without financial indicators (10 items) [right panel]. N=202,898, subgroup means and standard errors are computed accounting for the complex sampling design using all data simultaneously. Analyses conducted: Random-effects meta-analysis of country-specific means. Squares represent the the point estimate (mean) for each country. The lines represented the +/-1.96*SE, standard error, around the mean; the overall pooled mean is represented by the diamond. The reported p-value for Q-statistics is necessarily 1-sided because of the use of the chi-squared distribution to test whether heterogeneity is greater than zero (i.e., a two-sided test is not applicable). No adjustments for multiple testing were made.

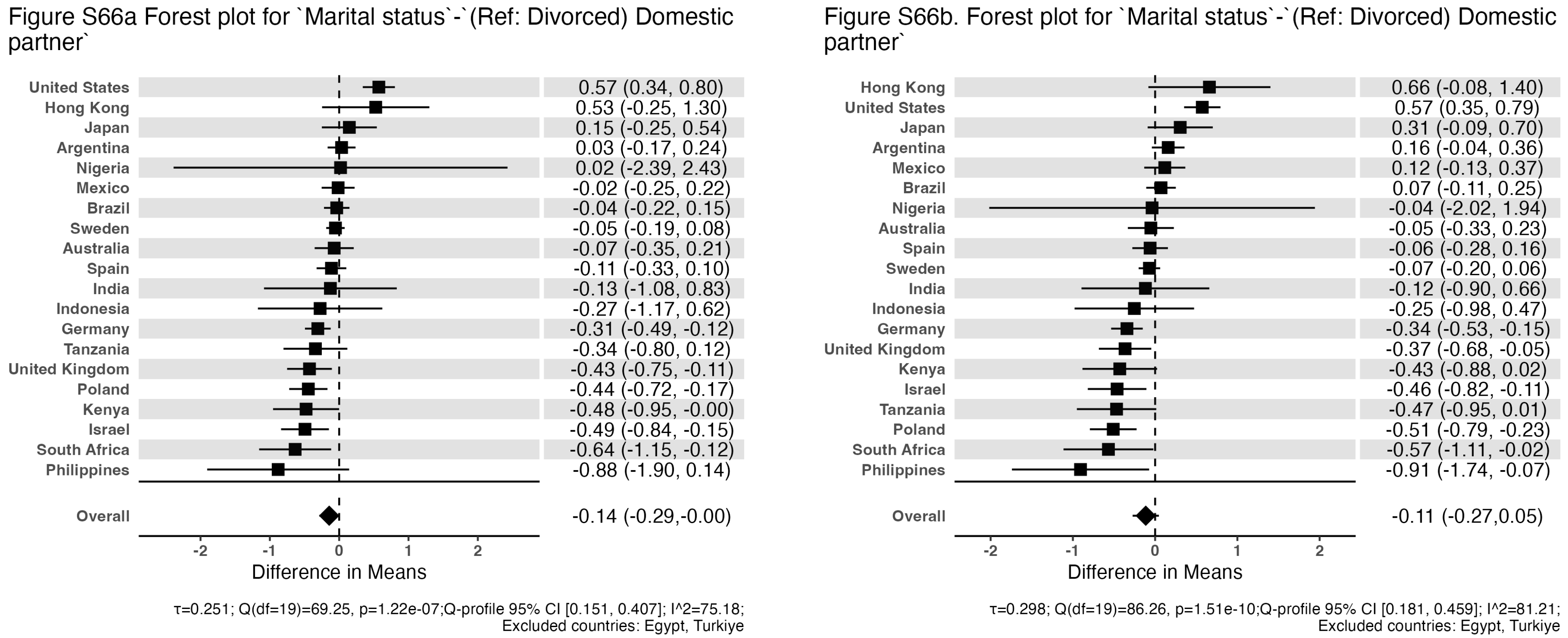

Figure S67. Heterogeneity in pairwise compairisons acrossx countries Marital status-(Ref: Divorced) Married. (a) Flourishing with financial indicators (12 items) [left panel]; (b) Flourishing without financial indicators (10 items) [right panel]. N=202,898, subgroup means and standard errors are computed accounting for the complex sampling design using all data simultaneously. Analyses conducted: Random-effects meta-analysis of country-specific means. Squares represent the the point estimate (mean) for each country. The lines represented the +/-1.96*SE, standard error, around the mean; the overall pooled mean is represented by the diamond. The reported p-value for Q-statistics is necessarily 1-sided because of the use of the chi-squared distribution to test whether heterogeneity is greater than zero (i.e., a two-sided test is not applicable). No adjustments for multiple testing were made.

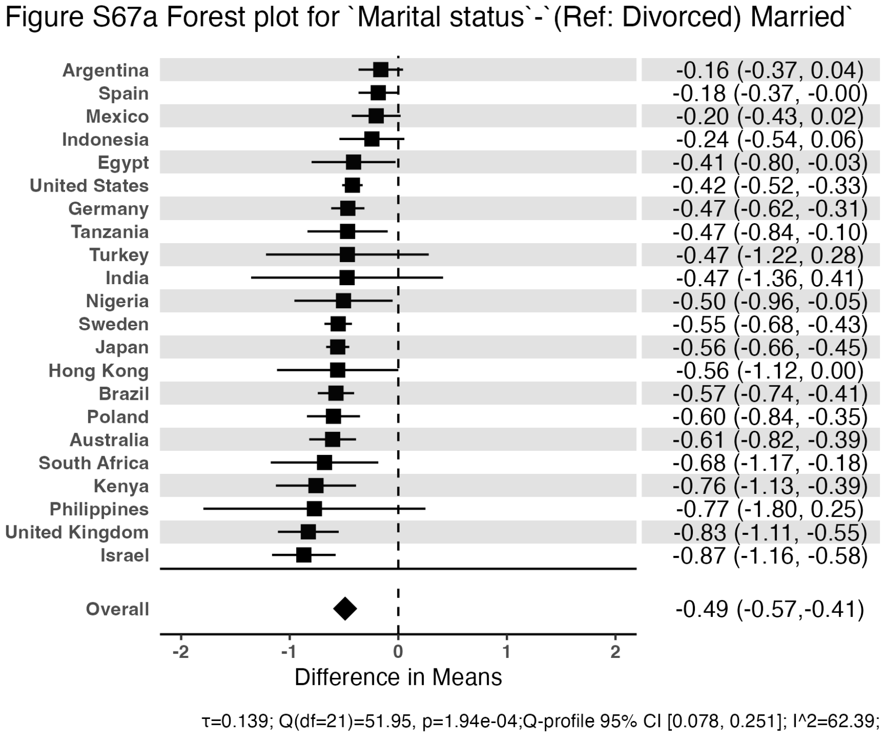

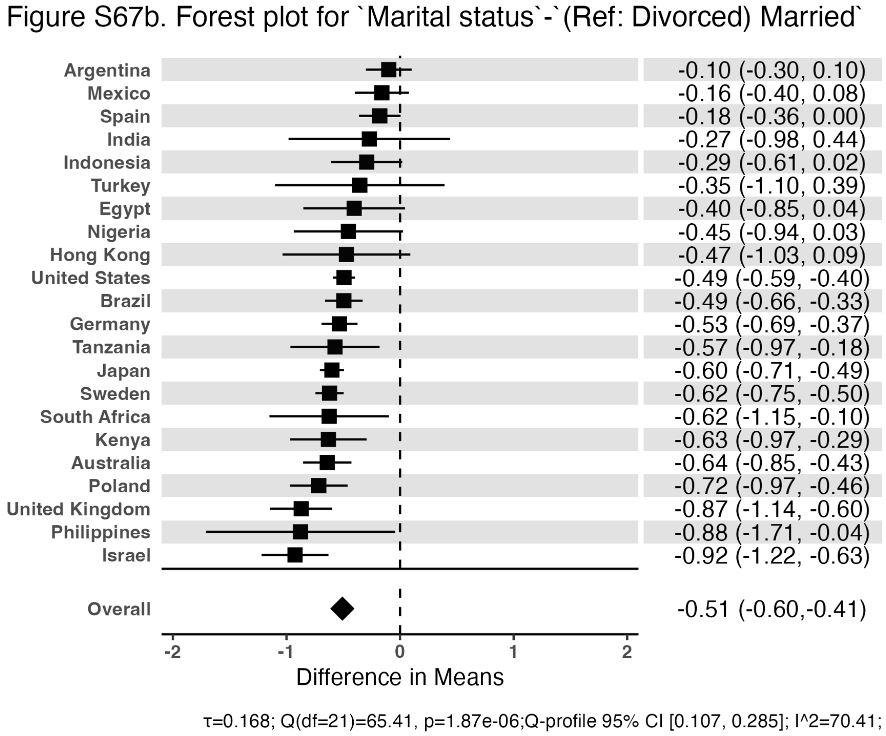

Figure S68. Heterogeneity in pairwise compairisons acrossx countries Marital status-(Ref: Divorced) Separated. (a) Flourishing with financial indicators (12 items) [left panel]; (b) Flourishing without financial indicators (10 items) [right panel]. N=202,898, subgroup means and standard errors are computed accounting for the complex sampling design using all data simultaneously. Analyses conducted: Random-effects meta-analysis of country-specific means. Squares represent the the point estimate (mean) for each country. The lines represented the +/-1.96*SE, standard error, around the mean; the overall pooled mean is represented by the diamond. The reported p-value for Q-statistics is necessarily 1-sided because of the use of the chi-squared distribution to test whether heterogeneity is greater than zero (i.e., a two-sided test is not applicable). No adjustments for multiple testing were made.

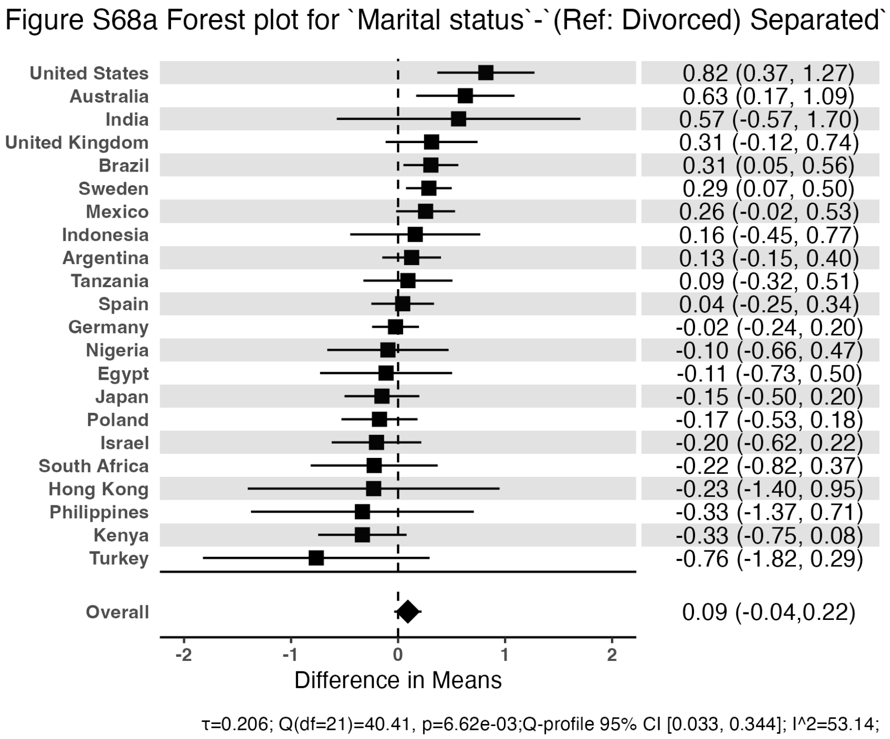

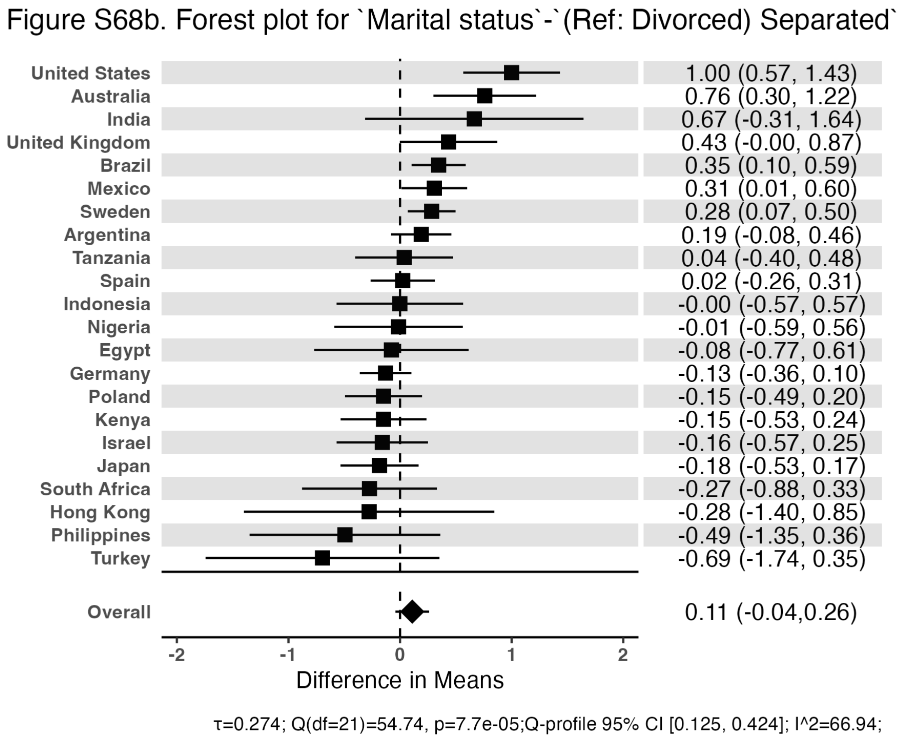

Figure S69. Heterogeneity in pairwise compairisons acrossx countries Marital status-(Ref: Divorced) Single, never married. (a) Flourishing with financial indicators (12 items) [left panel]; (b) Flourishing without financial indicators (10 items) [right panel]. N=202,898, subgroup means and standard errors are computed accounting for the complex sampling design using all data simultaneously. Analyses conducted: Random-effects meta-analysis of country-specific means. Squares represent the the point estimate (mean) for each country. The lines represented the +/-1.96*SE, standard error, around the mean; the overall pooled mean is represented by the diamond. The reported p-value for Q-statistics is necessarily 1-sided because of the use of the chi-squared distribution to test whether heterogeneity is greater than zero (i.e., a two-sided test is not applicable). No adjustments for multiple testing were made.

Figure S69a Forest plot for `Marital status`-`(Ref: Divorced) Single, neve married`

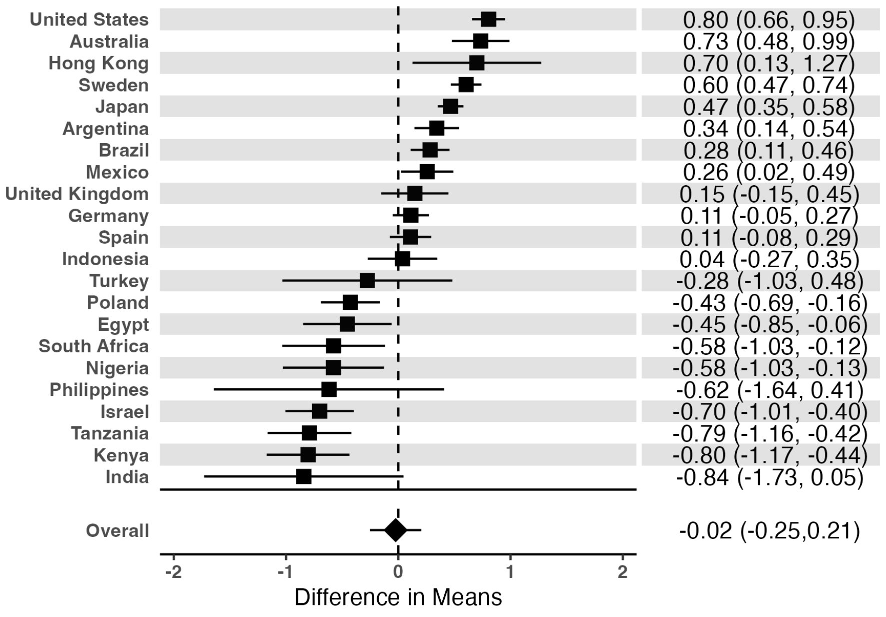

τ=0.510; Q(df=21)=289.41, p=<2e-16;Q-profile 95% CI [0.360, 0.717]; I^2=94.79;

Figure S69b. Forest plot for `Marital status`-`(Ref: Divorced) Single, never married`

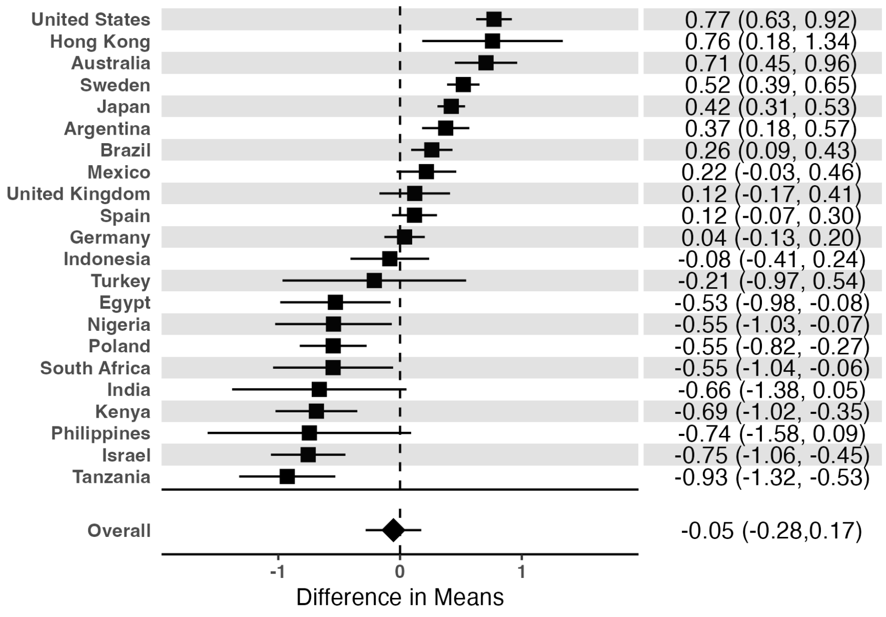

τ=0.511; Q(df=21)=286.02, p=<2e-16;Q-profile 95% CI [0.361, 0.717]; I^2=94.80;

Figure S70. Heterogeneity in pairwise compairisons acrossx countries Marital status-(Ref: Divorced) Widowed. (a) Flourishing with financial indicators (12 items) [left panel]; (b) Flourishing without financial indicators (10 items) [right panel]. N=202,898, subgroup means and standard errors are computed accounting for the complex sampling design using all data simultaneously. Analyses conducted: Random-effects meta-analysis of country-specific means. Squares represent the the point estimate (mean) for each country. The lines represented the +/-1.96*SE, standard error, around the mean; the overall pooled mean is represented by the diamond. The reported p-value for Q-statistics is necessarily 1-sided because of the use of the chi-squared distribution to test whether heterogeneity is greater than zero (i.e., a two-sided test is not applicable). No adjustments for multiple testing were made.

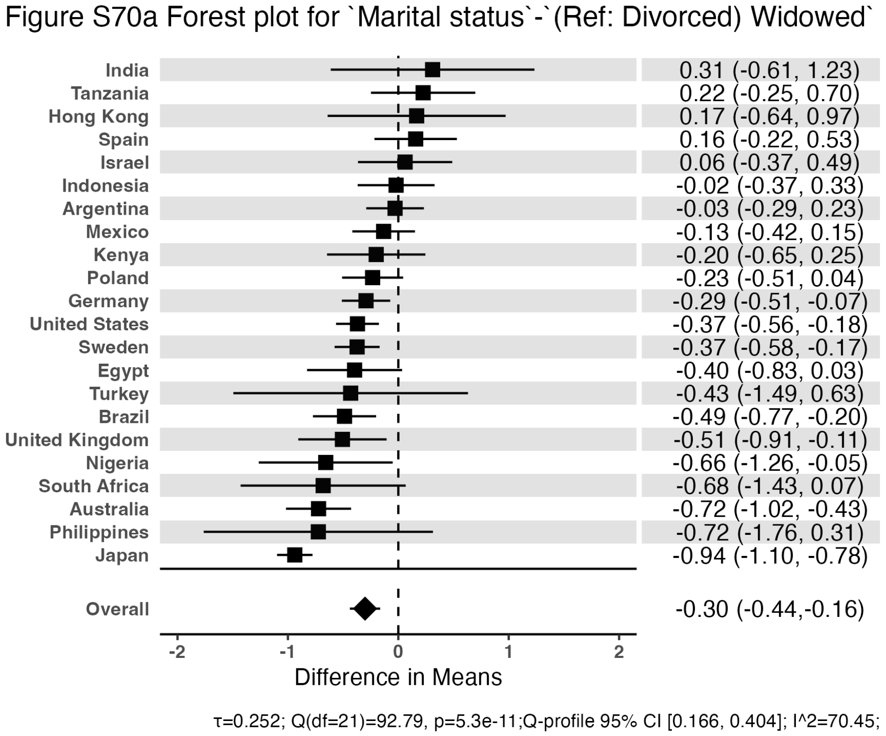

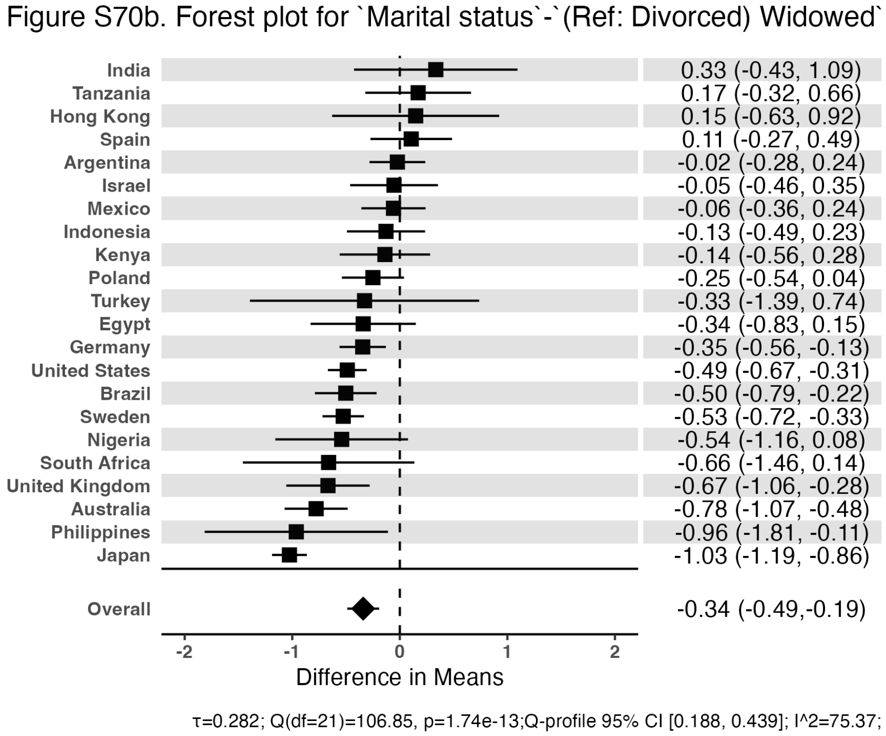

Figure S71. Heterogeneity in pairwise compairisons acrossx countries Marital status-(Ref: Domestic partner) Married. (a) Flourishing with financial indicators (12 items) [left panel]; (b) Flourishing without financial indicators (10 items) [right panel]. N=202,898, subgroup means and standard errors are computed accounting for the complex sampling design using all data simultaneously. Analyses conducted: Random-effects meta-analysis of country-specific means. Squares represent the the point estimate (mean) for each country. The lines represented the +/-1.96*SE, standard error, around the mean; the overall pooled mean is represented by the diamond. The reported p-value for Q-statistics is necessarily 1-sided because of the use of the chi-squared distribution to test whether heterogeneity is greater than zero (i.e., a two-sided test is not applicable). No adjustments for multiple testing were made.

Figure S71a Forest plot for `Marital status`-`(Ref: Domestic partner) Married`

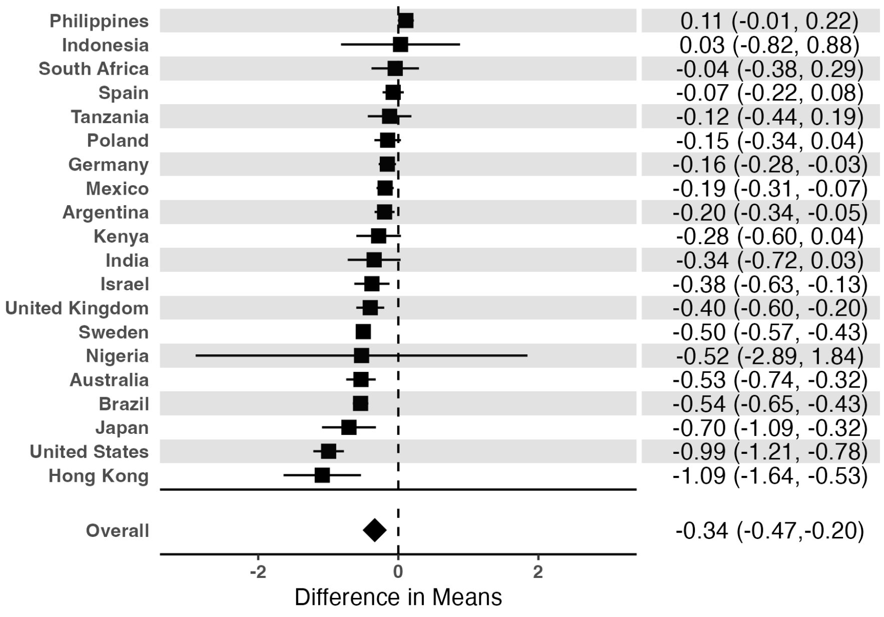

τ=0.262; Q(df=19)=177.90, p=<2e-16;Q-profile 95% CI [0.172, 0.391]; I^2=89.96;
Excluded countries: Egypt, Turkiye

Figure S71b. Forest plot for `Marital status`-`(Ref: Domestic partner) Married`

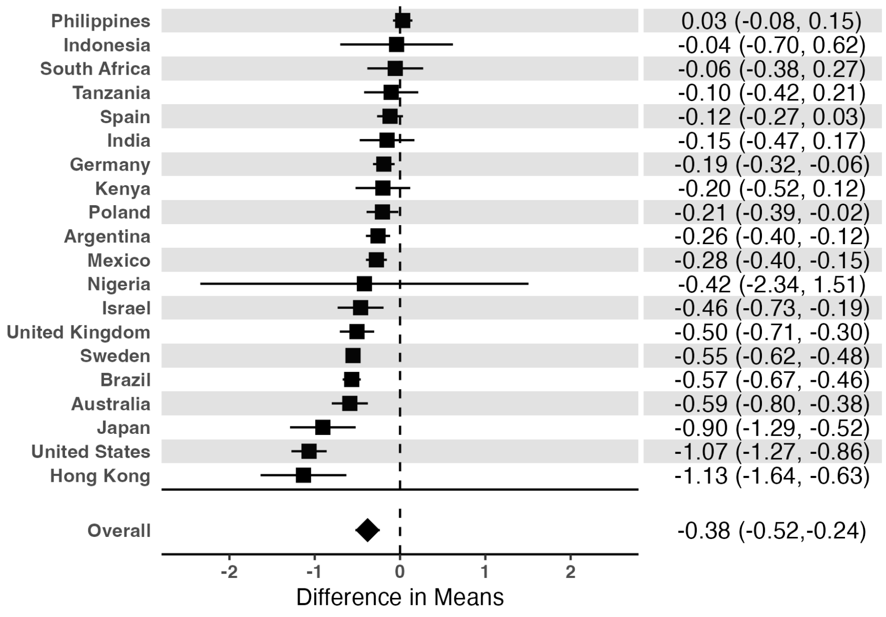

τ=0.291; Q(df=19)=188.68, p=<2e-16;Q-profile 95% CI [0.190, 0.426]; I^2=91.90;
Excluded countries: Egypt, Turkiye

Figure S72. Heterogeneity in pairwise compairisons acrossx countries Marital status-(Ref: Domestic partner) Separated. (a) Flourishing with financial indicators (12 items) [left panel]; (b) Flourishing without financial indicators (10 items) [right panel]. N=202,898, subgroup means and standard errors are computed accounting for the complex sampling design using all data simultaneously. Analyses conducted: Random-effects meta-analysis of country-specific means. Squares represent the the point estimate (mean) for each country. The lines represented the +/-1.96*SE, standard error, around the mean; the overall pooled mean is represented by the diamond. The reported p-value for Q-statistics is necessarily 1-sided because of the use of the chi-squared distribution to test whether heterogeneity is greater than zero (i.e., a two-sided test is not applicable). No adjustments for multiple testing were made.

Figure S72a Forest plot for `Marital status`-`(Ref: Domestic partner) Separated`

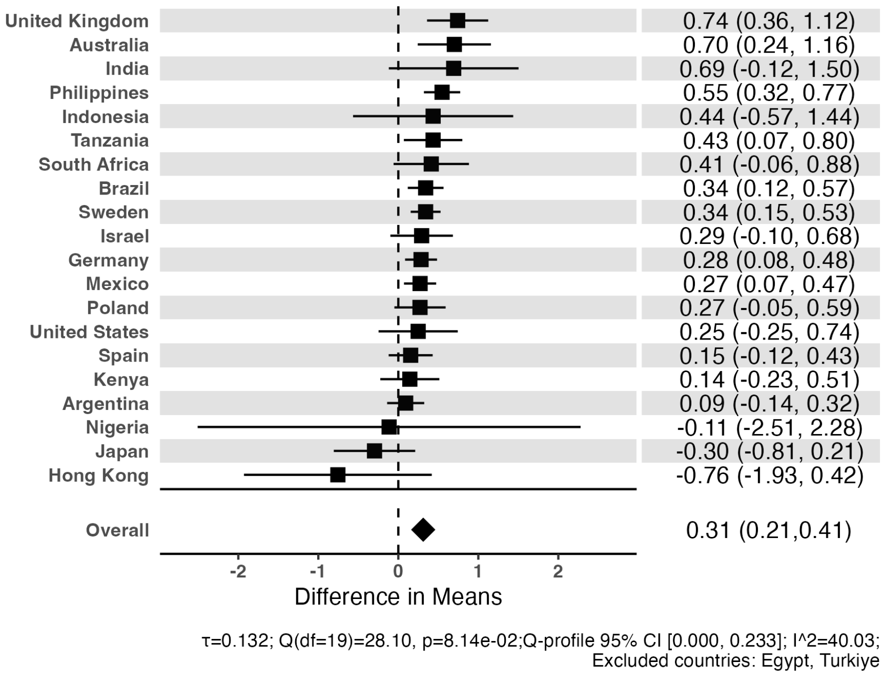

Figure S72b. Forest plot for `Marital status`-`(Ref: Domestic partner) Separated`

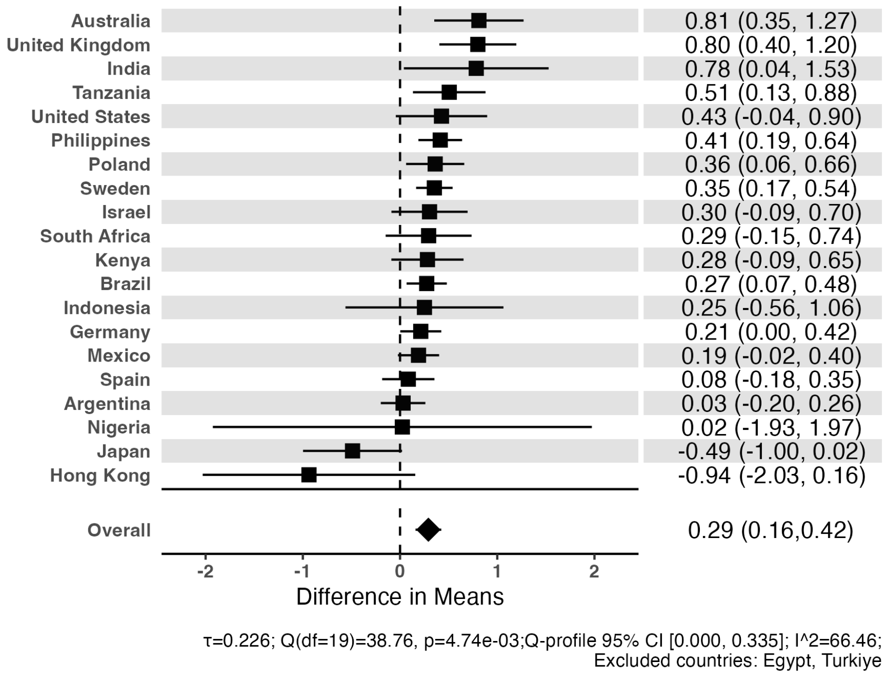

Figure S73. Heterogeneity in pairwise compairisons acrossx countries Marital status-(Ref: Domestic partner) Single, never married. (a) Flourishing with financial indicators (12 items) [left panel]; (b) Flourishing without financial indicators (10 items) [right panel]. N=202,898, subgroup means and standard errors are computed accounting for the complex sampling design using all data simultaneously. Analyses conducted: Random-effects meta-analysis of country-specific means. Squares represent the the point estimate (mean) for each country. The lines represented the +/-1.96*SE, standard error, around the mean; the overall pooled mean is represented by the diamond. The reported p-value for Q-statistics is necessarily 1-sided because of the use of the chi-squared distribution to test whether heterogeneity is greater than zero (i.e., a two-sided test is not applicable). No adjustments for multiple testing were made.

Figure S73a Forest plot for `Marital status`-`(Ref: Domestic partner) Single, never married`

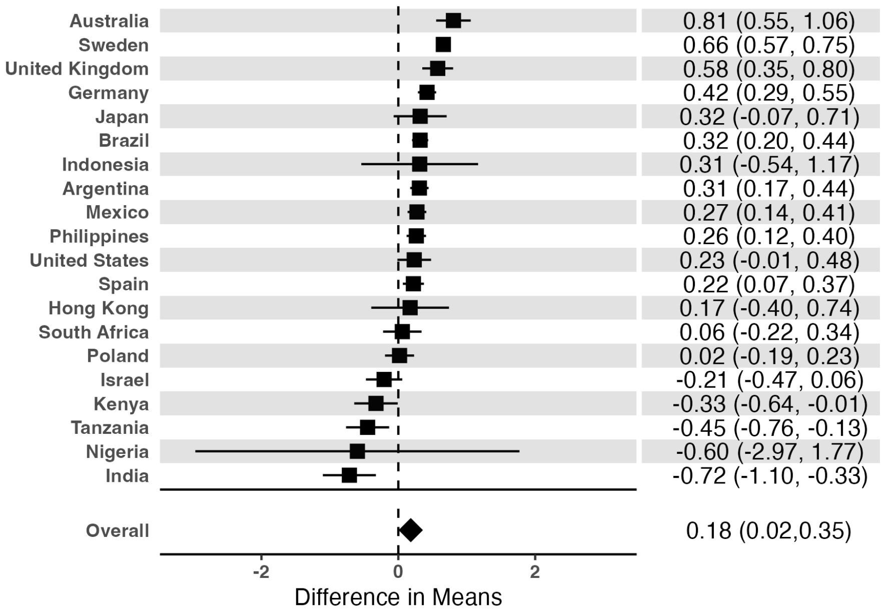

τ=0.338; Q(df=19)=173.76, p=<2e-16;Q-profile 95% CI [0.228, 0.498]; I^2=92.51;
Excluded countries: Egypt, Turkiye

Figure S73b. Forest plot for `Marital status`-`(Ref: Domestic partner) Single, never married`

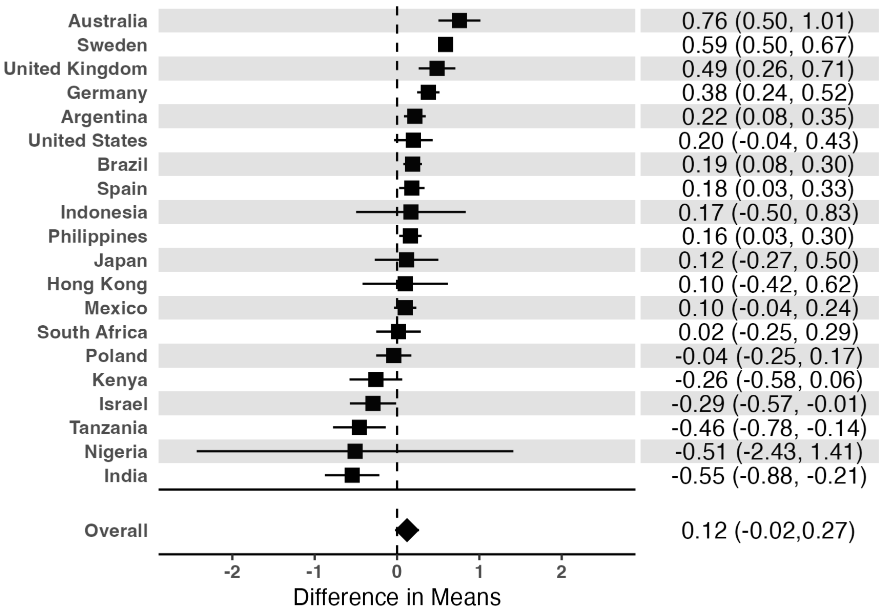

τ=0.296; Q(df=19)=169.07, p=<2e-16;Q-profile 95% CI [0.200, 0.440]; I^2=90.77;
Excluded countries: Egypt, Turkiye

Figure S74. Heterogeneity in pairwise compairisons acrossx countries Marital status-(Ref: Domestic partner) Widowed. (a) Flourishing with financial indicators (12 items) [left panel]; (b) Flourishing without financial indicators (10 items) [right panel]. N=202,898, subgroup means and standard errors are computed accounting for the complex sampling design using all data simultaneously. Analyses conducted: Random-effects meta-analysis of country-specific means. Squares represent the the point estimate (mean) for each country. The lines represented the +/-1.96*SE, standard error, around the mean; the overall pooled mean is represented by the diamond. The reported p-value for Q-statistics is necessarily 1-sided because of the use of the chi-squared distribution to test whether heterogeneity is greater than zero (i.e., a two-sided test is not applicable). No adjustments for multiple testing were made.

Figure S74a Forest plot for `Marital status`-`(Ref: Domestic partner) Widowed`

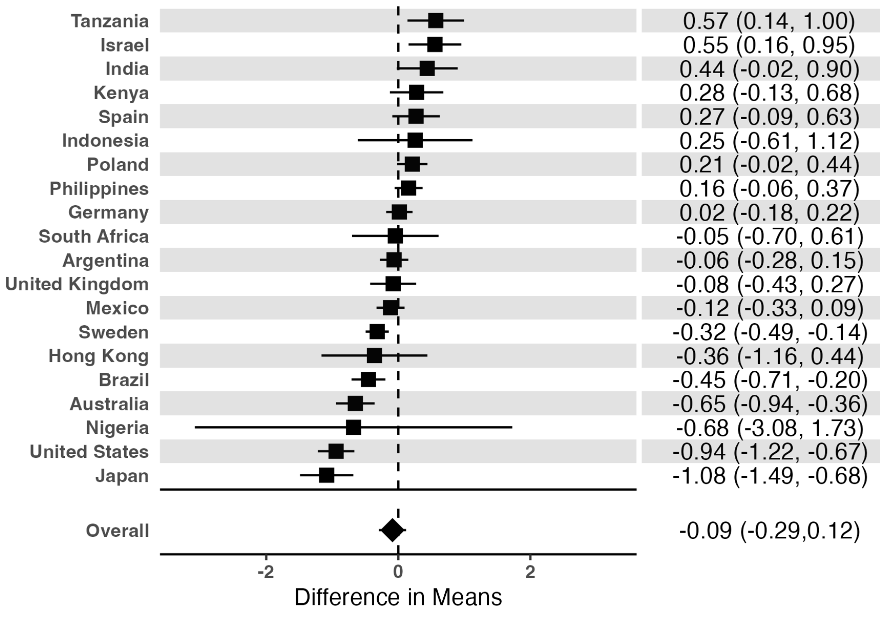

τ=0.415; Q(df=19)=132.76, p=<2e-16;Q-profile 95% CI [0.285, 0.622]; I^2=88.18;
Excluded countries: Egypt, Turkiye

Figure S74b. Forest plot for `Marital status`-`(Ref: Domestic partner) Widowed`

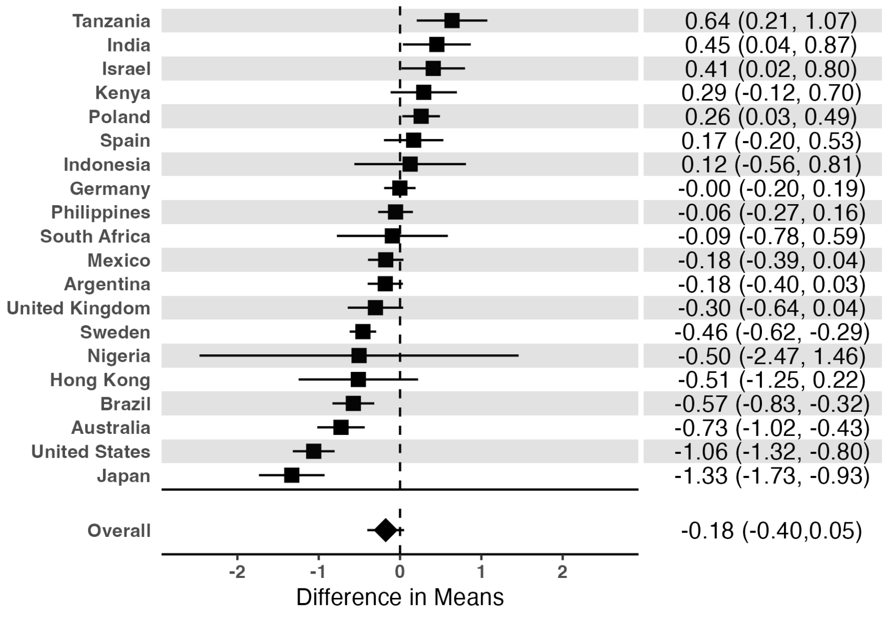

τ=0.469; Q(df=19)=168.46, p=<2e-16;Q-profile 95% CI [0.328, 0.690]; I^2=90.79;
Excluded countries: Egypt, Turkiye

Figure S75. Heterogeneity in pairwise compairisons acrossx countries Marital status-(Ref: Married) Separated. (a) Flourishing with financial indicators (12 items) [left panel]; (b) Flourishing without financial indicators (10 items) [right panel]. N=202,898, subgroup means and standard errors are computed accounting for the complex sampling design using all data simultaneously. Analyses conducted: Random-effects meta-analysis of country-specific means. Squares represent the the point estimate (mean) for each country. The lines represented the +/-1.96*SE, standard error, around the mean; the overall pooled mean is represented by the diamond. The reported p-value for Q-statistics is necessarily 1-sided because of the use of the chi-squared distribution to test whether heterogeneity is greater than zero (i.e., a two-sided test is not applicable). No adjustments for multiple testing were made.

Figure S75a Forest plot for `Marital status`-`(Ref: Married) Separated`

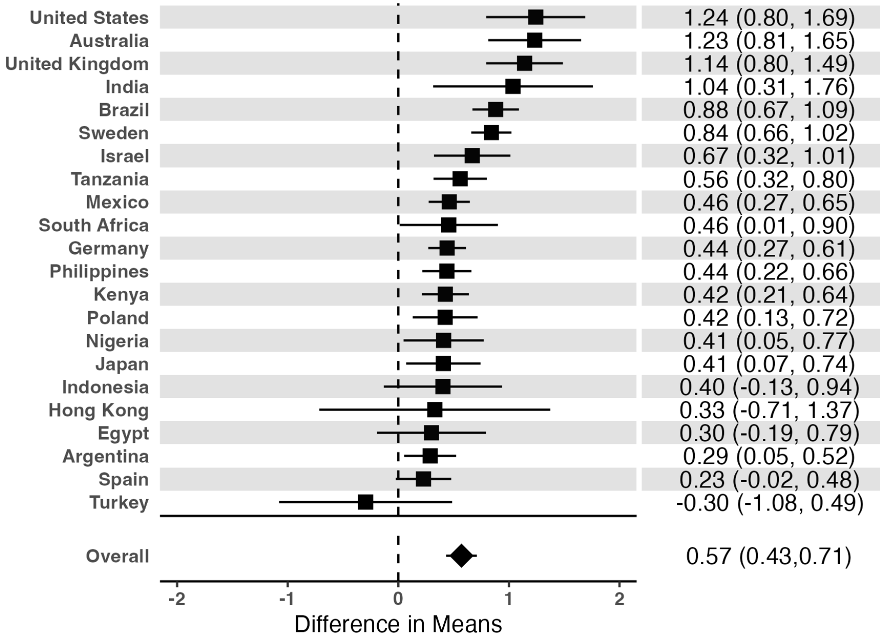

τ=0.276; Q(df=21)=76.06, p=3.64e-08;Q-profile 95% CI [0.143, 0.400]; I^2=78.56;

Figure S75b. Forest plot for `Marital status`-`(Ref: Married) Separated`

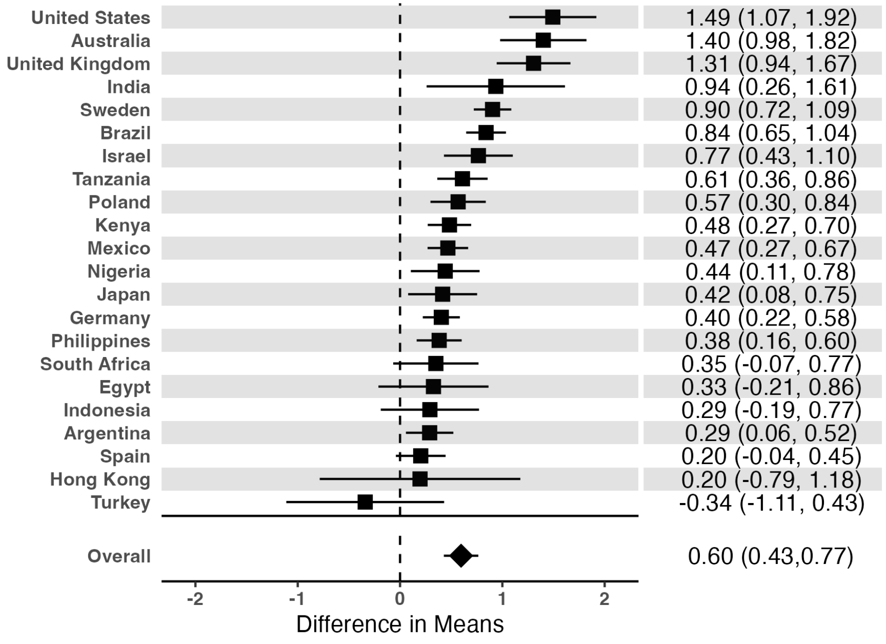

τ=0.356; Q(df=21)=104.08, p=5.44e-13;Q-profile 95% CI [0.207, 0.501]; I^2=85.97;

Figure S76. Heterogeneity in pairwise compairisons acrossx countries Marital status-(Ref: Married) Single, never married. (a) Flourishing with financial indicators (12 items) [left panel]; (b) Flourishing without financial indicators (10 items) [right panel]. N=202,898, subgroup means and standard errors are computed accounting for the complex sampling design using all data simultaneously. Analyses conducted: Random-effects meta-analysis of country-specific means. Squares represent the the point estimate (mean) for each country. The lines represented the +/-1.96*SE, standard error, around the mean; the overall pooled mean is represented by the diamond. The reported p-value for Q-statistics is necessarily 1-sided because of the use of the chi-squared distribution to test whether heterogeneity is greater than zero (i.e., a two-sided test is not applicable). No adjustments for multiple testing were made.

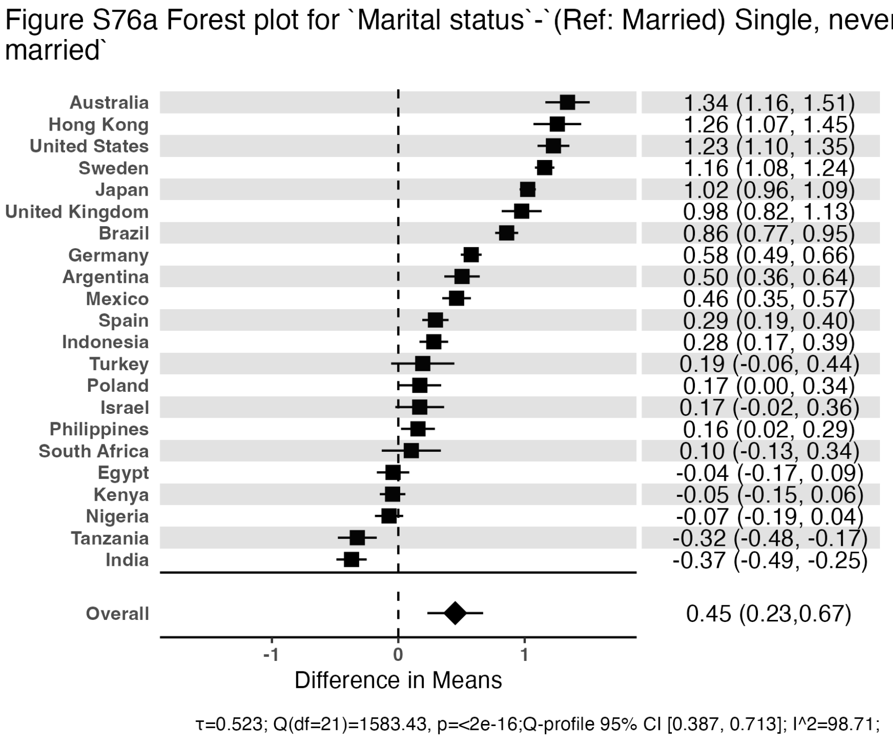

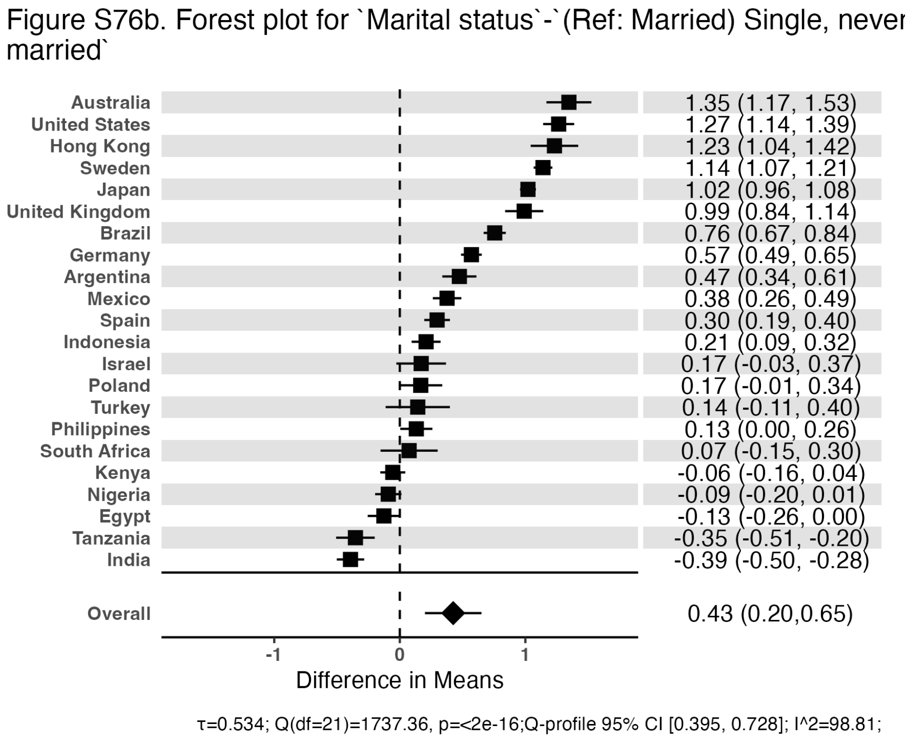

Figure S77. Heterogeneity in pairwise compairisons acrossx countries Marital status-(Ref: Married) Widowed. (a) Flourishing with financial indicators (12 items) [left panel]; (b) Flourishing without financial indicators (10 items) [right panel]. N=202,898, subgroup means and standard errors are computed accounting for the complex sampling design using all data simultaneously. Analyses conducted: Random-effects meta-analysis of country-specific means. Squares represent the the point estimate (mean) for each country. The lines represented the +/-1.96*SE, standard error, around the mean; the overall pooled mean is represented by the diamond. The reported p-value for Q-statistics is necessarily 1-sided because of the use of the chi-squared distribution to test whether heterogeneity is greater than zero (i.e., a two-sided test is not applicable). No adjustments for multiple testing were made.

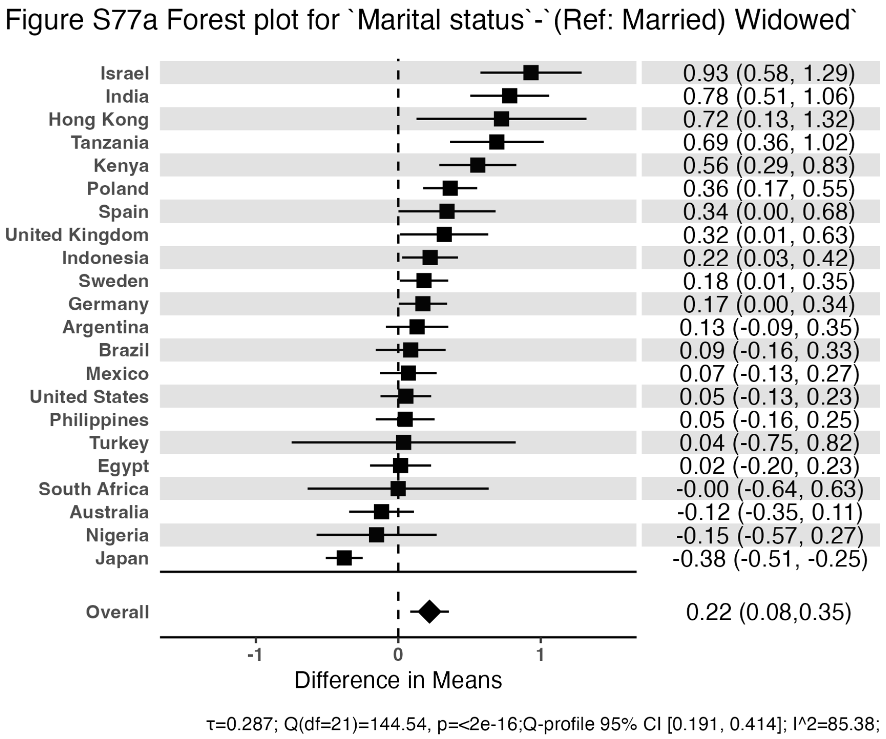

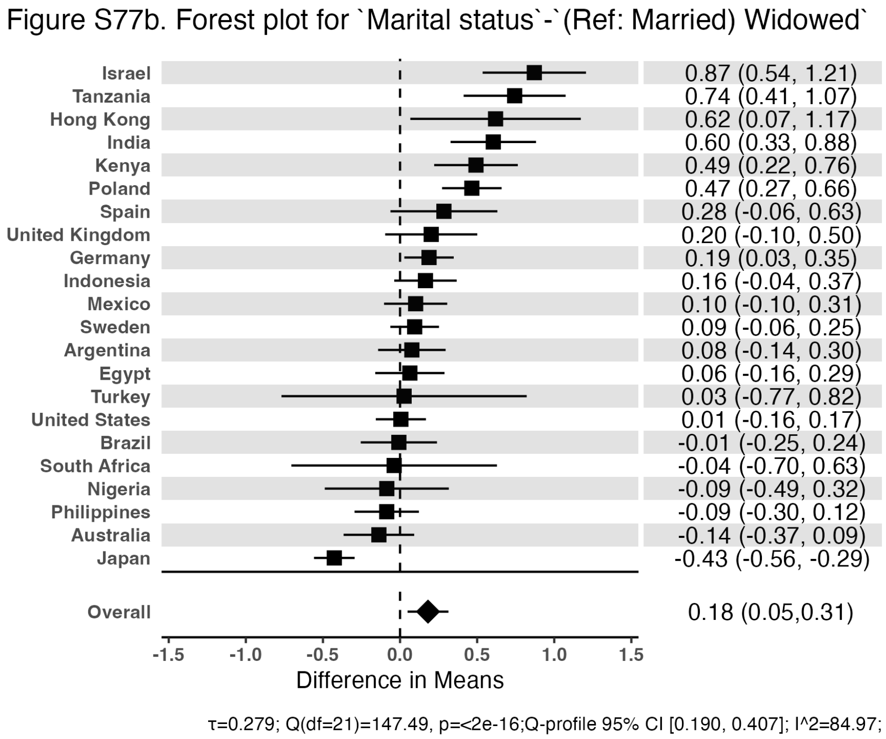

Figure S78. Heterogeneity in pairwise compairisons acrossx countries Marital status-(Ref: Separated) Single, never married. (a) Flourishing with financial indicators (12 items) [left panel]; (b) Flourishing without financial indicators (10 items) [right panel]. N=202,898, subgroup means and standard errors are computed accounting for the complex sampling design using all data simultaneously. Analyses conducted: Random-effects meta-analysis of country-specific means. Squares represent the the point estimate (mean) for each country. The lines represented the +/-1.96*SE, standard error, around the mean; the overall pooled mean is represented by the diamond. The reported p-value for Q-statistics is necessarily 1-sided because of the use of the chi-squared distribution to test whether heterogeneity is greater than zero (i.e., a two-sided test is not applicable). No adjustments for multiple testing were made.

Figure S78a Forest plot for `Marital status`-`(Ref: Separated) Single, never married`

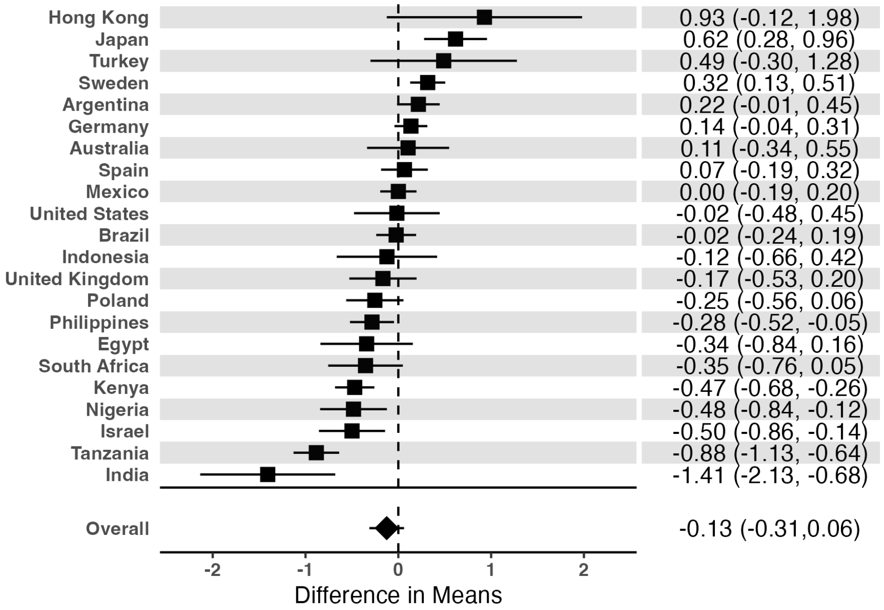

τ=0.400; Q(df=21)=137.70, p=<2e-16;Q-profile 95% CI [0.235, 0.551]; I^2=87.99;

Figure S78b. Forest plot for `Marital status`-`(Ref: Separated) Single, never married`

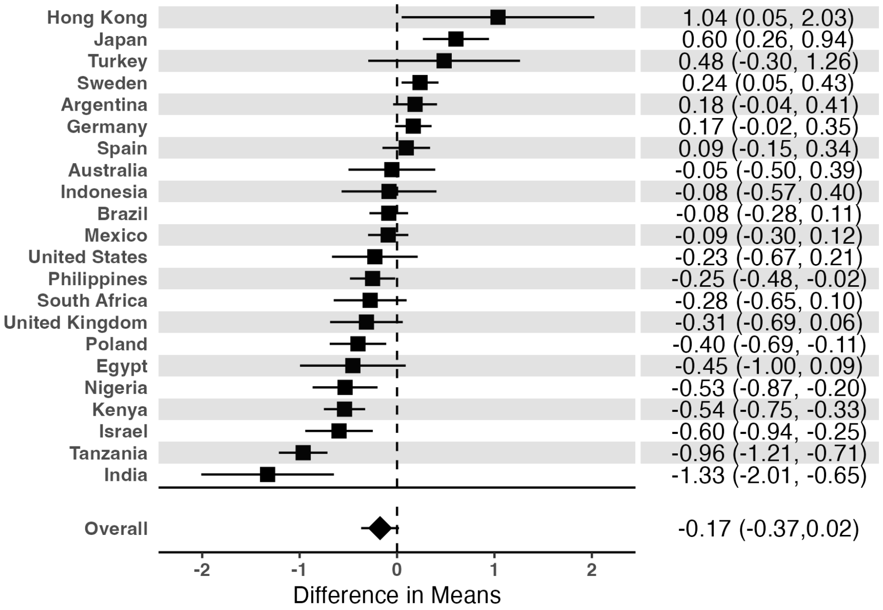

τ=0.421; Q(df=21)=149.93, p=<2e-16;Q-profile 95% CI [0.251, 0.577]; I^2=89.19;

Figure S79. Heterogeneity in pairwise compairisons acrossx countries Marital status-(Ref: Separated) Widowed. (a) Flourishing with financial indicators (12 items) [left panel]; (b) Flourishing without financial indicators (10 items) [right panel]. N=202,898, subgroup means and standard errors are computed accounting for the complex sampling design using all data simultaneously. Analyses conducted: Random-effects meta-analysis of country-specific means. Squares represent the the point estimate (mean) for each country. The lines represented the +/-1.96*SE, standard error, around the mean; the overall pooled mean is represented by the diamond. The reported p-value for Q-statistics is necessarily 1-sided because of the use of the chi-squared distribution to test whether heterogeneity is greater than zero (i.e., a two-sided test is not applicable). No adjustments for multiple testing were made.

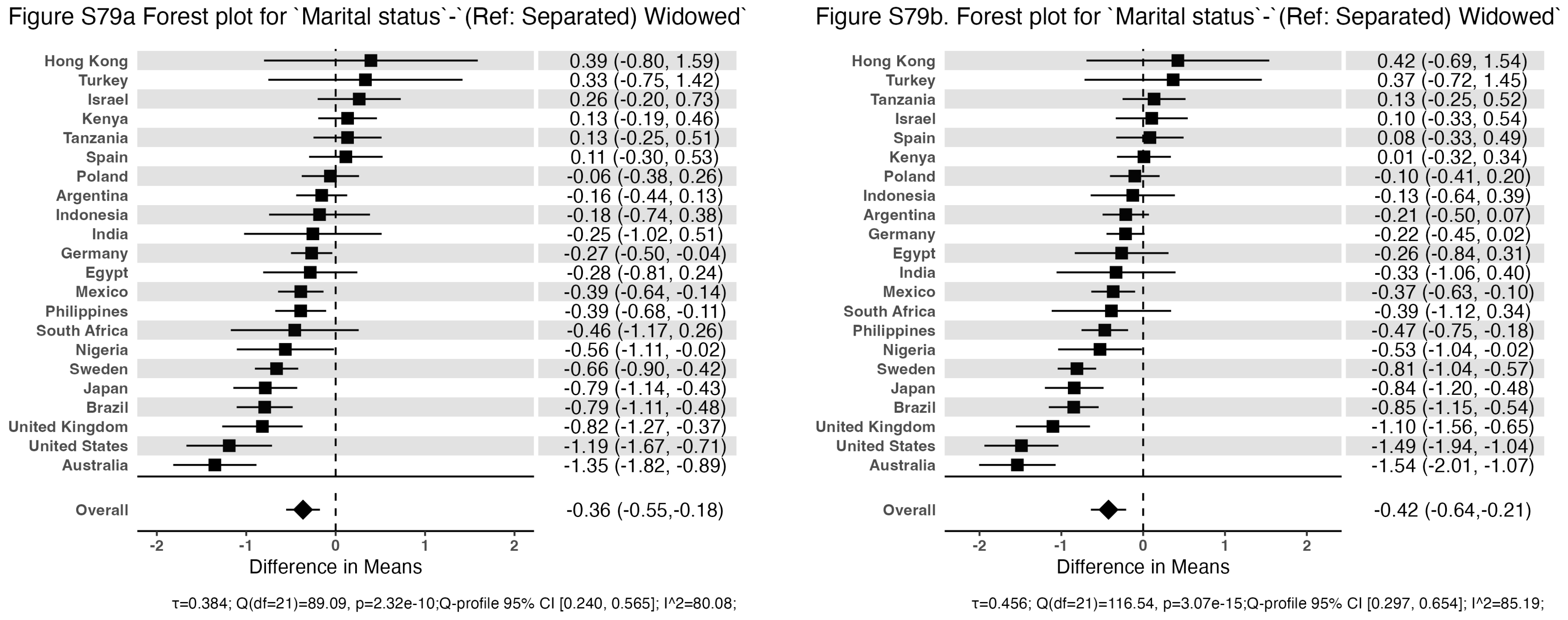

Figure S80. Heterogeneity in pairwise compairisons acrossx countries Marital status-(Ref: Single, never married) Widowed. (a) Flourishing with financial indicators (12 items) [left panel]; (b) Flourishing without financial indicators (10 items) [right panel]. N=202,898, subgroup means and standard errors are computed accounting for the complex sampling design using all data simultaneously. Analyses conducted: Random-effects meta-analysis of country-specific means. Squares represent the the point estimate (mean) for each country. The lines represented the +/-1.96*SE, standard error, around the mean; the overall pooled mean is represented by the diamond. The reported p-value for Q-statistics is necessarily 1-sided because of the use of the chi-squared distribution to test whether heterogeneity is greater than zero (i.e., a two-sided test is not applicable). No adjustments for multiple testing were made.

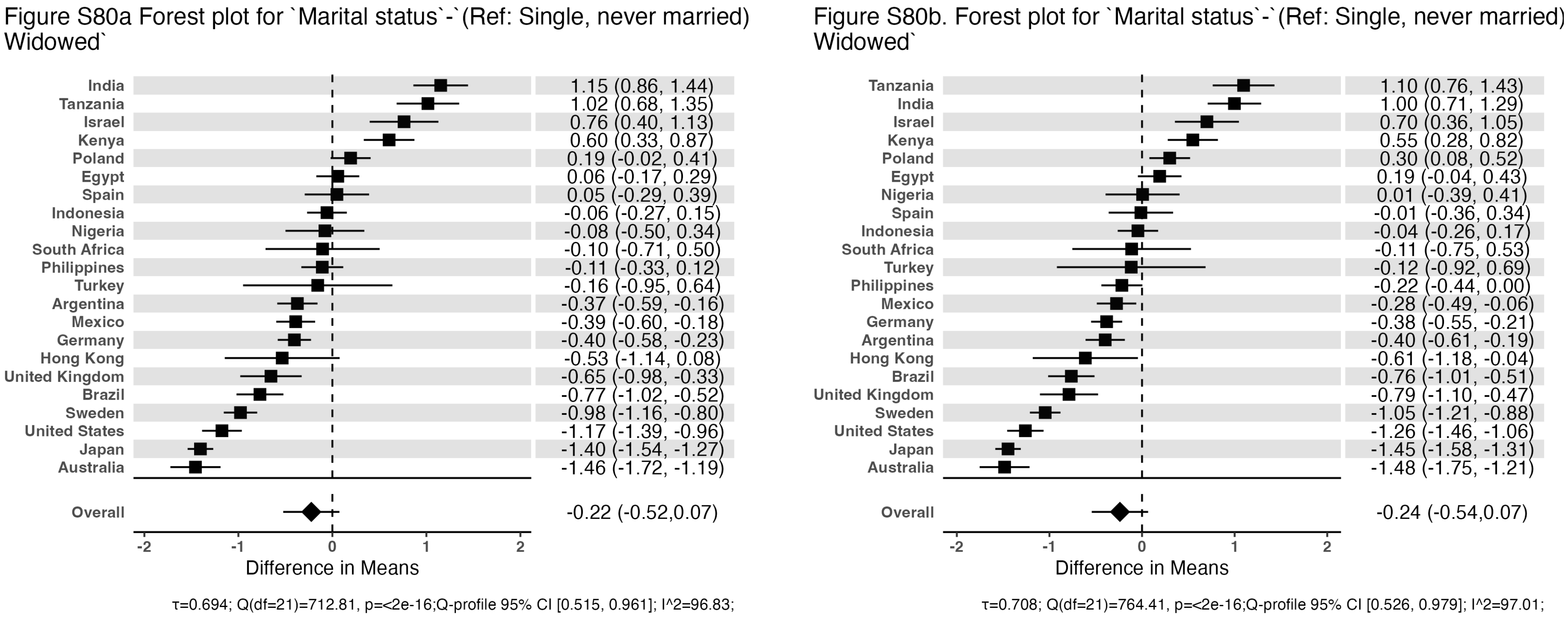

Figure S81. Heterogeneity in pairwise compairisons acrossx countries Employment status-(Ref: Employed for an employer) Homemaker. (a) Flourishing with financial indicators (12 items) [left panel]; (b) Flourishing without financial indicators (10 items) [right panel]. N=202,898, subgroup means and standard errors are computed accounting for the complex sampling design using all data simultaneously. Analyses conducted: Random-effects meta-analysis of country-specific means. Squares represent the the point estimate (mean) for each country. The lines represented the +/-1.96*SE, standard error, around the mean; the overall pooled mean is represented by the diamond. The reported p-value for Q-statistics is necessarily 1-sided because of the use of the chi-squared distribution to test whether heterogeneity is greater than zero (i.e., a two-sided test is not applicable). No adjustments for multiple testing were made.

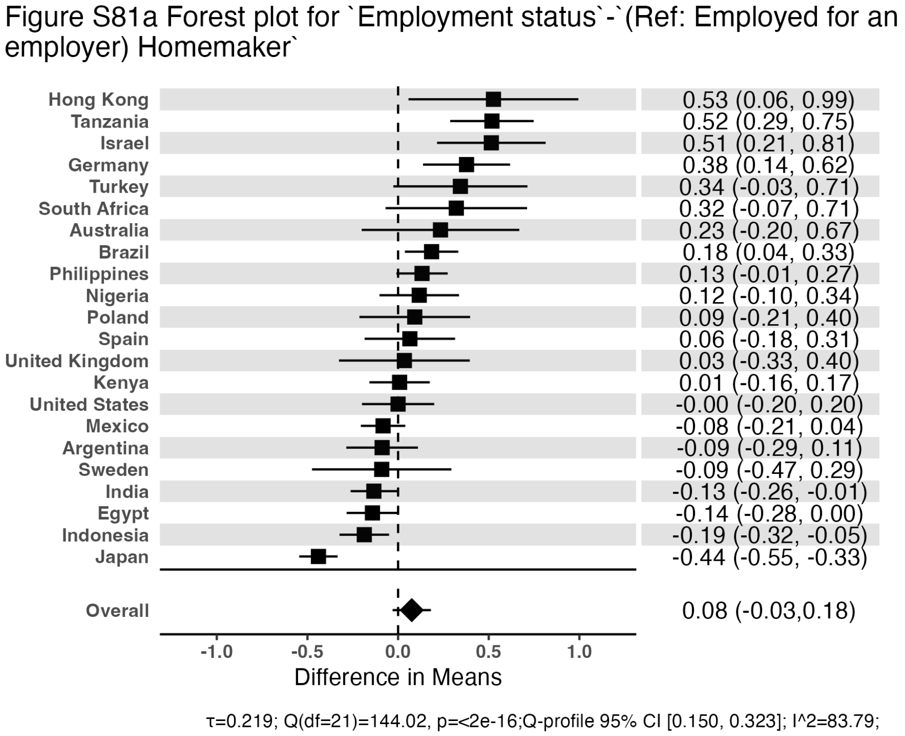

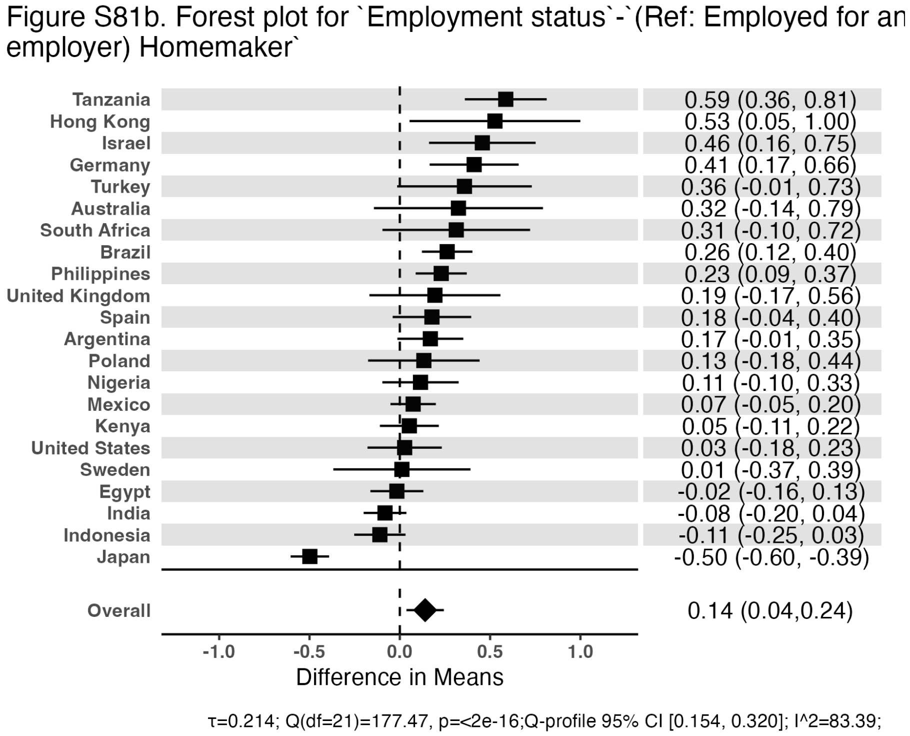

Figure S82. Heterogeneity in pairwise compairisons acrossx countries Employment status-(Ref: Employed for an employer) None of these/other. (a) Flourishing with financial indicators (12 items) [left panel]; (b) Flourishing without financial indicators (10 items) [right panel]. N=202,898, subgroup means and standard errors are computed accounting for the complex sampling design using all data simultaneously. Analyses conducted: Random-effects meta-analysis of country-specific means. Squares represent the the point estimate (mean) for each country. The lines represented the +/-1.96*SE, standard error, around the mean; the overall pooled mean is represented by the diamond. The reported p-value for Q-statistics is necessarily 1-sided because of the use of the chi-squared distribution to test whether heterogeneity is greater than zero (i.e., a two-sided test is not applicable). No adjustments for multiple testing were made.

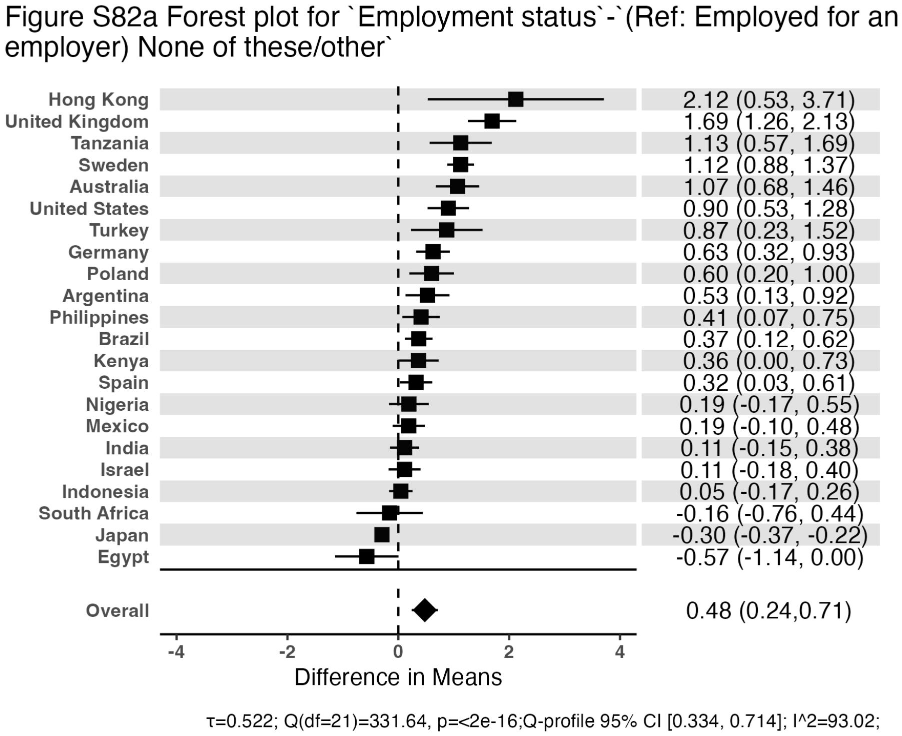

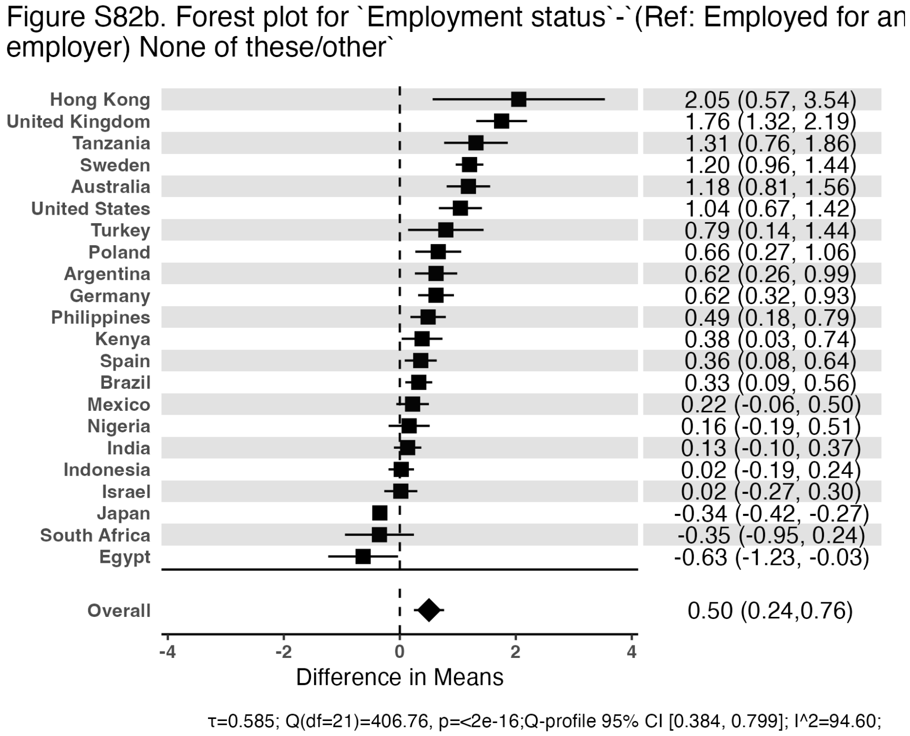

Figure S83. Heterogeneity in pairwise compairisons acrossx countries Employment status-(Ref: Employed for an employer) Retired. (a) Flourishing with financial indicators (12 items) [left panel]; (b) Flourishing without financial indicators (10 items) [right panel]. N=202,898, subgroup means and standard errors are computed accounting for the complex sampling design using all data simultaneously. Analyses conducted: Random-effects meta-analysis of country-specific means. Squares represent the the point estimate (mean) for each country. The lines represented the +/-1.96*SE, standard error, around the mean; the overall pooled mean is represented by the diamond. The reported p-value for Q-statistics is necessarily 1-sided because of the use of the chi-squared distribution to test whether heterogeneity is greater than zero (i.e., a two-sided test is not applicable). No adjustments for multiple testing were made.

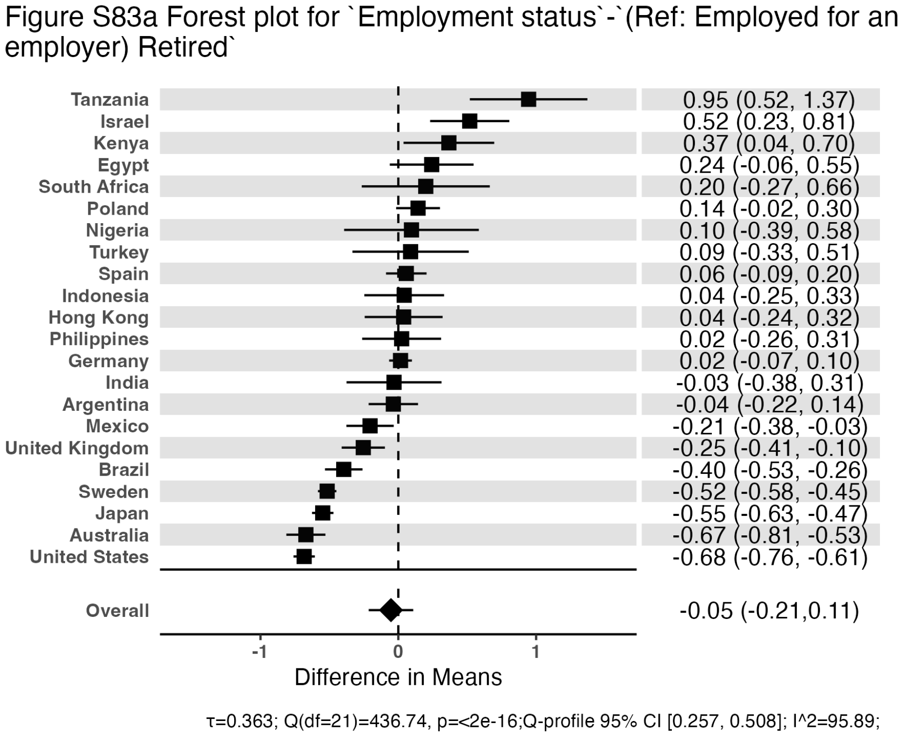

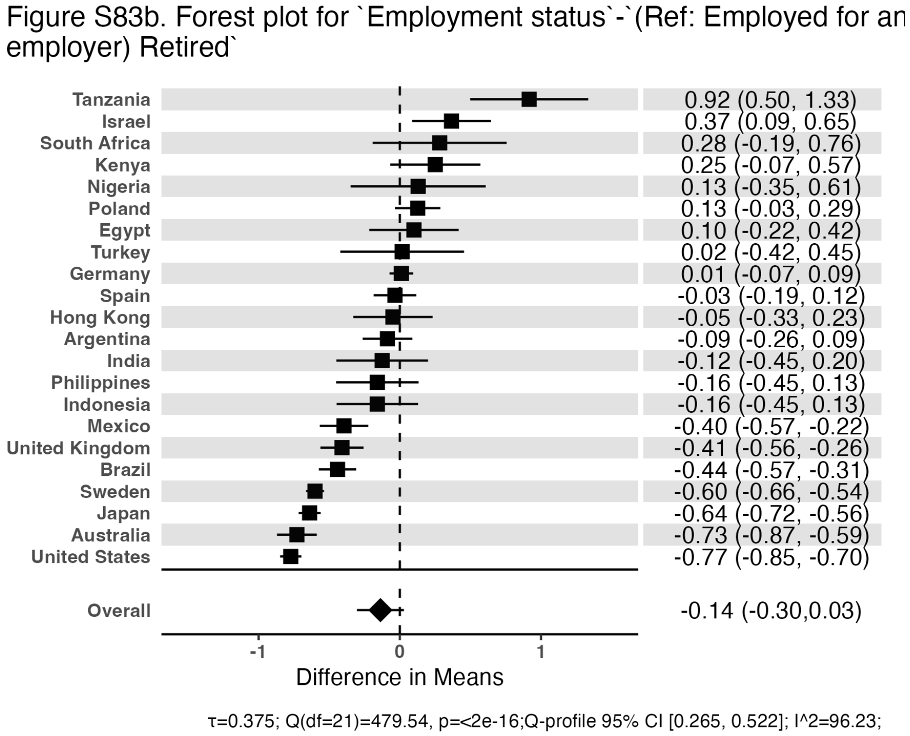

Figure S84. Heterogeneity in pairwise compairisons acrossx countries Employment status-(Ref: Employed for an employer) Self-employed. (a) Flourishing with financial indicators (12 items) [left panel]; (b) Flourishing without financial indicators (10 items) [right panel]. N=202,898, subgroup means and standard errors are computed accounting for the complex sampling design using all data simultaneously. Analyses conducted: Random-effects meta-analysis of country-specific means. Squares represent the the point estimate (mean) for each country. The lines represented the +/-1.96*SE, standard error, around the mean; the overall pooled mean is represented by the diamond. The reported p-value for Q-statistics is necessarily 1-sided because of the use of the chi-squared distribution to test whether heterogeneity is greater than zero (i.e., a two-sided test is not applicable). No adjustments for multiple testing were made.

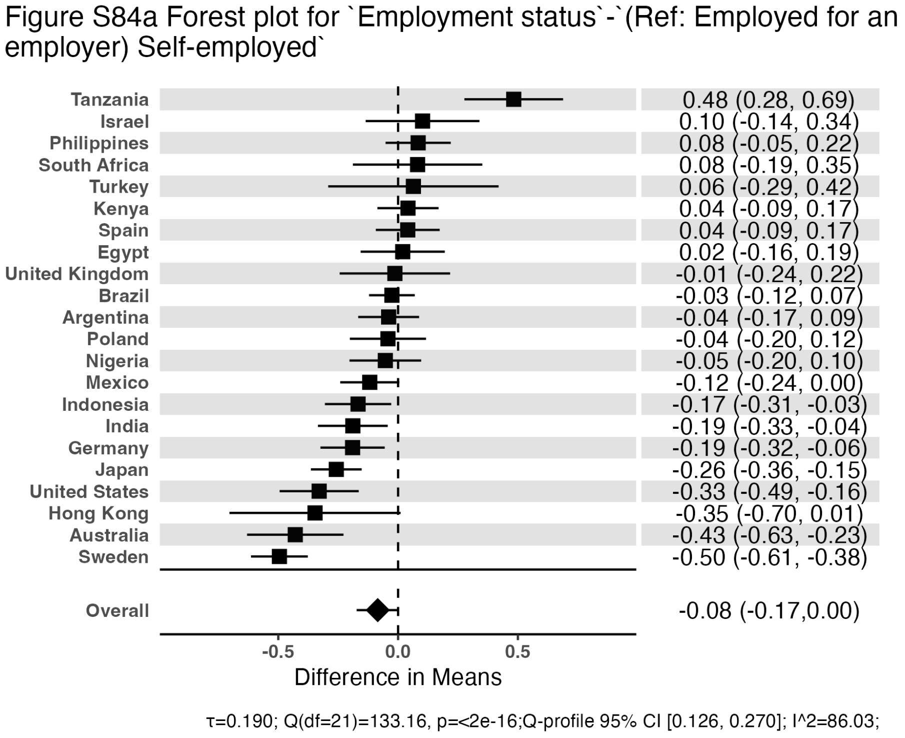

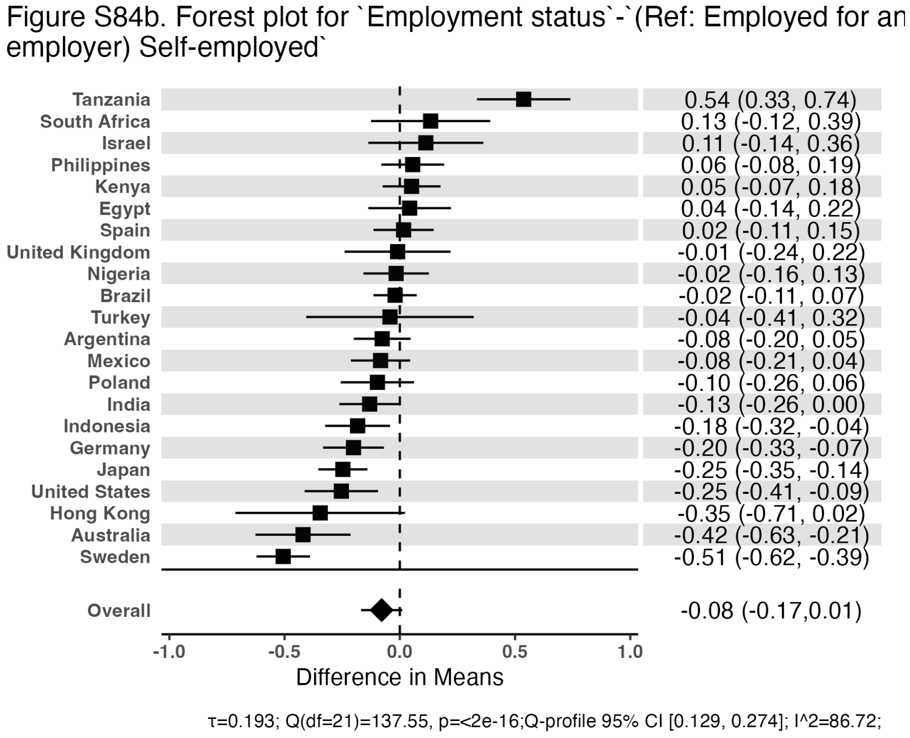

Figure S85. Heterogeneity in pairwise compairisons acrossx countries Employment status-(Ref: Employed for an employer) Student. (a) Flourishing with financial indicators (12 items) [left panel]; (b) Flourishing without financial indicators (10 items) [right panel]. N=202,898, subgroup means and standard errors are computed accounting for the complex sampling design using all data simultaneously. Analyses conducted: Random-effects meta-analysis of country-specific means. Squares represent the the point estimate (mean) for each country. The lines represented the +/-1.96*SE, standard error, around the mean; the overall pooled mean is represented by the diamond. The reported p-value for Q-statistics is necessarily 1-sided because of the use of the chi-squared distribution to test whether heterogeneity is greater than zero (i.e., a two-sided test is not applicable). No adjustments for multiple testing were made.

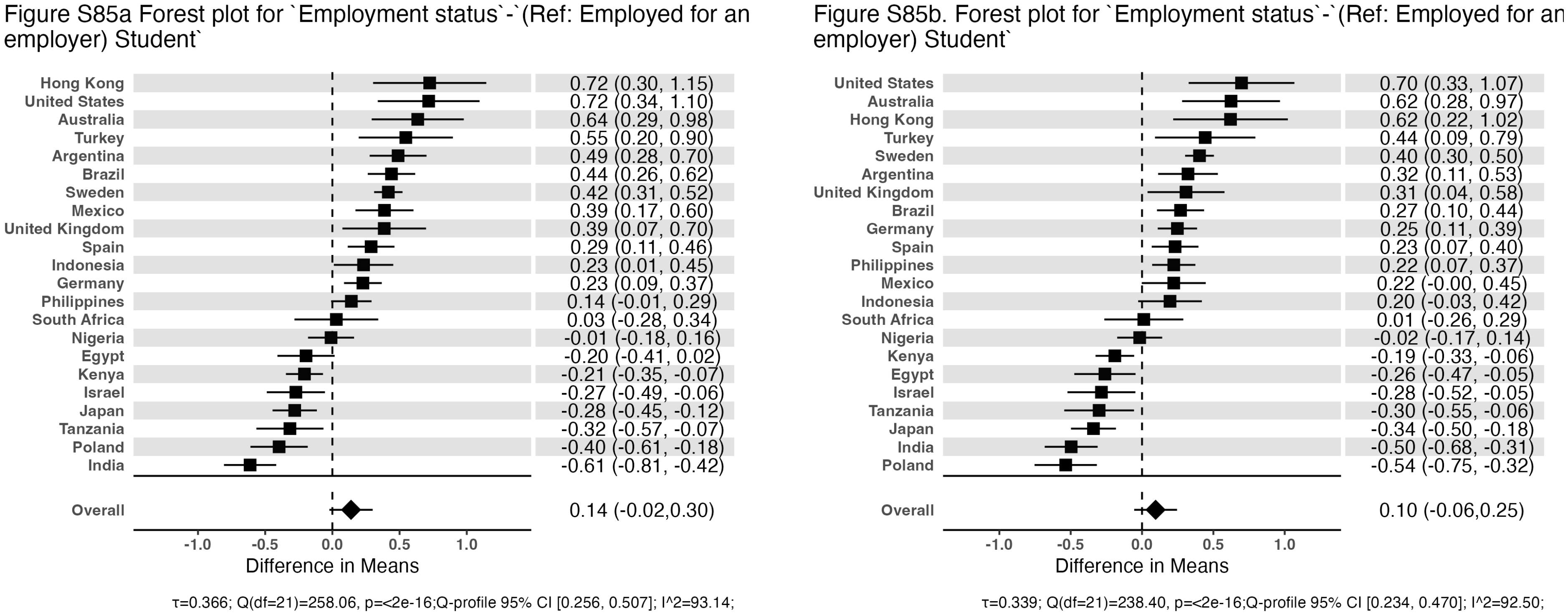

Figure S86. Heterogeneity in pairwise compairisons acrossx countries Employment status-(Ref: Employed for an employer) Unemployed and looking for a job. (a) Flourishing with financial indicators (12 items) [left panel]; (b) Flourishing without financial indicators (10 items) [right panel]. N=202,898, subgroup means and standard errors are computed accounting for the complex sampling design using all data simultaneously. Analyses conducted: Random-effects meta-analysis of country-specific means. Squares represent the the point estimate (mean) for each country. The lines represented the +/-1.96*SE, standard error, around the mean; the overall pooled mean is represented by the diamond. The reported p-value for Q-statistics is necessarily 1-sided because of the use of the chi-squared distribution to test whether heterogeneity is greater than zero (i.e., a two-sided test is not applicable). No adjustments for multiple testing were made.

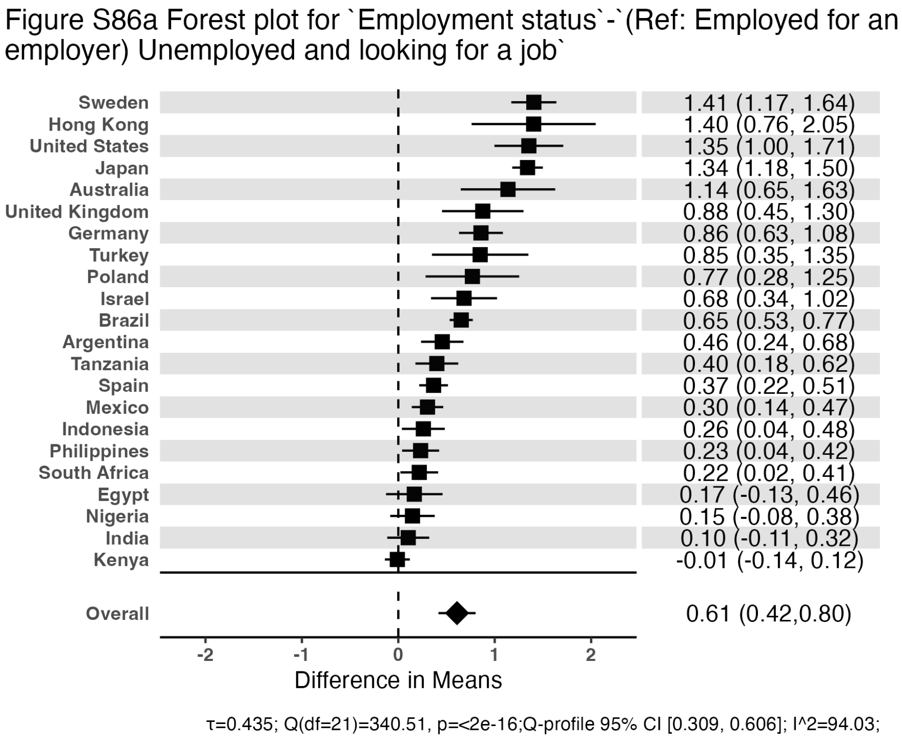

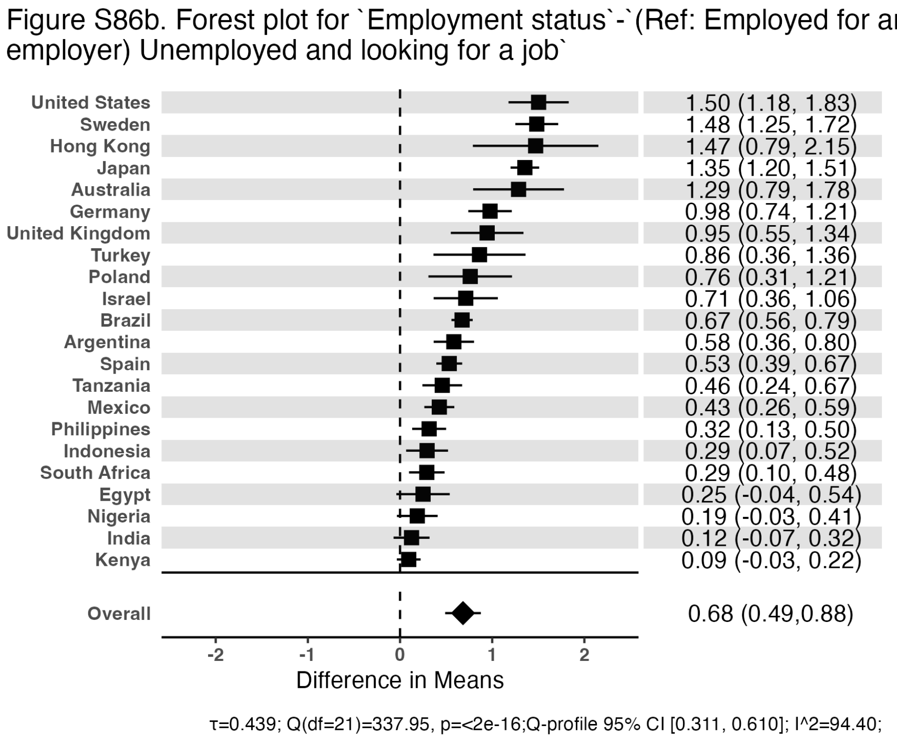

Figure S87. Heterogeneity in pairwise compairisons acrossx countries Employment status-(Ref: Homemaker) None of these/other. (a) Flourishing with financial indicators (12 items) [left panel]; (b) Flourishing without financial indicators (10 items) [right panel]. N=202,898, subgroup means and standard errors are computed accounting for the complex sampling design using all data simultaneously. Analyses conducted: Random-effects meta-analysis of country-specific means. Squares represent the the point estimate (mean) for each country. The lines represented the +/-1.96*SE, standard error, around the mean; the overall pooled mean is represented by the diamond. The reported p-value for Q-statistics is necessarily 1-sided because of the use of the chi-squared distribution to test whether heterogeneity is greater than zero (i.e., a two-sided test is not applicable). No adjustments for multiple testing were made.

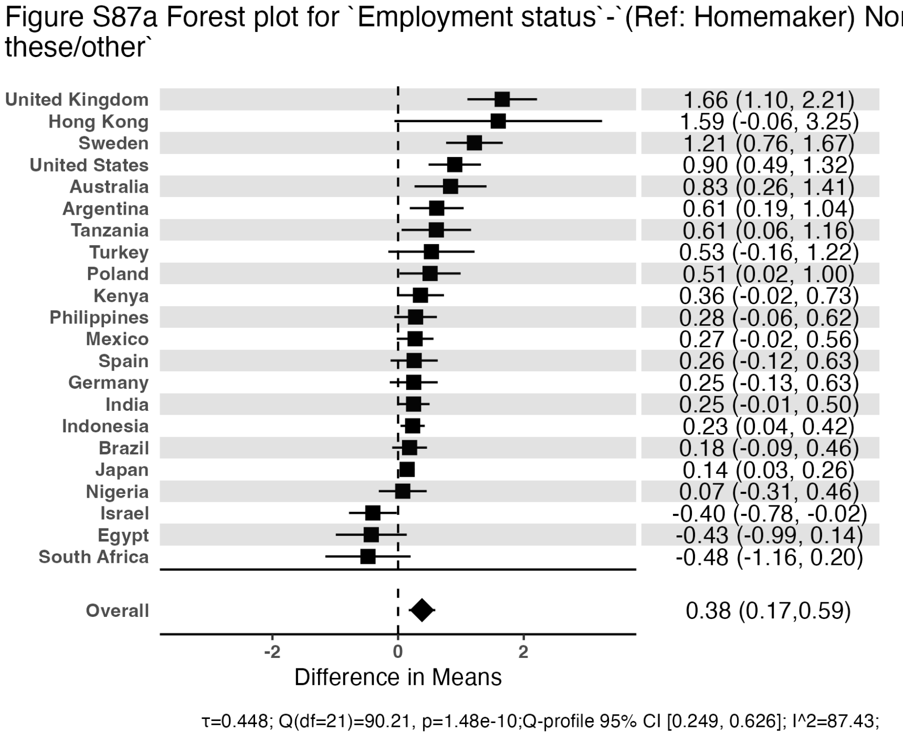

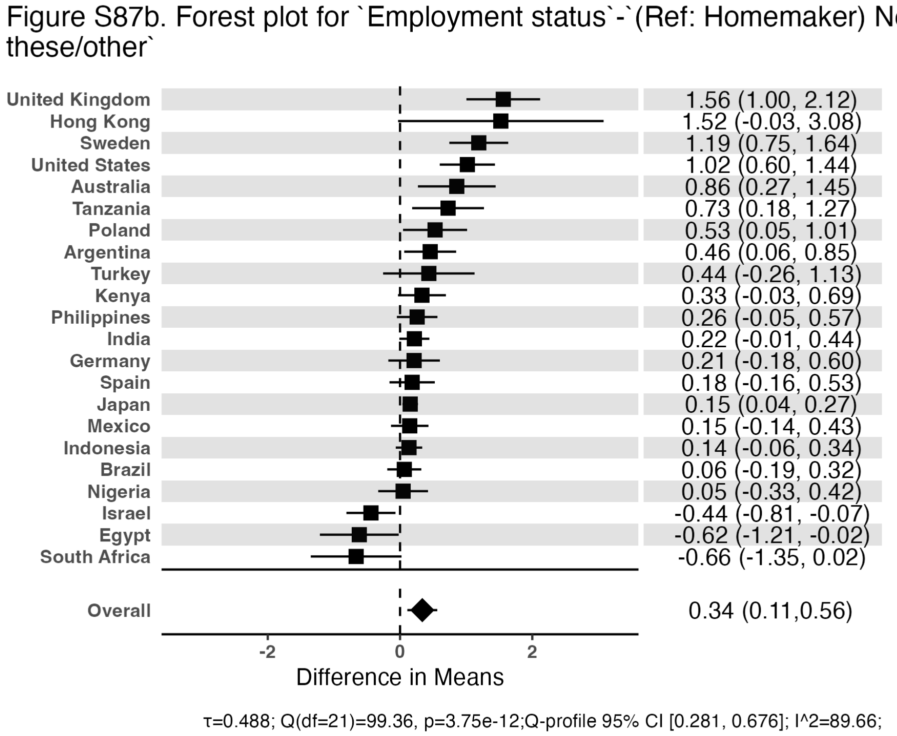

Figure S88. Heterogeneity in pairwise compairisons acrossx countries Employment status-(Ref: Homemaker) Retired. (a) Flourishing with financial indicators (12 items) [left panel]; (b) Flourishing without financial indicators (10 items) [right panel]. N=202,898, subgroup means and standard errors are computed accounting for the complex sampling design using all data simultaneously. Analyses conducted: Random-effects meta-analysis of country-specific means. Squares represent the the point estimate (mean) for each country. The lines represented the +/-1.96*SE, standard error, around the mean; the overall pooled mean is represented by the diamond. The reported p-value for Q-statistics is necessarily 1-sided because of the use of the chi-squared distribution to test whether heterogeneity is greater than zero (i.e., a two-sided test is not applicable). No adjustments for multiple testing were made.

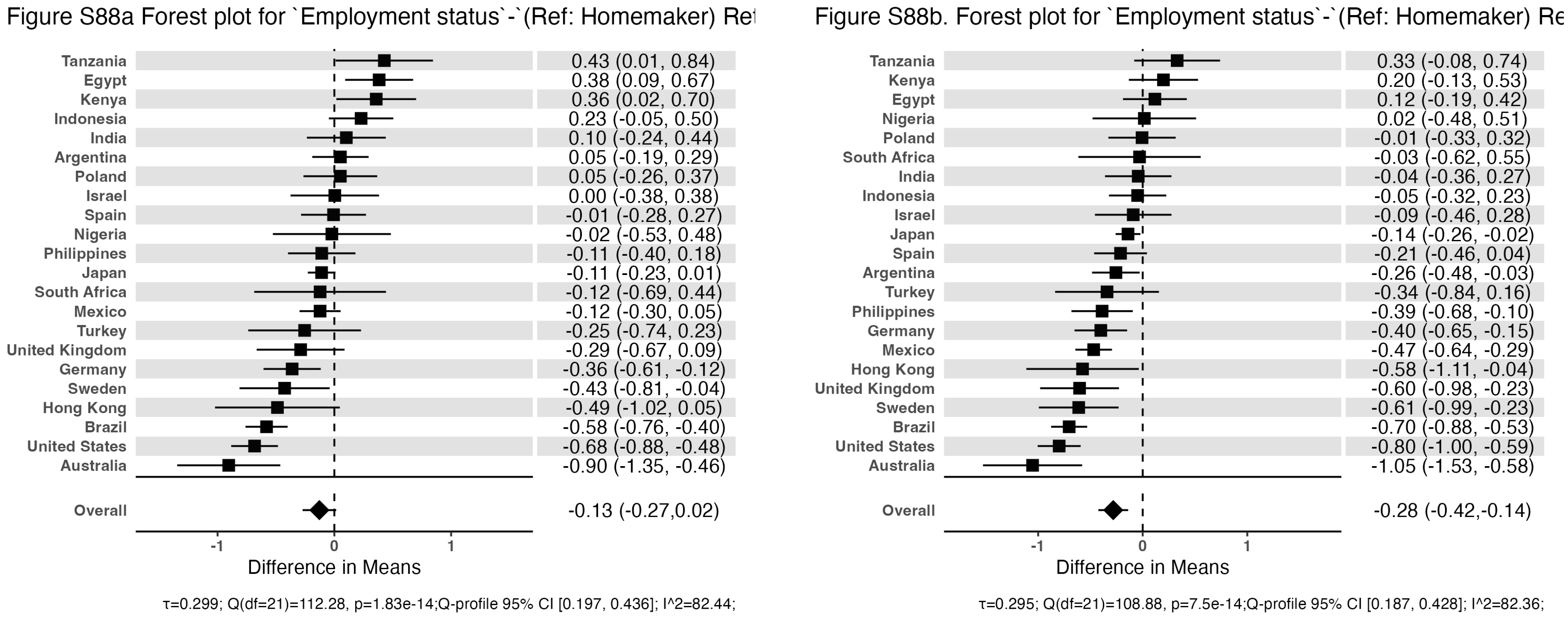

Figure S89. Heterogeneity in pairwise compairisons acrossx countries Employment status-(Ref: Homemaker) Self-employed. (a) Flourishing with financial indicators (12 items) [left panel]; (b) Flourishing without financial indicators (10 items) [right panel]. N=202,898, subgroup means and standard errors are computed accounting for the complex sampling design using all data simultaneously. Analyses conducted: Random-effects meta-analysis of country-specific means. Squares represent the the point estimate (mean) for each country. The lines represented the +/- 1.96*SE, standard error, around the mean; the overall pooled mean is represented by the diamond. The reported p-value for Q-statistics is necessarily 1-sided because of the use of the chi-squared distribution to test whether heterogeneity is greater than zero (i.e., a two-sided test is not applicable). No adjustments for multiple testing were made.

Figure S89a Forest plot for `Employment status`-`(Ref: Homemaker) Self-employed`

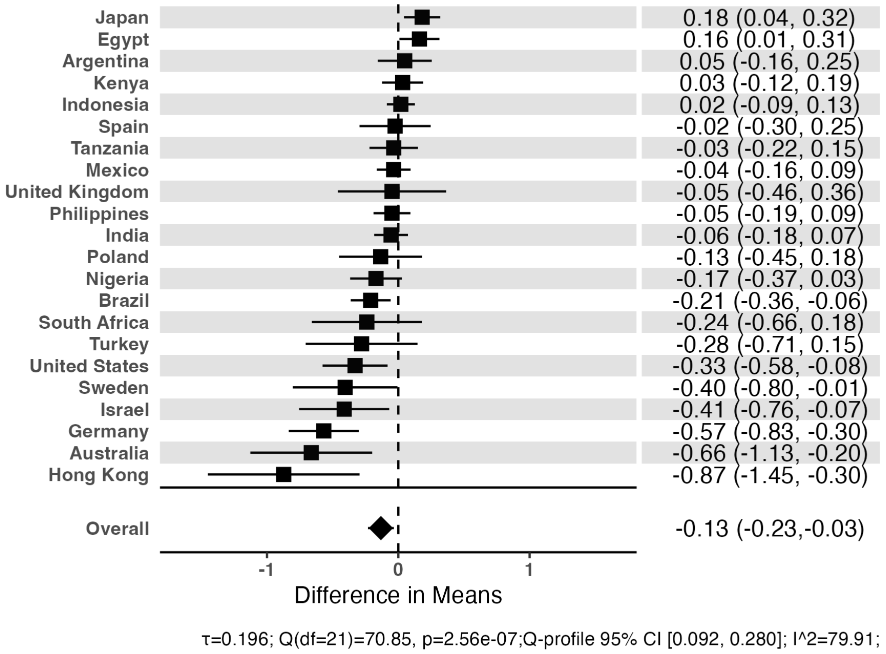

Figure S89b. Forest plot for `Employment status`-`(Ref: Homemaker) Self-employed`

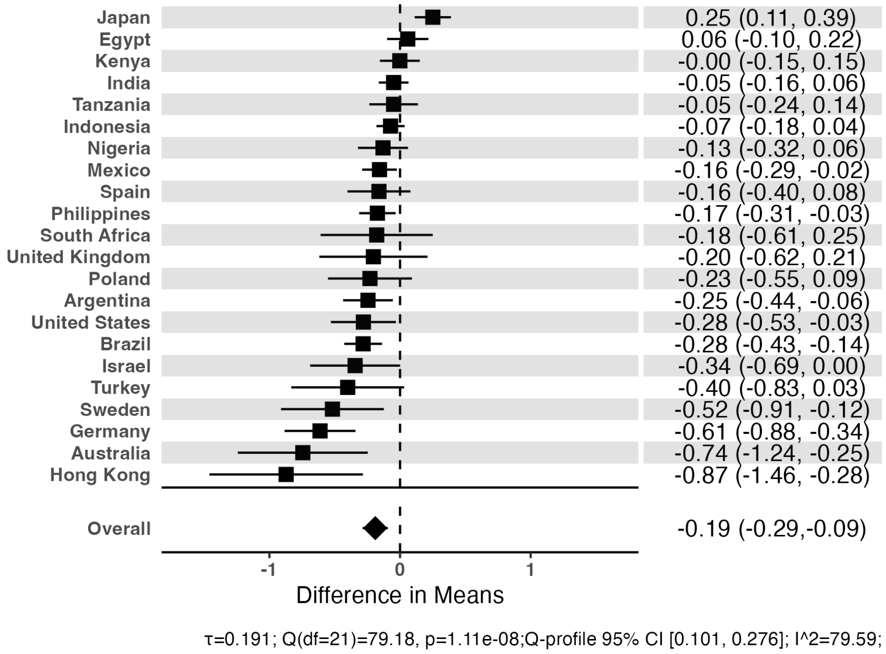

Figure S90. Heterogeneity in pairwise compairisons acrossx countries Employment status-(Ref: Homemaker) Student. (a) Flourishing with financial indicators (12 items) [left panel]; (b) Flourishing without financial indicators (10 items) [right panel]. N=202,898, subgroup means and standard errors are computed accounting for the complex sampling design using all data simultaneously. Analyses conducted: Random-effects meta-analysis of country-specific means. Squares represent the the point estimate (mean) for each country. The lines represented the +/-1.96*SE, standard error, around the mean; the overall pooled mean is represented by the diamond. The reported p-value for Q-statistics is necessarily 1-sided because of the use of the chi-squared distribution to test whether heterogeneity is greater than zero (i.e., a two-sided test is not applicable). No adjustments for multiple testing were made.

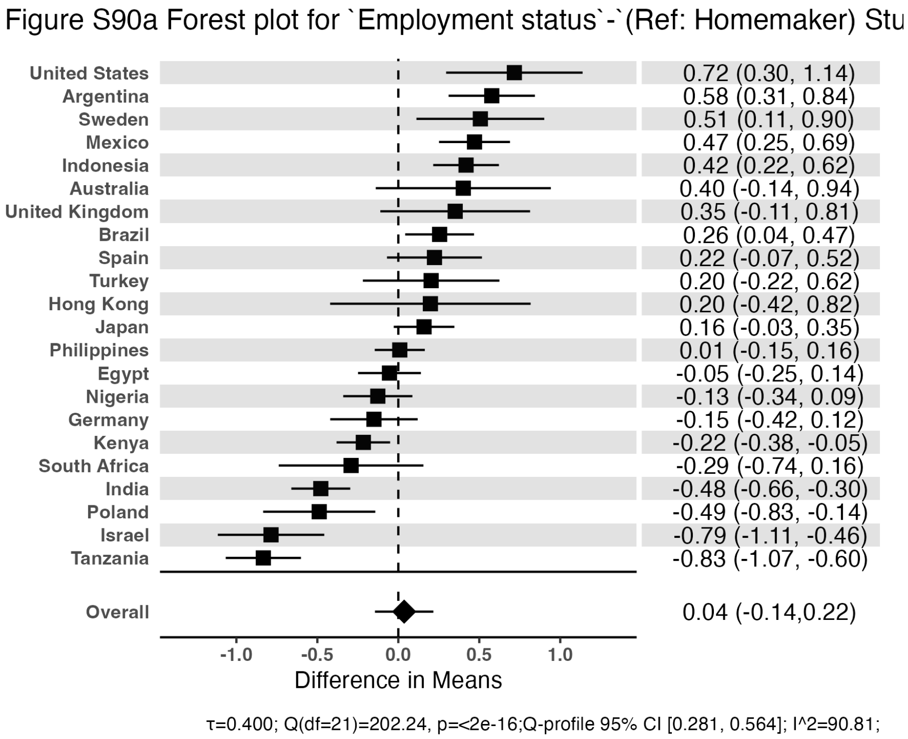

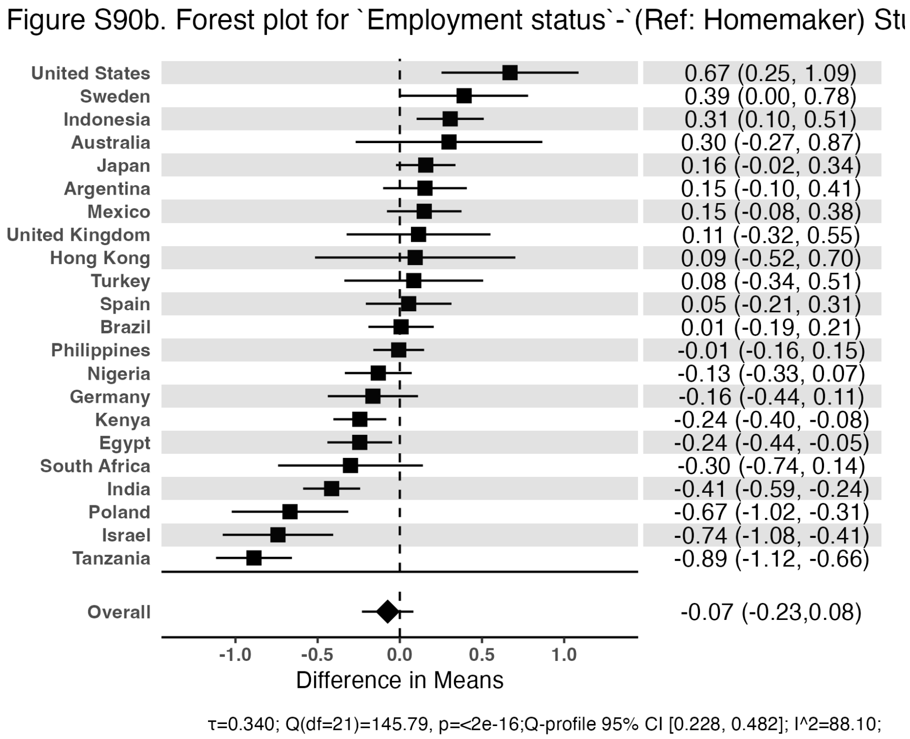

Figure S91. Heterogeneity in pairwise compairisons acrossx countries Employment status-(Ref: Homemaker) Unemployed and looking for a job. (a) Flourishing with financial indicators (12 items) [left panel]; (b) Flourishing without financial indicators (10 items) [right panel]. N=202,898, subgroup means and standard errors are computed accounting for the complex sampling design using all data simultaneously. Analyses conducted: Random-effects meta-analysis of country-specific means. Squares represent the the point estimate (mean) for each country. The lines represented the +/-1.96*SE, standard error, around the mean; the overall pooled mean is represented by the diamond. The reported p-value for Q-statistics is necessarily 1-sided because of the use of the chi-squared distribution to test whether heterogeneity is greater than zero (i.e., a two-sided test is not applicable). No adjustments for multiple testing were made.

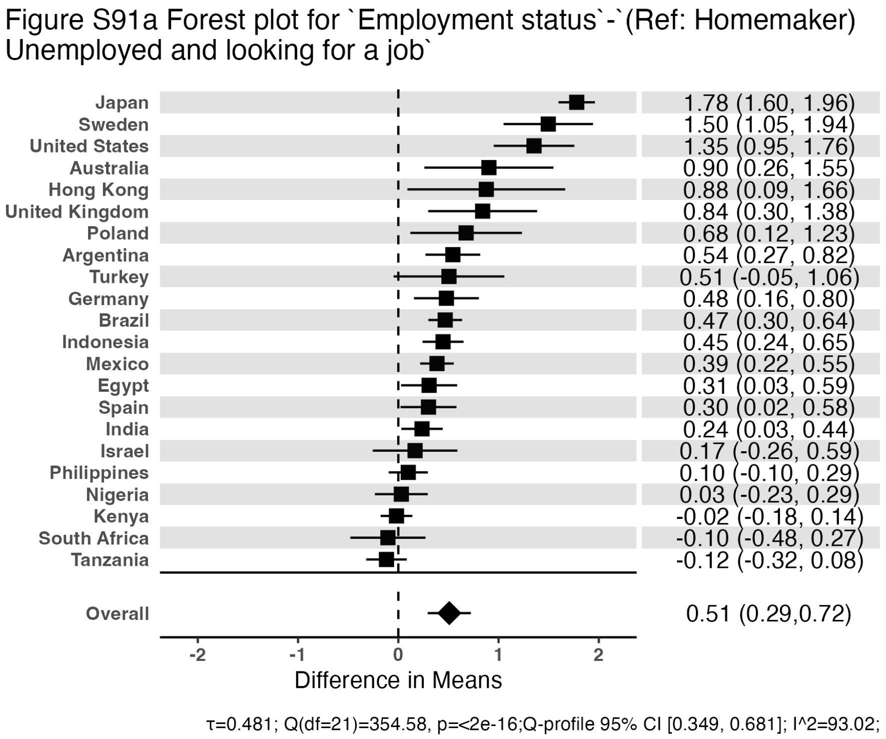

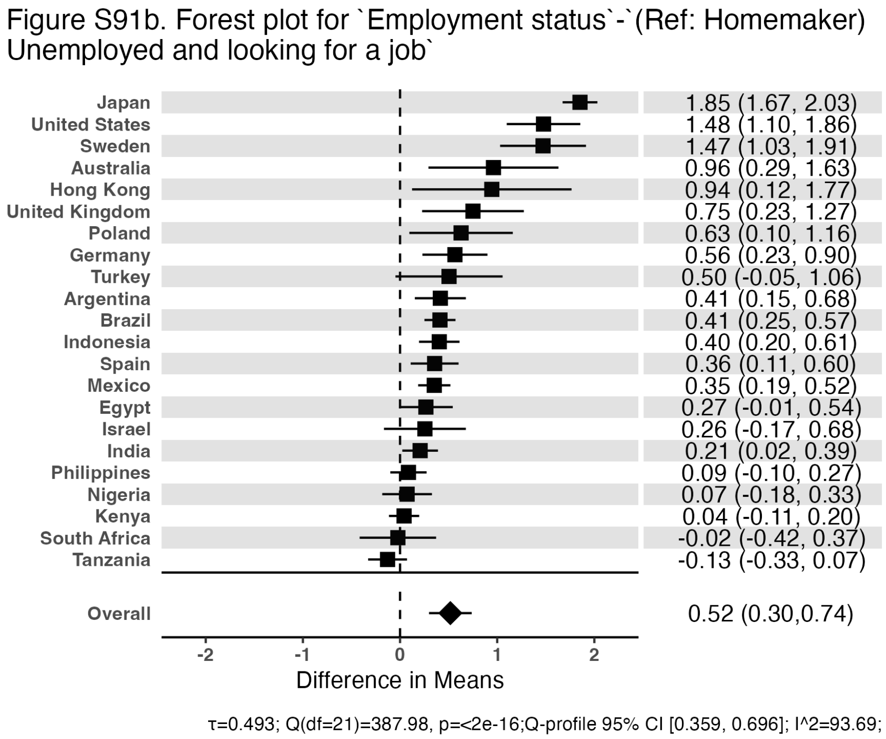

Figure S92. Heterogeneity in pairwise compairisons acrossx countries Employment status-(Ref: None of these/other) Retired. (a) Flourishing with financial indicators (12 items) [left panel]; (b) Flourishing without financial indicators (10 items) [right panel]. N=202,898, subgroup means and standard errors are computed accounting for the complex sampling design using all data simultaneously. Analyses conducted: Random-effects meta-analysis of country-specific means. Squares represent the the point estimate (mean) for each country. The lines represented the +/- 1.96*SE, standard error, around the mean; the overall pooled mean is represented by the diamond. The reported p-value for Q-statistics is necessarily 1-sided because of the use of the chi-squared distribution to test whether heterogeneity is greater than zero (i.e., a two-sided test is not applicable). No adjustments for multiple testing were made.

Figure S92a Forest plot for `Employment status`-`(Ref: None of these/ot Retired`

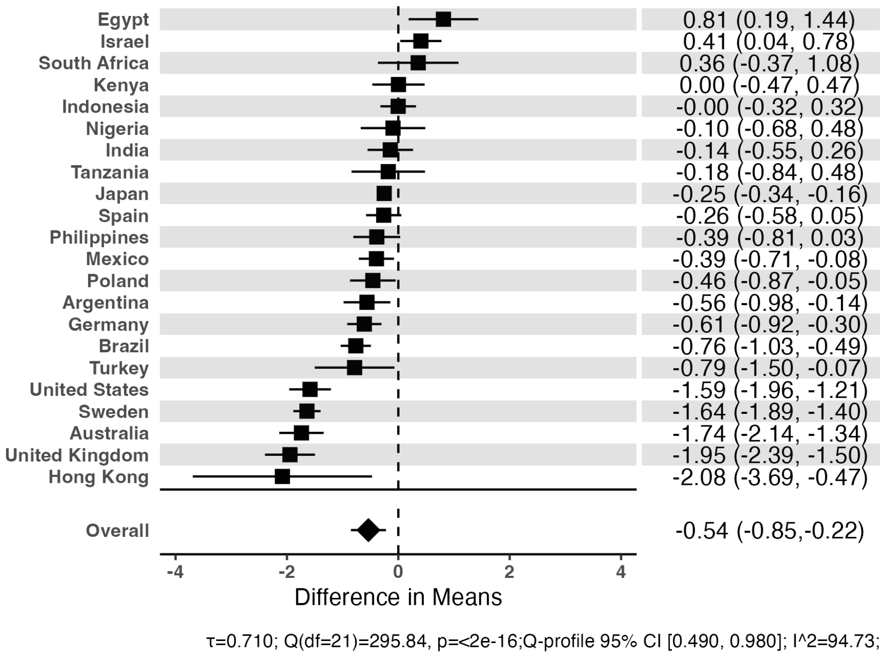

Figure S92b. Forest plot for `Employment status`-`(Ref: None of these/other) Retired`

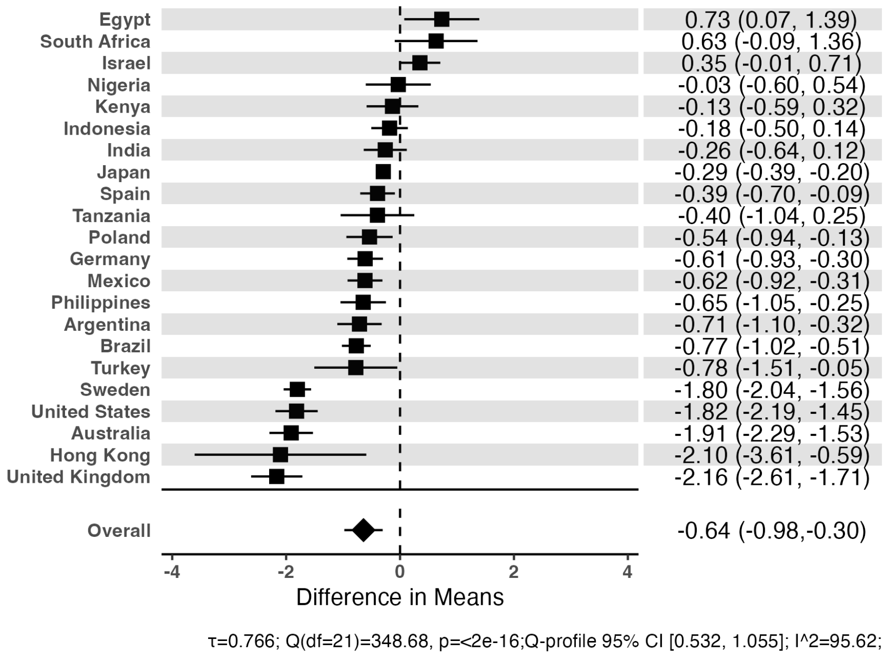

Figure S93. Heterogeneity in pairwise compairisons acrossx countries Employment status-(Ref: None of these/other) Self-employed. (a) Flourishing with financial indicators (12 items) [left panel]; (b) Flourishing without financial indicators (10 items) [right panel]. N=202,898, subgroup means and standard errors are computed accounting for the complex sampling design using all data simultaneously. Analyses conducted: Random-effects meta-analysis of country-specific means. Squares represent the the point estimate (mean) for each country. The lines represented the +/-1.96*SE, standard error, around the mean; the overall pooled mean is represented by the diamond. The reported p-value for Q-statistics is necessarily 1-sided because of the use of the chi-squared distribution to test whether heterogeneity is greater than zero (i.e., a two-sided test is not applicable). No adjustments for multiple testing were made.

Figure S93a Forest plot for `Employment status`-`(Ref: None of these/otl Self-employed`

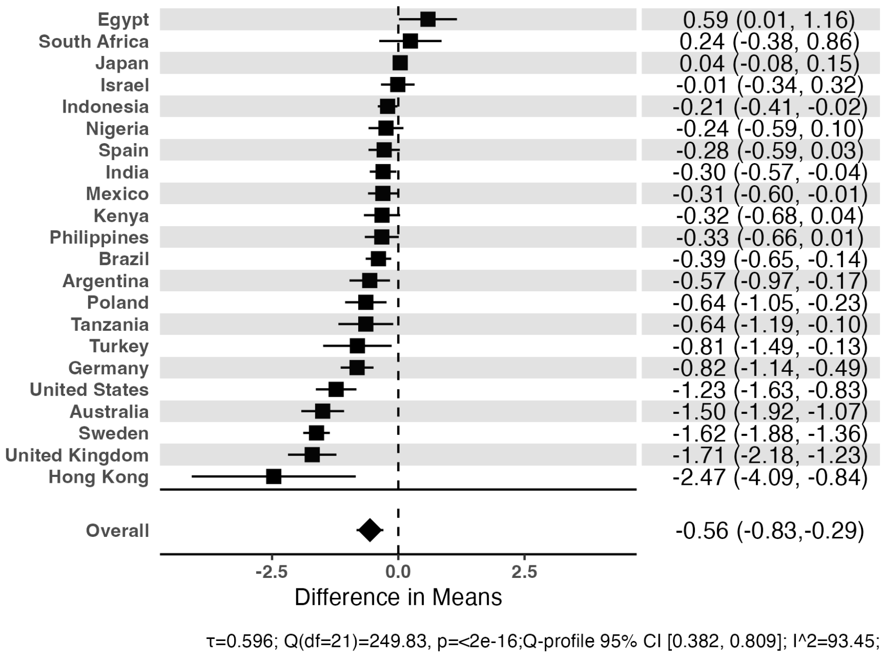

Figure S93b. Forest plot for `Employment status`-`(Ref: None of these/other) Self-employed`

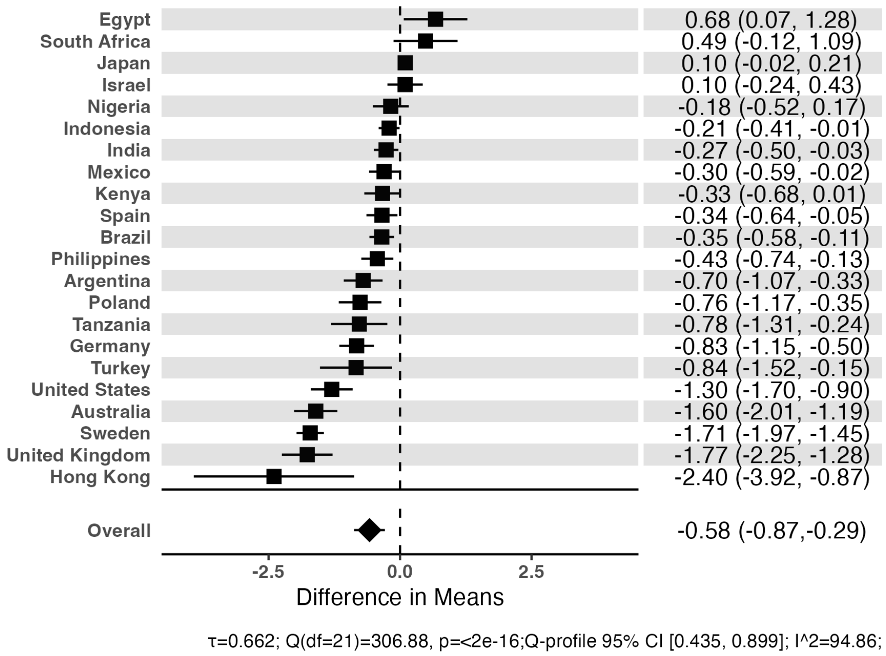

Figure S94. Heterogeneity in pairwise compairisons acrossx countries Employment status-(Ref: None of these/other) Student. (a) Flourishing with financial indicators (12 items) [left panel]; (b) Flourishing without financial indicators (10 items) [right panel]. N=202,898, subgroup means and standard errors are computed accounting for the complex sampling design using all data simultaneously. Analyses conducted: Random-effects meta-analysis of country-specific means. Squares represent the the point estimate (mean) for each country. The lines represented the +/- 1.96*SE, standard error, around the mean; the overall pooled mean is represented by the diamond. The reported p-value for Q-statistics is necessarily 1-sided because of the use of the chi-squared distribution to test whether heterogeneity is greater than zero (i.e., a two-sided test is not applicable). No adjustments for multiple testing were made.

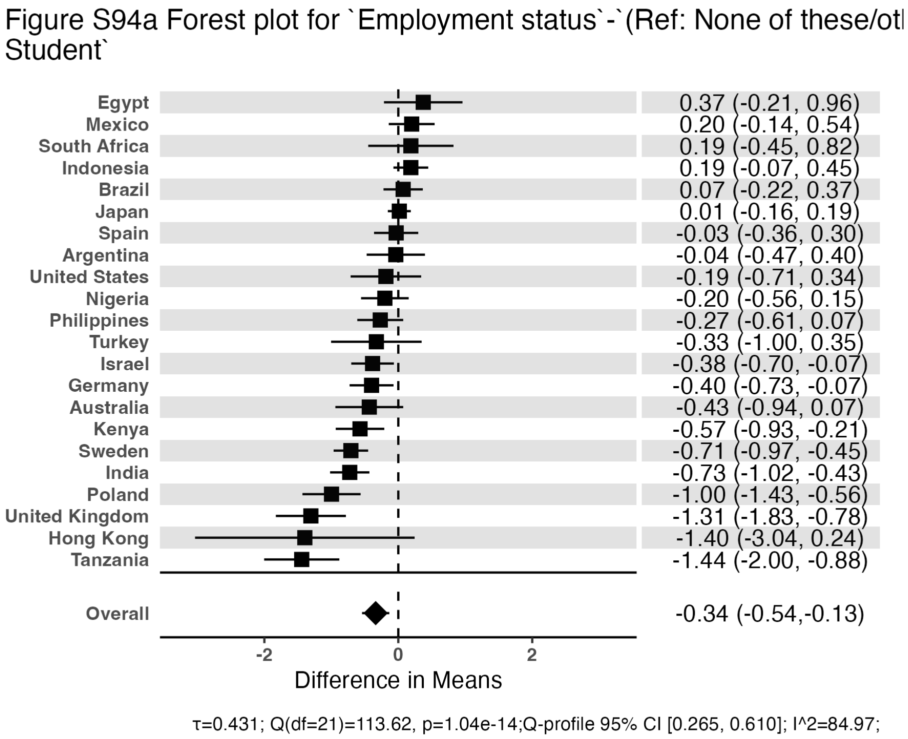

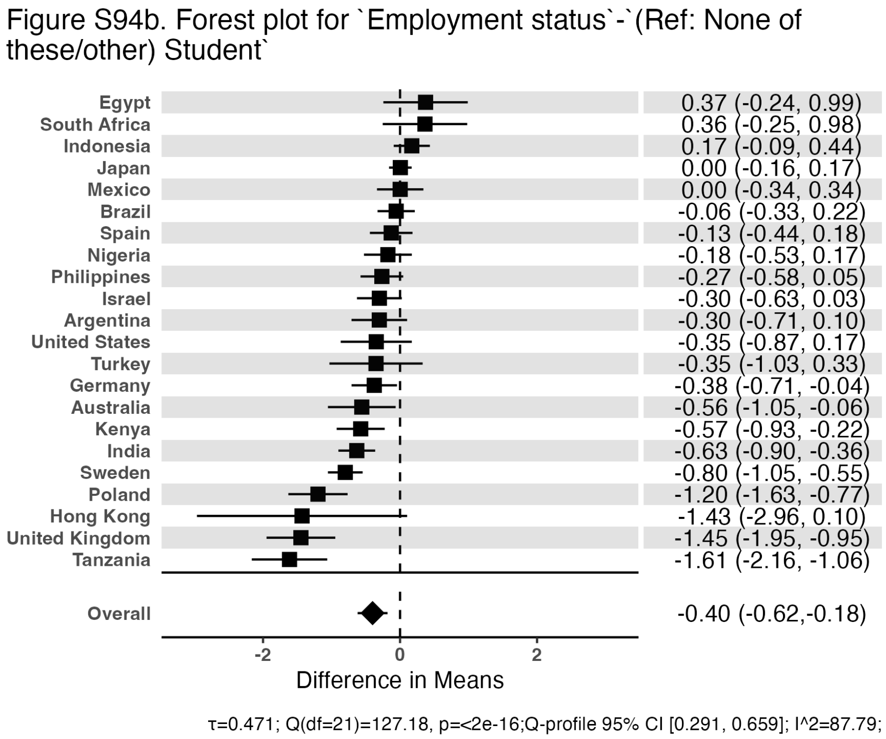

Figure S95. Heterogeneity in pairwise compairisons acrossx countries Employment status-(Ref: None of these/other) Unemployed and looking for a job. (a) Flourishing with financial indicators (12 items) [left panel]; (b) Flourishing without financial indicators (10 items) [right panel]. N=202,898, subgroup means and standard errors are computed accounting for the complex sampling design using all data simultaneously. Analyses conducted: Random-effects meta-analysis of country-specific means. Squares represent the the point estimate (mean) for each country. The lines represented the +/-1.96*SE, standard error, around the mean; the overall pooled mean is represented by the diamond. The reported p-value for Q-statistics is necessarily 1-sided because of the use of the chi-squared distribution to test whether heterogeneity is greater than zero (i.e., a two-sided test is not applicable). No adjustments for multiple testing were made.

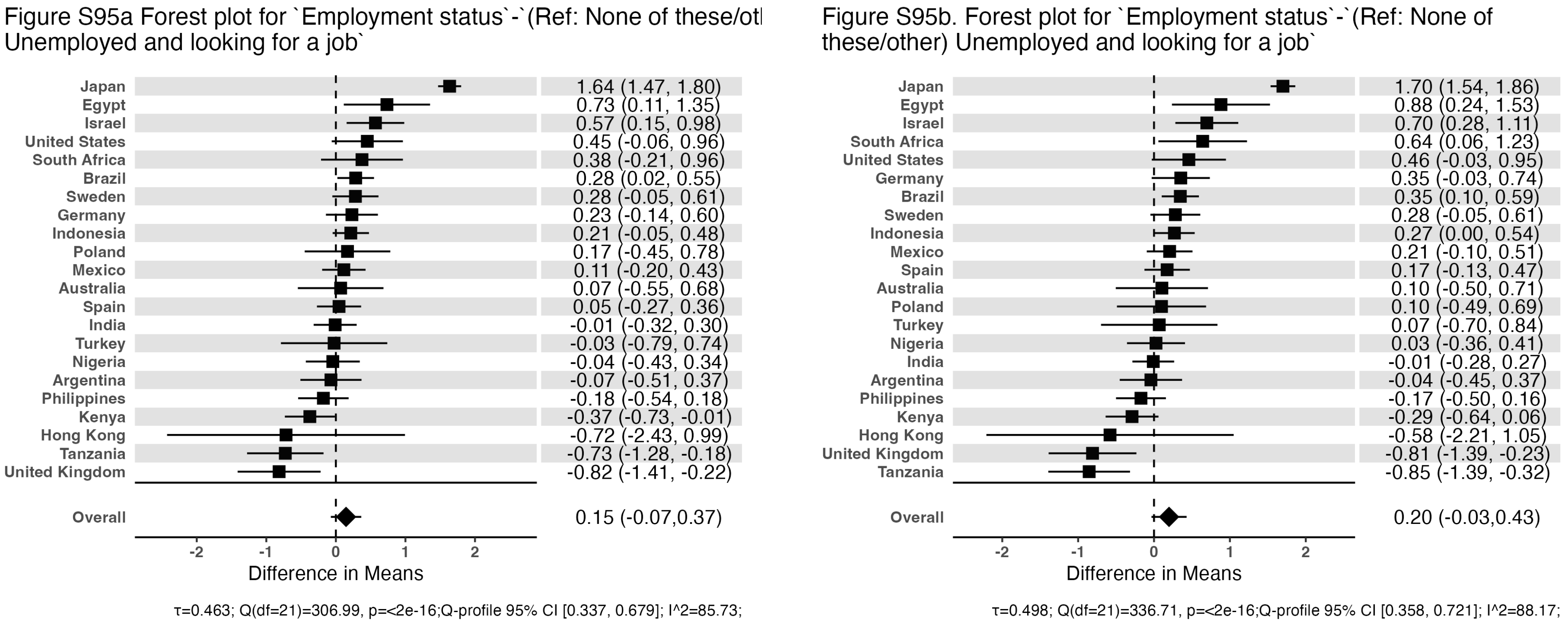

Figure S96. Heterogeneity in pairwise compairisons acrossx countries Employment status-(Ref: Retired) Self-employed. (a) Flourishing with financial indicators (12 items) [left panel]; (b) Flourishing without financial indicators (10 items) [right panel]. N=202,898, subgroup means and standard errors are computed accounting for the complex sampling design using all data simultaneously. Analyses conducted: Random-effects meta-analysis of country-specific means. Squares represent the the point estimate (mean) for each country. The lines represented the +/-1.96*SE, standard error, around the mean; the overall pooled mean is represented by the diamond. The reported p-value for Q-statistics is necessarily 1-sided because of the use of the chi-squared distribution to test whether heterogeneity is greater than zero (i.e., a two-sided test is not applicable). No adjustments for multiple testing were made.

Figure S96a Forest plot for `Employment status`-`(Ref: Retired) Self-employed`

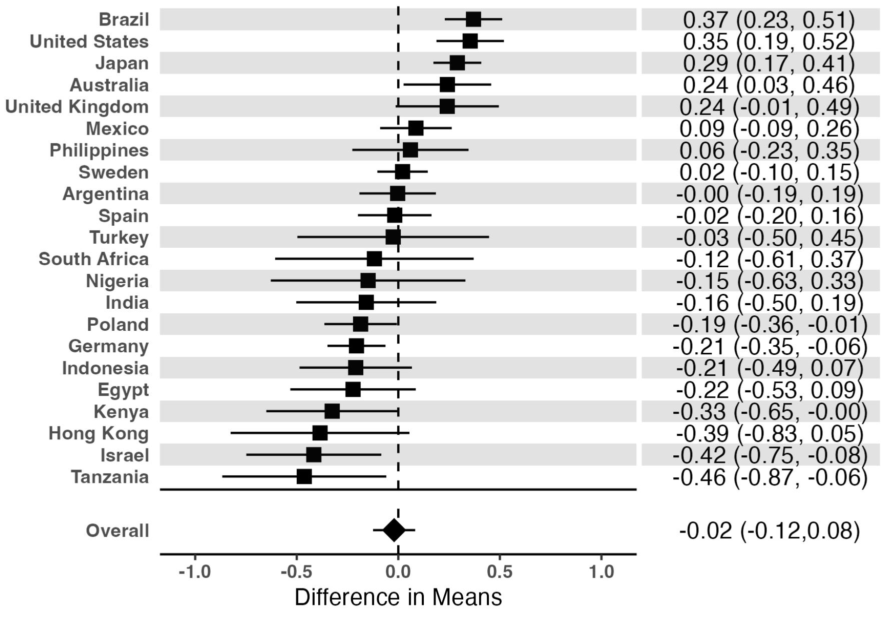

τ=0.207; Q(df=21)=105.54, p=2.99e-13;Q-profile 95% CI [0.138, 0.313]; I^2=78.93;

Figure S96b. Forest plot for `Employment status`-`(Ref: Retired) Self-employed`

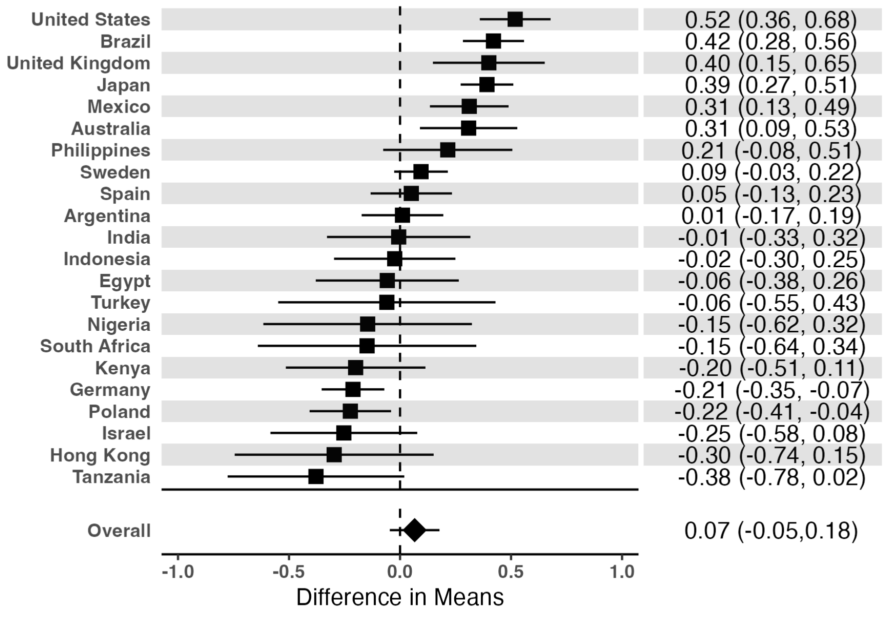

τ=0.229; Q(df=21)=134.58, p=<2e-16;Q-profile 95% CI [0.159, 0.342]; I^2=82.33;

Figure S97. Heterogeneity in pairwise compairisons acrossx countries Employment status-(Ref: Retired) Student. (a) Flourishing with financial indicators (12 items) [left panel]; (b) Flourishing without financial indicators (10 items) [right panel]. N=202,898, subgroup means and standard errors are computed accounting for the complex sampling design using all data simultaneously. Analyses conducted: Random-effects meta-analysis of country-specific means. Squares represent the the point estimate (mean) for each country. The lines represented the +/-1.96*SE, standard error, around the mean; the overall pooled mean is represented by the diamond. The reported p-value for Q-statistics is necessarily 1-sided because of the use of the chi-squared distribution to test whether heterogeneity is greater than zero (i.e., a two-sided test is not applicable). No adjustments for multiple testing were made.

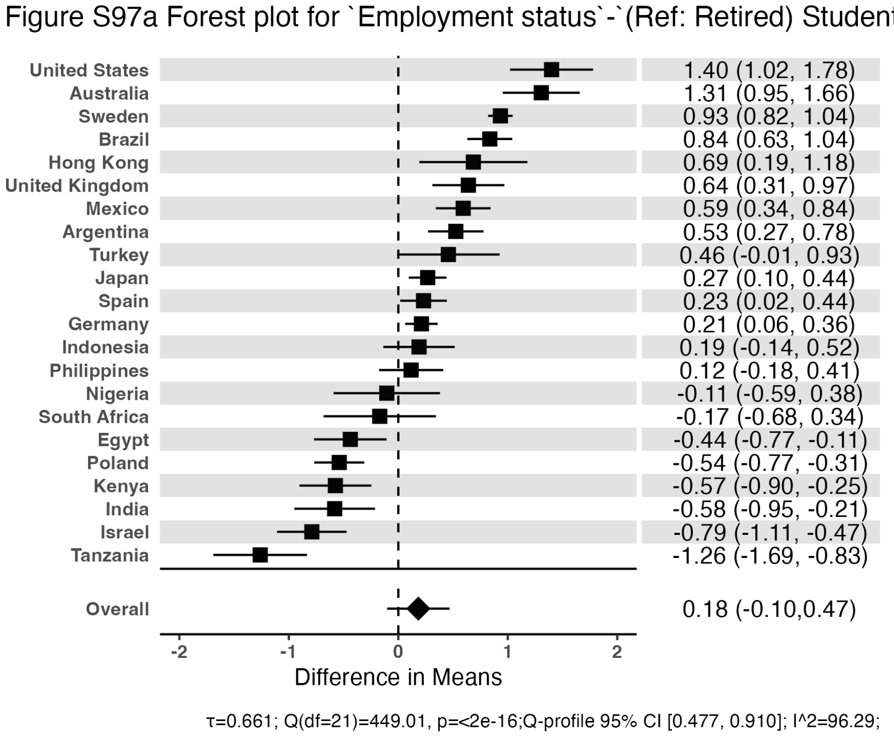

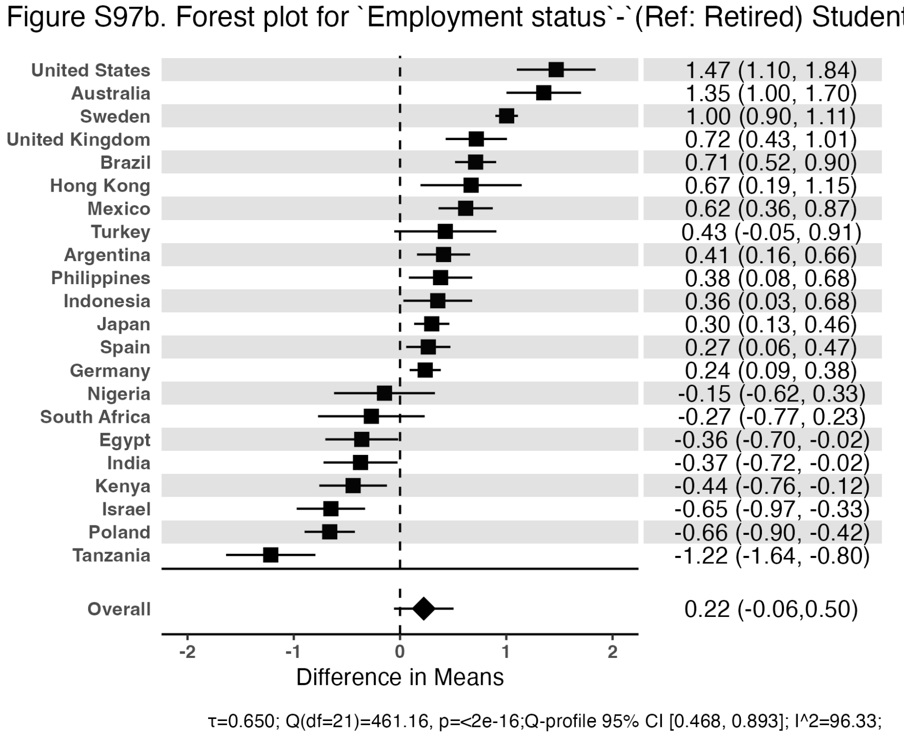

Figure S98. Heterogeneity in pairwise compairisons acrossx countries Employment status-(Ref: Retired) Unemployed and looking for a job. (a) Flourishing with financial indicators (12 items) [left panel]; (b) Flourishing without financial indicators (10 items) [right panel]. N=202,898, subgroup means and standard errors are computed accounting for the complex sampling design using all data simultaneously. Analyses conducted: Random-effects meta-analysis of country-specific means. Squares represent the the point estimate (mean) for each country. The lines represented the +/-1.96*SE, standard error, around the mean; the overall pooled mean is represented by the diamond. The reported p-value for Q-statistics is necessarily 1-sided because of the use of the chi-squared distribution to test whether heterogeneity is greater than zero (i.e., a two-sided test is not applicable). No adjustments for multiple testing were made.

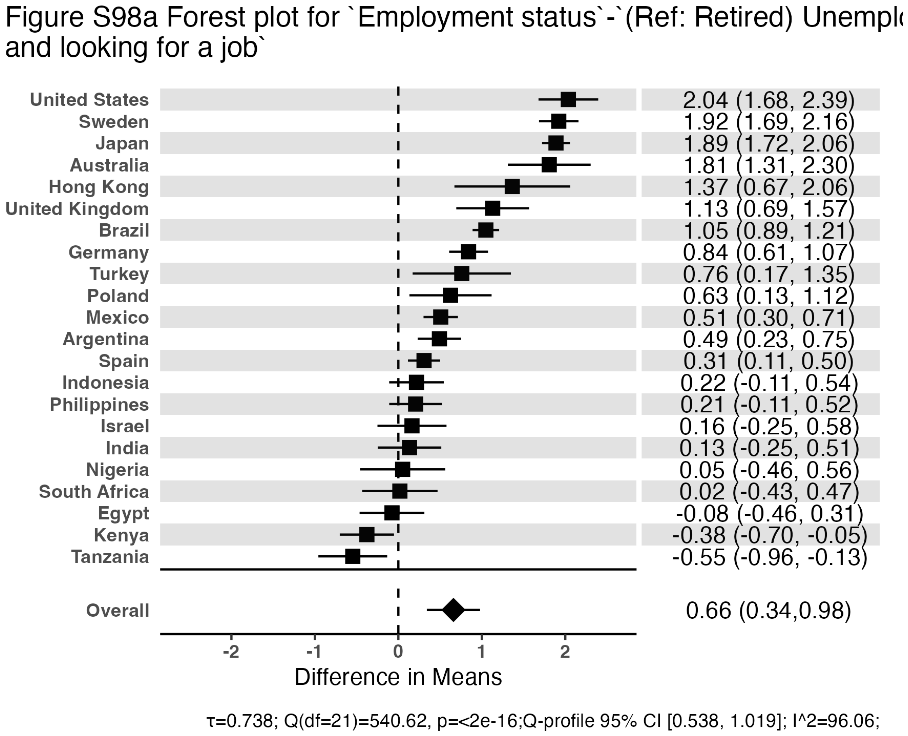

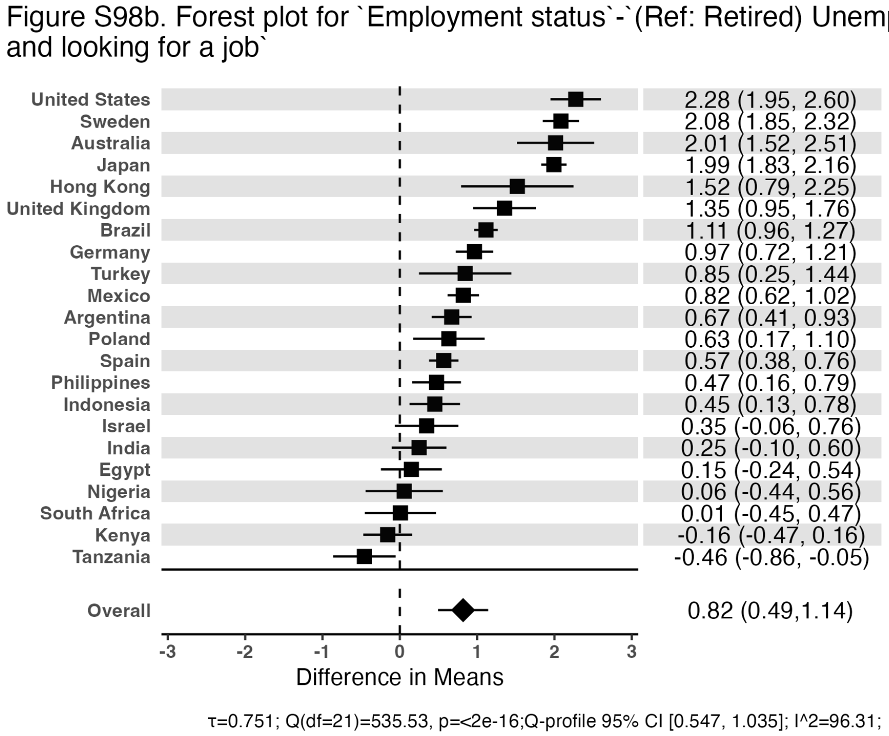

Figure S99. Heterogeneity in pairwise compairisons acrossx countries Employment status-(Ref: Self-employed) Student. (a) Flourishing with financial indicators (12 items) [left panel]; (b) Flourishing without financial indicators (10 items) [right panel]. N=202,898, subgroup means and standard errors are computed accounting for the complex sampling design using all data simultaneously. Analyses conducted: Random-effects meta-analysis of country-specific means. Squares represent the the point estimate (mean) for each country. The lines represented the +/-1.96*SE, standard error, around the mean; the overall pooled mean is represented by the diamond. The reported p-value for Q-statistics is necessarily 1-sided because of the use of the chi-squared distribution to test whether heterogeneity is greater than zero (i.e., a two-sided test is not applicable). No adjustments for multiple testing were made.

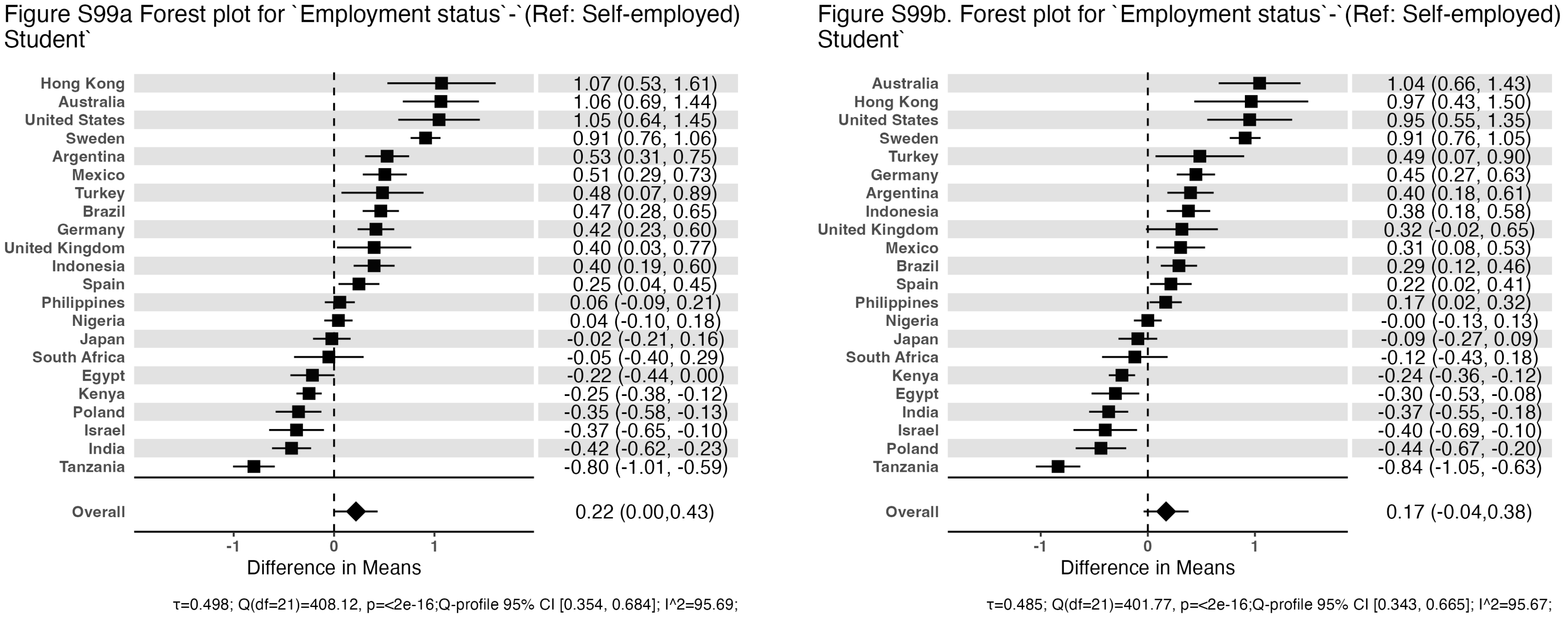

Figure S100. Heterogeneity in pairwise compairisons acrossx countries Employment status-(Ref: Self-employed) Unemployed and looking for a job. (a) Flourishing with financial indicators (12 items) [left panel]; (b) Flourishing without financial indicators (10 items) [right panel]. N=202,898, subgroup means and standard errors are computed accounting for the complex sampling design using all data simultaneously. Analyses conducted: Random-effects meta-analysis of country-specific means. Squares represent the the point estimate (mean) for each country. The lines represented the +/-1.96*SE, standard error, around the mean; the overall pooled mean is represented by the diamond. The reported p-value for Q-statistics is necessarily 1-sided because of the use of the chi-squared distribution to test whether heterogeneity is greater than zero (i.e., a two-sided test is not applicable). No adjustments for multiple testing were made.

Figure S100a Forest plot for `Employment status`-`(Ref: Self-employed) Unemployed and looking for a job`

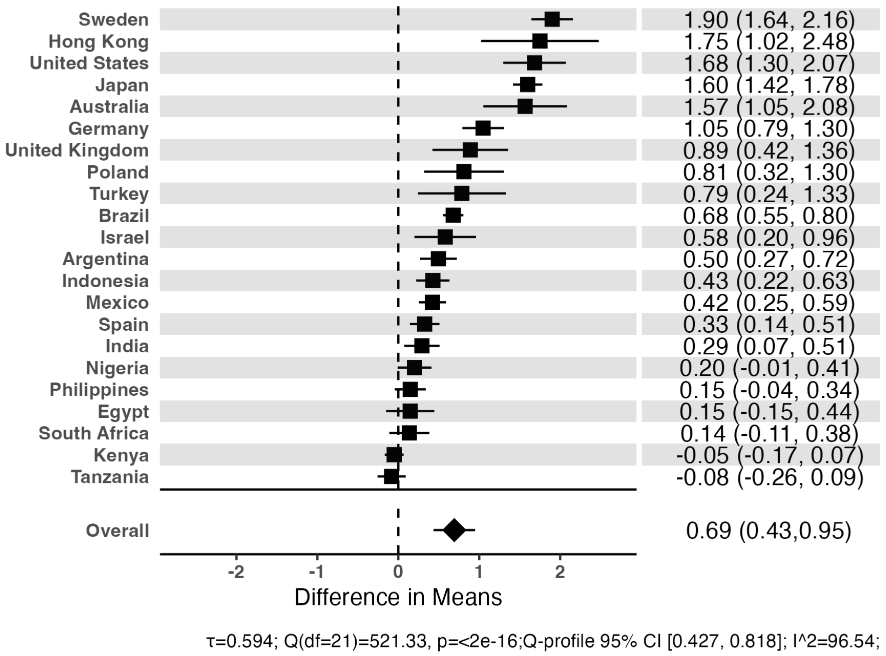

Figure S100b. Forest plot for `Employment status`-`(Ref: Self-employed) Unemployed and looking for a job`

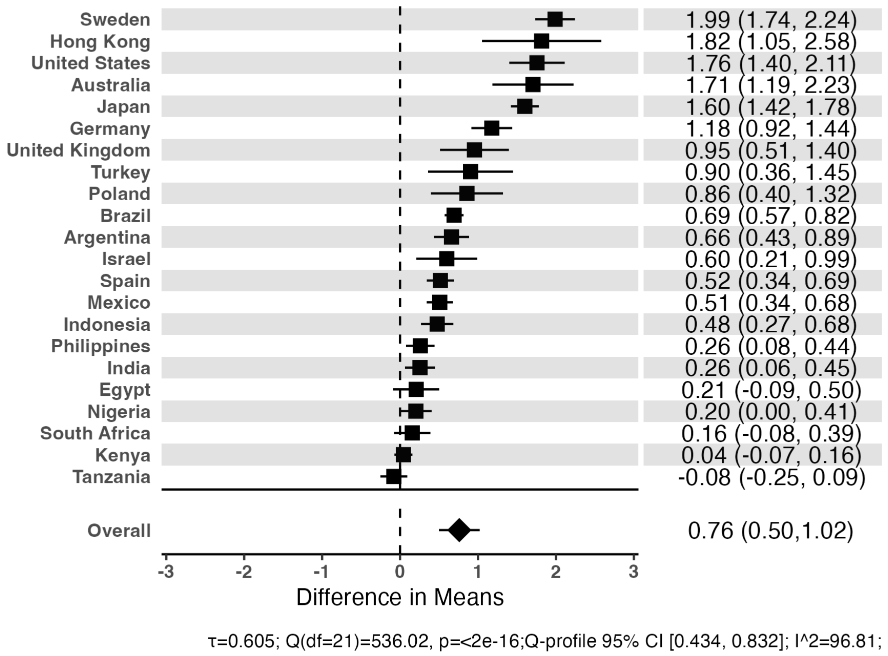

Figure S101. Heterogeneity in pairwise compairisons acrossx countries Employment status-(Ref: Student) Unemployed and looking for a job. (a) Flourishing with financial indicators (12 items) [left panel]; (b) Flourishing without financial indicators (10 items) [right panel]. N=202,898, subgroup means and standard errors are computed accounting for the complex sampling design using all data simultaneously. Analyses conducted: Random-effects meta-analysis of country-specific means. Squares represent the the point estimate (mean) for each country. The lines represented the +/-1.96*SE, standard error, around the mean; the overall pooled mean is represented by the diamond. The reported p-value for Q-statistics is necessarily 1-sided because of the use of the chi-squared distribution to test whether heterogeneity is greater than zero (i.e., a two-sided test is not applicable). No adjustments for multiple testing were made.

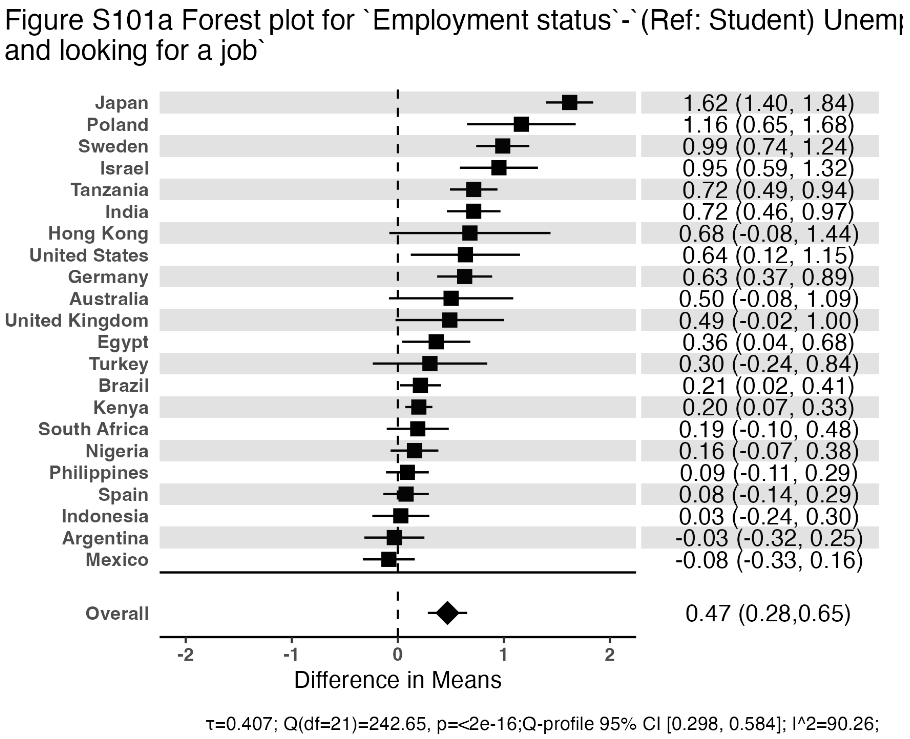

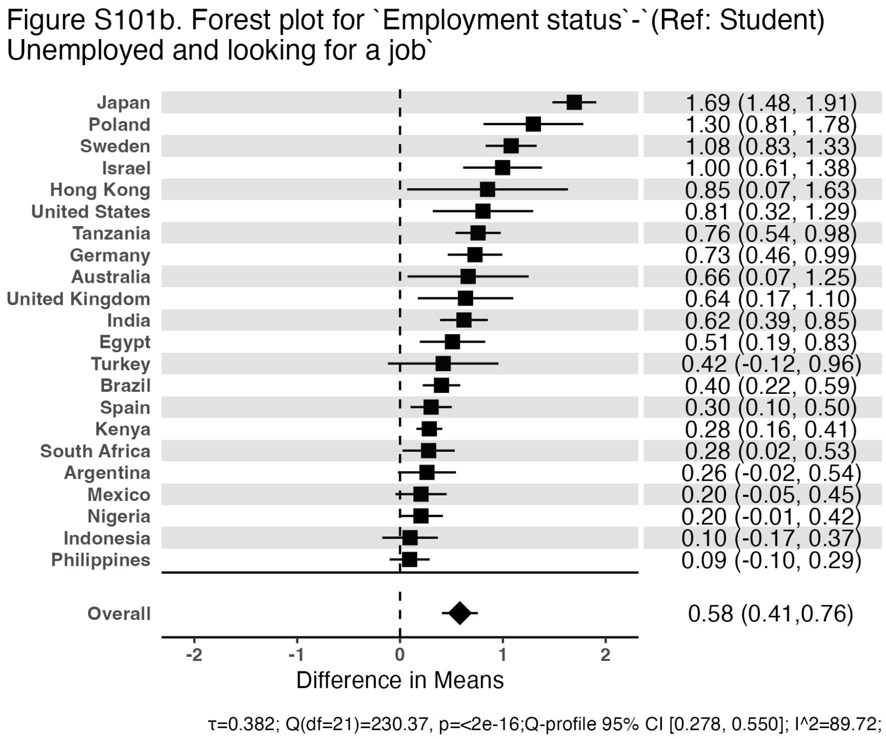

Figure S102. Heterogeneity in pairwise compairisons acrossx countries Religious service attendance-(Ref: A few times a year) >1/week. (a) Flourishing with financial indicators (12 items) [left panel]; (b) Flourishing without financial indicators (10 items) [right panel]. N=202,898, subgroup means and standard errors are computed accounting for the complex sampling design using all data simultaneously. Analyses conducted: Random-effects meta-analysis of country-specific means. Squares represent the the point estimate (mean) for each country. The lines represented the +/-1.96*SE, standard error, around the mean; the overall pooled mean is represented by the diamond. The reported p-value for Q-statistics is necessarily 1-sided because of the use of the chi-squared distribution to test whether heterogeneity is greater than zero (i.e., a two-sided test is not applicable). No adjustments for multiple testing were made.

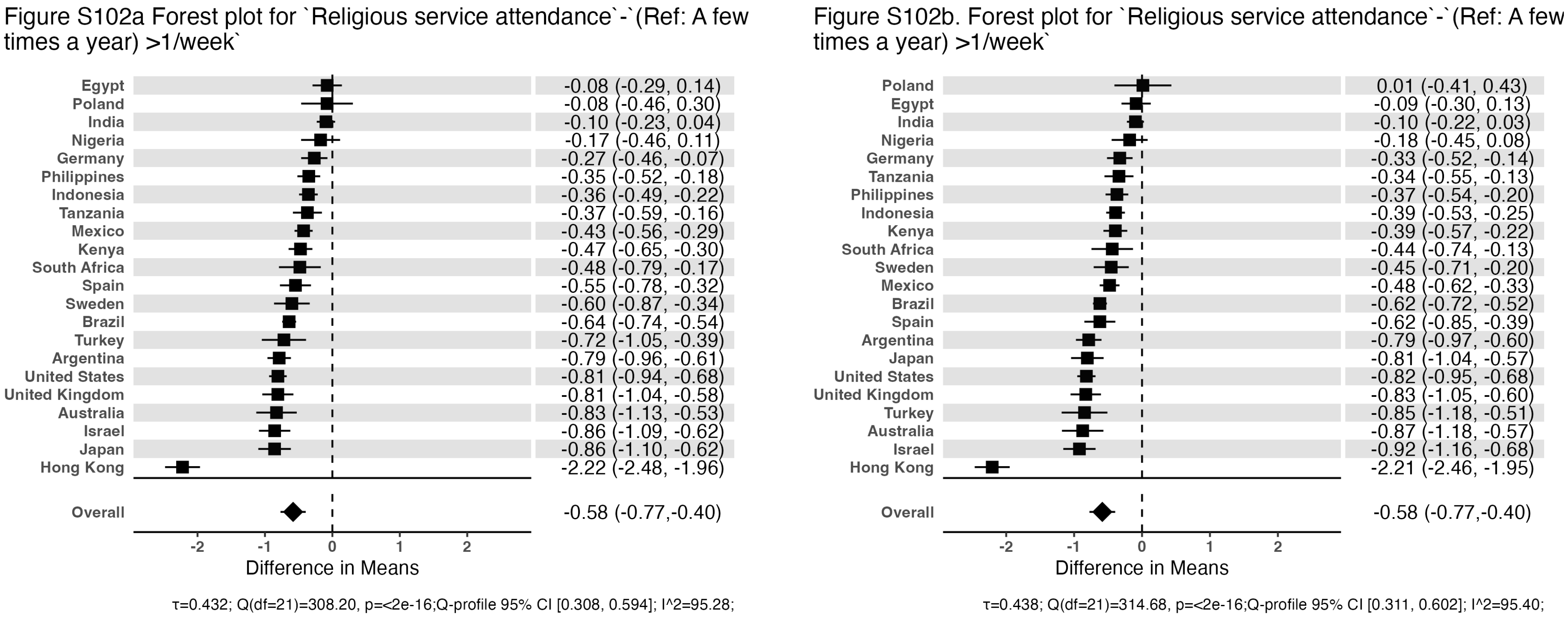

Figure S103. Heterogeneity in pairwise compairisons acrossx countries Religious service attendance-(Ref: A few times a year) Never. (a) Flourishing with financial indicators (12 items) [left panel]; (b) Flourishing without financial indicators (10 items) [right panel]. N=202,898, subgroup means and standard errors are computed accounting for the complex sampling design using all data simultaneously. Analyses conducted: Random-effects meta-analysis of country-specific means. Squares represent the the point estimate (mean) for each country. The lines represented the +/-1.96*SE, standard error, around the mean; the overall pooled mean is represented by the diamond. The reported p-value for Q-statistics is necessarily 1-sided because of the use of the chi-squared distribution to test whether heterogeneity is greater than zero (i.e., a two-sided test is not applicable). No adjustments for multiple testing were made.

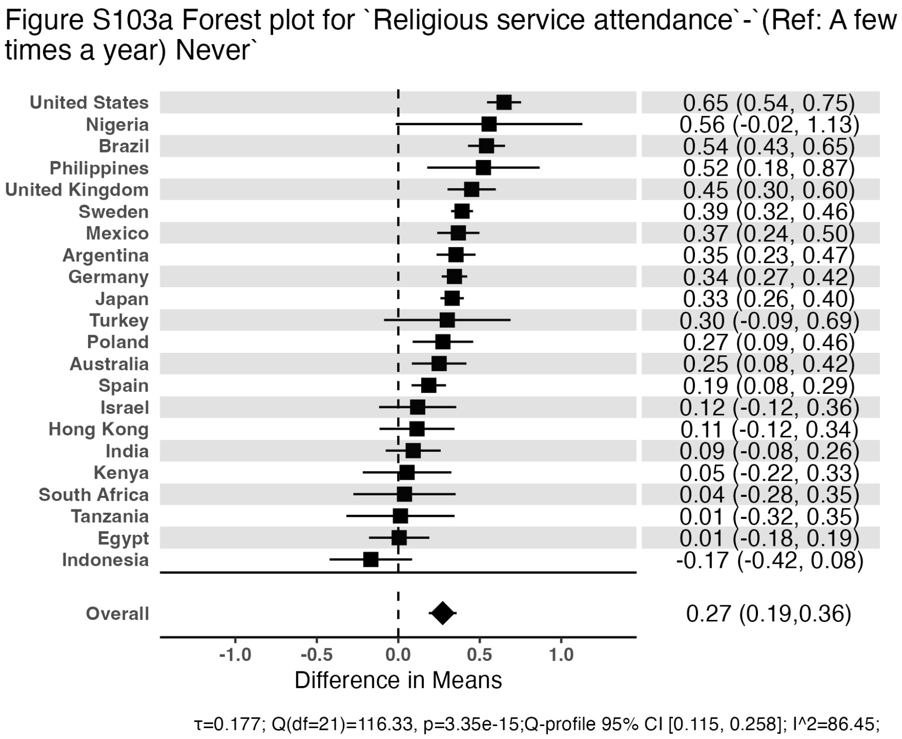

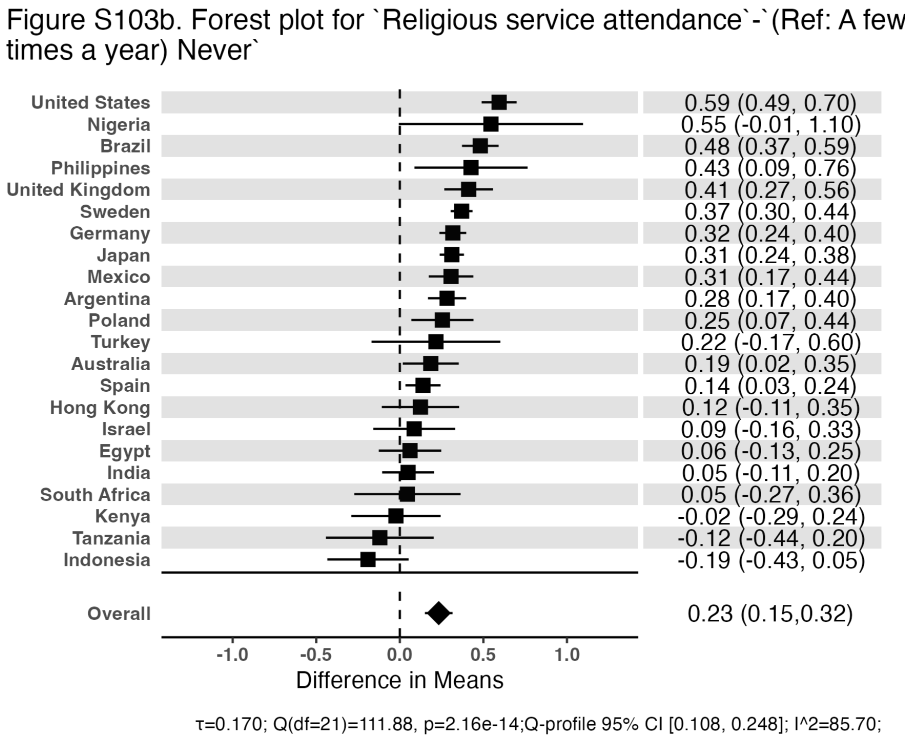

Figure S104. Heterogeneity in pairwise compairisons acrossx countries Religious service attendance-(Ref: A few times a year) 1/week. (a) Flourishing with financial indicators (12 items) [left panel]; (b) Flourishing without financial indicators (10 items) [right panel]. N=202,898, subgroup means and standard errors are computed accounting for the complex sampling design using all data simultaneously. Analyses conducted: Random-effects meta-analysis of country-specific means. Squares represent the the point estimate (mean) for each country. The lines represented the +/-1.96*SE, standard error, around the mean; the overall pooled mean is represented by the diamond. The reported p-value for Q-statistics is necessarily 1-sided because of the use of the chi-squared distribution to test whether heterogeneity is greater than zero (i.e., a two-sided test is not applicable). No adjustments for multiple testing were made.

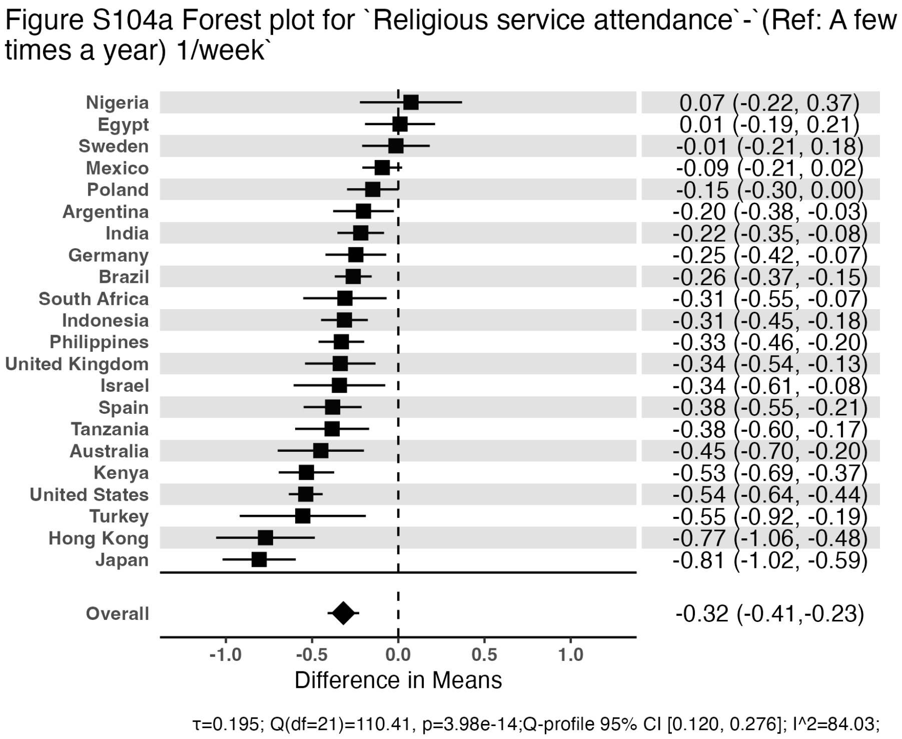

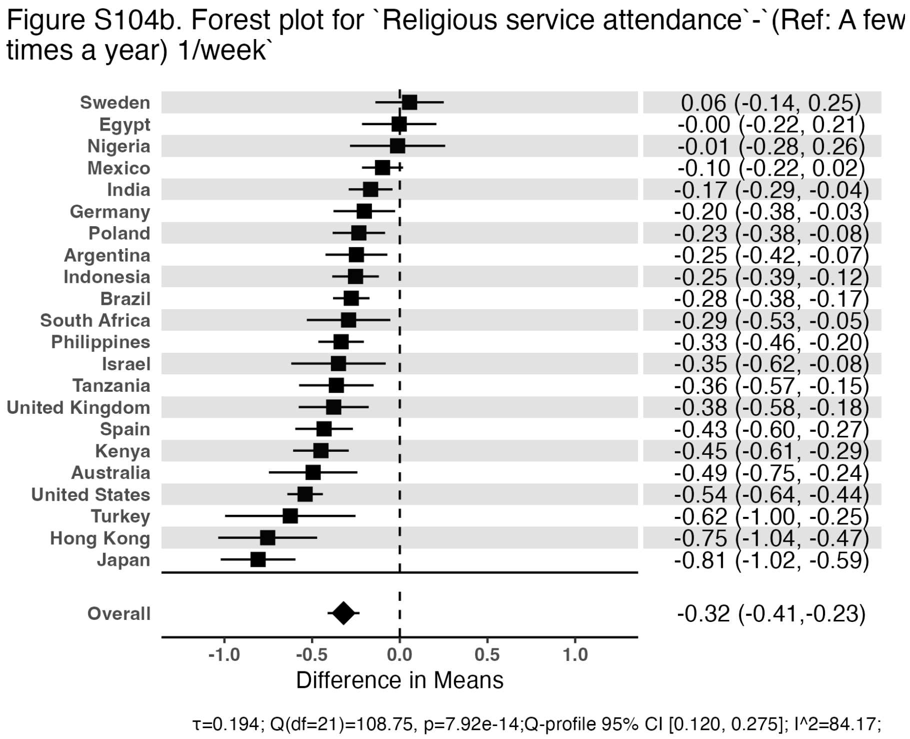

Figure S105. Heterogeneity in pairwise compairisons acrossx countries Religious service attendance-(Ref: A few times a year) 1-3/month. (a) Flourishing with financial indicators (12 items) [left panel]; (b) Flourishing without financial indicators (10 items) [right panel]. N=202,898, subgroup means and standard errors are computed accounting for the complex sampling design using all data simultaneously. Analyses conducted: Random-effects meta-analysis of country-specific means. Squares represent the the point estimate (mean) for each country. The lines represented the +/-1.96*SE, standard error, around the mean; the overall pooled mean is represented by the diamond. The reported p-value for Q-statistics is necessarily 1-sided because of the use of the chi-squared distribution to test whether heterogeneity is greater than zero (i.e., a two-sided test is not applicable). No adjustments for multiple testing were made.

Figure S105a Forest plot for `Religious service attendance`-`(Ref: A few times a year) 1-3/month`

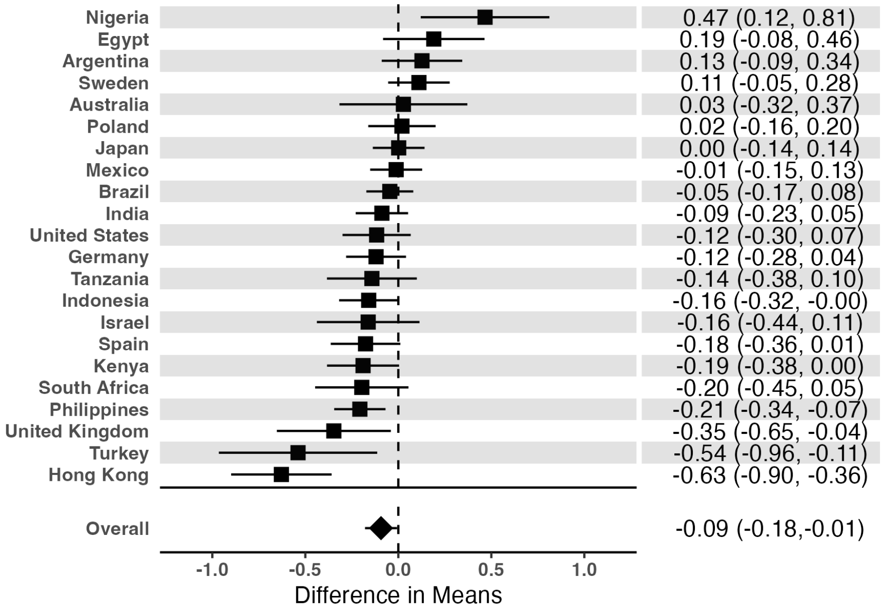

τ=0.173; Q(df=21)=57.53, p=3e-05;Q-profile 95% CI [0.045, 0.242]; I^2=76.38;

Figure S105b. Forest plot for `Religious service attendance`-`(Ref: A few times a year) 1-3/month`

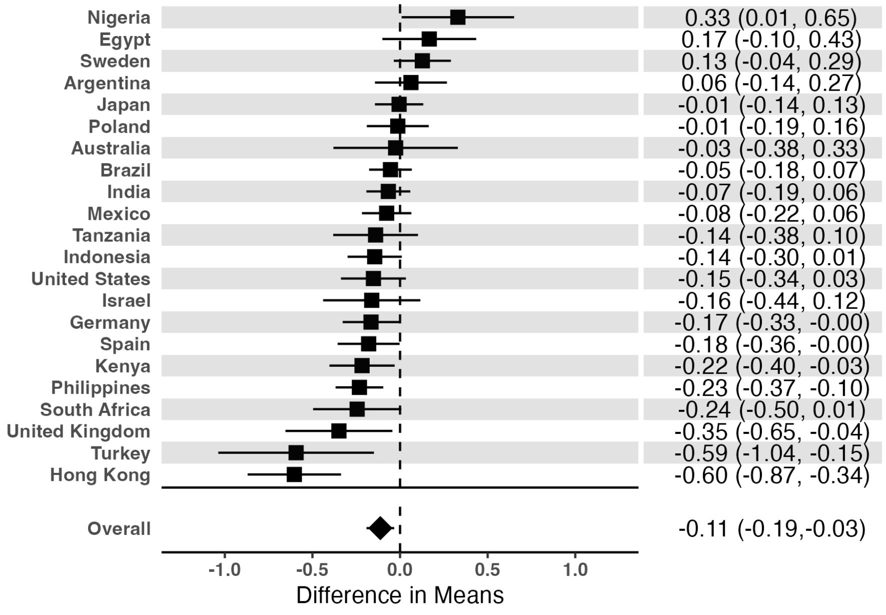

τ=0.155; Q(df=21)=54.08, p=9.62e-05;Q-profile 95% CI [0.037, 0.219]; I^2=73.06;

Figure S106. Heterogeneity in pairwise compairisons acrossx countries Religious service attendance-(Ref: >1/week) Never. (a) Flourishing with financial indicators (12 items) [left panel]; (b) Flourishing without financial indicators (10 items) [right panel]. N=202,898, subgroup means and standard errors are computed accounting for the complex sampling design using all data simultaneously. Analyses conducted: Random-effects meta-analysis of country-specific means. Squares represent the the point estimate (mean) for each country. The lines represented the +/-1.96*SE, standard error, around the mean; the overall pooled mean is represented by the diamond. The reported p-value for Q-statistics is necessarily 1-sided because of the use of the chi-squared distribution to test whether heterogeneity is greater than zero (i.e., a two-sided test is not applicable). No adjustments for multiple testing were made.

Figure S106a Forest plot for `Religious service attendance`-`(Ref: >1/we Never`

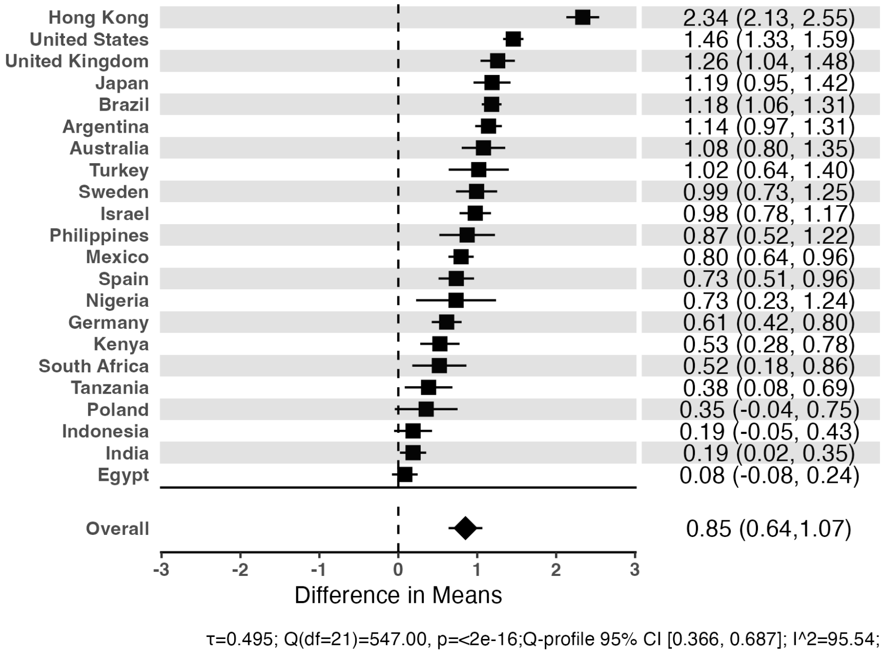

Figure S106b. Forest plot for `Religious service attendance`-`(Ref: >1/week) Never`

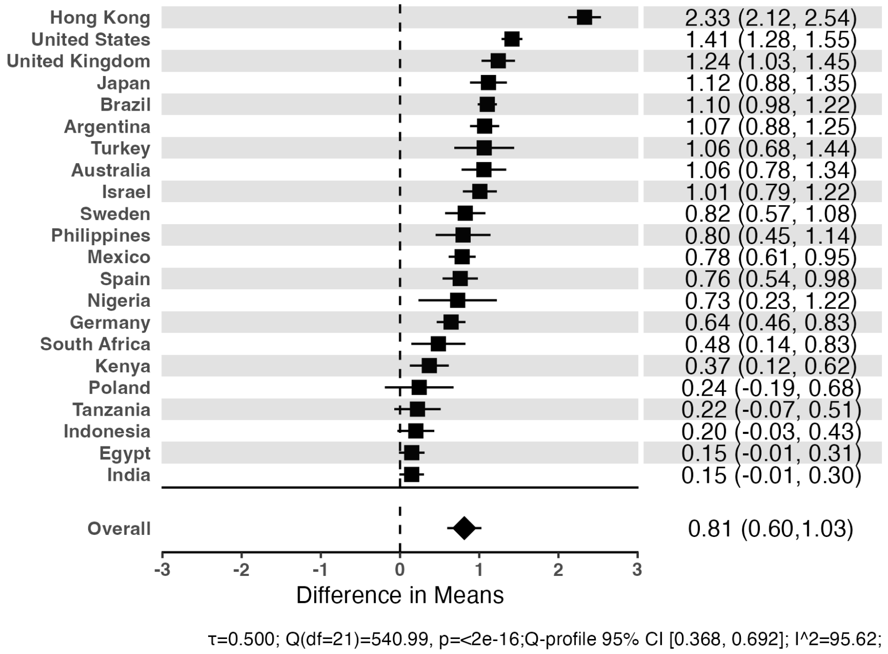

Figure S107. Heterogeneity in pairwise compairisons acrossx countries Religious service attendance-(Ref: >1/week) 1/week. (a) Flourishing with financial indicators (12 items) [left panel]; (b) Flourishing without financial indicators (10 items) [right panel]. N=202,898, subgroup means and standard errors are computed accounting for the complex sampling design using all data simultaneously. Analyses conducted: Random-effects meta-analysis of country-specific means. Squares represent the the point estimate (mean) for each country. The lines represented the +/-1.96*SE, standard error, around the mean; the overall pooled mean is represented by the diamond. The reported p-value for Q-statistics is necessarily 1-sided because of the use of the chi-squared distribution to test whether heterogeneity is greater than zero (i.e., a two-sided test is not applicable). No adjustments for multiple testing were made.

Figure S107a Forest plot for `Religious service attendance`-`(Ref: >1/we 1/week`

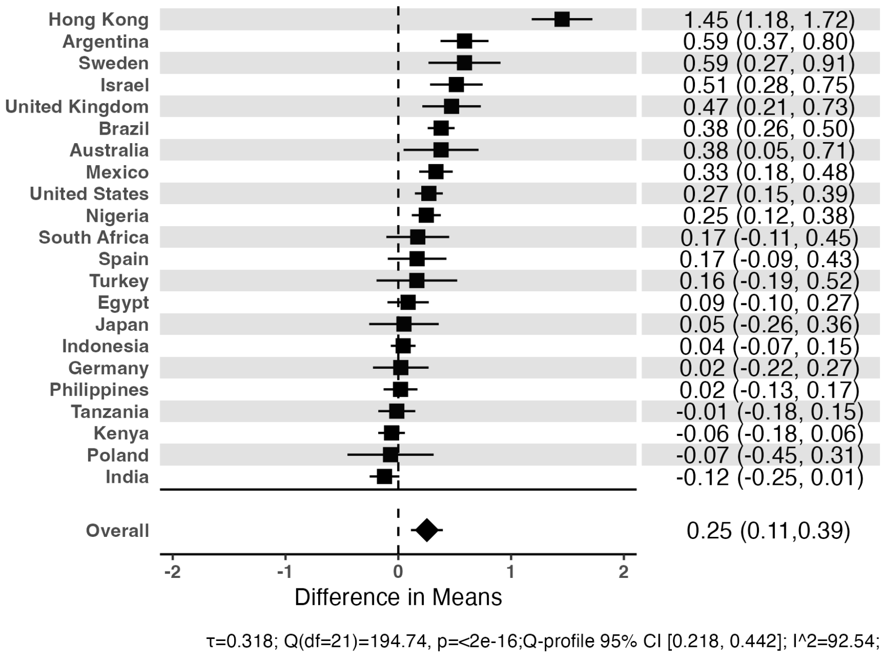

Figure S107b. Forest plot for `Religious service attendance`-`(Ref: >1/week) 1/week`

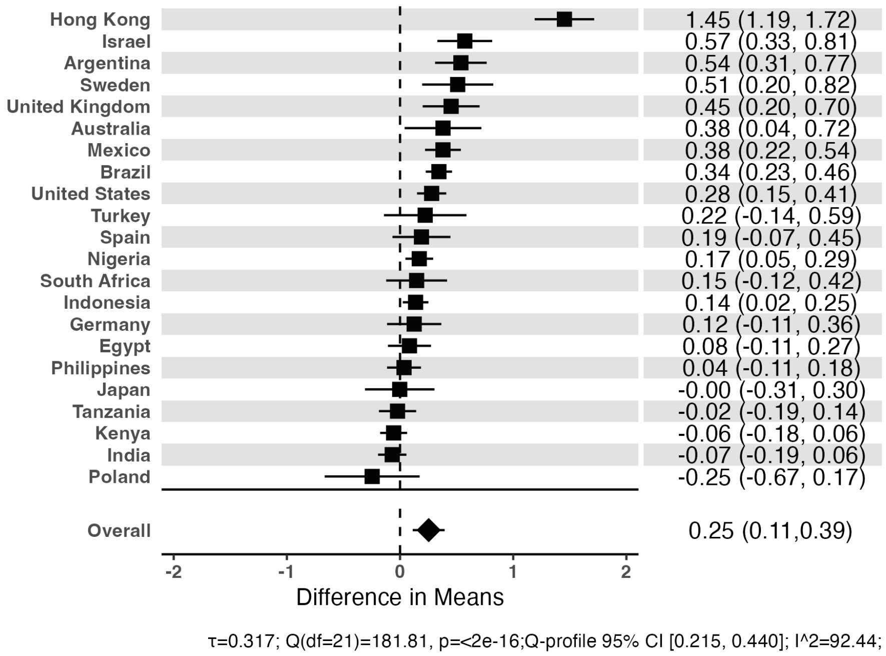

Figure S108. Heterogeneity in pairwise compairisons acrossx countries Religious service attendance-(Ref: >1/week) 1-3/month. (a) Flourishing with financial indicators (12 items) [left panel]; (b) Flourishing without financial indicators (10 items) [right panel]. N=202,898, subgroup means and standard errors are computed accounting for the complex sampling design using all data simultaneously. Analyses conducted: Random-effects meta-analysis of country-specific means. Squares represent the the point estimate (mean) for each country. The lines represented the +/- 1.96*SE, standard error, around the mean; the overall pooled mean is represented by the diamond. The reported p-value for Q-statistics is necessarily 1-sided because of the use of the chi-squared distribution to test whether heterogeneity is greater than zero (i.e., a two-sided test is not applicable). No adjustments for multiple testing were made.

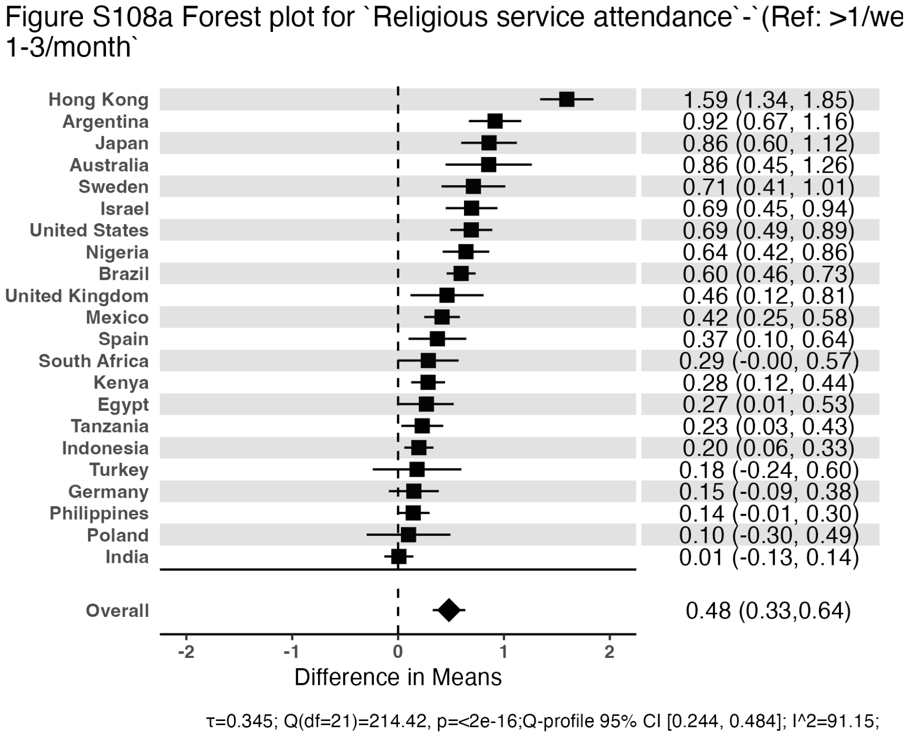

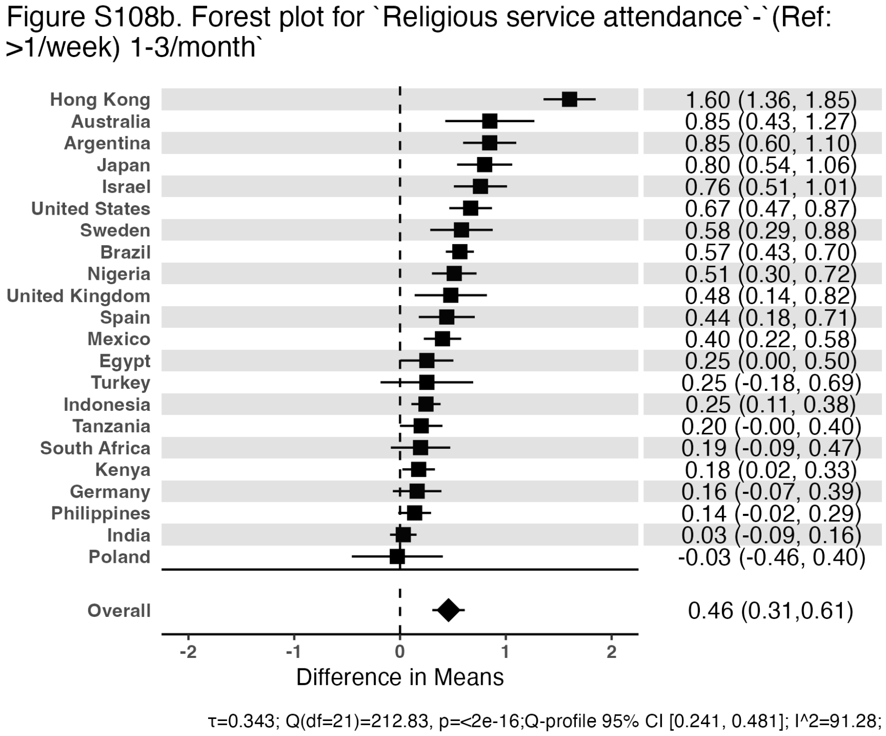

Figure S109. Heterogeneity in pairwise compairisons acrossx countries Religious service attendance-(Ref: Never) 1/week. (a) Flourishing with financial indicators (12 items) [left panel]; (b) Flourishing without financial indicators (10 items) [right panel]. N=202,898, subgroup means and standard errors are computed accounting for the complex sampling design using all data simultaneously. Analyses conducted: Random-effects meta-analysis of country-specific means. Squares represent the the point estimate (mean) for each country. The lines represented the +/-1.96*SE, standard error, around the mean; the overall pooled mean is represented by the diamond. The reported p-value for Q-statistics is necessarily 1-sided because of the use of the chi-squared distribution to test whether heterogeneity is greater than zero (i.e., a two-sided test is not applicable). No adjustments for multiple testing were made.

Figure S109a Forest plot for `Religious service attendance`-`(Ref: Never 1/week`

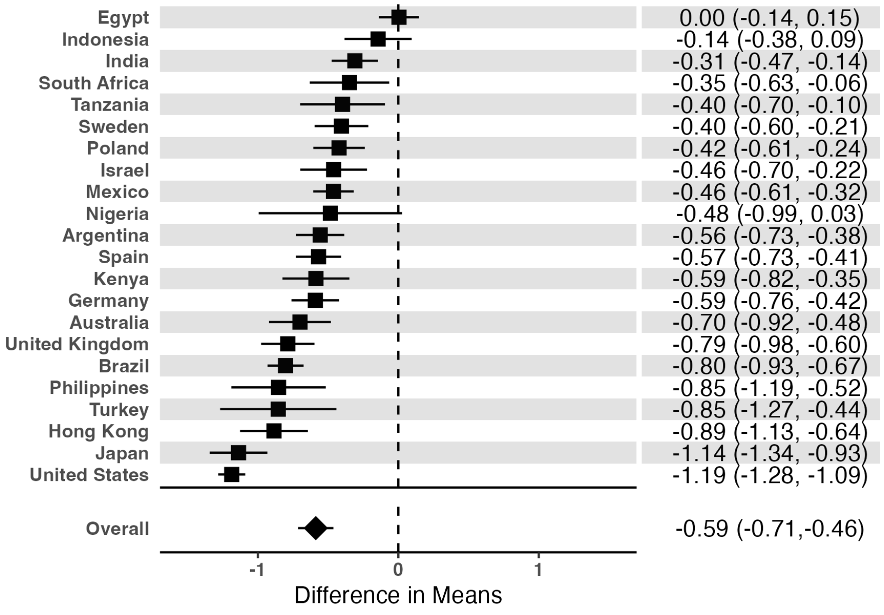

τ=0.276; Q(df=21)=300.52, p=<2e-16;Q-profile 95% CI [0.202, 0.395]; I^2=89.37;

Figure S109b. Forest plot for `Religious service attendance`-`(Ref: Neve 1/week`

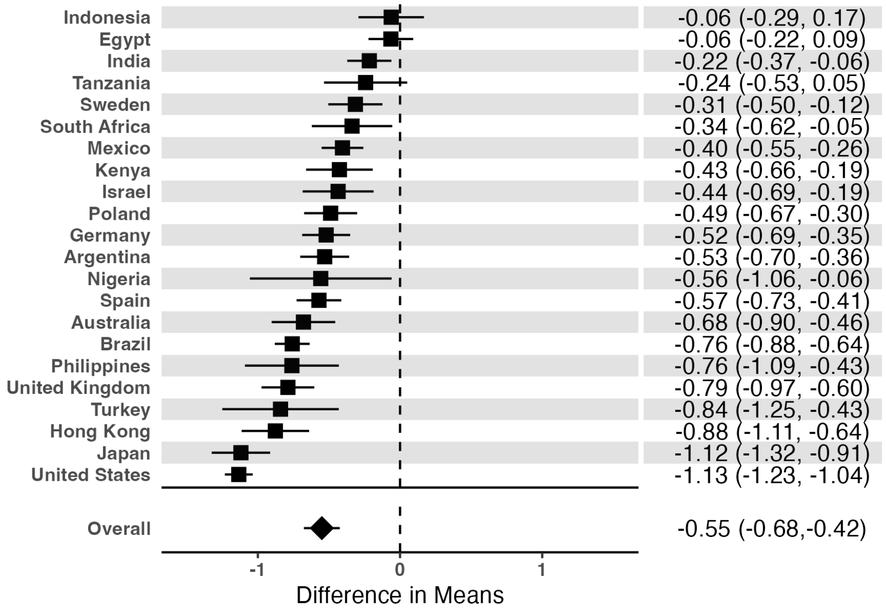

τ=0.278; Q(df=21)=277.24, p=<2e-16;Q-profile 95% CI [0.202, 0.397]; I^2=89.57;

Figure S110. Heterogeneity in pairwise compairisons acrossx countries Religious service attendance-(Ref: Never) 1-3/month. (a) Flourishing with financial indicators (12 items) [left panel]; (b) Flourishing without financial indicators (10 items) [right panel]. N=202,898, subgroup means and standard errors are computed accounting for the complex sampling design using all data simultaneously. Analyses conducted: Random-effects meta-analysis of country-specific means. Squares represent the the point estimate (mean) for each country. The lines represented the +/- 1.96*SE, standard error, around the mean; the overall pooled mean is represented by the diamond. The reported p-value for Q-statistics is necessarily 1-sided because of the use of the chi-squared distribution to test whether heterogeneity is greater than zero (i.e., a two-sided test is not applicable). No adjustments for multiple testing were made.

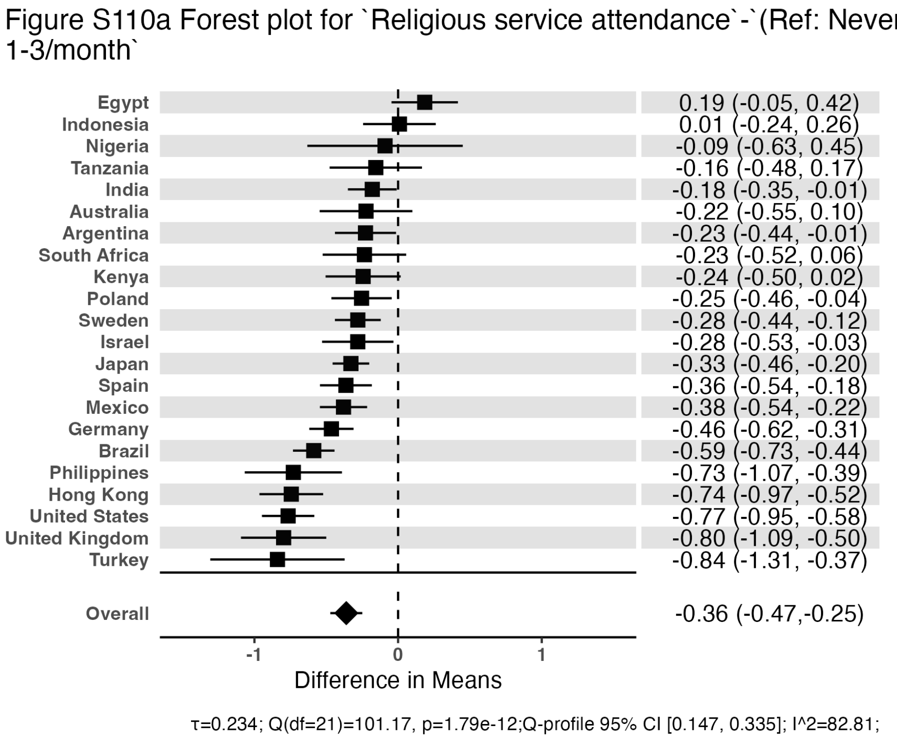

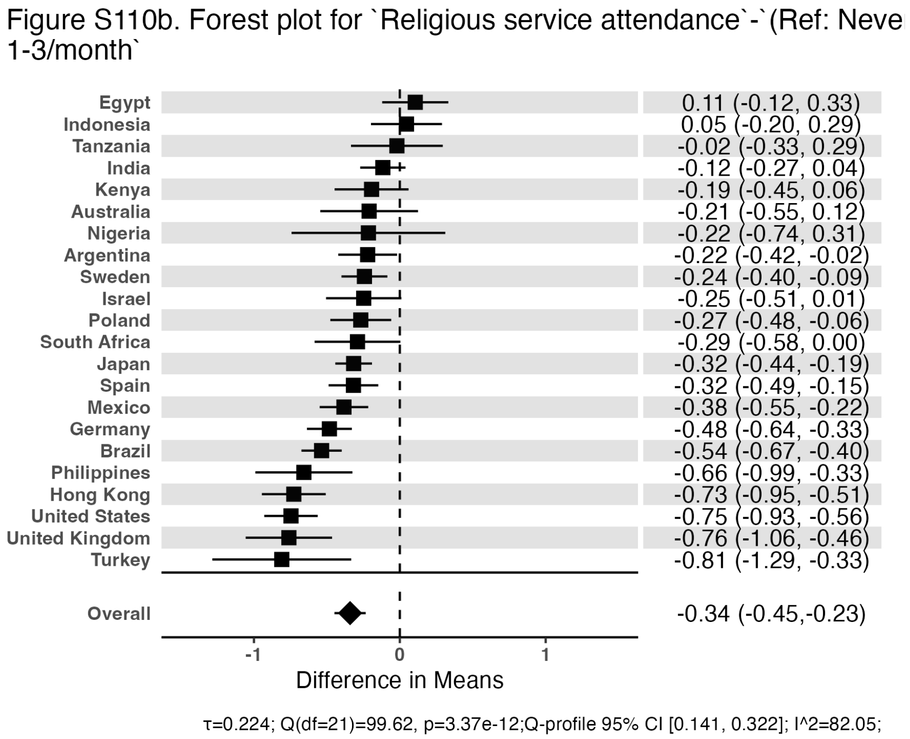

Figure S111. Heterogeneity in pairwise compairisons acrossx countries Religious service attendance-(Ref: 1/week) 1-3/month. (a) Flourishing with financial indicators (12 items) [left panel]; (b) Flourishing without financial indicators (10 items) [right panel]. N=202,898, subgroup means and standard errors are computed accounting for the complex sampling design using all data simultaneously. Analyses conducted: Random-effects meta-analysis of country-specific means. Squares represent the the point estimate (mean) for each country. The lines represented the +/- 1.96*SE, standard error, around the mean; the overall pooled mean is represented by the diamond. The reported p-value for Q-statistics is necessarily 1-sided because of the use of the chi-squared distribution to test whether heterogeneity is greater than zero (i.e., a two-sided test is not applicable). No adjustments for multiple testing were made.

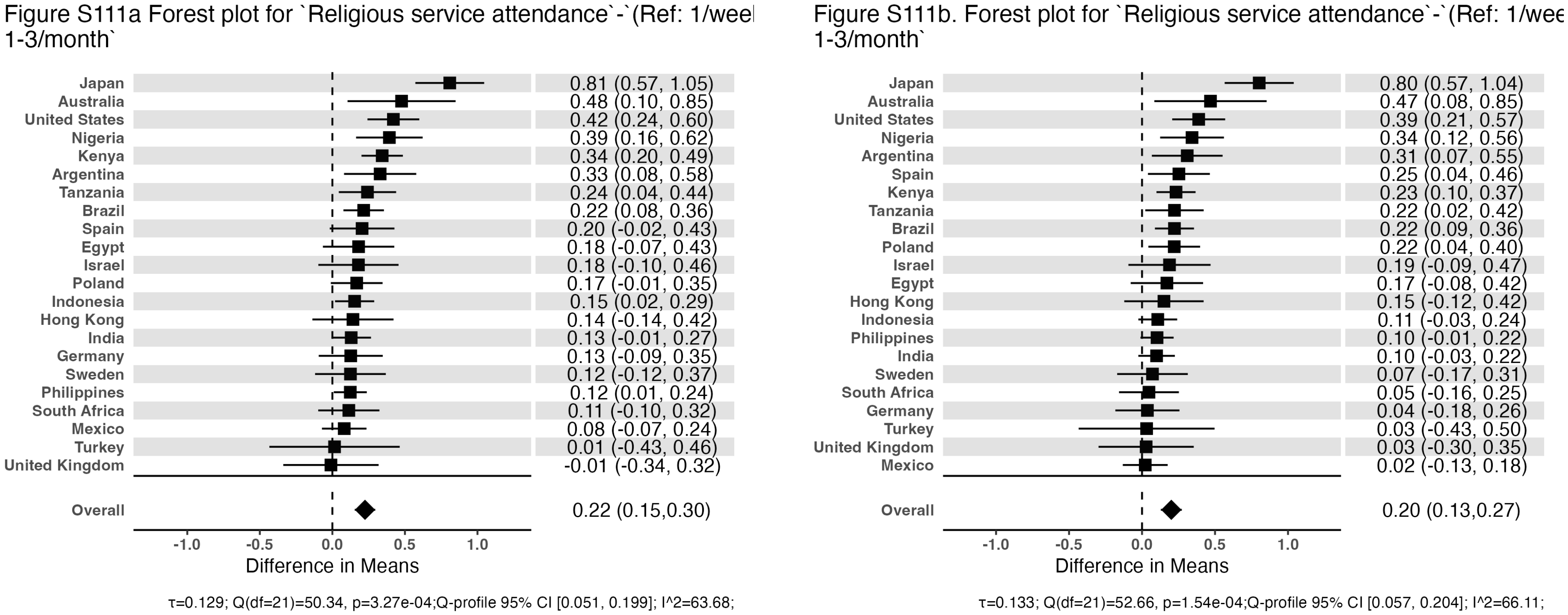

Figure S112. Heterogeneity in pairwise compairisons acrossx countries Education-(Ref: Up to 8 years) 16+ years. (a) Flourishing with financial indicators (12 items) [left panel]; (b) Flourishing without financial indicators (10 items) [right panel]. N=202,898, subgroup means and standard errors are computed accounting for the complex sampling design using all data simultaneously. Analyses conducted: Random-effects meta-analysis of country-specific means. Squares represent the the point estimate (mean) for each country. The lines represented the +/-1.96*SE, standard error, around the mean; the overall pooled mean is represented by the diamond. The reported p-value for Q-statistics is necessarily 1-sided because of the use of the chi-squared distribution to test whether heterogeneity is greater than zero (i.e., a two-sided test is not applicable). No adjustments for multiple testing were made.

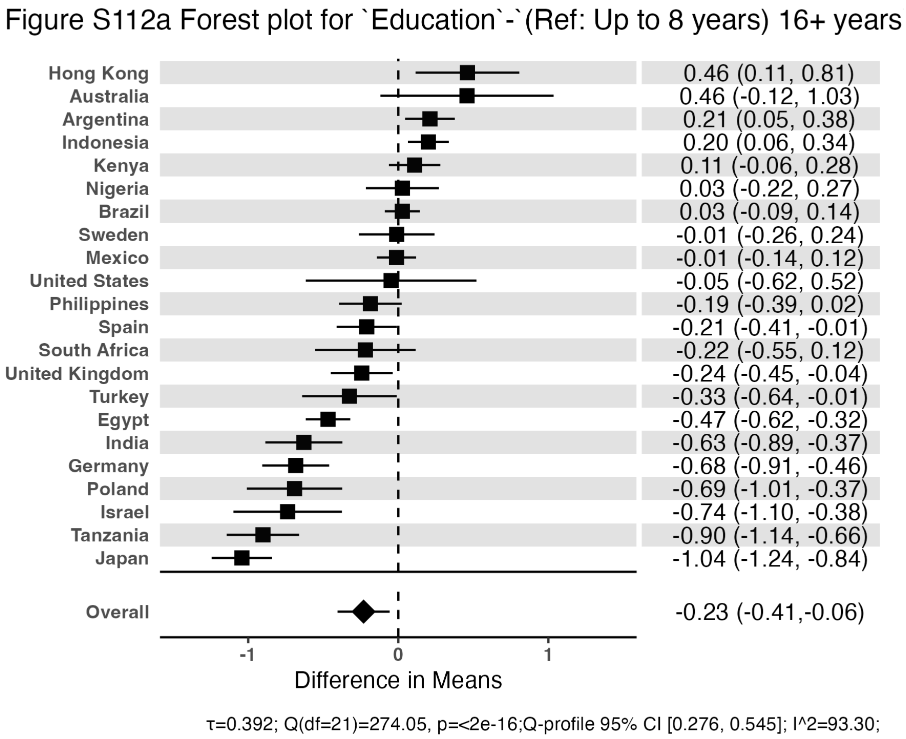

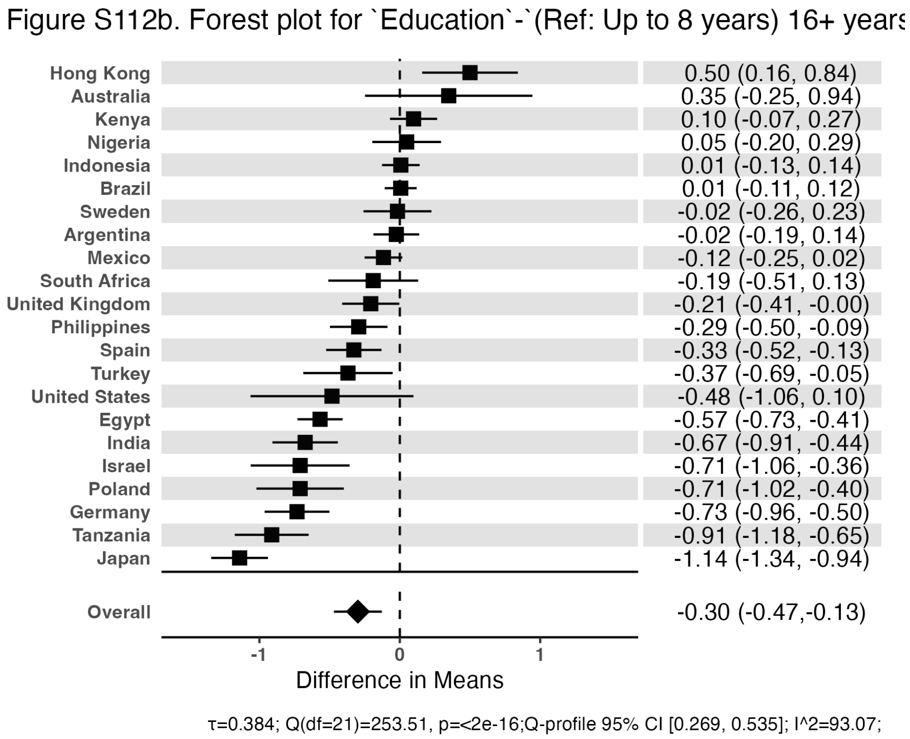

Figure S113. Heterogeneity in pairwise compairisons acrossx countries Education-(Ref: Up to 8 years) 9-15 years. (a) Flourishing with financial indicators (12 items) [left panel]; (b) Flourishing without financial indicators (10 items) [right panel]. N=202,898, subgroup means and standard errors are computed accounting for the complex sampling design using all data simultaneously. Analyses conducted: Random-effects meta-analysis of country-specific means. Squares represent the the point estimate (mean) for each country. The lines represented the +/-1.96*SE, standard error, around the mean; the overall pooled mean is represented by the diamond. The reported p-value for Q-statistics is necessarily 1-sided because of the use of the chi-squared distribution to test whether heterogeneity is greater than zero (i.e., a two-sided test is not applicable). No adjustments for multiple testing were made.

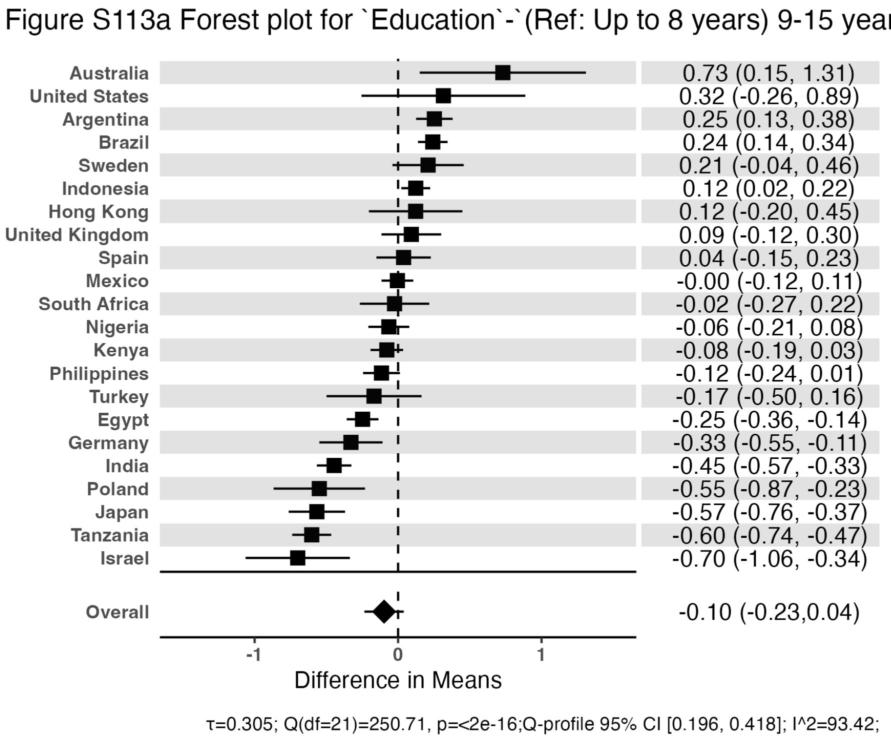

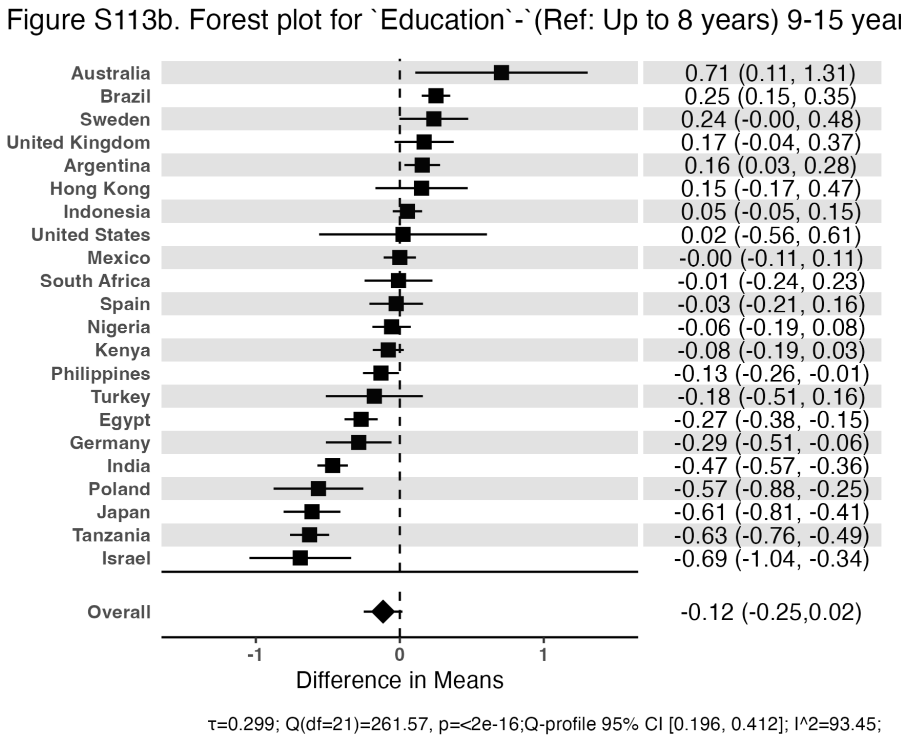

Figure S114. Heterogeneity in pairwise compairisons acrossx countries Education-(Ref: 16+ years) 9-15 years. (a) Flourishing with financial indicators (12 items) [left panel]; (b) Flourishing without financial indicators (10 items) [right panel]. N=202,898, subgroup means and standard errors are computed accounting for the complex sampling design using all data simultaneously. Analyses conducted: Random-effects meta-analysis of country-specific means. Squares represent the the point estimate (mean) for each country. The lines represented the +/-1.96*SE, standard error, around the mean; the overall pooled mean is represented by the diamond. The reported p-value for Q-statistics is necessarily 1-sided because of the use of the chi-squared distribution to test whether heterogeneity is greater than zero (i.e., a two-sided test is not applicable). No adjustments for multiple testing were made.

Figure S114a Forest plot for `Education`-`(Ref: 16+ years) 9-15 years`

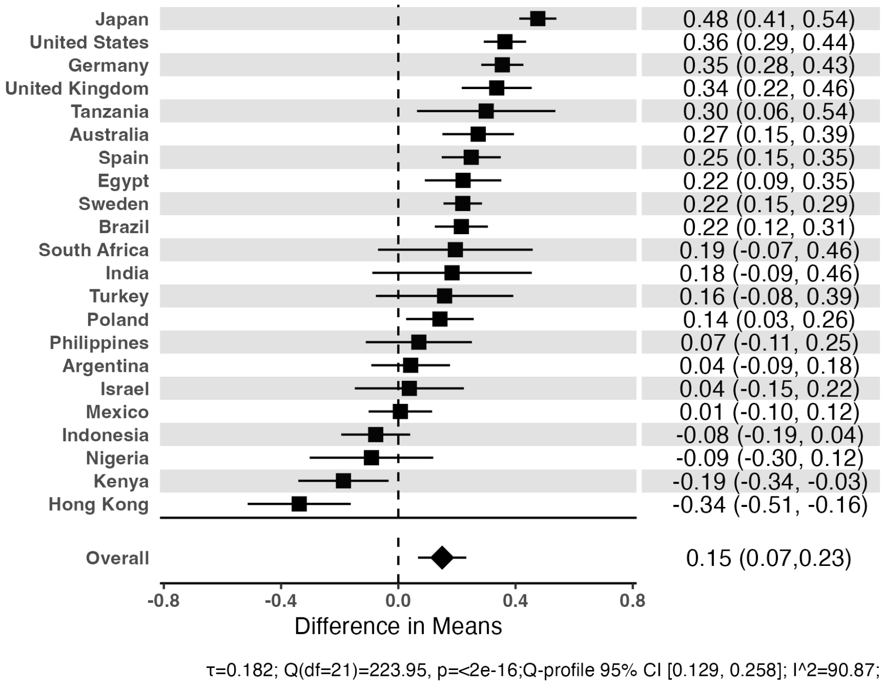

Figure S114b. Forest plot for `Education`-`(Ref: 16+ years) 9-15 years`

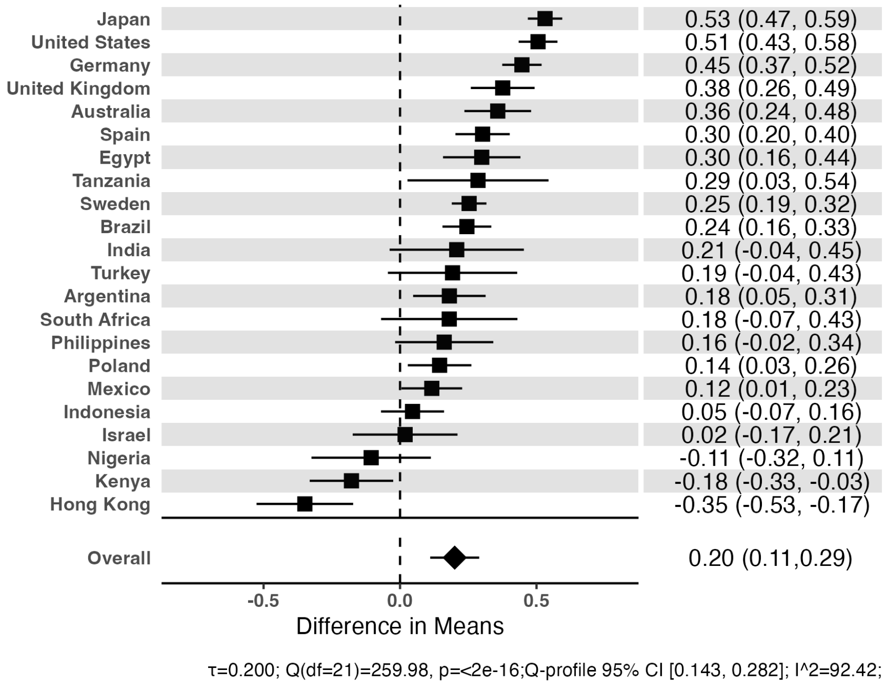

Figure S115. Heterogeneity in pairwise compairisons acrossx countries Immigration status-(Ref: Born in another country) Born in this country. (a) Flourishing with financial indicators (12 items) [left panel]; (b) Flourishing without financial indicators (10 items) [right panel]. N=202,898, subgroup means and standard errors are computed accounting for the complex sampling design using all data simultaneously. Analyses conducted: Random-effects meta-analysis of country-specific means. Squares represent the the point estimate (mean) for each country. The lines represented the +/-1.96*SE, standard error, around the mean; the overall pooled mean is represented by the diamond. The reported p-value for Q-statistics is necessarily 1-sided because of the use of the chi-squared distribution to test whether heterogeneity is greater than zero (i.e., a two-sided test is not applicable). No adjustments for multiple testing were made.

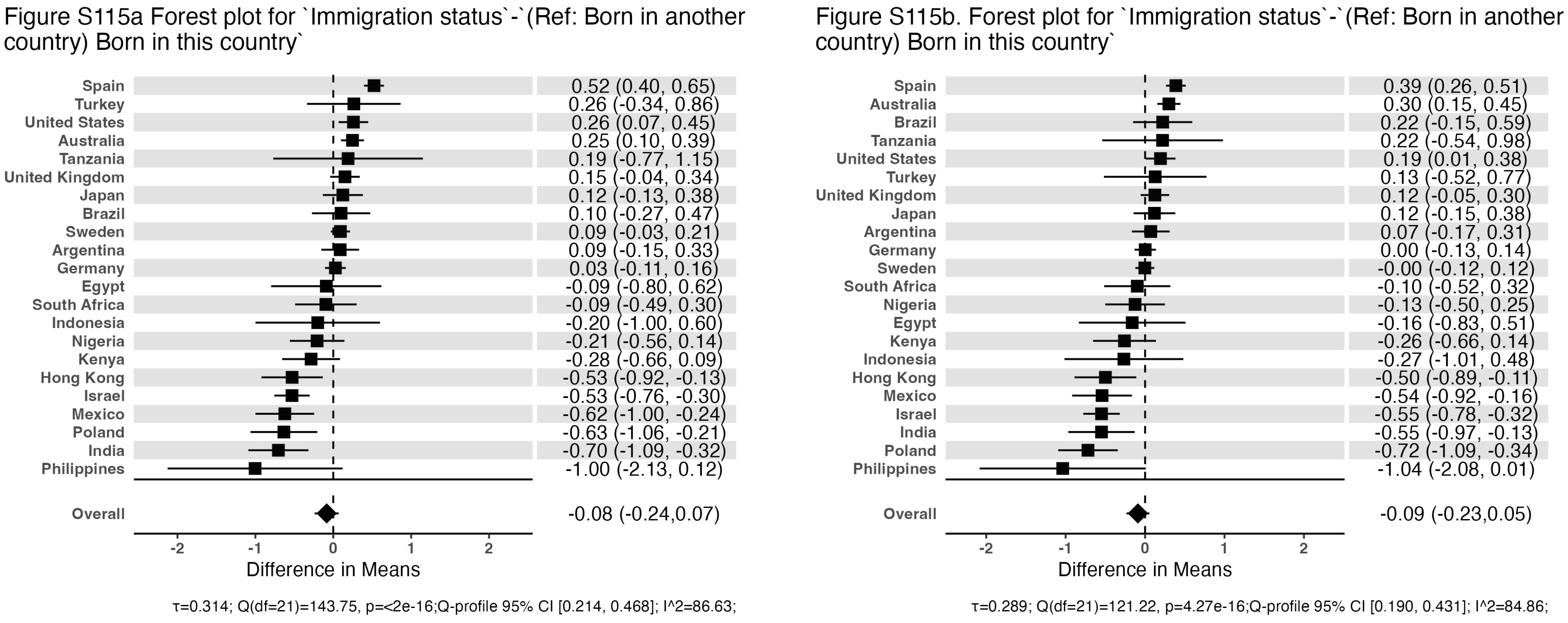

Part 4. Forest plots of childhood predictor effects.

Figure S116. Heterogeneity in pairwise comparisons across countries Relationship with mother (Ref: Very bad/somewhat bad) - Very good/somewhat good effect. (a) Flourishing with financial indicators (12 items) [left panel]; (b) Flourishing without financial indicators (10 items) [right panel]. N=202,898, subgroup means and standard errors are computed accounting for the complex sampling design using all data simultaneously. Analyses conducted: Random-effects meta-analysis of country-specific means. Squares represent the point estimate (mean) for each country. The lines represented the +/-1.96*SE, standard error, around the mean; the overall pooled mean is represented by the diamond. The reported p-value for Q-statistics is necessarily 1-sided because of the use of the chi-squared distribution to test whether heterogeneity is greater than zero (i.e., a two-sided test is not applicable). No adjustments were made for multiple testing.

Figure S116a Forest plot for `Relationship with mother`-`Very good/some
good` effect

Relationship with mother (Ref: Very bad/somewhat bad)

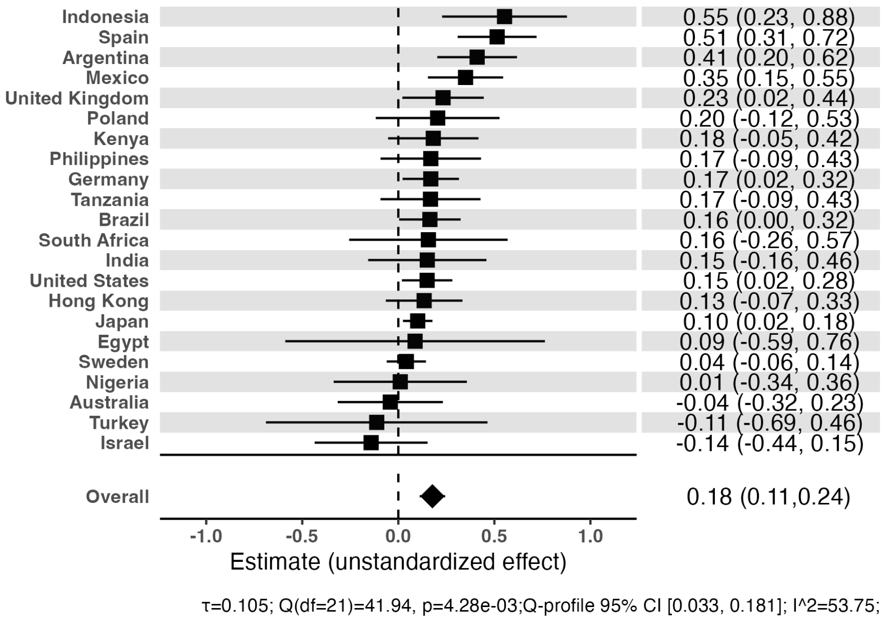

Figure S116b Forest plot for `Relationship with mother`-`Very good/some
good` effect

Relationship with mother (Ref: Very bad/somewhat bad)

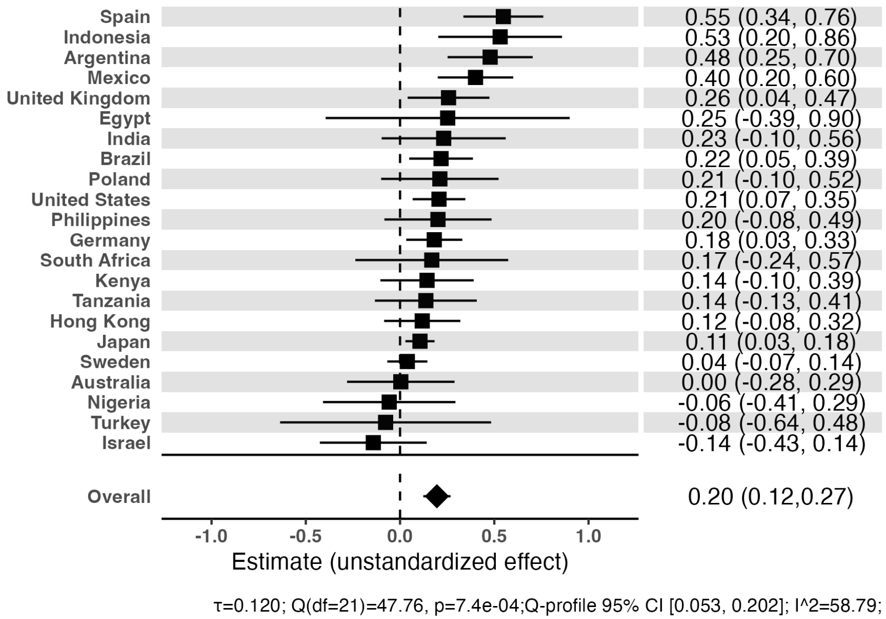

Figure S117. Heterogeneity in pairwise comparisons across countries Relationship with father (Ref: Very bad/somewhat bad) - Very good/somewhat good effect. (a) Flourishing with financial indicators (12 items) [left panel]; (b) Flourishing without financial indicators (10 items) [right panel]. N=202,898, subgroup means and standard errors are computed accounting for the complex sampling design using all data simultaneously. Analyses conducted: Random-effects meta-analysis of country-specific means. Squares represent the point estimate (mean) for each country. The lines represented the +/-1.96*SE, standard error, around the mean; the overall pooled mean is represented by the diamond. The reported p-value for Q-statistics is necessarily 1-sided because of the use of the chi-squared distribution to test whether heterogeneity is greater than zero (i.e., a two-sided test is not applicable). No adjustments were made for multiple testing.

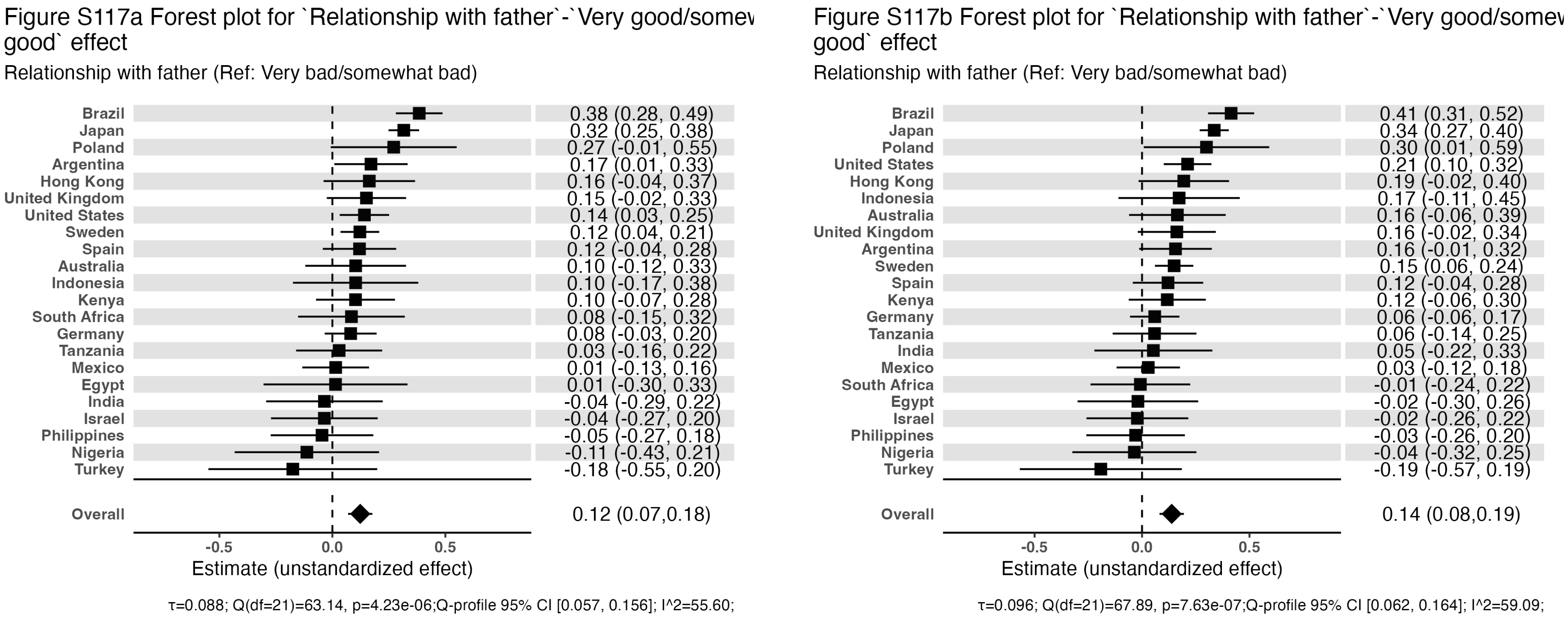

Figure S118. Heterogeneity in pairwise comparisons across countries Parent marital status (Ref: Parents married) - Divorced effect. (a) Flourishing with financial indicators (12 items) [left panel]; (b) Flourishing without financial indicators (10 items) [right panel]. N=202,898, subgroup means and standard errors are computed accounting for the complex sampling design using all data simultaneously. Analyses conducted: Random-effects meta-analysis of country-specific means. Squares represent the point estimate (mean) for each country. The lines represented the +/-1.96*SE, standard error, around the mean; the overall pooled mean is represented by the diamond. The reported p-value for Q-statistics is necessarily 1-sided because of the use of the chi-squared distribution to test whether heterogeneity is greater than zero (i.e., a two-sided test is not applicable). No adjustments were made for multiple testing.

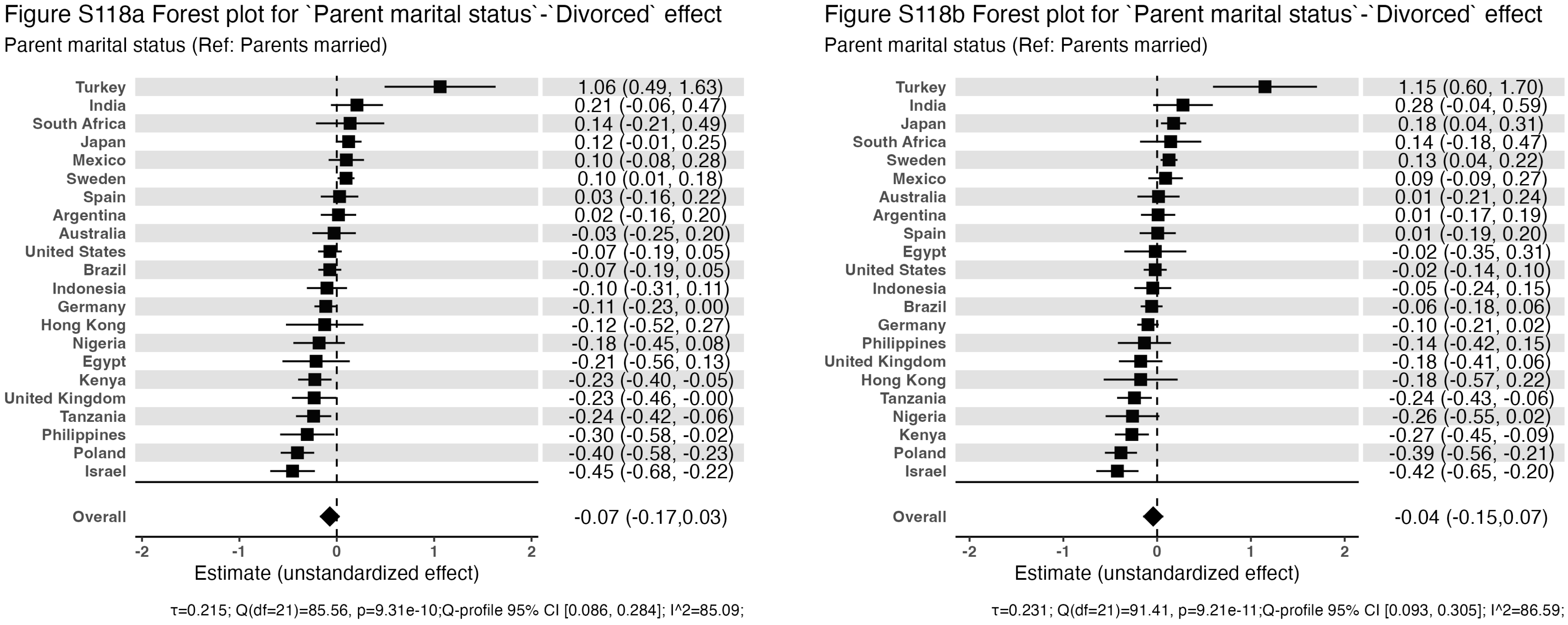

Figure S119. Heterogeneity in pairwise comparisons across countries Parent marital status (Ref: Parents married) - Single, never married effect. (a) Flourishing with financial indicators (12 items) [left panel]; (b) Flourishing without financial indicators (10 items) [right panel]. N=202,898, subgroup means and standard errors are computed accounting for the complex sampling design using all data simultaneously. Analyses conducted: Random-effects meta-analysis of country-specific means. Squares represent the point estimate (mean) for each country. The lines represented the +/-1.96*SE, standard error, around the mean; the overall pooled mean is represented by the diamond. The reported p-value for Q-statistics is necessarily 1-sided because of the use of the chi-squared distribution to test whether heterogeneity is greater than zero (i.e., a two-sided test is not applicable). No adjustments were made for multiple testing.

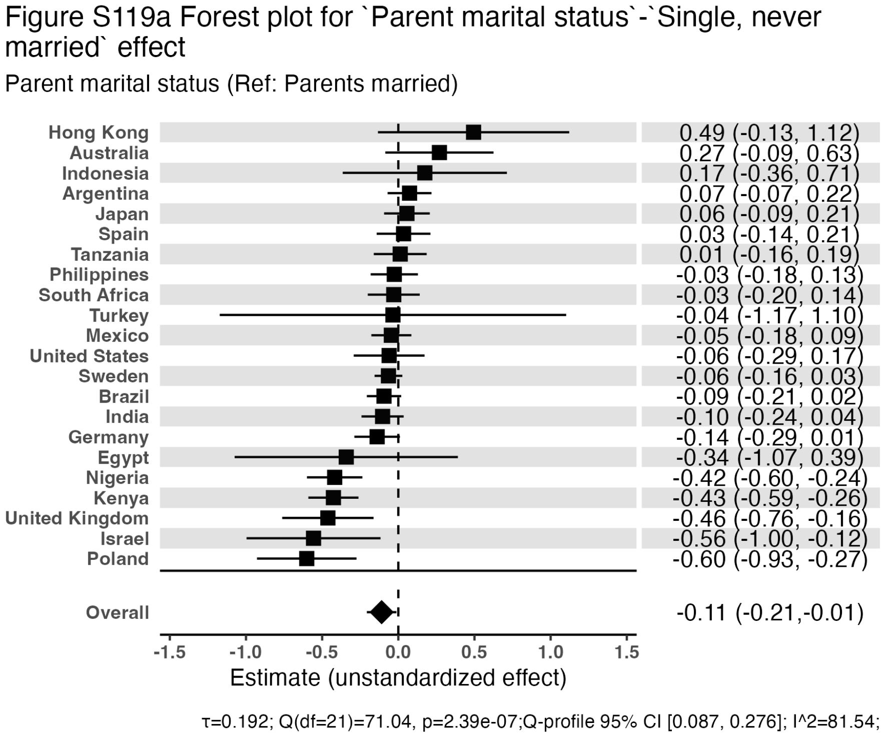

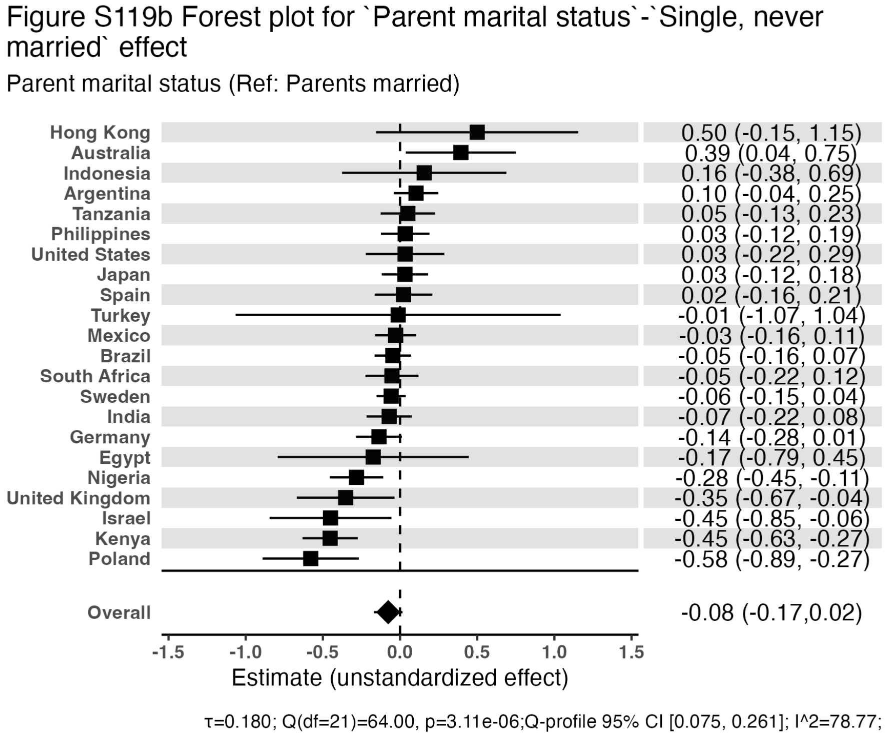

Figure S120. Heterogeneity in pairwise comparisons across countries Parent marital status (Ref: Parents married) - One or both parents had died effect. (a) Flourishing with financial indicators (12 items) [left panel]; (b) Flourishing without financial indicators (10 items) [right panel]. N=202,898, subgroup means and standard errors are computed accounting for the complex sampling design using all data simultaneously. Analyses conducted: Random-effects meta-analysis of country-specific means. Squares represent the point estimate (mean) for each country. The lines represented the +/-1.96*SE, standard error, around the mean; the overall pooled mean is represented by the diamond. The reported p-value for Q-statistics is necessarily 1-sided because of the use of the chi-squared distribution to test whether heterogeneity is greater than zero (i.e., a two-sided test is not applicable). No adjustments were made for multiple testing.

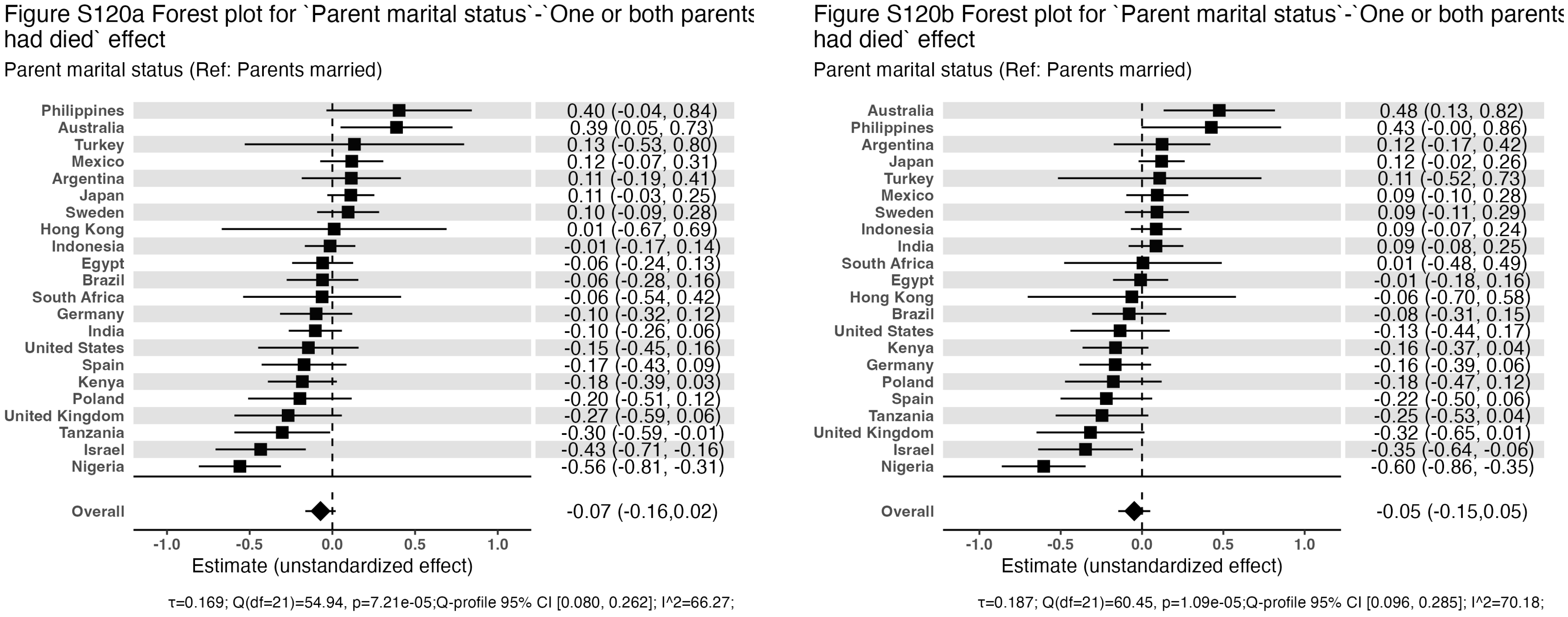

Figure S121. Heterogeneity in pairwise comparisons across countries Subjective financial status of family growing up (Ref: Got by) - Lived comfortably effect. (a) Flourishing with financial indicators (12 items) [left panel]; (b) Flourishing without financial indicators (10 items) [right panel]. N=202,898, subgroup means and standard errors are computed accounting for the complex sampling design using all data simultaneously. Analyses conducted: Random-effects meta-analysis of country-specific means. Squares represent the point estimate (mean) for each country. The lines represented the +/-1.96*SE, standard error, around the mean; the overall pooled mean is represented by the diamond. The reported p-value for Q-statistics is necessarily 1-sided because of the use of the chi-squared distribution to test whether heterogeneity is greater than zero (i.e., a two-sided test is not applicable). No adjustments were made for multiple testing.

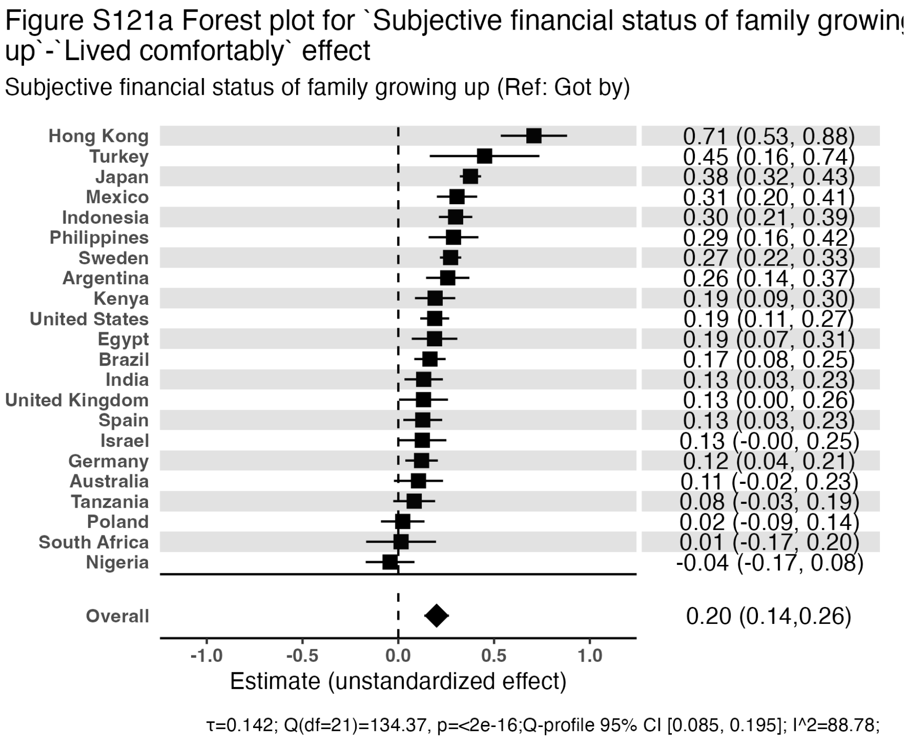

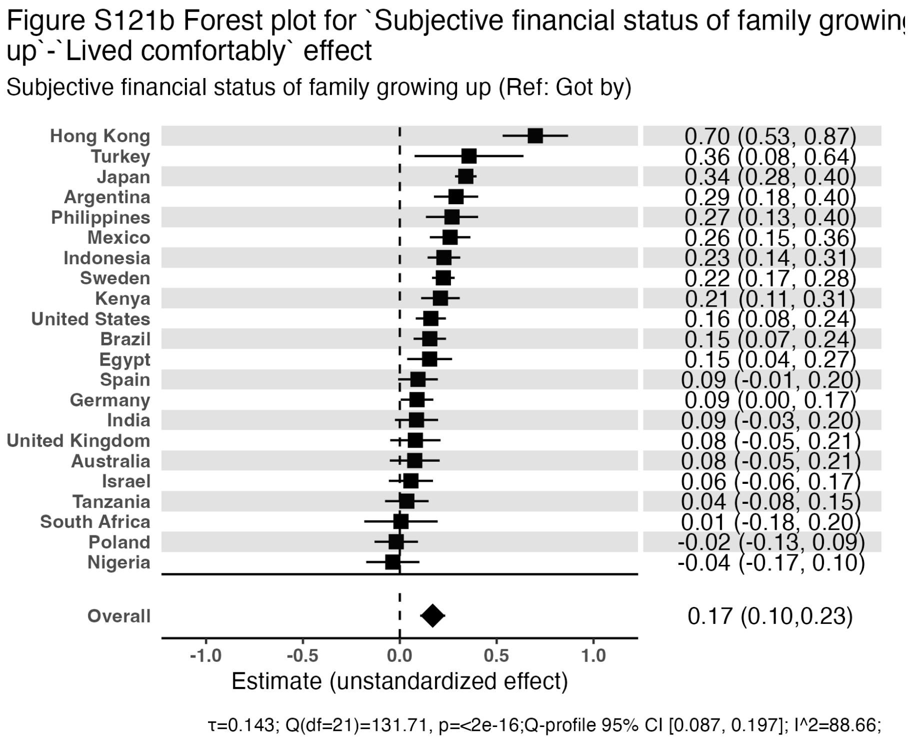

Figure S122. Heterogeneity in pairwise comparisons across countries Subjective financial status of family growing up (Ref: Got by) - Found it difficult effect. (a) Flourishing with financial indicators (12 items) [left panel]; (b) Flourishing without financial indicators (10 items) [right panel]. N=202,898, subgroup means and standard errors are computed accounting for the complex sampling design using all data simultaneously. Analyses conducted: Random-effects meta-analysis of country-specific means. Squares represent the point estimate (mean) for each country. The lines represented the +/-1.96*SE, standard error, around the mean; the overall pooled mean is represented by the diamond. The reported p-value for Q-statistics is necessarily 1-sided because of the use of the chi-squared distribution to test whether heterogeneity is greater than zero (i.e., a two-sided test is not applicable). No adjustments were made for multiple testing.

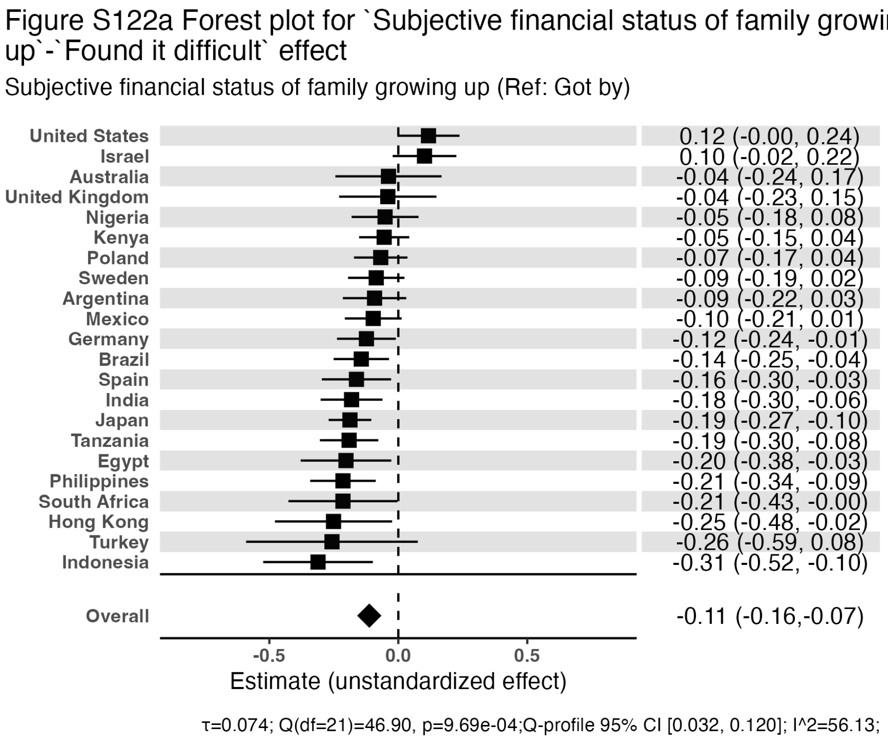

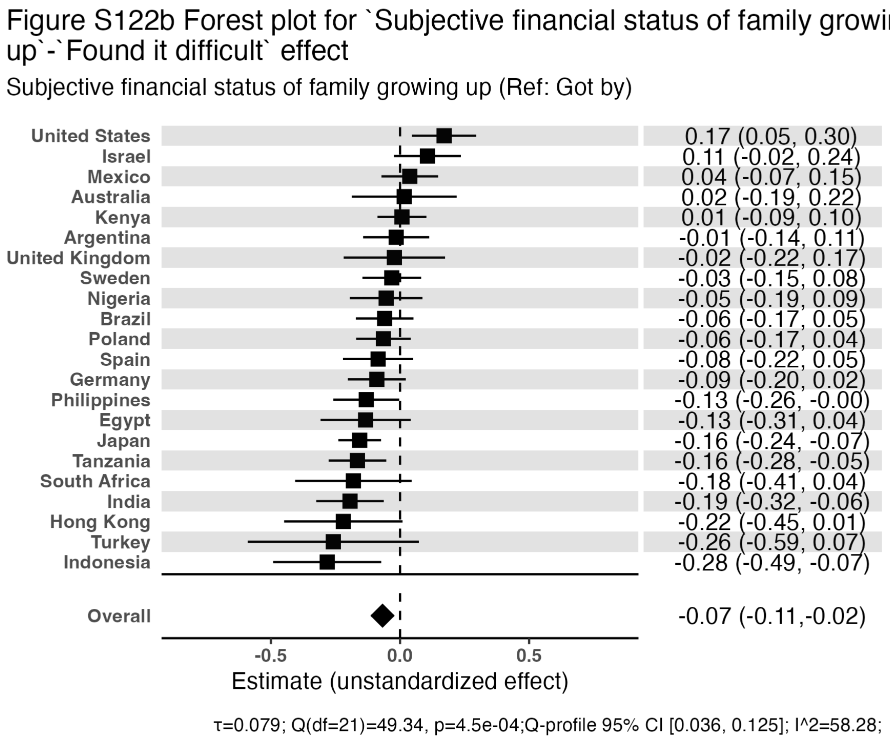

Figure S123. Heterogeneity in pairwise comparisons across countries Subjective financial status of family growing up (Ref: Got by) - Found it very difficult effect. (a) Flourishing with financial indicators (12 items) [left panel]; (b) Flourishing without financial indicators (10 items) [right panel]. N=202,898, subgroup means and standard errors are computed accounting for the complex sampling design using all data simultaneously. Analyses conducted: Random-effects meta-analysis of country-specific means. Squares represent the point estimate (mean) for each country. The lines represented the +/-1.96*SE, standard error, around the mean; the overall pooled mean is represented by the diamond. The reported p-value for Q-statistics is necessarily 1-sided because of the use of the chi-squared distribution to test whether heterogeneity is greater than zero (i.e., a two-sided test is not applicable). No adjustments were made for multiple testing.

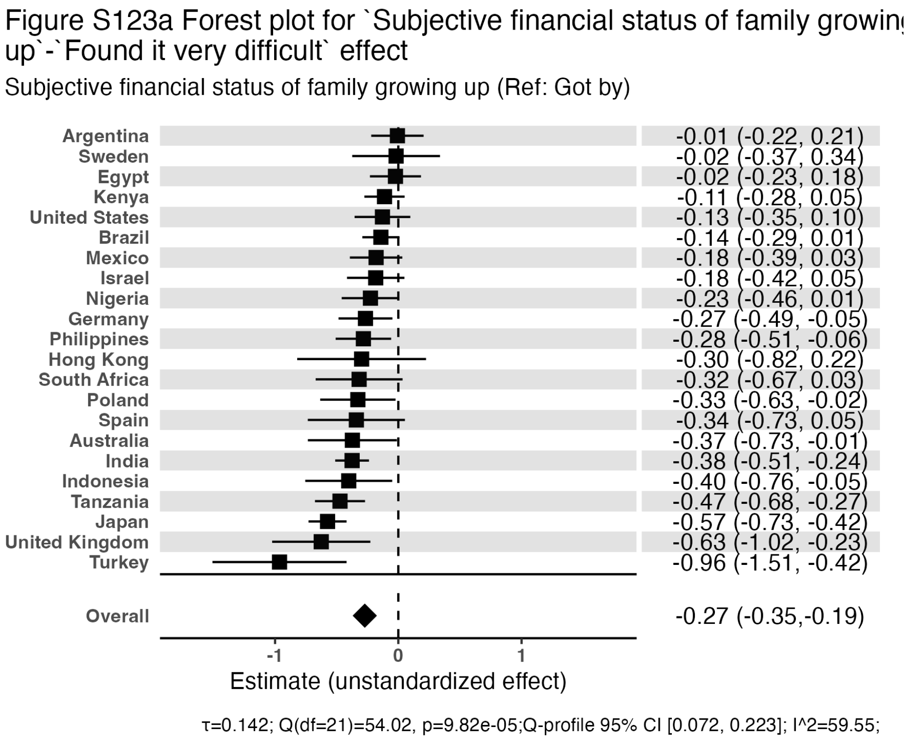

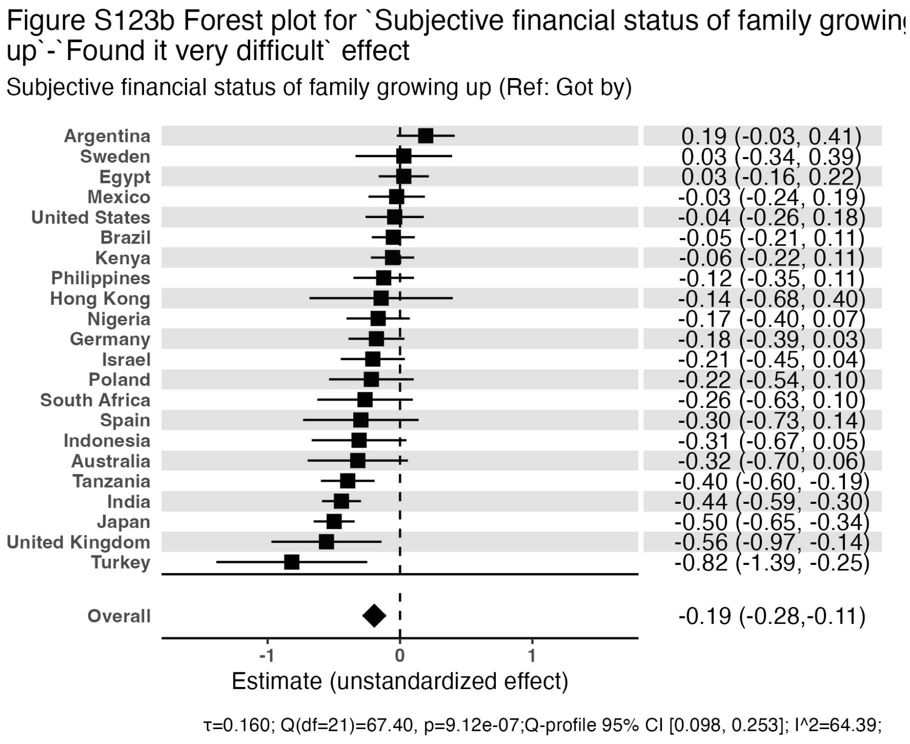

Figure S124. Heterogeneity in pairwise comparisons across countries Abuse (Ref: No) - Yes effect. (a) Flourishing with financial indicators (12 items) [left panel]; (b) Flourishing without financial indicators (10 items) [right panel]. N=202,898, subgroup means and standard errors are computed accounting for the complex sampling design using all data simultaneously. Analyses conducted: Random-effects meta-analysis of country-specific means. Squares represent the point estimate (mean) for each country. The lines represented the +/-1.96*SE, standard error, around the mean; the overall pooled mean is represented by the diamond. The reported p-value for Q-statistics is necessarily 1-sided because of the use of the chi-squared distribution to test whether heterogeneity is greater than zero (i.e., a two-sided test is not applicable). No adjustments were made for multiple testing.

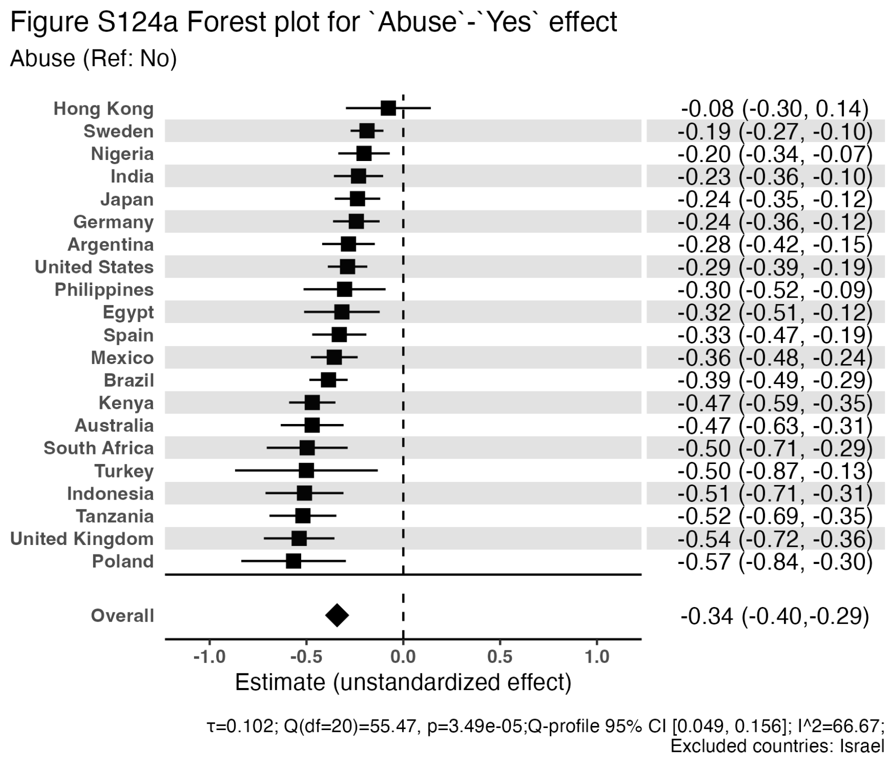

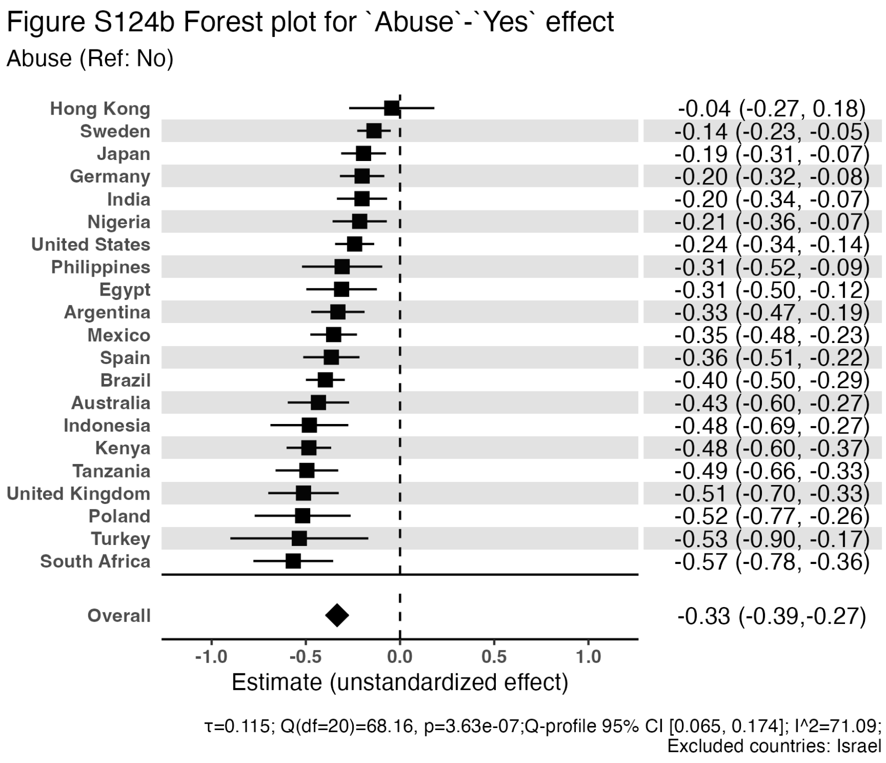

Figure S125. Heterogeneity in pairwise comparisons across countries Outsider growing up (Ref: No) - Yes effect. (a) Flourishing with financial indicators (12 items) [left panel]; (b) Flourishing without financial indicators (10 items) [right panel]. N=202,898, subgroup means and standard errors are computed accounting for the complex sampling design using all data simultaneously. Analyses conducted: Random-effects meta-analysis of country-specific means. Squares represent the point estimate (mean) for each country. The lines represented the +/-1.96*SE, standard error, around the mean; the overall pooled mean is represented by the diamond. The reported p-value for Q-statistics is necessarily 1-sided because of the use of the chi-squared distribution to test whether heterogeneity is greater than zero (i.e., a two-sided test is not applicable). No adjustments were made for multiple testing.

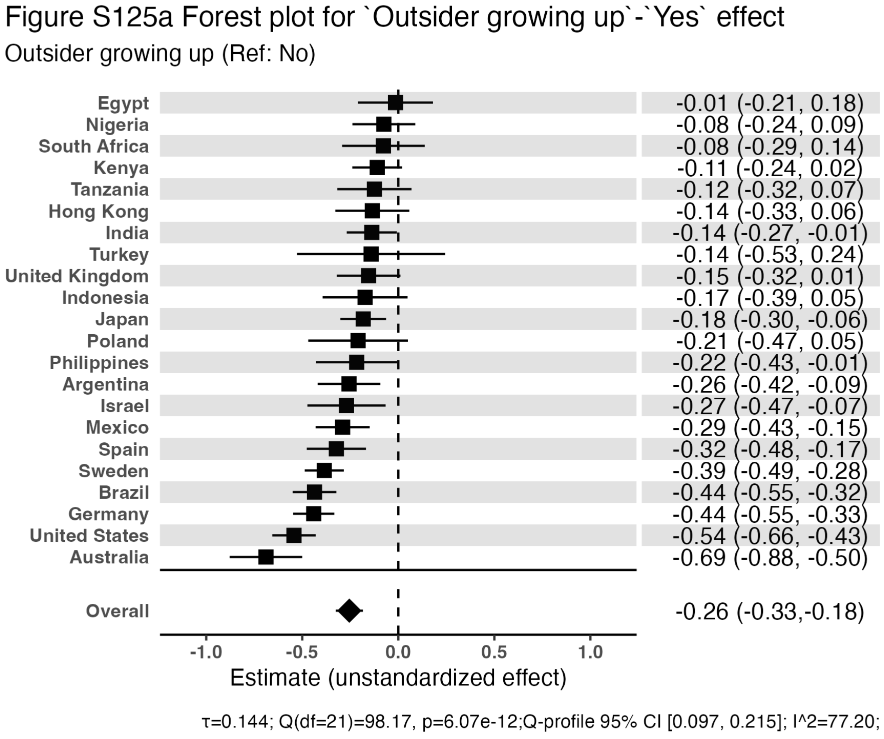

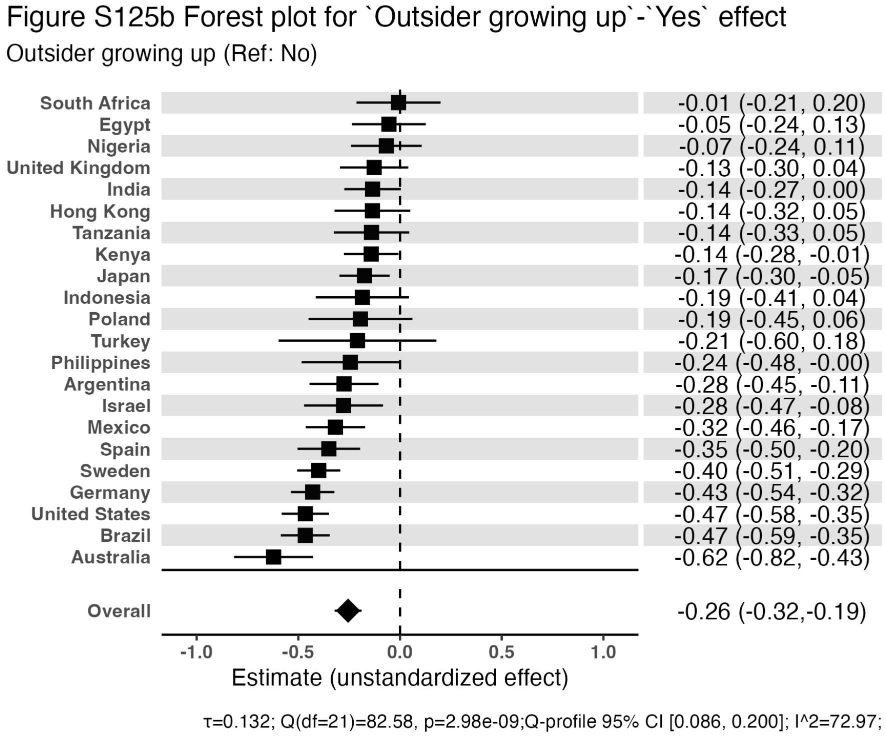

Figure S126. Heterogeneity in pairwise comparisons across countries Self-rated health growing up (Ref: Good) - Excellent effect. (a) Flourishing with financial indicators (12 items) [left panel]; (b) Flourishing without financial indicators (10 items) [right panel]. N=202,898, subgroup means and standard errors are computed accounting for the complex sampling design using all data simultaneously. Analyses conducted: Random-effects meta-analysis of country-specific means. Squares represent the point estimate (mean) for each country. The lines represented the +/-1.96*SE, standard error, around the mean; the overall pooled mean is represented by the diamond. The reported p-value for Q-statistics is necessarily 1-sided because of the use of the chi-squared distribution to test whether heterogeneity is greater than zero (i.e., a two-sided test is not applicable). No adjustments were made for multiple testing.

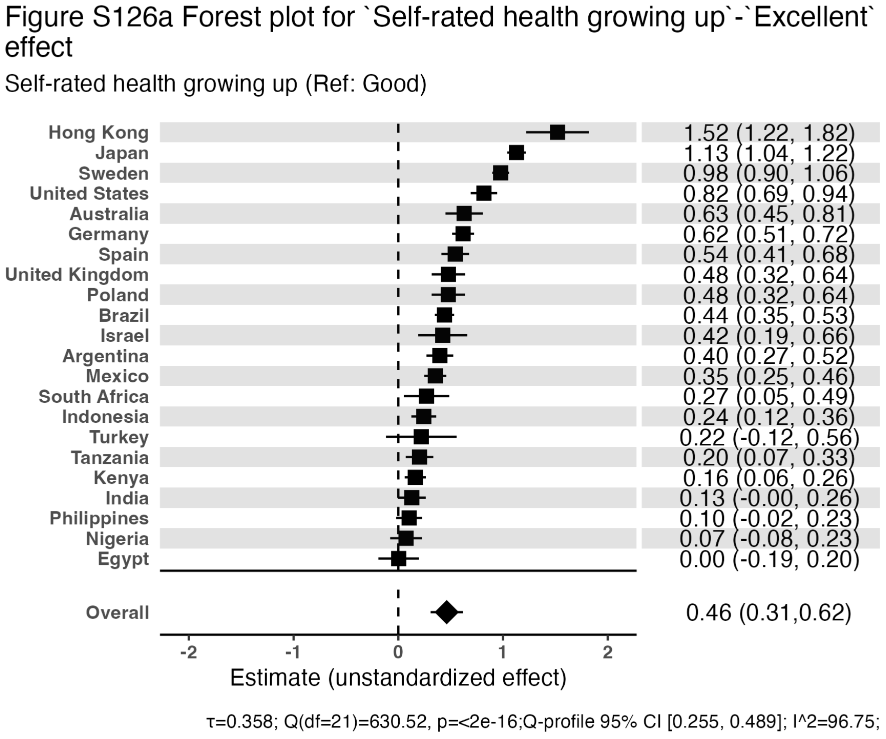

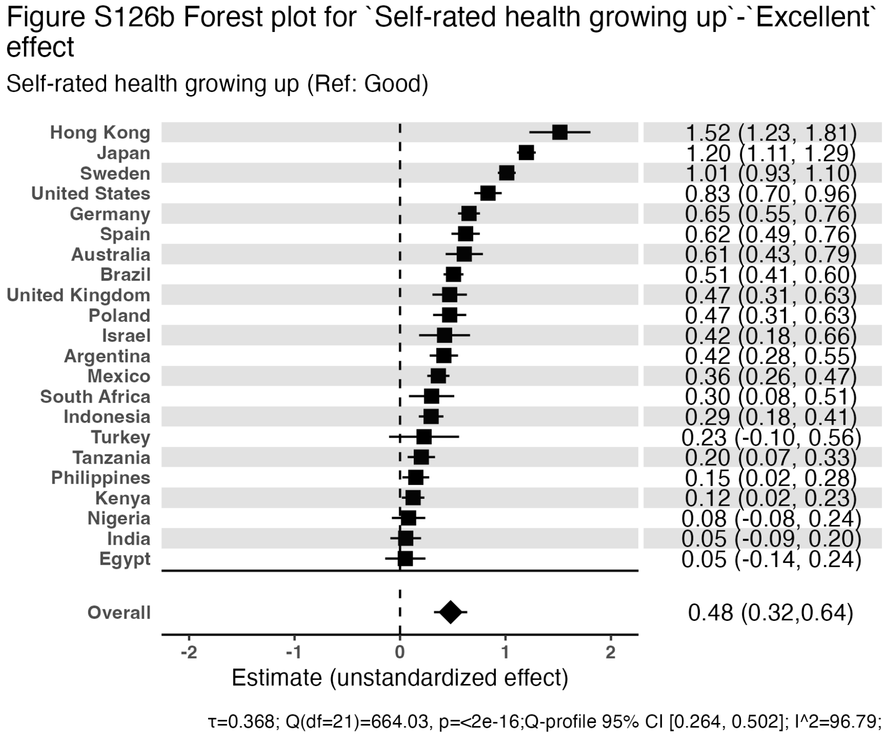

Figure S127. Heterogeneity in pairwise comparisons across countries Self-rated health growing up (Ref: Good) - Very good effect. (a) Flourishing with financial indicators (12 items) [left panel]; (b) Flourishing without financial indicators (10 items) [right panel]. N=202,898, subgroup means and standard errors are computed accounting for the complex sampling design using all data simultaneously. Analyses conducted: Random-effects meta-analysis of country-specific means. Squares represent the point estimate (mean) for each country. The lines represented the +/-1.96*SE, standard error, around the mean; the overall pooled mean is represented by the diamond. The reported p-value for Q-statistics is necessarily 1-sided because of the use of the chi-squared distribution to test whether heterogeneity is greater than zero (i.e., a two-sided test is not applicable). No adjustments were made for multiple testing.

Figure S127a Forest plot for `Self-rated health growing up`-`Very good` effect

Self-rated health growing up (Ref: Good)

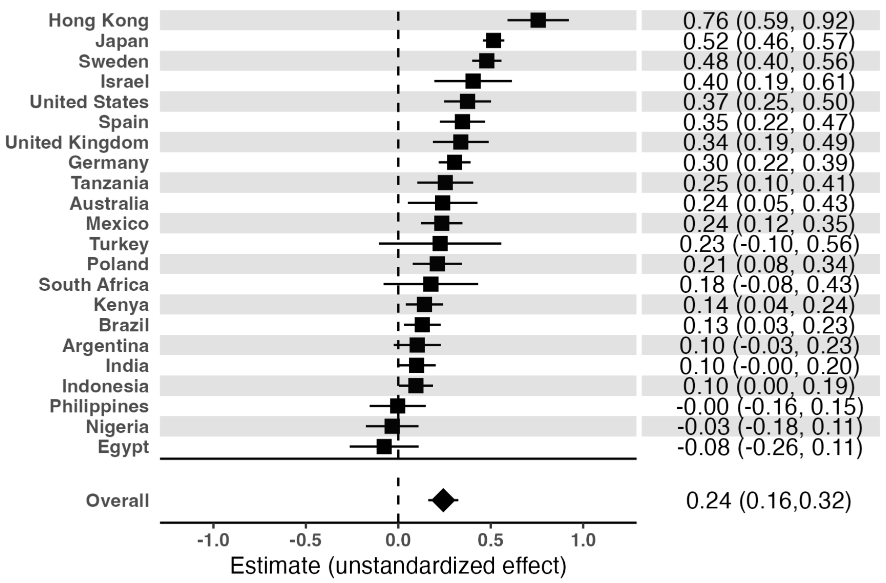

τ=0.179; Q(df=21)=223.07, p=<2e-16;Q-profile 95% CI [0.126, 0.254]; I^2=89.94;

Figure S127b Forest plot for `Self-rated health growing up`-`Very good` effect

Self-rated health growing up (Ref: Good)

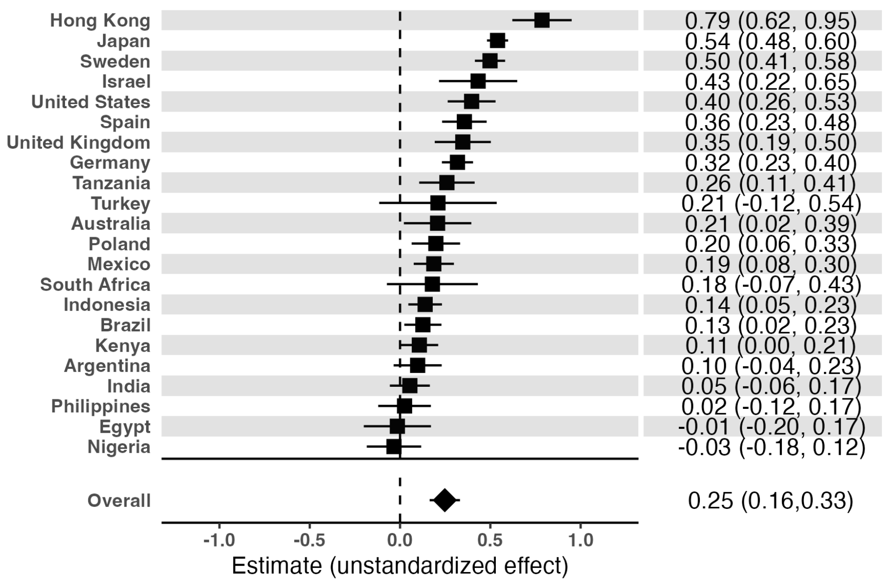

τ=0.186; Q(df=21)=239.67, p=<2e-16;Q-profile 95% CI [0.132, 0.263]; I^2=90.23;

Figure S128. Heterogeneity in pairwise comparisons across countries Self-rated health growing up (Ref: Good) - Fair effect. (a) Flourishing with financial indicators (12 items) [left panel]; (b) Flourishing without financial indicators (10 items) [right panel]. N=202,898, subgroup means and standard errors are computed accounting for the complex sampling design using all data simultaneously. Analyses conducted: Random-effects meta-analysis of country-specific means. Squares represent the point estimate (mean) for each country. The lines represented the +/-1.96*SE, standard error, around the mean; the overall pooled mean is represented by the diamond. The reported p-value for Q-statistics is necessarily 1-sided because of the use of the chi-squared distribution to test whether heterogeneity is greater than zero (i.e., a two-sided test is not applicable). No adjustments were made for multiple testing.

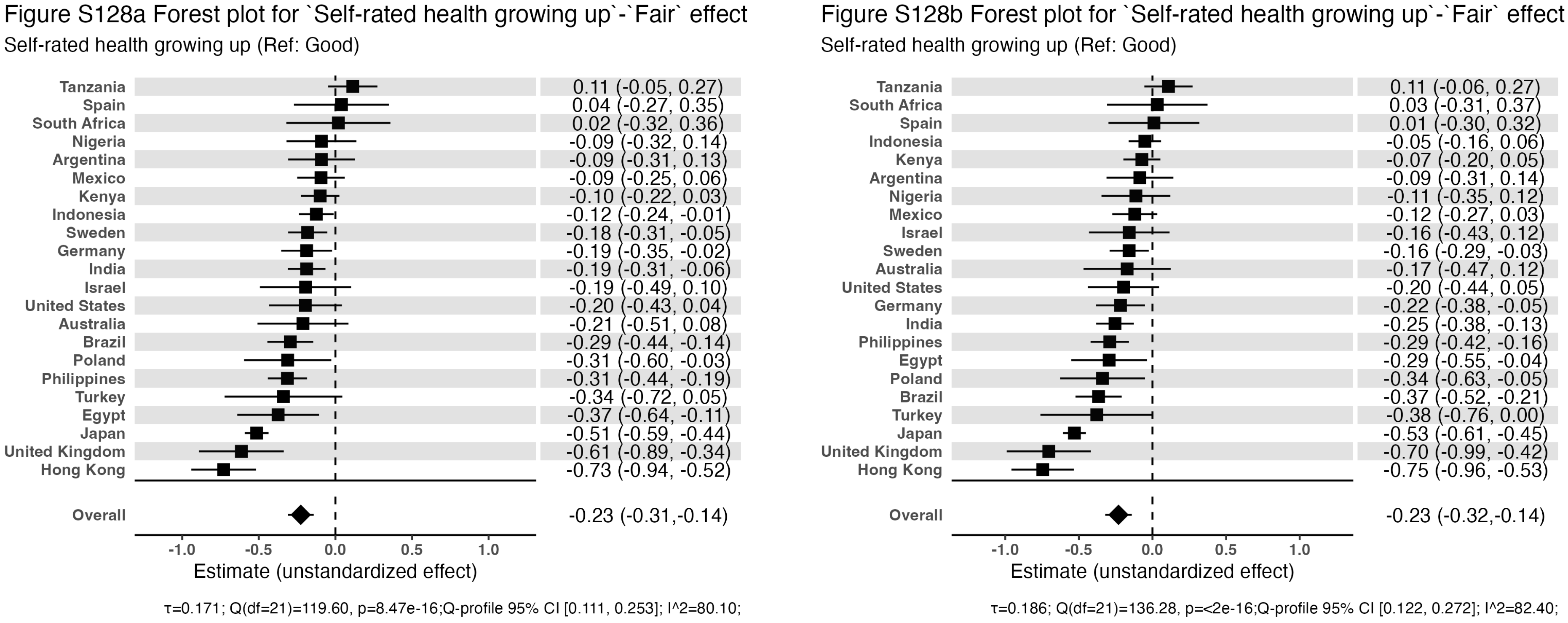

Figure S129. Heterogeneity in pairwise comparisons across countries Self-rated health growing up (Ref: Good) - Poor effect. (a) Flourishing with financial indicators (12 items) [left panel]; (b) Flourishing without financial indicators (10 items) [right panel]. N=202,898, subgroup means and standard errors are computed accounting for the complex sampling design using all data simultaneously. Analyses conducted: Random-effects meta-analysis of country-specific means. Squares represent the point estimate (mean) for each country. The lines represented the +/-1.96*SE, standard error, around the mean; the overall pooled mean is represented by the diamond. The reported p-value for Q-statistics is necessarily 1-sided because of the use of the chi-squared distribution to test whether heterogeneity is greater than zero (i.e., a two-sided test is not applicable). No adjustments were made for multiple testing.

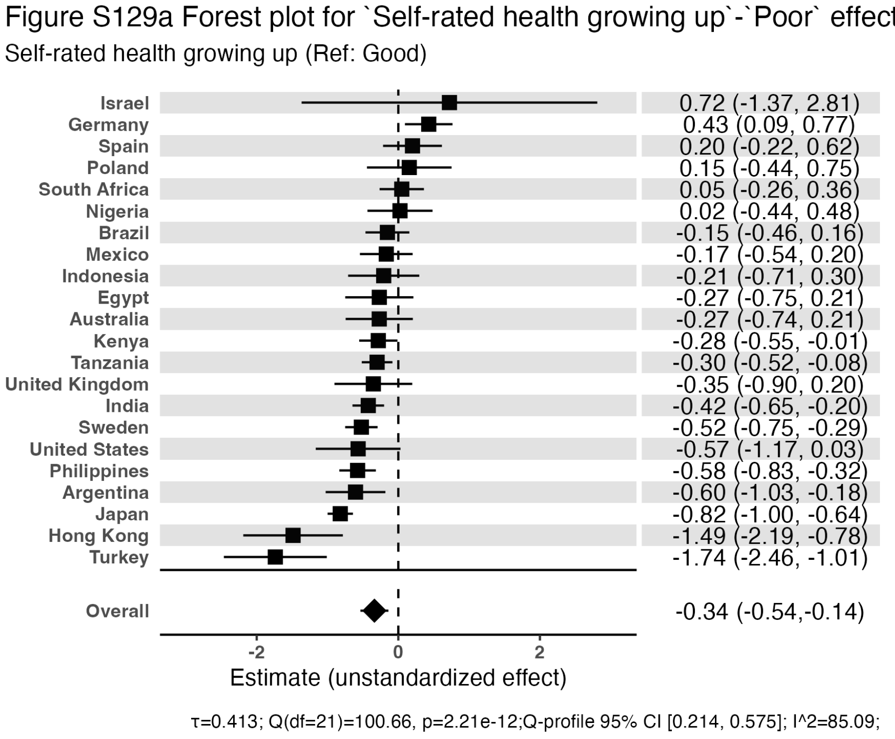

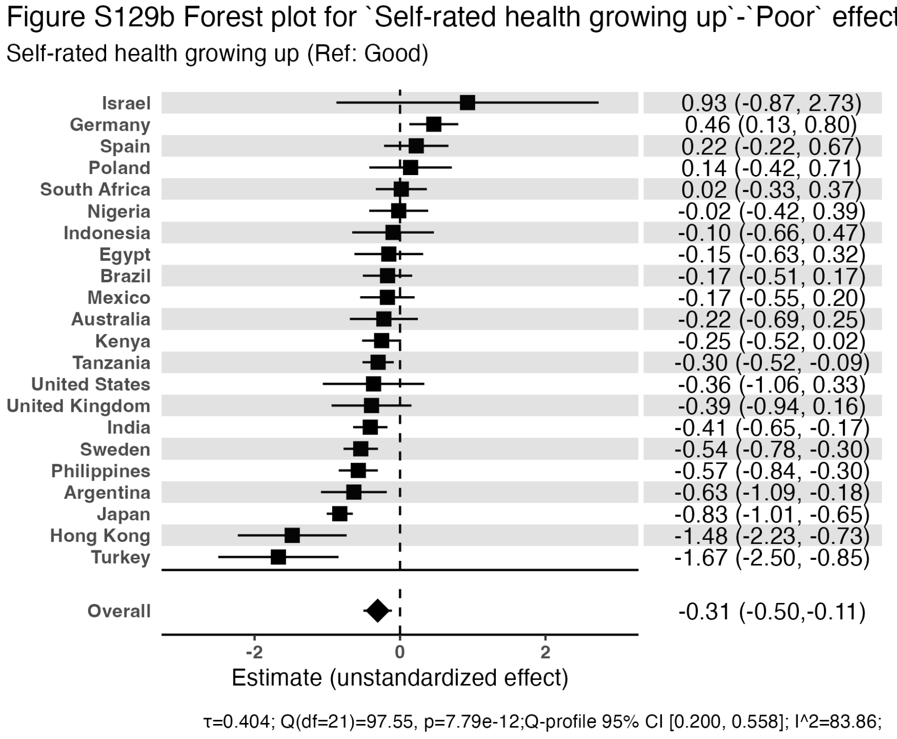

Figure S130. Heterogeneity in pairwise comparisons across countries Immigration status (Ref: Born in this country) - Born in another country effect. (a) Flourishing with financial indicators (12 items) [left panel]; (b) Flourishing without financial indicators (10 items) [right panel]. N=202,898, subgroup means and standard errors are computed accounting for the complex sampling design using all data simultaneously. Analyses conducted: Random-effects meta-analysis of country-specific means. Squares represent the point estimate (mean) for each country. The lines represented the +/-1.96*SE, standard error, around the mean; the overall pooled mean is represented by the diamond. The reported p-value for Q-statistics is necessarily 1-sided because of the use of the chi-squared distribution to test whether heterogeneity is greater than zero (i.e., a two-sided test is not applicable). No adjustments were made for multiple testing.

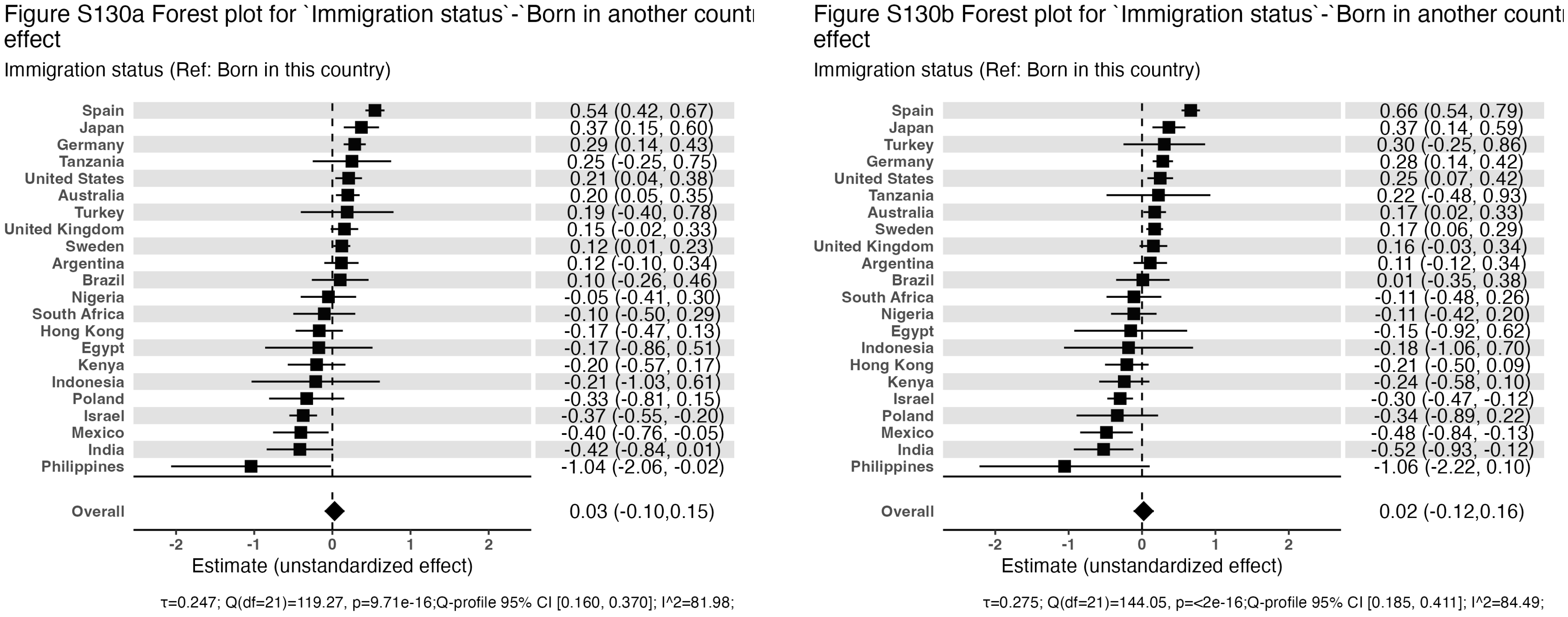

Figure S131. Heterogeneity in pairwise comparisons across countries Age 12 religious service attendance (Ref: Never) - At least 1/week effect. (a) Flourishing with financial indicators (12 items) [left panel]; (b) Flourishing without financial indicators (10 items) [right panel]. N=202,898, subgroup means and standard errors are computed accounting for the complex sampling design using all data simultaneously. Analyses conducted: Random-effects meta-analysis of country-specific means. Squares represent the point estimate (mean) for each country. The lines represented the +/-1.96*SE, standard error, around the mean; the overall pooled mean is represented by the diamond. The reported p-value for Q-statistics is necessarily 1-sided because of the use of the chi-squared distribution to test whether heterogeneity is greater than zero (i.e., a two-sided test is not applicable). No adjustments were made for multiple testing.

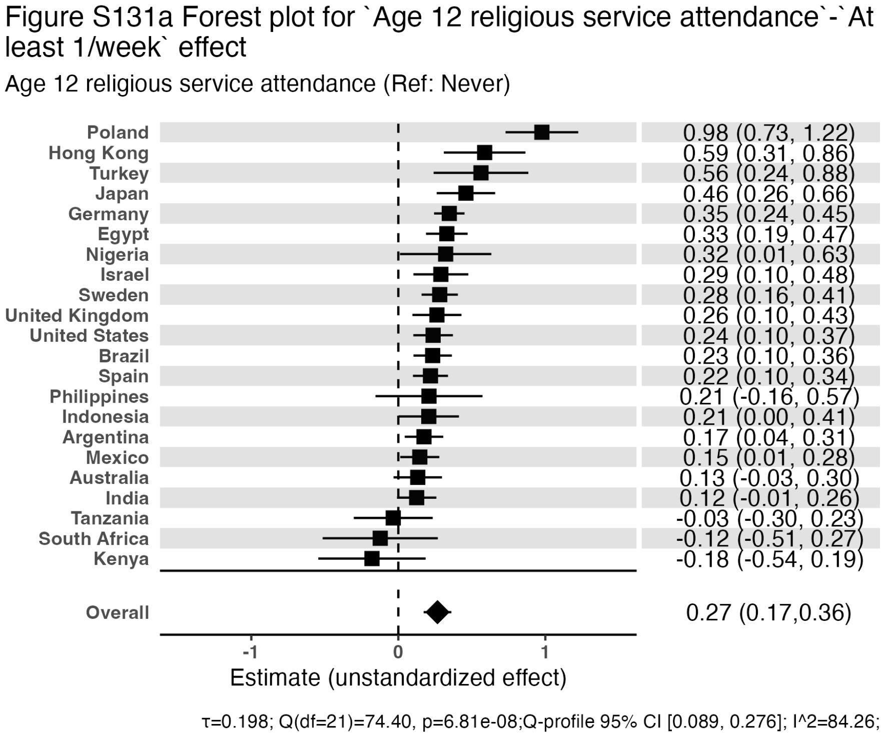

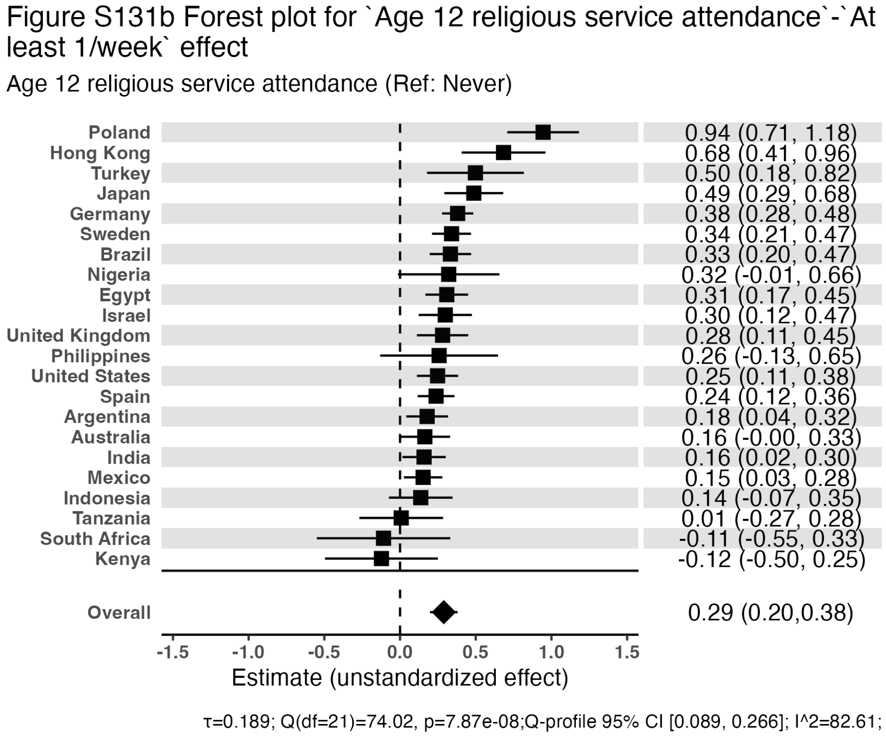

Figure S132. Heterogeneity in pairwise comparisons across countries Age 12 religious service attendance (Ref: Never) - 1-3/month effect. (a) Flourishing with financial indicators (12 items) [left panel]; (b) Flourishing without financial indicators (10 items) [right panel]. N=202,898, subgroup means and standard errors are computed accounting for the complex sampling design using all data simultaneously. Analyses conducted: Random-effects meta-analysis of country-specific means. Squares represent the point estimate (mean) for each country. The lines represented the +/-1.96*SE, standard error, around the mean; the overall pooled mean is represented by the diamond. The reported p-value for Q-statistics is necessarily 1-sided because of the use of the chi-squared distribution to test whether heterogeneity is greater than zero (i.e., a two-sided test is not applicable). No adjustments were made for multiple testing.

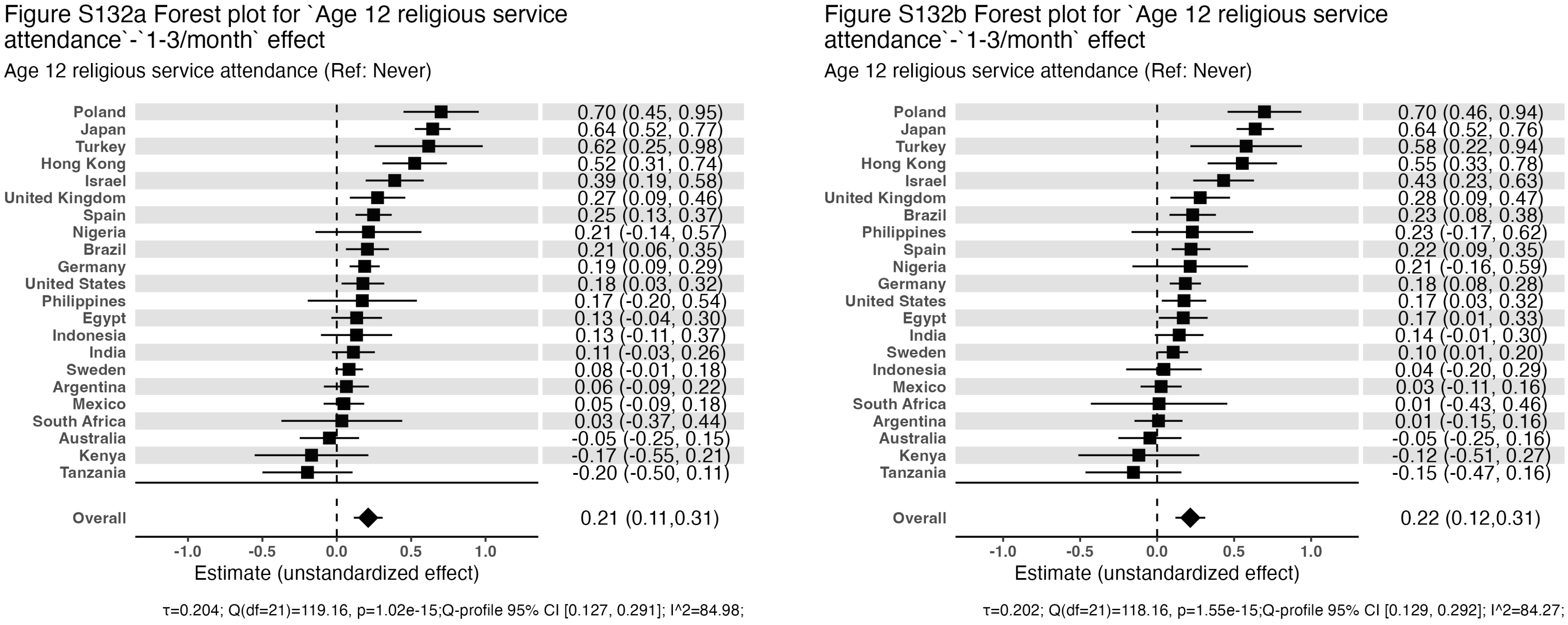

Figure S133. Heterogeneity in pairwise comparisons across countries Age 12 religious service attendance (Ref: Never) - < 1/month effect. (a) Flourishing with financial indicators (12 items) [left panel]; (b) Flourishing without financial indicators (10 items) [right panel]. N=202,898, subgroup means and standard errors are computed accounting for the complex sampling design using all data simultaneously. Analyses conducted: Random-effects meta-analysis of country-specific means. Squares represent the point estimate (mean) for each country. The lines represented the +/-1.96*SE, standard error, around the mean; the overall pooled mean is represented by the diamond. The reported p-value for Q-statistics is necessarily 1-sided because of the use of the chi-squared distribution to test whether heterogeneity is greater than zero (i.e., a two-sided test is not applicable). No adjustments were made for multiple testing.

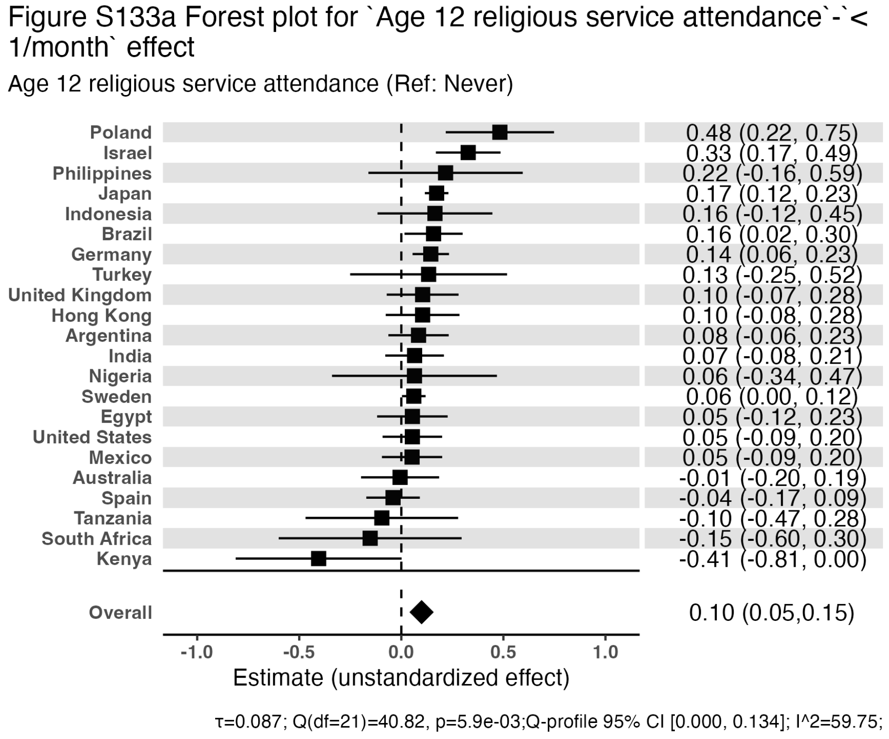

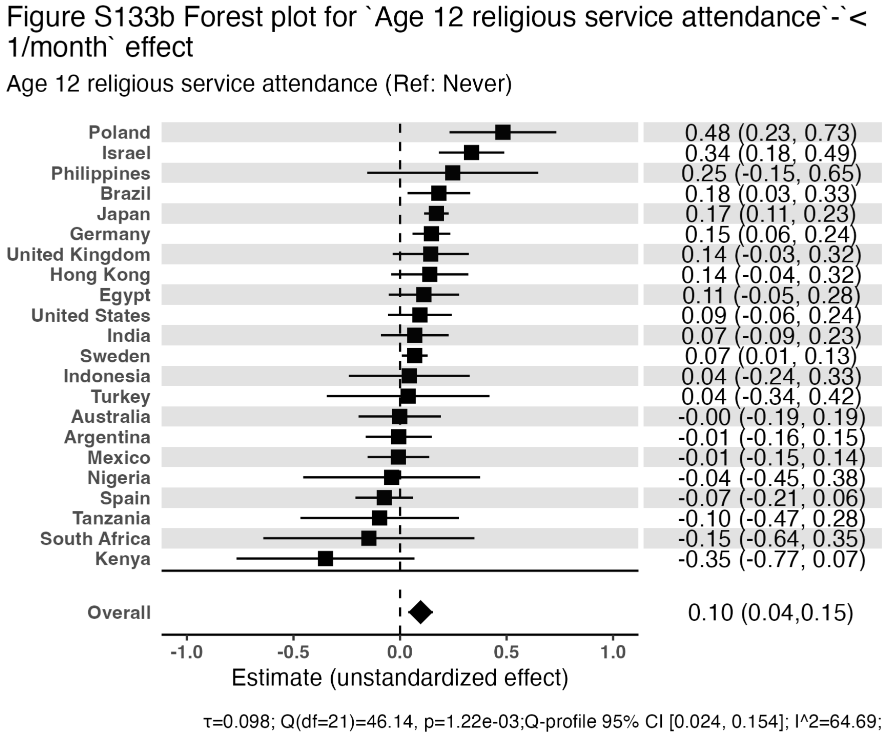

Figure S134. Heterogeneity in pairwise comparisons across countries Year of birth (Ref: 1998-2005; age 18-24) - 1993-1998; age 25-29 effect. (a) Flourishing with financial indicators (12 items) [left panel]; (b) Flourishing without financial indicators (10 items) [right panel]. N=202,898, subgroup means and standard errors are computed accounting for the complex sampling design using all data simultaneously. Analyses conducted: Random-effects meta-analysis of country-specific means. Squares represent the point estimate (mean) for each country. The lines represented the +/-1.96*SE, standard error, around the mean; the overall pooled mean is represented by the diamond. The reported p-value for Q-statistics is necessarily 1-sided because of the use of the chi-squared distribution to test whether heterogeneity is greater than zero (i.e., a two-sided test is not applicable). No adjustments were made for multiple testing.

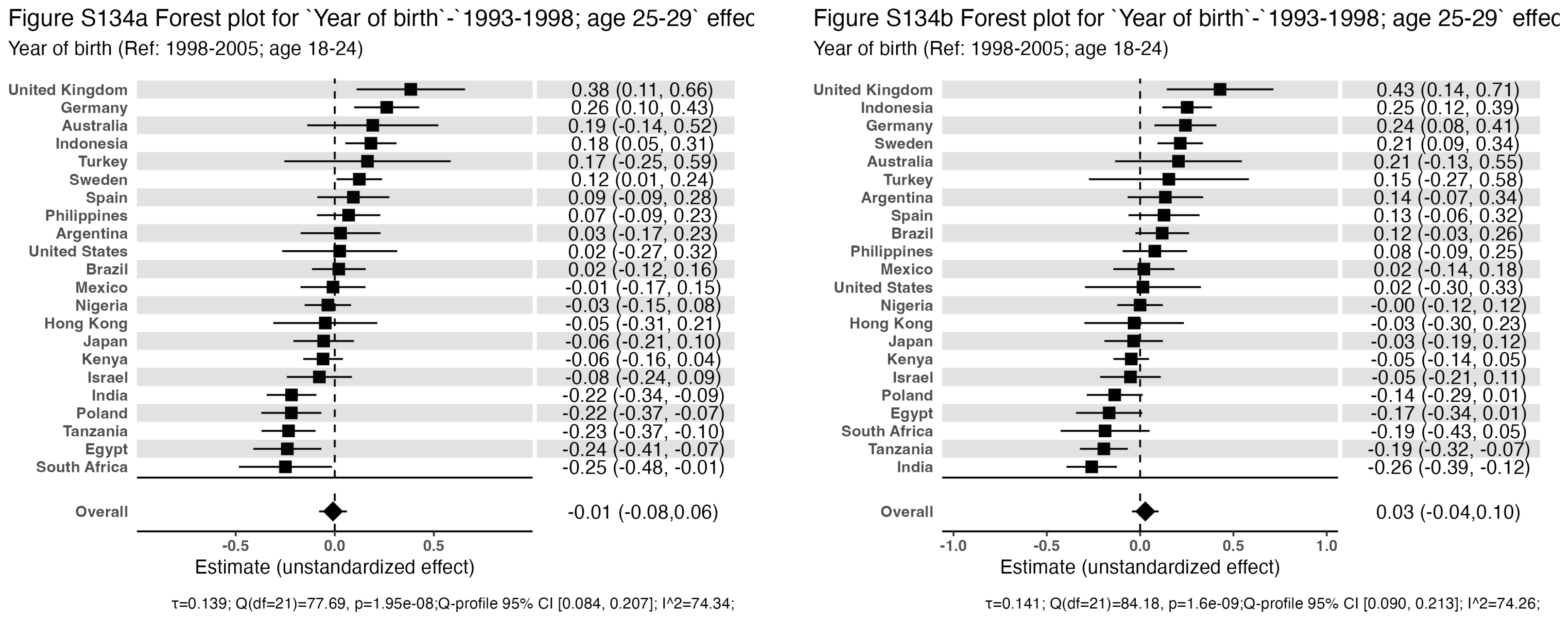

Figure S135. Heterogeneity in pairwise comparisons across countries Year of birth (Ref: 1998-2005; age 18-24) - 1983-1993; age 30-39 effect. (a) Flourishing with financial indicators (12 items) [left panel]; (b) Flourishing without financial indicators (10 items) [right panel]. N=202,898, subgroup means and standard errors are computed accounting for the complex sampling design using all data simultaneously. Analyses conducted: Random-effects meta-analysis of country-specific means. Squares represent the point estimate (mean) for each country. The lines represented the +/-1.96*SE, standard error, around the mean; the overall pooled mean is represented by the diamond. The reported p-value for Q-statistics is necessarily 1-sided because of the use of the chi-squared distribution to test whether heterogeneity is greater than zero (i.e., a two-sided test is not applicable). No adjustments were made for multiple testing.

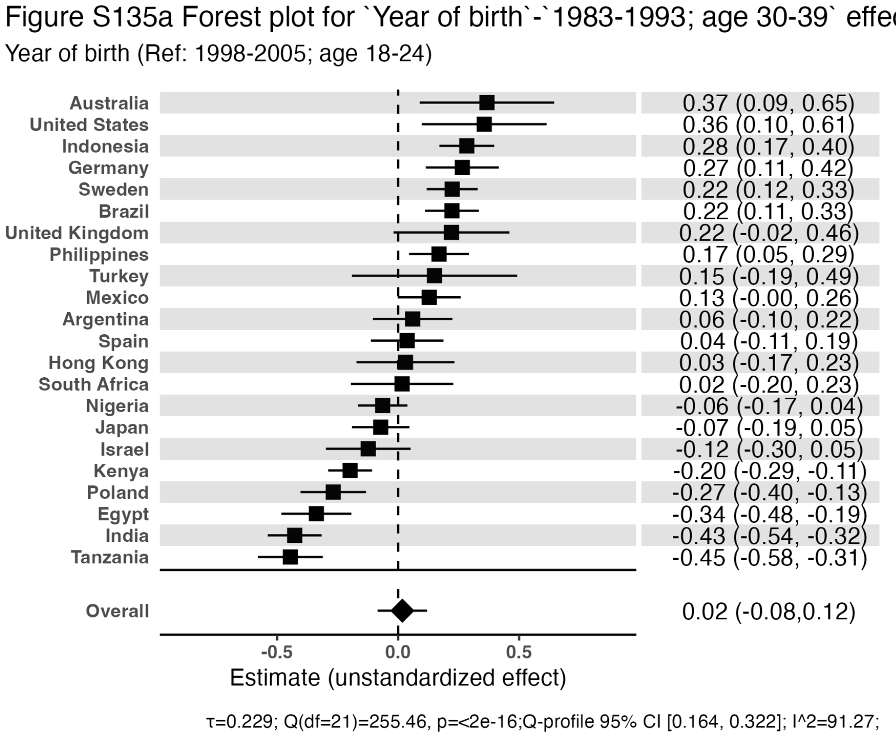

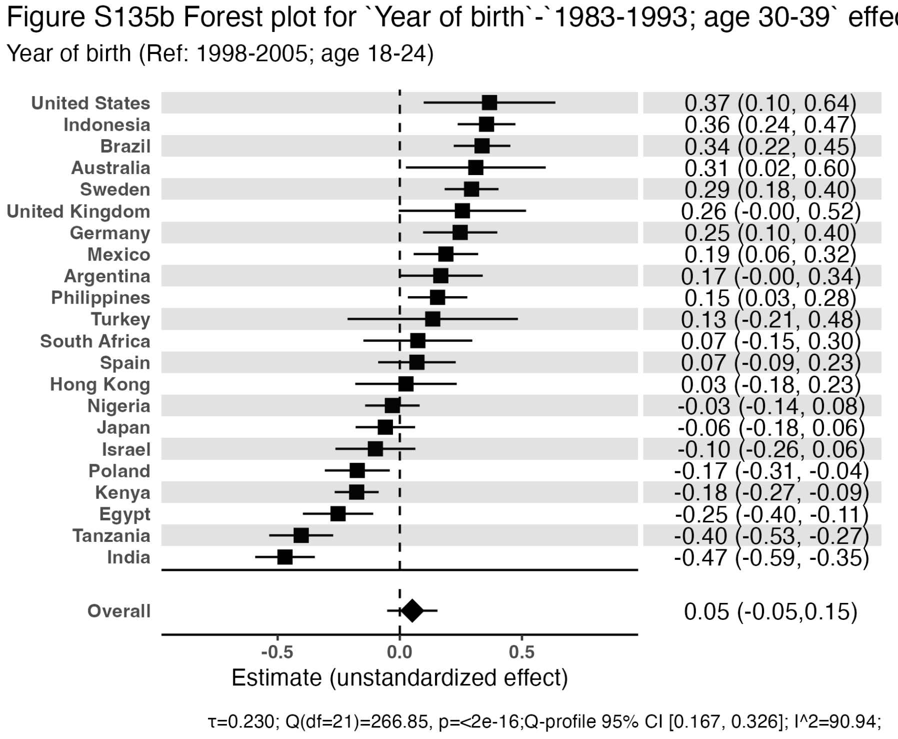

Figure S136. Heterogeneity in pairwise comparisons across countries Year of birth (Ref: 1998-2005; age 18-24) - 1973-1983; age 40-49 effect. (a) Flourishing with financial indicators (12 items) [left panel]; (b) Flourishing without financial indicators (10 items) [right panel]. N=202,898, subgroup means and standard errors are computed accounting for the complex sampling design using all data simultaneously. Analyses conducted: Random-effects meta-analysis of country-specific means. Squares represent the point estimate (mean) for each country. The lines represented the +/-1.96*SE, standard error, around the mean; the overall pooled mean is represented by the diamond. The reported p-value for Q-statistics is necessarily 1-sided because of the use of the chi-squared distribution to test whether heterogeneity is greater than zero (i.e., a two-sided test is not applicable). No adjustments were made for multiple testing.

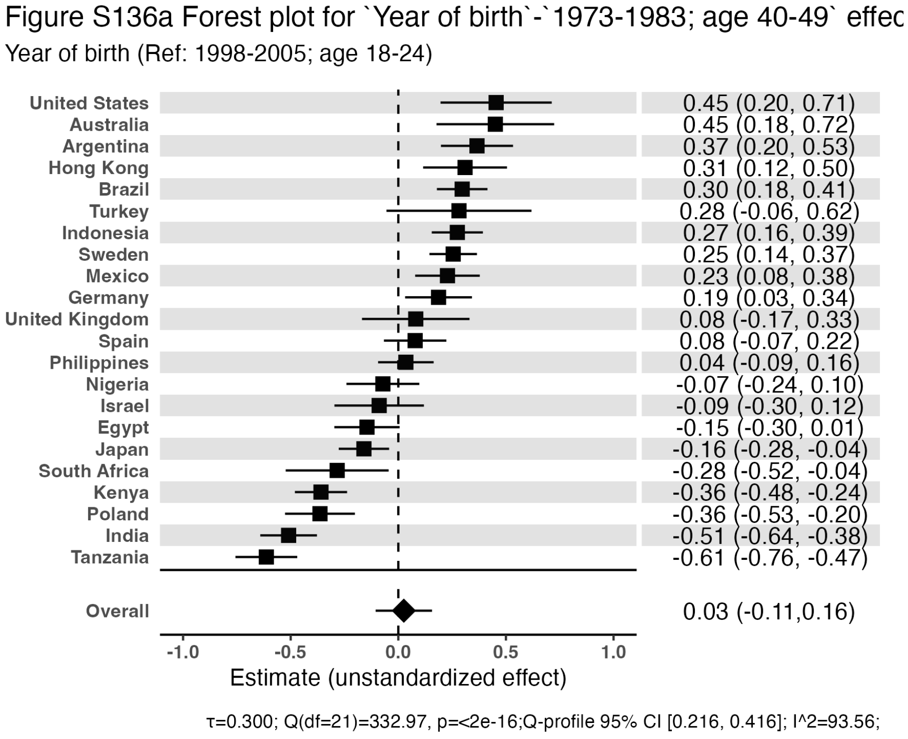

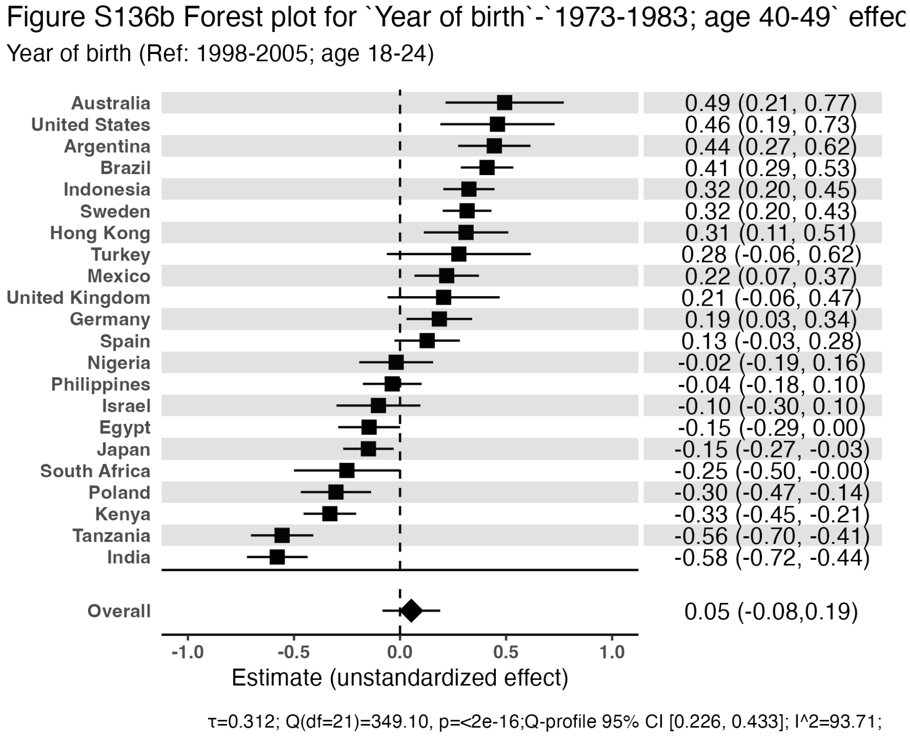

Figure S137. Heterogeneity in pairwise comparisons across countries Year of birth (Ref: 1998-2005; age 18-24) - 1963-1973; age 50-59 effect. (a) Flourishing with financial indicators (12 items) [left panel]; (b) Flourishing without financial indicators (10 items) [right panel]. N=202,898, subgroup means and standard errors are computed accounting for the complex sampling design using all data simultaneously. Analyses conducted: Random-effects meta-analysis of country-specific means. Squares represent the point estimate (mean) for each country. The lines represented the +/-1.96*SE, standard error, around the mean; the overall pooled mean is represented by the diamond. The reported p-value for Q-statistics is necessarily 1-sided because of the use of the chi-squared distribution to test whether heterogeneity is greater than zero (i.e., a two-sided test is not applicable). No adjustments were made for multiple testing.

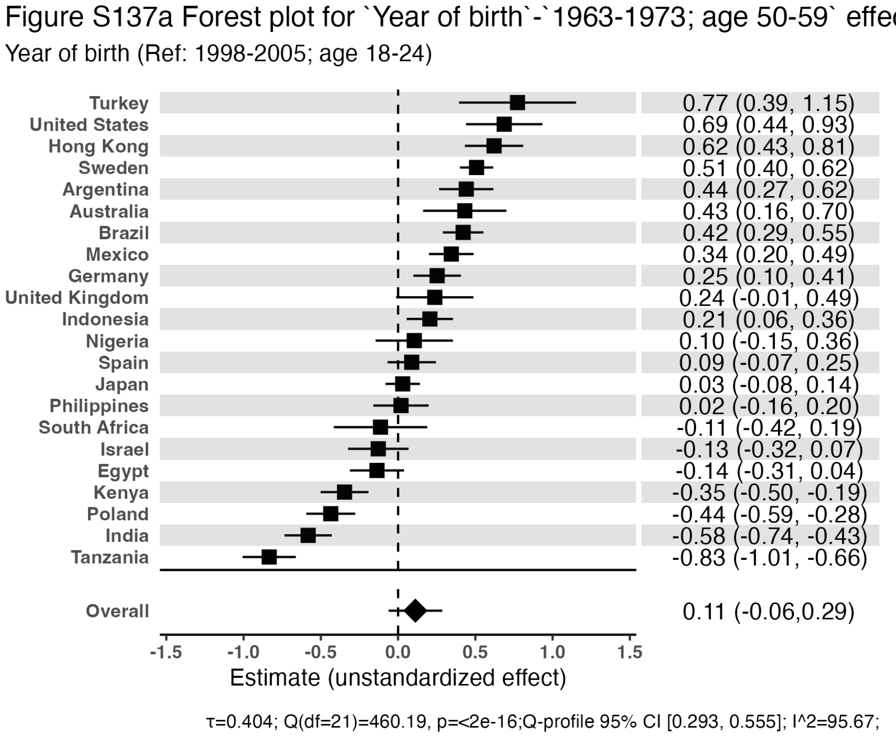

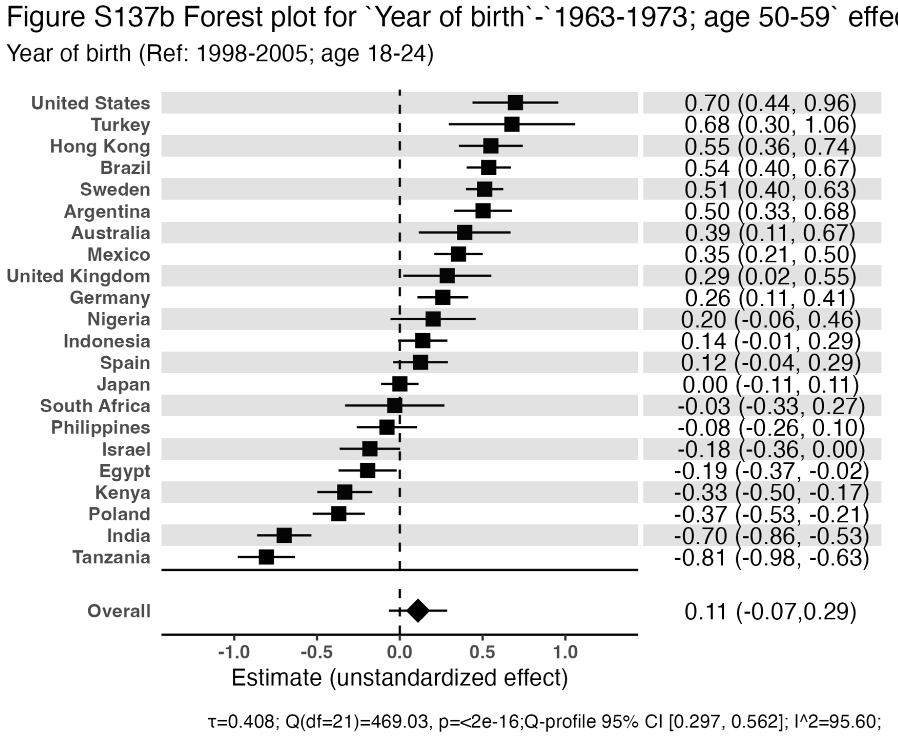

Figure S138. Heterogeneity in pairwise comparisons across countries Year of birth (Ref: 1998-2005; age 18-24) - 1953-1963; age 60-69 effect. (a) Flourishing with financial indicators (12 items) [left panel]; (b) Flourishing without financial indicators (10 items) [right panel]. N=202,898, subgroup means and standard errors are computed accounting for the complex sampling design using all data simultaneously. Analyses conducted: Random-effects meta-analysis of country-specific means. Squares represent the point estimate (mean) for each country. The lines represented the +/-1.96*SE, standard error, around the mean; the overall pooled mean is represented by the diamond. The reported p-value for Q-statistics is necessarily 1-sided because of the use of the chi-squared distribution to test whether heterogeneity is greater than zero (i.e., a two-sided test is not applicable). No adjustments were made for multiple testing.

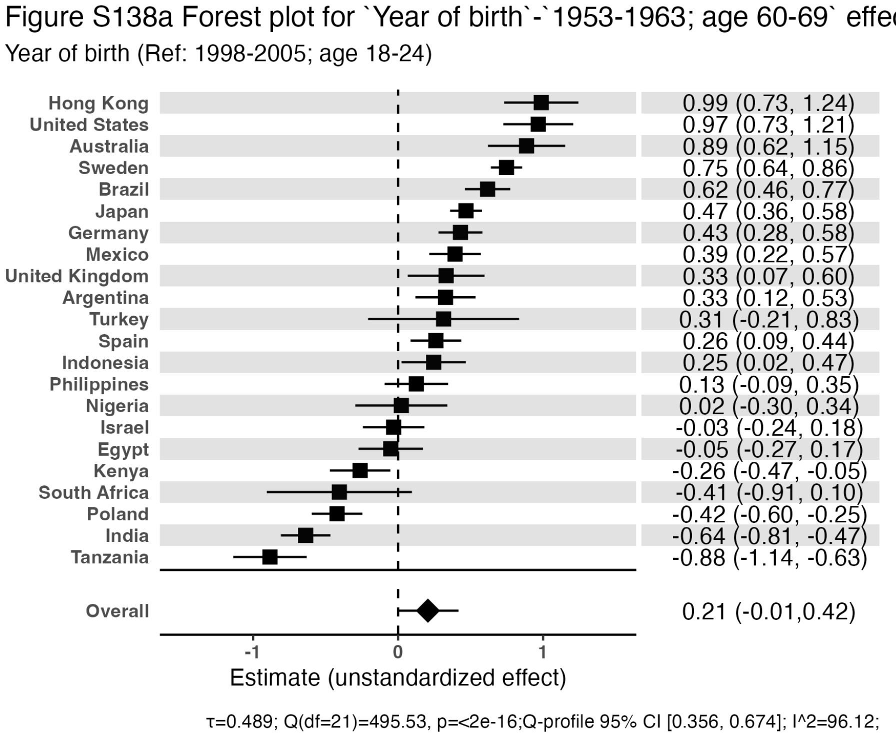

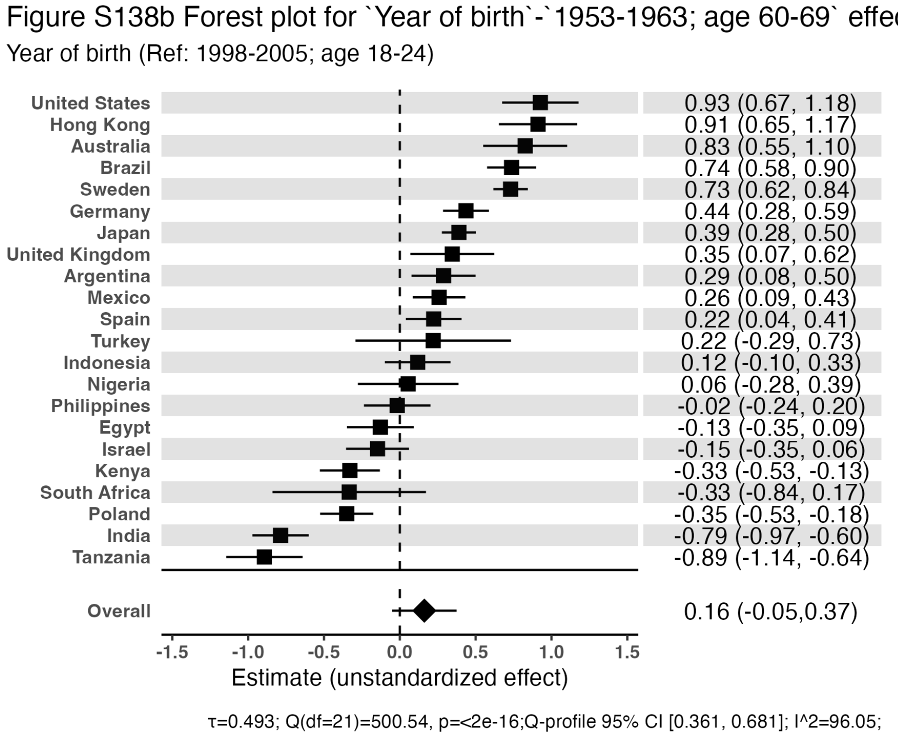

Figure S139. Heterogeneity in pairwise comparisons across countries Year of birth (Ref: 1998-2005; age 18-24) - 1943-1953; age 70-79 effect. (a) Flourishing with financial indicators (12 items) [left panel]; (b) Flourishing without financial indicators (10 items) [right panel]. N=202,898, subgroup means and standard errors are computed accounting for the complex sampling design using all data simultaneously. Analyses conducted: Random-effects meta-analysis of country-specific means. Squares represent the point estimate (mean) for each country. The lines represented the +/-1.96*SE, standard error, around the mean; the overall pooled mean is represented by the diamond. The reported p-value for Q-statistics is necessarily 1-sided because of the use of the chi-squared distribution to test whether heterogeneity is greater than zero (i.e., a two-sided test is not applicable). No adjustments were made for multiple testing.

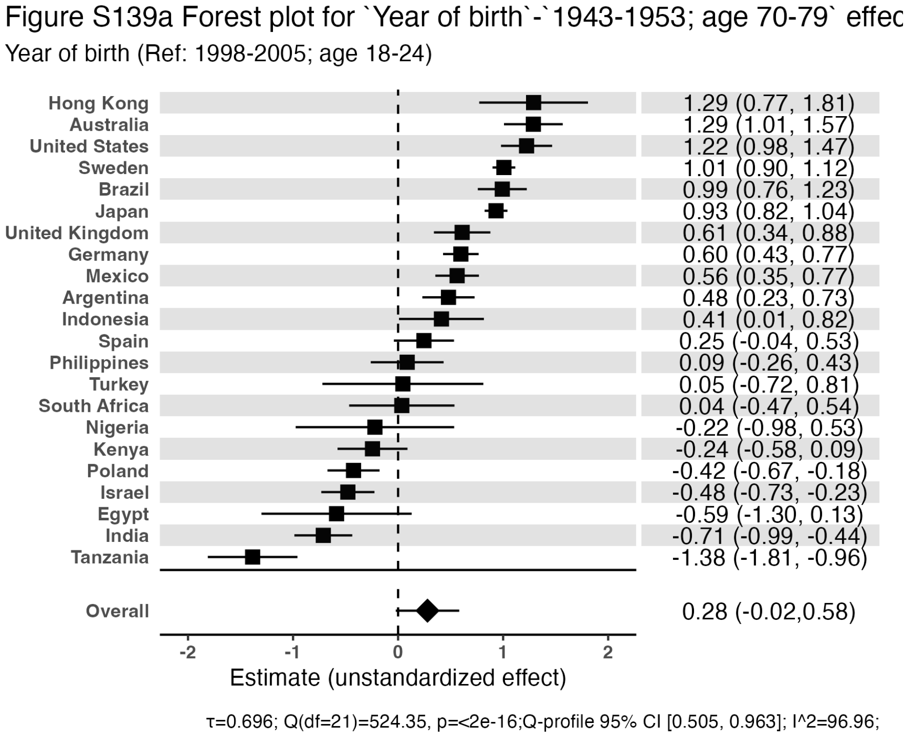

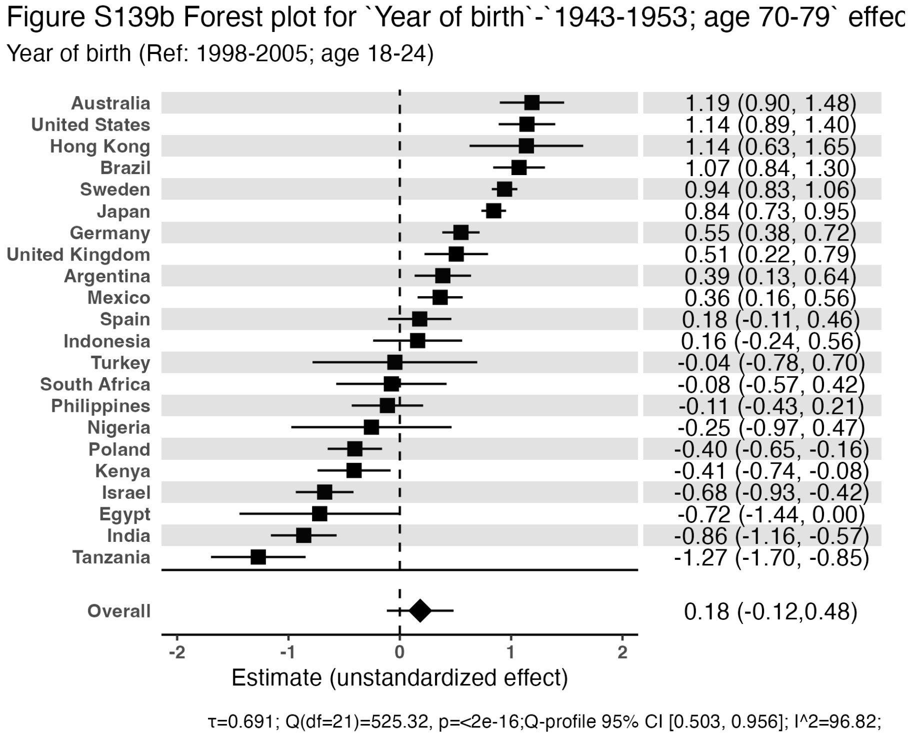

Figure S140. Heterogeneity in pairwise comparisons across countries Year of birth (Ref: 1998-2005; age 18-24) - 1943 or earlier; age 80+ effect. (a) Flourishing with financial indicators (12 items) [left panel]; (b) Flourishing without financial indicators (10 items) [right panel]. N=202,898, subgroup means and standard errors are computed accounting for the complex sampling design using all data simultaneously. Analyses conducted: Random-effects meta-analysis of country-specific means. Squares represent the point estimate (mean) for each country. The lines represented the +/-1.96*SE, standard error, around the mean; the overall pooled mean is represented by the diamond. The reported p-value for Q-statistics is necessarily 1-sided because of the use of the chi-squared distribution to test whether heterogeneity is greater than zero (i.e., a two-sided test is not applicable). No adjustments were made for multiple testing.

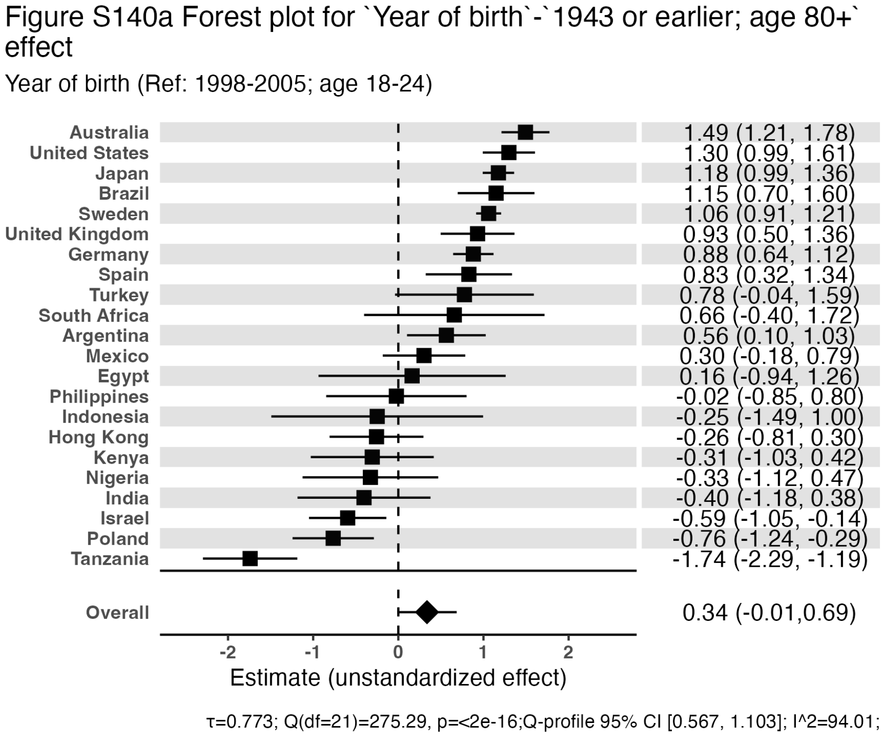

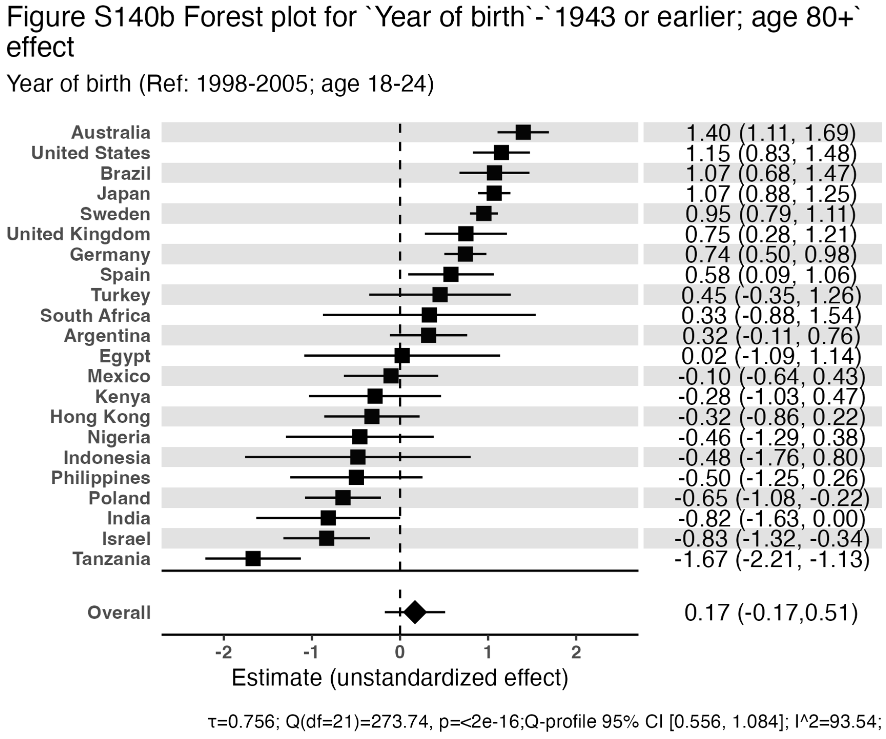

Figure S141. Heterogeneity in pairwise comparisons across countries Gender (Ref: Male) - Female effect. (a) Flourishing with financial indicators (12 items) [left panel]; (b) Flourishing without financial indicators (10 items) [right panel]. N=202,898, subgroup means and standard errors are computed accounting for the complex sampling design using all data simultaneously. Analyses conducted: Random-effects meta-analysis of country-specific means. Squares represent the point estimate (mean) for each country. The lines represented the +/-1.96*SE, standard error, around the mean; the overall pooled mean is represented by the diamond. The reported p-value for Q-statistics is necessarily 1-sided because of the use of the chi-squared distribution to test whether heterogeneity is greater than zero (i.e., a two-sided test is not applicable). No adjustments were made for multiple testing.

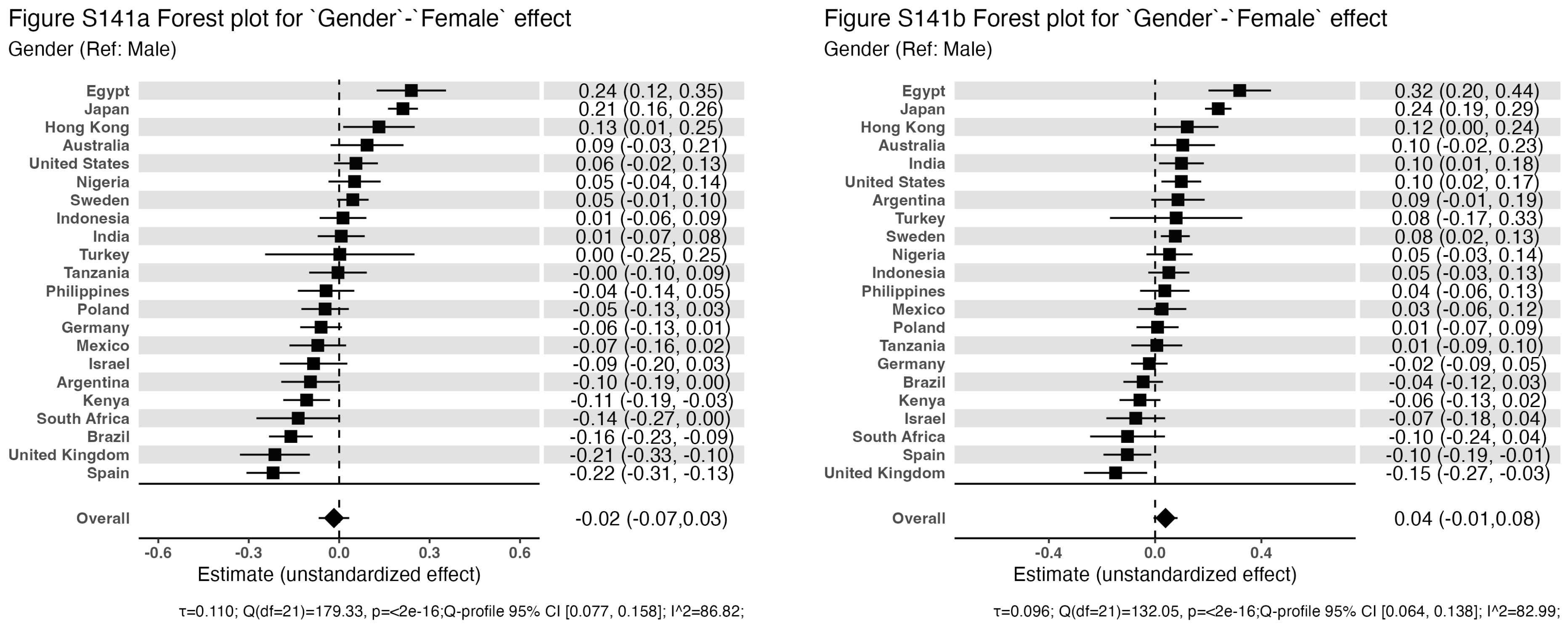

Figure S142. Heterogeneity in pairwise comparisons across countries Gender (Ref: Male) - Other effect. (a) Flourishing with financial indicators (12 items) [left panel]; (b) Flourishing without financial indicators (10 items) [right panel]. N=202,898, subgroup means and standard errors are computed accounting for the complex sampling design using all data simultaneously. Analyses conducted: Random-effects meta-analysis of country-specific means. Squares represent the point estimate (mean) for each country. The lines represented the +/-1.96*SE, standard error, around the mean; the overall pooled mean is represented by the diamond. The reported p-value for Q-statistics is necessarily 1-sided because of the use of the chi-squared distribution to test whether heterogeneity is greater than zero (i.e., a two-sided test is not applicable). No adjustments were made for multiple testing.

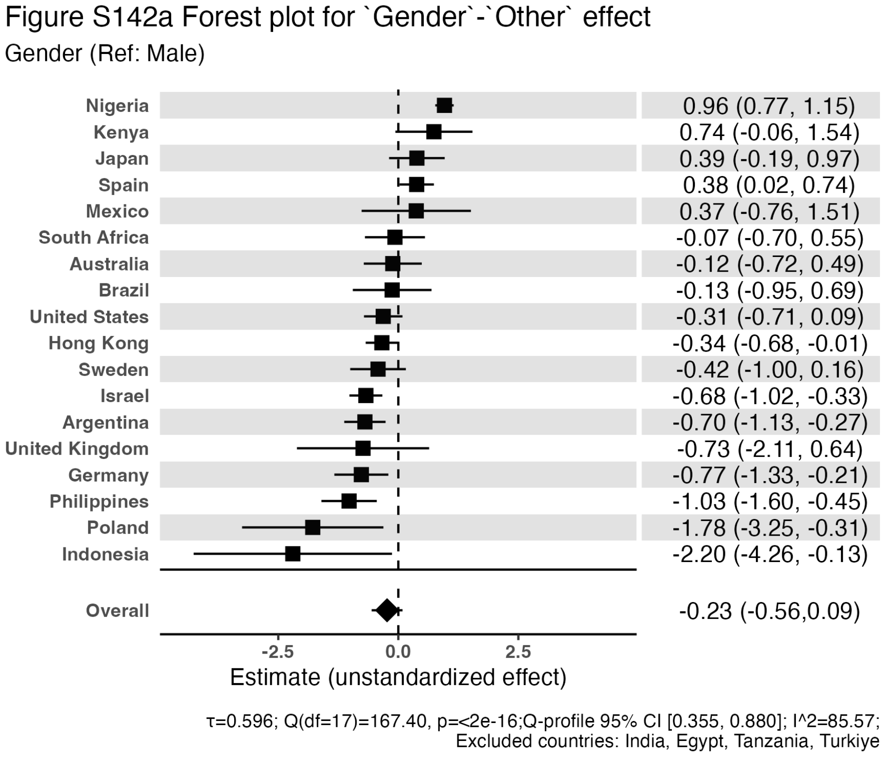

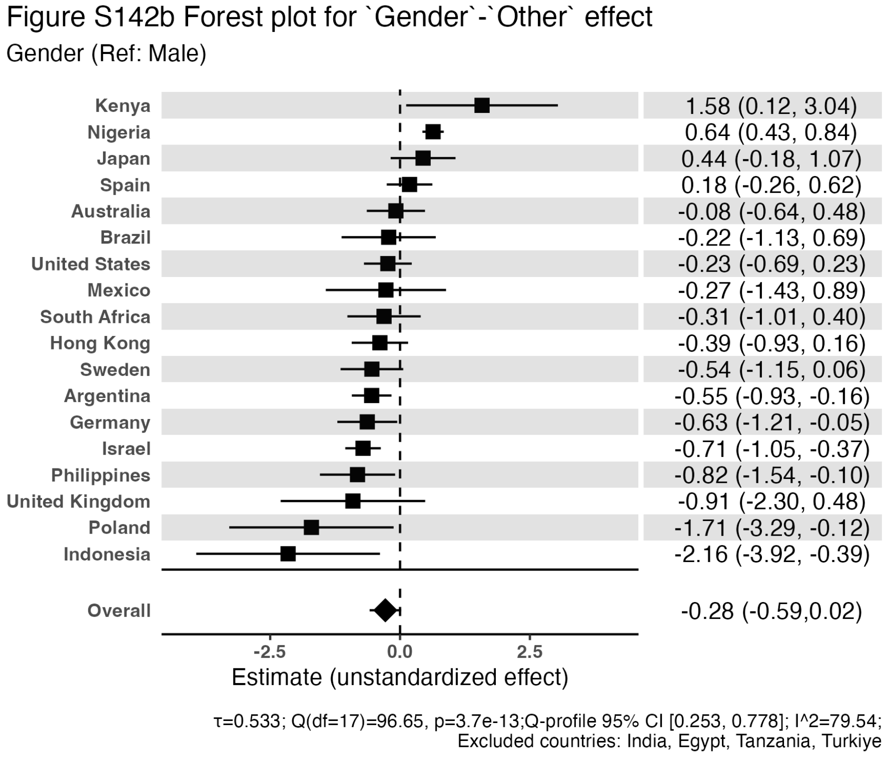

Figure S143. Heterogeneity in pairwise comparisons across countries Race_plurality - Non-plurality groups effect. (a) Flourishing with financial indicators (12 items) [left panel]; (b) Flourishing without financial indicators (10 items) [right panel]. N=202,898, subgroup means and standard errors are computed accounting for the complex sampling design using all data simultaneously. Analyses conducted: Random-effects meta-analysis of country-specific means. Squares represent the point estimate (mean) for each country. The lines represented the +/-1.96*SE, standard error, around the mean; the overall pooled mean is represented by the diamond. The reported p-value for Q-statistics is necessarily 1-sided because of the use of the chi-squared distribution to test whether heterogeneity is greater than zero (i.e., a two-sided test is not applicable). No adjustments were made for multiple testing.

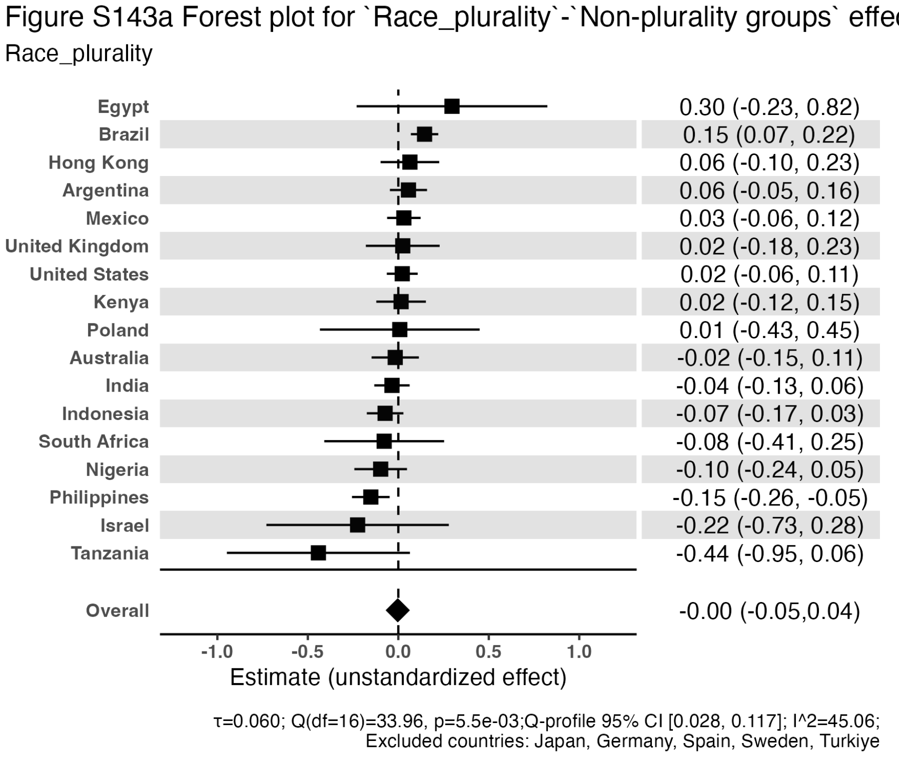

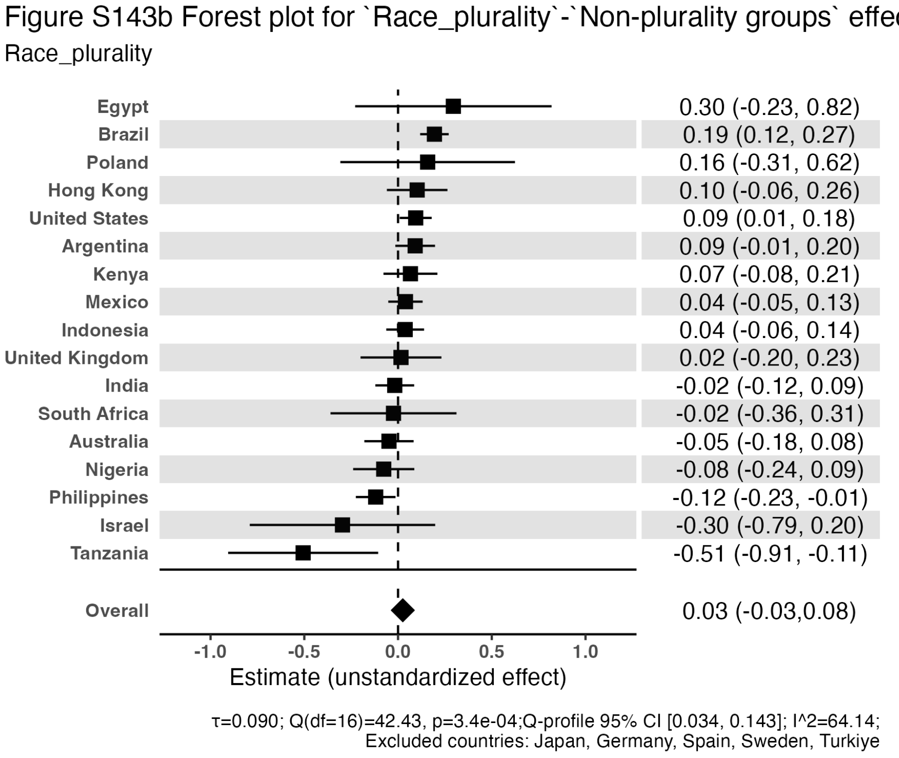

Supplement: Supplementary file 1 — Supplementary Figs. S1a–S143b and Tables S1a–S29. [file 44220_2025_423_MOESM1_ESM.pdf]
