# Supplementary material for: The Global Flourishing Study: Study Profile and Initial Results on Flourishing
Source: Nat Ment Health. 2025 Apr 30;3(6):636–53. doi: 10.1038/s44220-025-00423-5 (PMC12165845; doi:10.1038/s44220-025-00423-5)
Supplement: Supplementary file 2 — Reporting Summary [file 44220_2025_423_MOESM2_ESM.pdf]

nature portfolio

Corresponding author(s): Tyler J. VanderWeele

Last updated by author(s): Apr 2, 2025

Reporting Summary

Nature Portfolio wishes to improve the reproducibility of the work that we publish. This form provides structure for consistency and transparency in reporting. For further information on Nature Portfolio policies, see our Editorial Policies and the Editorial Policy Checklist.

Statistics

For all statistical analyses, confirm that the following items are present in the figure legend, table legend, main text, or Methods section.

n/a	Confirmed	
☐	☒	The exact sample size (*n*) for each experimental group/condition, given as a discrete number and unit of measurement
☐	☒	A statement on whether measurements were taken from distinct samples or whether the same sample was measured repeatedly
☐	☒	The statistical test(s) used AND whether they are one- or two-sided<br>*Only common tests should be described solely by name; describe more complex techniques in the Methods section.*
☐	☒	A description of all covariates tested
☐	☒	A description of any assumptions or corrections, such as tests of normality and adjustment for multiple comparisons
☐	☒	A full description of the statistical parameters including central tendency (e.g. means) or other basic estimates (e.g. regression coefficient) AND variation (e.g. standard deviation) or associated estimates of uncertainty (e.g. confidence intervals)
☐	☒	For null hypothesis testing, the test statistic (e.g. *F*, *t*, *r*) with confidence intervals, effect sizes, degrees of freedom and *P* value noted<br>*Give P values as exact values whenever suitable.*
☒	☐	For Bayesian analysis, information on the choice of priors and Markov chain Monte Carlo settings
☐	☒	For hierarchical and complex designs, identification of the appropriate level for tests and full reporting of outcomes
☐	☒	Estimates of effect sizes (e.g. Cohen's *d*, Pearson's *r*), indicating how they were calculated

Our web collection on statistics for biologists contains articles on many of the points above.

Software and code

Policy information about availability of computer code

Data collection	Data collection was conducted by Gallup, Inc. and details regarding data collection procedures are reported in the methodology documentation at https://osf.io/k2s7u.
Data analysis	All analyses conducted were generally standard and implemented in various software. Code to reproduce analysis results is provided in R (https://www.r-project.org/), SAS (https://www.sas.com/), and Stata (https://www.stata.com/). Code and documentation for use are provided through the Center for Open Science in the public repository [https://doi.org/10.17605/OSF.IO/VBYPE] with instructions for use.

For manuscripts utilizing custom algorithms or software that are central to the research but not yet described in published literature, software must be made available to editors and reviewers. We strongly encourage code deposition in a community repository (e.g. GitHub). See the Nature Portfolio guidelines for submitting code & software for further information.

Data

Policy information about availability of data

All manuscripts must include a data availability statement. This statement should provide the following information, where applicable:

- Accession codes, unique identifiers, or web links for publicly available datasets
- A description of any restrictions on data availability
- For clinical datasets or third party data, please ensure that the statement adheres to our policy

Data for Wave 1 of the GFS is available through the Center for Open Science upon submission of a pre-registration and will be openly available without pre-

registration beginning February 2025. Subsequent waves of the GFS will similarly be made available. Please see https://www.cos.io/gfs-access-data for more information about data access.

Research involving human participants, their data, or biological material

Policy information about studies with human participants or human data. See also policy information about sex, gender (identity/presentation), and sexual orientation and race, ethnicity and racism.

Reporting on sex and gender	In the survey participants were asked, "What is your gender?", ~with the response options being male (49% of participants), female (51%), or other (0.3%). In the result, we report random effects meta-analysis of flourishing means by demographic category, including in relation to gender.
Reporting on race, ethnicity, or other socially relevant groupings	Religious tradition/affiliation was captured with the categories of Christianity, Islam, Hinduism, Buddhism, Judaism, Sikhism, Baha'i, Jainism, Shinto, Taoism, Confucianism, Primal/Animist/Folk religion, Spiritism, African-Derived, some other religion, or no religion/atheist/agnostic; precise response categories varied by country. Racial/ethnic identity was assessed in some, but not all, countries, with response categories varying by country. The selection of these categories was made by Gallup on the basis of their extensive experience conducting surveys in the 22 countries involved in the study (in the context of conducting the Gallup World Poll in up to 160 countries annually since 2005.
Population characteristics	Age: 18-24 = 27,007 (13%); 25-29 = 20,700 (10%); 30-39 = 40,256 (20%); 40-49 = 34,464 (17%); 50-59 = 31,793 (16%); 60-69 = 27,763 (14%); 70-79 = 16,776 (8.3%); 80 or older = 4,119 (2.0%); Missing = 20 (<0.1%).<br><br>Gender: Male = 98,411 (49%); Female = 103,488 (51%); Other = 602 (0.3%); Missing = 397 (0.2%).<br><br>Current Marital status: Married = 107,354 (53%); Separated = 5,195 (2.6%); Divorced = 11,654 (5.7%); Widowed = 9,823 (4.8%); Single, never married = 52,115 (26%); Domestic Partner = 14,931 (7.4%); Missing = 1,826 (0.9%).<br><br>Employment status: Employed for an employer = 78,815 (39%); Self-employed = 36,362 (18%); Retired = 29,303 (14%); Student = 10,726 (5.3%); Homemaker = 21,677 (11%); Unemployed and looking for a job = 16,790 (8.3%); None of these/ Other = 8,431 (4.2%); Missing = 793 (0.4%).<br><br>Current Religious service attendance: More than 1/week = 26,537 (13%); 1/week = 39,157 (19%); 1-3/month = 19,749 (9.7%); A few times a year = 41,436 (20%); Never = 75,297 (37%); Missing = 722 (0.4%).<br><br>Education: Up to 8 years = 45,078 (22%); 9-15 years = 115,097 (57%); 16+ years = 42,578 (21%); Missing = 146 (<0.1%).<br><br>Immigration status: Born in this country = 190,998 (94%); Born in another country = 9,791 (4.8%). Missing = 2,110 (1.0%).<br><br>Country: Argentina = 6,724 (3.3%); Australia = 3,844 (1.9%); Brazil = 13,204 (6.5%); Egypt = 4,729 (2.3%); Germany = 9,506 (4.7%); Hong Kong (S.A.R. of China) = 3,012 (1.5%); India = 12,765 (6.3%); Indonesia = 6,992 (3.4%); Israel = 3,669 (1.8%); Japan = 20,543 (10%); Kenya = 11,389 (5.6%); Mexico = 5,776 (2.8%); Nigeria = 6,827 (3.4%); Philippines = 5,292 (2.6%); Poland = 10,389 (5.1%); South Africa = 2,651 (1.3%); Spain = 6,290 (3.1%); Sweden = 15,068 (7.4%); Tanzania = 9,075 (4.5%); Turkey = 1,473 (0.7%); United Kingdom = 5,368 (2.6%); United States = 38,312 (19%).<br><br>Participants provided informed consent to participate. Participants were generally compensated $3 - $6 USD depending on the country.
Recruitment	Recruitment was overseen by Gallup, and involved specialized recruitment partners in each of the countries involved.
Ethics oversight	Gallup approved the study protocol.

Note that full information on the approval of the study protocol must also be provided in the manuscript.

Field-specific reporting

Please select the one below that is the best fit for your research. If you are not sure, read the appropriate sections before making your selection.

☐ Life sciences ☒ Behavioural & social sciences ☐ Ecological, evolutionary & environmental sciences

For a reference copy of the document with all sections, see nature.com/documents/nr-reporting-summary-flat.pdf

Behavioural & social sciences study design

All studies must disclose on these points even when the disclosure is negative.

Study description	The Global Flourishing Study is a longitudinal panel study of over 200,000 participants in 22 geographically and culturally diverse countries, spanning all six populated continents, with nationally representative sampling, and intended annual longitudinal panel data collection for five years on numerous aspects of flourishing and its determinants. Details of the overall methodology are available at https://osf.io/k2s7u.
Research sample	Wave 1 of the Global Flourishing Study data involved 202,898 participants from the following countries and territories: Argentina,

Research sample	Australia, Brazil, Egypt, Germany, Hong Kong (Special Administrative Region of China), India, Indonesia, Israel, Japan, Kenya, Mexico, Nigeria, the Philippines, Poland, South Africa, Spain, Sweden, Tanzania, Turkey, United Kingdom, and the United States. The countries were selected to (a) maximize coverage of the world's population, (b) ensure geographic, cultural, and religious diversity, and (c) prioritize feasibility and existing data collection infrastructure.
Sampling strategy	Three major sampling frames were used for recruitment in the GFS: a probability-based sample, a non-probability-based sample, or a combination of the two. A probability-based sampling approach was used in Egypt, India, Indonesia, Israel, Kenya, Nigeria, Philippines, South Africa, Tanzania, Turkey, and the United States. A non-probability-based sample was recruited in some countries to supplement probability samples so that adequate coverage of population subgroups (i.e., sex, age, region) was achieved. Recruitment and empanelment for Wave 1 of the study occurred between April 2022 and December 2023. Details of data collection are available at https://doi.org/10.1007/s10654-024-01167-9 (preprint available: https://doi.org/10.31234/osf.io/yuc4q). Details of the overall methodology are available at https://osf.io/k2s7u
Data collection	The Global Flourishing Study involves a questionnaire featuring 109 items relating to various aspects of human flourishing, together with relevant demographic items, comprising a one-off intake survey of 43 items and an annual survey of 71 items, with five items shared by both. Details of the questionnaire are available at https://Osf.Io/36hry?View_only=0372838c315d46a995c122f9c637ae5d. The mode of data collection varied by country. While some countries relied entirely on web-based participation (e.g., United States), other countries exclusively used face-to-face participation (e.g., Israel, Kenya) or a mix of both (e.g., Argentina, Mexico). Details of data collection are available at https://doi.org/10.1007/s10654-024-01167-9 (preprint available: https://doi.org/10.31234/osf.io/yuc4q). Details of the overall methodology are available at https://osf.io/k2s7u
Timing	Argentina: 11/29/22 – 11/30/23 (probability sampling) and 4/11/23 – 11/14/23 (non-probability sampling). Australia: 3/21/22 – 9/26/23 (probability) and 4/12/23 – 9/26/23 (non-probability). Brazil: 11/23/22 – 11/26/23 (probability) and 4/11/23 -11/7/23 (non-probability). Egypt: 3/9/23 – 9/17/23 (probability). Germany: 8/11/22 – 11/16/23 (probability) and 6/12/23 – 8/27/23 (non-probability). Hong Kong: 10/10/23 -11/24/23 (probability). India: 4/25/23 – 12/8/23 (probability). Indonesia :11/7/23 – 12/27/23 (probability). Israel: 11/7/22 – 11/23/23 (probability). Japan: 12/13/22 – 6/30/23 (non-probability). Kenya: 4/13/23 – 11/21/23 (probability). Mexico :10/29/22 – 12/13/22 (probability) and 5/9/23 – 11/20/23 (non-probability). Nigeria: 5/16/23 – 11/7/23 (probability). Philippines: 4/4/23 – 1/5/24 (probability). Poland: 12/14/22 – 10/13/23 (probability) and 6/10/23 – 10/16/23 (non-probability). South Africa: 2/26/23 – 12/8/23 (probability). Spain: 8/17/22 – 11/8/23 (probability) and 6/22/23 – 8/30/23 (non-probability). Sweden: 1/16/23 – 2/22/23 (non-probability). Tanzania: 2/17/23 – 11/30/23 (probability). Turkey: 4/15/23 – 1/15/24 (probability). United Kingdom: 4/6/22 – 11/20/23 (probability) and 6/12/23 – 8/30/23 (non-probability). United States: 8/4/22 – 4/4/23 (probability).
Data exclusions	No data were excluded from analysis.
Non-participation	Intake survey response rates (only available for probability sampling frames): Argentina (Probability = 10.5%); Australia (Probability = 2.4%); Brazil (Probability = 13.3%); Egypt (Probability = 55%); Germany (Probability = 2.5%); India (Probability = 68.9%); Indonesia (Probability = 54.6%); Israel (Probability = 55.8%); Kenya (Probability = 63.7%); Mexico (Probability = 20.5%); Nigeria (Probability = 79.2%); Philippines (Probability = 42.6%); Poland (Probability = 71.1%); South Africa (Probability = 82%); Spain (Probability = 3.6%); Tanzania (Probability = 70.9%); Turkey (Probability = 19.2%); United Kingdom (Probability = 1.8%).<br><br>Annual survey response rates: Argentina (Probability = 41.3%, Non-probability = 97.9%); Australia (Probability = 61%, Non-probability = 69%); Brazil (Probability = 37%, Non-probability = 99.7%), Egypt (Probability = 63.1%); Germany (Probability = 41%, Non-probability = 97.8%); Hong Kong (Non-probability = 100%);I ndia (Probability = 44.6%); Indonesia (Probability = 59.2%); Israel (Probability = 66.6%, Non-probability = 100%); Kenya (Probability = 76.1%); Mexico (Probability = 27%, Non-probability = 95.7%); Nigeria (Probability = 49.3%); Philippines (Probability = 37.8%); Poland (Probability = 76.7%, Non-probability = 60.5%); South Africa (Probability = 24%); Spain (Probability = 42.4%, Non-probability = 96.7%); Sweden (Non-probability = 100%); Tanzania (Probability = 82.5%); Turkey (Probability = 27.2%); United Kingdom (Probability = 43.5%, Non-probability = 96%); United States (Probability = 100%).
Randomization	The study did not involve an experimental design.

Reporting for specific materials, systems and methods

We require information from authors about some types of materials, experimental systems and methods used in many studies. Here, indicate whether each material, system or method listed is relevant to your study. If you are not sure if a list item applies to your research, read the appropriate section before selecting a response.

Materials & experimental systems

n/a	Involved in the study	
☒	☐	Antibodies
☒	☐	Eukaryotic cell lines
☒	☐	Palaeontology and archaeology
☒	☐	Animals and other organisms
☒	☐	Clinical data
☒	☐	Dual use research of concern
☒	☐	Plants

Methods

n/a	Involved in the study	
☒	☐	ChIP-seq
☒	☐	Flow cytometry
☒	☐	MRI-based neuroimaging

Plants

Seed stocks

Report on the source of all seed stocks or other plant material used. If applicable, state the seed stock centre and catalogue number. If plant specimens were collected from the field, describe the collection location, date and sampling procedures.

Novel plant genotypes

Describe the methods by which all novel plant genotypes were produced. This includes those generated by transgenic approaches, gene editing, chemical/radiation-based mutagenesis and hybridization. For transgenic lines, describe the transformation method, the number of independent lines analyzed and the generation upon which experiments were performed. For gene-edited lines, describe the editor used, the endogenous sequence targeted for editing, the targeting guide RNA sequence (if applicable) and how the editor was applied.

Authentication

Describe any authentication procedures for each seed stock used or novel genotype generated. Describe any experiments used to assess the effect of a mutation and, where applicable, how potential secondary effects (e.g. second site T-DNA insertions, mosiacism, off-target gene editing) were examined.
